# Ontology based molecular signatures for immune cell types via gene expression analysis

**DOI:** 10.1186/1471-2105-14-263

**Published:** 2013-08-30

**Authors:** Terrence F Meehan, Nicole A Vasilevsky, Christopher J Mungall, David S Dougall, Melissa A Haendel, Judith A Blake, Alexander D Diehl

**Affiliations:** 1 European Molecular Biology Laboratory, European Bioinformatics Institute, Wellcome Trust Genome Campus, Hinxton, Cambridge CB10 1SD, UK; 2 Ontology Development Group, Library, Oregon Health and Science University, 3181 SW Sam Jackson Park Road, Portland, Oregon 97239, USA; 3 Genomics Division, Lawrence Berkeley National Laboratory, 1 Cyclotron Road, Berkeley, CA 94720, USA; 4 Baylor Institute for Immunology Research, Dallas, TX 75204, USA; 5 Bioinformatics and Computational Biology, The Jackson Laboratory, 600 Main Street, Bar Harbor, ME 04609, USA; 6 Department of Neurology, University at Buffalo School of Medicine and Biomedical Sciences, 100 High Street, Buffalo, NY 14203, USA

## Abstract

**Background:**

New technologies are focusing on characterizing cell types to better understand their heterogeneity. With large volumes of cellular data being generated, innovative methods are needed to structure the resulting data analyses. Here, we describe an ‘Ontologically BAsed Molecular Signature’ (OBAMS) method that identifies novel cellular biomarkers and infers biological functions as characteristics of particular cell types. This method finds molecular signatures for immune cell types based on mapping biological samples to the Cell Ontology (CL) and navigating the space of all possible pairwise comparisons between cell types to find genes whose expression is core to a particular cell type’s identity.

**Results:**

We illustrate this ontological approach by evaluating expression data available from the Immunological Genome project (IGP) to identify unique biomarkers of mature B cell subtypes. We find that using OBAMS, candidate biomarkers can be identified at every strata of cellular identity from broad classifications to very granular. Furthermore, we show that Gene Ontology can be used to cluster cell types by shared biological processes in order to find candidate genes responsible for somatic hypermutation in germinal center B cells. Moreover, through *in silico* experiments based on this approach, we have identified genes sets that represent genes overexpressed in germinal center B cells and identify genes uniquely expressed in these B cells compared to other B cell types.

**Conclusions:**

This work demonstrates the utility of incorporating structured ontological knowledge into biological data analysis – providing a new method for defining novel biomarkers and providing an opportunity for new biological insights.

## Background

Development of new technologies for genomic research has produced an exponentially increasing amount of cell-specific data [1,2]. These technologies and applications include microarrays, next-generation sequencing, epigenetic analyses, multi-color flow cytometry, next generation mass cytometry, and large scale *in situ* histological studies. Sequencing output alone is currently doubling every nine months with efforts now underway to sequence mRNA from all major cell types, and even from single cells [3]. Elucidation of the molecular profiles of cells can help inform hypotheses and experimental designs to confirm cell functions in normal and pathological processes. Dissemination of this cellular data is largely uncoordinated, due in part to a insufficient use of a shared, structured, controlled vocabulary for cell types as core metadata across multiple resource sites. To address these issues database repositories are increasingly using ontologies to define and classify data including the use of the Cell Ontology (CL) [4].

### The Cell Ontology

The Cell Ontology is in the OBO Foundry library and represents *in vivo* cell types and currently containing over 2,000 classes [4,5]. The CL has relationships to classes from other ontologies through the use of computable definitions (i.e. “logical definitions” or “cross-products”) [6,7]. These definitions have a genus-differentia structure wherein the defined class is refined from a more general class by some differentiating characteristics. For example, a “B-1a B cell” is a type of B-1 B cell that has the CD5 glycoprotein on its cell surface. As the differentia “CD5” is represented in the Protein Ontology (PR) [8], a computable definition can then be created that states “a ‘B-1a B cell; *is_a* [type of] ‘B-1 B cell’ that *has_plasma_membrane_part* ‘T-cell surface glycoprotein CD5 (PR:000001839)’”. The CL also makes extensive use of the Gene Ontology (GO) [9] in its computable definitions, thus linking cell types to the biological processes represented in the GO. Automated reasoners use the logic of these referenced ontologies to find errors in graph structure and to automatically build a class hierarchy. Critical to this approach is to restrict the definition of a cell type to only the logically necessary and sufficient conditions needed to uniquely describe the specific cell type. If too many constraints are added, inferred relationships of interest will be missed. If too few constraints are used, then mistaken associations will be included in the automatically built hierarchy. By careful construction of these computable definitions, biological insights may be gained through the integration of findings from different areas of research as we recently demonstrated with mucosal invariant T cells [7].

Generation of computable definitions for immune cells is complicated by the variety of ways in which immune cells have been previously classified. The common practice of defining immune cell types using protein markers and biological processes poses some problems when trying to encode this knowledge in an ontology. For example, follicular B cells are often described as expressing CD23, while Bm1 B cells, a type of follicular B cell, are characterized based on a lack of CD23 expression [10]. Humans are generally able to work around such inconsistencies, but in the context of a logic-based system such as an ontology, inconsistent combinations of statements such as this are detected automatically and must be resolved before the ontology can be used to make further inferences. In the process of developing the CL, we detected a number of such inconsistencies, and the resulting ontology only includes statements that are true for all members of a class.

### Using CL in transcriptome analysis

Elimination of inconsistent statements helped us identify the ‘necessary and sufficient’ criteria needed for a cell type’s computable definition. We explored if this approach may be applied to transcriptome analysis to filter out the hundreds of genes differentially expressed in a cell type to find those core to its identity. DNA microarray and RNA-Seq technologies allow for identification of differential expression of large numbers of transcripts, and various methods have been developed for analysis of these large data sets. These methods include ANOVA [11], gene expression clustering based on mixed model procedures [12], the use of Shannon entropy to detect tissue specificity [13] and determining the null distribution of each gene’s expression to find condition-specific outliers [14]. While each method has its advantages and drawbacks, the experimentalist must understand how experimental components such as cell types relate to each other in order to reliably interpret the data. We hypothesized that mapping transcriptome samples to the CL would allow identification of genes that are consistently up- or down- regulated among all members of a cell type class. By eliminating the thousands of genes whose expression is not consistent, we could hone in on the few genes whose expression defines the cell type. This ‘Ontology BAsed Molecular Signature’ (OBAMS) approach could then be used to identify candidate cell markers and infer associated biological processes.

To develop the OBAMS approach, we used data available from the Immunological Genome Project (IGP) [15]. The IGP Consortium is performing transcriptome analysis for over a hundred murine immune cell types and disseminates this data through the Gene Expression Omnibus resource and through an online portal [1]. Users of the portal can interact with normalized data in several ways. Expression of a single gene can be viewed across many cell types by the “Gene Skyline” tool. The “Gene Constellation” tool finds genes that share a similar expression profile. Other functionality includes gene expression heat maps organized by chromosomal location or by gene family and the recently added population comparison tool. This last tool allows users to place immune cell populations into one of two groups to find differentially expressed genes between the groupings. While this tool is very useful to immunologists, the minimal organization of the cell populations used as inputs limits its functionality for those not familiar with hematopoietic cell hierarchy.

Here we describe how the CL, with its vetted structure and defined relationships, can be used in transcriptome analysis to identify genes that distinguish a cell type from other closely related cell types. By using data from the IGP project, we identify novel candidate biomarkers for B cell subtypes by overlaying a genus-differentia structure to traditional transcriptome analysis. This approach is scalable from comparisons between cell types directly sampled to broad groupings of cell types where one representative population would be difficult to isolate. OBAMS can be used to discover biomarkers and provide new biological insights into the function of diverse array of cell types. This method provides a generalized approach for identifying cell type specific molecular signatures by using an ontology as part of the data analysis.

## Results

### Using ontologies as a component of data analysis

**Figure 1:**
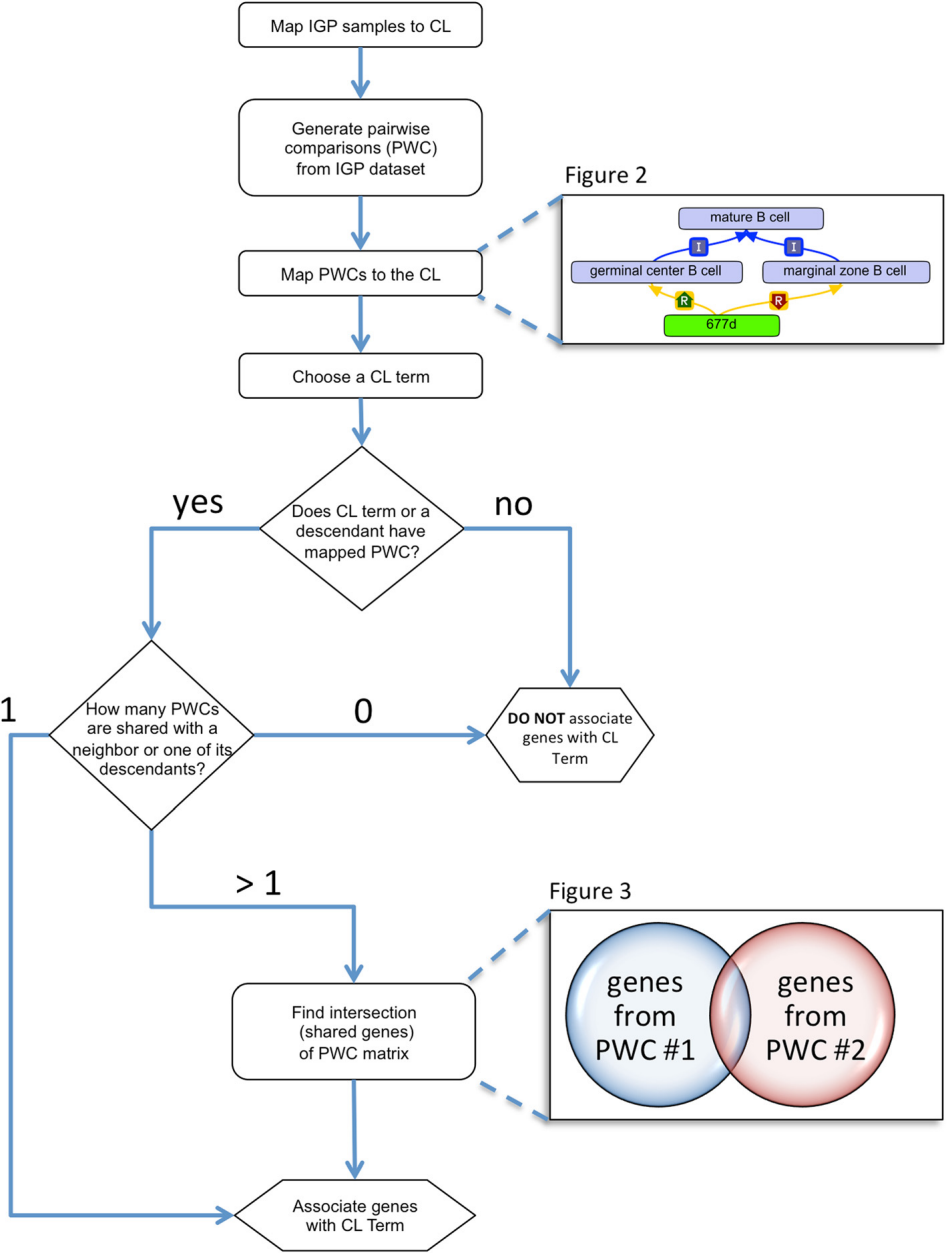
**Workflow of OBAMS project.** The project starts with a set of pairwise comparisons that are mapped to the Cell Ontology (CL) (Figure 2A). A user will check to see if a CL term of interest has a pairwise comparison mapped directly to it, or to one of its *is_a* descendants in the ontology (Figure 2B, Figure 2C). If yes, then a user determines if any pairwise comparisons are shared with their nearest neighbors (i.e. siblings) or any of their descendants. If more than one pairwise comparison exists, then shared genes are found among all the relevant gene sets (Figure 3).

**Figure 2:**
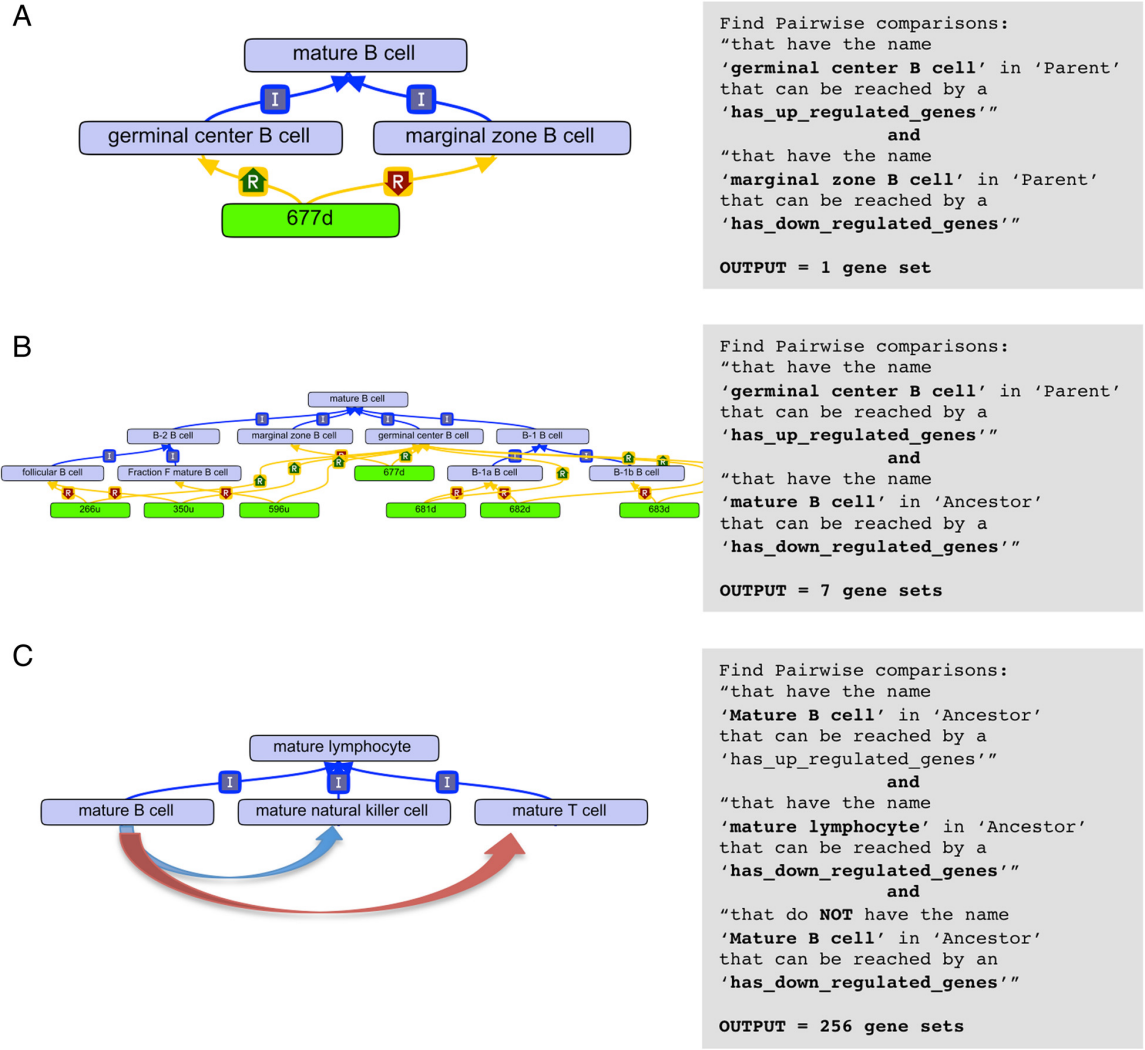
**Searching for pairwise comparisons mapped to the Cell Ontology. A)** Gene lists resulting from almost 8000 pairwise comparisons were mapped to the CL by creating terms (in green) that represent genes with 1.5 fold-higher or 1.5-fold-lower expression, between two cell types. These terms were mapped by creating *has_genes_up_regulated_for* (green R relation) and *has_genes_down_regulated_for* (red R relation) relationships. This structure allows for ontologically driven searches, depicted in text. **B)** Pairwise comparisons for genes up regulated in ‘germinal center B cell’ compared to all other mature B cells. Note how search finds the other B cell types by ‘Ancestor’ of ‘mature B cell’. **C)** Search strategy to find the 256 pairwise comparisons that exist between mature B cells and other types of mature lymphocytes. Note the third constraint that is added in the search to eliminate comparisons between types of mature B cells.

To demonstrate the utility of applying an ontological framework to analysis of a large dataset, we used the CL to perform analysis of immune cell gene expression characterized by the IGP (Figure 1). The IGP cell types included all mature myeloid and mature lymphocyte cell types for which data were available at the time of analysis (10/25/2010). A total of 88 cell types were analyzed including transitional B cell types. Pairwise comparisons were generated for all these cell types, and genes whose expression significantly differed for each cell type were identified (adjusted p-value < 0.05 after correction for multiple testing), with separate gene sets created for genes with ≥ 1.5-fold and for genes with ≤ 1.5-fold for a total of 7656 gene sets. An ontological framework was created to map these gene sets to CL classes with each gene set having one *has_up_regulated_genes_for* and one *has_down_regulated_genes_for* relationship to the two different CL classes. Thus, gene set “677d” represents the genes whose expression is up regulated in “germinal center B cell” compared to “marginal zone B cell” (Figure 2A, in green). In Figure 2A, the text box is the ontological search query used within OBO-Edit to retrieve the gene set. The query uses Boolean logic to string together two different criteria to find the relevant gene set.

By using our ontological framework, all the pairwise comparison gene sets associated with a cell type can be identified including those comparisons that are mapped to an ontological descendant of a cell type, i.e. a subtype of that cell type and further descendants through the *is_a* hierarchy. For example, “germinal center B cell” (Figure 2B) is one of 8 types of mature B cells analyzed by the IGP consortium. The simplest query to find gene sets that have “mature B cell” as an ancestor is through a search for a combination of *has_down_regulated_genes* and *is_a* relationships; the second criteria simply finds all gene sets that have *has_up_regulated_genes* directly mapped to “germinal center B cell”. In this case, this query suffices because no pairwise comparison gene sets are mapped to a descendant of germinal center B cell. When a descendant of a cell type has a gene set mapped to it, a more complex query is needed. The class “mature B cell” does not have any pairwise comparisons directly mapped to it. Instead, 8 up regulated gene sets are mapped to children and grandchildren terms of “mature B cell”. To find all the pairwise comparisons between types of mature B cells to other types of mature lymphocytes (“mature NK cell” and “mature T cell”), a third constraint is needed. The first two constraints are as described above while the third constraint eliminates any pairwise comparisons that exist between types of mature B cells. This distinction is important, as we want to include only those gene sets that compare a type of mature B cell to a type of mature NK or T cell. This query returns 256 gene sets that represent genes up regulated in a descendant of mature B cell when compared to a descendant of mature NK or T cell.

**Figure 3:**
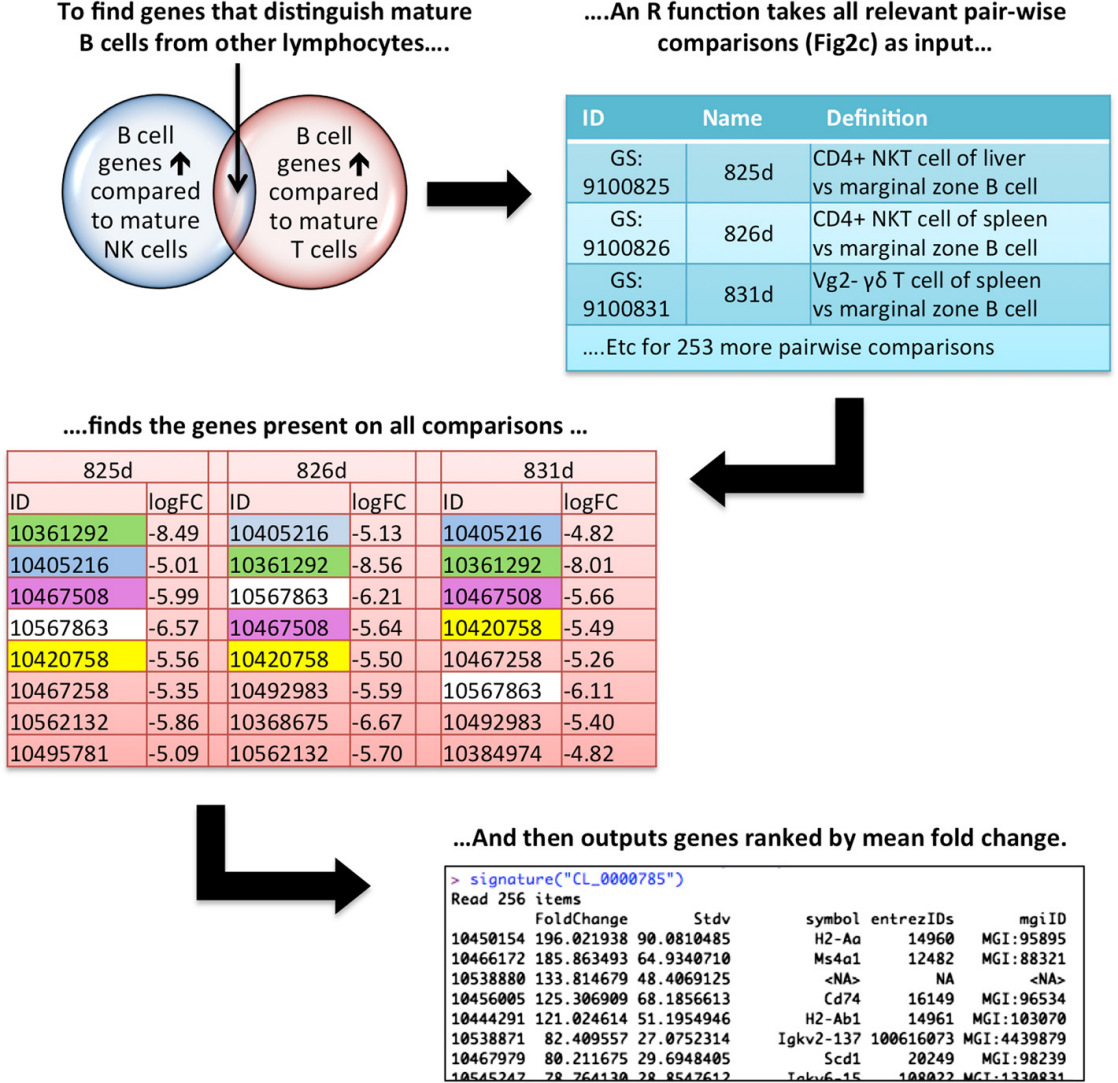
**Finding genes that distinguish mature B cells from other mature lymphocytes.** Names of the 256 pairwise comparisons identified in Figure 2C are entered into a custom R script. This script retrieves the gene lists from the file and finds common genes within all of the retrieved lists. Genes are ranked based on mean fold change across all the lists. Genes are outputted on screen (depicted) and as Excel files.

**Figure 4:**
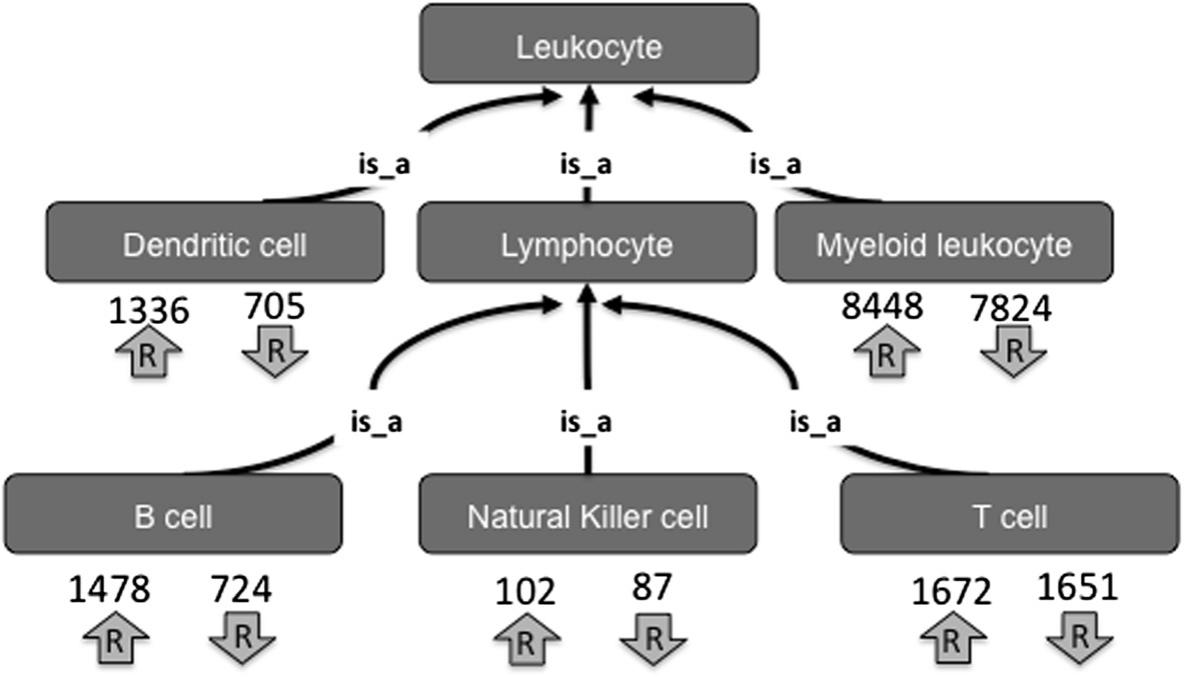
**Identification of ontology based molecular signature for leukocyte subtypes.** Gene sets (from the ImmGen Project) were mapped to immune cell types in the Cell Ontology. Pairwise comparisons were generated for all these cell types, and genes whose expression significantly differed for each cell type were identified. The numbers and arrows indicate the total number of genes that were identified as being up or down regulated among the descendants of each cell type.

This three constraint query allows for the discovery of genes that distinguish a cell type from other closely related cell types, i.e., an ontology based molecular signature (OBAMS). OBAMS reflects the structure of the CL by using a genus-differentia approach where a child class inherits all the characteristics of its parent but also contains additional characteristics that distinguishes it from its parents and any sibling terms. The differentia in this structure are represented by the intersection of gene sets returned from the three constraint query. In the mature B cell example, this intersection would contain those genes whose expression is up regulated across all descendants of mature B cells (Figure 3). By using a custom R function, gene sets can be retrieved from our ontological queries, genes within those sets can be parsed to find only those present within all the sets, and then genes can be ranked by mean fold expression. We have completed OBAMS profiles for all mature B cells, available in Additional file 1. In addition, preliminary analysis of all mature immune cells is available at the CL Immgen Data Archive [16] and summarized in Figure 4 with the total numbers of genes noted that were up or down regulated within each major immune cell branch. In summary, we are specifying sets of genes whose expression differentiates a cell type from its sibling cell types and the more general parent cell type. Because associations in an ontology are transitive, the genes associated to a general cell type are associated in every descendant cell type. For example, *Cd19* is up-regulated in “mature B cell” meaning that expression of *Cd19* is observed to be up-regulated in every mature B cell-subtype when its compared to any mature non-B cell lymphocyte (i.e. T cell and NK cell types).

### Ontological structuring of data can identify cell type specific markers

Finding new cell type specific markers can be challenging as very few transcripts are restricted to one cell type [17]. Instead, a unique combination of biomarkers must be used to isolate a functional cell type. Ontological structuring of the data verifies many of the commonly used B cell markers. For example, *Cd74* (invariant chain, Ii, MGI:96534) is overexpressed in all 256 gene sets generated by comparisons between mature B cell descendants to descendants of mature T or NK cells (Figure 3). Likewise *Cd5* (MGI:88340) is associated with B-1a B cells, *Cr2* (MGI:88489) with marginal zone B cells, and *Aicda* (MGI:1342279) within germinal center B cells (data not shown). Limits to this approach are demonstrated by the absence of some expected cell markers, such as *Spn* (MGI:98384), which encodes CD43, as a marker for all B-1 B cells. In this case, *Spn* expression is low in one subclass of B-1 B cells, that being B-1a B cells isolated from the peritoneal cavity. This could be attributed to biological reasons, such as post-transcriptional modification (which would not be observed at the transcript level) or to experimental issues (such as poor probe hybridization). Other potential issues are discussed below.

**Figure 5:**
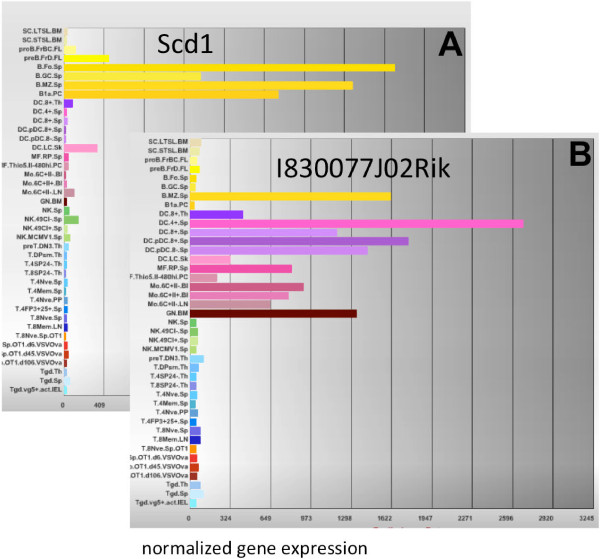
**Global expression of candidate cell marker genes. A)** Expression of Scd1, as represented for key immune cell populations at the Immunological Genome resource. B cells subtypes are labelled in yellow. **B)**. Expression of I830077J02Rik. The yellow peak is marginal zone B cell. All of the myeloid cells types also express high levels of this gene.

This hierarchical approach identifies new candidate cellular markers, especially when combined with the global expression data available at the IGP portal. For example, 205 genes are up regulated in mature B cells compared to other mature lymphocytes. Some genes like those of the MHC-II complex are widely expressed by non-lymphocyte cells. Other genes, such as *Scd1* (MGI:98239), an enzyme involved in biosynthesis of monounsaturated fatty acids [18], are highly restricted to mature B cells types (Figure 5) in comparison to the studied immune cell types included in this analysis. Similar expression patterns can be found in more granular cell types. *Satb1* (MGI:105084) is widely expressed across immune cell types, but in mature B cells there is a 10-fold upregulation in B-2 B cell compared to B-1 B cells (data not shown). Likewise, the gene *I830077J02Rik* (MGI:3588284), which is described by UniProt [19] (UniProtKB:Q3U7U4) as a single-pass transmembrane protein, but is otherwise uncharacterized, is widely expressed among myeloid cells. However, in lymphocytes, expression of this protein is restricted to marginal zone B cells (Figure 5B, B cell types in yellow, myeloid in purple). Using OBAMS, candidate biomarkers can be identified at every strata of cellular identity from broad classifications to very granular.

The transitive nature of the ontology allows us to build a gene expression profile for more granular cell types. We can infer that the B-1a B cell, when compared to other mature lymphocytes, has increased expression of the 205 genes associated with all mature B cells and the 63 genes up regulated for all B-1 B cells, in addition to the 105 genes up regulated specifically in this cell type. When gene expression of B-1a B cells is globally compared to all mature immune cells taken out of the ontological context and instead is globally compared to all mature immune cells, only three genes are confirmed to be up regulated in a statistically significant manner: *Fzd6* (MGI:108474), *Tmc3* (MGI:2669033) and *1810046K07Rik* (MGI:1917059). While of interest, very little information can be inferred about the B-1a cell type from such a limited sample, especially given that one of the genes is uncharacterized.

### Insights into cell function are gained using the gene ontology

Reflecting on the classification schemes of immunologists, the hematopoietic branch of the CL is built on a hybrid classification of cell surface markers, cell lineage, and biological function [6,7]. Although some associations between the CL and GO have been curated in the CL, this has proved a difficult task as there are large volumes of information to review and many inherent contradictions in the understanding of cell functions. We present a solution using GO term enrichment to find molecular functions or biological processes that are significantly overrepresented in a cell type’s OBAMS. From this, we then make new assertions of inferred function that are independent of experimental evidence published in the literature.

In a case example of this approach, 205 genes consistently up regulated by all mature B cell types were used as input to VLAD, an online GO term enrichment tool [20], with the result that there was an association of the gene set with a highly ranked biological process identified as “antigen processing and presentation of peptide or polysaccharide antigen via MHC class II” (GO:0002504), p-value = 5 × 10^-20^. According to this analysis, mature B cells can be differentiated from T and NK cells by their ability to present antigen to T cells in a manner similar to dendritic cells. While this function has been demonstrated experimentally [21], this association had not been captured by curators of the CL as most of the literature focuses on the production and use of immunoglobulin complexes. However, such associations are important for comprehensive representation of cell types. As a result of this computational analysis, an association has now been made in the CL between “mature B cell” and “professional antigen presenting cell” by declaring both cell types are capable of the GO process “antigen processing and presentation of peptide or polysaccharide antigen via MHC class II” (GO:0002504). Other interesting GO associations that we found through term enrichment and that are supported by evidence in the literature include the findings that B-2 B cells are in resting state compared to B-1 B cells [22] by “negative regulation of lymphocyte activation” (GO:0051250), and that marginal zone B cells are capable of “antigen processing and presentation, endogenous lipid antigen via MHC class Ib” (GO:0048006) [23]. Thus one benefit of this approach is the review of the completeness of CL representations. We performed GO term enrichment for all up regulated genes in particular mature B cell subtypes, and the results are summarized in Table 1 (and collected in Additional file 1).

**Table 1:** Summary of GO term enrichment for all up regulated genes in particular mature B cell subtypes

**B cell**	**Genes up regulated**	**Example biological process (up regulated genes)**	**GO ID**	**Genes down regulated**	**Example biological process (down regulated genes)**	**GO ID**
Mature B cell	134	Antigen processing and presentation of peptide or polysaccharide antigen via MHC class II	GO:0002504	57	Antigen receptor-mediated signaling pathway	GO:0050851
B-1a B cell	91	Anatomical structure development	GO:0048856	66	Cellular chloride ion homeostasis	GO:0030644
B-1b B cell	66	Cellular chloride ion homeostasis	GO:0030644	91	Anatomical structure development	GO:0048856
Fraction F mature B cell	2	Cellular iron ion homeostasis	GO:0006879	0		
Germinal center B cell	1160	Mitotic cell cycle	GO:0000278	524	Regulation of metabolic process	GO:0019222
Marginal zone B cell	99	Antigen processing and presentation, endogenous lipid antigen via MHC class Ib	GO:0048006	32	Regulation of phosphate metabolic process	GO:0019220
B-1 B cell	59	Activation of protein kinase C activity by G-protein coupled receptor protein signaling pathway	GO:0007205	6	Regulation of actin polymerization or depolymerization	GO:0008064
Total	1611			776		

We performed GO term enrichment for all up regulated genes in particular mature B cell subtypes. In cases where a single GO term is shown, its URI is listed.

Beyond those cell type process assertions with supporting experimental evidence, we identified other enriched GO biological processes associated with cell types that are *de novo* assertions. For example, our analysis revealed that B-1b B cells over express genes that encode mitochondrial proteins. This may support a report of a protective mechanism against apoptosis that allows B-1 B cells to survive after antigen stimulation [24]. Another interesting observation is that B-2 B cells express genes that are associated with dendrite development. A spherical shape is often associated with lymphocytes but *in vivo* imaging demonstrates this is not always case [25,26]. The dendrite development may reflect a branching morphology taken by these cells, as has been proposed for GC B cells [27]. Alternatively, genes involved in dendrite development may regulate cell migration through different tissues as suggested by expression associated with the trailing edge of cells, *Pip5k1b* (MGI:107930), and cilia, *Bbs9* (MGI:2442833) and *Rapgef4* (MGI:1917723). Novel testable hypotheses such as these can be potentially developed based on the use of ontologies to structure gene expression analysis.

### Ontologies can be used to perform ‘in silico’ experiments

**Figure 6:**
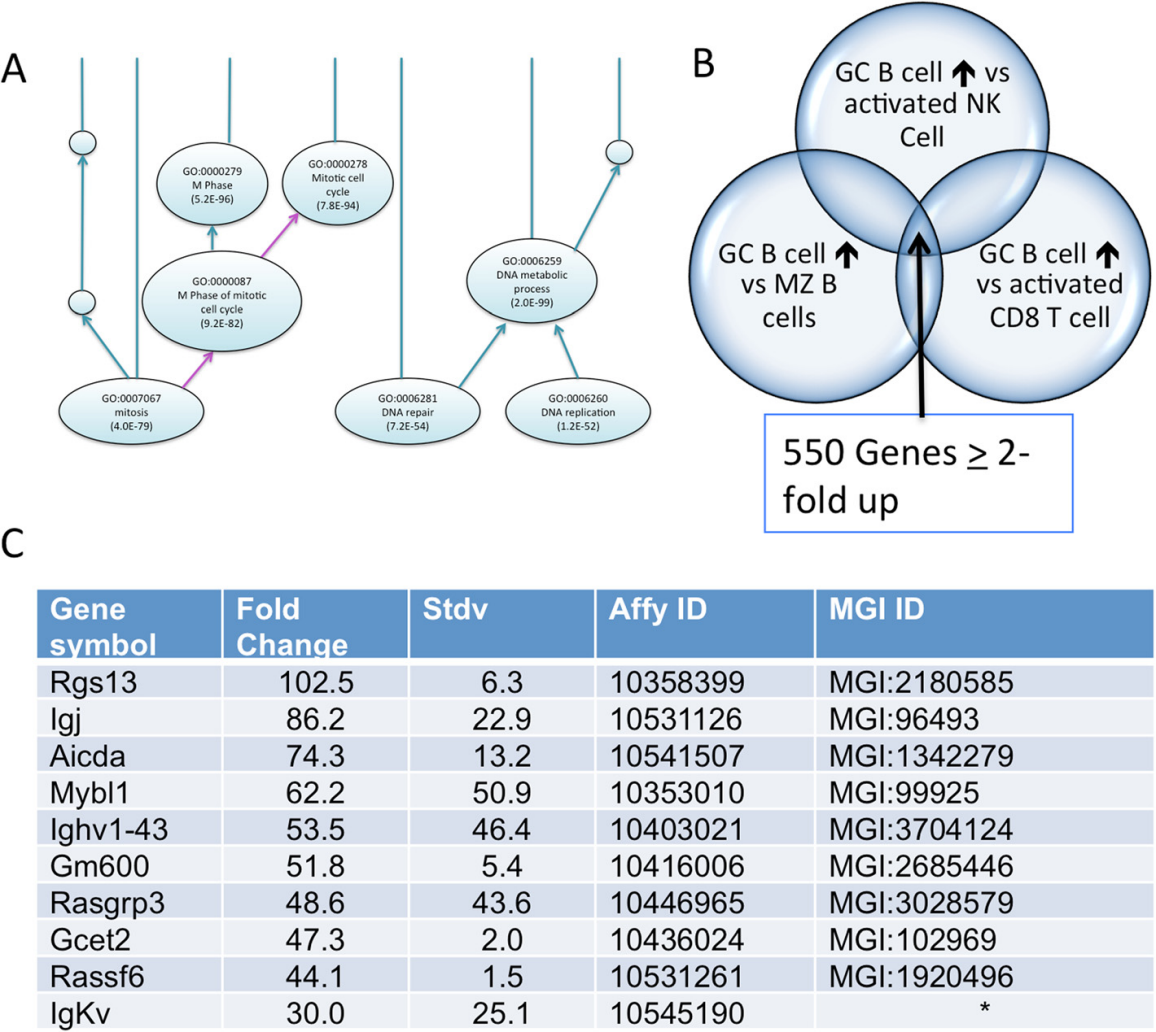
**Candidate genes involved in the unique functions of Germinal Center B cells. A)** Gene Ontology term enrichment of germinal center B cells (GC B cell) reveals genes involved in cell cycling and in the repair of DNA. **B)** DNA repair is often associated with cellular proliferation, so a new set of pairwise comparisons was generated against other proliferating lymphocytes using the methods described. Marginal zone B cells were included to eliminate genes found among B cells. **C)** Table of Top 10 genes overexpressed in GC B cells compared to activated lymphocytes and MZ B cells. * Probe maps to two different IgKappa variable segments.

GC B cells undergo somatic hypermutation of their genomic DNA, which allows for selection of immunoglobulin complexes with higher affinity for antigen. We hypothesized that the OBAMS signature for GC B cells would identify genes that regulate genomic DNA stability. However, GC B cells are also distinguished from other mature B cells by being in a proliferative state; the top ten GO enriched biological processes from their OBAMS profile involve various aspects of the cell cycle (Figure 6A). Because eukaryotic DNA replication is a flawed process that uses proofreading mechanisms during the normal sequence of events [28], we need to subtract out those genes up regulated in other proliferating immune cells to find genes that specifically regulate somatic hypermutation of GC B cells.

We initially used the ontology to find the closest neighbor to GC B cells in the IGP dataset that is activated (i.e. proliferating). At the time of analysis, activated B cell datasets were unavailable, so two other datasets were used. The first data were from mature Ly49-positive NK cells isolated from a mouse spleen, one day after infection with murine cytomegalovirus (MCMV). Since NK cells are activated after viral infection; we hypothesized that genes that regulate lymphocyte activation should be up regulated in NK cells from MCMV infected mice compared to controls [29]. The second cell type used were CD8^+^ T cells expressing a transgenic T cell receptor specific for OVA peptide (OT-I). T cells were isolated from mice at day 4 after exposure to a virus expressing the OVA peptide. Both of these cell types had high expression of the M-phase marker, *Cdk1* (MGI:88351), formerly known as *Cdc2a*, confirming these cell types were actively proliferating (data not shown). Raw data from these cell types and the controls were collected, as well as data from GC B cells and marginal zone B cells (MZ B cells). This last cell type, the MZ B cell, was added as this was the closest neighbor for which raw data were available and thus could provide a reference to remove B cell specific markers.

Using our ontological framework, we created gene sets that represented genes overexpressed in GC B cells compared to MZ B cells and activated NK and CD8^+^ T cells (Figure 6). Five hundred fifty genes were found to be up regulated by > 2-fold in this analysis; 106 of which are also found as being up regulated in GC B cells in the original analysis (Additional file 2). Several known markers of GC B cell are present in the ten most highly expressed genes, including the *Mybl1* (MGI:99925) transcription factor, the *Aicda* (MGI:1342279) gene that is responsible for deamination of cytosine that leads to point mutations in the immunoglobulin gene segments, and the *Igj* (MGI:96493), *Igk-V* (MGI:96499), and *Igh-V* (MGI:96477) gene segments involved in the class switch recombination [30,31]. Interestingly, the remaining genes are involved in the G protein coupled signaling pathways and are also implicated in cancers as discussed below.

A recent study performed by the ImmGen Consortium complements and validates our efforts [32]. The authors used network analysis to identify molecular markers for natural killer (NK) cells. Data from their study overlapped with our NK cell analysis; both their study and ours showed that NK cells encoded NK cell receptors such as *Klra8* (Ly49H) (MGI:102968) and *Ncr1* (MGI:1336212). Additionally, in both studies the genes *Adamts14* (MGI:2179942), *Serpinb9b* (MGI:894668) and *Styk1* (MGI:2141396) were identified specifically as being expressed in NK cells, which was previously unreported. In total, 74% of the 66 genes identified by OBAMS analysis overlapped with the larger 93 gene set from the ImmGen analysis. Genes identified in the former that were not in the later largely fell into one group – genes whose expression was restricted to NK cells among all lymphocytes, but were expressed in other non-lymphoid cell types. This highlights the differences in the two approaches. Genes identified in ImmGen analysis but not in the OBAMS approach fell largely into two categories – genes whose expression we excluded due to expression within a dataset not used in the network analysis, Vgamma5 GammaDelta IELs, or genes identified by the lowest scoring threshold category (see Additional file 3). Thus, the complementary approaches represented by network analysis and OBAMS can identify new biomarkers in immune cell subtypes with good reproducibility.

## Discussion

Mammalian cell types have distinct identities that reflect their diverse roles in executing the biological processes necessary for sustaining life. Finding genes defining their identity is a difficult task as genes whose expression is restricted to any one cell type are rare. Here we demonstrate a novel method of performing gene expression analysis within an ontological framework that provides new insights into understanding cellular identity. By generating pairwise comparisons within a logical hierarchy, we find genes whose expression is associated with cellular types at both a granular and a broad level. Importantly, this structure is transitive so that genes whose expression is associated with a broad level classification are also associated with all descendant cell types in the hierarchy. This allows a parsing out of gene expression to specific cell types, despite the evidence that most genes are expressed in more than one immune cell type.

Hypotheses can be developed about cellular functions based on genes associated with a cell type by OBAMS. Our approach illustrates, for example, how B cells are unique among lymphocytes in being able to present antigen by the MHC class II pathway. We find this despite wide expression of MHC Class II components in the myeloid branch of hematopoietic cells. Literature review confirms this role of B cells but other insights lack experimental evidence, and our results may initiate new avenues of research. While we noted expression of genes by B-2 B cells involved in dendrite formation as one such example, other hypotheses may be generated from unexpected OBAMS results, such as the expression of iron transport genes in Fraction-F B cells or the expression of genes that negatively regulate blood coagulation by B-1a B cells.

Ontologies can also be used in the next step: testing a hypothesis by helping to frame analysis of pre-existing data in relevant ways. In an illustration of this point, we identified genes involved in somatic hypermutation of DNA, a function used by GC B cells to generate high affinity antibodies. Identifying genes that specifically regulate this process is difficult due to the proliferative state of GC B cells and the associated DNA repair mechanisms inherent in any proliferating cell. By using the CL, we identified similar cell types that are proliferating and then subtracted any shared up regulated genes. Genes associated with B cells were also removed by excluding those genes expressed in the closely related B cell type MZ B Cells. We hypothesized the remaining genes would be enriched for those involved in somatic hypermutation of DNA.

Literature review of the identified genes from our GC B cell analysis confirmed the validity of our approach. Expression of *Aicda* and *IgJ* are considered hallmarks of GC B cells. *Nuggc* (MGI:2685446), formerly known as *Gm600*, has high similarity with the human GTPase SLIP-GC, a nuclear GTPase expressed in activation-induced deaminase-expressing lymphomas and germinal center B cells [33]. *Rgs13* (MGI:2180585) strongly impairs signaling through G-inhibitory linked signaling pathways and has been found to down regulate responsiveness of GC B cells to chemokine response [34]. The Ras activator *Rasgrp3* (MGI:3028579) promotes B cell signaling by enhancing signals from the B cell receptor complex, and is critical in the production of certain classes of immunoglobulins [35]. *Rassf6* (MGI:1920496), also a Ras-interacting protein, is implicated in inducing apoptosis in tumorigenic cells and in regulation of organ size [36-38]. This gene has not been previously described as playing a role in GC B cells. *Gcsam* (MGI:102969), formerly known as *Gcet2*, is a regulator of the RhoA signaling pathway that negatively regulates lymphocyte mobility and whose expression is associated with increased survival in at least two types of lymphoma [39,40]. In addition to involvement in G protein coupled signaling pathways, mutations of all these genes are implicated in development or progression of a number of cancers, suggesting that this approach can be used to discover chemotherapeutic targets.

### Known constraints and potential enhancements to our approach

There are constraints to this approach. First, this approach requires consistent gene expression across all the subtypes of a cell type being analyzed. For example, CD43 expression is considered a marker of B-1 B cells but was not detected as such in our analysis. This is because there are three datasets associated with descendants of this cell type; one did not have high expression of the *Spn* gene that encodes CD43. While this is a limitation in our approach, other resources such as the Gene Expression Atlas (GEA) address this issue. The GEA is a semantically enriched database of curated experiments from the Array Express transcriptome resource [41]. The GEA performs meta-analysis across multiple transcriptome assays and identifies genes that are up- or down regulated in factors that are shared between individual studies. These shared factors are represented by an application ontology known as the Experimental Factor Ontology (EFO) that includes as part of its structure portions of the CL [42]. Users can query the GEA by EFO classes such as “B cell” to find genes annotated to this term. In contrast to our approach, a gene whose expression is not consistent for all members of a class is not eliminated but rather the contrasting results are presented. Our restrictive approach serves as a compliment to the resources available at GEA.

Another issue that we have not addressed is the anatomical location of the cell types. The IGP project has generated data from the same cell type isolated from different locations such as the spleen, lymph node, bone marrow, and peritoneal cavity. For example, “Fraction-F B cell” isolated from bone marrow is described as CD21-positive, as they need to be distinguished from CD21-negative immature B cells present in this tissue. In our current schema, Fraction F B cells are compared to follicular B cells of the spleen, which have high levels of CD21. As a consequence, CD21 is not associated with Fraction F B cells. A more comprehensive ontological structuring of data will include anatomical axes of differentia by using anatomical ontologies such as UBERON [43]. Indeed, our approach is not limited to the use of the CL but is applicable to any well-structured ontology that represents classes for which transcriptome data exists. OBAMS could be used to identify overexpressed genes among a tumor type or genes whose expression changes as a tissue undergoes differentiation. Critical to this is access to robust ontologies for which samples can be mapped.

A potential limitation is a restriction in a OBAMS signature for a cell type as more data is brought in for a subtype and more pairwise comparisons are performed. Indeed as we were developing our OBAMS approach we were concerned there would only be a very few genes whose expression was consistent across all types of the higher level classes (i.e. mature B cell). This proved unfounded for mature B cell (over 200 genes or gene segments up, suppl file 1) and NK cell types (63 up-regulated, suppl file 3) with the latter having good correspondence with known cell markers and with the NK network analysis. This is in part due to the efforts of the ImmGen consortium to reduce inter-center and inter-sample variability but also reflects that cell type characteristics are tightly correlated with gene expression. This correlation is strong enough that researchers have recently deconvoluted gene expression results from complex tissues to determine the frequency of the constitutive cell types through expression of cell marker genes [44]. However, other cell types may have more variable gene expression among their subtypes, which would lead to a sub-optimal OBAMS. To address this, our OBAMS approach may be applied in other kinds of analyses, including the use of weighted gene co-expression networks or Shannon entropy [13,45,46]. These approaches have the advantage of not excluding a gene if it is not expressed in all relevant samples. A highly expressed gene present in the majority of samples with a shared trait will still be identified. In these types of transcriptome analyses, modules of genes are created based on similar expression within samples that share particular traits. Ontologies such as the CL can be used to assign traits to samples. For example, a “myeloid” or “lymphoid” trait can be assigned to all cell types analyzed in the IGP dataset. OBAMS may also be used in other types of molecular analysis where samples can be mapped to the CL. Such possibilities include mass cytometry and mapping of cellular epigenomes, both of which are suitable for high-throughput analyses and likely to generate large data volumes [47,48].

There are several guidelines that can be applied going forward to enhance the OBAMS approach. First, the samples being studied must be mapped to an appropriate ontology that has a robust representation of the biological context. An ontology with erroneous associations will lead to misleading OBAMs signatures. Samples being analyzed should be of a similar type (cell type, tissue, tumor, etc.) with appropriate efforts made to reduce variability, as our current implementation of OBAMS is sensitive to spurious expression in one sample. The scope of the samples being analyzed is important to consider. To determine an OBAMS signature for a class, representation of the major subtypes is important, but inclusion of a sample from every subtype is not critical and may not even be practical. What is critical is to have samples from a sibling class for which the OBAMS is being generated. This serves as a “background” control to remove genes whose expression is constant across the higher level branch that the class of interest resides in. For example, in our GC-B Cell analysis we wanted to find genes whose expression was unique when compared to other activated lymphocytes. As there were no activated B cell datasets available at time of analysis, we included comparisons to “marginal zone B cell” as this was a sibling class to germinal center B cell in the CL. If these comparisons were not included, B cell markers such as CD19 would have been included in the OBAMS. By taking this approach, we obtained biologically relevant results.

In summary, the IGP project has been an ongoing effort to characterize the transcriptomes of immune cells. While using an established technology, the breadth and scale of the consortium’s efforts presages the high-throughput “next-gen” and “next-next-gen” that are generating ever-increasing amounts of data. Structuring large volumes of data in an ontological context allows for integration of gene expression information with other disparate forms of data, the ability to do *in silico* experiments, and gain biological insights that might otherwise be missed.

## Conclusions

Characterization of molecular pathways can help elucidate the normal functioning of cells and the etiology of many diseases. We have developed a novel method for detecting pathways critical to cellular identity based on gene expression analysis using the Cell Ontology (CL). The CL is a manually constructed computer readable resource that links cell types by different relationships such as *develops_from* or *is_a* (subtype of). We have enhanced the CL to aid in analysis of complex biological data. We incorporated the CL into gene expression analysis of 88 immune cell types using publicly available data. We demonstrate we can find genes whose expression are associated with groupings of cell types and activated pathways that provides insights into cell type function. We extend our findings to study a cell type that selectively mutates its own genome and show that genes associated with this process are also implicated in cancer. These results establish that ontologies like the CL are valuable tools in analyzing complex biological data.

## Methods

### Pairwise analysis of gene expression data

The IGP has generated gene expression profiles for fluorescence-activated cell sorting (FACS) sorted mouse immune cell types by use of microarray gene chip analysis [15]. These data are available through the Gene Expression Omnibus (GSE15907) [49]. We downloaded and locally stored files that contain the original IGP gene expression data for 88 mature immune cell types (October 2010). We then developed a workflow to associate gene expression information with cell types (Figure 1). Standard Affymetrix gene chip analysis was done in R using the following Bioconductor packages: “affycoretools”, “affy”, “limma”, and “puma” [50-52]. Quality control of the original data was first determined using affystart. Gene expression across replicates was calculated using the Robust Multichip Average (RMA) normalization method. The “puma” package was used to generate a matrix of all possible pairwise comparisons between the 88 mature immune cell types. Differential expression of genes (as represented by hybridization to Affymetrix nucleotide probes) was calculated for each pairwise comparison by estimating the fold changes and standard errors through fitting a linear model for each gene followed by empirical Bayes smoothing of the standard errors. Two sets of genes were created for each pairwise comparison: one for genes whose expression was increased > 1.5-fold with an adjusted p-value < 0.05 using the Benjamini and Hochberg method to correct for multiple testing [53], and another set of genes whose expression decreased > 1.5-fold. The output was generated as a series of tab-delimited files with coordinates on the puma matrix and a “u” or “d” corresponding to up or down regulated genes.

### Mapping of pairwise comparisons to CL

Preliminary mappings between the mature immune cells sampled in the IGP and classes in the CL were established using text matching based on cell type names. This was followed by manual review of cell surface markers used. Immune cell representation in the CL was found to be robust, and the few omissions were added to CL by editing the ontology in OBO-Edit 2.1 [54]. CL classes that represented analyzed cell types in the IGP were referenced by inclusion of the IGP URL [1] in the dbxref of the cell type definition. The continuously updated source file for the CL ontology can be found at the Cell Ontology website [55]. Thus we maintain a robust mapping between CL and IGP cell types.

An analytical framework was created to leverage the logic of the CL in our pairwise analysis. First, we extracted a simple subset of the CL that excluded external ontologies (the complete CL includes portions of other ontologies such as the Protein Ontology as required to create logical definitions, a technique known as MIREOT [56]). We then extended this core ontology with a set of classes, each representing differentially expressed gene sets. We created a second root class (i.e. class with no parents) called “Gene-sets” with the child class “mature immune cell comparisons” (GS:0000001). We created a new ontology class s_i_,_j_ for every entry m_i_,_j_ in the differential expression matrix, where i ≠ j and i,j ≤ 88, yielding a total of 7,656 classes. We placed each class s_i,j_ as a subclass of GS:0000001, and added a *has_up_regulated_genes* relationship between s_i,j_ and c_i_ and a *has_down regulated_genes* relationship between s_i,j_ and c_j_. Note that the two halves of the matrix are not equivalent, since we are representing both up and down regulated genes. We then modified the resulting structure, removing redundant links. Also note that several c_i_ have more than one s_i,j_ they are associated with. These represent cases where the IGP consortium’s differentia of cell types was more refined than the CL. This includes the same cell type being isolated from different anatomical locations, and/or the use of an intermediate expression level of a cell marker to identify a cell type (e.g. peritoneal cavity macrophage expressing intermediate levels of *Emr1*).

The ontology, known as ‘CL-pairwise.obo’, is available online [57].

### Applying logic of CL to discover genes up regulated for B cell types

To determine the set of genes that are up regulated in a child class x compared to its parent y, the relevant pairwise comparisons were found using the CL-pairwise ontology. For each child–parent pair, we find the set of gene sets such that this gene set is inferred to be up regulated in any kind of x, down regulated in any kind of y, excluding gene sets down regulated in some kind of x.

For all x in C:

For all y in Parent( C ):

$$\;S*x,y=\left[\mathrm{Si},j\left|i\;\in \;\mathrm{anc}\left(x\right),j\;\notin \;\mathrm{anc}\left(y\right),j\;\notin \;\mathrm{anc}\left(x\right)\right)\right]$$

Here the anc function returns the reflexive closure of the ancestors of a class

In cases where there is more than one is_a parent to X, a search was done for each parent. The output of these searches was a list of pairwise comparisons that were fed into a custom R function. This function accessed the relevant pairwise comparison gene sets, found the genes common to all sets, calculated the mean fold expression change and standard deviation based on the fold-expression within each set, and added annotation information, including official gene symbols, from Mouse Genome Informatics (MGI) (Figure 2). The R function used can be found at the CL Immgen Data Archive [16].

### GO term enrichment

Genes found to be up regulated for a cell type were functionally evaluated using GO term enrichment statistical analysis tool, VLAD that is hosted at MGI [20]. This tool identifies GO classes that are significantly overrepresented in the annotations for a given set of genes. These computationally identified GO biological processes were compared to GO biological processes that were associated with CL terms by experiments reported in the literature. Also, a one-to-one correspondence was generated for species-specific mouse genes to corresponding protein terms in the Protein Ontology (PR) [8]. These PR terms were likewise compared to those PR terms manually associated with CL terms in logical definitions. In several cases, new associations were then made between particular CL classes and GO or PR classes.

## Competing interests

The authors declare that they have no competing interests.

## Authors’ contributions

TFM and NAV designed the study and performed the analyses. CJM, MAH, and JAB provided critical contributions to the study design, DSD contributed additional cell type definitions to the Cell Ontology to aid the study, ADD directed the study. TFM drafted the manuscripts and all authors contributed to its revision. All authors read and approved the final manuscript.

## Supplementary Material

Additional file 1 **OBAMS profiles for all mature B cells.** Additional file 1 contains a zip archive of OBAMS profiles for all mature B cells, including for each cell type individual spreadsheets showing up and down regulated genes for that cell type relative to parental cell types, and VLAD (GO term enrichment) results for all mature B cells. Click here for file

Additional file 2 **Genes Upregulated in GC-B cells vs MZ B cells.** This Excel spreadsheet presents the OBAMS profile for genes up-regulated in GC-B cells vs MZ B cells (to remove B cell marker type genes) and activated mature lymphocyte cell types. Click here for file

Additional file 3 **Genes upregulated in NK cells.** Side-by-side comparison of genes identified in OBAMS and ImmGen analyses with the genes ranked according to their fold-change (OBAMS) or delta score (ImmGen, data from supplementary file of Bezman et al. [32]) with the matches between the two lists indicated and potential reasons given to explain genes missing from either list. Click here for file
