# Supplementary material for: Ontology based molecular signatures for immune cell types via gene expression analysis
Source: BMC Bioinformatics. 2013 Aug 30;14:263. doi: 10.1186/1471-2105-14-263 (PMC3844401; doi:10.1186/1471-2105-14-263)
Supplement: Additional file 1 — OBAMS profiles for all mature B cells. Additional file 1 contains a zip archive of OBAMS profiles for all mature B cells, including for each cell type individual spreadsheets showing up and down regulated genes for that cell type relative to parental cell types, and VLAD (GO term enrichment) results for all mature B cells. [file 1471-2105-14-263-S1.zip › Additional File 1/B-1 B cell/VLAD.CL_0000819_up/results.html]

# CL\_0000819\_up

|  |  |
| --- | --- |
| Vlad version: | v1.5 |
| Date: | Mon Aug 29 17:20:30 2011 |
| Run time: | 31.36 sec |
| Ontology file: | gene\_ontology.obo |
| Ontology date: | Fri Aug 26 19:30:00 2011 |
| Annotation file: | gene\_association.mgi |
| Annotation date: | ??? |
| Analysis type: | enrichment |
| Excluded evidence codes: | ND |
| Number of query sets: | 1 |
| Query set 1: | CL\_0000819-up.xls (n=59; 254 not found) |
| Universe set: | default (everything) |
| Graph display: | Top 25 scoring terms and their ancestors. Interior nodes have been culled. |

**Jump to:** biological\_process | cellular\_component | molecular\_function | Unannotated id/symbols

### biological\_process (top)

  
  


### cellular\_component (top)

  
  


### molecular\_function (top)

  
  


### Unannotated IDs

|  |
| --- |
| **CL\_0000819-up.xls**  0.189780476903999 0.204383315146949 0.343153900027546 0.423692933945528 0.437659211795718 0.466072995712785 0.537650939426117 0.591158091889036 0.61260460925464 0.696005081110295 0.706776858412852 0.731075935026781 0.766500691249157 0.792667501682443 0.889472095138668 0.937232234313923 1.0263058399969 1.02804134996661 1.04864193379166 1.17822990676881 1.19009891161403 1.19753306520634 1.2208986390625 1.30409186232368 1.44239682128012 1.46946784075766 1.48934612263328 1.54121198087376 1.74810684407841 1.77442389773384 1.83811806074023 1.97550844596767 1.98199204093935 1.98225299533317 1.98848226243954 10.1305123036062 10.4596410093756 10.4807305801178 100038391 100041194 101202 10346790 10356082 10356299 10358928 10363541 10364030 10369276 10373680 10389581 10399908 10401937 10402783 10403584 10407327 10409265 10410995 10419744 10421774 10425066 10435704 10435712 10441864 10446282 10449741 10456904 10457733 10462398 10467139 10470614 10470959 10471154 10476021 10482448 10494978 10496771 10498302 10502780 10502785 10504891 10510150 10523281 10530269 10538892 10542740 10542917 10546762 10547177 10548105 10549276 10552125 10553598 10562044 10563077 10565924 10566877 10571958 10572949 10576661 10578771 10587792 10588577 10597000 10603346 108150 11.2209449629127 11.673769528451 11.6956307518169 110784 11540 11898 11923 11992 12290 12444 12477 12519 12700 12983 13.7289487991632 13.7652637122593 13723 13733 14.6067072441136 15.7809831222414 16192 16412 16651 16889 17.8875815474895 17356 17691 18.8428734780926 18073 18624 18824 19088 19201 19260 19261 19418 2.02548906663989 2.02758383123512 2.03182125582799 2.05132408271688 2.09670908564387 2.16500099276464 2.17440684612435 2.18360446322415 2.22181080927888 2.25835878325551 2.3308748845035 2.39887922244825 2.48977813358946 2.5545494558269 2.62531358326863 2.72924120503483 2.73159953681926 2.74164679822496 2.90096584803475 2.92259758282899 20430 20540 211305 213391 215999 22038 227326 227696 230157 24136 242037 245945 27.7716210661302 3.07461564729398 3.11540799166496 3.12059311604138 3.27563391260805 3.30638905015309 3.31466699678761 3.36226303340566 3.39278416896066 3.44313711487739 3.46762796143583 3.50701867687047 3.52133122040973 3.52212753984431 3.53118620020948 3.6353655102971 3.82965797251136 3.93154215047431 3.94265133850502 319934 320100 352945 380921 4.00367500638054 4.24268259805305 4.27660524700809 4.38937884172914 4.42849638027614 4.42930708390442 4.43991160126692 4.50653579097158 4.51896963406065 4.65908760260561 4.69327491868346 4.78356000303637 4.79659463968531 4.86418987627795 4.87692426962011 433804 5.10594911470604 5.33997568535661 5.43809661751172 5.47290318723646 5.8870688657884 52377 52398 56386 58205 58206 59009 6.2666184521181 6.29999216144792 6.60888389251752 6.65365717552009 6.69224255173483 6.75366660805475 6.95673676564374 6.99025982672274 68279 68870 7.13737176933074 7.5293898922453 7.55552035621063 7.61454888592618 7.96335765513284 77864 79362 8.57317063711052 8.58688933877526 8.81399471287975 9.13014488104933 9.52232157699763 9.59820602819205 9.7687111073148 99633 FoldChange NA Stdv entrezIDs mgiID symbol |

|  |  |  |
| --- | --- | --- |
| [close] | **Legend: Edge Types** | (details) |
|  | | |
