# Supplementary material for: Ontology based molecular signatures for immune cell types via gene expression analysis
Source: BMC Bioinformatics. 2013 Aug 30;14:263. doi: 10.1186/1471-2105-14-263 (PMC3844401; doi:10.1186/1471-2105-14-263)
Supplement: Additional file 1 — OBAMS profiles for all mature B cells. Additional file 1 contains a zip archive of OBAMS profiles for all mature B cells, including for each cell type individual spreadsheets showing up and down regulated genes for that cell type relative to parental cell types, and VLAD (GO term enrichment) results for all mature B cells. [file 1471-2105-14-263-S1.zip › Additional File 1/B-1b B cell/VLAD.CL_0000821_down/results.html]

# CL\_0000821\_down

|  |  |
| --- | --- |
| Vlad version: | v1.5 |
| Date: | Mon Aug 29 17:24:22 2011 |
| Run time: | 34.25 sec |
| Ontology file: | gene\_ontology.obo |
| Ontology date: | Fri Aug 26 19:30:00 2011 |
| Annotation file: | gene\_association.mgi |
| Annotation date: | ??? |
| Analysis type: | enrichment |
| Excluded evidence codes: | ND |
| Number of query sets: | 1 |
| Query set 1: | CL\_0000821-down.xls (n=91; 412 not found) |
| Universe set: | default (everything) |
| Graph display: | Top 25 scoring terms and their ancestors. Interior nodes have been culled. |

**Jump to:** biological\_process | cellular\_component | molecular\_function | Unannotated id/symbols

### biological\_process (top)

  
  


### cellular\_component (top)

  
  


### molecular\_function (top)

  
  


### Unannotated IDs

|  |
| --- |
| **CL\_0000821-down.xls**  0.00252254144875113 0.00489105170051326 0.00763808485520858 0.0173850392984892 0.0182571314125075 0.0246910266447011 0.0399108349186516 0.0428522921679794 0.0465129464682093 0.0534176091625998 0.0584687123180593 0.071477442959313 0.0717157860054372 0.0722250570623187 0.0854668062545594 0.0938356892332196 0.103332803738255 0.106471025386661 0.109840286865361 0.125023071164514 0.125442186621684 0.125908673013525 0.129683271435181 0.13374852782996 0.14896187691878 0.166588839866548 0.180934283901554 0.181849111906592 0.184328954447376 0.190067940124856 0.193138659862312 0.20926290146836 0.209444251977864 0.220245943212606 0.22229432083952 0.223734718193912 0.224493155083948 0.236065458297718 0.253540304952098 0.27405464990717 0.280697562831976 0.284745266491436 0.28877996005969 0.290413874565926 0.304558117782358 0.311816001213509 0.322518201980296 0.334359541159067 0.340595930436266 0.346467098857489 0.349981840580452 0.350348767259866 0.372676728105999 0.379465121701398 0.388366590142404 0.399594094963408 0.406109212318368 0.407418288945939 0.468650633745627 0.514407474820095 0.561972506538533 0.578581881162426 0.6074297003881 0.611598944182531 0.631865608028671 0.676362224010198 0.677256171511009 0.684145959136538 0.687591231662045 0.717999612777517 0.729519941923317 0.750546751430472 0.753740726761813 0.753942749364763 0.756333734715846 0.81236442236423 0.817848151301946 0.846198471276601 0.857377644480179 0.89534145454223 0.90759480110027 1.00886239854695 1.07355343149911 1.18484362135342 1.22145468566422 1.25705808811861 1.40044644171335 1.44573567091785 1.46242770263019 1.56218559185273 1.56236938772511 1.58132808123124 1.64744656420669 1.66454725628918 1.69167409001365 1.69497963862877 1.72083468880574 1.73456916360043 1.79332792293347 1.80250034511527 1.80352669445178 1.80424709939137 1.80657576687965 1.81379793751319 1.81417411491323 1.81518631011114 1.82856172402252 1.83424211581465 1.86322916871585 1.88004713024138 1.89210581014088 1.90009264379213 1.90523609461765 1.910143824211 1.91774868492337 1.92220507167505 1.9453224352317 1.95046443668091 1.95767719205093 1.97626503037971 1.98464013217573 1.98566880724452 100434 100504518 10346321 10349580 10349593 10351015 10353192 10353307 10353438 10354979 10355984 10359190 10359689 10359713 10368199 10374767 10379321 10385776 10386909 10389022 10389025 10392845 10393559 10399148 10400006 10401244 10402268 10411680 10415857 10417526 10418842 10419744 10422227 10423570 10423825 10430006 10435769 10435789 10435791 10437687 10439845 10439895 10440099 10445977 10447383 10456904 10459854 10459944 10461553 10461558 10466040 10469581 10472820 10473384 10479938 10480238 10481857 10497149 10499095 10499108 10500610 10504375 10505008 10508465 10510129 10515220 10516620 10516658 10523376 10523579 10525134 10530269 10531261 10533751 10542993 10544002 10548030 10553354 10554094 10554667 10555059 10556266 10556297 10560624 10565315 10566877 10568174 10573008 10576090 10587226 10587639 10590620 10592515 10593015 10593473 10593492 10593668 10593671 10595059 10598004 10598207 10603247 10605181 10608535 10608666 10608670 10608672 108735 109711 11535 11622 11658 11816 11905 11931 12502 12507 12527 12768 12769 13421 14048 14073 14085 14368 16001 16403 16818 17075 170829 17357 17863 18018 18516 18703 19141 19201 19415 19679 19703 2.00858864483571 2.0175212854464 2.02746058684874 2.04767079312212 2.07458881769686 2.07657151609188 2.10427234896953 2.11844621487014 2.11931237011732 2.12286710234784 2.17885302385824 2.18732250361024 2.19159256940033 2.20828551232298 2.21573234350239 2.22049215402288 2.22269702587687 2.22310694176053 2.2259297219569 2.24675954161176 2.24710469564107 2.24771958491131 2.26061084216326 2.27186889227163 2.28985196657281 2.48734090409493 2.5248984667344 2.53993776183103 2.56060235329905 2.59384281583469 2.60943888666694 2.63120560891838 2.63284479461148 2.64562596134238 2.65162093523351 2.65634083813495 2.69053233335917 2.71459325915254 2.72885320912059 2.74078414940877 2.754643211702 2.76639969578723 2.7703509679424 2.80712878759702 2.85732946210413 2.85888659825018 2.98846079204819 20148 20540 20720 20737 208618 208647 21414 215015 217944 218518 21858 219148 22390 22761 229488 230103 231470 231532 233424 233529 235380 23959 24064 241230 244871 245945 246746 268451 269823 3.02128992099559 3.03051139466273 3.08288612636138 3.13052443905106 3.13498205386517 3.17033262401188 3.23688494990521 3.34070814594344 3.35443421885053 3.41763745352632 3.46423283081761 3.61745650687542 3.68786982036174 3.96557408425666 3.97088716427443 319934 320302 338367 387285 4.23466972941532 4.27450375278418 4.27989931108787 4.42834549531153 4.47786318303065 4.64067586381955 4.76516456148877 4.99067104403676 403202 433024 5.02501599649606 5.10552810981405 5.24253166115128 5.25153819404723 5.31427815573124 5.38178465017408 5.49671258608353 5.71968261771182 5.75813534544773 5.95678204420875 50498 54613 56490 56722 58207 6.04179729455216 6.14098277234131 6.14619349535747 6.24101176860295 6.40757656288634 6.98200914240137 622675 628004 65221 66264 67856 68151 69169 69809 7.2364384527184 7.7160054455882 70082 72027 72828 73139 73246 74018 74645 78286 FoldChange NA Stdv entrezIDs mgiID symbol |

|  |  |  |
| --- | --- | --- |
| [close] | **Legend: Edge Types** | (details) |
|  | | |
