# Supplementary material for: Ontology based molecular signatures for immune cell types via gene expression analysis
Source: BMC Bioinformatics. 2013 Aug 30;14:263. doi: 10.1186/1471-2105-14-263 (PMC3844401; doi:10.1186/1471-2105-14-263)
Supplement: Additional file 1 — OBAMS profiles for all mature B cells. Additional file 1 contains a zip archive of OBAMS profiles for all mature B cells, including for each cell type individual spreadsheets showing up and down regulated genes for that cell type relative to parental cell types, and VLAD (GO term enrichment) results for all mature B cells. [file 1471-2105-14-263-S1.zip › Additional File 1/B-1b B cell/VLAD.CL_0000821_up/results.html]

# CL\_0000821\_up

|  |  |
| --- | --- |
| Vlad version: | v1.5 |
| Date: | Mon Aug 29 17:24:36 2011 |
| Run time: | 32.31 sec |
| Ontology file: | gene\_ontology.obo |
| Ontology date: | Fri Aug 26 19:30:00 2011 |
| Annotation file: | gene\_association.mgi |
| Annotation date: | ??? |
| Analysis type: | enrichment |
| Excluded evidence codes: | ND |
| Number of query sets: | 1 |
| Query set 1: | CL\_0000821-up.xls (n=66; 287 not found) |
| Universe set: | default (everything) |
| Graph display: | Top 25 scoring terms and their ancestors. Interior nodes have been culled. |

**Jump to:** biological\_process | cellular\_component | molecular\_function | Unannotated id/symbols

### biological\_process (top)

  
  


### cellular\_component (top)

  
  


### molecular\_function (top)

  
  


### Unannotated IDs

|  |
| --- |
| **CL\_0000821-up.xls**  0.0128870527857004 0.026363185148509 0.038592820355335 0.0457308330895416 0.0524135672321641 0.0561402248950635 0.0800014964837322 0.0915215692174068 0.122635659782747 0.122854446510172 0.165290805064016 0.188296574081404 0.201418061311243 0.211652141533818 0.213469935759167 0.218604993687062 0.226485045193119 0.267680135698595 0.293387409180316 0.294561319695724 0.323013965001069 0.333405207340858 0.358457238497142 0.367482513515057 0.386556776062857 0.390709511192344 0.423613255363257 0.42845971293084 0.43888942892634 0.453819843888247 0.463701130662252 0.491002235012466 0.546799744070746 0.591705853188112 0.607054297668369 0.607777217113763 0.640191680434896 0.645420194317165 0.650089345220377 0.653922591751311 0.654669766095442 0.695433820635524 0.700211022059032 0.716737947374597 0.722499075800658 0.789481931864266 0.806729700704678 0.813049637431531 0.832883760597028 0.878190049202736 0.903225299118691 0.929014040279274 0.94827494504178 0.982396809800623 0.991680523519602 1.00159928212368 1.0248165484278 1.04515434120449 1.10779487100559 1.18519431135433 1.19519839231122 1.31264371456792 1.53461756041792 1.55124069312471 1.57266154514075 1.58394077984895 1.64870485912213 1.66300700342264 1.68374708261491 1.68516684524059 1.68791236293686 1.74133736475607 1.75051690655714 1.82585460448881 1.82967309583245 1.83418984849869 1.83635859368871 1.84196168691722 1.86629753422536 1.87939458224394 1.88432490632924 1.89992432662307 1.90099708177869 1.92540649682159 1.98697813405483 10.1643395506648 10.4892502587172 100034251 100041581 10347481 10350840 10357590 10360028 10365658 10366546 10368647 10371321 10372781 10375402 10379727 10383010 10384974 10390032 10390542 10390560 10393341 10393449 10399299 10399419 10400762 10402708 104086 10408610 10408613 10408975 10409162 10421697 10422822 10425370 10426315 10426368 10428534 10428536 10429341 10432661 10434758 10437516 10441456 10441633 10442115 10442211 10458767 10464251 10466606 10469278 10479463 10479852 10481574 10482448 10484402 10487447 10489569 10491952 10499766 10505163 10523547 10524284 10527012 10532085 10538887 10541507 10545014 10545425 10546855 10557862 10577517 10581737 10584870 10593050 10597000 10600852 112 11492 11628 12295 12388 12458 12702 12709 14083 14130 16.5804406257009 16154 16184 16199 16409 16553 16848 16880 16952 18.7884262781878 18408 18830 19041 2.0069528424782 2.02154314429918 2.02463111578349 2.02489472491906 2.02791354018153 2.05567541275597 2.06640851427838 2.07456950342904 2.11969314151267 2.13436985070094 2.14742087936368 2.18486689133266 2.24721541660267 2.24859774187542 2.25361470245405 2.27212664829774 2.28001465835009 2.32707480128532 2.348990837829 2.40809241656967 2.42252394926761 2.4486409883456 2.48523464253072 2.5139949287551 2.60817611491995 2.62449629912928 2.70881939971278 2.7390060055732 2.75614916675727 2.79428277180771 2.85871316003056 2.95000365692315 2.97138177737238 20440 207839 207921 209683 20975 210808 211305 211666 212898 214531 217154 217344 21814 22151 226255 226419 227541 228576 228993 231510 232023 234733 239556 241303 24132 24136 259302 264895 26568 28105 3.00905925345392 3.05446456514616 3.12941230873431 3.1699960518423 3.19763642862627 3.22953046372707 3.4109167479816 3.50900464358909 3.91311451698254 338365 381776 399510 4.05025322479715 4.07225187860924 4.1397650788838 4.5821716730822 4.87395891195873 4.88578086159633 4.93515608716902 433070 5.22955465669667 5.62532354111651 5.7136190015138 5.76236653571375 66329 66725 70574 71130 72713 73710 73914 75089 83925 FoldChange Igkv2 NA Stdv entrezIDs mgiID symbol |

|  |  |  |
| --- | --- | --- |
| [close] | **Legend: Edge Types** | (details) |
|  | | |
