# Supplementary material for: Ontology based molecular signatures for immune cell types via gene expression analysis
Source: BMC Bioinformatics. 2013 Aug 30;14:263. doi: 10.1186/1471-2105-14-263 (PMC3844401; doi:10.1186/1471-2105-14-263)
Supplement: Additional file 1 — OBAMS profiles for all mature B cells. Additional file 1 contains a zip archive of OBAMS profiles for all mature B cells, including for each cell type individual spreadsheets showing up and down regulated genes for that cell type relative to parental cell types, and VLAD (GO term enrichment) results for all mature B cells. [file 1471-2105-14-263-S1.zip › Additional File 1/B-2 B cell/VLAD.CL_0000822-up/results.html]

# CL\_0000822-up

|  |  |
| --- | --- |
| Vlad version: | v1.5 |
| Date: | Tue Oct 25 10:09:43 2011 |
| Run time: | 53.57 sec |
| Ontology file: | gene\_ontology.obo |
| Ontology date: | Mon Oct 24 19:30:00 2011 |
| Annotation file: | gene\_association.mgi |
| Annotation date: | Wed Oct 19 00:00:00 2011 |
| Analysis type: | enrichment |
| Excluded evidence codes: | ND |
| Number of query sets: | 1 |
| Query set 1: | Mature B cell (n=20; 1 not found) |
| Universe set: | default (everything) |
| Graph display: | Top 25 scoring terms and their ancestors. Interior nodes have been culled. |

**Jump to:** biological\_process | cellular\_component | molecular\_function | Unannotated id/symbols

### biological\_process (top)

  
  


### cellular\_component (top)

  
  


### molecular\_function (top)

  
  


### Unannotated IDs

|  |
| --- |
| **Mature B cell**  NA |

|  |  |  |
| --- | --- | --- |
| [close] | **Legend: Edge Types** | (details) |
|  | | |
