# Supplementary material for: Ontology based molecular signatures for immune cell types via gene expression analysis
Source: BMC Bioinformatics. 2013 Aug 30;14:263. doi: 10.1186/1471-2105-14-263 (PMC3844401; doi:10.1186/1471-2105-14-263)
Supplement: Additional file 1 — OBAMS profiles for all mature B cells. Additional file 1 contains a zip archive of OBAMS profiles for all mature B cells, including for each cell type individual spreadsheets showing up and down regulated genes for that cell type relative to parental cell types, and VLAD (GO term enrichment) results for all mature B cells. [file 1471-2105-14-263-S1.zip › Additional File 1/B-2 B cell/VLAD.CL_0000822_down/results.html]

# CL\_0000822\_down

|  |  |
| --- | --- |
| Vlad version: | v1.5.1 |
| Date: | Wed Jun 20 07:20:35 2012 |
| Run time: | 61.82 sec |
| Ontology file: | gene\_ontology.obo |
| Ontology date: | Tue Jun 19 19:30:00 2012 |
| Annotation file: | gene\_association.mgi |
| Annotation date: | Wed Jun 6 00:00:00 2012 |
| Analysis type: | enrichment |
| Excluded evidence codes: | ND |
| Number of query sets: | 1 |
| Query set 1: | CL\_0000822\_down (n=26; 2 not found) |
| Universe set: | default (everything) |
| Graph display: | Top 25 scoring terms and their ancestors. Interior nodes have been culled. |

**Jump to:** biological\_process | cellular\_component | molecular\_function | Unannotated id/symbols

### biological\_process (top)

  
  


| TermID | Term | P | Q | k | n | K | N | k/n | K/N | k/K | n/N | Qset | Symbols |
| --- | --- | --- | --- | --- | --- | --- | --- | --- | --- | --- | --- | --- | --- |
| GO:0015801 | aromatic amino acid transport | 1.45e-03 | 1.72e-01 | 1 | 22 | 1 | 15137 | 4.55% | 0.01% | 100.00% | 0.15% | CL\_0000822\_down | Slc36a4 |
| GO:0002642 | positive regulation of immunoglobulin biosynthetic process | 1.45e-03 | 1.72e-01 | 1 | 22 | 1 | 15137 | 4.55% | 0.01% | 100.00% | 0.15% | CL\_0000822\_down | 2010001M09Rik |
| GO:0002640 | regulation of immunoglobulin biosynthetic process | 1.45e-03 | 1.72e-01 | 1 | 22 | 1 | 15137 | 4.55% | 0.01% | 100.00% | 0.15% | CL\_0000822\_down | 2010001M09Rik |
| GO:0015827 | tryptophan transport | 1.45e-03 | 1.72e-01 | 1 | 22 | 1 | 15137 | 4.55% | 0.01% | 100.00% | 0.15% | CL\_0000822\_down | Slc36a4 |
| GO:2000491 | positive regulation of hepatic stellate cell activation | 2.90e-03 | 1.79e-01 | 1 | 22 | 2 | 15137 | 4.55% | 0.01% | 50.00% | 0.15% | CL\_0000822\_down | Rps6ka1 |
| GO:0032594 | protein transport within lipid bilayer | 2.90e-03 | 1.79e-01 | 1 | 22 | 2 | 15137 | 4.55% | 0.01% | 50.00% | 0.15% | CL\_0000822\_down | Itgb1 |
| GO:2000489 | regulation of hepatic stellate cell activation | 2.90e-03 | 1.79e-01 | 1 | 22 | 2 | 15137 | 4.55% | 0.01% | 50.00% | 0.15% | CL\_0000822\_down | Rps6ka1 |
| GO:0002700 | regulation of production of molecular mediator of immune response | 3.73e-03 | 1.79e-01 | 2 | 22 | 63 | 15137 | 9.09% | 0.42% | 3.17% | 0.15% | CL\_0000822\_down | 2010001M09Rik, Sema7a |
| GO:0015866 | ADP transport | 4.35e-03 | 1.79e-01 | 1 | 22 | 3 | 15137 | 4.55% | 0.02% | 33.33% | 0.15% | CL\_0000822\_down | Slc25a4 |
| GO:0007161 | calcium-independent cell-matrix adhesion | 4.35e-03 | 1.79e-01 | 1 | 22 | 3 | 15137 | 4.55% | 0.02% | 33.33% | 0.15% | CL\_0000822\_down | Itgb1 |
| GO:0032497 | detection of lipopolysaccharide | 4.35e-03 | 1.79e-01 | 1 | 22 | 3 | 15137 | 4.55% | 0.02% | 33.33% | 0.15% | CL\_0000822\_down | Ly96 |
| GO:0007229 | integrin-mediated signaling pathway | 5.11e-03 | 1.79e-01 | 2 | 22 | 74 | 15137 | 9.09% | 0.49% | 2.70% | 0.15% | CL\_0000822\_down | Itgb1, Sema7a |
| GO:0015867 | ATP transport | 5.80e-03 | 1.79e-01 | 1 | 22 | 4 | 15137 | 4.55% | 0.03% | 25.00% | 0.15% | CL\_0000822\_down | Slc25a4 |
| GO:0051503 | adenine nucleotide transport | 5.80e-03 | 1.79e-01 | 1 | 22 | 4 | 15137 | 4.55% | 0.03% | 25.00% | 0.15% | CL\_0000822\_down | Slc25a4 |
| GO:0008295 | spermidine biosynthetic process | 5.80e-03 | 1.79e-01 | 1 | 22 | 4 | 15137 | 4.55% | 0.03% | 25.00% | 0.15% | CL\_0000822\_down | Srm |
| GO:0015808 | L-alanine transport | 7.25e-03 | 1.79e-01 | 1 | 22 | 5 | 15137 | 4.55% | 0.03% | 20.00% | 0.15% | CL\_0000822\_down | Slc36a4 |
| GO:0006862 | nucleotide transport | 7.25e-03 | 1.79e-01 | 1 | 22 | 5 | 15137 | 4.55% | 0.03% | 20.00% | 0.15% | CL\_0000822\_down | Slc25a4 |
| GO:0015865 | purine nucleotide transport | 7.25e-03 | 1.79e-01 | 1 | 22 | 5 | 15137 | 4.55% | 0.03% | 20.00% | 0.15% | CL\_0000822\_down | Slc25a4 |
| GO:0015868 | purine ribonucleotide transport | 7.25e-03 | 1.79e-01 | 1 | 22 | 5 | 15137 | 4.55% | 0.03% | 20.00% | 0.15% | CL\_0000822\_down | Slc25a4 |
| GO:0008277 | regulation of G-protein coupled receptor protein signaling pathway | 8.30e-03 | 1.79e-01 | 2 | 22 | 95 | 15137 | 9.09% | 0.63% | 2.11% | 0.15% | CL\_0000822\_down | Arhgef12, Itgb1 |
| GO:0032328 | alanine transport | 8.69e-03 | 1.79e-01 | 1 | 22 | 6 | 15137 | 4.55% | 0.04% | 16.67% | 0.15% | CL\_0000822\_down | Slc36a4 |
| GO:0060907 | positive regulation of macrophage cytokine production | 8.69e-03 | 1.79e-01 | 1 | 22 | 6 | 15137 | 4.55% | 0.04% | 16.67% | 0.15% | CL\_0000822\_down | Sema7a |
| GO:0008216 | spermidine metabolic process | 8.69e-03 | 1.79e-01 | 1 | 22 | 6 | 15137 | 4.55% | 0.04% | 16.67% | 0.15% | CL\_0000822\_down | Srm |
| GO:0060547 | negative regulation of necrotic cell death | 1.16e-02 | 2.03e-01 | 1 | 22 | 8 | 15137 | 4.55% | 0.05% | 12.50% | 0.15% | CL\_0000822\_down | Slc25a4 |
| GO:0006596 | polyamine biosynthetic process | 1.16e-02 | 2.03e-01 | 1 | 22 | 8 | 15137 | 4.55% | 0.05% | 12.50% | 0.15% | CL\_0000822\_down | Srm |
| GO:0061081 | positive regulation of myeloid leukocyte cytokine production involved in immune response | 1.16e-02 | 2.03e-01 | 1 | 22 | 8 | 15137 | 4.55% | 0.05% | 12.50% | 0.15% | CL\_0000822\_down | Sema7a |
| GO:0015824 | proline transport | 1.16e-02 | 2.03e-01 | 1 | 22 | 8 | 15137 | 4.55% | 0.05% | 12.50% | 0.15% | CL\_0000822\_down | Slc36a4 |
| GO:0032490 | detection of molecule of bacterial origin | 1.30e-02 | 2.07e-01 | 1 | 22 | 9 | 15137 | 4.55% | 0.06% | 11.11% | 0.15% | CL\_0000822\_down | Ly96 |
| GO:0051329 | interphase of mitotic cell cycle | 1.36e-02 | 2.07e-01 | 2 | 22 | 123 | 15137 | 9.09% | 0.81% | 1.63% | 0.15% | CL\_0000822\_down | Itgb1, Tfdp1 |
| GO:0006817 | phosphate ion transport | 1.44e-02 | 2.07e-01 | 1 | 22 | 10 | 15137 | 4.55% | 0.07% | 10.00% | 0.15% | CL\_0000822\_down | Slc20a2 |
| GO:0010935 | regulation of macrophage cytokine production | 1.44e-02 | 2.07e-01 | 1 | 22 | 10 | 15137 | 4.55% | 0.07% | 10.00% | 0.15% | CL\_0000822\_down | Sema7a |
| GO:0070830 | tight junction assembly | 1.44e-02 | 2.07e-01 | 1 | 22 | 10 | 15137 | 4.55% | 0.07% | 10.00% | 0.15% | CL\_0000822\_down | Itgb1 |
| GO:0051325 | interphase | 1.45e-02 | 2.07e-01 | 2 | 22 | 127 | 15137 | 9.09% | 0.84% | 1.57% | 0.15% | CL\_0000822\_down | Itgb1, Tfdp1 |
| GO:0000084 | S phase of mitotic cell cycle | 1.73e-02 | 2.33e-01 | 1 | 22 | 12 | 15137 | 4.55% | 0.08% | 8.33% | 0.15% | CL\_0000822\_down | Tfdp1 |
| GO:0008354 | germ cell migration | 1.73e-02 | 2.33e-01 | 1 | 22 | 12 | 15137 | 4.55% | 0.08% | 8.33% | 0.15% | CL\_0000822\_down | Itgb1 |
| GO:0002042 | cell migration involved in sprouting angiogenesis | 1.87e-02 | 2.33e-01 | 1 | 22 | 13 | 15137 | 4.55% | 0.09% | 7.69% | 0.15% | CL\_0000822\_down | Itgb1 |
| GO:0072530 | purine-containing compound transmembrane transport | 1.87e-02 | 2.33e-01 | 1 | 22 | 13 | 15137 | 4.55% | 0.09% | 7.69% | 0.15% | CL\_0000822\_down | Slc25a4 |
| GO:0010939 | regulation of necrotic cell death | 1.87e-02 | 2.33e-01 | 1 | 22 | 13 | 15137 | 4.55% | 0.09% | 7.69% | 0.15% | CL\_0000822\_down | Slc25a4 |
| GO:0031346 | positive regulation of cell projection organization | 1.98e-02 | 2.40e-01 | 2 | 22 | 150 | 15137 | 9.09% | 0.99% | 1.33% | 0.15% | CL\_0000822\_down | Itgb1, Sema7a |
| GO:0051320 | S phase | 2.16e-02 | 2.55e-01 | 1 | 22 | 15 | 15137 | 4.55% | 0.10% | 6.67% | 0.15% | CL\_0000822\_down | Tfdp1 |
| GO:0071705 | nitrogen compound transport | 2.36e-02 | 2.73e-01 | 2 | 22 | 165 | 15137 | 9.09% | 1.09% | 1.21% | 0.15% | CL\_0000822\_down | Slc25a4, Slc36a4 |
| GO:0006595 | polyamine metabolic process | 2.44e-02 | 2.75e-01 | 1 | 22 | 17 | 15137 | 4.55% | 0.11% | 5.88% | 0.15% | CL\_0000822\_down | Srm |
| GO:0043297 | apical junction assembly | 2.59e-02 | 2.84e-01 | 1 | 22 | 18 | 15137 | 4.55% | 0.12% | 5.56% | 0.15% | CL\_0000822\_down | Itgb1 |
| GO:0002720 | positive regulation of cytokine production involved in immune response | 2.73e-02 | 2.93e-01 | 1 | 22 | 19 | 15137 | 4.55% | 0.13% | 5.26% | 0.15% | CL\_0000822\_down | Sema7a |
| GO:0043410 | positive regulation of MAPK cascade | 2.87e-02 | 2.99e-01 | 2 | 22 | 183 | 15137 | 9.09% | 1.21% | 1.09% | 0.15% | CL\_0000822\_down | Itgb1, Sema7a |
| GO:0045214 | sarcomere organization | 3.01e-02 | 2.99e-01 | 1 | 22 | 21 | 15137 | 4.55% | 0.14% | 4.76% | 0.15% | CL\_0000822\_down | Itgb1 |
| GO:0043534 | blood vessel endothelial cell migration | 3.15e-02 | 2.99e-01 | 1 | 22 | 22 | 15137 | 4.55% | 0.15% | 4.55% | 0.15% | CL\_0000822\_down | Itgb1 |
| GO:0015804 | neutral amino acid transport | 3.15e-02 | 2.99e-01 | 1 | 22 | 22 | 15137 | 4.55% | 0.15% | 4.55% | 0.15% | CL\_0000822\_down | Slc36a4 |
| GO:0045773 | positive regulation of axon extension | 3.15e-02 | 2.99e-01 | 1 | 22 | 22 | 15137 | 4.55% | 0.15% | 4.55% | 0.15% | CL\_0000822\_down | Sema7a |
| GO:0002697 | regulation of immune effector process | 3.22e-02 | 2.99e-01 | 2 | 22 | 195 | 15137 | 9.09% | 1.29% | 1.03% | 0.15% | CL\_0000822\_down | 2010001M09Rik, Sema7a |
| GO:0009595 | detection of biotic stimulus | 3.29e-02 | 2.99e-01 | 1 | 22 | 23 | 15137 | 4.55% | 0.15% | 4.35% | 0.15% | CL\_0000822\_down | Ly96 |
| GO:0006826 | iron ion transport | 3.29e-02 | 2.99e-01 | 1 | 22 | 23 | 15137 | 4.55% | 0.15% | 4.35% | 0.15% | CL\_0000822\_down | Sfxn1 |
| GO:0002702 | positive regulation of production of molecular mediator of immune response | 3.43e-02 | 3.01e-01 | 1 | 22 | 24 | 15137 | 4.55% | 0.16% | 4.17% | 0.15% | CL\_0000822\_down | Sema7a |
| GO:0061178 | regulation of insulin secretion involved in cellular response to glucose stimulus | 3.43e-02 | 3.01e-01 | 1 | 22 | 24 | 15137 | 4.55% | 0.16% | 4.17% | 0.15% | CL\_0000822\_down | Hmgn3 |
| GO:0070098 | chemokine-mediated signaling pathway | 3.85e-02 | 3.26e-01 | 1 | 22 | 27 | 15137 | 4.55% | 0.18% | 3.70% | 0.15% | CL\_0000822\_down | Ccbp2 |
| GO:0031663 | lipopolysaccharide-mediated signaling pathway | 3.85e-02 | 3.26e-01 | 1 | 22 | 27 | 15137 | 4.55% | 0.18% | 3.70% | 0.15% | CL\_0000822\_down | Ly96 |
| GO:0007043 | cell-cell junction assembly | 3.99e-02 | 3.31e-01 | 1 | 22 | 28 | 15137 | 4.55% | 0.18% | 3.57% | 0.15% | CL\_0000822\_down | Itgb1 |
| GO:0010975 | regulation of neuron projection development | 4.25e-02 | 3.42e-01 | 2 | 22 | 227 | 15137 | 9.09% | 1.50% | 0.88% | 0.15% | CL\_0000822\_down | Itgb1, Sema7a |
| GO:0038032 | termination of G-protein coupled receptor signaling pathway | 4.41e-02 | 3.42e-01 | 1 | 22 | 31 | 15137 | 4.55% | 0.20% | 3.23% | 0.15% | CL\_0000822\_down | Arhgef12 |
| GO:0015807 | L-amino acid transport | 4.55e-02 | 3.42e-01 | 1 | 22 | 32 | 15137 | 4.55% | 0.21% | 3.12% | 0.15% | CL\_0000822\_down | Slc36a4 |
| GO:0007159 | leukocyte cell-cell adhesion | 4.55e-02 | 3.42e-01 | 1 | 22 | 32 | 15137 | 4.55% | 0.21% | 3.12% | 0.15% | CL\_0000822\_down | Itgb1 |
| GO:0030239 | myofibril assembly | 4.55e-02 | 3.42e-01 | 1 | 22 | 32 | 15137 | 4.55% | 0.21% | 3.12% | 0.15% | CL\_0000822\_down | Itgb1 |
| GO:0002040 | sprouting angiogenesis | 4.55e-02 | 3.42e-01 | 1 | 22 | 32 | 15137 | 4.55% | 0.21% | 3.12% | 0.15% | CL\_0000822\_down | Itgb1 |
| GO:0023021 | termination of signal transduction | 4.83e-02 | 3.56e-01 | 1 | 22 | 34 | 15137 | 4.55% | 0.22% | 2.94% | 0.15% | CL\_0000822\_down | Arhgef12 |
| GO:0042401 | cellular biogenic amine biosynthetic process | 4.97e-02 | 3.56e-01 | 1 | 22 | 35 | 15137 | 4.55% | 0.23% | 2.86% | 0.15% | CL\_0000822\_down | Srm |
| GO:0002718 | regulation of cytokine production involved in immune response | 4.97e-02 | 3.56e-01 | 1 | 22 | 35 | 15137 | 4.55% | 0.23% | 2.86% | 0.15% | CL\_0000822\_down | Sema7a |
| GO:0006954 | inflammatory response | 5.23e-02 | 3.65e-01 | 2 | 22 | 255 | 15137 | 9.09% | 1.68% | 0.78% | 0.15% | CL\_0000822\_down | Ly96, Sema7a |
| GO:0002637 | regulation of immunoglobulin production | 5.25e-02 | 3.65e-01 | 1 | 22 | 37 | 15137 | 4.55% | 0.24% | 2.70% | 0.15% | CL\_0000822\_down | 2010001M09Rik |
| GO:0008637 | apoptotic mitochondrial changes | 5.52e-02 | 3.78e-01 | 1 | 22 | 39 | 15137 | 4.55% | 0.26% | 2.56% | 0.15% | CL\_0000822\_down | Slc25a4 |
| GO:0030516 | regulation of axon extension | 5.80e-02 | 3.91e-01 | 1 | 22 | 41 | 15137 | 4.55% | 0.27% | 2.44% | 0.15% | CL\_0000822\_down | Sema7a |
| GO:0031344 | regulation of cell projection organization | 5.87e-02 | 3.91e-01 | 2 | 22 | 272 | 15137 | 9.09% | 1.80% | 0.74% | 0.15% | CL\_0000822\_down | Itgb1, Sema7a |
| GO:0048639 | positive regulation of developmental growth | 6.07e-02 | 3.97e-01 | 1 | 22 | 43 | 15137 | 4.55% | 0.28% | 2.33% | 0.15% | CL\_0000822\_down | Sema7a |
| GO:0031032 | actomyosin structure organization | 6.21e-02 | 3.97e-01 | 1 | 22 | 44 | 15137 | 4.55% | 0.29% | 2.27% | 0.15% | CL\_0000822\_down | Itgb1 |
| GO:0043542 | endothelial cell migration | 6.21e-02 | 3.97e-01 | 1 | 22 | 44 | 15137 | 4.55% | 0.29% | 2.27% | 0.15% | CL\_0000822\_down | Itgb1 |
| GO:0050772 | positive regulation of axonogenesis | 6.48e-02 | 4.03e-01 | 1 | 22 | 46 | 15137 | 4.55% | 0.30% | 2.17% | 0.15% | CL\_0000822\_down | Sema7a |
| GO:0061387 | regulation of extent of cell growth | 6.48e-02 | 4.03e-01 | 1 | 22 | 46 | 15137 | 4.55% | 0.30% | 2.17% | 0.15% | CL\_0000822\_down | Sema7a |
| GO:0055007 | cardiac muscle cell differentiation | 6.75e-02 | 4.15e-01 | 1 | 22 | 48 | 15137 | 4.55% | 0.32% | 2.08% | 0.15% | CL\_0000822\_down | Itgb1 |
| GO:0006915 | apoptotic process | 7.14e-02 | 4.26e-01 | 3 | 22 | 671 | 15137 | 13.64% | 4.43% | 0.45% | 0.15% | CL\_0000822\_down | Rps6ka1, Slc25a4, Tfdp1 |
| GO:0010811 | positive regulation of cell-substrate adhesion | 7.43e-02 | 4.26e-01 | 1 | 22 | 53 | 15137 | 4.55% | 0.35% | 1.89% | 0.15% | CL\_0000822\_down | Itgb1 |
| GO:0012501 | programmed cell death | 7.48e-02 | 4.26e-01 | 3 | 22 | 684 | 15137 | 13.64% | 4.52% | 0.44% | 0.15% | CL\_0000822\_down | Rps6ka1, Slc25a4, Tfdp1 |
| GO:0051346 | negative regulation of hydrolase activity | 7.55e-02 | 4.26e-01 | 2 | 22 | 314 | 15137 | 9.09% | 2.07% | 0.64% | 0.15% | CL\_0000822\_down | Rps6ka1, Rrp1b |
| GO:0043408 | regulation of MAPK cascade | 7.55e-02 | 4.26e-01 | 2 | 22 | 314 | 15137 | 9.09% | 2.07% | 0.64% | 0.15% | CL\_0000822\_down | Itgb1, Sema7a |
| GO:0010557 | positive regulation of macromolecule biosynthetic process | 7.56e-02 | 4.26e-01 | 4 | 22 | 1123 | 15137 | 18.18% | 7.42% | 0.36% | 0.15% | CL\_0000822\_down | 2010001M09Rik, Hmgn3, Rps6ka1, Tfdp1 |
| GO:0009593 | detection of chemical stimulus | 7.70e-02 | 4.26e-01 | 1 | 22 | 55 | 15137 | 4.55% | 0.36% | 1.82% | 0.15% | CL\_0000822\_down | Ly96 |
| GO:0006810 | transport | 7.75e-02 | 4.26e-01 | 7 | 22 | 2667 | 15137 | 31.82% | 17.62% | 0.26% | 0.15% | CL\_0000822\_down | Itgb1, Kcnk5, Sfxn1, Slc20a2, Slc25a4, Slc35f2, Slc36a4 |
| GO:0055072 | iron ion homeostasis | 7.84e-02 | 4.26e-01 | 1 | 22 | 56 | 15137 | 4.55% | 0.37% | 1.79% | 0.15% | CL\_0000822\_down | Sfxn1 |
| GO:0045744 | negative regulation of G-protein coupled receptor protein signaling pathway | 7.84e-02 | 4.26e-01 | 1 | 22 | 56 | 15137 | 4.55% | 0.37% | 1.79% | 0.15% | CL\_0000822\_down | Arhgef12 |
| GO:0034329 | cell junction assembly | 8.37e-02 | 4.31e-01 | 1 | 22 | 60 | 15137 | 4.55% | 0.40% | 1.67% | 0.15% | CL\_0000822\_down | Itgb1 |
| GO:0045665 | negative regulation of neuron differentiation | 8.37e-02 | 4.31e-01 | 1 | 22 | 60 | 15137 | 4.55% | 0.40% | 1.67% | 0.15% | CL\_0000822\_down | Itgb1 |
| GO:0010604 | positive regulation of macromolecule metabolic process | 8.42e-02 | 4.31e-01 | 5 | 22 | 1653 | 15137 | 22.73% | 10.92% | 0.30% | 0.15% | CL\_0000822\_down | 2010001M09Rik, Hmgn3, Itgb1, Rps6ka1, Tfdp1 |
| GO:0051234 | establishment of localization | 8.44e-02 | 4.31e-01 | 7 | 22 | 2719 | 15137 | 31.82% | 17.96% | 0.26% | 0.15% | CL\_0000822\_down | Itgb1, Kcnk5, Sfxn1, Slc20a2, Slc25a4, Slc35f2, Slc36a4 |
| GO:0000082 | G1/S transition of mitotic cell cycle | 8.51e-02 | 4.31e-01 | 1 | 22 | 61 | 15137 | 4.55% | 0.40% | 1.64% | 0.15% | CL\_0000822\_down | Itgb1 |
| GO:0010923 | negative regulation of phosphatase activity | 8.51e-02 | 4.31e-01 | 1 | 22 | 61 | 15137 | 4.55% | 0.40% | 1.64% | 0.15% | CL\_0000822\_down | Rrp1b |
| GO:0045216 | cell-cell junction organization | 8.64e-02 | 4.31e-01 | 1 | 22 | 62 | 15137 | 4.55% | 0.41% | 1.61% | 0.15% | CL\_0000822\_down | Itgb1 |
| GO:0001708 | cell fate specification | 8.77e-02 | 4.31e-01 | 1 | 22 | 63 | 15137 | 4.55% | 0.42% | 1.59% | 0.15% | CL\_0000822\_down | Itgb1 |
| GO:0010740 | positive regulation of intracellular protein kinase cascade | 8.96e-02 | 4.31e-01 | 2 | 22 | 347 | 15137 | 9.09% | 2.29% | 0.58% | 0.15% | CL\_0000822\_down | Itgb1, Sema7a |
| GO:0051179 | localization | 8.97e-02 | 4.31e-01 | 8 | 22 | 3328 | 15137 | 36.36% | 21.99% | 0.24% | 0.15% | CL\_0000822\_down | Itgb1, Kcnk5, Sfxn1, Slc20a2, Slc25a4, Slc35f2, Slc36a4, Tmem18 |
| GO:0043154 | negative regulation of cysteine-type endopeptidase activity involved in apoptotic process | 9.04e-02 | 4.31e-01 | 1 | 22 | 65 | 15137 | 4.55% | 0.43% | 1.54% | 0.15% | CL\_0000822\_down | Rps6ka1 |
| GO:0008219 | cell death | 9.05e-02 | 4.31e-01 | 3 | 22 | 742 | 15137 | 13.64% | 4.90% | 0.40% | 0.15% | CL\_0000822\_down | Rps6ka1, Slc25a4, Tfdp1 |
| GO:0045664 | regulation of neuron differentiation | 9.23e-02 | 4.31e-01 | 2 | 22 | 353 | 15137 | 9.09% | 2.33% | 0.57% | 0.15% | CL\_0000822\_down | Itgb1, Sema7a |
| GO:0016265 | death | 9.30e-02 | 4.31e-01 | 3 | 22 | 751 | 15137 | 13.64% | 4.96% | 0.40% | 0.15% | CL\_0000822\_down | Rps6ka1, Slc25a4, Tfdp1 |
| GO:0031345 | negative regulation of cell projection organization | 9.44e-02 | 4.31e-01 | 1 | 22 | 68 | 15137 | 4.55% | 0.45% | 1.47% | 0.15% | CL\_0000822\_down | Itgb1 |
| GO:2000117 | negative regulation of cysteine-type endopeptidase activity | 9.44e-02 | 4.31e-01 | 1 | 22 | 68 | 15137 | 4.55% | 0.45% | 1.47% | 0.15% | CL\_0000822\_down | Rps6ka1 |
| GO:0055080 | cation homeostasis | 9.49e-02 | 4.31e-01 | 2 | 22 | 359 | 15137 | 9.09% | 2.37% | 0.56% | 0.15% | CL\_0000822\_down | Itgb1, Sfxn1 |
| GO:0000041 | transition metal ion transport | 9.57e-02 | 4.31e-01 | 1 | 22 | 69 | 15137 | 4.55% | 0.46% | 1.45% | 0.15% | CL\_0000822\_down | Sfxn1 |
| GO:0030218 | erythrocyte differentiation | 9.70e-02 | 4.32e-01 | 1 | 22 | 70 | 15137 | 4.55% | 0.46% | 1.43% | 0.15% | CL\_0000822\_down | Sfxn1 |
| GO:0009891 | positive regulation of biosynthetic process | 1.01e-01 | 4.32e-01 | 4 | 22 | 1241 | 15137 | 18.18% | 8.20% | 0.32% | 0.15% | CL\_0000822\_down | 2010001M09Rik, Hmgn3, Rps6ka1, Tfdp1 |
| GO:0006811 | ion transport | 1.01e-01 | 4.32e-01 | 3 | 22 | 778 | 15137 | 13.64% | 5.14% | 0.39% | 0.15% | CL\_0000822\_down | Kcnk5, Sfxn1, Slc20a2 |
| GO:0007160 | cell-matrix adhesion | 1.01e-01 | 4.32e-01 | 1 | 22 | 73 | 15137 | 4.55% | 0.48% | 1.37% | 0.15% | CL\_0000822\_down | Itgb1 |
| GO:0010976 | positive regulation of neuron projection development | 1.01e-01 | 4.32e-01 | 1 | 22 | 73 | 15137 | 4.55% | 0.48% | 1.37% | 0.15% | CL\_0000822\_down | Itgb1 |
| GO:0000278 | mitotic cell cycle | 1.02e-01 | 4.32e-01 | 2 | 22 | 374 | 15137 | 9.09% | 2.47% | 0.53% | 0.15% | CL\_0000822\_down | Itgb1, Tfdp1 |
| GO:0035051 | cardiac cell differentiation | 1.02e-01 | 4.32e-01 | 1 | 22 | 74 | 15137 | 4.55% | 0.49% | 1.35% | 0.15% | CL\_0000822\_down | Itgb1 |
| GO:0045666 | positive regulation of neuron differentiation | 1.06e-01 | 4.41e-01 | 1 | 22 | 77 | 15137 | 4.55% | 0.51% | 1.30% | 0.15% | CL\_0000822\_down | Itgb1 |
| GO:0008361 | regulation of cell size | 1.06e-01 | 4.41e-01 | 1 | 22 | 77 | 15137 | 4.55% | 0.51% | 1.30% | 0.15% | CL\_0000822\_down | Sema7a |
| GO:0034101 | erythrocyte homeostasis | 1.08e-01 | 4.42e-01 | 1 | 22 | 78 | 15137 | 4.55% | 0.52% | 1.28% | 0.15% | CL\_0000822\_down | Sfxn1 |
| GO:0009893 | positive regulation of metabolic process | 1.09e-01 | 4.46e-01 | 5 | 22 | 1788 | 15137 | 22.73% | 11.81% | 0.28% | 0.15% | CL\_0000822\_down | 2010001M09Rik, Hmgn3, Itgb1, Rps6ka1, Tfdp1 |
| GO:0045807 | positive regulation of endocytosis | 1.11e-01 | 4.50e-01 | 1 | 22 | 81 | 15137 | 4.55% | 0.54% | 1.23% | 0.15% | CL\_0000822\_down | Itgb1 |
| GO:0070374 | positive regulation of ERK1 and ERK2 cascade | 1.15e-01 | 4.62e-01 | 1 | 22 | 84 | 15137 | 4.55% | 0.55% | 1.19% | 0.15% | CL\_0000822\_down | Sema7a |
| GO:0001894 | tissue homeostasis | 1.18e-01 | 4.69e-01 | 1 | 22 | 86 | 15137 | 4.55% | 0.57% | 1.16% | 0.15% | CL\_0000822\_down | Itgb1 |
| GO:0055002 | striated muscle cell development | 1.20e-01 | 4.75e-01 | 1 | 22 | 88 | 15137 | 4.55% | 0.58% | 1.14% | 0.15% | CL\_0000822\_down | Itgb1 |
| GO:0010921 | regulation of phosphatase activity | 1.22e-01 | 4.76e-01 | 1 | 22 | 89 | 15137 | 4.55% | 0.59% | 1.12% | 0.15% | CL\_0000822\_down | Rrp1b |
| GO:0010646 | regulation of cell communication | 1.24e-01 | 4.78e-01 | 4 | 22 | 1337 | 15137 | 18.18% | 8.83% | 0.30% | 0.15% | CL\_0000822\_down | Arhgef12, Hmgn3, Itgb1, Sema7a |
| GO:0034330 | cell junction organization | 1.24e-01 | 4.78e-01 | 1 | 22 | 91 | 15137 | 4.55% | 0.60% | 1.10% | 0.15% | CL\_0000822\_down | Itgb1 |
| GO:0050767 | regulation of neurogenesis | 1.26e-01 | 4.82e-01 | 2 | 22 | 426 | 15137 | 9.09% | 2.81% | 0.47% | 0.15% | CL\_0000822\_down | Itgb1, Sema7a |
| GO:0010810 | regulation of cell-substrate adhesion | 1.28e-01 | 4.82e-01 | 1 | 22 | 94 | 15137 | 4.55% | 0.62% | 1.06% | 0.15% | CL\_0000822\_down | Itgb1 |
| GO:0015931 | nucleobase-containing compound transport | 1.31e-01 | 4.82e-01 | 1 | 22 | 96 | 15137 | 4.55% | 0.63% | 1.04% | 0.15% | CL\_0000822\_down | Slc25a4 |
| GO:0006865 | amino acid transport | 1.32e-01 | 4.82e-01 | 1 | 22 | 97 | 15137 | 4.55% | 0.64% | 1.03% | 0.15% | CL\_0000822\_down | Slc36a4 |
| GO:0050770 | regulation of axonogenesis | 1.32e-01 | 4.82e-01 | 1 | 22 | 97 | 15137 | 4.55% | 0.64% | 1.03% | 0.15% | CL\_0000822\_down | Sema7a |
| GO:0006576 | cellular biogenic amine metabolic process | 1.33e-01 | 4.82e-01 | 1 | 22 | 98 | 15137 | 4.55% | 0.65% | 1.02% | 0.15% | CL\_0000822\_down | Srm |
| GO:0030307 | positive regulation of cell growth | 1.33e-01 | 4.82e-01 | 1 | 22 | 98 | 15137 | 4.55% | 0.65% | 1.02% | 0.15% | CL\_0000822\_down | Sema7a |
| GO:0044419 | interspecies interaction between organisms | 1.35e-01 | 4.82e-01 | 1 | 22 | 99 | 15137 | 4.55% | 0.65% | 1.01% | 0.15% | CL\_0000822\_down | Slc20a2 |
| GO:0009611 | response to wounding | 1.37e-01 | 4.82e-01 | 2 | 22 | 448 | 15137 | 9.09% | 2.96% | 0.45% | 0.15% | CL\_0000822\_down | Ly96, Sema7a |
| GO:0006364 | rRNA processing | 1.40e-01 | 4.82e-01 | 1 | 22 | 103 | 15137 | 4.55% | 0.68% | 0.97% | 0.15% | CL\_0000822\_down | Rrp1b |
| GO:0050796 | regulation of insulin secretion | 1.40e-01 | 4.82e-01 | 1 | 22 | 103 | 15137 | 4.55% | 0.68% | 0.97% | 0.15% | CL\_0000822\_down | Hmgn3 |
| GO:0071222 | cellular response to lipopolysaccharide | 1.45e-01 | 4.82e-01 | 1 | 22 | 107 | 15137 | 4.55% | 0.71% | 0.93% | 0.15% | CL\_0000822\_down | Ly96 |
| GO:0016072 | rRNA metabolic process | 1.45e-01 | 4.82e-01 | 1 | 22 | 107 | 15137 | 4.55% | 0.71% | 0.93% | 0.15% | CL\_0000822\_down | Rrp1b |
| GO:0030001 | metal ion transport | 1.46e-01 | 4.82e-01 | 2 | 22 | 465 | 15137 | 9.09% | 3.07% | 0.43% | 0.15% | CL\_0000822\_down | Sfxn1, Slc20a2 |
| GO:0048738 | cardiac muscle tissue development | 1.46e-01 | 4.82e-01 | 1 | 22 | 108 | 15137 | 4.55% | 0.71% | 0.93% | 0.15% | CL\_0000822\_down | Itgb1 |
| GO:0031589 | cell-substrate adhesion | 1.46e-01 | 4.82e-01 | 1 | 22 | 108 | 15137 | 4.55% | 0.71% | 0.93% | 0.15% | CL\_0000822\_down | Itgb1 |
| GO:0015698 | inorganic anion transport | 1.46e-01 | 4.82e-01 | 1 | 22 | 108 | 15137 | 4.55% | 0.71% | 0.93% | 0.15% | CL\_0000822\_down | Slc20a2 |
| GO:0035303 | regulation of dephosphorylation | 1.46e-01 | 4.82e-01 | 1 | 22 | 108 | 15137 | 4.55% | 0.71% | 0.93% | 0.15% | CL\_0000822\_down | Rrp1b |
| GO:0055001 | muscle cell development | 1.47e-01 | 4.82e-01 | 1 | 22 | 109 | 15137 | 4.55% | 0.72% | 0.92% | 0.15% | CL\_0000822\_down | Itgb1 |
| GO:0048638 | regulation of developmental growth | 1.47e-01 | 4.82e-01 | 1 | 22 | 109 | 15137 | 4.55% | 0.72% | 0.92% | 0.15% | CL\_0000822\_down | Sema7a |
| GO:0051960 | regulation of nervous system development | 1.49e-01 | 4.82e-01 | 2 | 22 | 472 | 15137 | 9.09% | 3.12% | 0.42% | 0.15% | CL\_0000822\_down | Itgb1, Sema7a |
| GO:0070372 | regulation of ERK1 and ERK2 cascade | 1.52e-01 | 4.82e-01 | 1 | 22 | 113 | 15137 | 4.55% | 0.75% | 0.88% | 0.15% | CL\_0000822\_down | Sema7a |
| GO:0016477 | cell migration | 1.53e-01 | 4.82e-01 | 2 | 22 | 479 | 15137 | 9.09% | 3.16% | 0.42% | 0.15% | CL\_0000822\_down | Itgb1, Tmem18 |
| GO:0022403 | cell cycle phase | 1.53e-01 | 4.82e-01 | 2 | 22 | 480 | 15137 | 9.09% | 3.17% | 0.42% | 0.15% | CL\_0000822\_down | Itgb1, Tfdp1 |
| GO:0090276 | regulation of peptide hormone secretion | 1.53e-01 | 4.82e-01 | 1 | 22 | 114 | 15137 | 4.55% | 0.75% | 0.88% | 0.15% | CL\_0000822\_down | Hmgn3 |
| GO:0071219 | cellular response to molecule of bacterial origin | 1.55e-01 | 4.82e-01 | 1 | 22 | 115 | 15137 | 4.55% | 0.76% | 0.87% | 0.15% | CL\_0000822\_down | Ly96 |
| GO:0002699 | positive regulation of immune effector process | 1.55e-01 | 4.82e-01 | 1 | 22 | 115 | 15137 | 4.55% | 0.76% | 0.87% | 0.15% | CL\_0000822\_down | Sema7a |
| GO:0045597 | positive regulation of cell differentiation | 1.55e-01 | 4.82e-01 | 2 | 22 | 484 | 15137 | 9.09% | 3.20% | 0.41% | 0.15% | CL\_0000822\_down | Itgb1, Sema7a |
| GO:0002791 | regulation of peptide secretion | 1.56e-01 | 4.82e-01 | 1 | 22 | 116 | 15137 | 4.55% | 0.77% | 0.86% | 0.15% | CL\_0000822\_down | Hmgn3 |
| GO:0090087 | regulation of peptide transport | 1.56e-01 | 4.82e-01 | 1 | 22 | 116 | 15137 | 4.55% | 0.77% | 0.86% | 0.15% | CL\_0000822\_down | Hmgn3 |
| GO:0051130 | positive regulation of cellular component organization | 1.59e-01 | 4.84e-01 | 2 | 22 | 491 | 15137 | 9.09% | 3.24% | 0.41% | 0.15% | CL\_0000822\_down | Itgb1, Sema7a |
| GO:0048871 | multicellular organismal homeostasis | 1.59e-01 | 4.84e-01 | 1 | 22 | 119 | 15137 | 4.55% | 0.79% | 0.84% | 0.15% | CL\_0000822\_down | Itgb1 |
| GO:0045785 | positive regulation of cell adhesion | 1.59e-01 | 4.84e-01 | 1 | 22 | 119 | 15137 | 4.55% | 0.79% | 0.84% | 0.15% | CL\_0000822\_down | Itgb1 |
| GO:0009309 | amine biosynthetic process | 1.61e-01 | 4.84e-01 | 1 | 22 | 120 | 15137 | 4.55% | 0.79% | 0.83% | 0.15% | CL\_0000822\_down | Srm |
| GO:0010927 | cellular component assembly involved in morphogenesis | 1.63e-01 | 4.85e-01 | 1 | 22 | 122 | 15137 | 4.55% | 0.81% | 0.82% | 0.15% | CL\_0000822\_down | Itgb1 |
| GO:0050731 | positive regulation of peptidyl-tyrosine phosphorylation | 1.63e-01 | 4.85e-01 | 1 | 22 | 122 | 15137 | 4.55% | 0.81% | 0.82% | 0.15% | CL\_0000822\_down | Itgb1 |
| GO:0071216 | cellular response to biotic stimulus | 1.69e-01 | 4.97e-01 | 1 | 22 | 127 | 15137 | 4.55% | 0.84% | 0.79% | 0.15% | CL\_0000822\_down | Ly96 |
| GO:0045893 | positive regulation of transcription, DNA-dependent | 1.70e-01 | 4.97e-01 | 3 | 22 | 986 | 15137 | 13.64% | 6.51% | 0.30% | 0.15% | CL\_0000822\_down | Hmgn3, Rps6ka1, Tfdp1 |
| GO:0006814 | sodium ion transport | 1.71e-01 | 4.97e-01 | 1 | 22 | 128 | 15137 | 4.55% | 0.85% | 0.78% | 0.15% | CL\_0000822\_down | Slc20a2 |
| GO:0060284 | regulation of cell development | 1.71e-01 | 4.97e-01 | 2 | 22 | 515 | 15137 | 9.09% | 3.40% | 0.39% | 0.15% | CL\_0000822\_down | Itgb1, Sema7a |
| GO:0030100 | regulation of endocytosis | 1.74e-01 | 5.02e-01 | 1 | 22 | 131 | 15137 | 4.55% | 0.87% | 0.76% | 0.15% | CL\_0000822\_down | Itgb1 |
| GO:0051254 | positive regulation of RNA metabolic process | 1.78e-01 | 5.05e-01 | 3 | 22 | 1008 | 15137 | 13.64% | 6.66% | 0.30% | 0.15% | CL\_0000822\_down | Hmgn3, Rps6ka1, Tfdp1 |
| GO:0060249 | anatomical structure homeostasis | 1.78e-01 | 5.05e-01 | 1 | 22 | 134 | 15137 | 4.55% | 0.89% | 0.75% | 0.15% | CL\_0000822\_down | Itgb1 |
| GO:0010627 | regulation of intracellular protein kinase cascade | 1.79e-01 | 5.05e-01 | 2 | 22 | 530 | 15137 | 9.09% | 3.50% | 0.38% | 0.15% | CL\_0000822\_down | Itgb1, Sema7a |
| GO:0035023 | regulation of Rho protein signal transduction | 1.81e-01 | 5.05e-01 | 1 | 22 | 137 | 15137 | 4.55% | 0.91% | 0.73% | 0.15% | CL\_0000822\_down | Arhgef12 |
| GO:0048870 | cell motility | 1.82e-01 | 5.05e-01 | 2 | 22 | 536 | 15137 | 9.09% | 3.54% | 0.37% | 0.15% | CL\_0000822\_down | Itgb1, Tmem18 |
| GO:0051674 | localization of cell | 1.82e-01 | 5.05e-01 | 2 | 22 | 536 | 15137 | 9.09% | 3.54% | 0.37% | 0.15% | CL\_0000822\_down | Itgb1, Tmem18 |
| GO:0015837 | amine transport | 1.83e-01 | 5.05e-01 | 1 | 22 | 138 | 15137 | 4.55% | 0.91% | 0.72% | 0.15% | CL\_0000822\_down | Slc36a4 |
| GO:0050769 | positive regulation of neurogenesis | 1.86e-01 | 5.12e-01 | 1 | 22 | 141 | 15137 | 4.55% | 0.93% | 0.71% | 0.15% | CL\_0000822\_down | Sema7a |
| GO:0043086 | negative regulation of catalytic activity | 1.88e-01 | 5.13e-01 | 2 | 22 | 547 | 15137 | 9.09% | 3.61% | 0.37% | 0.15% | CL\_0000822\_down | Rps6ka1, Rrp1b |
| GO:0051606 | detection of stimulus | 1.89e-01 | 5.13e-01 | 1 | 22 | 143 | 15137 | 4.55% | 0.94% | 0.70% | 0.15% | CL\_0000822\_down | Ly96 |
| GO:0019221 | cytokine-mediated signaling pathway | 1.93e-01 | 5.22e-01 | 1 | 22 | 147 | 15137 | 4.55% | 0.97% | 0.68% | 0.15% | CL\_0000822\_down | Ccbp2 |
| GO:0030099 | myeloid cell differentiation | 1.96e-01 | 5.22e-01 | 1 | 22 | 149 | 15137 | 4.55% | 0.98% | 0.67% | 0.15% | CL\_0000822\_down | Sfxn1 |
| GO:0010951 | negative regulation of endopeptidase activity | 1.97e-01 | 5.22e-01 | 1 | 22 | 150 | 15137 | 4.55% | 0.99% | 0.67% | 0.15% | CL\_0000822\_down | Rps6ka1 |
| GO:0051091 | positive regulation of sequence-specific DNA binding transcription factor activity | 1.98e-01 | 5.22e-01 | 1 | 22 | 151 | 15137 | 4.55% | 1.00% | 0.66% | 0.15% | CL\_0000822\_down | Hmgn3 |
| GO:0051146 | striated muscle cell differentiation | 1.98e-01 | 5.22e-01 | 1 | 22 | 151 | 15137 | 4.55% | 1.00% | 0.66% | 0.15% | CL\_0000822\_down | Itgb1 |
| GO:0050801 | ion homeostasis | 1.99e-01 | 5.22e-01 | 2 | 22 | 568 | 15137 | 9.09% | 3.75% | 0.35% | 0.15% | CL\_0000822\_down | Itgb1, Sfxn1 |
| GO:0046883 | regulation of hormone secretion | 2.00e-01 | 5.22e-01 | 1 | 22 | 153 | 15137 | 4.55% | 1.01% | 0.65% | 0.15% | CL\_0000822\_down | Hmgn3 |
| GO:0010628 | positive regulation of gene expression | 2.04e-01 | 5.22e-01 | 3 | 22 | 1078 | 15137 | 13.64% | 7.12% | 0.28% | 0.15% | CL\_0000822\_down | Hmgn3, Rps6ka1, Tfdp1 |
| GO:0042254 | ribosome biogenesis | 2.04e-01 | 5.22e-01 | 1 | 22 | 156 | 15137 | 4.55% | 1.03% | 0.64% | 0.15% | CL\_0000822\_down | Rrp1b |
| GO:0046942 | carboxylic acid transport | 2.05e-01 | 5.22e-01 | 1 | 22 | 157 | 15137 | 4.55% | 1.04% | 0.64% | 0.15% | CL\_0000822\_down | Slc36a4 |
| GO:0045927 | positive regulation of growth | 2.06e-01 | 5.22e-01 | 1 | 22 | 158 | 15137 | 4.55% | 1.04% | 0.63% | 0.15% | CL\_0000822\_down | Sema7a |
| GO:0006812 | cation transport | 2.07e-01 | 5.22e-01 | 2 | 22 | 582 | 15137 | 9.09% | 3.84% | 0.34% | 0.15% | CL\_0000822\_down | Sfxn1, Slc20a2 |
| GO:0015849 | organic acid transport | 2.07e-01 | 5.22e-01 | 1 | 22 | 159 | 15137 | 4.55% | 1.05% | 0.63% | 0.15% | CL\_0000822\_down | Slc36a4 |
| GO:0043281 | regulation of cysteine-type endopeptidase activity involved in apoptotic process | 2.07e-01 | 5.22e-01 | 1 | 22 | 159 | 15137 | 4.55% | 1.05% | 0.63% | 0.15% | CL\_0000822\_down | Rps6ka1 |
| GO:0006820 | anion transport | 2.10e-01 | 5.22e-01 | 1 | 22 | 161 | 15137 | 4.55% | 1.06% | 0.62% | 0.15% | CL\_0000822\_down | Slc20a2 |
| GO:0050730 | regulation of peptidyl-tyrosine phosphorylation | 2.10e-01 | 5.22e-01 | 1 | 22 | 161 | 15137 | 4.55% | 1.06% | 0.62% | 0.15% | CL\_0000822\_down | Itgb1 |
| GO:2000116 | regulation of cysteine-type endopeptidase activity | 2.11e-01 | 5.22e-01 | 1 | 22 | 162 | 15137 | 4.55% | 1.07% | 0.62% | 0.15% | CL\_0000822\_down | Rps6ka1 |
| GO:0022402 | cell cycle process | 2.12e-01 | 5.22e-01 | 2 | 22 | 593 | 15137 | 9.09% | 3.92% | 0.34% | 0.15% | CL\_0000822\_down | Itgb1, Tfdp1 |
| GO:0006952 | defense response | 2.14e-01 | 5.22e-01 | 2 | 22 | 596 | 15137 | 9.09% | 3.94% | 0.34% | 0.15% | CL\_0000822\_down | Ly96, Sema7a |
| GO:0050727 | regulation of inflammatory response | 2.16e-01 | 5.22e-01 | 1 | 22 | 166 | 15137 | 4.55% | 1.10% | 0.60% | 0.15% | CL\_0000822\_down | Sema7a |
| GO:0048872 | homeostasis of number of cells | 2.17e-01 | 5.22e-01 | 1 | 22 | 167 | 15137 | 4.55% | 1.10% | 0.60% | 0.15% | CL\_0000822\_down | Sfxn1 |
| GO:0032535 | regulation of cellular component size | 2.17e-01 | 5.22e-01 | 1 | 22 | 167 | 15137 | 4.55% | 1.10% | 0.60% | 0.15% | CL\_0000822\_down | Sema7a |
| GO:0010941 | regulation of cell death | 2.17e-01 | 5.22e-01 | 3 | 22 | 1114 | 15137 | 13.64% | 7.36% | 0.27% | 0.15% | CL\_0000822\_down | Itgb1, Rps6ka1, Slc25a4 |
| GO:0010769 | regulation of cell morphogenesis involved in differentiation | 2.20e-01 | 5.25e-01 | 1 | 22 | 170 | 15137 | 4.55% | 1.12% | 0.59% | 0.15% | CL\_0000822\_down | Sema7a |
| GO:0010720 | positive regulation of cell development | 2.22e-01 | 5.25e-01 | 1 | 22 | 172 | 15137 | 4.55% | 1.14% | 0.58% | 0.15% | CL\_0000822\_down | Sema7a |
| GO:0031325 | positive regulation of cellular metabolic process | 2.22e-01 | 5.25e-01 | 4 | 22 | 1680 | 15137 | 18.18% | 11.10% | 0.24% | 0.15% | CL\_0000822\_down | Hmgn3, Itgb1, Rps6ka1, Tfdp1 |
| GO:0060548 | negative regulation of cell death | 2.23e-01 | 5.25e-01 | 2 | 22 | 613 | 15137 | 9.09% | 4.05% | 0.33% | 0.15% | CL\_0000822\_down | Rps6ka1, Slc25a4 |
| GO:0045935 | positive regulation of nucleobase-containing compound metabolic process | 2.26e-01 | 5.30e-01 | 3 | 22 | 1137 | 15137 | 13.64% | 7.51% | 0.26% | 0.15% | CL\_0000822\_down | Hmgn3, Rps6ka1, Tfdp1 |
| GO:0001819 | positive regulation of cytokine production | 2.29e-01 | 5.34e-01 | 1 | 22 | 178 | 15137 | 4.55% | 1.18% | 0.56% | 0.15% | CL\_0000822\_down | Sema7a |
| GO:0032496 | response to lipopolysaccharide | 2.32e-01 | 5.37e-01 | 1 | 22 | 180 | 15137 | 4.55% | 1.19% | 0.56% | 0.15% | CL\_0000822\_down | Ly96 |
| GO:0007155 | cell adhesion | 2.33e-01 | 5.37e-01 | 2 | 22 | 631 | 15137 | 9.09% | 4.17% | 0.32% | 0.15% | CL\_0000822\_down | Emb, Itgb1 |
| GO:0007005 | mitochondrion organization | 2.34e-01 | 5.37e-01 | 1 | 22 | 182 | 15137 | 4.55% | 1.20% | 0.55% | 0.15% | CL\_0000822\_down | Slc25a4 |
| GO:0051173 | positive regulation of nitrogen compound metabolic process | 2.35e-01 | 5.37e-01 | 3 | 22 | 1160 | 15137 | 13.64% | 7.66% | 0.26% | 0.15% | CL\_0000822\_down | Hmgn3, Rps6ka1, Tfdp1 |
| GO:0022610 | biological adhesion | 2.36e-01 | 5.37e-01 | 2 | 22 | 637 | 15137 | 9.09% | 4.21% | 0.31% | 0.15% | CL\_0000822\_down | Emb, Itgb1 |
| GO:0023051 | regulation of signaling | 2.41e-01 | 5.44e-01 | 4 | 22 | 1738 | 15137 | 18.18% | 11.48% | 0.23% | 0.15% | CL\_0000822\_down | Arhgef12, Hmgn3, Itgb1, Sema7a |
| GO:0050867 | positive regulation of cell activation | 2.42e-01 | 5.44e-01 | 1 | 22 | 189 | 15137 | 4.55% | 1.25% | 0.53% | 0.15% | CL\_0000822\_down | Rps6ka1 |
| GO:0045087 | innate immune response | 2.46e-01 | 5.47e-01 | 1 | 22 | 193 | 15137 | 4.55% | 1.28% | 0.52% | 0.15% | CL\_0000822\_down | Ly96 |
| GO:0008544 | epidermis development | 2.48e-01 | 5.47e-01 | 1 | 22 | 195 | 15137 | 4.55% | 1.29% | 0.51% | 0.15% | CL\_0000822\_down | Tfdp1 |
| GO:0034470 | ncRNA processing | 2.48e-01 | 5.47e-01 | 1 | 22 | 195 | 15137 | 4.55% | 1.29% | 0.51% | 0.15% | CL\_0000822\_down | Rrp1b |
| GO:0002682 | regulation of immune system process | 2.49e-01 | 5.47e-01 | 2 | 22 | 660 | 15137 | 9.09% | 4.36% | 0.30% | 0.15% | CL\_0000822\_down | 2010001M09Rik, Sema7a |
| GO:0009967 | positive regulation of signal transduction | 2.50e-01 | 5.47e-01 | 2 | 22 | 663 | 15137 | 9.09% | 4.38% | 0.30% | 0.15% | CL\_0000822\_down | Itgb1, Sema7a |
| GO:0055085 | transmembrane transport | 2.53e-01 | 5.47e-01 | 2 | 22 | 667 | 15137 | 9.09% | 4.41% | 0.30% | 0.15% | CL\_0000822\_down | Sfxn1, Slc25a4 |
| GO:0045165 | cell fate commitment | 2.53e-01 | 5.47e-01 | 1 | 22 | 199 | 15137 | 4.55% | 1.31% | 0.50% | 0.15% | CL\_0000822\_down | Itgb1 |
| GO:0051704 | multi-organism process | 2.53e-01 | 5.47e-01 | 2 | 22 | 668 | 15137 | 9.09% | 4.41% | 0.30% | 0.15% | CL\_0000822\_down | Ly96, Slc20a2 |
| GO:0002237 | response to molecule of bacterial origin | 2.54e-01 | 5.47e-01 | 1 | 22 | 200 | 15137 | 4.55% | 1.32% | 0.50% | 0.15% | CL\_0000822\_down | Ly96 |
| GO:0006928 | cellular component movement | 2.55e-01 | 5.47e-01 | 2 | 22 | 671 | 15137 | 9.09% | 4.43% | 0.30% | 0.15% | CL\_0000822\_down | Itgb1, Tmem18 |
| GO:0051094 | positive regulation of developmental process | 2.55e-01 | 5.47e-01 | 2 | 22 | 672 | 15137 | 9.09% | 4.44% | 0.30% | 0.15% | CL\_0000822\_down | Itgb1, Sema7a |
| GO:0031328 | positive regulation of cellular biosynthetic process | 2.58e-01 | 5.48e-01 | 3 | 22 | 1216 | 15137 | 13.64% | 8.03% | 0.25% | 0.15% | CL\_0000822\_down | Hmgn3, Rps6ka1, Tfdp1 |
| GO:0022613 | ribonucleoprotein complex biogenesis | 2.58e-01 | 5.48e-01 | 1 | 22 | 204 | 15137 | 4.55% | 1.35% | 0.49% | 0.15% | CL\_0000822\_down | Rrp1b |
| GO:0042692 | muscle cell differentiation | 2.62e-01 | 5.52e-01 | 1 | 22 | 207 | 15137 | 4.55% | 1.37% | 0.48% | 0.15% | CL\_0000822\_down | Itgb1 |
| GO:0051336 | regulation of hydrolase activity | 2.64e-01 | 5.54e-01 | 2 | 22 | 687 | 15137 | 9.09% | 4.54% | 0.29% | 0.15% | CL\_0000822\_down | Rps6ka1, Rrp1b |
| GO:0071843 | cellular component biogenesis at cellular level | 2.70e-01 | 5.60e-01 | 1 | 22 | 215 | 15137 | 4.55% | 1.42% | 0.47% | 0.15% | CL\_0000822\_down | Rrp1b |
| GO:0071345 | cellular response to cytokine stimulus | 2.70e-01 | 5.60e-01 | 1 | 22 | 215 | 15137 | 4.55% | 1.42% | 0.47% | 0.15% | CL\_0000822\_down | Ccbp2 |
| GO:0010466 | negative regulation of peptidase activity | 2.71e-01 | 5.60e-01 | 1 | 22 | 216 | 15137 | 4.55% | 1.43% | 0.46% | 0.15% | CL\_0000822\_down | Rps6ka1 |
| GO:0040011 | locomotion | 2.71e-01 | 5.60e-01 | 2 | 22 | 701 | 15137 | 9.09% | 4.63% | 0.29% | 0.15% | CL\_0000822\_down | Itgb1, Tmem18 |
| GO:0014706 | striated muscle tissue development | 2.73e-01 | 5.60e-01 | 1 | 22 | 218 | 15137 | 4.55% | 1.44% | 0.46% | 0.15% | CL\_0000822\_down | Itgb1 |
| GO:0044092 | negative regulation of molecular function | 2.73e-01 | 5.60e-01 | 2 | 22 | 705 | 15137 | 9.09% | 4.66% | 0.28% | 0.15% | CL\_0000822\_down | Rps6ka1, Rrp1b |
| GO:0045944 | positive regulation of transcription from RNA polymerase II promoter | 2.75e-01 | 5.60e-01 | 2 | 22 | 707 | 15137 | 9.09% | 4.67% | 0.28% | 0.15% | CL\_0000822\_down | Hmgn3, Rps6ka1 |
| GO:0010647 | positive regulation of cell communication | 2.78e-01 | 5.60e-01 | 2 | 22 | 714 | 15137 | 9.09% | 4.72% | 0.28% | 0.15% | CL\_0000822\_down | Itgb1, Sema7a |
| GO:0001525 | angiogenesis | 2.79e-01 | 5.60e-01 | 1 | 22 | 223 | 15137 | 4.55% | 1.47% | 0.45% | 0.15% | CL\_0000822\_down | Itgb1 |
| GO:0030335 | positive regulation of cell migration | 2.79e-01 | 5.60e-01 | 1 | 22 | 223 | 15137 | 4.55% | 1.47% | 0.45% | 0.15% | CL\_0000822\_down | Itgb1 |
| GO:0060627 | regulation of vesicle-mediated transport | 2.80e-01 | 5.60e-01 | 1 | 22 | 224 | 15137 | 4.55% | 1.48% | 0.45% | 0.15% | CL\_0000822\_down | Itgb1 |
| GO:0006874 | cellular calcium ion homeostasis | 2.81e-01 | 5.60e-01 | 1 | 22 | 225 | 15137 | 4.55% | 1.49% | 0.44% | 0.15% | CL\_0000822\_down | Itgb1 |
| GO:0023056 | positive regulation of signaling | 2.82e-01 | 5.60e-01 | 2 | 22 | 721 | 15137 | 9.09% | 4.76% | 0.28% | 0.15% | CL\_0000822\_down | Itgb1, Sema7a |
| GO:0048878 | chemical homeostasis | 2.83e-01 | 5.60e-01 | 2 | 22 | 723 | 15137 | 9.09% | 4.78% | 0.28% | 0.15% | CL\_0000822\_down | Itgb1, Sfxn1 |
| GO:2000147 | positive regulation of cell motility | 2.84e-01 | 5.60e-01 | 1 | 22 | 228 | 15137 | 4.55% | 1.51% | 0.44% | 0.15% | CL\_0000822\_down | Itgb1 |
| GO:0090066 | regulation of anatomical structure size | 2.86e-01 | 5.62e-01 | 1 | 22 | 230 | 15137 | 4.55% | 1.52% | 0.43% | 0.15% | CL\_0000822\_down | Sema7a |
| GO:0055074 | calcium ion homeostasis | 2.90e-01 | 5.64e-01 | 1 | 22 | 234 | 15137 | 4.55% | 1.55% | 0.43% | 0.15% | CL\_0000822\_down | Itgb1 |
| GO:0060537 | muscle tissue development | 2.91e-01 | 5.64e-01 | 1 | 22 | 235 | 15137 | 4.55% | 1.55% | 0.43% | 0.15% | CL\_0000822\_down | Itgb1 |
| GO:0051272 | positive regulation of cellular component movement | 2.91e-01 | 5.64e-01 | 1 | 22 | 235 | 15137 | 4.55% | 1.55% | 0.43% | 0.15% | CL\_0000822\_down | Itgb1 |
| GO:0016337 | cell-cell adhesion | 2.95e-01 | 5.64e-01 | 1 | 22 | 238 | 15137 | 4.55% | 1.57% | 0.42% | 0.15% | CL\_0000822\_down | Itgb1 |
| GO:0072503 | cellular divalent inorganic cation homeostasis | 2.95e-01 | 5.64e-01 | 1 | 22 | 238 | 15137 | 4.55% | 1.57% | 0.42% | 0.15% | CL\_0000822\_down | Itgb1 |
| GO:0040017 | positive regulation of locomotion | 2.95e-01 | 5.64e-01 | 1 | 22 | 238 | 15137 | 4.55% | 1.57% | 0.42% | 0.15% | CL\_0000822\_down | Itgb1 |
| GO:0032844 | regulation of homeostatic process | 2.98e-01 | 5.68e-01 | 1 | 22 | 241 | 15137 | 4.55% | 1.59% | 0.41% | 0.15% | CL\_0000822\_down | Hmgn3 |
| GO:0030155 | regulation of cell adhesion | 3.03e-01 | 5.75e-01 | 1 | 22 | 246 | 15137 | 4.55% | 1.63% | 0.41% | 0.15% | CL\_0000822\_down | Itgb1 |
| GO:0072507 | divalent inorganic cation homeostasis | 3.06e-01 | 5.75e-01 | 1 | 22 | 249 | 15137 | 4.55% | 1.64% | 0.40% | 0.15% | CL\_0000822\_down | Itgb1 |
| GO:0034660 | ncRNA metabolic process | 3.06e-01 | 5.75e-01 | 1 | 22 | 249 | 15137 | 4.55% | 1.64% | 0.40% | 0.15% | CL\_0000822\_down | Rrp1b |
| GO:0048583 | regulation of response to stimulus | 3.07e-01 | 5.75e-01 | 4 | 22 | 1933 | 15137 | 18.18% | 12.77% | 0.21% | 0.15% | CL\_0000822\_down | Arhgef12, Hmgn3, Itgb1, Sema7a |
| GO:0046578 | regulation of Ras protein signal transduction | 3.09e-01 | 5.75e-01 | 1 | 22 | 252 | 15137 | 4.55% | 1.66% | 0.40% | 0.15% | CL\_0000822\_down | Arhgef12 |
| GO:0048518 | positive regulation of biological process | 3.10e-01 | 5.75e-01 | 6 | 22 | 3183 | 15137 | 27.27% | 21.03% | 0.19% | 0.15% | CL\_0000822\_down | 2010001M09Rik, Hmgn3, Itgb1, Rps6ka1, Sema7a, Tfdp1 |
| GO:0052548 | regulation of endopeptidase activity | 3.10e-01 | 5.75e-01 | 1 | 22 | 253 | 15137 | 4.55% | 1.67% | 0.40% | 0.15% | CL\_0000822\_down | Rps6ka1 |
| GO:0016044 | cellular membrane organization | 3.13e-01 | 5.78e-01 | 1 | 22 | 256 | 15137 | 4.55% | 1.69% | 0.39% | 0.15% | CL\_0000822\_down | Itgb1 |
| GO:0061024 | membrane organization | 3.15e-01 | 5.80e-01 | 1 | 22 | 258 | 15137 | 4.55% | 1.70% | 0.39% | 0.15% | CL\_0000822\_down | Itgb1 |
| GO:0030036 | actin cytoskeleton organization | 3.22e-01 | 5.88e-01 | 1 | 22 | 265 | 15137 | 4.55% | 1.75% | 0.38% | 0.15% | CL\_0000822\_down | Itgb1 |
| GO:0001558 | regulation of cell growth | 3.22e-01 | 5.88e-01 | 1 | 22 | 265 | 15137 | 4.55% | 1.75% | 0.38% | 0.15% | CL\_0000822\_down | Sema7a |
| GO:0006875 | cellular metal ion homeostasis | 3.27e-01 | 5.91e-01 | 1 | 22 | 270 | 15137 | 4.55% | 1.78% | 0.37% | 0.15% | CL\_0000822\_down | Itgb1 |
| GO:0022604 | regulation of cell morphogenesis | 3.27e-01 | 5.91e-01 | 1 | 22 | 270 | 15137 | 4.55% | 1.78% | 0.37% | 0.15% | CL\_0000822\_down | Sema7a |
| GO:0031347 | regulation of defense response | 3.27e-01 | 5.91e-01 | 1 | 22 | 270 | 15137 | 4.55% | 1.78% | 0.37% | 0.15% | CL\_0000822\_down | Sema7a |
| GO:0051056 | regulation of small GTPase mediated signal transduction | 3.34e-01 | 5.96e-01 | 1 | 22 | 277 | 15137 | 4.55% | 1.83% | 0.36% | 0.15% | CL\_0000822\_down | Arhgef12 |
| GO:0019220 | regulation of phosphate metabolic process | 3.34e-01 | 5.96e-01 | 2 | 22 | 816 | 15137 | 9.09% | 5.39% | 0.25% | 0.15% | CL\_0000822\_down | Itgb1, Rrp1b |
| GO:0051174 | regulation of phosphorus metabolic process | 3.34e-01 | 5.96e-01 | 2 | 22 | 816 | 15137 | 9.09% | 5.39% | 0.25% | 0.15% | CL\_0000822\_down | Itgb1, Rrp1b |
| GO:0051129 | negative regulation of cellular component organization | 3.40e-01 | 6.01e-01 | 1 | 22 | 283 | 15137 | 4.55% | 1.87% | 0.35% | 0.15% | CL\_0000822\_down | Itgb1 |
| GO:0030029 | actin filament-based process | 3.41e-01 | 6.01e-01 | 1 | 22 | 284 | 15137 | 4.55% | 1.88% | 0.35% | 0.15% | CL\_0000822\_down | Itgb1 |
| GO:0055065 | metal ion homeostasis | 3.41e-01 | 6.01e-01 | 1 | 22 | 284 | 15137 | 4.55% | 1.88% | 0.35% | 0.15% | CL\_0000822\_down | Itgb1 |
| GO:0007049 | cell cycle | 3.42e-01 | 6.01e-01 | 2 | 22 | 830 | 15137 | 9.09% | 5.48% | 0.24% | 0.15% | CL\_0000822\_down | Itgb1, Tfdp1 |
| GO:0034097 | response to cytokine stimulus | 3.46e-01 | 6.05e-01 | 1 | 22 | 289 | 15137 | 4.55% | 1.91% | 0.35% | 0.15% | CL\_0000822\_down | Ccbp2 |
| GO:0051090 | regulation of sequence-specific DNA binding transcription factor activity | 3.47e-01 | 6.05e-01 | 1 | 22 | 290 | 15137 | 4.55% | 1.92% | 0.34% | 0.15% | CL\_0000822\_down | Hmgn3 |
| GO:0007243 | intracellular protein kinase cascade | 3.50e-01 | 6.08e-01 | 1 | 22 | 293 | 15137 | 4.55% | 1.94% | 0.34% | 0.15% | CL\_0000822\_down | Rps6ka1 |
| GO:0015672 | monovalent inorganic cation transport | 3.51e-01 | 6.08e-01 | 1 | 22 | 294 | 15137 | 4.55% | 1.94% | 0.34% | 0.15% | CL\_0000822\_down | Slc20a2 |
| GO:0030003 | cellular cation homeostasis | 3.59e-01 | 6.20e-01 | 1 | 22 | 303 | 15137 | 4.55% | 2.00% | 0.33% | 0.15% | CL\_0000822\_down | Itgb1 |
| GO:0048514 | blood vessel morphogenesis | 3.67e-01 | 6.30e-01 | 1 | 22 | 311 | 15137 | 4.55% | 2.05% | 0.32% | 0.15% | CL\_0000822\_down | Itgb1 |
| GO:0050865 | regulation of cell activation | 3.68e-01 | 6.30e-01 | 1 | 22 | 312 | 15137 | 4.55% | 2.06% | 0.32% | 0.15% | CL\_0000822\_down | Rps6ka1 |
| GO:0010556 | regulation of macromolecule biosynthetic process | 3.73e-01 | 6.35e-01 | 5 | 22 | 2760 | 15137 | 22.73% | 18.23% | 0.18% | 0.15% | CL\_0000822\_down | 2010001M09Rik, Hmgn3, Rps6ka1, Taf1d, Tfdp1 |
| GO:0009966 | regulation of signal transduction | 3.73e-01 | 6.35e-01 | 3 | 22 | 1498 | 15137 | 13.64% | 9.90% | 0.20% | 0.15% | CL\_0000822\_down | Arhgef12, Itgb1, Sema7a |
| GO:0061061 | muscle structure development | 3.78e-01 | 6.41e-01 | 1 | 22 | 323 | 15137 | 4.55% | 2.13% | 0.31% | 0.15% | CL\_0000822\_down | Itgb1 |
| GO:0052547 | regulation of peptidase activity | 3.83e-01 | 6.43e-01 | 1 | 22 | 328 | 15137 | 4.55% | 2.17% | 0.30% | 0.15% | CL\_0000822\_down | Rps6ka1 |
| GO:0044271 | cellular nitrogen compound biosynthetic process | 3.85e-01 | 6.43e-01 | 1 | 22 | 331 | 15137 | 4.55% | 2.19% | 0.30% | 0.15% | CL\_0000822\_down | Srm |
| GO:0009617 | response to bacterium | 3.85e-01 | 6.43e-01 | 1 | 22 | 331 | 15137 | 4.55% | 2.19% | 0.30% | 0.15% | CL\_0000822\_down | Ly96 |
| GO:0048699 | generation of neurons | 3.86e-01 | 6.43e-01 | 2 | 22 | 912 | 15137 | 9.09% | 6.02% | 0.22% | 0.15% | CL\_0000822\_down | Itgb1, Sema7a |
| GO:0050776 | regulation of immune response | 3.87e-01 | 6.43e-01 | 1 | 22 | 333 | 15137 | 4.55% | 2.20% | 0.30% | 0.15% | CL\_0000822\_down | Sema7a |
| GO:0048610 | cellular process involved in reproduction | 3.90e-01 | 6.43e-01 | 1 | 22 | 336 | 15137 | 4.55% | 2.22% | 0.30% | 0.15% | CL\_0000822\_down | Itgb1 |
| GO:0032101 | regulation of response to external stimulus | 3.90e-01 | 6.43e-01 | 1 | 22 | 336 | 15137 | 4.55% | 2.22% | 0.30% | 0.15% | CL\_0000822\_down | Sema7a |
| GO:0001817 | regulation of cytokine production | 3.91e-01 | 6.43e-01 | 1 | 22 | 337 | 15137 | 4.55% | 2.23% | 0.30% | 0.15% | CL\_0000822\_down | Sema7a |
| GO:0048584 | positive regulation of response to stimulus | 3.92e-01 | 6.43e-01 | 2 | 22 | 923 | 15137 | 9.09% | 6.10% | 0.22% | 0.15% | CL\_0000822\_down | Itgb1, Sema7a |
| GO:0001701 | in utero embryonic development | 3.99e-01 | 6.52e-01 | 1 | 22 | 346 | 15137 | 4.55% | 2.29% | 0.29% | 0.15% | CL\_0000822\_down | Itgb1 |
| GO:0035556 | intracellular signal transduction | 4.00e-01 | 6.52e-01 | 2 | 22 | 939 | 15137 | 9.09% | 6.20% | 0.21% | 0.15% | CL\_0000822\_down | Arhgef12, Rps6ka1 |
| GO:0007507 | heart development | 4.04e-01 | 6.57e-01 | 1 | 22 | 352 | 15137 | 4.55% | 2.33% | 0.28% | 0.15% | CL\_0000822\_down | Itgb1 |
| GO:0048522 | positive regulation of cellular process | 4.10e-01 | 6.61e-01 | 5 | 22 | 2878 | 15137 | 22.73% | 19.01% | 0.17% | 0.15% | CL\_0000822\_down | Hmgn3, Itgb1, Rps6ka1, Sema7a, Tfdp1 |
| GO:0071702 | organic substance transport | 4.13e-01 | 6.61e-01 | 1 | 22 | 362 | 15137 | 4.55% | 2.39% | 0.28% | 0.15% | CL\_0000822\_down | Slc36a4 |
| GO:0016568 | chromatin modification | 4.16e-01 | 6.61e-01 | 1 | 22 | 365 | 15137 | 4.55% | 2.41% | 0.27% | 0.15% | CL\_0000822\_down | Hmgn3 |
| GO:0006886 | intracellular protein transport | 4.16e-01 | 6.61e-01 | 1 | 22 | 365 | 15137 | 4.55% | 2.41% | 0.27% | 0.15% | CL\_0000822\_down | Itgb1 |
| GO:0009889 | regulation of biosynthetic process | 4.19e-01 | 6.61e-01 | 5 | 22 | 2905 | 15137 | 22.73% | 19.19% | 0.17% | 0.15% | CL\_0000822\_down | 2010001M09Rik, Hmgn3, Rps6ka1, Taf1d, Tfdp1 |
| GO:0022008 | neurogenesis | 4.19e-01 | 6.61e-01 | 2 | 22 | 976 | 15137 | 9.09% | 6.45% | 0.20% | 0.15% | CL\_0000822\_down | Itgb1, Sema7a |
| GO:0016070 | RNA metabolic process | 4.19e-01 | 6.61e-01 | 4 | 22 | 2255 | 15137 | 18.18% | 14.90% | 0.18% | 0.15% | CL\_0000822\_down | Rrp1b, Taf1d, Tfdp1, Tmem18 |
| GO:0006996 | organelle organization | 4.20e-01 | 6.61e-01 | 3 | 22 | 1610 | 15137 | 13.64% | 10.64% | 0.19% | 0.15% | CL\_0000822\_down | Hmgn3, Itgb1, Slc25a4 |
| GO:0016310 | phosphorylation | 4.21e-01 | 6.61e-01 | 2 | 22 | 980 | 15137 | 9.09% | 6.47% | 0.20% | 0.15% | CL\_0000822\_down | Rps6ka1, Uck2 |
| GO:0001568 | blood vessel development | 4.22e-01 | 6.61e-01 | 1 | 22 | 372 | 15137 | 4.55% | 2.46% | 0.27% | 0.15% | CL\_0000822\_down | Itgb1 |
| GO:0044106 | cellular amine metabolic process | 4.24e-01 | 6.61e-01 | 1 | 22 | 374 | 15137 | 4.55% | 2.47% | 0.27% | 0.15% | CL\_0000822\_down | Srm |
| GO:0030334 | regulation of cell migration | 4.25e-01 | 6.61e-01 | 1 | 22 | 376 | 15137 | 4.55% | 2.48% | 0.27% | 0.15% | CL\_0000822\_down | Itgb1 |
| GO:0007166 | cell surface receptor signaling pathway | 4.26e-01 | 6.61e-01 | 5 | 22 | 2927 | 15137 | 22.73% | 19.34% | 0.17% | 0.15% | CL\_0000822\_down | Arhgef12, Ccbp2, Itgb1, Ly96, Sema7a |
| GO:0045595 | regulation of cell differentiation | 4.27e-01 | 6.61e-01 | 2 | 22 | 990 | 15137 | 9.09% | 6.54% | 0.20% | 0.15% | CL\_0000822\_down | Itgb1, Sema7a |
| GO:0031175 | neuron projection development | 4.28e-01 | 6.61e-01 | 1 | 22 | 379 | 15137 | 4.55% | 2.50% | 0.26% | 0.15% | CL\_0000822\_down | Itgb1 |
| GO:0030097 | hemopoiesis | 4.32e-01 | 6.66e-01 | 1 | 22 | 384 | 15137 | 4.55% | 2.54% | 0.26% | 0.15% | CL\_0000822\_down | Sfxn1 |
| GO:2000145 | regulation of cell motility | 4.37e-01 | 6.68e-01 | 1 | 22 | 390 | 15137 | 4.55% | 2.58% | 0.26% | 0.15% | CL\_0000822\_down | Itgb1 |
| GO:0042592 | homeostatic process | 4.37e-01 | 6.68e-01 | 2 | 22 | 1011 | 15137 | 9.09% | 6.68% | 0.20% | 0.15% | CL\_0000822\_down | Itgb1, Sfxn1 |
| GO:0051049 | regulation of transport | 4.38e-01 | 6.68e-01 | 2 | 22 | 1012 | 15137 | 9.09% | 6.69% | 0.20% | 0.15% | CL\_0000822\_down | Hmgn3, Itgb1 |
| GO:0002684 | positive regulation of immune system process | 4.42e-01 | 6.72e-01 | 1 | 22 | 396 | 15137 | 4.55% | 2.62% | 0.25% | 0.15% | CL\_0000822\_down | Sema7a |
| GO:0002376 | immune system process | 4.43e-01 | 6.72e-01 | 2 | 22 | 1022 | 15137 | 9.09% | 6.75% | 0.20% | 0.15% | CL\_0000822\_down | Ly96, Sfxn1 |
| GO:0007276 | gamete generation | 4.45e-01 | 6.72e-01 | 1 | 22 | 399 | 15137 | 4.55% | 2.64% | 0.25% | 0.15% | CL\_0000822\_down | Itgb1 |
| GO:0009888 | tissue development | 4.50e-01 | 6.76e-01 | 2 | 22 | 1035 | 15137 | 9.09% | 6.84% | 0.19% | 0.15% | CL\_0000822\_down | Itgb1, Tfdp1 |
| GO:0001944 | vasculature development | 4.52e-01 | 6.76e-01 | 1 | 22 | 408 | 15137 | 4.55% | 2.70% | 0.25% | 0.15% | CL\_0000822\_down | Itgb1 |
| GO:0006351 | transcription, DNA-dependent | 4.54e-01 | 6.76e-01 | 3 | 22 | 1694 | 15137 | 13.64% | 11.19% | 0.18% | 0.15% | CL\_0000822\_down | Taf1d, Tfdp1, Tmem18 |
| GO:0032774 | RNA biosynthetic process | 4.54e-01 | 6.76e-01 | 3 | 22 | 1696 | 15137 | 13.64% | 11.20% | 0.18% | 0.15% | CL\_0000822\_down | Taf1d, Tfdp1, Tmem18 |
| GO:0051270 | regulation of cellular component movement | 4.56e-01 | 6.76e-01 | 1 | 22 | 413 | 15137 | 4.55% | 2.73% | 0.24% | 0.15% | CL\_0000822\_down | Itgb1 |
| GO:0060255 | regulation of macromolecule metabolic process | 4.61e-01 | 6.76e-01 | 6 | 22 | 3706 | 15137 | 27.27% | 24.48% | 0.16% | 0.15% | CL\_0000822\_down | 2010001M09Rik, Hmgn3, Itgb1, Rps6ka1, Taf1d, Tfdp1 |
| GO:0051046 | regulation of secretion | 4.62e-01 | 6.76e-01 | 1 | 22 | 420 | 15137 | 4.55% | 2.77% | 0.24% | 0.15% | CL\_0000822\_down | Hmgn3 |
| GO:0040012 | regulation of locomotion | 4.63e-01 | 6.76e-01 | 1 | 22 | 421 | 15137 | 4.55% | 2.78% | 0.24% | 0.15% | CL\_0000822\_down | Itgb1 |
| GO:0065009 | regulation of molecular function | 4.64e-01 | 6.76e-01 | 3 | 22 | 1720 | 15137 | 13.64% | 11.36% | 0.17% | 0.15% | CL\_0000822\_down | Hmgn3, Rps6ka1, Rrp1b |
| GO:0042981 | regulation of apoptotic process | 4.65e-01 | 6.76e-01 | 2 | 22 | 1065 | 15137 | 9.09% | 7.04% | 0.19% | 0.15% | CL\_0000822\_down | Itgb1, Rps6ka1 |
| GO:0006325 | chromatin organization | 4.65e-01 | 6.76e-01 | 1 | 22 | 424 | 15137 | 4.55% | 2.80% | 0.24% | 0.15% | CL\_0000822\_down | Hmgn3 |
| GO:0071840 | cellular component organization or biogenesis | 4.65e-01 | 6.76e-01 | 5 | 22 | 3053 | 15137 | 22.73% | 20.17% | 0.16% | 0.15% | CL\_0000822\_down | Hmgn3, Itgb1, Rrp1b, Sema7a, Slc25a4 |
| GO:0045596 | negative regulation of cell differentiation | 4.66e-01 | 6.76e-01 | 1 | 22 | 425 | 15137 | 4.55% | 2.81% | 0.24% | 0.15% | CL\_0000822\_down | Itgb1 |
| GO:0048534 | hemopoietic or lymphoid organ development | 4.69e-01 | 6.77e-01 | 1 | 22 | 429 | 15137 | 4.55% | 2.83% | 0.23% | 0.15% | CL\_0000822\_down | Sfxn1 |
| GO:0043067 | regulation of programmed cell death | 4.70e-01 | 6.77e-01 | 2 | 22 | 1075 | 15137 | 9.09% | 7.10% | 0.19% | 0.15% | CL\_0000822\_down | Itgb1, Rps6ka1 |
| GO:0019222 | regulation of metabolic process | 4.73e-01 | 6.81e-01 | 7 | 22 | 4423 | 15137 | 31.82% | 29.22% | 0.16% | 0.15% | CL\_0000822\_down | 2010001M09Rik, Hmgn3, Itgb1, Rps6ka1, Rrp1b, Taf1d, Tfdp1 |
| GO:0031323 | regulation of cellular metabolic process | 4.76e-01 | 6.83e-01 | 6 | 22 | 3760 | 15137 | 27.27% | 24.84% | 0.16% | 0.15% | CL\_0000822\_down | Hmgn3, Itgb1, Rps6ka1, Rrp1b, Taf1d, Tfdp1 |
| GO:0006955 | immune response | 4.78e-01 | 6.83e-01 | 1 | 22 | 441 | 15137 | 4.55% | 2.91% | 0.23% | 0.15% | CL\_0000822\_down | Ly96 |
| GO:2000026 | regulation of multicellular organismal development | 4.80e-01 | 6.83e-01 | 2 | 22 | 1095 | 15137 | 9.09% | 7.23% | 0.18% | 0.15% | CL\_0000822\_down | Itgb1, Sema7a |
| GO:0002520 | immune system development | 4.86e-01 | 6.88e-01 | 1 | 22 | 451 | 15137 | 4.55% | 2.98% | 0.22% | 0.15% | CL\_0000822\_down | Sfxn1 |
| GO:0008285 | negative regulation of cell proliferation | 4.86e-01 | 6.88e-01 | 1 | 22 | 451 | 15137 | 4.55% | 2.98% | 0.22% | 0.15% | CL\_0000822\_down | Itgb1 |
| GO:0001934 | positive regulation of protein phosphorylation | 4.89e-01 | 6.88e-01 | 1 | 22 | 454 | 15137 | 4.55% | 3.00% | 0.22% | 0.15% | CL\_0000822\_down | Itgb1 |
| GO:0019953 | sexual reproduction | 4.90e-01 | 6.88e-01 | 1 | 22 | 456 | 15137 | 4.55% | 3.01% | 0.22% | 0.15% | CL\_0000822\_down | Itgb1 |
| GO:0006355 | regulation of transcription, DNA-dependent | 4.95e-01 | 6.88e-01 | 4 | 22 | 2472 | 15137 | 18.18% | 16.33% | 0.16% | 0.15% | CL\_0000822\_down | Hmgn3, Rps6ka1, Taf1d, Tfdp1 |
| GO:2001141 | regulation of RNA biosynthetic process | 4.96e-01 | 6.88e-01 | 4 | 22 | 2476 | 15137 | 18.18% | 16.36% | 0.16% | 0.15% | CL\_0000822\_down | Hmgn3, Rps6ka1, Taf1d, Tfdp1 |
| GO:0009308 | amine metabolic process | 4.98e-01 | 6.88e-01 | 1 | 22 | 466 | 15137 | 4.55% | 3.08% | 0.21% | 0.15% | CL\_0000822\_down | Srm |
| GO:0051707 | response to other organism | 4.99e-01 | 6.88e-01 | 1 | 22 | 468 | 15137 | 4.55% | 3.09% | 0.21% | 0.15% | CL\_0000822\_down | Ly96 |
| GO:0010467 | gene expression | 4.99e-01 | 6.88e-01 | 4 | 22 | 2486 | 15137 | 18.18% | 16.42% | 0.16% | 0.15% | CL\_0000822\_down | Rrp1b, Taf1d, Tfdp1, Tmem18 |
| GO:0071841 | cellular component organization or biogenesis at cellular level | 5.01e-01 | 6.88e-01 | 4 | 22 | 2490 | 15137 | 18.18% | 16.45% | 0.16% | 0.15% | CL\_0000822\_down | Hmgn3, Itgb1, Rrp1b, Slc25a4 |
| GO:0042327 | positive regulation of phosphorylation | 5.02e-01 | 6.88e-01 | 1 | 22 | 472 | 15137 | 4.55% | 3.12% | 0.21% | 0.15% | CL\_0000822\_down | Itgb1 |
| GO:0006357 | regulation of transcription from RNA polymerase II promoter | 5.04e-01 | 6.88e-01 | 2 | 22 | 1146 | 15137 | 9.09% | 7.57% | 0.17% | 0.15% | CL\_0000822\_down | Hmgn3, Rps6ka1 |
| GO:0045937 | positive regulation of phosphate metabolic process | 5.07e-01 | 6.88e-01 | 1 | 22 | 479 | 15137 | 4.55% | 3.16% | 0.21% | 0.15% | CL\_0000822\_down | Itgb1 |
| GO:0010562 | positive regulation of phosphorus metabolic process | 5.07e-01 | 6.88e-01 | 1 | 22 | 479 | 15137 | 4.55% | 3.16% | 0.21% | 0.15% | CL\_0000822\_down | Itgb1 |
| GO:0051128 | regulation of cellular component organization | 5.09e-01 | 6.88e-01 | 2 | 22 | 1155 | 15137 | 9.09% | 7.63% | 0.17% | 0.15% | CL\_0000822\_down | Itgb1, Sema7a |
| GO:0006796 | phosphate-containing compound metabolic process | 5.12e-01 | 6.88e-01 | 2 | 22 | 1162 | 15137 | 9.09% | 7.68% | 0.17% | 0.15% | CL\_0000822\_down | Rps6ka1, Uck2 |
| GO:0006793 | phosphorus metabolic process | 5.13e-01 | 6.88e-01 | 2 | 22 | 1164 | 15137 | 9.09% | 7.69% | 0.17% | 0.15% | CL\_0000822\_down | Rps6ka1, Uck2 |
| GO:0051050 | positive regulation of transport | 5.13e-01 | 6.88e-01 | 1 | 22 | 487 | 15137 | 4.55% | 3.22% | 0.21% | 0.15% | CL\_0000822\_down | Itgb1 |
| GO:0048666 | neuron development | 5.14e-01 | 6.88e-01 | 1 | 22 | 488 | 15137 | 4.55% | 3.22% | 0.20% | 0.15% | CL\_0000822\_down | Itgb1 |
| GO:0043065 | positive regulation of apoptotic process | 5.14e-01 | 6.88e-01 | 1 | 22 | 488 | 15137 | 4.55% | 3.22% | 0.20% | 0.15% | CL\_0000822\_down | Itgb1 |
| GO:0051252 | regulation of RNA metabolic process | 5.15e-01 | 6.88e-01 | 4 | 22 | 2532 | 15137 | 18.18% | 16.73% | 0.16% | 0.15% | CL\_0000822\_down | Hmgn3, Rps6ka1, Taf1d, Tfdp1 |
| GO:0043068 | positive regulation of programmed cell death | 5.18e-01 | 6.88e-01 | 1 | 22 | 493 | 15137 | 4.55% | 3.26% | 0.20% | 0.15% | CL\_0000822\_down | Itgb1 |
| GO:0009607 | response to biotic stimulus | 5.18e-01 | 6.88e-01 | 1 | 22 | 493 | 15137 | 4.55% | 3.26% | 0.20% | 0.15% | CL\_0000822\_down | Ly96 |
| GO:0051240 | positive regulation of multicellular organismal process | 5.18e-01 | 6.88e-01 | 1 | 22 | 494 | 15137 | 4.55% | 3.26% | 0.20% | 0.15% | CL\_0000822\_down | Sema7a |
| GO:0040008 | regulation of growth | 5.22e-01 | 6.88e-01 | 1 | 22 | 499 | 15137 | 4.55% | 3.30% | 0.20% | 0.15% | CL\_0000822\_down | Sema7a |
| GO:0048523 | negative regulation of cellular process | 5.22e-01 | 6.88e-01 | 4 | 22 | 2554 | 15137 | 18.18% | 16.87% | 0.16% | 0.15% | CL\_0000822\_down | Arhgef12, Itgb1, Rps6ka1, Slc25a4 |
| GO:0006396 | RNA processing | 5.24e-01 | 6.88e-01 | 1 | 22 | 502 | 15137 | 4.55% | 3.32% | 0.20% | 0.15% | CL\_0000822\_down | Rrp1b |
| GO:0065008 | regulation of biological quality | 5.25e-01 | 6.88e-01 | 3 | 22 | 1876 | 15137 | 13.64% | 12.39% | 0.16% | 0.15% | CL\_0000822\_down | Itgb1, Sema7a, Sfxn1 |
| GO:0044085 | cellular component biogenesis | 5.25e-01 | 6.88e-01 | 2 | 22 | 1190 | 15137 | 9.09% | 7.86% | 0.17% | 0.15% | CL\_0000822\_down | Itgb1, Rrp1b |
| GO:0010942 | positive regulation of cell death | 5.28e-01 | 6.89e-01 | 1 | 22 | 508 | 15137 | 4.55% | 3.36% | 0.20% | 0.15% | CL\_0000822\_down | Itgb1 |
| GO:0006873 | cellular ion homeostasis | 5.30e-01 | 6.89e-01 | 1 | 22 | 511 | 15137 | 4.55% | 3.38% | 0.20% | 0.15% | CL\_0000822\_down | Itgb1 |
| GO:0032504 | multicellular organism reproduction | 5.32e-01 | 6.89e-01 | 1 | 22 | 513 | 15137 | 4.55% | 3.39% | 0.19% | 0.15% | CL\_0000822\_down | Itgb1 |
| GO:0048609 | multicellular organismal reproductive process | 5.32e-01 | 6.89e-01 | 1 | 22 | 513 | 15137 | 4.55% | 3.39% | 0.19% | 0.15% | CL\_0000822\_down | Itgb1 |
| GO:0009968 | negative regulation of signal transduction | 5.43e-01 | 7.02e-01 | 1 | 22 | 529 | 15137 | 4.55% | 3.49% | 0.19% | 0.15% | CL\_0000822\_down | Arhgef12 |
| GO:0051093 | negative regulation of developmental process | 5.46e-01 | 7.02e-01 | 1 | 22 | 533 | 15137 | 4.55% | 3.52% | 0.19% | 0.15% | CL\_0000822\_down | Itgb1 |
| GO:0055082 | cellular chemical homeostasis | 5.46e-01 | 7.02e-01 | 1 | 22 | 534 | 15137 | 4.55% | 3.53% | 0.19% | 0.15% | CL\_0000822\_down | Itgb1 |
| GO:0080134 | regulation of response to stress | 5.51e-01 | 7.06e-01 | 1 | 22 | 540 | 15137 | 4.55% | 3.57% | 0.19% | 0.15% | CL\_0000822\_down | Sema7a |
| GO:0031401 | positive regulation of protein modification process | 5.53e-01 | 7.06e-01 | 1 | 22 | 543 | 15137 | 4.55% | 3.59% | 0.18% | 0.15% | CL\_0000822\_down | Itgb1 |
| GO:0022603 | regulation of anatomical structure morphogenesis | 5.54e-01 | 7.06e-01 | 1 | 22 | 545 | 15137 | 4.55% | 3.60% | 0.18% | 0.15% | CL\_0000822\_down | Sema7a |
| GO:0007010 | cytoskeleton organization | 5.59e-01 | 7.06e-01 | 1 | 22 | 553 | 15137 | 4.55% | 3.65% | 0.18% | 0.15% | CL\_0000822\_down | Itgb1 |
| GO:0051276 | chromosome organization | 5.63e-01 | 7.06e-01 | 1 | 22 | 558 | 15137 | 4.55% | 3.69% | 0.18% | 0.15% | CL\_0000822\_down | Hmgn3 |
| GO:0043009 | chordate embryonic development | 5.65e-01 | 7.06e-01 | 1 | 22 | 561 | 15137 | 4.55% | 3.71% | 0.18% | 0.15% | CL\_0000822\_down | Itgb1 |
| GO:0051726 | regulation of cell cycle | 5.65e-01 | 7.06e-01 | 1 | 22 | 561 | 15137 | 4.55% | 3.71% | 0.18% | 0.15% | CL\_0000822\_down | Itgb1 |
| GO:0060341 | regulation of cellular localization | 5.67e-01 | 7.06e-01 | 1 | 22 | 565 | 15137 | 4.55% | 3.73% | 0.18% | 0.15% | CL\_0000822\_down | Hmgn3 |
| GO:0090304 | nucleic acid metabolic process | 5.67e-01 | 7.06e-01 | 4 | 22 | 2692 | 15137 | 18.18% | 17.78% | 0.15% | 0.15% | CL\_0000822\_down | Rrp1b, Taf1d, Tfdp1, Tmem18 |
| GO:2000112 | regulation of cellular macromolecule biosynthetic process | 5.68e-01 | 7.06e-01 | 4 | 22 | 2694 | 15137 | 18.18% | 17.80% | 0.15% | 0.15% | CL\_0000822\_down | Hmgn3, Rps6ka1, Taf1d, Tfdp1 |
| GO:0009792 | embryo development ending in birth or egg hatching | 5.69e-01 | 7.06e-01 | 1 | 22 | 567 | 15137 | 4.55% | 3.75% | 0.18% | 0.15% | CL\_0000822\_down | Itgb1 |
| GO:0007165 | signal transduction | 5.69e-01 | 7.06e-01 | 6 | 22 | 4085 | 15137 | 27.27% | 26.99% | 0.15% | 0.15% | CL\_0000822\_down | Arhgef12, Ccbp2, Itgb1, Ly96, Rps6ka1, Sema7a |
| GO:0023057 | negative regulation of signaling | 5.69e-01 | 7.06e-01 | 1 | 22 | 568 | 15137 | 4.55% | 3.75% | 0.18% | 0.15% | CL\_0000822\_down | Arhgef12 |
| GO:0010648 | negative regulation of cell communication | 5.70e-01 | 7.06e-01 | 1 | 22 | 569 | 15137 | 4.55% | 3.76% | 0.18% | 0.15% | CL\_0000822\_down | Arhgef12 |
| GO:0048513 | organ development | 5.75e-01 | 7.09e-01 | 3 | 22 | 2013 | 15137 | 13.64% | 13.30% | 0.15% | 0.15% | CL\_0000822\_down | Itgb1, Sfxn1, Tfdp1 |
| GO:0043066 | negative regulation of apoptotic process | 5.76e-01 | 7.09e-01 | 1 | 22 | 579 | 15137 | 4.55% | 3.83% | 0.17% | 0.15% | CL\_0000822\_down | Rps6ka1 |
| GO:0030030 | cell projection organization | 5.78e-01 | 7.09e-01 | 1 | 22 | 581 | 15137 | 4.55% | 3.84% | 0.17% | 0.15% | CL\_0000822\_down | Itgb1 |
| GO:0043069 | negative regulation of programmed cell death | 5.79e-01 | 7.09e-01 | 1 | 22 | 584 | 15137 | 4.55% | 3.86% | 0.17% | 0.15% | CL\_0000822\_down | Rps6ka1 |
| GO:0034613 | cellular protein localization | 5.80e-01 | 7.09e-01 | 1 | 22 | 585 | 15137 | 4.55% | 3.86% | 0.17% | 0.15% | CL\_0000822\_down | Itgb1 |
| GO:0070727 | cellular macromolecule localization | 5.82e-01 | 7.09e-01 | 1 | 22 | 588 | 15137 | 4.55% | 3.88% | 0.17% | 0.15% | CL\_0000822\_down | Itgb1 |
| GO:0032989 | cellular component morphogenesis | 5.84e-01 | 7.10e-01 | 1 | 22 | 591 | 15137 | 4.55% | 3.90% | 0.17% | 0.15% | CL\_0000822\_down | Itgb1 |
| GO:0048731 | system development | 5.91e-01 | 7.13e-01 | 4 | 22 | 2767 | 15137 | 18.18% | 18.28% | 0.14% | 0.15% | CL\_0000822\_down | Itgb1, Sema7a, Sfxn1, Tfdp1 |
| GO:0050790 | regulation of catalytic activity | 5.92e-01 | 7.13e-01 | 2 | 22 | 1339 | 15137 | 9.09% | 8.85% | 0.15% | 0.15% | CL\_0000822\_down | Rps6ka1, Rrp1b |
| GO:0032879 | regulation of localization | 5.94e-01 | 7.13e-01 | 2 | 22 | 1344 | 15137 | 9.09% | 8.88% | 0.15% | 0.15% | CL\_0000822\_down | Hmgn3, Itgb1 |
| GO:0008284 | positive regulation of cell proliferation | 5.95e-01 | 7.13e-01 | 1 | 22 | 609 | 15137 | 4.55% | 4.02% | 0.16% | 0.15% | CL\_0000822\_down | Itgb1 |
| GO:0032270 | positive regulation of cellular protein metabolic process | 5.95e-01 | 7.13e-01 | 1 | 22 | 609 | 15137 | 4.55% | 4.02% | 0.16% | 0.15% | CL\_0000822\_down | Itgb1 |
| GO:0019725 | cellular homeostasis | 5.96e-01 | 7.13e-01 | 1 | 22 | 610 | 15137 | 4.55% | 4.03% | 0.16% | 0.15% | CL\_0000822\_down | Itgb1 |
| GO:0030182 | neuron differentiation | 5.99e-01 | 7.13e-01 | 1 | 22 | 615 | 15137 | 4.55% | 4.06% | 0.16% | 0.15% | CL\_0000822\_down | Itgb1 |
| GO:0010033 | response to organic substance | 6.00e-01 | 7.13e-01 | 2 | 22 | 1358 | 15137 | 9.09% | 8.97% | 0.15% | 0.15% | CL\_0000822\_down | Ccbp2, Ly96 |
| GO:0072358 | cardiovascular system development | 6.01e-01 | 7.13e-01 | 1 | 22 | 619 | 15137 | 4.55% | 4.09% | 0.16% | 0.15% | CL\_0000822\_down | Itgb1 |
| GO:0072359 | circulatory system development | 6.01e-01 | 7.13e-01 | 1 | 22 | 619 | 15137 | 4.55% | 4.09% | 0.16% | 0.15% | CL\_0000822\_down | Itgb1 |
| GO:0007399 | nervous system development | 6.05e-01 | 7.16e-01 | 2 | 22 | 1372 | 15137 | 9.09% | 9.06% | 0.15% | 0.15% | CL\_0000822\_down | Itgb1, Sema7a |
| GO:0046907 | intracellular transport | 6.10e-01 | 7.20e-01 | 1 | 22 | 634 | 15137 | 4.55% | 4.19% | 0.16% | 0.15% | CL\_0000822\_down | Itgb1 |
| GO:0048519 | negative regulation of biological process | 6.13e-01 | 7.21e-01 | 4 | 22 | 2839 | 15137 | 18.18% | 18.76% | 0.14% | 0.15% | CL\_0000822\_down | Arhgef12, Itgb1, Rps6ka1, Slc25a4 |
| GO:0050793 | regulation of developmental process | 6.14e-01 | 7.21e-01 | 2 | 22 | 1393 | 15137 | 9.09% | 9.20% | 0.14% | 0.15% | CL\_0000822\_down | Itgb1, Sema7a |
| GO:0034641 | cellular nitrogen compound metabolic process | 6.16e-01 | 7.21e-01 | 5 | 22 | 3557 | 15137 | 22.73% | 23.50% | 0.14% | 0.15% | CL\_0000822\_down | Rrp1b, Srm, Taf1d, Tfdp1, Tmem18 |
| GO:0031326 | regulation of cellular biosynthetic process | 6.23e-01 | 7.28e-01 | 4 | 22 | 2872 | 15137 | 18.18% | 18.97% | 0.14% | 0.15% | CL\_0000822\_down | Hmgn3, Rps6ka1, Taf1d, Tfdp1 |
| GO:0048646 | anatomical structure formation involved in morphogenesis | 6.28e-01 | 7.31e-01 | 1 | 22 | 665 | 15137 | 4.55% | 4.39% | 0.15% | 0.15% | CL\_0000822\_down | Itgb1 |
| GO:0001932 | regulation of protein phosphorylation | 6.31e-01 | 7.31e-01 | 1 | 22 | 671 | 15137 | 4.55% | 4.43% | 0.15% | 0.15% | CL\_0000822\_down | Itgb1 |
| GO:0019219 | regulation of nucleobase-containing compound metabolic process | 6.32e-01 | 7.31e-01 | 4 | 22 | 2902 | 15137 | 18.18% | 19.17% | 0.14% | 0.15% | CL\_0000822\_down | Hmgn3, Rps6ka1, Taf1d, Tfdp1 |
| GO:0071844 | cellular component assembly at cellular level | 6.35e-01 | 7.31e-01 | 1 | 22 | 678 | 15137 | 4.55% | 4.48% | 0.15% | 0.15% | CL\_0000822\_down | Itgb1 |
| GO:0048585 | negative regulation of response to stimulus | 6.35e-01 | 7.31e-01 | 1 | 22 | 678 | 15137 | 4.55% | 4.48% | 0.15% | 0.15% | CL\_0000822\_down | Arhgef12 |
| GO:0016043 | cellular component organization | 6.36e-01 | 7.31e-01 | 4 | 22 | 2914 | 15137 | 18.18% | 19.25% | 0.14% | 0.15% | CL\_0000822\_down | Hmgn3, Itgb1, Sema7a, Slc25a4 |
| GO:0051247 | positive regulation of protein metabolic process | 6.37e-01 | 7.31e-01 | 1 | 22 | 681 | 15137 | 4.55% | 4.50% | 0.15% | 0.15% | CL\_0000822\_down | Itgb1 |
| GO:0051171 | regulation of nitrogen compound metabolic process | 6.42e-01 | 7.35e-01 | 4 | 22 | 2935 | 15137 | 18.18% | 19.39% | 0.14% | 0.15% | CL\_0000822\_down | Hmgn3, Rps6ka1, Taf1d, Tfdp1 |
| GO:0006807 | nitrogen compound metabolic process | 6.45e-01 | 7.36e-01 | 5 | 22 | 3665 | 15137 | 22.73% | 24.21% | 0.14% | 0.15% | CL\_0000822\_down | Rrp1b, Srm, Taf1d, Tfdp1, Tmem18 |
| GO:0065007 | biological regulation | 6.46e-01 | 7.36e-01 | 13 | 22 | 9171 | 15137 | 59.09% | 60.59% | 0.14% | 0.15% | CL\_0000822\_down | 2010001M09Rik, Arhgef12, Ccbp2, Hmgn3, Itgb1, Ly96, Rps6ka1, Rrp1b, Sema7a, Sfxn1, Slc25a4, Taf1d, Tfdp1 |
| GO:0023052 | signaling | 6.49e-01 | 7.38e-01 | 6 | 22 | 4387 | 15137 | 27.27% | 28.98% | 0.14% | 0.15% | CL\_0000822\_down | Arhgef12, Ccbp2, Itgb1, Ly96, Rps6ka1, Sema7a |
| GO:0044249 | cellular biosynthetic process | 6.55e-01 | 7.42e-01 | 4 | 22 | 2982 | 15137 | 18.18% | 19.70% | 0.13% | 0.15% | CL\_0000822\_down | Srm, Taf1d, Tfdp1, Tmem18 |
| GO:0030154 | cell differentiation | 6.56e-01 | 7.42e-01 | 3 | 22 | 2252 | 15137 | 13.64% | 14.88% | 0.13% | 0.15% | CL\_0000822\_down | Itgb1, Sema7a, Sfxn1 |
| GO:0010468 | regulation of gene expression | 6.57e-01 | 7.42e-01 | 4 | 22 | 2988 | 15137 | 18.18% | 19.74% | 0.13% | 0.15% | CL\_0000822\_down | Hmgn3, Rps6ka1, Taf1d, Tfdp1 |
| GO:0080090 | regulation of primary metabolic process | 6.61e-01 | 7.44e-01 | 5 | 22 | 3724 | 15137 | 22.73% | 24.60% | 0.13% | 0.15% | CL\_0000822\_down | Hmgn3, Itgb1, Rps6ka1, Taf1d, Tfdp1 |
| GO:0042325 | regulation of phosphorylation | 6.65e-01 | 7.47e-01 | 1 | 22 | 733 | 15137 | 4.55% | 4.84% | 0.14% | 0.15% | CL\_0000822\_down | Itgb1 |
| GO:0006468 | protein phosphorylation | 6.68e-01 | 7.49e-01 | 1 | 22 | 740 | 15137 | 4.55% | 4.89% | 0.14% | 0.15% | CL\_0000822\_down | Rps6ka1 |
| GO:0034645 | cellular macromolecule biosynthetic process | 6.77e-01 | 7.56e-01 | 3 | 22 | 2320 | 15137 | 13.64% | 15.33% | 0.13% | 0.15% | CL\_0000822\_down | Taf1d, Tfdp1, Tmem18 |
| GO:0007154 | cell communication | 6.78e-01 | 7.56e-01 | 6 | 22 | 4503 | 15137 | 27.27% | 29.75% | 0.13% | 0.15% | CL\_0000822\_down | Arhgef12, Ccbp2, Itgb1, Ly96, Rps6ka1, Sema7a |
| GO:0071310 | cellular response to organic substance | 6.79e-01 | 7.56e-01 | 1 | 22 | 762 | 15137 | 4.55% | 5.03% | 0.13% | 0.15% | CL\_0000822\_down | Ccbp2 |
| GO:0009058 | biosynthetic process | 6.83e-01 | 7.57e-01 | 4 | 22 | 3082 | 15137 | 18.18% | 20.36% | 0.13% | 0.15% | CL\_0000822\_down | Srm, Taf1d, Tfdp1, Tmem18 |
| GO:0048869 | cellular developmental process | 6.85e-01 | 7.57e-01 | 3 | 22 | 2345 | 15137 | 13.64% | 15.49% | 0.13% | 0.15% | CL\_0000822\_down | Itgb1, Sema7a, Sfxn1 |
| GO:0009059 | macromolecule biosynthetic process | 6.85e-01 | 7.57e-01 | 3 | 22 | 2346 | 15137 | 13.64% | 15.50% | 0.13% | 0.15% | CL\_0000822\_down | Taf1d, Tfdp1, Tmem18 |
| GO:0015031 | protein transport | 6.90e-01 | 7.60e-01 | 1 | 22 | 785 | 15137 | 4.55% | 5.19% | 0.13% | 0.15% | CL\_0000822\_down | Itgb1 |
| GO:0071842 | cellular component organization at cellular level | 6.91e-01 | 7.60e-01 | 3 | 22 | 2366 | 15137 | 13.64% | 15.63% | 0.13% | 0.15% | CL\_0000822\_down | Hmgn3, Itgb1, Slc25a4 |
| GO:0031399 | regulation of protein modification process | 7.02e-01 | 7.69e-01 | 1 | 22 | 809 | 15137 | 4.55% | 5.34% | 0.12% | 0.15% | CL\_0000822\_down | Itgb1 |
| GO:0045184 | establishment of protein localization | 7.02e-01 | 7.69e-01 | 1 | 22 | 810 | 15137 | 4.55% | 5.35% | 0.12% | 0.15% | CL\_0000822\_down | Itgb1 |
| GO:0050789 | regulation of biological process | 7.15e-01 | 7.79e-01 | 12 | 22 | 8805 | 15137 | 54.55% | 58.17% | 0.14% | 0.15% | CL\_0000822\_down | 2010001M09Rik, Arhgef12, Ccbp2, Hmgn3, Itgb1, Ly96, Rps6ka1, Rrp1b, Sema7a, Slc25a4, Taf1d, Tfdp1 |
| GO:0022414 | reproductive process | 7.15e-01 | 7.79e-01 | 1 | 22 | 840 | 15137 | 4.55% | 5.55% | 0.12% | 0.15% | CL\_0000822\_down | Itgb1 |
| GO:0000003 | reproduction | 7.17e-01 | 7.79e-01 | 1 | 22 | 843 | 15137 | 4.55% | 5.57% | 0.12% | 0.15% | CL\_0000822\_down | Itgb1 |
| GO:0006139 | nucleobase-containing compound metabolic process | 7.19e-01 | 7.80e-01 | 4 | 22 | 3219 | 15137 | 18.18% | 21.27% | 0.12% | 0.15% | CL\_0000822\_down | Rrp1b, Taf1d, Tfdp1, Tmem18 |
| GO:0044093 | positive regulation of molecular function | 7.20e-01 | 7.80e-01 | 1 | 22 | 851 | 15137 | 4.55% | 5.62% | 0.12% | 0.15% | CL\_0000822\_down | Hmgn3 |
| GO:0048856 | anatomical structure development | 7.22e-01 | 7.80e-01 | 4 | 22 | 3232 | 15137 | 18.18% | 21.35% | 0.12% | 0.15% | CL\_0000822\_down | Itgb1, Sema7a, Sfxn1, Tfdp1 |
| GO:0055114 | oxidation-reduction process | 7.28e-01 | 7.85e-01 | 1 | 22 | 870 | 15137 | 4.55% | 5.75% | 0.11% | 0.15% | CL\_0000822\_down | Akr1e1 |
| GO:0050794 | regulation of cellular process | 7.34e-01 | 7.89e-01 | 11 | 22 | 8223 | 15137 | 50.00% | 54.32% | 0.13% | 0.15% | CL\_0000822\_down | Arhgef12, Ccbp2, Hmgn3, Itgb1, Ly96, Rps6ka1, Rrp1b, Sema7a, Slc25a4, Taf1d, Tfdp1 |
| GO:0051239 | regulation of multicellular organismal process | 7.42e-01 | 7.94e-01 | 2 | 22 | 1754 | 15137 | 9.09% | 11.59% | 0.11% | 0.15% | CL\_0000822\_down | Itgb1, Sema7a |
| GO:0051649 | establishment of localization in cell | 7.42e-01 | 7.94e-01 | 1 | 22 | 903 | 15137 | 4.55% | 5.97% | 0.11% | 0.15% | CL\_0000822\_down | Itgb1 |
| GO:0009790 | embryo development | 7.43e-01 | 7.94e-01 | 1 | 22 | 907 | 15137 | 4.55% | 5.99% | 0.11% | 0.15% | CL\_0000822\_down | Itgb1 |
| GO:0007275 | multicellular organismal development | 7.45e-01 | 7.94e-01 | 4 | 22 | 3327 | 15137 | 18.18% | 21.98% | 0.12% | 0.15% | CL\_0000822\_down | Itgb1, Sema7a, Sfxn1, Tfdp1 |
| GO:0006950 | response to stress | 7.48e-01 | 7.95e-01 | 2 | 22 | 1775 | 15137 | 9.09% | 11.73% | 0.11% | 0.15% | CL\_0000822\_down | Ly96, Sema7a |
| GO:0070887 | cellular response to chemical stimulus | 7.76e-01 | 8.23e-01 | 1 | 22 | 994 | 15137 | 4.55% | 6.57% | 0.10% | 0.15% | CL\_0000822\_down | Ccbp2 |
| GO:0051716 | cellular response to stimulus | 7.85e-01 | 8.29e-01 | 6 | 22 | 4999 | 15137 | 27.27% | 33.03% | 0.12% | 0.15% | CL\_0000822\_down | Arhgef12, Ccbp2, Itgb1, Ly96, Rps6ka1, Sema7a |
| GO:0032268 | regulation of cellular protein metabolic process | 7.85e-01 | 8.29e-01 | 1 | 22 | 1022 | 15137 | 4.55% | 6.75% | 0.10% | 0.15% | CL\_0000822\_down | Itgb1 |
| GO:0048468 | cell development | 7.93e-01 | 8.33e-01 | 1 | 22 | 1046 | 15137 | 4.55% | 6.91% | 0.10% | 0.15% | CL\_0000822\_down | Itgb1 |
| GO:0022607 | cellular component assembly | 7.94e-01 | 8.33e-01 | 1 | 22 | 1049 | 15137 | 4.55% | 6.93% | 0.10% | 0.15% | CL\_0000822\_down | Itgb1 |
| GO:0042127 | regulation of cell proliferation | 7.95e-01 | 8.33e-01 | 1 | 22 | 1050 | 15137 | 4.55% | 6.94% | 0.10% | 0.15% | CL\_0000822\_down | Itgb1 |
| GO:0007186 | G-protein coupled receptor signaling pathway | 8.01e-01 | 8.38e-01 | 2 | 22 | 1974 | 15137 | 9.09% | 13.04% | 0.10% | 0.15% | CL\_0000822\_down | Arhgef12, Ccbp2 |
| GO:0008104 | protein localization | 8.03e-01 | 8.38e-01 | 1 | 22 | 1076 | 15137 | 4.55% | 7.11% | 0.09% | 0.15% | CL\_0000822\_down | Itgb1 |
| GO:0051641 | cellular localization | 8.17e-01 | 8.52e-01 | 1 | 22 | 1125 | 15137 | 4.55% | 7.43% | 0.09% | 0.15% | CL\_0000822\_down | Itgb1 |
| GO:0032502 | developmental process | 8.19e-01 | 8.52e-01 | 4 | 22 | 3680 | 15137 | 18.18% | 24.31% | 0.11% | 0.15% | CL\_0000822\_down | Itgb1, Sema7a, Sfxn1, Tfdp1 |
| GO:0042221 | response to chemical stimulus | 8.30e-01 | 8.61e-01 | 2 | 22 | 2101 | 15137 | 9.09% | 13.88% | 0.10% | 0.15% | CL\_0000822\_down | Ccbp2, Ly96 |
| GO:0051246 | regulation of protein metabolic process | 8.39e-01 | 8.68e-01 | 1 | 22 | 1205 | 15137 | 4.55% | 7.96% | 0.08% | 0.15% | CL\_0000822\_down | Itgb1 |
| GO:0033036 | macromolecule localization | 8.51e-01 | 8.79e-01 | 1 | 22 | 1253 | 15137 | 4.55% | 8.28% | 0.08% | 0.15% | CL\_0000822\_down | Itgb1 |
| GO:0044260 | cellular macromolecule metabolic process | 8.54e-01 | 8.80e-01 | 5 | 22 | 4660 | 15137 | 22.73% | 30.79% | 0.11% | 0.15% | CL\_0000822\_down | Rps6ka1, Rrp1b, Taf1d, Tfdp1, Tmem18 |
| GO:0044237 | cellular metabolic process | 8.88e-01 | 9.13e-01 | 7 | 22 | 6399 | 15137 | 31.82% | 42.27% | 0.11% | 0.15% | CL\_0000822\_down | Rps6ka1, Rrp1b, Srm, Taf1d, Tfdp1, Tmem18, Uck2 |
| GO:0009653 | anatomical structure morphogenesis | 9.13e-01 | 9.36e-01 | 1 | 22 | 1586 | 15137 | 4.55% | 10.48% | 0.06% | 0.15% | CL\_0000822\_down | Itgb1 |
| GO:0009987 | cellular process | 9.21e-01 | 9.41e-01 | 16 | 22 | 12456 | 15137 | 72.73% | 82.29% | 0.13% | 0.15% | CL\_0000822\_down | Arhgef12, Ccbp2, Emb, Hmgn3, Itgb1, Ly96, Rps6ka1, Rrp1b, Sema7a, Sfxn1, Slc25a4, Srm, Taf1d, Tfdp1, Tmem18, Uck2 |
| GO:0043170 | macromolecule metabolic process | 9.21e-01 | 9.41e-01 | 5 | 22 | 5219 | 15137 | 22.73% | 34.48% | 0.10% | 0.15% | CL\_0000822\_down | Rps6ka1, Rrp1b, Taf1d, Tfdp1, Tmem18 |
| GO:0008152 | metabolic process | 9.31e-01 | 9.45e-01 | 8 | 22 | 7543 | 15137 | 36.36% | 49.83% | 0.11% | 0.15% | CL\_0000822\_down | Akr1e1, Rps6ka1, Rrp1b, Srm, Taf1d, Tfdp1, Tmem18, Uck2 |
| GO:0006464 | cellular protein modification process | 9.31e-01 | 9.45e-01 | 1 | 22 | 1735 | 15137 | 4.55% | 11.46% | 0.06% | 0.15% | CL\_0000822\_down | Rps6ka1 |
| GO:0036211 | protein modification process | 9.31e-01 | 9.45e-01 | 1 | 22 | 1735 | 15137 | 4.55% | 11.46% | 0.06% | 0.15% | CL\_0000822\_down | Rps6ka1 |
| GO:0043412 | macromolecule modification | 9.40e-01 | 9.52e-01 | 1 | 22 | 1817 | 15137 | 4.55% | 12.00% | 0.06% | 0.15% | CL\_0000822\_down | Rps6ka1 |
| GO:0050896 | response to stimulus | 9.50e-01 | 9.60e-01 | 6 | 22 | 6348 | 15137 | 27.27% | 41.94% | 0.09% | 0.15% | CL\_0000822\_down | Arhgef12, Ccbp2, Itgb1, Ly96, Rps6ka1, Sema7a |
| GO:0044267 | cellular protein metabolic process | 9.72e-01 | 9.80e-01 | 1 | 22 | 2265 | 15137 | 4.55% | 14.96% | 0.04% | 0.15% | CL\_0000822\_down | Rps6ka1 |
| GO:0032501 | multicellular organismal process | 9.79e-01 | 9.85e-01 | 4 | 22 | 5391 | 15137 | 18.18% | 35.61% | 0.07% | 0.15% | CL\_0000822\_down | Itgb1, Sema7a, Sfxn1, Tfdp1 |
| GO:0044238 | primary metabolic process | 9.87e-01 | 9.91e-01 | 5 | 22 | 6542 | 15137 | 22.73% | 43.22% | 0.08% | 0.15% | CL\_0000822\_down | Rps6ka1, Rrp1b, Taf1d, Tfdp1, Tmem18 |
| GO:0019538 | protein metabolic process | 9.89e-01 | 9.91e-01 | 1 | 22 | 2789 | 15137 | 4.55% | 18.43% | 0.04% | 0.15% | CL\_0000822\_down | Rps6ka1 |
| GO:0008150 | biological\_process | 1.00e+00 | 1.00e+00 | 22 | 22 | 15137 | 15137 | 100.00% | 100.00% | 0.15% | 0.15% | CL\_0000822\_down | 2010001M09Rik, Akr1e1, Arhgef12, Ccbp2, Emb, Hmgn3, Itgb1, Kcnk5, Ly96, Rps6ka1, Rrp1b, Sema7a, Sfxn1, Slc20a2, Slc25a4, Slc35f2, Slc36a4, Srm, Taf1d, Tfdp1, Tmem18, Uck2 |

### cellular\_component (top)

  
  


| TermID | Term | P | Q | k | n | K | N | k/n | K/N | k/K | n/N | Qset | Symbols |
| --- | --- | --- | --- | --- | --- | --- | --- | --- | --- | --- | --- | --- | --- |
| GO:0034667 | alpha3-beta1 integrin complex | 1.34e-03 | 1.10e-01 | 1 | 22 | 1 | 16439 | 4.55% | 0.01% | 100.00% | 0.13% | CL\_0000822\_down | Itgb1 |
| GO:0030688 | preribosome, small subunit precursor | 2.67e-03 | 1.10e-01 | 1 | 22 | 2 | 16439 | 4.55% | 0.01% | 50.00% | 0.13% | CL\_0000822\_down | Rrp1b |
| GO:0034679 | alpha9-beta1 integrin complex | 4.01e-03 | 1.10e-01 | 1 | 22 | 3 | 16439 | 4.55% | 0.02% | 33.33% | 0.13% | CL\_0000822\_down | Itgb1 |
| GO:0034663 | endoplasmic reticulum chaperone complex | 4.01e-03 | 1.10e-01 | 1 | 22 | 3 | 16439 | 4.55% | 0.02% | 33.33% | 0.13% | CL\_0000822\_down | 2010001M09Rik |
| GO:0046696 | lipopolysaccharide receptor complex | 5.34e-03 | 1.18e-01 | 1 | 22 | 4 | 16439 | 4.55% | 0.02% | 25.00% | 0.13% | CL\_0000822\_down | Ly96 |
| GO:0035748 | myelin sheath abaxonal region | 9.33e-03 | 1.18e-01 | 1 | 22 | 7 | 16439 | 4.55% | 0.04% | 14.29% | 0.13% | CL\_0000822\_down | Itgb1 |
| GO:0005741 | mitochondrial outer membrane | 9.56e-03 | 1.18e-01 | 2 | 22 | 111 | 16439 | 9.09% | 0.68% | 1.80% | 0.13% | CL\_0000822\_down | Bri3bp, Slc25a4 |
| GO:0030056 | hemidesmosome | 1.07e-02 | 1.18e-01 | 1 | 22 | 8 | 16439 | 4.55% | 0.05% | 12.50% | 0.13% | CL\_0000822\_down | Itgb1 |
| GO:0031967 | organelle envelope | 1.14e-02 | 1.18e-01 | 4 | 22 | 674 | 16439 | 18.18% | 4.10% | 0.59% | 0.13% | CL\_0000822\_down | Bri3bp, Sfxn1, Slc25a4, Tmem18 |
| GO:0031975 | envelope | 1.20e-02 | 1.18e-01 | 4 | 22 | 685 | 16439 | 18.18% | 4.17% | 0.58% | 0.13% | CL\_0000822\_down | Bri3bp, Sfxn1, Slc25a4, Tmem18 |
| GO:0031968 | organelle outer membrane | 1.22e-02 | 1.18e-01 | 2 | 22 | 126 | 16439 | 9.09% | 0.77% | 1.59% | 0.13% | CL\_0000822\_down | Bri3bp, Slc25a4 |
| GO:0019867 | outer membrane | 1.29e-02 | 1.18e-01 | 2 | 22 | 130 | 16439 | 9.09% | 0.79% | 1.54% | 0.13% | CL\_0000822\_down | Bri3bp, Slc25a4 |
| GO:0043235 | receptor complex | 1.72e-02 | 1.45e-01 | 2 | 22 | 151 | 16439 | 9.09% | 0.92% | 1.32% | 0.13% | CL\_0000822\_down | Itgb1, Ly96 |
| GO:0030684 | preribosome | 1.99e-02 | 1.45e-01 | 1 | 22 | 15 | 16439 | 4.55% | 0.09% | 6.67% | 0.13% | CL\_0000822\_down | Rrp1b |
| GO:0031224 | intrinsic to membrane | 1.99e-02 | 1.45e-01 | 13 | 22 | 5824 | 16439 | 59.09% | 35.43% | 0.22% | 0.13% | CL\_0000822\_down | Bri3bp, Ccbp2, Emb, Itgb1, Kcnk5, Ly96, Sema7a, Sfxn1, Slc20a2, Slc25a4, Slc35f2, Slc36a4, Tmem18 |
| GO:0031966 | mitochondrial membrane | 2.11e-02 | 1.45e-01 | 3 | 22 | 448 | 16439 | 13.64% | 2.73% | 0.67% | 0.13% | CL\_0000822\_down | Bri3bp, Sfxn1, Slc25a4 |
| GO:0005740 | mitochondrial envelope | 2.40e-02 | 1.55e-01 | 3 | 22 | 471 | 16439 | 13.64% | 2.87% | 0.64% | 0.13% | CL\_0000822\_down | Bri3bp, Sfxn1, Slc25a4 |
| GO:0008305 | integrin complex | 3.55e-02 | 2.06e-01 | 1 | 22 | 27 | 16439 | 4.55% | 0.16% | 3.70% | 0.13% | CL\_0000822\_down | Itgb1 |
| GO:0014704 | intercalated disc | 3.55e-02 | 2.06e-01 | 1 | 22 | 27 | 16439 | 4.55% | 0.16% | 3.70% | 0.13% | CL\_0000822\_down | Itgb1 |
| GO:0044291 | cell-cell contact zone | 3.81e-02 | 2.10e-01 | 1 | 22 | 29 | 16439 | 4.55% | 0.18% | 3.45% | 0.13% | CL\_0000822\_down | Itgb1 |
| GO:0044429 | mitochondrial part | 4.00e-02 | 2.10e-01 | 3 | 22 | 575 | 16439 | 13.64% | 3.50% | 0.52% | 0.13% | CL\_0000822\_down | Bri3bp, Sfxn1, Slc25a4 |
| GO:0016021 | integral to membrane | 4.35e-02 | 2.10e-01 | 12 | 22 | 5690 | 16439 | 54.55% | 34.61% | 0.21% | 0.13% | CL\_0000822\_down | Bri3bp, Ccbp2, Emb, Itgb1, Kcnk5, Ly96, Sfxn1, Slc20a2, Slc25a4, Slc35f2, Slc36a4, Tmem18 |
| GO:0009897 | external side of plasma membrane | 4.45e-02 | 2.10e-01 | 2 | 22 | 253 | 16439 | 9.09% | 1.54% | 0.79% | 0.13% | CL\_0000822\_down | Itgb1, Sema7a |
| GO:0009925 | basal plasma membrane | 4.58e-02 | 2.10e-01 | 1 | 22 | 35 | 16439 | 4.55% | 0.21% | 2.86% | 0.13% | CL\_0000822\_down | Itgb1 |
| GO:0044425 | membrane part | 5.25e-02 | 2.23e-01 | 13 | 22 | 6537 | 16439 | 59.09% | 39.77% | 0.20% | 0.13% | CL\_0000822\_down | Bri3bp, Ccbp2, Emb, Itgb1, Kcnk5, Ly96, Sema7a, Sfxn1, Slc20a2, Slc25a4, Slc35f2, Slc36a4, Tmem18 |
| GO:0045178 | basal part of cell | 5.35e-02 | 2.23e-01 | 1 | 22 | 41 | 16439 | 4.55% | 0.25% | 2.44% | 0.13% | CL\_0000822\_down | Itgb1 |
| GO:0043209 | myelin sheath | 5.48e-02 | 2.23e-01 | 1 | 22 | 42 | 16439 | 4.55% | 0.26% | 2.38% | 0.13% | CL\_0000822\_down | Itgb1 |
| GO:0031594 | neuromuscular junction | 5.73e-02 | 2.25e-01 | 1 | 22 | 44 | 16439 | 4.55% | 0.27% | 2.27% | 0.13% | CL\_0000822\_down | Itgb1 |
| GO:0031090 | organelle membrane | 6.24e-02 | 2.37e-01 | 4 | 22 | 1143 | 16439 | 18.18% | 6.95% | 0.35% | 0.13% | CL\_0000822\_down | Bri3bp, Sfxn1, Slc25a4, Tmem18 |
| GO:0005743 | mitochondrial inner membrane | 7.36e-02 | 2.70e-01 | 2 | 22 | 336 | 16439 | 9.09% | 2.04% | 0.60% | 0.13% | CL\_0000822\_down | Sfxn1, Slc25a4 |
| GO:0044446 | intracellular organelle part | 7.95e-02 | 2.77e-01 | 9 | 22 | 4160 | 16439 | 40.91% | 25.31% | 0.22% | 0.13% | CL\_0000822\_down | 2010001M09Rik, Bri3bp, Hmgn3, Rps6ka1, Rrp1b, Sfxn1, Slc25a4, Tfdp1, Tmem18 |
| GO:0019866 | organelle inner membrane | 8.05e-02 | 2.77e-01 | 2 | 22 | 354 | 16439 | 9.09% | 2.15% | 0.56% | 0.13% | CL\_0000822\_down | Sfxn1, Slc25a4 |
| GO:0044422 | organelle part | 8.94e-02 | 2.90e-01 | 9 | 22 | 4252 | 16439 | 40.91% | 25.87% | 0.21% | 0.13% | CL\_0000822\_down | 2010001M09Rik, Bri3bp, Hmgn3, Rps6ka1, Rrp1b, Sfxn1, Slc25a4, Tfdp1, Tmem18 |
| GO:0001669 | acrosomal vesicle | 8.97e-02 | 2.90e-01 | 1 | 22 | 70 | 16439 | 4.55% | 0.43% | 1.43% | 0.13% | CL\_0000822\_down | Itgb1 |
| GO:0042383 | sarcolemma | 1.13e-01 | 3.52e-01 | 1 | 22 | 89 | 16439 | 4.55% | 0.54% | 1.12% | 0.13% | CL\_0000822\_down | Itgb1 |
| GO:0005604 | basement membrane | 1.17e-01 | 3.52e-01 | 1 | 22 | 93 | 16439 | 4.55% | 0.57% | 1.08% | 0.13% | CL\_0000822\_down | Itgb1 |
| GO:0005925 | focal adhesion | 1.19e-01 | 3.52e-01 | 1 | 22 | 94 | 16439 | 4.55% | 0.57% | 1.06% | 0.13% | CL\_0000822\_down | Itgb1 |
| GO:0005924 | cell-substrate adherens junction | 1.25e-01 | 3.52e-01 | 1 | 22 | 99 | 16439 | 4.55% | 0.60% | 1.01% | 0.13% | CL\_0000822\_down | Itgb1 |
| GO:0016020 | membrane | 1.28e-01 | 3.52e-01 | 14 | 22 | 8099 | 16439 | 63.64% | 49.27% | 0.17% | 0.13% | CL\_0000822\_down | Arhgef12, Bri3bp, Ccbp2, Emb, Itgb1, Kcnk5, Ly96, Sema7a, Sfxn1, Slc20a2, Slc25a4, Slc35f2, Slc36a4, Tmem18 |
| GO:0031965 | nuclear membrane | 1.28e-01 | 3.52e-01 | 1 | 22 | 102 | 16439 | 4.55% | 0.62% | 0.98% | 0.13% | CL\_0000822\_down | Tmem18 |
| GO:0030055 | cell-substrate junction | 1.32e-01 | 3.53e-01 | 1 | 22 | 105 | 16439 | 4.55% | 0.64% | 0.95% | 0.13% | CL\_0000822\_down | Itgb1 |
| GO:0009986 | cell surface | 1.65e-01 | 4.25e-01 | 2 | 22 | 547 | 16439 | 9.09% | 3.33% | 0.37% | 0.13% | CL\_0000822\_down | Itgb1, Sema7a |
| GO:0031225 | anchored to membrane | 1.66e-01 | 4.25e-01 | 1 | 22 | 135 | 16439 | 4.55% | 0.82% | 0.74% | 0.13% | CL\_0000822\_down | Sema7a |
| GO:0005819 | spindle | 1.70e-01 | 4.26e-01 | 1 | 22 | 139 | 16439 | 4.55% | 0.85% | 0.72% | 0.13% | CL\_0000822\_down | Rps6ka1 |
| GO:0030529 | ribonucleoprotein complex | 1.81e-01 | 4.36e-01 | 2 | 22 | 580 | 16439 | 9.09% | 3.53% | 0.34% | 0.13% | CL\_0000822\_down | Rps6ka1, Rrp1b |
| GO:0005912 | adherens junction | 1.84e-01 | 4.36e-01 | 1 | 22 | 151 | 16439 | 4.55% | 0.92% | 0.66% | 0.13% | CL\_0000822\_down | Itgb1 |
| GO:0005624 | membrane fraction | 1.86e-01 | 4.36e-01 | 2 | 22 | 591 | 16439 | 9.09% | 3.60% | 0.34% | 0.13% | CL\_0000822\_down | Itgb1, Uck2 |
| GO:0070161 | anchoring junction | 1.98e-01 | 4.54e-01 | 1 | 22 | 164 | 16439 | 4.55% | 1.00% | 0.61% | 0.13% | CL\_0000822\_down | Itgb1 |
| GO:0005626 | insoluble fraction | 2.07e-01 | 4.65e-01 | 2 | 22 | 633 | 16439 | 9.09% | 3.85% | 0.32% | 0.13% | CL\_0000822\_down | Itgb1, Uck2 |
| GO:0044420 | extracellular matrix part | 2.19e-01 | 4.83e-01 | 1 | 22 | 184 | 16439 | 4.55% | 1.12% | 0.54% | 0.13% | CL\_0000822\_down | Itgb1 |
| GO:0005635 | nuclear envelope | 2.47e-01 | 5.17e-01 | 1 | 22 | 210 | 16439 | 4.55% | 1.28% | 0.48% | 0.13% | CL\_0000822\_down | Tmem18 |
| GO:0005792 | microsome | 2.48e-01 | 5.17e-01 | 1 | 22 | 211 | 16439 | 4.55% | 1.28% | 0.47% | 0.13% | CL\_0000822\_down | Uck2 |
| GO:0045121 | membrane raft | 2.53e-01 | 5.17e-01 | 1 | 22 | 216 | 16439 | 4.55% | 1.31% | 0.46% | 0.13% | CL\_0000822\_down | Itgb1 |
| GO:0042598 | vesicular fraction | 2.54e-01 | 5.17e-01 | 1 | 22 | 217 | 16439 | 4.55% | 1.32% | 0.46% | 0.13% | CL\_0000822\_down | Uck2 |
| GO:0030141 | secretory granule | 2.59e-01 | 5.17e-01 | 1 | 22 | 222 | 16439 | 4.55% | 1.35% | 0.45% | 0.13% | CL\_0000822\_down | Itgb1 |
| GO:0005840 | ribosome | 2.76e-01 | 5.41e-01 | 1 | 22 | 239 | 16439 | 4.55% | 1.45% | 0.42% | 0.13% | CL\_0000822\_down | Rps6ka1 |
| GO:0016323 | basolateral plasma membrane | 2.87e-01 | 5.54e-01 | 1 | 22 | 251 | 16439 | 4.55% | 1.53% | 0.40% | 0.13% | CL\_0000822\_down | Itgb1 |
| GO:0000785 | chromatin | 3.04e-01 | 5.70e-01 | 1 | 22 | 268 | 16439 | 4.55% | 1.63% | 0.37% | 0.13% | CL\_0000822\_down | Hmgn3 |
| GO:0044432 | endoplasmic reticulum part | 3.09e-01 | 5.70e-01 | 1 | 22 | 274 | 16439 | 4.55% | 1.67% | 0.36% | 0.13% | CL\_0000822\_down | 2010001M09Rik |
| GO:0005911 | cell-cell junction | 3.11e-01 | 5.70e-01 | 1 | 22 | 276 | 16439 | 4.55% | 1.68% | 0.36% | 0.13% | CL\_0000822\_down | Itgb1 |
| GO:0000267 | cell fraction | 3.27e-01 | 5.90e-01 | 2 | 22 | 872 | 16439 | 9.09% | 5.30% | 0.23% | 0.13% | CL\_0000822\_down | Itgb1, Uck2 |
| GO:0032991 | macromolecular complex | 3.37e-01 | 5.98e-01 | 6 | 22 | 3562 | 16439 | 27.27% | 21.67% | 0.17% | 0.13% | CL\_0000822\_down | 2010001M09Rik, Itgb1, Ly96, Rps6ka1, Rrp1b, Tfdp1 |
| GO:0005739 | mitochondrion | 3.54e-01 | 6.02e-01 | 3 | 22 | 1575 | 16439 | 13.64% | 9.58% | 0.19% | 0.13% | CL\_0000822\_down | Bri3bp, Sfxn1, Slc25a4 |
| GO:0005667 | transcription factor complex | 3.57e-01 | 6.02e-01 | 1 | 22 | 326 | 16439 | 4.55% | 1.98% | 0.31% | 0.13% | CL\_0000822\_down | Tfdp1 |
| GO:0005829 | cytosol | 3.58e-01 | 6.02e-01 | 2 | 22 | 934 | 16439 | 9.09% | 5.68% | 0.21% | 0.13% | CL\_0000822\_down | Rrp1b, Uck2 |
| GO:0005578 | proteinaceous extracellular matrix | 3.61e-01 | 6.02e-01 | 1 | 22 | 331 | 16439 | 4.55% | 2.01% | 0.30% | 0.13% | CL\_0000822\_down | Itgb1 |
| GO:0031012 | extracellular matrix | 3.97e-01 | 6.51e-01 | 1 | 22 | 373 | 16439 | 4.55% | 2.27% | 0.27% | 0.13% | CL\_0000822\_down | Itgb1 |
| GO:0044428 | nuclear part | 4.26e-01 | 6.89e-01 | 3 | 22 | 1765 | 16439 | 13.64% | 10.74% | 0.17% | 0.13% | CL\_0000822\_down | Rrp1b, Tfdp1, Tmem18 |
| GO:0005737 | cytoplasm | 4.41e-01 | 7.03e-01 | 12 | 22 | 8331 | 16439 | 54.55% | 50.68% | 0.14% | 0.13% | CL\_0000822\_down | 2010001M09Rik, Akr1e1, Arhgef12, Bri3bp, Hmgn3, Itgb1, Rps6ka1, Rrp1b, Sfxn1, Slc25a4, Tmem18, Uck2 |
| GO:0044444 | cytoplasmic part | 4.60e-01 | 7.23e-01 | 8 | 22 | 5480 | 16439 | 36.36% | 33.34% | 0.15% | 0.13% | CL\_0000822\_down | 2010001M09Rik, Bri3bp, Itgb1, Rps6ka1, Rrp1b, Sfxn1, Slc25a4, Uck2 |
| GO:0005730 | nucleolus | 4.73e-01 | 7.32e-01 | 1 | 22 | 471 | 16439 | 4.55% | 2.87% | 0.21% | 0.13% | CL\_0000822\_down | Rrp1b |
| GO:0044427 | chromosomal part | 4.83e-01 | 7.39e-01 | 1 | 22 | 486 | 16439 | 4.55% | 2.96% | 0.21% | 0.13% | CL\_0000822\_down | Hmgn3 |
| GO:0016023 | cytoplasmic membrane-bounded vesicle | 5.19e-01 | 7.74e-01 | 1 | 22 | 537 | 16439 | 4.55% | 3.27% | 0.19% | 0.13% | CL\_0000822\_down | Itgb1 |
| GO:0045202 | synapse | 5.21e-01 | 7.74e-01 | 1 | 22 | 540 | 16439 | 4.55% | 3.28% | 0.19% | 0.13% | CL\_0000822\_down | Itgb1 |
| GO:0005694 | chromosome | 5.39e-01 | 7.84e-01 | 1 | 22 | 568 | 16439 | 4.55% | 3.46% | 0.18% | 0.13% | CL\_0000822\_down | Hmgn3 |
| GO:0031988 | membrane-bounded vesicle | 5.41e-01 | 7.84e-01 | 1 | 22 | 572 | 16439 | 4.55% | 3.48% | 0.17% | 0.13% | CL\_0000822\_down | Itgb1 |
| GO:0031981 | nuclear lumen | 5.71e-01 | 7.89e-01 | 2 | 22 | 1402 | 16439 | 9.09% | 8.53% | 0.14% | 0.13% | CL\_0000822\_down | Rrp1b, Tfdp1 |
| GO:0043234 | protein complex | 5.73e-01 | 7.89e-01 | 4 | 22 | 2944 | 16439 | 18.18% | 17.91% | 0.14% | 0.13% | CL\_0000822\_down | 2010001M09Rik, Itgb1, Ly96, Tfdp1 |
| GO:0005887 | integral to plasma membrane | 5.83e-01 | 7.89e-01 | 1 | 22 | 641 | 16439 | 4.55% | 3.90% | 0.16% | 0.13% | CL\_0000822\_down | Itgb1 |
| GO:0031226 | intrinsic to plasma membrane | 6.08e-01 | 7.89e-01 | 1 | 22 | 684 | 16439 | 4.55% | 4.16% | 0.15% | 0.13% | CL\_0000822\_down | Itgb1 |
| GO:0015630 | microtubule cytoskeleton | 6.21e-01 | 7.89e-01 | 1 | 22 | 709 | 16439 | 4.55% | 4.31% | 0.14% | 0.13% | CL\_0000822\_down | Rps6ka1 |
| GO:0030054 | cell junction | 6.22e-01 | 7.89e-01 | 1 | 22 | 711 | 16439 | 4.55% | 4.33% | 0.14% | 0.13% | CL\_0000822\_down | Itgb1 |
| GO:0044451 | nucleoplasm part | 6.33e-01 | 7.89e-01 | 1 | 22 | 732 | 16439 | 4.55% | 4.45% | 0.14% | 0.13% | CL\_0000822\_down | Tfdp1 |
| GO:0012505 | endomembrane system | 6.34e-01 | 7.89e-01 | 1 | 22 | 734 | 16439 | 4.55% | 4.46% | 0.14% | 0.13% | CL\_0000822\_down | Tmem18 |
| GO:0044459 | plasma membrane part | 6.39e-01 | 7.89e-01 | 2 | 22 | 1582 | 16439 | 9.09% | 9.62% | 0.13% | 0.13% | CL\_0000822\_down | Itgb1, Sema7a |
| GO:0043231 | intracellular membrane-bounded organelle | 6.42e-01 | 7.89e-01 | 11 | 22 | 8480 | 16439 | 50.00% | 51.58% | 0.13% | 0.13% | CL\_0000822\_down | 2010001M09Rik, Bri3bp, Hmgn3, Itgb1, Rps6ka1, Rrp1b, Sfxn1, Slc25a4, Taf1d, Tfdp1, Tmem18 |
| GO:0070013 | intracellular organelle lumen | 6.43e-01 | 7.89e-01 | 2 | 22 | 1593 | 16439 | 9.09% | 9.69% | 0.13% | 0.13% | CL\_0000822\_down | Rrp1b, Tfdp1 |
| GO:0031410 | cytoplasmic vesicle | 6.45e-01 | 7.89e-01 | 1 | 22 | 755 | 16439 | 4.55% | 4.59% | 0.13% | 0.13% | CL\_0000822\_down | Itgb1 |
| GO:0043233 | organelle lumen | 6.45e-01 | 7.89e-01 | 2 | 22 | 1598 | 16439 | 9.09% | 9.72% | 0.13% | 0.13% | CL\_0000822\_down | Rrp1b, Tfdp1 |
| GO:0043227 | membrane-bounded organelle | 6.46e-01 | 7.89e-01 | 11 | 22 | 8499 | 16439 | 50.00% | 51.70% | 0.13% | 0.13% | CL\_0000822\_down | 2010001M09Rik, Bri3bp, Hmgn3, Itgb1, Rps6ka1, Rrp1b, Sfxn1, Slc25a4, Taf1d, Tfdp1, Tmem18 |
| GO:0031974 | membrane-enclosed lumen | 6.59e-01 | 7.97e-01 | 2 | 22 | 1639 | 16439 | 9.09% | 9.97% | 0.12% | 0.13% | CL\_0000822\_down | Rrp1b, Tfdp1 |
| GO:0031982 | vesicle | 6.71e-01 | 8.01e-01 | 1 | 22 | 810 | 16439 | 4.55% | 4.93% | 0.12% | 0.13% | CL\_0000822\_down | Itgb1 |
| GO:0005654 | nucleoplasm | 6.77e-01 | 8.01e-01 | 1 | 22 | 823 | 16439 | 4.55% | 5.01% | 0.12% | 0.13% | CL\_0000822\_down | Tfdp1 |
| GO:0005576 | extracellular region | 7.15e-01 | 8.17e-01 | 2 | 22 | 1812 | 16439 | 9.09% | 11.02% | 0.11% | 0.13% | CL\_0000822\_down | Itgb1, Ly96 |
| GO:0044424 | intracellular part | 7.25e-01 | 8.17e-01 | 14 | 22 | 11042 | 16439 | 63.64% | 67.17% | 0.13% | 0.13% | CL\_0000822\_down | 2010001M09Rik, Akr1e1, Arhgef12, Bri3bp, Hmgn3, Itgb1, Rps6ka1, Rrp1b, Sfxn1, Slc25a4, Taf1d, Tfdp1, Tmem18, Uck2 |
| GO:0044464 | cell part | 7.38e-01 | 8.17e-01 | 17 | 22 | 13174 | 16439 | 77.27% | 80.14% | 0.13% | 0.13% | CL\_0000822\_down | 2010001M09Rik, Akr1e1, Arhgef12, Bri3bp, Ccbp2, Hmgn3, Itgb1, Rps6ka1, Rrp1b, Sema7a, Sfxn1, Slc20a2, Slc25a4, Taf1d, Tfdp1, Tmem18, Uck2 |
| GO:0005623 | cell | 7.39e-01 | 8.17e-01 | 17 | 22 | 13175 | 16439 | 77.27% | 80.14% | 0.13% | 0.13% | CL\_0000822\_down | 2010001M09Rik, Akr1e1, Arhgef12, Bri3bp, Ccbp2, Hmgn3, Itgb1, Rps6ka1, Rrp1b, Sema7a, Sfxn1, Slc20a2, Slc25a4, Taf1d, Tfdp1, Tmem18, Uck2 |
| GO:0005634 | nucleus | 7.43e-01 | 8.17e-01 | 6 | 22 | 5200 | 16439 | 27.27% | 31.63% | 0.12% | 0.13% | CL\_0000822\_down | Hmgn3, Rps6ka1, Rrp1b, Taf1d, Tfdp1, Tmem18 |
| GO:0005886 | plasma membrane | 7.53e-01 | 8.17e-01 | 4 | 22 | 3649 | 16439 | 18.18% | 22.20% | 0.11% | 0.13% | CL\_0000822\_down | Ccbp2, Itgb1, Sema7a, Slc20a2 |
| GO:0044421 | extracellular region part | 7.59e-01 | 8.17e-01 | 1 | 22 | 1029 | 16439 | 4.55% | 6.26% | 0.10% | 0.13% | CL\_0000822\_down | Itgb1 |
| GO:0043232 | intracellular non-membrane-bounded organelle | 7.61e-01 | 8.17e-01 | 3 | 22 | 2851 | 16439 | 13.64% | 17.34% | 0.11% | 0.13% | CL\_0000822\_down | Hmgn3, Rps6ka1, Rrp1b |
| GO:0043228 | non-membrane-bounded organelle | 7.61e-01 | 8.17e-01 | 3 | 22 | 2851 | 16439 | 13.64% | 17.34% | 0.11% | 0.13% | CL\_0000822\_down | Hmgn3, Rps6ka1, Rrp1b |
| GO:0071944 | cell periphery | 7.71e-01 | 8.17e-01 | 4 | 22 | 3737 | 16439 | 18.18% | 22.73% | 0.11% | 0.13% | CL\_0000822\_down | Ccbp2, Itgb1, Sema7a, Slc20a2 |
| GO:0005622 | intracellular | 7.73e-01 | 8.17e-01 | 14 | 22 | 11282 | 16439 | 63.64% | 68.63% | 0.12% | 0.13% | CL\_0000822\_down | 2010001M09Rik, Akr1e1, Arhgef12, Bri3bp, Hmgn3, Itgb1, Rps6ka1, Rrp1b, Sfxn1, Slc25a4, Taf1d, Tfdp1, Tmem18, Uck2 |
| GO:0005783 | endoplasmic reticulum | 7.80e-01 | 8.17e-01 | 1 | 22 | 1093 | 16439 | 4.55% | 6.65% | 0.09% | 0.13% | CL\_0000822\_down | 2010001M09Rik |
| GO:0044430 | cytoskeletal part | 7.90e-01 | 8.19e-01 | 1 | 22 | 1124 | 16439 | 4.55% | 6.84% | 0.09% | 0.13% | CL\_0000822\_down | Rps6ka1 |
| GO:0043229 | intracellular organelle | 8.44e-01 | 8.63e-01 | 11 | 22 | 9595 | 16439 | 50.00% | 58.37% | 0.11% | 0.13% | CL\_0000822\_down | 2010001M09Rik, Bri3bp, Hmgn3, Itgb1, Rps6ka1, Rrp1b, Sfxn1, Slc25a4, Taf1d, Tfdp1, Tmem18 |
| GO:0043226 | organelle | 8.47e-01 | 8.63e-01 | 11 | 22 | 9616 | 16439 | 50.00% | 58.50% | 0.11% | 0.13% | CL\_0000822\_down | 2010001M09Rik, Bri3bp, Hmgn3, Itgb1, Rps6ka1, Rrp1b, Sfxn1, Slc25a4, Taf1d, Tfdp1, Tmem18 |
| GO:0005856 | cytoskeleton | 9.04e-01 | 9.13e-01 | 1 | 22 | 1662 | 16439 | 4.55% | 10.11% | 0.06% | 0.13% | CL\_0000822\_down | Rps6ka1 |
| GO:0005575 | cellular\_component | 1.00e+00 | 1.00e+00 | 22 | 22 | 16439 | 16439 | 100.00% | 100.00% | 0.13% | 0.13% | CL\_0000822\_down | 2010001M09Rik, Akr1e1, Arhgef12, Bri3bp, Ccbp2, Emb, Hmgn3, Itgb1, Kcnk5, Ly96, Rps6ka1, Rrp1b, Sema7a, Sfxn1, Slc20a2, Slc25a4, Slc35f2, Slc36a4, Taf1d, Tfdp1, Tmem18, Uck2 |

### molecular\_function (top)

  
  


| TermID | Term | P | Q | k | n | K | N | k/n | K/N | k/K | n/N | Qset | Symbols |
| --- | --- | --- | --- | --- | --- | --- | --- | --- | --- | --- | --- | --- | --- |
| GO:0050571 | 1,5-anhydro-D-fructose reductase activity | 1.28e-03 | 5.51e-02 | 1 | 19 | 1 | 14853 | 5.26% | 0.01% | 100.00% | 0.13% | CL\_0000822\_down | Akr1e1 |
| GO:0004766 | spermidine synthase activity | 1.28e-03 | 5.51e-02 | 1 | 19 | 1 | 14853 | 5.26% | 0.01% | 100.00% | 0.13% | CL\_0000822\_down | Srm |
| GO:0015291 | secondary active transmembrane transporter activity | 1.36e-03 | 5.51e-02 | 3 | 19 | 175 | 14853 | 15.79% | 1.18% | 1.71% | 0.13% | CL\_0000822\_down | Slc20a2, Slc25a4, Slc36a4 |
| GO:0005471 | ATP:ADP antiporter activity | 2.56e-03 | 6.68e-02 | 1 | 19 | 2 | 14853 | 5.26% | 0.01% | 50.00% | 0.13% | CL\_0000822\_down | Slc25a4 |
| GO:0005178 | integrin binding | 3.36e-03 | 6.68e-02 | 2 | 19 | 68 | 14853 | 10.53% | 0.46% | 2.94% | 0.13% | CL\_0000822\_down | Itgb1, Sema7a |
| GO:0022857 | transmembrane transporter activity | 3.53e-03 | 6.68e-02 | 5 | 19 | 846 | 14853 | 26.32% | 5.70% | 0.59% | 0.13% | CL\_0000822\_down | Kcnk5, Sfxn1, Slc20a2, Slc25a4, Slc36a4 |
| GO:0004849 | uridine kinase activity | 3.83e-03 | 6.68e-02 | 1 | 19 | 3 | 14853 | 5.26% | 0.02% | 33.33% | 0.13% | CL\_0000822\_down | Uck2 |
| GO:0005315 | inorganic phosphate transmembrane transporter activity | 5.11e-03 | 6.92e-02 | 1 | 19 | 4 | 14853 | 5.26% | 0.03% | 25.00% | 0.13% | CL\_0000822\_down | Slc20a2 |
| GO:0001875 | lipopolysaccharide receptor activity | 5.11e-03 | 6.92e-02 | 1 | 19 | 4 | 14853 | 5.26% | 0.03% | 25.00% | 0.13% | CL\_0000822\_down | Ly96 |
| GO:0031492 | nucleosomal DNA binding | 6.38e-03 | 7.48e-02 | 1 | 19 | 5 | 14853 | 5.26% | 0.03% | 20.00% | 0.13% | CL\_0000822\_down | Hmgn3 |
| GO:0022804 | active transmembrane transporter activity | 6.75e-03 | 7.48e-02 | 3 | 19 | 309 | 14853 | 15.79% | 2.08% | 0.97% | 0.13% | CL\_0000822\_down | Slc20a2, Slc25a4, Slc36a4 |
| GO:0005215 | transporter activity | 8.61e-03 | 7.77e-02 | 5 | 19 | 1044 | 14853 | 26.32% | 7.03% | 0.48% | 0.13% | CL\_0000822\_down | Kcnk5, Sfxn1, Slc20a2, Slc25a4, Slc36a4 |
| GO:0019957 | C-C chemokine binding | 8.92e-03 | 7.77e-02 | 1 | 19 | 7 | 14853 | 5.26% | 0.05% | 14.29% | 0.13% | CL\_0000822\_down | Ccbp2 |
| GO:0015293 | symporter activity | 9.96e-03 | 7.77e-02 | 2 | 19 | 119 | 14853 | 10.53% | 0.80% | 1.68% | 0.13% | CL\_0000822\_down | Slc20a2, Slc36a4 |
| GO:0016494 | C-X-C chemokine receptor activity | 1.02e-02 | 7.77e-02 | 1 | 19 | 8 | 14853 | 5.26% | 0.05% | 12.50% | 0.13% | CL\_0000822\_down | Ccbp2 |
| GO:0019206 | nucleoside kinase activity | 1.02e-02 | 7.77e-02 | 1 | 19 | 8 | 14853 | 5.26% | 0.05% | 12.50% | 0.13% | CL\_0000822\_down | Uck2 |
| GO:0015114 | phosphate ion transmembrane transporter activity | 1.15e-02 | 8.22e-02 | 1 | 19 | 9 | 14853 | 5.26% | 0.06% | 11.11% | 0.13% | CL\_0000822\_down | Slc20a2 |
| GO:0019956 | chemokine binding | 1.52e-02 | 9.30e-02 | 1 | 19 | 12 | 14853 | 5.26% | 0.08% | 8.33% | 0.13% | CL\_0000822\_down | Ccbp2 |
| GO:0031491 | nucleosome binding | 1.52e-02 | 9.30e-02 | 1 | 19 | 12 | 14853 | 5.26% | 0.08% | 8.33% | 0.13% | CL\_0000822\_down | Hmgn3 |
| GO:0008329 | pattern recognition receptor activity | 1.52e-02 | 9.30e-02 | 1 | 19 | 12 | 14853 | 5.26% | 0.08% | 8.33% | 0.13% | CL\_0000822\_down | Ly96 |
| GO:0051393 | alpha-actinin binding | 1.65e-02 | 9.59e-02 | 1 | 19 | 13 | 14853 | 5.26% | 0.09% | 7.69% | 0.13% | CL\_0000822\_down | Itgb1 |
| GO:0016493 | C-C chemokine receptor activity | 1.78e-02 | 9.85e-02 | 1 | 19 | 14 | 14853 | 5.26% | 0.09% | 7.14% | 0.13% | CL\_0000822\_down | Ccbp2 |
| GO:0042805 | actinin binding | 2.28e-02 | 1.21e-01 | 1 | 19 | 18 | 14853 | 5.26% | 0.12% | 5.56% | 0.13% | CL\_0000822\_down | Itgb1 |
| GO:0001968 | fibronectin binding | 2.53e-02 | 1.29e-01 | 1 | 19 | 20 | 14853 | 5.26% | 0.13% | 5.00% | 0.13% | CL\_0000822\_down | Itgb1 |
| GO:0043236 | laminin binding | 2.78e-02 | 1.31e-01 | 1 | 19 | 22 | 14853 | 5.26% | 0.15% | 4.55% | 0.13% | CL\_0000822\_down | Itgb1 |
| GO:0001637 | G-protein coupled chemoattractant receptor activity | 2.90e-02 | 1.31e-01 | 1 | 19 | 23 | 14853 | 5.26% | 0.15% | 4.35% | 0.13% | CL\_0000822\_down | Ccbp2 |
| GO:0004950 | chemokine receptor activity | 2.90e-02 | 1.31e-01 | 1 | 19 | 23 | 14853 | 5.26% | 0.15% | 4.35% | 0.13% | CL\_0000822\_down | Ccbp2 |
| GO:0043027 | cysteine-type endopeptidase inhibitor activity involved in apoptotic process | 3.03e-02 | 1.32e-01 | 1 | 19 | 24 | 14853 | 5.26% | 0.16% | 4.17% | 0.13% | CL\_0000822\_down | Rps6ka1 |
| GO:0031490 | chromatin DNA binding | 3.89e-02 | 1.64e-01 | 1 | 19 | 31 | 14853 | 5.26% | 0.21% | 3.23% | 0.13% | CL\_0000822\_down | Hmgn3 |
| GO:0019205 | nucleobase-containing compound kinase activity | 4.51e-02 | 1.73e-01 | 1 | 19 | 36 | 14853 | 5.26% | 0.24% | 2.78% | 0.13% | CL\_0000822\_down | Uck2 |
| GO:0043028 | cysteine-type endopeptidase regulator activity involved in apoptotic process | 4.63e-02 | 1.73e-01 | 1 | 19 | 37 | 14853 | 5.26% | 0.25% | 2.70% | 0.13% | CL\_0000822\_down | Rps6ka1 |
| GO:0050840 | extracellular matrix binding | 4.75e-02 | 1.73e-01 | 1 | 19 | 38 | 14853 | 5.26% | 0.26% | 2.63% | 0.13% | CL\_0000822\_down | Itgb1 |
| GO:0015300 | solute:solute antiporter activity | 4.75e-02 | 1.73e-01 | 1 | 19 | 38 | 14853 | 5.26% | 0.26% | 2.63% | 0.13% | CL\_0000822\_down | Slc25a4 |
| GO:0015075 | ion transmembrane transporter activity | 4.81e-02 | 1.73e-01 | 3 | 19 | 651 | 14853 | 15.79% | 4.38% | 0.46% | 0.13% | CL\_0000822\_down | Kcnk5, Sfxn1, Slc20a2 |
| GO:0005518 | collagen binding | 5.12e-02 | 1.78e-01 | 1 | 19 | 41 | 14853 | 5.26% | 0.28% | 2.44% | 0.13% | CL\_0000822\_down | Itgb1 |
| GO:0015297 | antiporter activity | 6.21e-02 | 2.10e-01 | 1 | 19 | 50 | 14853 | 5.26% | 0.34% | 2.00% | 0.13% | CL\_0000822\_down | Slc25a4 |
| GO:0016765 | transferase activity, transferring alkyl or aryl (other than methyl) groups | 6.69e-02 | 2.15e-01 | 1 | 19 | 54 | 14853 | 5.26% | 0.36% | 1.85% | 0.13% | CL\_0000822\_down | Srm |
| GO:0004869 | cysteine-type endopeptidase inhibitor activity | 6.81e-02 | 2.15e-01 | 1 | 19 | 55 | 14853 | 5.26% | 0.37% | 1.82% | 0.13% | CL\_0000822\_down | Rps6ka1 |
| GO:0022891 | substrate-specific transmembrane transporter activity | 6.93e-02 | 2.15e-01 | 3 | 19 | 756 | 14853 | 15.79% | 5.09% | 0.40% | 0.13% | CL\_0000822\_down | Kcnk5, Sfxn1, Slc20a2 |
| GO:0019955 | cytokine binding | 7.05e-02 | 2.15e-01 | 1 | 19 | 57 | 14853 | 5.26% | 0.38% | 1.75% | 0.13% | CL\_0000822\_down | Ccbp2 |
| GO:0005089 | Rho guanyl-nucleotide exchange factor activity | 7.64e-02 | 2.27e-01 | 1 | 19 | 62 | 14853 | 5.26% | 0.42% | 1.61% | 0.13% | CL\_0000822\_down | Arhgef12 |
| GO:0002020 | protease binding | 7.88e-02 | 2.29e-01 | 1 | 19 | 64 | 14853 | 5.26% | 0.43% | 1.56% | 0.13% | CL\_0000822\_down | Itgb1 |
| GO:0001948 | glycoprotein binding | 8.35e-02 | 2.37e-01 | 1 | 19 | 68 | 14853 | 5.26% | 0.46% | 1.47% | 0.13% | CL\_0000822\_down | Itgb1 |
| GO:0004896 | cytokine receptor activity | 9.06e-02 | 2.43e-01 | 1 | 19 | 74 | 14853 | 5.26% | 0.50% | 1.35% | 0.13% | CL\_0000822\_down | Ccbp2 |
| GO:0005088 | Ras guanyl-nucleotide exchange factor activity | 9.41e-02 | 2.43e-01 | 1 | 19 | 77 | 14853 | 5.26% | 0.52% | 1.30% | 0.13% | CL\_0000822\_down | Arhgef12 |
| GO:0008134 | transcription factor binding | 9.43e-02 | 2.43e-01 | 2 | 19 | 407 | 14853 | 10.53% | 2.74% | 0.49% | 0.13% | CL\_0000822\_down | Rps6ka1, Tfdp1 |
| GO:0022892 | substrate-specific transporter activity | 9.55e-02 | 2.43e-01 | 3 | 19 | 866 | 14853 | 15.79% | 5.83% | 0.35% | 0.13% | CL\_0000822\_down | Kcnk5, Sfxn1, Slc20a2 |
| GO:0005515 | protein binding | 9.56e-02 | 2.43e-01 | 11 | 19 | 6013 | 14853 | 57.89% | 40.48% | 0.18% | 0.13% | CL\_0000822\_down | Arhgef12, Ccbp2, Fam92a, Itgb1, Ly96, Rps6ka1, Sema7a, Slc25a4, Srm, Tfdp1, Uck2 |
| GO:0032403 | protein complex binding | 9.79e-02 | 2.44e-01 | 2 | 19 | 416 | 14853 | 10.53% | 2.80% | 0.48% | 0.13% | CL\_0000822\_down | Itgb1, Sema7a |
| GO:0015103 | inorganic anion transmembrane transporter activity | 1.19e-01 | 2.91e-01 | 1 | 19 | 99 | 14853 | 5.26% | 0.67% | 1.01% | 0.13% | CL\_0000822\_down | Slc20a2 |
| GO:0008528 | G-protein coupled peptide receptor activity | 1.33e-01 | 3.17e-01 | 1 | 19 | 111 | 14853 | 5.26% | 0.75% | 0.90% | 0.13% | CL\_0000822\_down | Ccbp2 |
| GO:0001653 | peptide receptor activity | 1.35e-01 | 3.17e-01 | 1 | 19 | 113 | 14853 | 5.26% | 0.76% | 0.88% | 0.13% | CL\_0000822\_down | Ccbp2 |
| GO:0016616 | oxidoreductase activity, acting on the CH-OH group of donors, NAD or NADP as acceptor | 1.50e-01 | 3.44e-01 | 1 | 19 | 126 | 14853 | 5.26% | 0.85% | 0.79% | 0.13% | CL\_0000822\_down | Akr1e1 |
| GO:0008509 | anion transmembrane transporter activity | 1.58e-01 | 3.58e-01 | 1 | 19 | 134 | 14853 | 5.26% | 0.90% | 0.75% | 0.13% | CL\_0000822\_down | Slc20a2 |
| GO:0016614 | oxidoreductase activity, acting on CH-OH group of donors | 1.62e-01 | 3.58e-01 | 1 | 19 | 137 | 14853 | 5.26% | 0.92% | 0.73% | 0.13% | CL\_0000822\_down | Akr1e1 |
| GO:0005085 | guanyl-nucleotide exchange factor activity | 1.66e-01 | 3.61e-01 | 1 | 19 | 141 | 14853 | 5.26% | 0.95% | 0.71% | 0.13% | CL\_0000822\_down | Arhgef12 |
| GO:0019904 | protein domain specific binding | 1.78e-01 | 3.70e-01 | 2 | 19 | 601 | 14853 | 10.53% | 4.05% | 0.33% | 0.13% | CL\_0000822\_down | Itgb1, Tfdp1 |
| GO:0042277 | peptide binding | 1.80e-01 | 3.70e-01 | 1 | 19 | 154 | 14853 | 5.26% | 1.04% | 0.65% | 0.13% | CL\_0000822\_down | Itgb1 |
| GO:0005102 | receptor binding | 1.81e-01 | 3.70e-01 | 3 | 19 | 1159 | 14853 | 15.79% | 7.80% | 0.26% | 0.13% | CL\_0000822\_down | Arhgef12, Itgb1, Sema7a |
| GO:0003677 | DNA binding | 1.84e-01 | 3.70e-01 | 4 | 19 | 1774 | 14853 | 21.05% | 11.94% | 0.23% | 0.13% | CL\_0000822\_down | Hmgn3, Taf1d, Tfdp1, Tmem18 |
| GO:0000287 | magnesium ion binding | 1.85e-01 | 3.70e-01 | 1 | 19 | 159 | 14853 | 5.26% | 1.07% | 0.63% | 0.13% | CL\_0000822\_down | Rps6ka1 |
| GO:0004866 | endopeptidase inhibitor activity | 2.02e-01 | 3.94e-01 | 1 | 19 | 175 | 14853 | 5.26% | 1.18% | 0.57% | 0.13% | CL\_0000822\_down | Rps6ka1 |
| GO:0061135 | endopeptidase regulator activity | 2.08e-01 | 3.94e-01 | 1 | 19 | 181 | 14853 | 5.26% | 1.22% | 0.55% | 0.13% | CL\_0000822\_down | Rps6ka1 |
| GO:0016773 | phosphotransferase activity, alcohol group as acceptor | 2.09e-01 | 3.94e-01 | 2 | 19 | 666 | 14853 | 10.53% | 4.48% | 0.30% | 0.13% | CL\_0000822\_down | Rps6ka1, Uck2 |
| GO:0030414 | peptidase inhibitor activity | 2.10e-01 | 3.94e-01 | 1 | 19 | 183 | 14853 | 5.26% | 1.23% | 0.55% | 0.13% | CL\_0000822\_down | Rps6ka1 |
| GO:0001664 | G-protein coupled receptor binding | 2.24e-01 | 4.14e-01 | 1 | 19 | 197 | 14853 | 5.26% | 1.33% | 0.51% | 0.13% | CL\_0000822\_down | Arhgef12 |
| GO:0005096 | GTPase activator activity | 2.30e-01 | 4.19e-01 | 1 | 19 | 203 | 14853 | 5.26% | 1.37% | 0.49% | 0.13% | CL\_0000822\_down | Arhgef12 |
| GO:0061134 | peptidase regulator activity | 2.39e-01 | 4.29e-01 | 1 | 19 | 212 | 14853 | 5.26% | 1.43% | 0.47% | 0.13% | CL\_0000822\_down | Rps6ka1 |
| GO:0005083 | small GTPase regulator activity | 2.45e-01 | 4.31e-01 | 1 | 19 | 218 | 14853 | 5.26% | 1.47% | 0.46% | 0.13% | CL\_0000822\_down | Arhgef12 |
| GO:0004872 | receptor activity | 2.51e-01 | 4.31e-01 | 5 | 19 | 2687 | 14853 | 26.32% | 18.09% | 0.19% | 0.13% | CL\_0000822\_down | Ccbp2, Itgb1, Ly96, Sema7a, Slc20a2 |
| GO:0043566 | structure-specific DNA binding | 2.51e-01 | 4.31e-01 | 1 | 19 | 224 | 14853 | 5.26% | 1.51% | 0.45% | 0.13% | CL\_0000822\_down | Hmgn3 |
| GO:0016301 | kinase activity | 2.62e-01 | 4.45e-01 | 2 | 19 | 778 | 14853 | 10.53% | 5.24% | 0.26% | 0.13% | CL\_0000822\_down | Rps6ka1, Uck2 |
| GO:0046983 | protein dimerization activity | 2.86e-01 | 4.74e-01 | 2 | 19 | 826 | 14853 | 10.53% | 5.56% | 0.24% | 0.13% | CL\_0000822\_down | Itgb1, Srm |
| GO:0030234 | enzyme regulator activity | 2.88e-01 | 4.74e-01 | 2 | 19 | 830 | 14853 | 10.53% | 5.59% | 0.24% | 0.13% | CL\_0000822\_down | Arhgef12, Rps6ka1 |
| GO:0003682 | chromatin binding | 3.02e-01 | 4.91e-01 | 1 | 19 | 278 | 14853 | 5.26% | 1.87% | 0.36% | 0.13% | CL\_0000822\_down | Hmgn3 |
| GO:0004857 | enzyme inhibitor activity | 3.21e-01 | 5.12e-01 | 1 | 19 | 299 | 14853 | 5.26% | 2.01% | 0.33% | 0.13% | CL\_0000822\_down | Rps6ka1 |
| GO:0016772 | transferase activity, transferring phosphorus-containing groups | 3.23e-01 | 5.12e-01 | 2 | 19 | 904 | 14853 | 10.53% | 6.09% | 0.22% | 0.13% | CL\_0000822\_down | Rps6ka1, Uck2 |
| GO:0003779 | actin binding | 3.39e-01 | 5.30e-01 | 1 | 19 | 320 | 14853 | 5.26% | 2.15% | 0.31% | 0.13% | CL\_0000822\_down | Itgb1 |
| GO:0008047 | enzyme activator activity | 3.43e-01 | 5.30e-01 | 1 | 19 | 325 | 14853 | 5.26% | 2.19% | 0.31% | 0.13% | CL\_0000822\_down | Arhgef12 |
| GO:0046982 | protein heterodimerization activity | 3.50e-01 | 5.34e-01 | 1 | 19 | 333 | 14853 | 5.26% | 2.24% | 0.30% | 0.13% | CL\_0000822\_down | Itgb1 |
| GO:0030695 | GTPase regulator activity | 3.65e-01 | 5.45e-01 | 1 | 19 | 351 | 14853 | 5.26% | 2.36% | 0.28% | 0.13% | CL\_0000822\_down | Arhgef12 |
| GO:0016740 | transferase activity | 3.66e-01 | 5.45e-01 | 3 | 19 | 1681 | 14853 | 15.79% | 11.32% | 0.18% | 0.13% | CL\_0000822\_down | Rps6ka1, Srm, Uck2 |
| GO:0060589 | nucleoside-triphosphatase regulator activity | 3.75e-01 | 5.51e-01 | 1 | 19 | 363 | 14853 | 5.26% | 2.44% | 0.28% | 0.13% | CL\_0000822\_down | Arhgef12 |
| GO:0005216 | ion channel activity | 3.79e-01 | 5.51e-01 | 1 | 19 | 368 | 14853 | 5.26% | 2.48% | 0.27% | 0.13% | CL\_0000822\_down | Kcnk5 |
| GO:0022838 | substrate-specific channel activity | 3.87e-01 | 5.52e-01 | 1 | 19 | 377 | 14853 | 5.26% | 2.54% | 0.27% | 0.13% | CL\_0000822\_down | Kcnk5 |
| GO:0019901 | protein kinase binding | 3.91e-01 | 5.52e-01 | 1 | 19 | 382 | 14853 | 5.26% | 2.57% | 0.26% | 0.13% | CL\_0000822\_down | Itgb1 |
| GO:0015267 | channel activity | 3.98e-01 | 5.52e-01 | 1 | 19 | 391 | 14853 | 5.26% | 2.63% | 0.26% | 0.13% | CL\_0000822\_down | Kcnk5 |
| GO:0022803 | passive transmembrane transporter activity | 3.98e-01 | 5.52e-01 | 1 | 19 | 391 | 14853 | 5.26% | 2.63% | 0.26% | 0.13% | CL\_0000822\_down | Kcnk5 |
| GO:0004674 | protein serine/threonine kinase activity | 4.12e-01 | 5.65e-01 | 1 | 19 | 409 | 14853 | 5.26% | 2.75% | 0.24% | 0.13% | CL\_0000822\_down | Rps6ka1 |
| GO:0005543 | phospholipid binding | 4.20e-01 | 5.68e-01 | 1 | 19 | 420 | 14853 | 5.26% | 2.83% | 0.24% | 0.13% | CL\_0000822\_down | Arhgef12 |
| GO:0019899 | enzyme binding | 4.23e-01 | 5.68e-01 | 2 | 19 | 1116 | 14853 | 10.53% | 7.51% | 0.18% | 0.13% | CL\_0000822\_down | Itgb1, Slc25a4 |
| GO:0003676 | nucleic acid binding | 4.30e-01 | 5.70e-01 | 4 | 19 | 2594 | 14853 | 21.05% | 17.46% | 0.15% | 0.13% | CL\_0000822\_down | Hmgn3, Taf1d, Tfdp1, Tmem18 |
| GO:0019900 | kinase binding | 4.35e-01 | 5.70e-01 | 1 | 19 | 439 | 14853 | 5.26% | 2.96% | 0.23% | 0.13% | CL\_0000822\_down | Itgb1 |
| GO:0008324 | cation transmembrane transporter activity | 4.62e-01 | 6.00e-01 | 1 | 19 | 477 | 14853 | 5.26% | 3.21% | 0.21% | 0.13% | CL\_0000822\_down | Sfxn1 |
| GO:0042803 | protein homodimerization activity | 5.18e-01 | 6.60e-01 | 1 | 19 | 559 | 14853 | 5.26% | 3.76% | 0.18% | 0.13% | CL\_0000822\_down | Srm |
| GO:0004672 | protein kinase activity | 5.19e-01 | 6.60e-01 | 1 | 19 | 561 | 14853 | 5.26% | 3.78% | 0.18% | 0.13% | CL\_0000822\_down | Rps6ka1 |
| GO:0008092 | cytoskeletal protein binding | 5.28e-01 | 6.64e-01 | 1 | 19 | 575 | 14853 | 5.26% | 3.87% | 0.17% | 0.13% | CL\_0000822\_down | Itgb1 |
| GO:0005524 | ATP binding | 5.53e-01 | 6.82e-01 | 2 | 19 | 1416 | 14853 | 10.53% | 9.53% | 0.14% | 0.13% | CL\_0000822\_down | Rps6ka1, Uck2 |
| GO:0008289 | lipid binding | 5.58e-01 | 6.82e-01 | 1 | 19 | 625 | 14853 | 5.26% | 4.21% | 0.16% | 0.13% | CL\_0000822\_down | Arhgef12 |
| GO:0032559 | adenyl ribonucleotide binding | 5.62e-01 | 6.82e-01 | 2 | 19 | 1440 | 14853 | 10.53% | 9.70% | 0.14% | 0.13% | CL\_0000822\_down | Rps6ka1, Uck2 |
| GO:0030554 | adenyl nucleotide binding | 5.65e-01 | 6.82e-01 | 2 | 19 | 1447 | 14853 | 10.53% | 9.74% | 0.14% | 0.13% | CL\_0000822\_down | Rps6ka1, Uck2 |
| GO:0005488 | binding | 5.72e-01 | 6.85e-01 | 14 | 19 | 10773 | 14853 | 73.68% | 72.53% | 0.13% | 0.13% | CL\_0000822\_down | Arhgef12, Ccbp2, Fam92a, Hmgn3, Itgb1, Ly96, Rps6ka1, Sema7a, Slc25a4, Srm, Taf1d, Tfdp1, Tmem18, Uck2 |
| GO:0016491 | oxidoreductase activity | 6.16e-01 | 7.30e-01 | 1 | 19 | 730 | 14853 | 5.26% | 4.91% | 0.14% | 0.13% | CL\_0000822\_down | Akr1e1 |
| GO:0003700 | sequence-specific DNA binding transcription factor activity | 6.29e-01 | 7.32e-01 | 1 | 19 | 754 | 14853 | 5.26% | 5.08% | 0.13% | 0.13% | CL\_0000822\_down | Tfdp1 |
| GO:0001071 | nucleic acid binding transcription factor activity | 6.30e-01 | 7.32e-01 | 1 | 19 | 756 | 14853 | 5.26% | 5.09% | 0.13% | 0.13% | CL\_0000822\_down | Tfdp1 |
| GO:0042802 | identical protein binding | 6.64e-01 | 7.54e-01 | 1 | 19 | 829 | 14853 | 5.26% | 5.58% | 0.12% | 0.13% | CL\_0000822\_down | Srm |
| GO:0035639 | purine ribonucleoside triphosphate binding | 6.66e-01 | 7.54e-01 | 2 | 19 | 1728 | 14853 | 10.53% | 11.63% | 0.12% | 0.13% | CL\_0000822\_down | Rps6ka1, Uck2 |
| GO:0032555 | purine ribonucleotide binding | 6.77e-01 | 7.54e-01 | 2 | 19 | 1761 | 14853 | 10.53% | 11.86% | 0.11% | 0.13% | CL\_0000822\_down | Rps6ka1, Uck2 |
| GO:0032553 | ribonucleotide binding | 6.77e-01 | 7.54e-01 | 2 | 19 | 1762 | 14853 | 10.53% | 11.86% | 0.11% | 0.13% | CL\_0000822\_down | Rps6ka1, Uck2 |
| GO:0017076 | purine nucleotide binding | 6.79e-01 | 7.54e-01 | 2 | 19 | 1769 | 14853 | 10.53% | 11.91% | 0.11% | 0.13% | CL\_0000822\_down | Rps6ka1, Uck2 |
| GO:0000166 | nucleotide binding | 7.99e-01 | 8.79e-01 | 2 | 19 | 2217 | 14853 | 10.53% | 14.93% | 0.09% | 0.13% | CL\_0000822\_down | Rps6ka1, Uck2 |
| GO:0036094 | small molecule binding | 8.35e-01 | 9.09e-01 | 2 | 19 | 2392 | 14853 | 10.53% | 16.10% | 0.08% | 0.13% | CL\_0000822\_down | Rps6ka1, Uck2 |
| GO:0004930 | G-protein coupled receptor activity | 8.88e-01 | 9.59e-01 | 1 | 19 | 1618 | 14853 | 5.26% | 10.89% | 0.06% | 0.13% | CL\_0000822\_down | Ccbp2 |
| GO:0003824 | catalytic activity | 9.34e-01 | 9.95e-01 | 4 | 19 | 5097 | 14853 | 21.05% | 34.32% | 0.08% | 0.13% | CL\_0000822\_down | Akr1e1, Rps6ka1, Srm, Uck2 |
| GO:0004888 | transmembrane signaling receptor activity | 9.40e-01 | 9.95e-01 | 1 | 19 | 2046 | 14853 | 5.26% | 13.77% | 0.05% | 0.13% | CL\_0000822\_down | Ccbp2 |
| GO:0038023 | signaling receptor activity | 9.47e-01 | 9.95e-01 | 1 | 19 | 2128 | 14853 | 5.26% | 14.33% | 0.05% | 0.13% | CL\_0000822\_down | Ccbp2 |
| GO:0060089 | molecular transducer activity | 9.62e-01 | 9.95e-01 | 1 | 19 | 2352 | 14853 | 5.26% | 15.84% | 0.04% | 0.13% | CL\_0000822\_down | Ccbp2 |
| GO:0004871 | signal transducer activity | 9.62e-01 | 9.95e-01 | 1 | 19 | 2352 | 14853 | 5.26% | 15.84% | 0.04% | 0.13% | CL\_0000822\_down | Ccbp2 |
| GO:0046872 | metal ion binding | 9.93e-01 | 1.00e+00 | 1 | 19 | 3425 | 14853 | 5.26% | 23.06% | 0.03% | 0.13% | CL\_0000822\_down | Rps6ka1 |
| GO:0043169 | cation binding | 9.94e-01 | 1.00e+00 | 1 | 19 | 3467 | 14853 | 5.26% | 23.34% | 0.03% | 0.13% | CL\_0000822\_down | Rps6ka1 |
| GO:0043167 | ion binding | 9.94e-01 | 1.00e+00 | 1 | 19 | 3479 | 14853 | 5.26% | 23.42% | 0.03% | 0.13% | CL\_0000822\_down | Rps6ka1 |
| GO:0003674 | molecular\_function | 1.00e+00 | 1.00e+00 | 19 | 19 | 14853 | 14853 | 100.00% | 100.00% | 0.13% | 0.13% | CL\_0000822\_down | Akr1e1, Arhgef12, Ccbp2, Fam92a, Hmgn3, Itgb1, Kcnk5, Ly96, Rps6ka1, Sema7a, Sfxn1, Slc20a2, Slc25a4, Slc36a4, Srm, Taf1d, Tfdp1, Tmem18, Uck2 |

### Unannotated IDs

|  |
| --- |
| **CL\_0000822\_down**  MGI:1891441 NA |

|  |  |  |
| --- | --- | --- |
| [close] | **Legend: Edge Types** | (details) |
|  | | |
