# Supplementary material for: Ontology based molecular signatures for immune cell types via gene expression analysis
Source: BMC Bioinformatics. 2013 Aug 30;14:263. doi: 10.1186/1471-2105-14-263 (PMC3844401; doi:10.1186/1471-2105-14-263)
Supplement: Additional file 1 — OBAMS profiles for all mature B cells. Additional file 1 contains a zip archive of OBAMS profiles for all mature B cells, including for each cell type individual spreadsheets showing up and down regulated genes for that cell type relative to parental cell types, and VLAD (GO term enrichment) results for all mature B cells. [file 1471-2105-14-263-S1.zip › Additional File 1/follicular B cell/VLAD.CL_0000843_down/results.html]

# CL\_0000843\_down

|  |  |
| --- | --- |
| Vlad version: | v1.5.1 |
| Date: | Wed Jun 20 07:24:29 2012 |
| Run time: | 61.23 sec |
| Ontology file: | gene\_ontology.obo |
| Ontology date: | Tue Jun 19 19:30:00 2012 |
| Annotation file: | gene\_association.mgi |
| Annotation date: | Wed Jun 6 00:00:00 2012 |
| Analysis type: | enrichment |
| Excluded evidence codes: | ND |
| Number of query sets: | 1 |
| Query set 1: | CL\_0000843\_down (n=14; 0 not found) |
| Universe set: | default (everything) |
| Graph display: | Top 25 scoring terms and their ancestors. Interior nodes have been culled. |

**Jump to:** biological\_process | cellular\_component | molecular\_function | Unannotated id/symbols

### biological\_process (top)

  
  


| TermID | Term | P | Q | k | n | K | N | k/n | K/N | k/K | n/N | Qset | Symbols |
| --- | --- | --- | --- | --- | --- | --- | --- | --- | --- | --- | --- | --- | --- |
| GO:0006826 | iron ion transport | 1.44e-04 | 2.25e-02 | 2 | 12 | 23 | 15137 | 16.67% | 0.15% | 8.70% | 0.08% | CL\_0000843\_down | Lcn2, Ltf |
| GO:0006879 | cellular iron ion homeostasis | 2.65e-04 | 2.25e-02 | 2 | 12 | 31 | 15137 | 16.67% | 0.20% | 6.45% | 0.08% | CL\_0000843\_down | Lcn2, Ltf |
| GO:0009635 | response to herbicide | 7.93e-04 | 2.25e-02 | 1 | 12 | 1 | 15137 | 8.33% | 0.01% | 100.00% | 0.08% | CL\_0000843\_down | Lcn2 |
| GO:0055072 | iron ion homeostasis | 8.66e-04 | 2.25e-02 | 2 | 12 | 56 | 15137 | 16.67% | 0.37% | 3.57% | 0.08% | CL\_0000843\_down | Lcn2, Ltf |
| GO:0006875 | cellular metal ion homeostasis | 1.10e-03 | 2.25e-02 | 3 | 12 | 270 | 15137 | 25.00% | 1.78% | 1.11% | 0.08% | CL\_0000843\_down | Cxcr2, Lcn2, Ltf |
| GO:0030595 | leukocyte chemotaxis | 1.24e-03 | 2.25e-02 | 2 | 12 | 67 | 15137 | 16.67% | 0.44% | 2.99% | 0.08% | CL\_0000843\_down | Cxcr2, S100a9 |
| GO:0055065 | metal ion homeostasis | 1.27e-03 | 2.25e-02 | 3 | 12 | 284 | 15137 | 25.00% | 1.88% | 1.06% | 0.08% | CL\_0000843\_down | Cxcr2, Lcn2, Ltf |
| GO:0000041 | transition metal ion transport | 1.31e-03 | 2.25e-02 | 2 | 12 | 69 | 15137 | 16.67% | 0.46% | 2.90% | 0.08% | CL\_0000843\_down | Lcn2, Ltf |
| GO:0009605 | response to external stimulus | 1.33e-03 | 2.25e-02 | 4 | 12 | 659 | 15137 | 33.33% | 4.35% | 0.61% | 0.08% | CL\_0000843\_down | Bhlhe41, Cxcr2, S100a8, S100a9 |
| GO:0006935 | chemotaxis | 1.51e-03 | 2.25e-02 | 3 | 12 | 302 | 15137 | 25.00% | 2.00% | 0.99% | 0.08% | CL\_0000843\_down | Cxcr2, S100a8, S100a9 |
| GO:0030003 | cellular cation homeostasis | 1.53e-03 | 2.25e-02 | 3 | 12 | 303 | 15137 | 25.00% | 2.00% | 0.99% | 0.08% | CL\_0000843\_down | Cxcr2, Lcn2, Ltf |
| GO:0042330 | taxis | 1.53e-03 | 2.25e-02 | 3 | 12 | 303 | 15137 | 25.00% | 2.00% | 0.99% | 0.08% | CL\_0000843\_down | Cxcr2, S100a8, S100a9 |
| GO:0038112 | interleukin-8-mediated signaling pathway | 1.58e-03 | 2.25e-02 | 1 | 12 | 2 | 15137 | 8.33% | 0.01% | 50.00% | 0.08% | CL\_0000843\_down | Cxcr2 |
| GO:0033212 | iron assimilation | 1.58e-03 | 2.25e-02 | 1 | 12 | 2 | 15137 | 8.33% | 0.01% | 50.00% | 0.08% | CL\_0000843\_down | Lcn2 |
| GO:0033214 | iron assimilation by chelation and transport | 1.58e-03 | 2.25e-02 | 1 | 12 | 2 | 15137 | 8.33% | 0.01% | 50.00% | 0.08% | CL\_0000843\_down | Lcn2 |
| GO:0015688 | iron chelate transport | 1.58e-03 | 2.25e-02 | 1 | 12 | 2 | 15137 | 8.33% | 0.01% | 50.00% | 0.08% | CL\_0000843\_down | Lcn2 |
| GO:0015891 | siderophore transport | 1.58e-03 | 2.25e-02 | 1 | 12 | 2 | 15137 | 8.33% | 0.01% | 50.00% | 0.08% | CL\_0000843\_down | Lcn2 |
| GO:0060326 | cell chemotaxis | 2.22e-03 | 2.97e-02 | 2 | 12 | 90 | 15137 | 16.67% | 0.59% | 2.22% | 0.08% | CL\_0000843\_down | Cxcr2, S100a9 |
| GO:0055080 | cation homeostasis | 2.48e-03 | 3.15e-02 | 3 | 12 | 359 | 15137 | 25.00% | 2.37% | 0.84% | 0.08% | CL\_0000843\_down | Cxcr2, Lcn2, Ltf |
| GO:0050900 | leukocyte migration | 2.73e-03 | 3.29e-02 | 2 | 12 | 100 | 15137 | 16.67% | 0.66% | 2.00% | 0.08% | CL\_0000843\_down | Cxcr2, S100a9 |
| GO:0045113 | regulation of integrin biosynthetic process | 3.17e-03 | 3.64e-02 | 1 | 12 | 4 | 15137 | 8.33% | 0.03% | 25.00% | 0.08% | CL\_0000843\_down | S100a9 |
| GO:0071350 | cellular response to interleukin-15 | 4.75e-03 | 4.97e-02 | 1 | 12 | 6 | 15137 | 8.33% | 0.04% | 16.67% | 0.08% | CL\_0000843\_down | Cxcr2 |
| GO:0010832 | negative regulation of myotube differentiation | 4.75e-03 | 4.97e-02 | 1 | 12 | 6 | 15137 | 8.33% | 0.04% | 16.67% | 0.08% | CL\_0000843\_down | Bhlhe41 |
| GO:0009649 | entrainment of circadian clock | 5.54e-03 | 5.56e-02 | 1 | 12 | 7 | 15137 | 8.33% | 0.05% | 14.29% | 0.08% | CL\_0000843\_down | Bhlhe41 |
| GO:0010944 | negative regulation of transcription by competitive promoter binding | 6.33e-03 | 5.77e-02 | 1 | 12 | 8 | 15137 | 8.33% | 0.05% | 12.50% | 0.08% | CL\_0000843\_down | Bhlhe41 |
| GO:0070672 | response to interleukin-15 | 6.33e-03 | 5.77e-02 | 1 | 12 | 8 | 15137 | 8.33% | 0.05% | 12.50% | 0.08% | CL\_0000843\_down | Cxcr2 |
| GO:0002376 | immune system process | 6.60e-03 | 5.77e-02 | 4 | 12 | 1022 | 15137 | 33.33% | 6.75% | 0.39% | 0.08% | CL\_0000843\_down | Cxcr2, Igj, Lcn2, S100a9 |
| GO:0006873 | cellular ion homeostasis | 6.70e-03 | 5.77e-02 | 3 | 12 | 511 | 15137 | 25.00% | 3.38% | 0.59% | 0.08% | CL\_0000843\_down | Cxcr2, Lcn2, Ltf |
| GO:0055082 | cellular chemical homeostasis | 7.57e-03 | 6.29e-02 | 3 | 12 | 534 | 15137 | 25.00% | 3.53% | 0.56% | 0.08% | CL\_0000843\_down | Cxcr2, Lcn2, Ltf |
| GO:0007010 | cytoskeleton organization | 8.34e-03 | 6.68e-02 | 3 | 12 | 553 | 15137 | 25.00% | 3.65% | 0.54% | 0.08% | CL\_0000843\_down | Actn1, Pstpip2, S100a9 |
| GO:0050801 | ion homeostasis | 8.97e-03 | 6.68e-02 | 3 | 12 | 568 | 15137 | 25.00% | 3.75% | 0.53% | 0.08% | CL\_0000843\_down | Cxcr2, Lcn2, Ltf |
| GO:0042119 | neutrophil activation | 9.48e-03 | 6.68e-02 | 1 | 12 | 12 | 15137 | 8.33% | 0.08% | 8.33% | 0.08% | CL\_0000843\_down | Cxcr2 |
| GO:0006952 | defense response | 1.02e-02 | 6.68e-02 | 3 | 12 | 596 | 15137 | 25.00% | 3.94% | 0.50% | 0.08% | CL\_0000843\_down | Cxcr2, Lcn2, Lyz2 |
| GO:0016998 | cell wall macromolecule catabolic process | 1.03e-02 | 6.68e-02 | 1 | 12 | 13 | 15137 | 8.33% | 0.09% | 7.69% | 0.08% | CL\_0000843\_down | Lyz2 |
| GO:0044036 | cell wall macromolecule metabolic process | 1.03e-02 | 6.68e-02 | 1 | 12 | 13 | 15137 | 8.33% | 0.09% | 7.69% | 0.08% | CL\_0000843\_down | Lyz2 |
| GO:0071554 | cell wall organization or biogenesis | 1.03e-02 | 6.68e-02 | 1 | 12 | 13 | 15137 | 8.33% | 0.09% | 7.69% | 0.08% | CL\_0000843\_down | Lyz2 |
| GO:0032922 | circadian regulation of gene expression | 1.03e-02 | 6.68e-02 | 1 | 12 | 13 | 15137 | 8.33% | 0.09% | 7.69% | 0.08% | CL\_0000843\_down | Bhlhe41 |
| GO:0019725 | cellular homeostasis | 1.09e-02 | 6.83e-02 | 3 | 12 | 610 | 15137 | 25.00% | 4.03% | 0.49% | 0.08% | CL\_0000843\_down | Cxcr2, Lcn2, Ltf |
| GO:0006968 | cellular defense response | 1.10e-02 | 6.83e-02 | 1 | 12 | 14 | 15137 | 8.33% | 0.09% | 7.14% | 0.08% | CL\_0000843\_down | Cxcr2 |
| GO:0010830 | regulation of myotube differentiation | 1.18e-02 | 7.13e-02 | 1 | 12 | 15 | 15137 | 8.33% | 0.10% | 6.67% | 0.08% | CL\_0000843\_down | Bhlhe41 |
| GO:0030574 | collagen catabolic process | 1.34e-02 | 7.51e-02 | 1 | 12 | 17 | 15137 | 8.33% | 0.11% | 5.88% | 0.08% | CL\_0000843\_down | Mmp8 |
| GO:0048041 | focal adhesion assembly | 1.34e-02 | 7.51e-02 | 1 | 12 | 17 | 15137 | 8.33% | 0.11% | 5.88% | 0.08% | CL\_0000843\_down | Actn1 |
| GO:2001015 | negative regulation of skeletal muscle cell differentiation | 1.34e-02 | 7.51e-02 | 1 | 12 | 17 | 15137 | 8.33% | 0.11% | 5.88% | 0.08% | CL\_0000843\_down | Bhlhe41 |
| GO:0070207 | protein homotrimerization | 1.50e-02 | 8.20e-02 | 1 | 12 | 19 | 15137 | 8.33% | 0.13% | 5.26% | 0.08% | CL\_0000843\_down | Lcn2 |
| GO:0044243 | multicellular organismal catabolic process | 1.57e-02 | 8.33e-02 | 1 | 12 | 20 | 15137 | 8.33% | 0.13% | 5.00% | 0.08% | CL\_0000843\_down | Mmp8 |
| GO:0040011 | locomotion | 1.59e-02 | 8.33e-02 | 3 | 12 | 701 | 15137 | 25.00% | 4.63% | 0.43% | 0.08% | CL\_0000843\_down | Cxcr2, S100a8, S100a9 |
| GO:0048878 | chemical homeostasis | 1.73e-02 | 8.34e-02 | 3 | 12 | 723 | 15137 | 25.00% | 4.78% | 0.41% | 0.08% | CL\_0000843\_down | Cxcr2, Lcn2, Ltf |
| GO:0030865 | cortical cytoskeleton organization | 1.73e-02 | 8.34e-02 | 1 | 12 | 22 | 15137 | 8.33% | 0.15% | 4.55% | 0.08% | CL\_0000843\_down | Actn1 |
| GO:0050829 | defense response to Gram-negative bacterium | 1.73e-02 | 8.34e-02 | 1 | 12 | 22 | 15137 | 8.33% | 0.15% | 4.55% | 0.08% | CL\_0000843\_down | Lyz2 |
| GO:0051154 | negative regulation of striated muscle cell differentiation | 1.73e-02 | 8.34e-02 | 1 | 12 | 22 | 15137 | 8.33% | 0.15% | 4.55% | 0.08% | CL\_0000843\_down | Bhlhe41 |
| GO:0030036 | actin cytoskeleton organization | 1.79e-02 | 8.38e-02 | 2 | 12 | 265 | 15137 | 16.67% | 1.75% | 0.75% | 0.08% | CL\_0000843\_down | Actn1, S100a9 |
| GO:0019835 | cytolysis | 1.81e-02 | 8.38e-02 | 1 | 12 | 23 | 15137 | 8.33% | 0.15% | 4.35% | 0.08% | CL\_0000843\_down | Lyz2 |
| GO:0008219 | cell death | 1.85e-02 | 8.42e-02 | 3 | 12 | 742 | 15137 | 25.00% | 4.90% | 0.40% | 0.08% | CL\_0000843\_down | Cxcr2, Lcn2, Lyz2 |
| GO:0016265 | death | 1.91e-02 | 8.53e-02 | 3 | 12 | 751 | 15137 | 25.00% | 4.96% | 0.40% | 0.08% | CL\_0000843\_down | Cxcr2, Lcn2, Lyz2 |
| GO:0006508 | proteolysis | 1.98e-02 | 8.68e-02 | 3 | 12 | 761 | 15137 | 25.00% | 5.03% | 0.39% | 0.08% | CL\_0000843\_down | Cpd, Ltf, Mmp8 |
| GO:0030029 | actin filament-based process | 2.04e-02 | 8.80e-02 | 2 | 12 | 284 | 15137 | 16.67% | 1.88% | 0.70% | 0.08% | CL\_0000843\_down | Actn1, S100a9 |
| GO:0070098 | chemokine-mediated signaling pathway | 2.12e-02 | 8.96e-02 | 1 | 12 | 27 | 15137 | 8.33% | 0.18% | 3.70% | 0.08% | CL\_0000843\_down | Cxcr2 |
| GO:0007044 | cell-substrate junction assembly | 2.20e-02 | 9.13e-02 | 1 | 12 | 28 | 15137 | 8.33% | 0.18% | 3.57% | 0.08% | CL\_0000843\_down | Actn1 |
| GO:0030593 | neutrophil chemotaxis | 2.28e-02 | 9.14e-02 | 1 | 12 | 29 | 15137 | 8.33% | 0.19% | 3.45% | 0.08% | CL\_0000843\_down | Cxcr2 |
| GO:2001014 | regulation of skeletal muscle cell differentiation | 2.28e-02 | 9.14e-02 | 1 | 12 | 29 | 15137 | 8.33% | 0.19% | 3.45% | 0.08% | CL\_0000843\_down | Bhlhe41 |
| GO:0051017 | actin filament bundle assembly | 2.35e-02 | 9.30e-02 | 1 | 12 | 30 | 15137 | 8.33% | 0.20% | 3.33% | 0.08% | CL\_0000843\_down | Actn1 |
| GO:0032963 | collagen metabolic process | 2.43e-02 | 9.45e-02 | 1 | 12 | 31 | 15137 | 8.33% | 0.20% | 3.23% | 0.08% | CL\_0000843\_down | Mmp8 |
| GO:0070301 | cellular response to hydrogen peroxide | 2.59e-02 | 9.74e-02 | 1 | 12 | 33 | 15137 | 8.33% | 0.22% | 3.03% | 0.08% | CL\_0000843\_down | Lcn2 |
| GO:0044259 | multicellular organismal macromolecule metabolic process | 2.59e-02 | 9.74e-02 | 1 | 12 | 33 | 15137 | 8.33% | 0.22% | 3.03% | 0.08% | CL\_0000843\_down | Mmp8 |
| GO:0031532 | actin cytoskeleton reorganization | 2.66e-02 | 9.86e-02 | 1 | 12 | 34 | 15137 | 8.33% | 0.22% | 2.94% | 0.08% | CL\_0000843\_down | S100a9 |
| GO:0070206 | protein trimerization | 2.74e-02 | 9.86e-02 | 1 | 12 | 35 | 15137 | 8.33% | 0.23% | 2.86% | 0.08% | CL\_0000843\_down | Lcn2 |
| GO:0042752 | regulation of circadian rhythm | 2.74e-02 | 9.86e-02 | 1 | 12 | 35 | 15137 | 8.33% | 0.23% | 2.86% | 0.08% | CL\_0000843\_down | Bhlhe41 |
| GO:0044236 | multicellular organismal metabolic process | 2.97e-02 | 1.05e-01 | 1 | 12 | 38 | 15137 | 8.33% | 0.25% | 2.63% | 0.08% | CL\_0000843\_down | Mmp8 |
| GO:0031623 | receptor internalization | 3.05e-02 | 1.07e-01 | 1 | 12 | 39 | 15137 | 8.33% | 0.26% | 2.56% | 0.08% | CL\_0000843\_down | Cxcr2 |
| GO:0051148 | negative regulation of muscle cell differentiation | 3.13e-02 | 1.08e-01 | 1 | 12 | 40 | 15137 | 8.33% | 0.26% | 2.50% | 0.08% | CL\_0000843\_down | Bhlhe41 |
| GO:0048742 | regulation of skeletal muscle fiber development | 3.20e-02 | 1.09e-01 | 1 | 12 | 41 | 15137 | 8.33% | 0.27% | 2.44% | 0.08% | CL\_0000843\_down | Bhlhe41 |
| GO:0007200 | phospholipase C-activating G-protein coupled receptor signaling pathway | 3.66e-02 | 1.23e-01 | 1 | 12 | 47 | 15137 | 8.33% | 0.31% | 2.13% | 0.08% | CL\_0000843\_down | Cxcr2 |
| GO:0050830 | defense response to Gram-positive bacterium | 3.74e-02 | 1.24e-01 | 1 | 12 | 48 | 15137 | 8.33% | 0.32% | 2.08% | 0.08% | CL\_0000843\_down | Lyz2 |
| GO:0034614 | cellular response to reactive oxygen species | 3.82e-02 | 1.24e-01 | 1 | 12 | 49 | 15137 | 8.33% | 0.32% | 2.04% | 0.08% | CL\_0000843\_down | Lcn2 |
| GO:0070887 | cellular response to chemical stimulus | 3.97e-02 | 1.28e-01 | 3 | 12 | 994 | 15137 | 25.00% | 6.57% | 0.30% | 0.08% | CL\_0000843\_down | Cxcr2, Lcn2, S100a9 |
| GO:0042592 | homeostatic process | 4.15e-02 | 1.32e-01 | 3 | 12 | 1011 | 15137 | 25.00% | 6.68% | 0.30% | 0.08% | CL\_0000843\_down | Cxcr2, Lcn2, Ltf |
| GO:0048641 | regulation of skeletal muscle tissue development | 4.35e-02 | 1.34e-01 | 1 | 12 | 56 | 15137 | 8.33% | 0.37% | 1.79% | 0.08% | CL\_0000843\_down | Bhlhe41 |
| GO:0042542 | response to hydrogen peroxide | 4.35e-02 | 1.34e-01 | 1 | 12 | 56 | 15137 | 8.33% | 0.37% | 1.79% | 0.08% | CL\_0000843\_down | Lcn2 |
| GO:0006955 | immune response | 4.61e-02 | 1.39e-01 | 2 | 12 | 441 | 15137 | 16.67% | 2.91% | 0.45% | 0.08% | CL\_0000843\_down | Igj, Lcn2 |
| GO:0034329 | cell junction assembly | 4.66e-02 | 1.39e-01 | 1 | 12 | 60 | 15137 | 8.33% | 0.40% | 1.67% | 0.08% | CL\_0000843\_down | Actn1 |
| GO:0043112 | receptor metabolic process | 4.66e-02 | 1.39e-01 | 1 | 12 | 60 | 15137 | 8.33% | 0.40% | 1.67% | 0.08% | CL\_0000843\_down | Cxcr2 |
| GO:0007623 | circadian rhythm | 4.81e-02 | 1.41e-01 | 1 | 12 | 62 | 15137 | 8.33% | 0.41% | 1.61% | 0.08% | CL\_0000843\_down | Bhlhe41 |
| GO:0009636 | response to toxin | 4.88e-02 | 1.42e-01 | 1 | 12 | 63 | 15137 | 8.33% | 0.42% | 1.59% | 0.08% | CL\_0000843\_down | Lcn2 |
| GO:0030001 | metal ion transport | 5.07e-02 | 1.45e-01 | 2 | 12 | 465 | 15137 | 16.67% | 3.07% | 0.43% | 0.08% | CL\_0000843\_down | Lcn2, Ltf |
| GO:0051707 | response to other organism | 5.13e-02 | 1.45e-01 | 2 | 12 | 468 | 15137 | 16.67% | 3.09% | 0.43% | 0.08% | CL\_0000843\_down | Lcn2, Lyz2 |
| GO:0006959 | humoral immune response | 5.26e-02 | 1.47e-01 | 1 | 12 | 68 | 15137 | 8.33% | 0.45% | 1.47% | 0.08% | CL\_0000843\_down | Igj |
| GO:0016477 | cell migration | 5.35e-02 | 1.48e-01 | 2 | 12 | 479 | 15137 | 16.67% | 3.16% | 0.42% | 0.08% | CL\_0000843\_down | Cxcr2, S100a9 |
| GO:0002274 | myeloid leukocyte activation | 5.41e-02 | 1.48e-01 | 1 | 12 | 70 | 15137 | 8.33% | 0.46% | 1.43% | 0.08% | CL\_0000843\_down | Cxcr2 |
| GO:0006898 | receptor-mediated endocytosis | 5.56e-02 | 1.48e-01 | 1 | 12 | 72 | 15137 | 8.33% | 0.48% | 1.39% | 0.08% | CL\_0000843\_down | Cxcr2 |
| GO:0051153 | regulation of striated muscle cell differentiation | 5.56e-02 | 1.48e-01 | 1 | 12 | 72 | 15137 | 8.33% | 0.48% | 1.39% | 0.08% | CL\_0000843\_down | Bhlhe41 |
| GO:0009607 | response to biotic stimulus | 5.63e-02 | 1.48e-01 | 2 | 12 | 493 | 15137 | 16.67% | 3.26% | 0.41% | 0.08% | CL\_0000843\_down | Lcn2, Lyz2 |
| GO:0007160 | cell-matrix adhesion | 5.64e-02 | 1.48e-01 | 1 | 12 | 73 | 15137 | 8.33% | 0.48% | 1.37% | 0.08% | CL\_0000843\_down | Actn1 |
| GO:0034599 | cellular response to oxidative stress | 6.09e-02 | 1.58e-01 | 1 | 12 | 79 | 15137 | 8.33% | 0.52% | 1.27% | 0.08% | CL\_0000843\_down | Lcn2 |
| GO:0048870 | cell motility | 6.53e-02 | 1.66e-01 | 2 | 12 | 536 | 15137 | 16.67% | 3.54% | 0.37% | 0.08% | CL\_0000843\_down | Cxcr2, S100a9 |
| GO:0051674 | localization of cell | 6.53e-02 | 1.66e-01 | 2 | 12 | 536 | 15137 | 16.67% | 3.54% | 0.37% | 0.08% | CL\_0000843\_down | Cxcr2, S100a9 |
| GO:0000302 | response to reactive oxygen species | 6.61e-02 | 1.66e-01 | 1 | 12 | 86 | 15137 | 8.33% | 0.57% | 1.16% | 0.08% | CL\_0000843\_down | Lcn2 |
| GO:0007015 | actin filament organization | 6.91e-02 | 1.70e-01 | 1 | 12 | 90 | 15137 | 8.33% | 0.59% | 1.11% | 0.08% | CL\_0000843\_down | Actn1 |
| GO:0034330 | cell junction organization | 6.98e-02 | 1.70e-01 | 1 | 12 | 91 | 15137 | 8.33% | 0.60% | 1.10% | 0.08% | CL\_0000843\_down | Actn1 |
| GO:0051147 | regulation of muscle cell differentiation | 6.98e-02 | 1.70e-01 | 1 | 12 | 91 | 15137 | 8.33% | 0.60% | 1.10% | 0.08% | CL\_0000843\_down | Bhlhe41 |
| GO:0042221 | response to chemical stimulus | 7.30e-02 | 1.76e-01 | 4 | 12 | 2101 | 15137 | 33.33% | 13.88% | 0.19% | 0.08% | CL\_0000843\_down | Cxcr2, Lcn2, S100a8, S100a9 |
| GO:0071840 | cellular component organization or biogenesis | 7.47e-02 | 1.77e-01 | 5 | 12 | 3053 | 15137 | 41.67% | 20.17% | 0.16% | 0.08% | CL\_0000843\_down | Actn1, Lcn2, Lyz2, Pstpip2, S100a9 |
| GO:0006812 | cation transport | 7.55e-02 | 1.77e-01 | 2 | 12 | 582 | 15137 | 16.67% | 3.84% | 0.34% | 0.08% | CL\_0000843\_down | Lcn2, Ltf |
| GO:0016202 | regulation of striated muscle tissue development | 7.57e-02 | 1.77e-01 | 1 | 12 | 99 | 15137 | 8.33% | 0.65% | 1.01% | 0.08% | CL\_0000843\_down | Bhlhe41 |
| GO:0048634 | regulation of muscle organ development | 7.65e-02 | 1.77e-01 | 1 | 12 | 100 | 15137 | 8.33% | 0.66% | 1.00% | 0.08% | CL\_0000843\_down | Bhlhe41 |
| GO:0031589 | cell-substrate adhesion | 8.24e-02 | 1.89e-01 | 1 | 12 | 108 | 15137 | 8.33% | 0.71% | 0.93% | 0.08% | CL\_0000843\_down | Actn1 |
| GO:0051271 | negative regulation of cellular component movement | 9.33e-02 | 2.12e-01 | 1 | 12 | 123 | 15137 | 8.33% | 0.81% | 0.81% | 0.08% | CL\_0000843\_down | Actn1 |
| GO:0051704 | multi-organism process | 9.58e-02 | 2.13e-01 | 2 | 12 | 668 | 15137 | 16.67% | 4.41% | 0.30% | 0.08% | CL\_0000843\_down | Lcn2, Lyz2 |
| GO:0006915 | apoptotic process | 9.65e-02 | 2.13e-01 | 2 | 12 | 671 | 15137 | 16.67% | 4.43% | 0.30% | 0.08% | CL\_0000843\_down | Cxcr2, Lcn2 |
| GO:0006928 | cellular component movement | 9.65e-02 | 2.13e-01 | 2 | 12 | 671 | 15137 | 16.67% | 4.43% | 0.30% | 0.08% | CL\_0000843\_down | Cxcr2, S100a9 |
| GO:0012501 | programmed cell death | 9.97e-02 | 2.19e-01 | 2 | 12 | 684 | 15137 | 16.67% | 4.52% | 0.29% | 0.08% | CL\_0000843\_down | Cxcr2, Lcn2 |
| GO:0007204 | elevation of cytosolic calcium ion concentration | 1.03e-01 | 2.22e-01 | 1 | 12 | 136 | 15137 | 8.33% | 0.90% | 0.74% | 0.08% | CL\_0000843\_down | Cxcr2 |
| GO:0009615 | response to virus | 1.03e-01 | 2.22e-01 | 1 | 12 | 137 | 15137 | 8.33% | 0.91% | 0.73% | 0.08% | CL\_0000843\_down | Lcn2 |
| GO:0019221 | cytokine-mediated signaling pathway | 1.11e-01 | 2.36e-01 | 1 | 12 | 147 | 15137 | 8.33% | 0.97% | 0.68% | 0.08% | CL\_0000843\_down | Cxcr2 |
| GO:0031346 | positive regulation of cell projection organization | 1.13e-01 | 2.38e-01 | 1 | 12 | 150 | 15137 | 8.33% | 0.99% | 0.67% | 0.08% | CL\_0000843\_down | Lcn2 |
| GO:0042742 | defense response to bacterium | 1.14e-01 | 2.39e-01 | 1 | 12 | 152 | 15137 | 8.33% | 1.00% | 0.66% | 0.08% | CL\_0000843\_down | Lyz2 |
| GO:0051480 | cytosolic calcium ion homeostasis | 1.16e-01 | 2.40e-01 | 1 | 12 | 154 | 15137 | 8.33% | 1.02% | 0.65% | 0.08% | CL\_0000843\_down | Cxcr2 |
| GO:0048511 | rhythmic process | 1.23e-01 | 2.52e-01 | 1 | 12 | 164 | 15137 | 8.33% | 1.08% | 0.61% | 0.08% | CL\_0000843\_down | Bhlhe41 |
| GO:0006811 | ion transport | 1.24e-01 | 2.53e-01 | 2 | 12 | 778 | 15137 | 16.67% | 5.14% | 0.26% | 0.08% | CL\_0000843\_down | Lcn2, Ltf |
| GO:0006996 | organelle organization | 1.28e-01 | 2.58e-01 | 3 | 12 | 1610 | 15137 | 25.00% | 10.64% | 0.19% | 0.08% | CL\_0000843\_down | Actn1, Pstpip2, S100a9 |
| GO:0042493 | response to drug | 1.30e-01 | 2.60e-01 | 1 | 12 | 174 | 15137 | 8.33% | 1.15% | 0.57% | 0.08% | CL\_0000843\_down | Lcn2 |
| GO:0006979 | response to oxidative stress | 1.39e-01 | 2.76e-01 | 1 | 12 | 187 | 15137 | 8.33% | 1.24% | 0.53% | 0.08% | CL\_0000843\_down | Lcn2 |
| GO:0045087 | innate immune response | 1.43e-01 | 2.82e-01 | 1 | 12 | 193 | 15137 | 8.33% | 1.28% | 0.52% | 0.08% | CL\_0000843\_down | Lcn2 |
| GO:0071345 | cellular response to cytokine stimulus | 1.58e-01 | 3.06e-01 | 1 | 12 | 215 | 15137 | 8.33% | 1.42% | 0.47% | 0.08% | CL\_0000843\_down | Cxcr2 |
| GO:0051260 | protein homooligomerization | 1.58e-01 | 3.06e-01 | 1 | 12 | 215 | 15137 | 8.33% | 1.42% | 0.47% | 0.08% | CL\_0000843\_down | Lcn2 |
| GO:0006950 | response to stress | 1.58e-01 | 3.06e-01 | 3 | 12 | 1775 | 15137 | 25.00% | 11.73% | 0.17% | 0.08% | CL\_0000843\_down | Cxcr2, Lcn2, Lyz2 |
| GO:0006874 | cellular calcium ion homeostasis | 1.65e-01 | 3.15e-01 | 1 | 12 | 225 | 15137 | 8.33% | 1.49% | 0.44% | 0.08% | CL\_0000843\_down | Cxcr2 |
| GO:0055074 | calcium ion homeostasis | 1.71e-01 | 3.24e-01 | 1 | 12 | 234 | 15137 | 8.33% | 1.55% | 0.43% | 0.08% | CL\_0000843\_down | Cxcr2 |
| GO:0010035 | response to inorganic substance | 1.72e-01 | 3.24e-01 | 1 | 12 | 236 | 15137 | 8.33% | 1.56% | 0.42% | 0.08% | CL\_0000843\_down | Lcn2 |
| GO:0072503 | cellular divalent inorganic cation homeostasis | 1.73e-01 | 3.24e-01 | 1 | 12 | 238 | 15137 | 8.33% | 1.57% | 0.42% | 0.08% | CL\_0000843\_down | Cxcr2 |
| GO:0065008 | regulation of biological quality | 1.79e-01 | 3.31e-01 | 3 | 12 | 1876 | 15137 | 25.00% | 12.39% | 0.16% | 0.08% | CL\_0000843\_down | Cxcr2, Lcn2, Ltf |
| GO:0072507 | divalent inorganic cation homeostasis | 1.81e-01 | 3.32e-01 | 1 | 12 | 249 | 15137 | 8.33% | 1.64% | 0.40% | 0.08% | CL\_0000843\_down | Cxcr2 |
| GO:0016043 | cellular component organization | 1.86e-01 | 3.39e-01 | 4 | 12 | 2914 | 15137 | 33.33% | 19.25% | 0.14% | 0.08% | CL\_0000843\_down | Actn1, Lcn2, Pstpip2, S100a9 |
| GO:0006897 | endocytosis | 1.94e-01 | 3.49e-01 | 1 | 12 | 269 | 15137 | 8.33% | 1.78% | 0.37% | 0.08% | CL\_0000843\_down | Cxcr2 |
| GO:0050896 | response to stimulus | 1.95e-01 | 3.49e-01 | 7 | 12 | 6348 | 15137 | 58.33% | 41.94% | 0.11% | 0.08% | CL\_0000843\_down | Bhlhe41, Cxcr2, Igj, Lcn2, Lyz2, S100a8, S100a9 |
| GO:0031344 | regulation of cell projection organization | 1.96e-01 | 3.49e-01 | 1 | 12 | 272 | 15137 | 8.33% | 1.80% | 0.37% | 0.08% | CL\_0000843\_down | Lcn2 |
| GO:0022607 | cellular component assembly | 2.00e-01 | 3.54e-01 | 2 | 12 | 1049 | 15137 | 16.67% | 6.93% | 0.19% | 0.08% | CL\_0000843\_down | Actn1, Lcn2 |
| GO:0043170 | macromolecule metabolic process | 2.01e-01 | 3.54e-01 | 6 | 12 | 5219 | 15137 | 50.00% | 34.48% | 0.11% | 0.08% | CL\_0000843\_down | Bhlhe41, Cpd, Cxcr2, Ltf, Lyz2, Mmp8 |
| GO:0034097 | response to cytokine stimulus | 2.07e-01 | 3.61e-01 | 1 | 12 | 289 | 15137 | 8.33% | 1.91% | 0.35% | 0.08% | CL\_0000843\_down | Cxcr2 |
| GO:0045321 | leukocyte activation | 2.29e-01 | 3.97e-01 | 1 | 12 | 324 | 15137 | 8.33% | 2.14% | 0.31% | 0.08% | CL\_0000843\_down | Cxcr2 |
| GO:0051128 | regulation of cellular component organization | 2.32e-01 | 3.98e-01 | 2 | 12 | 1155 | 15137 | 16.67% | 7.63% | 0.17% | 0.08% | CL\_0000843\_down | Lcn2, S100a9 |
| GO:0009617 | response to bacterium | 2.33e-01 | 3.98e-01 | 1 | 12 | 331 | 15137 | 8.33% | 2.19% | 0.30% | 0.08% | CL\_0000843\_down | Lyz2 |
| GO:0044085 | cellular component biogenesis | 2.42e-01 | 4.08e-01 | 2 | 12 | 1190 | 15137 | 16.67% | 7.86% | 0.17% | 0.08% | CL\_0000843\_down | Actn1, Lcn2 |
| GO:0051259 | protein oligomerization | 2.42e-01 | 4.08e-01 | 1 | 12 | 346 | 15137 | 8.33% | 2.29% | 0.29% | 0.08% | CL\_0000843\_down | Lcn2 |
| GO:0051179 | localization | 2.61e-01 | 4.33e-01 | 4 | 12 | 3328 | 15137 | 33.33% | 21.99% | 0.12% | 0.08% | CL\_0000843\_down | Cxcr2, Lcn2, Ltf, S100a9 |
| GO:0001775 | cell activation | 2.61e-01 | 4.33e-01 | 1 | 12 | 376 | 15137 | 8.33% | 2.48% | 0.27% | 0.08% | CL\_0000843\_down | Cxcr2 |
| GO:0051270 | regulation of cellular component movement | 2.83e-01 | 4.66e-01 | 1 | 12 | 413 | 15137 | 8.33% | 2.73% | 0.24% | 0.08% | CL\_0000843\_down | Actn1 |
| GO:0071842 | cellular component organization at cellular level | 2.86e-01 | 4.69e-01 | 3 | 12 | 2366 | 15137 | 25.00% | 15.63% | 0.13% | 0.08% | CL\_0000843\_down | Actn1, Pstpip2, S100a9 |
| GO:0045596 | negative regulation of cell differentiation | 2.90e-01 | 4.72e-01 | 1 | 12 | 425 | 15137 | 8.33% | 2.81% | 0.24% | 0.08% | CL\_0000843\_down | Bhlhe41 |
| GO:0071841 | cellular component organization or biogenesis at cellular level | 3.15e-01 | 5.09e-01 | 3 | 12 | 2490 | 15137 | 25.00% | 16.45% | 0.12% | 0.08% | CL\_0000843\_down | Actn1, Pstpip2, S100a9 |
| GO:0051130 | positive regulation of cellular component organization | 3.27e-01 | 5.25e-01 | 1 | 12 | 491 | 15137 | 8.33% | 3.24% | 0.20% | 0.08% | CL\_0000843\_down | Lcn2 |
| GO:0060284 | regulation of cell development | 3.40e-01 | 5.43e-01 | 1 | 12 | 515 | 15137 | 8.33% | 3.40% | 0.19% | 0.08% | CL\_0000843\_down | Bhlhe41 |
| GO:0000122 | negative regulation of transcription from RNA polymerase II promoter | 3.48e-01 | 5.51e-01 | 1 | 12 | 529 | 15137 | 8.33% | 3.49% | 0.19% | 0.08% | CL\_0000843\_down | Bhlhe41 |
| GO:0051093 | negative regulation of developmental process | 3.50e-01 | 5.51e-01 | 1 | 12 | 533 | 15137 | 8.33% | 3.52% | 0.19% | 0.08% | CL\_0000843\_down | Bhlhe41 |
| GO:0006810 | transport | 3.57e-01 | 5.55e-01 | 3 | 12 | 2667 | 15137 | 25.00% | 17.62% | 0.11% | 0.08% | CL\_0000843\_down | Cxcr2, Lcn2, Ltf |
| GO:0006461 | protein complex assembly | 3.58e-01 | 5.55e-01 | 1 | 12 | 549 | 15137 | 8.33% | 3.63% | 0.18% | 0.08% | CL\_0000843\_down | Lcn2 |
| GO:0070271 | protein complex biogenesis | 3.59e-01 | 5.55e-01 | 1 | 12 | 551 | 15137 | 8.33% | 3.64% | 0.18% | 0.08% | CL\_0000843\_down | Lcn2 |
| GO:0071822 | protein complex subunit organization | 3.68e-01 | 5.63e-01 | 1 | 12 | 567 | 15137 | 8.33% | 3.75% | 0.18% | 0.08% | CL\_0000843\_down | Lcn2 |
| GO:0051234 | establishment of localization | 3.69e-01 | 5.63e-01 | 3 | 12 | 2719 | 15137 | 25.00% | 17.96% | 0.11% | 0.08% | CL\_0000843\_down | Cxcr2, Lcn2, Ltf |
| GO:0019538 | protein metabolic process | 3.85e-01 | 5.84e-01 | 3 | 12 | 2789 | 15137 | 25.00% | 18.43% | 0.11% | 0.08% | CL\_0000843\_down | Cpd, Ltf, Mmp8 |
| GO:0008284 | positive regulation of cell proliferation | 3.89e-01 | 5.86e-01 | 1 | 12 | 609 | 15137 | 8.33% | 4.02% | 0.16% | 0.08% | CL\_0000843\_down | Cxcr2 |
| GO:0007155 | cell adhesion | 4.00e-01 | 5.99e-01 | 1 | 12 | 631 | 15137 | 8.33% | 4.17% | 0.16% | 0.08% | CL\_0000843\_down | Actn1 |
| GO:0022610 | biological adhesion | 4.03e-01 | 6.00e-01 | 1 | 12 | 637 | 15137 | 8.33% | 4.21% | 0.16% | 0.08% | CL\_0000843\_down | Actn1 |
| GO:0016192 | vesicle-mediated transport | 4.11e-01 | 6.07e-01 | 1 | 12 | 652 | 15137 | 8.33% | 4.31% | 0.15% | 0.08% | CL\_0000843\_down | Cxcr2 |
| GO:0071844 | cellular component assembly at cellular level | 4.23e-01 | 6.22e-01 | 1 | 12 | 678 | 15137 | 8.33% | 4.48% | 0.15% | 0.08% | CL\_0000843\_down | Actn1 |
| GO:0065003 | macromolecular complex assembly | 4.30e-01 | 6.28e-01 | 1 | 12 | 692 | 15137 | 8.33% | 4.57% | 0.14% | 0.08% | CL\_0000843\_down | Lcn2 |
| GO:0043933 | macromolecular complex subunit organization | 4.56e-01 | 6.62e-01 | 1 | 12 | 749 | 15137 | 8.33% | 4.95% | 0.13% | 0.08% | CL\_0000843\_down | Lcn2 |
| GO:0071310 | cellular response to organic substance | 4.62e-01 | 6.67e-01 | 1 | 12 | 762 | 15137 | 8.33% | 5.03% | 0.13% | 0.08% | CL\_0000843\_down | Cxcr2 |
| GO:0033554 | cellular response to stress | 4.67e-01 | 6.69e-01 | 1 | 12 | 772 | 15137 | 8.33% | 5.10% | 0.13% | 0.08% | CL\_0000843\_down | Lcn2 |
| GO:0045892 | negative regulation of transcription, DNA-dependent | 4.77e-01 | 6.80e-01 | 1 | 12 | 795 | 15137 | 8.33% | 5.25% | 0.13% | 0.08% | CL\_0000843\_down | Bhlhe41 |
| GO:0051253 | negative regulation of RNA metabolic process | 4.90e-01 | 6.94e-01 | 1 | 12 | 825 | 15137 | 8.33% | 5.45% | 0.12% | 0.08% | CL\_0000843\_down | Bhlhe41 |
| GO:2000113 | negative regulation of cellular macromolecule biosynthetic process | 5.14e-01 | 7.23e-01 | 1 | 12 | 883 | 15137 | 8.33% | 5.83% | 0.11% | 0.08% | CL\_0000843\_down | Bhlhe41 |
| GO:0045934 | negative regulation of nucleobase-containing compound metabolic process | 5.16e-01 | 7.23e-01 | 1 | 12 | 888 | 15137 | 8.33% | 5.87% | 0.11% | 0.08% | CL\_0000843\_down | Bhlhe41 |
| GO:0051172 | negative regulation of nitrogen compound metabolic process | 5.21e-01 | 7.26e-01 | 1 | 12 | 901 | 15137 | 8.33% | 5.95% | 0.11% | 0.08% | CL\_0000843\_down | Bhlhe41 |
| GO:0010558 | negative regulation of macromolecule biosynthetic process | 5.24e-01 | 7.26e-01 | 1 | 12 | 908 | 15137 | 8.33% | 6.00% | 0.11% | 0.08% | CL\_0000843\_down | Bhlhe41 |
| GO:0010629 | negative regulation of gene expression | 5.36e-01 | 7.38e-01 | 1 | 12 | 937 | 15137 | 8.33% | 6.19% | 0.11% | 0.08% | CL\_0000843\_down | Bhlhe41 |
| GO:0031327 | negative regulation of cellular biosynthetic process | 5.40e-01 | 7.39e-01 | 1 | 12 | 948 | 15137 | 8.33% | 6.26% | 0.11% | 0.08% | CL\_0000843\_down | Bhlhe41 |
| GO:0009890 | negative regulation of biosynthetic process | 5.46e-01 | 7.44e-01 | 1 | 12 | 964 | 15137 | 8.33% | 6.37% | 0.10% | 0.08% | CL\_0000843\_down | Bhlhe41 |
| GO:0045595 | regulation of cell differentiation | 5.56e-01 | 7.53e-01 | 1 | 12 | 990 | 15137 | 8.33% | 6.54% | 0.10% | 0.08% | CL\_0000843\_down | Bhlhe41 |
| GO:0042127 | regulation of cell proliferation | 5.78e-01 | 7.78e-01 | 1 | 12 | 1050 | 15137 | 8.33% | 6.94% | 0.10% | 0.08% | CL\_0000843\_down | Cxcr2 |
| GO:0042981 | regulation of apoptotic process | 5.83e-01 | 7.78e-01 | 1 | 12 | 1065 | 15137 | 8.33% | 7.04% | 0.09% | 0.08% | CL\_0000843\_down | Lcn2 |
| GO:0043067 | regulation of programmed cell death | 5.87e-01 | 7.78e-01 | 1 | 12 | 1075 | 15137 | 8.33% | 7.10% | 0.09% | 0.08% | CL\_0000843\_down | Lcn2 |
| GO:0010628 | positive regulation of gene expression | 5.88e-01 | 7.78e-01 | 1 | 12 | 1078 | 15137 | 8.33% | 7.12% | 0.09% | 0.08% | CL\_0000843\_down | Lcn2 |
| GO:0060255 | regulation of macromolecule metabolic process | 5.93e-01 | 7.78e-01 | 3 | 12 | 3706 | 15137 | 25.00% | 24.48% | 0.08% | 0.08% | CL\_0000843\_down | Bhlhe41, Lcn2, S100a9 |
| GO:2000026 | regulation of multicellular organismal development | 5.94e-01 | 7.78e-01 | 1 | 12 | 1095 | 15137 | 8.33% | 7.23% | 0.09% | 0.08% | CL\_0000843\_down | Bhlhe41 |
| GO:0010941 | regulation of cell death | 6.01e-01 | 7.82e-01 | 1 | 12 | 1114 | 15137 | 8.33% | 7.36% | 0.09% | 0.08% | CL\_0000843\_down | Lcn2 |
| GO:0008152 | metabolic process | 6.08e-01 | 7.88e-01 | 6 | 12 | 7543 | 15137 | 50.00% | 49.83% | 0.08% | 0.08% | CL\_0000843\_down | Bhlhe41, Cpd, Cxcr2, Ltf, Lyz2, Mmp8 |
| GO:0006357 | regulation of transcription from RNA polymerase II promoter | 6.11e-01 | 7.88e-01 | 1 | 12 | 1146 | 15137 | 8.33% | 7.57% | 0.09% | 0.08% | CL\_0000843\_down | Bhlhe41 |
| GO:0048523 | negative regulation of cellular process | 6.26e-01 | 8.03e-01 | 2 | 12 | 2554 | 15137 | 16.67% | 16.87% | 0.08% | 0.08% | CL\_0000843\_down | Actn1, Bhlhe41 |
| GO:0031324 | negative regulation of cellular metabolic process | 6.32e-01 | 8.07e-01 | 1 | 12 | 1211 | 15137 | 8.33% | 8.00% | 0.08% | 0.08% | CL\_0000843\_down | Bhlhe41 |
| GO:0009056 | catabolic process | 6.49e-01 | 8.23e-01 | 1 | 12 | 1264 | 15137 | 8.33% | 8.35% | 0.08% | 0.08% | CL\_0000843\_down | Lyz2 |
| GO:0010605 | negative regulation of macromolecule metabolic process | 6.57e-01 | 8.29e-01 | 1 | 12 | 1290 | 15137 | 8.33% | 8.52% | 0.08% | 0.08% | CL\_0000843\_down | Bhlhe41 |
| GO:0010556 | regulation of macromolecule biosynthetic process | 6.72e-01 | 8.40e-01 | 2 | 12 | 2760 | 15137 | 16.67% | 18.23% | 0.07% | 0.08% | CL\_0000843\_down | Bhlhe41, S100a9 |
| GO:0032879 | regulation of localization | 6.72e-01 | 8.40e-01 | 1 | 12 | 1344 | 15137 | 8.33% | 8.88% | 0.07% | 0.08% | CL\_0000843\_down | Actn1 |
| GO:0010033 | response to organic substance | 6.76e-01 | 8.40e-01 | 1 | 12 | 1358 | 15137 | 8.33% | 8.97% | 0.07% | 0.08% | CL\_0000843\_down | Cxcr2 |
| GO:0009892 | negative regulation of metabolic process | 6.80e-01 | 8.41e-01 | 1 | 12 | 1372 | 15137 | 8.33% | 9.06% | 0.07% | 0.08% | CL\_0000843\_down | Bhlhe41 |
| GO:0050793 | regulation of developmental process | 6.86e-01 | 8.42e-01 | 1 | 12 | 1393 | 15137 | 8.33% | 9.20% | 0.07% | 0.08% | CL\_0000843\_down | Bhlhe41 |
| GO:0048519 | negative regulation of biological process | 6.88e-01 | 8.42e-01 | 2 | 12 | 2839 | 15137 | 16.67% | 18.76% | 0.07% | 0.08% | CL\_0000843\_down | Actn1, Bhlhe41 |
| GO:0048522 | positive regulation of cellular process | 6.96e-01 | 8.47e-01 | 2 | 12 | 2878 | 15137 | 16.67% | 19.01% | 0.07% | 0.08% | CL\_0000843\_down | Cxcr2, Lcn2 |
| GO:0009889 | regulation of biosynthetic process | 7.02e-01 | 8.50e-01 | 2 | 12 | 2905 | 15137 | 16.67% | 19.19% | 0.07% | 0.08% | CL\_0000843\_down | Bhlhe41, S100a9 |
| GO:0010468 | regulation of gene expression | 7.18e-01 | 8.65e-01 | 2 | 12 | 2988 | 15137 | 16.67% | 19.74% | 0.07% | 0.08% | CL\_0000843\_down | Bhlhe41, Lcn2 |
| GO:0019222 | regulation of metabolic process | 7.28e-01 | 8.73e-01 | 3 | 12 | 4423 | 15137 | 25.00% | 29.22% | 0.07% | 0.08% | CL\_0000843\_down | Bhlhe41, Lcn2, S100a9 |
| GO:0010604 | positive regulation of macromolecule metabolic process | 7.50e-01 | 8.93e-01 | 1 | 12 | 1653 | 15137 | 8.33% | 10.92% | 0.06% | 0.08% | CL\_0000843\_down | Lcn2 |
| GO:0048518 | positive regulation of biological process | 7.53e-01 | 8.93e-01 | 2 | 12 | 3183 | 15137 | 16.67% | 21.03% | 0.06% | 0.08% | CL\_0000843\_down | Cxcr2, Lcn2 |
| GO:0006351 | transcription, DNA-dependent | 7.59e-01 | 8.93e-01 | 1 | 12 | 1694 | 15137 | 8.33% | 11.19% | 0.06% | 0.08% | CL\_0000843\_down | Bhlhe41 |
| GO:0032774 | RNA biosynthetic process | 7.60e-01 | 8.93e-01 | 1 | 12 | 1696 | 15137 | 8.33% | 11.20% | 0.06% | 0.08% | CL\_0000843\_down | Bhlhe41 |
| GO:0051239 | regulation of multicellular organismal process | 7.72e-01 | 9.03e-01 | 1 | 12 | 1754 | 15137 | 8.33% | 11.59% | 0.06% | 0.08% | CL\_0000843\_down | Bhlhe41 |
| GO:0009893 | positive regulation of metabolic process | 7.79e-01 | 9.07e-01 | 1 | 12 | 1788 | 15137 | 8.33% | 11.81% | 0.06% | 0.08% | CL\_0000843\_down | Lcn2 |
| GO:0051716 | cellular response to stimulus | 8.13e-01 | 9.38e-01 | 3 | 12 | 4999 | 15137 | 25.00% | 33.03% | 0.06% | 0.08% | CL\_0000843\_down | Cxcr2, Lcn2, S100a9 |
| GO:0007186 | G-protein coupled receptor signaling pathway | 8.13e-01 | 9.38e-01 | 1 | 12 | 1974 | 15137 | 8.33% | 13.04% | 0.05% | 0.08% | CL\_0000843\_down | Cxcr2 |
| GO:0044238 | primary metabolic process | 8.37e-01 | 9.60e-01 | 4 | 12 | 6542 | 15137 | 33.33% | 43.22% | 0.06% | 0.08% | CL\_0000843\_down | Bhlhe41, Cpd, Ltf, Mmp8 |
| GO:0065007 | biological regulation | 8.52e-01 | 9.73e-01 | 6 | 12 | 9171 | 15137 | 50.00% | 60.59% | 0.07% | 0.08% | CL\_0000843\_down | Actn1, Bhlhe41, Cxcr2, Lcn2, Ltf, S100a9 |
| GO:0016070 | RNA metabolic process | 8.56e-01 | 9.73e-01 | 1 | 12 | 2255 | 15137 | 8.33% | 14.90% | 0.04% | 0.08% | CL\_0000843\_down | Bhlhe41 |
| GO:0034645 | cellular macromolecule biosynthetic process | 8.64e-01 | 9.77e-01 | 1 | 12 | 2320 | 15137 | 8.33% | 15.33% | 0.04% | 0.08% | CL\_0000843\_down | Bhlhe41 |
| GO:0009059 | macromolecule biosynthetic process | 8.68e-01 | 9.77e-01 | 1 | 12 | 2346 | 15137 | 8.33% | 15.50% | 0.04% | 0.08% | CL\_0000843\_down | Bhlhe41 |
| GO:0050794 | regulation of cellular process | 8.79e-01 | 9.77e-01 | 5 | 12 | 8223 | 15137 | 41.67% | 54.32% | 0.06% | 0.08% | CL\_0000843\_down | Actn1, Bhlhe41, Cxcr2, Lcn2, S100a9 |
| GO:0006355 | regulation of transcription, DNA-dependent | 8.82e-01 | 9.77e-01 | 1 | 12 | 2472 | 15137 | 8.33% | 16.33% | 0.04% | 0.08% | CL\_0000843\_down | Bhlhe41 |
| GO:2001141 | regulation of RNA biosynthetic process | 8.83e-01 | 9.77e-01 | 1 | 12 | 2476 | 15137 | 8.33% | 16.36% | 0.04% | 0.08% | CL\_0000843\_down | Bhlhe41 |
| GO:0010467 | gene expression | 8.84e-01 | 9.77e-01 | 1 | 12 | 2486 | 15137 | 8.33% | 16.42% | 0.04% | 0.08% | CL\_0000843\_down | Bhlhe41 |
| GO:0051252 | regulation of RNA metabolic process | 8.89e-01 | 9.78e-01 | 1 | 12 | 2532 | 15137 | 8.33% | 16.73% | 0.04% | 0.08% | CL\_0000843\_down | Bhlhe41 |
| GO:0090304 | nucleic acid metabolic process | 9.05e-01 | 9.81e-01 | 1 | 12 | 2692 | 15137 | 8.33% | 17.78% | 0.04% | 0.08% | CL\_0000843\_down | Bhlhe41 |
| GO:2000112 | regulation of cellular macromolecule biosynthetic process | 9.05e-01 | 9.81e-01 | 1 | 12 | 2694 | 15137 | 8.33% | 17.80% | 0.04% | 0.08% | CL\_0000843\_down | Bhlhe41 |
| GO:0031326 | regulation of cellular biosynthetic process | 9.20e-01 | 9.81e-01 | 1 | 12 | 2872 | 15137 | 8.33% | 18.97% | 0.03% | 0.08% | CL\_0000843\_down | Bhlhe41 |
| GO:0019219 | regulation of nucleobase-containing compound metabolic process | 9.22e-01 | 9.81e-01 | 1 | 12 | 2902 | 15137 | 8.33% | 19.17% | 0.03% | 0.08% | CL\_0000843\_down | Bhlhe41 |
| GO:0044260 | cellular macromolecule metabolic process | 9.23e-01 | 9.81e-01 | 2 | 12 | 4660 | 15137 | 16.67% | 30.79% | 0.04% | 0.08% | CL\_0000843\_down | Bhlhe41, Cxcr2 |
| GO:0007166 | cell surface receptor signaling pathway | 9.24e-01 | 9.81e-01 | 1 | 12 | 2927 | 15137 | 8.33% | 19.34% | 0.03% | 0.08% | CL\_0000843\_down | Cxcr2 |
| GO:0051171 | regulation of nitrogen compound metabolic process | 9.25e-01 | 9.81e-01 | 1 | 12 | 2935 | 15137 | 8.33% | 19.39% | 0.03% | 0.08% | CL\_0000843\_down | Bhlhe41 |
| GO:0050789 | regulation of biological process | 9.26e-01 | 9.81e-01 | 5 | 12 | 8805 | 15137 | 41.67% | 58.17% | 0.06% | 0.08% | CL\_0000843\_down | Actn1, Bhlhe41, Cxcr2, Lcn2, S100a9 |
| GO:0044249 | cellular biosynthetic process | 9.28e-01 | 9.81e-01 | 1 | 12 | 2982 | 15137 | 8.33% | 19.70% | 0.03% | 0.08% | CL\_0000843\_down | Bhlhe41 |
| GO:0009058 | biosynthetic process | 9.35e-01 | 9.84e-01 | 1 | 12 | 3082 | 15137 | 8.33% | 20.36% | 0.03% | 0.08% | CL\_0000843\_down | Bhlhe41 |
| GO:0006139 | nucleobase-containing compound metabolic process | 9.43e-01 | 9.88e-01 | 1 | 12 | 3219 | 15137 | 8.33% | 21.27% | 0.03% | 0.08% | CL\_0000843\_down | Bhlhe41 |
| GO:0009987 | cellular process | 9.54e-01 | 9.92e-01 | 8 | 12 | 12456 | 15137 | 66.67% | 82.29% | 0.06% | 0.08% | CL\_0000843\_down | Actn1, Bhlhe41, Cxcr2, Lcn2, Ltf, Lyz2, Pstpip2, S100a9 |
| GO:0034641 | cellular nitrogen compound metabolic process | 9.60e-01 | 9.92e-01 | 1 | 12 | 3557 | 15137 | 8.33% | 23.50% | 0.03% | 0.08% | CL\_0000843\_down | Bhlhe41 |
| GO:0006807 | nitrogen compound metabolic process | 9.64e-01 | 9.92e-01 | 1 | 12 | 3665 | 15137 | 8.33% | 24.21% | 0.03% | 0.08% | CL\_0000843\_down | Bhlhe41 |
| GO:0080090 | regulation of primary metabolic process | 9.66e-01 | 9.92e-01 | 1 | 12 | 3724 | 15137 | 8.33% | 24.60% | 0.03% | 0.08% | CL\_0000843\_down | Bhlhe41 |
| GO:0031323 | regulation of cellular metabolic process | 9.68e-01 | 9.92e-01 | 1 | 12 | 3760 | 15137 | 8.33% | 24.84% | 0.03% | 0.08% | CL\_0000843\_down | Bhlhe41 |
| GO:0007165 | signal transduction | 9.77e-01 | 9.95e-01 | 1 | 12 | 4085 | 15137 | 8.33% | 26.99% | 0.02% | 0.08% | CL\_0000843\_down | Cxcr2 |
| GO:0023052 | signaling | 9.84e-01 | 9.95e-01 | 1 | 12 | 4387 | 15137 | 8.33% | 28.98% | 0.02% | 0.08% | CL\_0000843\_down | Cxcr2 |
| GO:0007154 | cell communication | 9.86e-01 | 9.95e-01 | 1 | 12 | 4503 | 15137 | 8.33% | 29.75% | 0.02% | 0.08% | CL\_0000843\_down | Cxcr2 |
| GO:0044237 | cellular metabolic process | 9.87e-01 | 9.95e-01 | 2 | 12 | 6399 | 15137 | 16.67% | 42.27% | 0.03% | 0.08% | CL\_0000843\_down | Bhlhe41, Cxcr2 |
| GO:0032501 | multicellular organismal process | 9.95e-01 | 9.99e-01 | 1 | 12 | 5391 | 15137 | 8.33% | 35.61% | 0.02% | 0.08% | CL\_0000843\_down | Mmp8 |
| GO:0008150 | biological\_process | 1.00e+00 | 1.00e+00 | 12 | 12 | 15137 | 15137 | 100.00% | 100.00% | 0.08% | 0.08% | CL\_0000843\_down | Actn1, Bhlhe41, Cpd, Cxcr2, Igj, Lcn2, Ltf, Lyz2, Mmp8, Pstpip2, S100a8, S100a9 |

### cellular\_component (top)

  
  


| TermID | Term | P | Q | k | n | K | N | k/n | K/N | k/K | n/N | Qset | Symbols |
| --- | --- | --- | --- | --- | --- | --- | --- | --- | --- | --- | --- | --- | --- |
| GO:0005576 | extracellular region | 3.49e-05 | 4.09e-03 | 8 | 14 | 1812 | 16439 | 57.14% | 11.02% | 0.44% | 0.09% | CL\_0000843\_down | Igj, Lcn2, Ltf, Lyz2, Mmp8, Ngp, S100a8, S100a9 |
| GO:0030141 | secretory granule | 7.92e-04 | 4.63e-02 | 3 | 14 | 222 | 16439 | 21.43% | 1.35% | 1.35% | 0.09% | CL\_0000843\_down | Actn1, Ltf, Lyz2 |
| GO:0032127 | dense core granule membrane | 2.55e-03 | 5.76e-02 | 1 | 14 | 3 | 16439 | 7.14% | 0.02% | 33.33% | 0.09% | CL\_0000843\_down | Actn1 |
| GO:0042629 | mast cell granule | 2.55e-03 | 5.76e-02 | 1 | 14 | 3 | 16439 | 7.14% | 0.02% | 33.33% | 0.09% | CL\_0000843\_down | Cxcr2 |
| GO:0048237 | rough endoplasmic reticulum lumen | 2.55e-03 | 5.76e-02 | 1 | 14 | 3 | 16439 | 7.14% | 0.02% | 33.33% | 0.09% | CL\_0000843\_down | Lyz2 |
| GO:0031410 | cytoplasmic vesicle | 3.05e-03 | 5.76e-02 | 4 | 14 | 755 | 16439 | 28.57% | 4.59% | 0.53% | 0.09% | CL\_0000843\_down | Actn1, Ltf, Lyz2, Ngp |
| GO:0005615 | extracellular space | 3.62e-03 | 5.76e-02 | 4 | 14 | 791 | 16439 | 28.57% | 4.81% | 0.51% | 0.09% | CL\_0000843\_down | Lcn2, Mmp8, S100a8, S100a9 |
| GO:0031982 | vesicle | 3.94e-03 | 5.76e-02 | 4 | 14 | 810 | 16439 | 28.57% | 4.93% | 0.49% | 0.09% | CL\_0000843\_down | Actn1, Ltf, Lyz2, Ngp |
| GO:0000137 | Golgi cis cisterna | 5.10e-03 | 6.63e-02 | 1 | 14 | 6 | 16439 | 7.14% | 0.04% | 16.67% | 0.09% | CL\_0000843\_down | Lyz2 |
| GO:0044421 | extracellular region part | 9.21e-03 | 9.40e-02 | 4 | 14 | 1029 | 16439 | 28.57% | 6.26% | 0.39% | 0.09% | CL\_0000843\_down | Lcn2, Mmp8, S100a8, S100a9 |
| GO:0031045 | dense core granule | 9.33e-03 | 9.40e-02 | 1 | 14 | 11 | 16439 | 7.14% | 0.07% | 9.09% | 0.09% | CL\_0000843\_down | Actn1 |
| GO:0016023 | cytoplasmic membrane-bounded vesicle | 9.64e-03 | 9.40e-02 | 3 | 14 | 537 | 16439 | 21.43% | 3.27% | 0.56% | 0.09% | CL\_0000843\_down | Actn1, Ltf, Lyz2 |
| GO:0005916 | fascia adherens | 1.10e-02 | 9.57e-02 | 1 | 14 | 13 | 16439 | 7.14% | 0.08% | 7.69% | 0.09% | CL\_0000843\_down | Actn1 |
| GO:0031988 | membrane-bounded vesicle | 1.15e-02 | 9.57e-02 | 3 | 14 | 572 | 16439 | 21.43% | 3.48% | 0.52% | 0.09% | CL\_0000843\_down | Actn1, Ltf, Lyz2 |
| GO:0030140 | trans-Golgi network transport vesicle | 1.61e-02 | 1.25e-01 | 1 | 14 | 19 | 16439 | 7.14% | 0.12% | 5.26% | 0.09% | CL\_0000843\_down | Lyz2 |
| GO:0031985 | Golgi cisterna | 2.11e-02 | 1.48e-01 | 1 | 14 | 25 | 16439 | 7.14% | 0.15% | 4.00% | 0.09% | CL\_0000843\_down | Lyz2 |
| GO:0031984 | organelle subcompartment | 2.19e-02 | 1.48e-01 | 1 | 14 | 26 | 16439 | 7.14% | 0.16% | 3.85% | 0.09% | CL\_0000843\_down | Lyz2 |
| GO:0014704 | intercalated disc | 2.28e-02 | 1.48e-01 | 1 | 14 | 27 | 16439 | 7.14% | 0.16% | 3.70% | 0.09% | CL\_0000843\_down | Actn1 |
| GO:0044291 | cell-cell contact zone | 2.44e-02 | 1.50e-01 | 1 | 14 | 29 | 16439 | 7.14% | 0.18% | 3.45% | 0.09% | CL\_0000843\_down | Actn1 |
| GO:0030864 | cortical actin cytoskeleton | 2.78e-02 | 1.62e-01 | 1 | 14 | 33 | 16439 | 7.14% | 0.20% | 3.03% | 0.09% | CL\_0000843\_down | Actn1 |
| GO:0005788 | endoplasmic reticulum lumen | 3.19e-02 | 1.75e-01 | 1 | 14 | 38 | 16439 | 7.14% | 0.23% | 2.63% | 0.09% | CL\_0000843\_down | Lyz2 |
| GO:0000267 | cell fraction | 3.49e-02 | 1.75e-01 | 3 | 14 | 872 | 16439 | 21.43% | 5.30% | 0.34% | 0.09% | CL\_0000843\_down | Cpd, Lyz2, Pstpip2 |
| GO:0005795 | Golgi stack | 3.77e-02 | 1.75e-01 | 1 | 14 | 45 | 16439 | 7.14% | 0.27% | 2.22% | 0.09% | CL\_0000843\_down | Lyz2 |
| GO:0030667 | secretory granule membrane | 3.93e-02 | 1.75e-01 | 1 | 14 | 47 | 16439 | 7.14% | 0.29% | 2.13% | 0.09% | CL\_0000843\_down | Actn1 |
| GO:0005791 | rough endoplasmic reticulum | 4.09e-02 | 1.75e-01 | 1 | 14 | 49 | 16439 | 7.14% | 0.30% | 2.04% | 0.09% | CL\_0000843\_down | Lyz2 |
| GO:0005913 | cell-cell adherens junction | 4.18e-02 | 1.75e-01 | 1 | 14 | 50 | 16439 | 7.14% | 0.30% | 2.00% | 0.09% | CL\_0000843\_down | Actn1 |
| GO:0005798 | Golgi-associated vesicle | 4.34e-02 | 1.75e-01 | 1 | 14 | 52 | 16439 | 7.14% | 0.32% | 1.92% | 0.09% | CL\_0000843\_down | Lyz2 |
| GO:0001725 | stress fiber | 4.42e-02 | 1.75e-01 | 1 | 14 | 53 | 16439 | 7.14% | 0.32% | 1.89% | 0.09% | CL\_0000843\_down | Actn1 |
| GO:0005856 | cytoskeleton | 4.56e-02 | 1.75e-01 | 4 | 14 | 1662 | 16439 | 28.57% | 10.11% | 0.24% | 0.09% | CL\_0000843\_down | Actn1, Pstpip2, S100a8, S100a9 |
| GO:0032432 | actin filament bundle | 4.67e-02 | 1.75e-01 | 1 | 14 | 56 | 16439 | 7.14% | 0.34% | 1.79% | 0.09% | CL\_0000843\_down | Actn1 |
| GO:0017053 | transcriptional repressor complex | 4.67e-02 | 1.75e-01 | 1 | 14 | 56 | 16439 | 7.14% | 0.34% | 1.79% | 0.09% | CL\_0000843\_down | Bhlhe41 |
| GO:0030863 | cortical cytoskeleton | 4.99e-02 | 1.75e-01 | 1 | 14 | 60 | 16439 | 7.14% | 0.36% | 1.67% | 0.09% | CL\_0000843\_down | Actn1 |
| GO:0042641 | actomyosin | 5.15e-02 | 1.75e-01 | 1 | 14 | 62 | 16439 | 7.14% | 0.38% | 1.61% | 0.09% | CL\_0000843\_down | Actn1 |
| GO:0005902 | microvillus | 5.24e-02 | 1.75e-01 | 1 | 14 | 63 | 16439 | 7.14% | 0.38% | 1.59% | 0.09% | CL\_0000843\_down | Lyz2 |
| GO:0030133 | transport vesicle | 5.24e-02 | 1.75e-01 | 1 | 14 | 63 | 16439 | 7.14% | 0.38% | 1.59% | 0.09% | CL\_0000843\_down | Lyz2 |
| GO:0030018 | Z disc | 5.56e-02 | 1.81e-01 | 1 | 14 | 67 | 16439 | 7.14% | 0.41% | 1.49% | 0.09% | CL\_0000843\_down | Actn1 |
| GO:0031674 | I band | 6.52e-02 | 2.06e-01 | 1 | 14 | 79 | 16439 | 7.14% | 0.48% | 1.27% | 0.09% | CL\_0000843\_down | Actn1 |
| GO:0044448 | cell cortex part | 7.72e-02 | 2.26e-01 | 1 | 14 | 94 | 16439 | 7.14% | 0.57% | 1.06% | 0.09% | CL\_0000843\_down | Actn1 |
| GO:0005925 | focal adhesion | 7.72e-02 | 2.26e-01 | 1 | 14 | 94 | 16439 | 7.14% | 0.57% | 1.06% | 0.09% | CL\_0000843\_down | Actn1 |
| GO:0009986 | cell surface | 7.72e-02 | 2.26e-01 | 2 | 14 | 547 | 16439 | 14.29% | 3.33% | 0.37% | 0.09% | CL\_0000843\_down | Cxcr2, Ly6g |
| GO:0005924 | cell-substrate adherens junction | 8.11e-02 | 2.31e-01 | 1 | 14 | 99 | 16439 | 7.14% | 0.60% | 1.01% | 0.09% | CL\_0000843\_down | Actn1 |
| GO:0030055 | cell-substrate junction | 8.58e-02 | 2.35e-01 | 1 | 14 | 105 | 16439 | 7.14% | 0.64% | 0.95% | 0.09% | CL\_0000843\_down | Actn1 |
| GO:0005802 | trans-Golgi network | 8.82e-02 | 2.35e-01 | 1 | 14 | 108 | 16439 | 7.14% | 0.66% | 0.93% | 0.09% | CL\_0000843\_down | Cpd |
| GO:0005624 | membrane fraction | 8.83e-02 | 2.35e-01 | 2 | 14 | 591 | 16439 | 14.29% | 3.60% | 0.34% | 0.09% | CL\_0000843\_down | Cpd, Pstpip2 |
| GO:0030017 | sarcomere | 9.05e-02 | 2.35e-01 | 1 | 14 | 111 | 16439 | 7.14% | 0.68% | 0.90% | 0.09% | CL\_0000843\_down | Actn1 |
| GO:0005626 | insoluble fraction | 9.93e-02 | 2.50e-01 | 2 | 14 | 633 | 16439 | 14.29% | 3.85% | 0.32% | 0.09% | CL\_0000843\_down | Cpd, Pstpip2 |
| GO:0044449 | contractile fiber part | 1.01e-01 | 2.50e-01 | 1 | 14 | 124 | 16439 | 7.14% | 0.75% | 0.81% | 0.09% | CL\_0000843\_down | Actn1 |
| GO:0031225 | anchored to membrane | 1.09e-01 | 2.57e-01 | 1 | 14 | 135 | 16439 | 7.14% | 0.82% | 0.74% | 0.09% | CL\_0000843\_down | Ly6g |
| GO:0030016 | myofibril | 1.09e-01 | 2.57e-01 | 1 | 14 | 135 | 16439 | 7.14% | 0.82% | 0.74% | 0.09% | CL\_0000843\_down | Actn1 |
| GO:0030659 | cytoplasmic vesicle membrane | 1.10e-01 | 2.57e-01 | 1 | 14 | 136 | 16439 | 7.14% | 0.83% | 0.74% | 0.09% | CL\_0000843\_down | Actn1 |
| GO:0044433 | cytoplasmic vesicle part | 1.14e-01 | 2.62e-01 | 1 | 14 | 142 | 16439 | 7.14% | 0.86% | 0.70% | 0.09% | CL\_0000843\_down | Actn1 |
| GO:0043292 | contractile fiber | 1.19e-01 | 2.63e-01 | 1 | 14 | 148 | 16439 | 7.14% | 0.90% | 0.68% | 0.09% | CL\_0000843\_down | Actn1 |
| GO:0005912 | adherens junction | 1.21e-01 | 2.63e-01 | 1 | 14 | 151 | 16439 | 7.14% | 0.92% | 0.66% | 0.09% | CL\_0000843\_down | Actn1 |
| GO:0012506 | vesicle membrane | 1.27e-01 | 2.63e-01 | 1 | 14 | 158 | 16439 | 7.14% | 0.96% | 0.63% | 0.09% | CL\_0000843\_down | Actn1 |
| GO:0030136 | clathrin-coated vesicle | 1.27e-01 | 2.63e-01 | 1 | 14 | 159 | 16439 | 7.14% | 0.97% | 0.63% | 0.09% | CL\_0000843\_down | Lyz2 |
| GO:0043197 | dendritic spine | 1.30e-01 | 2.63e-01 | 1 | 14 | 162 | 16439 | 7.14% | 0.99% | 0.62% | 0.09% | CL\_0000843\_down | Actn1 |
| GO:0044309 | neuron spine | 1.30e-01 | 2.63e-01 | 1 | 14 | 162 | 16439 | 7.14% | 0.99% | 0.62% | 0.09% | CL\_0000843\_down | Actn1 |
| GO:0070161 | anchoring junction | 1.31e-01 | 2.63e-01 | 1 | 14 | 164 | 16439 | 7.14% | 1.00% | 0.61% | 0.09% | CL\_0000843\_down | Actn1 |
| GO:0005938 | cell cortex | 1.33e-01 | 2.63e-01 | 1 | 14 | 166 | 16439 | 7.14% | 1.01% | 0.60% | 0.09% | CL\_0000843\_down | Actn1 |
| GO:0030135 | coated vesicle | 1.46e-01 | 2.84e-01 | 1 | 14 | 184 | 16439 | 7.14% | 1.12% | 0.54% | 0.09% | CL\_0000843\_down | Lyz2 |
| GO:0044444 | cytoplasmic part | 1.49e-01 | 2.87e-01 | 7 | 14 | 5480 | 16439 | 50.00% | 33.34% | 0.13% | 0.09% | CL\_0000843\_down | Actn1, Cxcr2, Lcn2, Ltf, Lyz2, Ngp, Pstpip2 |
| GO:0005792 | microsome | 1.66e-01 | 3.10e-01 | 1 | 14 | 211 | 16439 | 7.14% | 1.28% | 0.47% | 0.09% | CL\_0000843\_down | Cpd |
| GO:0044431 | Golgi apparatus part | 1.70e-01 | 3.10e-01 | 1 | 14 | 217 | 16439 | 7.14% | 1.32% | 0.46% | 0.09% | CL\_0000843\_down | Lyz2 |
| GO:0042598 | vesicular fraction | 1.70e-01 | 3.10e-01 | 1 | 14 | 217 | 16439 | 7.14% | 1.32% | 0.46% | 0.09% | CL\_0000843\_down | Cpd |
| GO:0005886 | plasma membrane | 1.81e-01 | 3.26e-01 | 5 | 14 | 3649 | 16439 | 35.71% | 22.20% | 0.14% | 0.09% | CL\_0000843\_down | Actn1, Cxcr2, Ly6g, S100a8, S100a9 |
| GO:0005829 | cytosol | 1.87e-01 | 3.31e-01 | 2 | 14 | 934 | 16439 | 14.29% | 5.68% | 0.21% | 0.09% | CL\_0000843\_down | Lcn2, Pstpip2 |
| GO:0016323 | basolateral plasma membrane | 1.94e-01 | 3.31e-01 | 1 | 14 | 251 | 16439 | 7.14% | 1.53% | 0.40% | 0.09% | CL\_0000843\_down | Actn1 |
| GO:0071944 | cell periphery | 1.95e-01 | 3.31e-01 | 5 | 14 | 3737 | 16439 | 35.71% | 22.73% | 0.13% | 0.09% | CL\_0000843\_down | Actn1, Cxcr2, Ly6g, S100a8, S100a9 |
| GO:0009897 | external side of plasma membrane | 1.95e-01 | 3.31e-01 | 1 | 14 | 253 | 16439 | 7.14% | 1.54% | 0.40% | 0.09% | CL\_0000843\_down | Ly6g |
| GO:0005764 | lysosome | 2.04e-01 | 3.34e-01 | 1 | 14 | 265 | 16439 | 7.14% | 1.61% | 0.38% | 0.09% | CL\_0000843\_down | Cxcr2 |
| GO:0000323 | lytic vacuole | 2.04e-01 | 3.34e-01 | 1 | 14 | 265 | 16439 | 7.14% | 1.61% | 0.38% | 0.09% | CL\_0000843\_down | Cxcr2 |
| GO:0044432 | endoplasmic reticulum part | 2.10e-01 | 3.34e-01 | 1 | 14 | 274 | 16439 | 7.14% | 1.67% | 0.36% | 0.09% | CL\_0000843\_down | Lyz2 |
| GO:0005911 | cell-cell junction | 2.11e-01 | 3.34e-01 | 1 | 14 | 276 | 16439 | 7.14% | 1.68% | 0.36% | 0.09% | CL\_0000843\_down | Actn1 |
| GO:0043232 | intracellular non-membrane-bounded organelle | 2.14e-01 | 3.34e-01 | 4 | 14 | 2851 | 16439 | 28.57% | 17.34% | 0.14% | 0.09% | CL\_0000843\_down | Actn1, Pstpip2, S100a8, S100a9 |
| GO:0043228 | non-membrane-bounded organelle | 2.14e-01 | 3.34e-01 | 4 | 14 | 2851 | 16439 | 28.57% | 17.34% | 0.14% | 0.09% | CL\_0000843\_down | Actn1, Pstpip2, S100a8, S100a9 |
| GO:0005737 | cytoplasm | 2.27e-01 | 3.50e-01 | 9 | 14 | 8331 | 16439 | 64.29% | 50.68% | 0.11% | 0.09% | CL\_0000843\_down | Actn1, Cxcr2, Lcn2, Ltf, Lyz2, Ngp, Pstpip2, S100a8, S100a9 |
| GO:0005773 | vacuole | 2.34e-01 | 3.56e-01 | 1 | 14 | 310 | 16439 | 7.14% | 1.89% | 0.32% | 0.09% | CL\_0000843\_down | Cxcr2 |
| GO:0043229 | intracellular organelle | 2.39e-01 | 3.56e-01 | 10 | 14 | 9595 | 16439 | 71.43% | 58.37% | 0.10% | 0.09% | CL\_0000843\_down | Actn1, Bhlhe41, Cpd, Cxcr2, Ltf, Lyz2, Ngp, Pstpip2, S100a8, S100a9 |
| GO:0043226 | organelle | 2.42e-01 | 3.56e-01 | 10 | 14 | 9616 | 16439 | 71.43% | 58.50% | 0.10% | 0.09% | CL\_0000843\_down | Actn1, Bhlhe41, Cpd, Cxcr2, Ltf, Lyz2, Ngp, Pstpip2, S100a8, S100a9 |
| GO:0015629 | actin cytoskeleton | 2.43e-01 | 3.56e-01 | 1 | 14 | 324 | 16439 | 7.14% | 1.97% | 0.31% | 0.09% | CL\_0000843\_down | Actn1 |
| GO:0005578 | proteinaceous extracellular matrix | 2.48e-01 | 3.56e-01 | 1 | 14 | 331 | 16439 | 7.14% | 2.01% | 0.30% | 0.09% | CL\_0000843\_down | Mmp8 |
| GO:0005625 | soluble fraction | 2.49e-01 | 3.56e-01 | 1 | 14 | 333 | 16439 | 7.14% | 2.03% | 0.30% | 0.09% | CL\_0000843\_down | Lyz2 |
| GO:0044424 | intracellular part | 2.74e-01 | 3.83e-01 | 11 | 14 | 11042 | 16439 | 78.57% | 67.17% | 0.10% | 0.09% | CL\_0000843\_down | Actn1, Bhlhe41, Cpd, Cxcr2, Lcn2, Ltf, Lyz2, Ngp, Pstpip2, S100a8, S100a9 |
| GO:0031012 | extracellular matrix | 2.75e-01 | 3.83e-01 | 1 | 14 | 373 | 16439 | 7.14% | 2.27% | 0.27% | 0.09% | CL\_0000843\_down | Mmp8 |
| GO:0030425 | dendrite | 2.85e-01 | 3.92e-01 | 1 | 14 | 389 | 16439 | 7.14% | 2.37% | 0.26% | 0.09% | CL\_0000843\_down | Actn1 |
| GO:0042995 | cell projection | 3.02e-01 | 4.11e-01 | 2 | 14 | 1292 | 16439 | 14.29% | 7.86% | 0.15% | 0.09% | CL\_0000843\_down | Actn1, Lyz2 |
| GO:0005622 | intracellular | 3.14e-01 | 4.23e-01 | 11 | 14 | 11282 | 16439 | 78.57% | 68.63% | 0.10% | 0.09% | CL\_0000843\_down | Actn1, Bhlhe41, Cpd, Cxcr2, Lcn2, Ltf, Lyz2, Ngp, Pstpip2, S100a8, S100a9 |
| GO:0044463 | cell projection part | 3.91e-01 | 5.16e-01 | 1 | 14 | 572 | 16439 | 7.14% | 3.48% | 0.17% | 0.09% | CL\_0000843\_down | Actn1 |
| GO:0044459 | plasma membrane part | 3.96e-01 | 5.16e-01 | 2 | 14 | 1582 | 16439 | 14.29% | 9.62% | 0.13% | 0.09% | CL\_0000843\_down | Actn1, Ly6g |
| GO:0070013 | intracellular organelle lumen | 3.99e-01 | 5.16e-01 | 2 | 14 | 1593 | 16439 | 14.29% | 9.69% | 0.13% | 0.09% | CL\_0000843\_down | Bhlhe41, Lyz2 |
| GO:0043233 | organelle lumen | 4.01e-01 | 5.16e-01 | 2 | 14 | 1598 | 16439 | 14.29% | 9.72% | 0.13% | 0.09% | CL\_0000843\_down | Bhlhe41, Lyz2 |
| GO:0031974 | membrane-enclosed lumen | 4.14e-01 | 5.26e-01 | 2 | 14 | 1639 | 16439 | 14.29% | 9.97% | 0.12% | 0.09% | CL\_0000843\_down | Bhlhe41, Lyz2 |
| GO:0044464 | cell part | 4.53e-01 | 5.61e-01 | 12 | 14 | 13174 | 16439 | 85.71% | 80.14% | 0.09% | 0.09% | CL\_0000843\_down | Actn1, Bhlhe41, Cpd, Cxcr2, Lcn2, Ltf, Ly6g, Lyz2, Ngp, Pstpip2, S100a8, S100a9 |
| GO:0005623 | cell | 4.53e-01 | 5.61e-01 | 12 | 14 | 13175 | 16439 | 85.71% | 80.14% | 0.09% | 0.09% | CL\_0000843\_down | Actn1, Bhlhe41, Cpd, Cxcr2, Lcn2, Ltf, Ly6g, Lyz2, Ngp, Pstpip2, S100a8, S100a9 |
| GO:0043005 | neuron projection | 4.56e-01 | 5.61e-01 | 1 | 14 | 699 | 16439 | 7.14% | 4.25% | 0.14% | 0.09% | CL\_0000843\_down | Actn1 |
| GO:0030054 | cell junction | 4.62e-01 | 5.63e-01 | 1 | 14 | 711 | 16439 | 7.14% | 4.33% | 0.14% | 0.09% | CL\_0000843\_down | Actn1 |
| GO:0044451 | nucleoplasm part | 4.72e-01 | 5.64e-01 | 1 | 14 | 732 | 16439 | 7.14% | 4.45% | 0.14% | 0.09% | CL\_0000843\_down | Bhlhe41 |
| GO:0012505 | endomembrane system | 4.73e-01 | 5.64e-01 | 1 | 14 | 734 | 16439 | 7.14% | 4.46% | 0.14% | 0.09% | CL\_0000843\_down | Actn1 |
| GO:0005654 | nucleoplasm | 5.13e-01 | 6.06e-01 | 1 | 14 | 823 | 16439 | 7.14% | 5.01% | 0.12% | 0.09% | CL\_0000843\_down | Bhlhe41 |
| GO:0005794 | Golgi apparatus | 5.78e-01 | 6.76e-01 | 1 | 14 | 983 | 16439 | 7.14% | 5.98% | 0.10% | 0.09% | CL\_0000843\_down | Lyz2 |
| GO:0016020 | membrane | 5.83e-01 | 6.76e-01 | 7 | 14 | 8099 | 16439 | 50.00% | 49.27% | 0.09% | 0.09% | CL\_0000843\_down | Actn1, Cpd, Cxcr2, Ly6g, Pstpip2, S100a8, S100a9 |
| GO:0005783 | endoplasmic reticulum | 6.18e-01 | 7.09e-01 | 1 | 14 | 1093 | 16439 | 7.14% | 6.65% | 0.09% | 0.09% | CL\_0000843\_down | Lyz2 |
| GO:0044430 | cytoskeletal part | 6.29e-01 | 7.15e-01 | 1 | 14 | 1124 | 16439 | 7.14% | 6.84% | 0.09% | 0.09% | CL\_0000843\_down | Actn1 |
| GO:0031090 | organelle membrane | 6.36e-01 | 7.15e-01 | 1 | 14 | 1143 | 16439 | 7.14% | 6.95% | 0.09% | 0.09% | CL\_0000843\_down | Actn1 |
| GO:0043231 | intracellular membrane-bounded organelle | 6.50e-01 | 7.22e-01 | 7 | 14 | 8480 | 16439 | 50.00% | 51.58% | 0.08% | 0.09% | CL\_0000843\_down | Actn1, Bhlhe41, Cpd, Cxcr2, Ltf, Lyz2, S100a9 |
| GO:0043227 | membrane-bounded organelle | 6.54e-01 | 7.22e-01 | 7 | 14 | 8499 | 16439 | 50.00% | 51.70% | 0.08% | 0.09% | CL\_0000843\_down | Actn1, Bhlhe41, Cpd, Cxcr2, Ltf, Lyz2, S100a9 |
| GO:0005634 | nucleus | 6.93e-01 | 7.58e-01 | 4 | 14 | 5200 | 16439 | 28.57% | 31.63% | 0.08% | 0.09% | CL\_0000843\_down | Actn1, Bhlhe41, Cpd, S100a9 |
| GO:0031981 | nuclear lumen | 7.13e-01 | 7.72e-01 | 1 | 14 | 1402 | 16439 | 7.14% | 8.53% | 0.07% | 0.09% | CL\_0000843\_down | Bhlhe41 |
| GO:0044446 | intracellular organelle part | 7.28e-01 | 7.81e-01 | 3 | 14 | 4160 | 16439 | 21.43% | 25.31% | 0.07% | 0.09% | CL\_0000843\_down | Actn1, Bhlhe41, Lyz2 |
| GO:0044422 | organelle part | 7.43e-01 | 7.91e-01 | 3 | 14 | 4252 | 16439 | 21.43% | 25.87% | 0.07% | 0.09% | CL\_0000843\_down | Actn1, Bhlhe41, Lyz2 |
| GO:0044428 | nuclear part | 7.96e-01 | 8.39e-01 | 1 | 14 | 1765 | 16439 | 7.14% | 10.74% | 0.06% | 0.09% | CL\_0000843\_down | Bhlhe41 |
| GO:0044425 | membrane part | 8.72e-01 | 9.11e-01 | 4 | 14 | 6537 | 16439 | 28.57% | 39.77% | 0.06% | 0.09% | CL\_0000843\_down | Actn1, Cpd, Cxcr2, Ly6g |
| GO:0031224 | intrinsic to membrane | 9.21e-01 | 9.54e-01 | 3 | 14 | 5824 | 16439 | 21.43% | 35.43% | 0.05% | 0.09% | CL\_0000843\_down | Cpd, Cxcr2, Ly6g |
| GO:0043234 | protein complex | 9.37e-01 | 9.62e-01 | 1 | 14 | 2944 | 16439 | 7.14% | 17.91% | 0.03% | 0.09% | CL\_0000843\_down | Bhlhe41 |
| GO:0032991 | macromolecular complex | 9.67e-01 | 9.84e-01 | 1 | 14 | 3562 | 16439 | 7.14% | 21.67% | 0.03% | 0.09% | CL\_0000843\_down | Bhlhe41 |
| GO:0016021 | integral to membrane | 9.78e-01 | 9.87e-01 | 2 | 14 | 5690 | 16439 | 14.29% | 34.61% | 0.04% | 0.09% | CL\_0000843\_down | Cpd, Cxcr2 |
| GO:0005575 | cellular\_component | 1.00e+00 | 1.00e+00 | 14 | 14 | 16439 | 16439 | 100.00% | 100.00% | 0.09% | 0.09% | CL\_0000843\_down | Actn1, Bhlhe41, Cpd, Cxcr2, Igj, Lcn2, Ltf, Ly6g, Lyz2, Mmp8, Ngp, Pstpip2, S100a8, S100a9 |

### molecular\_function (top)

  
  


| TermID | Term | P | Q | k | n | K | N | k/n | K/N | k/K | n/N | Qset | Symbols |
| --- | --- | --- | --- | --- | --- | --- | --- | --- | --- | --- | --- | --- | --- |
| GO:0008236 | serine-type peptidase activity | 5.13e-04 | 1.99e-02 | 3 | 12 | 204 | 14853 | 25.00% | 1.37% | 1.47% | 0.08% | CL\_0000843\_down | Cpd, Ltf, Mmp8 |
| GO:0017171 | serine hydrolase activity | 5.35e-04 | 1.99e-02 | 3 | 12 | 207 | 14853 | 25.00% | 1.39% | 1.45% | 0.08% | CL\_0000843\_down | Cpd, Ltf, Mmp8 |
| GO:0005509 | calcium ion binding | 5.79e-04 | 1.99e-02 | 4 | 12 | 518 | 14853 | 33.33% | 3.49% | 0.77% | 0.08% | CL\_0000843\_down | Actn1, Mmp8, S100a8, S100a9 |
| GO:0004918 | interleukin-8 receptor activity | 1.62e-03 | 4.16e-02 | 1 | 12 | 2 | 14853 | 8.33% | 0.01% | 50.00% | 0.08% | CL\_0000843\_down | Cxcr2 |
| GO:0019958 | C-X-C chemokine binding | 2.42e-03 | 4.16e-02 | 1 | 12 | 3 | 14853 | 8.33% | 0.02% | 33.33% | 0.08% | CL\_0000843\_down | Cxcr2 |
| GO:0019959 | interleukin-8 binding | 2.42e-03 | 4.16e-02 | 1 | 12 | 3 | 14853 | 8.33% | 0.02% | 33.33% | 0.08% | CL\_0000843\_down | Cxcr2 |
| GO:0004185 | serine-type carboxypeptidase activity | 3.23e-03 | 4.75e-02 | 1 | 12 | 4 | 14853 | 8.33% | 0.03% | 25.00% | 0.08% | CL\_0000843\_down | Cpd |
| GO:0043426 | MRF binding | 4.03e-03 | 4.84e-02 | 1 | 12 | 5 | 14853 | 8.33% | 0.03% | 20.00% | 0.08% | CL\_0000843\_down | Bhlhe41 |
| GO:0016494 | C-X-C chemokine receptor activity | 6.45e-03 | 4.84e-02 | 1 | 12 | 8 | 14853 | 8.33% | 0.05% | 12.50% | 0.08% | CL\_0000843\_down | Cxcr2 |
| GO:0003796 | lysozyme activity | 7.25e-03 | 4.84e-02 | 1 | 12 | 9 | 14853 | 8.33% | 0.06% | 11.11% | 0.08% | CL\_0000843\_down | Lyz2 |
| GO:0070008 | serine-type exopeptidase activity | 7.25e-03 | 4.84e-02 | 1 | 12 | 9 | 14853 | 8.33% | 0.06% | 11.11% | 0.08% | CL\_0000843\_down | Cpd |
| GO:0008199 | ferric iron binding | 8.05e-03 | 4.84e-02 | 1 | 12 | 10 | 14853 | 8.33% | 0.07% | 10.00% | 0.08% | CL\_0000843\_down | Ltf |
| GO:0017166 | vinculin binding | 8.05e-03 | 4.84e-02 | 1 | 12 | 10 | 14853 | 8.33% | 0.07% | 10.00% | 0.08% | CL\_0000843\_down | Actn1 |
| GO:0008237 | metallopeptidase activity | 8.15e-03 | 4.84e-02 | 2 | 12 | 172 | 14853 | 16.67% | 1.16% | 1.16% | 0.08% | CL\_0000843\_down | Cpd, Mmp8 |
| GO:0046872 | metal ion binding | 8.97e-03 | 4.84e-02 | 7 | 12 | 3425 | 14853 | 58.33% | 23.06% | 0.20% | 0.08% | CL\_0000843\_down | Actn1, Cpd, Lcn2, Ltf, Mmp8, S100a8, S100a9 |
| GO:0042803 | protein homodimerization activity | 9.05e-03 | 4.84e-02 | 3 | 12 | 559 | 14853 | 25.00% | 3.76% | 0.54% | 0.08% | CL\_0000843\_down | Actn1, Bhlhe41, Lcn2 |
| GO:0070011 | peptidase activity, acting on L-amino acid peptides | 9.54e-03 | 4.84e-02 | 3 | 12 | 570 | 14853 | 25.00% | 3.84% | 0.53% | 0.08% | CL\_0000843\_down | Cpd, Ltf, Mmp8 |
| GO:0043169 | cation binding | 9.62e-03 | 4.84e-02 | 7 | 12 | 3467 | 14853 | 58.33% | 23.34% | 0.20% | 0.08% | CL\_0000843\_down | Actn1, Cpd, Lcn2, Ltf, Mmp8, S100a8, S100a9 |
| GO:0019956 | chemokine binding | 9.66e-03 | 4.84e-02 | 1 | 12 | 12 | 14853 | 8.33% | 0.08% | 8.33% | 0.08% | CL\_0000843\_down | Cxcr2 |
| GO:0043167 | ion binding | 9.81e-03 | 4.84e-02 | 7 | 12 | 3479 | 14853 | 58.33% | 23.42% | 0.20% | 0.08% | CL\_0000843\_down | Actn1, Cpd, Lcn2, Ltf, Mmp8, S100a8, S100a9 |
| GO:0005506 | iron ion binding | 9.87e-03 | 4.84e-02 | 2 | 12 | 190 | 14853 | 16.67% | 1.28% | 1.05% | 0.08% | CL\_0000843\_down | Lcn2, Ltf |
| GO:0008233 | peptidase activity | 1.09e-02 | 5.12e-02 | 3 | 12 | 599 | 14853 | 25.00% | 4.03% | 0.50% | 0.08% | CL\_0000843\_down | Cpd, Ltf, Mmp8 |
| GO:0004181 | metallocarboxypeptidase activity | 1.68e-02 | 6.60e-02 | 1 | 12 | 21 | 14853 | 8.33% | 0.14% | 4.76% | 0.08% | CL\_0000843\_down | Cpd |
| GO:0001191 | RNA polymerase II transcription factor binding transcription factor activity involved in negative regulation of transcription | 1.76e-02 | 6.60e-02 | 1 | 12 | 22 | 14853 | 8.33% | 0.15% | 4.55% | 0.08% | CL\_0000843\_down | Bhlhe41 |
| GO:0001637 | G-protein coupled chemoattractant receptor activity | 1.84e-02 | 6.60e-02 | 1 | 12 | 23 | 14853 | 8.33% | 0.15% | 4.35% | 0.08% | CL\_0000843\_down | Cxcr2 |
| GO:0004950 | chemokine receptor activity | 1.84e-02 | 6.60e-02 | 1 | 12 | 23 | 14853 | 8.33% | 0.15% | 4.35% | 0.08% | CL\_0000843\_down | Cxcr2 |
| GO:0070888 | E-box binding | 1.92e-02 | 6.60e-02 | 1 | 12 | 24 | 14853 | 8.33% | 0.16% | 4.17% | 0.08% | CL\_0000843\_down | Bhlhe41 |
| GO:0001102 | RNA polymerase II activating transcription factor binding | 1.92e-02 | 6.60e-02 | 1 | 12 | 24 | 14853 | 8.33% | 0.16% | 4.17% | 0.08% | CL\_0000843\_down | Bhlhe41 |
| GO:0043425 | bHLH transcription factor binding | 1.92e-02 | 6.60e-02 | 1 | 12 | 24 | 14853 | 8.33% | 0.16% | 4.17% | 0.08% | CL\_0000843\_down | Bhlhe41 |
| GO:0044325 | ion channel binding | 1.92e-02 | 6.60e-02 | 1 | 12 | 24 | 14853 | 8.33% | 0.16% | 4.17% | 0.08% | CL\_0000843\_down | Actn1 |
| GO:0000978 | RNA polymerase II core promoter proximal region sequence-specific DNA binding | 2.08e-02 | 6.92e-02 | 1 | 12 | 26 | 14853 | 8.33% | 0.18% | 3.85% | 0.08% | CL\_0000843\_down | Bhlhe41 |
| GO:0001078 | RNA polymerase II core promoter proximal region sequence-specific DNA binding transcription factor activity involved in negative regulation of transcription | 2.48e-02 | 7.55e-02 | 1 | 12 | 31 | 14853 | 8.33% | 0.21% | 3.23% | 0.08% | CL\_0000843\_down | Bhlhe41 |
| GO:0001227 | RNA polymerase II transcription regulatory region sequence-specific DNA binding transcription factor activity involved in negative regulation of transcription | 2.48e-02 | 7.55e-02 | 1 | 12 | 31 | 14853 | 8.33% | 0.21% | 3.23% | 0.08% | CL\_0000843\_down | Bhlhe41 |
| GO:0046983 | protein dimerization activity | 2.58e-02 | 7.55e-02 | 3 | 12 | 826 | 14853 | 25.00% | 5.56% | 0.36% | 0.08% | CL\_0000843\_down | Actn1, Bhlhe41, Lcn2 |
| GO:0042802 | identical protein binding | 2.61e-02 | 7.55e-02 | 3 | 12 | 829 | 14853 | 25.00% | 5.58% | 0.36% | 0.08% | CL\_0000843\_down | Actn1, Bhlhe41, Lcn2 |
| GO:0003779 | actin binding | 2.65e-02 | 7.55e-02 | 2 | 12 | 320 | 14853 | 16.67% | 2.15% | 0.62% | 0.08% | CL\_0000843\_down | Actn1, Pstpip2 |
| GO:0008235 | metalloexopeptidase activity | 2.71e-02 | 7.55e-02 | 1 | 12 | 34 | 14853 | 8.33% | 0.23% | 2.94% | 0.08% | CL\_0000843\_down | Cpd |
| GO:0004180 | carboxypeptidase activity | 2.79e-02 | 7.57e-02 | 1 | 12 | 35 | 14853 | 8.33% | 0.24% | 2.86% | 0.08% | CL\_0000843\_down | Cpd |
| GO:0000987 | core promoter proximal region sequence-specific DNA binding | 3.03e-02 | 8.00e-02 | 1 | 12 | 38 | 14853 | 8.33% | 0.26% | 2.63% | 0.08% | CL\_0000843\_down | Bhlhe41 |
| GO:0046914 | transition metal ion binding | 3.26e-02 | 8.20e-02 | 4 | 12 | 1599 | 14853 | 33.33% | 10.77% | 0.25% | 0.08% | CL\_0000843\_down | Cpd, Lcn2, Ltf, Mmp8 |
| GO:0001159 | core promoter proximal region DNA binding | 3.26e-02 | 8.20e-02 | 1 | 12 | 41 | 14853 | 8.33% | 0.28% | 2.44% | 0.08% | CL\_0000843\_down | Bhlhe41 |
| GO:0033613 | activating transcription factor binding | 3.50e-02 | 8.57e-02 | 1 | 12 | 44 | 14853 | 8.33% | 0.30% | 2.27% | 0.08% | CL\_0000843\_down | Bhlhe41 |
| GO:0003823 | antigen binding | 3.58e-02 | 8.57e-02 | 1 | 12 | 45 | 14853 | 8.33% | 0.30% | 2.22% | 0.08% | CL\_0000843\_down | Igj |
| GO:0019955 | cytokine binding | 4.51e-02 | 1.06e-01 | 1 | 12 | 57 | 14853 | 8.33% | 0.38% | 1.75% | 0.08% | CL\_0000843\_down | Cxcr2 |
| GO:0001085 | RNA polymerase II transcription factor binding | 4.82e-02 | 1.10e-01 | 1 | 12 | 61 | 14853 | 8.33% | 0.41% | 1.64% | 0.08% | CL\_0000843\_down | Bhlhe41 |
| GO:0042826 | histone deacetylase binding | 5.05e-02 | 1.11e-01 | 1 | 12 | 64 | 14853 | 8.33% | 0.43% | 1.56% | 0.08% | CL\_0000843\_down | Bhlhe41 |
| GO:0002020 | protease binding | 5.05e-02 | 1.11e-01 | 1 | 12 | 64 | 14853 | 8.33% | 0.43% | 1.56% | 0.08% | CL\_0000843\_down | Lcn2 |
| GO:0051015 | actin filament binding | 5.28e-02 | 1.13e-01 | 1 | 12 | 67 | 14853 | 8.33% | 0.45% | 1.49% | 0.08% | CL\_0000843\_down | Actn1 |
| GO:0005178 | integrin binding | 5.36e-02 | 1.13e-01 | 1 | 12 | 68 | 14853 | 8.33% | 0.46% | 1.47% | 0.08% | CL\_0000843\_down | Actn1 |
| GO:0004896 | cytokine receptor activity | 5.82e-02 | 1.20e-01 | 1 | 12 | 74 | 14853 | 8.33% | 0.50% | 1.35% | 0.08% | CL\_0000843\_down | Cxcr2 |
| GO:0000977 | RNA polymerase II regulatory region sequence-specific DNA binding | 6.05e-02 | 1.22e-01 | 1 | 12 | 77 | 14853 | 8.33% | 0.52% | 1.30% | 0.08% | CL\_0000843\_down | Bhlhe41 |
| GO:0001076 | RNA polymerase II transcription factor binding transcription factor activity | 6.43e-02 | 1.27e-01 | 1 | 12 | 82 | 14853 | 8.33% | 0.55% | 1.22% | 0.08% | CL\_0000843\_down | Bhlhe41 |
| GO:0001012 | RNA polymerase II regulatory region DNA binding | 6.66e-02 | 1.28e-01 | 1 | 12 | 85 | 14853 | 8.33% | 0.57% | 1.18% | 0.08% | CL\_0000843\_down | Bhlhe41 |
| GO:0008238 | exopeptidase activity | 6.81e-02 | 1.28e-01 | 1 | 12 | 87 | 14853 | 8.33% | 0.59% | 1.15% | 0.08% | CL\_0000843\_down | Cpd |
| GO:0004553 | hydrolase activity, hydrolyzing O-glycosyl compounds | 6.81e-02 | 1.28e-01 | 1 | 12 | 87 | 14853 | 8.33% | 0.59% | 1.15% | 0.08% | CL\_0000843\_down | Lyz2 |
| GO:0008092 | cytoskeletal protein binding | 7.64e-02 | 1.37e-01 | 2 | 12 | 575 | 14853 | 16.67% | 3.87% | 0.35% | 0.08% | CL\_0000843\_down | Actn1, Pstpip2 |
| GO:0000982 | RNA polymerase II core promoter proximal region sequence-specific DNA binding transcription factor activity | 7.71e-02 | 1.37e-01 | 1 | 12 | 99 | 14853 | 8.33% | 0.67% | 1.01% | 0.08% | CL\_0000843\_down | Bhlhe41 |
| GO:0004222 | metalloendopeptidase activity | 7.71e-02 | 1.37e-01 | 1 | 12 | 99 | 14853 | 8.33% | 0.67% | 1.01% | 0.08% | CL\_0000843\_down | Mmp8 |
| GO:0003705 | RNA polymerase II distal enhancer sequence-specific DNA binding transcription factor activity | 7.86e-02 | 1.37e-01 | 1 | 12 | 101 | 14853 | 8.33% | 0.68% | 0.99% | 0.08% | CL\_0000843\_down | Bhlhe41 |
| GO:0016798 | hydrolase activity, acting on glycosyl bonds | 8.39e-02 | 1.44e-01 | 1 | 12 | 108 | 14853 | 8.33% | 0.73% | 0.93% | 0.08% | CL\_0000843\_down | Lyz2 |
| GO:0008528 | G-protein coupled peptide receptor activity | 8.61e-02 | 1.45e-01 | 1 | 12 | 111 | 14853 | 8.33% | 0.75% | 0.90% | 0.08% | CL\_0000843\_down | Cxcr2 |
| GO:0001653 | peptide receptor activity | 8.76e-02 | 1.45e-01 | 1 | 12 | 113 | 14853 | 8.33% | 0.76% | 0.88% | 0.08% | CL\_0000843\_down | Cxcr2 |
| GO:0016787 | hydrolase activity | 8.89e-02 | 1.45e-01 | 4 | 12 | 2201 | 14853 | 33.33% | 14.82% | 0.18% | 0.08% | CL\_0000843\_down | Cpd, Ltf, Lyz2, Mmp8 |
| GO:0008201 | heparin binding | 8.98e-02 | 1.45e-01 | 1 | 12 | 116 | 14853 | 8.33% | 0.78% | 0.86% | 0.08% | CL\_0000843\_down | Ltf |
| GO:0003714 | transcription corepressor activity | 9.43e-02 | 1.49e-01 | 1 | 12 | 122 | 14853 | 8.33% | 0.82% | 0.82% | 0.08% | CL\_0000843\_down | Bhlhe41 |
| GO:0000976 | transcription regulatory region sequence-specific DNA binding | 1.02e-01 | 1.60e-01 | 1 | 12 | 133 | 14853 | 8.33% | 0.90% | 0.75% | 0.08% | CL\_0000843\_down | Bhlhe41 |
| GO:0005539 | glycosaminoglycan binding | 1.14e-01 | 1.75e-01 | 1 | 12 | 149 | 14853 | 8.33% | 1.00% | 0.67% | 0.08% | CL\_0000843\_down | Ltf |
| GO:0005488 | binding | 1.17e-01 | 1.77e-01 | 11 | 12 | 10773 | 14853 | 91.67% | 72.53% | 0.10% | 0.08% | CL\_0000843\_down | Actn1, Bhlhe41, Cpd, Cxcr2, Igj, Lcn2, Ltf, Mmp8, Pstpip2, S100a8, S100a9 |
| GO:0004252 | serine-type endopeptidase activity | 1.18e-01 | 1.77e-01 | 1 | 12 | 155 | 14853 | 8.33% | 1.04% | 0.65% | 0.08% | CL\_0000843\_down | Mmp8 |
| GO:0001871 | pattern binding | 1.30e-01 | 1.89e-01 | 1 | 12 | 172 | 14853 | 8.33% | 1.16% | 0.58% | 0.08% | CL\_0000843\_down | Ltf |
| GO:0030247 | polysaccharide binding | 1.30e-01 | 1.89e-01 | 1 | 12 | 172 | 14853 | 8.33% | 1.16% | 0.58% | 0.08% | CL\_0000843\_down | Ltf |
| GO:0000981 | sequence-specific DNA binding RNA polymerase II transcription factor activity | 1.75e-01 | 2.50e-01 | 1 | 12 | 236 | 14853 | 8.33% | 1.59% | 0.42% | 0.08% | CL\_0000843\_down | Bhlhe41 |
| GO:0019899 | enzyme binding | 2.26e-01 | 3.15e-01 | 2 | 12 | 1116 | 14853 | 16.67% | 7.51% | 0.18% | 0.08% | CL\_0000843\_down | Bhlhe41, Lcn2 |
| GO:0003712 | transcription cofactor activity | 2.30e-01 | 3.15e-01 | 1 | 12 | 320 | 14853 | 8.33% | 2.15% | 0.31% | 0.08% | CL\_0000843\_down | Bhlhe41 |
| GO:0044212 | transcription regulatory region DNA binding | 2.30e-01 | 3.15e-01 | 1 | 12 | 320 | 14853 | 8.33% | 2.15% | 0.31% | 0.08% | CL\_0000843\_down | Bhlhe41 |
| GO:0000975 | regulatory region DNA binding | 2.35e-01 | 3.15e-01 | 1 | 12 | 328 | 14853 | 8.33% | 2.21% | 0.30% | 0.08% | CL\_0000843\_down | Bhlhe41 |
| GO:0001067 | regulatory region nucleic acid binding | 2.35e-01 | 3.15e-01 | 1 | 12 | 328 | 14853 | 8.33% | 2.21% | 0.30% | 0.08% | CL\_0000843\_down | Bhlhe41 |
| GO:0046982 | protein heterodimerization activity | 2.38e-01 | 3.15e-01 | 1 | 12 | 333 | 14853 | 8.33% | 2.24% | 0.30% | 0.08% | CL\_0000843\_down | Bhlhe41 |
| GO:0000989 | transcription factor binding transcription factor activity | 2.41e-01 | 3.15e-01 | 1 | 12 | 338 | 14853 | 8.33% | 2.28% | 0.30% | 0.08% | CL\_0000843\_down | Bhlhe41 |
| GO:0030246 | carbohydrate binding | 2.46e-01 | 3.17e-01 | 1 | 12 | 346 | 14853 | 8.33% | 2.33% | 0.29% | 0.08% | CL\_0000843\_down | Ltf |
| GO:0000988 | protein binding transcription factor activity | 2.50e-01 | 3.17e-01 | 1 | 12 | 351 | 14853 | 8.33% | 2.36% | 0.28% | 0.08% | CL\_0000843\_down | Bhlhe41 |
| GO:0004175 | endopeptidase activity | 2.53e-01 | 3.18e-01 | 1 | 12 | 357 | 14853 | 8.33% | 2.40% | 0.28% | 0.08% | CL\_0000843\_down | Mmp8 |
| GO:0008134 | transcription factor binding | 2.84e-01 | 3.52e-01 | 1 | 12 | 407 | 14853 | 8.33% | 2.74% | 0.25% | 0.08% | CL\_0000843\_down | Bhlhe41 |
| GO:0032403 | protein complex binding | 2.89e-01 | 3.54e-01 | 1 | 12 | 416 | 14853 | 8.33% | 2.80% | 0.24% | 0.08% | CL\_0000843\_down | Actn1 |
| GO:0008270 | zinc ion binding | 2.95e-01 | 3.57e-01 | 2 | 12 | 1337 | 14853 | 16.67% | 9.00% | 0.15% | 0.08% | CL\_0000843\_down | Cpd, Mmp8 |
| GO:0019904 | protein domain specific binding | 3.91e-01 | 4.68e-01 | 1 | 12 | 601 | 14853 | 8.33% | 4.05% | 0.17% | 0.08% | CL\_0000843\_down | Actn1 |
| GO:0043565 | sequence-specific DNA binding | 4.03e-01 | 4.77e-01 | 1 | 12 | 624 | 14853 | 8.33% | 4.20% | 0.16% | 0.08% | CL\_0000843\_down | Bhlhe41 |
| GO:0003700 | sequence-specific DNA binding transcription factor activity | 4.65e-01 | 5.39e-01 | 1 | 12 | 754 | 14853 | 8.33% | 5.08% | 0.13% | 0.08% | CL\_0000843\_down | Bhlhe41 |
| GO:0001071 | nucleic acid binding transcription factor activity | 4.66e-01 | 5.39e-01 | 1 | 12 | 756 | 14853 | 8.33% | 5.09% | 0.13% | 0.08% | CL\_0000843\_down | Bhlhe41 |
| GO:0005515 | protein binding | 5.76e-01 | 6.59e-01 | 5 | 12 | 6013 | 14853 | 41.67% | 40.48% | 0.08% | 0.08% | CL\_0000843\_down | Actn1, Bhlhe41, Cxcr2, Lcn2, Pstpip2 |
| GO:0005215 | transporter activity | 5.83e-01 | 6.60e-01 | 1 | 12 | 1044 | 14853 | 8.33% | 7.03% | 0.10% | 0.08% | CL\_0000843\_down | Lcn2 |
| GO:0005102 | receptor binding | 6.23e-01 | 6.97e-01 | 1 | 12 | 1159 | 14853 | 8.33% | 7.80% | 0.09% | 0.08% | CL\_0000843\_down | Actn1 |
| GO:0003824 | catalytic activity | 6.35e-01 | 7.03e-01 | 4 | 12 | 5097 | 14853 | 33.33% | 34.32% | 0.08% | 0.08% | CL\_0000843\_down | Cpd, Ltf, Lyz2, Mmp8 |
| GO:0004930 | G-protein coupled receptor activity | 7.50e-01 | 8.21e-01 | 1 | 12 | 1618 | 14853 | 8.33% | 10.89% | 0.06% | 0.08% | CL\_0000843\_down | Cxcr2 |
| GO:0003677 | DNA binding | 7.83e-01 | 8.49e-01 | 1 | 12 | 1774 | 14853 | 8.33% | 11.94% | 0.06% | 0.08% | CL\_0000843\_down | Bhlhe41 |
| GO:0004888 | transmembrane signaling receptor activity | 8.31e-01 | 8.92e-01 | 1 | 12 | 2046 | 14853 | 8.33% | 13.77% | 0.05% | 0.08% | CL\_0000843\_down | Cxcr2 |
| GO:0038023 | signaling receptor activity | 8.44e-01 | 8.96e-01 | 1 | 12 | 2128 | 14853 | 8.33% | 14.33% | 0.05% | 0.08% | CL\_0000843\_down | Cxcr2 |
| GO:0060089 | molecular transducer activity | 8.74e-01 | 9.05e-01 | 1 | 12 | 2352 | 14853 | 8.33% | 15.84% | 0.04% | 0.08% | CL\_0000843\_down | Cxcr2 |
| GO:0004871 | signal transducer activity | 8.74e-01 | 9.05e-01 | 1 | 12 | 2352 | 14853 | 8.33% | 15.84% | 0.04% | 0.08% | CL\_0000843\_down | Cxcr2 |
| GO:0036094 | small molecule binding | 8.79e-01 | 9.05e-01 | 1 | 12 | 2392 | 14853 | 8.33% | 16.10% | 0.04% | 0.08% | CL\_0000843\_down | Lcn2 |
| GO:0003676 | nucleic acid binding | 9.00e-01 | 9.18e-01 | 1 | 12 | 2594 | 14853 | 8.33% | 17.46% | 0.04% | 0.08% | CL\_0000843\_down | Bhlhe41 |
| GO:0004872 | receptor activity | 9.09e-01 | 9.18e-01 | 1 | 12 | 2687 | 14853 | 8.33% | 18.09% | 0.04% | 0.08% | CL\_0000843\_down | Cxcr2 |
| GO:0003674 | molecular\_function | 1.00e+00 | 1.00e+00 | 12 | 12 | 14853 | 14853 | 100.00% | 100.00% | 0.08% | 0.08% | CL\_0000843\_down | Actn1, Bhlhe41, Cpd, Cxcr2, Igj, Lcn2, Ltf, Lyz2, Mmp8, Pstpip2, S100a8, S100a9 |

### Unannotated IDs

|  |
| --- |
| **CL\_0000843\_down** |

|  |  |  |
| --- | --- | --- |
| [close] | **Legend: Edge Types** | (details) |
|  | | |
