# Supplementary material for: Ontology based molecular signatures for immune cell types via gene expression analysis
Source: BMC Bioinformatics. 2013 Aug 30;14:263. doi: 10.1186/1471-2105-14-263 (PMC3844401; doi:10.1186/1471-2105-14-263)
Supplement: Additional file 1 — OBAMS profiles for all mature B cells. Additional file 1 contains a zip archive of OBAMS profiles for all mature B cells, including for each cell type individual spreadsheets showing up and down regulated genes for that cell type relative to parental cell types, and VLAD (GO term enrichment) results for all mature B cells. [file 1471-2105-14-263-S1.zip › Additional File 1/Fraction F mature B cell/VLAD.fraction_F-down/results.html]

# fraction\_f\_mature\_down

|  |  |
| --- | --- |
| Vlad version: | v1.5.1 |
| Date: | Wed Jun 20 07:30:05 2012 |
| Run time: | 60.47 sec |
| Ontology file: | gene\_ontology.obo |
| Ontology date: | Tue Jun 19 19:30:00 2012 |
| Annotation file: | gene\_association.mgi |
| Annotation date: | Wed Jun 6 00:00:00 2012 |
| Analysis type: | enrichment |
| Excluded evidence codes: | ND |
| Number of query sets: | 1 |
| Query set 1: | fraction\_f\_mature\_down (n=3; 0 not found) |
| Universe set: | default (everything) |
| Graph display: | Top 25 scoring terms and their ancestors. Interior nodes have been culled. |

**Jump to:** biological\_process | cellular\_component | molecular\_function | Unannotated id/symbols

### biological\_process (top)

  
  


| TermID | Term | P | Q | k | n | K | N | k/n | K/N | k/K | n/N | Qset | Symbols |
| --- | --- | --- | --- | --- | --- | --- | --- | --- | --- | --- | --- | --- | --- |
| GO:0007616 | long-term memory | 2.77e-03 | 6.65e-02 | 1 | 2 | 21 | 15137 | 50.00% | 0.14% | 4.76% | 0.01% | fraction\_f\_mature\_down | Chst10 |
| GO:0007613 | memory | 1.00e-02 | 9.16e-02 | 1 | 2 | 76 | 15137 | 50.00% | 0.50% | 1.32% | 0.01% | fraction\_f\_mature\_down | Chst10 |
| GO:0007612 | learning | 1.24e-02 | 9.16e-02 | 1 | 2 | 94 | 15137 | 50.00% | 0.62% | 1.06% | 0.01% | fraction\_f\_mature\_down | Chst10 |
| GO:0016051 | carbohydrate biosynthetic process | 1.53e-02 | 9.16e-02 | 1 | 2 | 116 | 15137 | 50.00% | 0.77% | 0.86% | 0.01% | fraction\_f\_mature\_down | Chst10 |
| GO:0007611 | learning or memory | 2.14e-02 | 9.20e-02 | 1 | 2 | 163 | 15137 | 50.00% | 1.08% | 0.61% | 0.01% | fraction\_f\_mature\_down | Chst10 |
| GO:0050890 | cognition | 2.30e-02 | 9.20e-02 | 1 | 2 | 175 | 15137 | 50.00% | 1.16% | 0.57% | 0.01% | fraction\_f\_mature\_down | Chst10 |
| GO:0007610 | behavior | 5.79e-02 | 1.99e-01 | 1 | 2 | 445 | 15137 | 50.00% | 2.94% | 0.22% | 0.01% | fraction\_f\_mature\_down | Chst10 |
| GO:0005975 | carbohydrate metabolic process | 6.68e-02 | 2.00e-01 | 1 | 2 | 514 | 15137 | 50.00% | 3.40% | 0.19% | 0.01% | fraction\_f\_mature\_down | Chst10 |
| GO:0050896 | response to stimulus | 1.76e-01 | 4.69e-01 | 2 | 2 | 6348 | 15137 | 100.00% | 41.94% | 0.03% | 0.01% | fraction\_f\_mature\_down | Chst10, Tagap |
| GO:0050877 | neurological system process | 2.48e-01 | 5.95e-01 | 1 | 2 | 2011 | 15137 | 50.00% | 13.29% | 0.05% | 0.01% | fraction\_f\_mature\_down | Chst10 |
| GO:0003008 | system process | 2.78e-01 | 6.07e-01 | 1 | 2 | 2275 | 15137 | 50.00% | 15.03% | 0.04% | 0.01% | fraction\_f\_mature\_down | Chst10 |
| GO:0009058 | biosynthetic process | 3.66e-01 | 7.32e-01 | 1 | 2 | 3082 | 15137 | 50.00% | 20.36% | 0.03% | 0.01% | fraction\_f\_mature\_down | Chst10 |
| GO:0007165 | signal transduction | 4.67e-01 | 8.10e-01 | 1 | 2 | 4085 | 15137 | 50.00% | 26.99% | 0.02% | 0.01% | fraction\_f\_mature\_down | Tagap |
| GO:0023052 | signaling | 4.96e-01 | 8.10e-01 | 1 | 2 | 4387 | 15137 | 50.00% | 28.98% | 0.02% | 0.01% | fraction\_f\_mature\_down | Tagap |
| GO:0007154 | cell communication | 5.06e-01 | 8.10e-01 | 1 | 2 | 4503 | 15137 | 50.00% | 29.75% | 0.02% | 0.01% | fraction\_f\_mature\_down | Tagap |
| GO:0051716 | cellular response to stimulus | 5.51e-01 | 8.27e-01 | 1 | 2 | 4999 | 15137 | 50.00% | 33.03% | 0.02% | 0.01% | fraction\_f\_mature\_down | Tagap |
| GO:0032501 | multicellular organismal process | 5.85e-01 | 8.27e-01 | 1 | 2 | 5391 | 15137 | 50.00% | 35.61% | 0.02% | 0.01% | fraction\_f\_mature\_down | Chst10 |
| GO:0044238 | primary metabolic process | 6.78e-01 | 9.03e-01 | 1 | 2 | 6542 | 15137 | 50.00% | 43.22% | 0.02% | 0.01% | fraction\_f\_mature\_down | Chst10 |
| GO:0008152 | metabolic process | 7.48e-01 | 9.21e-01 | 1 | 2 | 7543 | 15137 | 50.00% | 49.83% | 0.01% | 0.01% | fraction\_f\_mature\_down | Chst10 |
| GO:0050794 | regulation of cellular process | 7.91e-01 | 9.21e-01 | 1 | 2 | 8223 | 15137 | 50.00% | 54.32% | 0.01% | 0.01% | fraction\_f\_mature\_down | Tagap |
| GO:0050789 | regulation of biological process | 8.25e-01 | 9.21e-01 | 1 | 2 | 8805 | 15137 | 50.00% | 58.17% | 0.01% | 0.01% | fraction\_f\_mature\_down | Tagap |
| GO:0065007 | biological regulation | 8.45e-01 | 9.21e-01 | 1 | 2 | 9171 | 15137 | 50.00% | 60.59% | 0.01% | 0.01% | fraction\_f\_mature\_down | Tagap |
| GO:0009987 | cellular process | 9.69e-01 | 1.00e+00 | 1 | 2 | 12456 | 15137 | 50.00% | 82.29% | 0.01% | 0.01% | fraction\_f\_mature\_down | Tagap |
| GO:0008150 | biological\_process | 1.00e+00 | 1.00e+00 | 2 | 2 | 15137 | 15137 | 100.00% | 100.00% | 0.01% | 0.01% | fraction\_f\_mature\_down | Chst10, Tagap |

### cellular\_component (top)

  
  


| TermID | Term | P | Q | k | n | K | N | k/n | K/N | k/K | n/N | Qset | Symbols |
| --- | --- | --- | --- | --- | --- | --- | --- | --- | --- | --- | --- | --- | --- |
| GO:0009897 | external side of plasma membrane | 4.55e-02 | 9.43e-01 | 1 | 3 | 253 | 16439 | 33.33% | 1.54% | 0.40% | 0.02% | fraction\_f\_mature\_down | Cd69 |
| GO:0009986 | cell surface | 9.65e-02 | 9.43e-01 | 1 | 3 | 547 | 16439 | 33.33% | 3.33% | 0.18% | 0.02% | fraction\_f\_mature\_down | Cd69 |
| GO:0005794 | Golgi apparatus | 1.69e-01 | 9.43e-01 | 1 | 3 | 983 | 16439 | 33.33% | 5.98% | 0.10% | 0.02% | fraction\_f\_mature\_down | Chst10 |
| GO:0044459 | plasma membrane part | 2.62e-01 | 9.43e-01 | 1 | 3 | 1582 | 16439 | 33.33% | 9.62% | 0.06% | 0.02% | fraction\_f\_mature\_down | Cd69 |
| GO:0016021 | integral to membrane | 2.76e-01 | 9.43e-01 | 2 | 3 | 5690 | 16439 | 66.67% | 34.61% | 0.04% | 0.02% | fraction\_f\_mature\_down | Cd69, Chst10 |
| GO:0031224 | intrinsic to membrane | 2.88e-01 | 9.43e-01 | 2 | 3 | 5824 | 16439 | 66.67% | 35.43% | 0.03% | 0.02% | fraction\_f\_mature\_down | Cd69, Chst10 |
| GO:0044425 | membrane part | 3.49e-01 | 9.43e-01 | 2 | 3 | 6537 | 16439 | 66.67% | 39.77% | 0.03% | 0.02% | fraction\_f\_mature\_down | Cd69, Chst10 |
| GO:0016020 | membrane | 4.89e-01 | 9.43e-01 | 2 | 3 | 8099 | 16439 | 66.67% | 49.27% | 0.02% | 0.02% | fraction\_f\_mature\_down | Cd69, Chst10 |
| GO:0044464 | cell part | 5.15e-01 | 9.43e-01 | 3 | 3 | 13174 | 16439 | 100.00% | 80.14% | 0.02% | 0.02% | fraction\_f\_mature\_down | Cd69, Chst10, Tagap |
| GO:0005623 | cell | 5.15e-01 | 9.43e-01 | 3 | 3 | 13175 | 16439 | 100.00% | 80.14% | 0.02% | 0.02% | fraction\_f\_mature\_down | Cd69, Chst10, Tagap |
| GO:0005886 | plasma membrane | 5.29e-01 | 9.43e-01 | 1 | 3 | 3649 | 16439 | 33.33% | 22.20% | 0.03% | 0.02% | fraction\_f\_mature\_down | Cd69 |
| GO:0071944 | cell periphery | 5.39e-01 | 9.43e-01 | 1 | 3 | 3737 | 16439 | 33.33% | 22.73% | 0.03% | 0.02% | fraction\_f\_mature\_down | Cd69 |
| GO:0044444 | cytoplasmic part | 7.04e-01 | 1.00e+00 | 1 | 3 | 5480 | 16439 | 33.33% | 33.34% | 0.02% | 0.02% | fraction\_f\_mature\_down | Chst10 |
| GO:0005622 | intracellular | 7.67e-01 | 1.00e+00 | 2 | 3 | 11282 | 16439 | 66.67% | 68.63% | 0.02% | 0.02% | fraction\_f\_mature\_down | Chst10, Tagap |
| GO:0005737 | cytoplasm | 8.80e-01 | 1.00e+00 | 1 | 3 | 8331 | 16439 | 33.33% | 50.68% | 0.01% | 0.02% | fraction\_f\_mature\_down | Chst10 |
| GO:0043231 | intracellular membrane-bounded organelle | 8.87e-01 | 1.00e+00 | 1 | 3 | 8480 | 16439 | 33.33% | 51.58% | 0.01% | 0.02% | fraction\_f\_mature\_down | Chst10 |
| GO:0043227 | membrane-bounded organelle | 8.87e-01 | 1.00e+00 | 1 | 3 | 8499 | 16439 | 33.33% | 51.70% | 0.01% | 0.02% | fraction\_f\_mature\_down | Chst10 |
| GO:0043229 | intracellular organelle | 9.28e-01 | 1.00e+00 | 1 | 3 | 9595 | 16439 | 33.33% | 58.37% | 0.01% | 0.02% | fraction\_f\_mature\_down | Chst10 |
| GO:0043226 | organelle | 9.29e-01 | 1.00e+00 | 1 | 3 | 9616 | 16439 | 33.33% | 58.50% | 0.01% | 0.02% | fraction\_f\_mature\_down | Chst10 |
| GO:0044424 | intracellular part | 9.65e-01 | 1.00e+00 | 1 | 3 | 11042 | 16439 | 33.33% | 67.17% | 0.01% | 0.02% | fraction\_f\_mature\_down | Chst10 |
| GO:0005575 | cellular\_component | 1.00e+00 | 1.00e+00 | 3 | 3 | 16439 | 16439 | 100.00% | 100.00% | 0.02% | 0.02% | fraction\_f\_mature\_down | Cd69, Chst10, Tagap |

### molecular\_function (top)

  
  


| TermID | Term | P | Q | k | n | K | N | k/n | K/N | k/K | n/N | Qset | Symbols |
| --- | --- | --- | --- | --- | --- | --- | --- | --- | --- | --- | --- | --- | --- |
| GO:0016232 | HNK-1 sulfotransferase activity | 2.02e-04 | 3.23e-03 | 1 | 3 | 1 | 14853 | 33.33% | 0.01% | 100.00% | 0.02% | fraction\_f\_mature\_down | Chst10 |
| GO:0008146 | sulfotransferase activity | 9.66e-03 | 6.01e-02 | 1 | 3 | 48 | 14853 | 33.33% | 0.32% | 2.08% | 0.02% | fraction\_f\_mature\_down | Chst10 |
| GO:0016782 | transferase activity, transferring sulfur-containing groups | 1.13e-02 | 6.01e-02 | 1 | 3 | 56 | 14853 | 33.33% | 0.38% | 1.79% | 0.02% | fraction\_f\_mature\_down | Chst10 |
| GO:0005085 | guanyl-nucleotide exchange factor activity | 2.82e-02 | 1.13e-01 | 1 | 3 | 141 | 14853 | 33.33% | 0.95% | 0.71% | 0.02% | fraction\_f\_mature\_down | Tagap |
| GO:0030246 | carbohydrate binding | 6.83e-02 | 1.64e-01 | 1 | 3 | 346 | 14853 | 33.33% | 2.33% | 0.29% | 0.02% | fraction\_f\_mature\_down | Cd69 |
| GO:0030695 | GTPase regulator activity | 6.92e-02 | 1.64e-01 | 1 | 3 | 351 | 14853 | 33.33% | 2.36% | 0.28% | 0.02% | fraction\_f\_mature\_down | Tagap |
| GO:0060589 | nucleoside-triphosphatase regulator activity | 7.15e-02 | 1.64e-01 | 1 | 3 | 363 | 14853 | 33.33% | 2.44% | 0.28% | 0.02% | fraction\_f\_mature\_down | Tagap |
| GO:0005509 | calcium ion binding | 1.01e-01 | 2.02e-01 | 1 | 3 | 518 | 14853 | 33.33% | 3.49% | 0.19% | 0.02% | fraction\_f\_mature\_down | Cd69 |
| GO:0030234 | enzyme regulator activity | 1.58e-01 | 2.82e-01 | 1 | 3 | 830 | 14853 | 33.33% | 5.59% | 0.12% | 0.02% | fraction\_f\_mature\_down | Tagap |
| GO:0016740 | transferase activity | 3.03e-01 | 4.84e-01 | 1 | 3 | 1681 | 14853 | 33.33% | 11.32% | 0.06% | 0.02% | fraction\_f\_mature\_down | Chst10 |
| GO:0046872 | metal ion binding | 5.45e-01 | 6.78e-01 | 1 | 3 | 3425 | 14853 | 33.33% | 23.06% | 0.03% | 0.02% | fraction\_f\_mature\_down | Cd69 |
| GO:0043169 | cation binding | 5.50e-01 | 6.78e-01 | 1 | 3 | 3467 | 14853 | 33.33% | 23.34% | 0.03% | 0.02% | fraction\_f\_mature\_down | Cd69 |
| GO:0043167 | ion binding | 5.51e-01 | 6.78e-01 | 1 | 3 | 3479 | 14853 | 33.33% | 23.42% | 0.03% | 0.02% | fraction\_f\_mature\_down | Cd69 |
| GO:0003824 | catalytic activity | 7.17e-01 | 8.19e-01 | 1 | 3 | 5097 | 14853 | 33.33% | 34.32% | 0.02% | 0.02% | fraction\_f\_mature\_down | Chst10 |
| GO:0005488 | binding | 9.79e-01 | 1.00e+00 | 1 | 3 | 10773 | 14853 | 33.33% | 72.53% | 0.01% | 0.02% | fraction\_f\_mature\_down | Cd69 |
| GO:0003674 | molecular\_function | 1.00e+00 | 1.00e+00 | 3 | 3 | 14853 | 14853 | 100.00% | 100.00% | 0.02% | 0.02% | fraction\_f\_mature\_down | Cd69, Chst10, Tagap |

### Unannotated IDs

|  |
| --- |
| **fraction\_f\_mature\_down** |

|  |  |  |
| --- | --- | --- |
| [close] | **Legend: Edge Types** | (details) |
|  | | |
