# Supplementary material for: Ontology based molecular signatures for immune cell types via gene expression analysis
Source: BMC Bioinformatics. 2013 Aug 30;14:263. doi: 10.1186/1471-2105-14-263 (PMC3844401; doi:10.1186/1471-2105-14-263)
Supplement: Additional file 1 — OBAMS profiles for all mature B cells. Additional file 1 contains a zip archive of OBAMS profiles for all mature B cells, including for each cell type individual spreadsheets showing up and down regulated genes for that cell type relative to parental cell types, and VLAD (GO term enrichment) results for all mature B cells. [file 1471-2105-14-263-S1.zip › Additional File 1/Fraction F mature B cell/VLAD.fraction_F_up/results.html]

# fraction F up

|  |  |
| --- | --- |
| Vlad version: | v1.5 |
| Date: | Thu Nov 3 13:20:47 2011 |
| Run time: | 26.81 sec |
| Ontology file: | gene\_ontology.obo |
| Ontology date: | Wed Nov 2 19:30:00 2011 |
| Annotation file: | gene\_association.mgi |
| Annotation date: | ??? |
| Analysis type: | enrichment |
| Excluded evidence codes: | ND |
| Number of query sets: | 1 |
| Query set 1: | Fraction F up (n=14; 0 not found) |
| Universe set: | default (everything) |
| Graph display: | Top 25 scoring terms and their ancestors. Interior nodes have been culled. |

**Jump to:** biological\_process | cellular\_component | molecular\_function | Unannotated id/symbols

### biological\_process (top)

  
  


| TermID | Term | Pval | k | n | K | N | k/n | K/N | k/K | n/N | Qset | Symbols |
| --- | --- | --- | --- | --- | --- | --- | --- | --- | --- | --- | --- | --- |
| GO:0006826 | iron ion transport | 1.23e-04 | 2 | 12 | 21 | 14950 | 16.67% | 0.14% | 9.52% | 0.08% | Fraction F up | Lcn2, Ltf |
| GO:0006879 | cellular iron ion homeostasis | 2.37e-04 | 2 | 12 | 29 | 14950 | 16.67% | 0.19% | 6.90% | 0.08% | Fraction F up | Lcn2, Ltf |
| GO:0055072 | iron ion homeostasis | 7.08e-04 | 2 | 12 | 50 | 14950 | 16.67% | 0.33% | 4.00% | 0.08% | Fraction F up | Lcn2, Ltf |
| GO:0009635 | response to herbicide | 8.03e-04 | 1 | 12 | 1 | 14950 | 8.33% | 0.01% | 100.00% | 0.08% | Fraction F up | Lcn2 |
| GO:0006875 | cellular metal ion homeostasis | 9.52e-04 | 3 | 12 | 254 | 14950 | 25.00% | 1.70% | 1.18% | 0.08% | Fraction F up | Cxcr2, Lcn2, Ltf |
| GO:0030595 | leukocyte chemotaxis | 9.85e-04 | 2 | 12 | 59 | 14950 | 16.67% | 0.39% | 3.39% | 0.08% | Fraction F up | Cxcr2, S100a9 |
| GO:0055065 | metal ion homeostasis | 1.08e-03 | 3 | 12 | 265 | 14950 | 25.00% | 1.77% | 1.13% | 0.08% | Fraction F up | Cxcr2, Lcn2, Ltf |
| GO:0009605 | response to external stimulus | 1.09e-03 | 4 | 12 | 617 | 14950 | 33.33% | 4.13% | 0.65% | 0.08% | Fraction F up | Bhlhe41, Cxcr2, S100a8, S100a9 |
| GO:0000041 | transition metal ion transport | 1.23e-03 | 2 | 12 | 66 | 14950 | 16.67% | 0.44% | 3.03% | 0.08% | Fraction F up | Lcn2, Ltf |
| GO:0030003 | cellular cation homeostasis | 1.31e-03 | 3 | 12 | 284 | 14950 | 25.00% | 1.90% | 1.06% | 0.08% | Fraction F up | Cxcr2, Lcn2, Ltf |
| GO:0006935 | chemotaxis | 1.37e-03 | 3 | 12 | 288 | 14950 | 25.00% | 1.93% | 1.04% | 0.08% | Fraction F up | Cxcr2, S100a8, S100a9 |
| GO:0042330 | taxis | 1.38e-03 | 3 | 12 | 289 | 14950 | 25.00% | 1.93% | 1.04% | 0.08% | Fraction F up | Cxcr2, S100a8, S100a9 |
| GO:0060326 | cell chemotaxis | 1.94e-03 | 2 | 12 | 83 | 14950 | 16.67% | 0.56% | 2.41% | 0.08% | Fraction F up | Cxcr2, S100a9 |
| GO:0055080 | cation homeostasis | 2.07e-03 | 3 | 12 | 333 | 14950 | 25.00% | 2.23% | 0.90% | 0.08% | Fraction F up | Cxcr2, Lcn2, Ltf |
| GO:0050900 | leukocyte migration | 2.18e-03 | 2 | 12 | 88 | 14950 | 16.67% | 0.59% | 2.27% | 0.08% | Fraction F up | Cxcr2, S100a9 |
| GO:0033212 | iron assimilation | 2.41e-03 | 1 | 12 | 3 | 14950 | 8.33% | 0.02% | 33.33% | 0.08% | Fraction F up | Lcn2 |
| GO:0033214 | iron assimilation by chelation and transport | 2.41e-03 | 1 | 12 | 3 | 14950 | 8.33% | 0.02% | 33.33% | 0.08% | Fraction F up | Lcn2 |
| GO:0015688 | iron chelate transport | 2.41e-03 | 1 | 12 | 3 | 14950 | 8.33% | 0.02% | 33.33% | 0.08% | Fraction F up | Lcn2 |
| GO:0015891 | siderophore transport | 2.41e-03 | 1 | 12 | 3 | 14950 | 8.33% | 0.02% | 33.33% | 0.08% | Fraction F up | Lcn2 |
| GO:0045113 | regulation of integrin biosynthetic process | 3.21e-03 | 1 | 12 | 4 | 14950 | 8.33% | 0.03% | 25.00% | 0.08% | Fraction F up | S100a9 |
| GO:0009649 | entrainment of circadian clock | 4.81e-03 | 1 | 12 | 6 | 14950 | 8.33% | 0.04% | 16.67% | 0.08% | Fraction F up | Bhlhe41 |
| GO:0006873 | cellular ion homeostasis | 5.18e-03 | 3 | 12 | 460 | 14950 | 25.00% | 3.08% | 0.65% | 0.08% | Fraction F up | Cxcr2, Lcn2, Ltf |
| GO:0002376 | immune system process | 5.27e-03 | 4 | 12 | 948 | 14950 | 33.33% | 6.34% | 0.42% | 0.08% | Fraction F up | Cxcr2, Igj, Lcn2, S100a9 |
| GO:0055082 | cellular chemical homeostasis | 5.63e-03 | 3 | 12 | 474 | 14950 | 25.00% | 3.17% | 0.63% | 0.08% | Fraction F up | Cxcr2, Lcn2, Ltf |
| GO:0007010 | cytoskeleton organization | 6.49e-03 | 3 | 12 | 499 | 14950 | 25.00% | 3.34% | 0.60% | 0.08% | Fraction F up | Actn1, Pstpip2, S100a9 |
| GO:0050801 | ion homeostasis | 6.90e-03 | 3 | 12 | 510 | 14950 | 25.00% | 3.41% | 0.59% | 0.08% | Fraction F up | Cxcr2, Lcn2, Ltf |
| GO:0042119 | neutrophil activation | 7.20e-03 | 1 | 12 | 9 | 14950 | 8.33% | 0.06% | 11.11% | 0.08% | Fraction F up | Cxcr2 |
| GO:0019725 | cellular homeostasis | 8.46e-03 | 3 | 12 | 549 | 14950 | 25.00% | 3.67% | 0.55% | 0.08% | Fraction F up | Cxcr2, Lcn2, Ltf |
| GO:0006952 | defense response | 8.80e-03 | 3 | 12 | 557 | 14950 | 25.00% | 3.73% | 0.54% | 0.08% | Fraction F up | Cxcr2, Lcn2, Lyz2 |
| GO:0016998 | cell wall macromolecule catabolic process | 1.04e-02 | 1 | 12 | 13 | 14950 | 8.33% | 0.09% | 7.69% | 0.08% | Fraction F up | Lyz2 |
| GO:0044036 | cell wall macromolecule metabolic process | 1.04e-02 | 1 | 12 | 13 | 14950 | 8.33% | 0.09% | 7.69% | 0.08% | Fraction F up | Lyz2 |
| GO:0071554 | cell wall organization or biogenesis | 1.04e-02 | 1 | 12 | 13 | 14950 | 8.33% | 0.09% | 7.69% | 0.08% | Fraction F up | Lyz2 |
| GO:0006968 | cellular defense response | 1.12e-02 | 1 | 12 | 14 | 14950 | 8.33% | 0.09% | 7.14% | 0.08% | Fraction F up | Cxcr2 |
| GO:0048041 | focal adhesion assembly | 1.28e-02 | 1 | 12 | 16 | 14950 | 8.33% | 0.11% | 6.25% | 0.08% | Fraction F up | Actn1 |
| GO:0048878 | chemical homeostasis | 1.35e-02 | 3 | 12 | 651 | 14950 | 25.00% | 4.35% | 0.46% | 0.08% | Fraction F up | Cxcr2, Lcn2, Ltf |
| GO:0030574 | collagen catabolic process | 1.36e-02 | 1 | 12 | 17 | 14950 | 8.33% | 0.11% | 5.88% | 0.08% | Fraction F up | Mmp8 |
| GO:0040011 | locomotion | 1.38e-02 | 3 | 12 | 657 | 14950 | 25.00% | 4.39% | 0.46% | 0.08% | Fraction F up | Cxcr2, S100a8, S100a9 |
| GO:0050829 | defense response to Gram-negative bacterium | 1.52e-02 | 1 | 12 | 19 | 14950 | 8.33% | 0.13% | 5.26% | 0.08% | Fraction F up | Lyz2 |
| GO:0070207 | protein homotrimerization | 1.52e-02 | 1 | 12 | 19 | 14950 | 8.33% | 0.13% | 5.26% | 0.08% | Fraction F up | Lcn2 |
| GO:0030036 | actin cytoskeleton organization | 1.55e-02 | 2 | 12 | 242 | 14950 | 16.67% | 1.62% | 0.83% | 0.08% | Fraction F up | Actn1, S100a9 |
| GO:0044243 | multicellular organismal catabolic process | 1.59e-02 | 1 | 12 | 20 | 14950 | 8.33% | 0.13% | 5.00% | 0.08% | Fraction F up | Mmp8 |
| GO:0008219 | cell death | 1.63e-02 | 3 | 12 | 699 | 14950 | 25.00% | 4.68% | 0.43% | 0.08% | Fraction F up | Cxcr2, Lcn2, Lyz2 |
| GO:0030865 | cortical cytoskeleton organization | 1.67e-02 | 1 | 12 | 21 | 14950 | 8.33% | 0.14% | 4.76% | 0.08% | Fraction F up | Actn1 |
| GO:0016265 | death | 1.69e-02 | 3 | 12 | 708 | 14950 | 25.00% | 4.74% | 0.42% | 0.08% | Fraction F up | Cxcr2, Lcn2, Lyz2 |
| GO:0030029 | actin filament-based process | 1.75e-02 | 2 | 12 | 258 | 14950 | 16.67% | 1.73% | 0.78% | 0.08% | Fraction F up | Actn1, S100a9 |
| GO:0019835 | cytolysis | 1.75e-02 | 1 | 12 | 22 | 14950 | 8.33% | 0.15% | 4.55% | 0.08% | Fraction F up | Lyz2 |
| GO:0006508 | proteolysis | 1.96e-02 | 3 | 12 | 749 | 14950 | 25.00% | 5.01% | 0.40% | 0.08% | Fraction F up | Cpd, Ltf, Mmp8 |
| GO:0051017 | actin filament bundle assembly | 2.07e-02 | 1 | 12 | 26 | 14950 | 8.33% | 0.17% | 3.85% | 0.08% | Fraction F up | Actn1 |
| GO:0007044 | cell-substrate junction assembly | 2.15e-02 | 1 | 12 | 27 | 14950 | 8.33% | 0.18% | 3.70% | 0.08% | Fraction F up | Actn1 |
| GO:0030593 | neutrophil chemotaxis | 2.15e-02 | 1 | 12 | 27 | 14950 | 8.33% | 0.18% | 3.70% | 0.08% | Fraction F up | Cxcr2 |
| GO:0070301 | cellular response to hydrogen peroxide | 2.38e-02 | 1 | 12 | 30 | 14950 | 8.33% | 0.20% | 3.33% | 0.08% | Fraction F up | Lcn2 |
| GO:0032963 | collagen metabolic process | 2.38e-02 | 1 | 12 | 30 | 14950 | 8.33% | 0.20% | 3.33% | 0.08% | Fraction F up | Mmp8 |
| GO:0070206 | protein trimerization | 2.38e-02 | 1 | 12 | 30 | 14950 | 8.33% | 0.20% | 3.33% | 0.08% | Fraction F up | Lcn2 |
| GO:0031532 | actin cytoskeleton reorganization | 2.54e-02 | 1 | 12 | 32 | 14950 | 8.33% | 0.21% | 3.12% | 0.08% | Fraction F up | S100a9 |
| GO:0044259 | multicellular organismal macromolecule metabolic process | 2.54e-02 | 1 | 12 | 32 | 14950 | 8.33% | 0.21% | 3.12% | 0.08% | Fraction F up | Mmp8 |
| GO:0042752 | regulation of circadian rhythm | 2.70e-02 | 1 | 12 | 34 | 14950 | 8.33% | 0.23% | 2.94% | 0.08% | Fraction F up | Bhlhe41 |
| GO:0070887 | cellular response to chemical stimulus | 2.74e-02 | 3 | 12 | 850 | 14950 | 25.00% | 5.69% | 0.35% | 0.08% | Fraction F up | Cxcr2, Lcn2, S100a9 |
| GO:0044236 | multicellular organismal metabolic process | 2.93e-02 | 1 | 12 | 37 | 14950 | 8.33% | 0.25% | 2.70% | 0.08% | Fraction F up | Mmp8 |
| GO:0007200 | activation of phospholipase C activity by G-protein coupled receptor protein signaling pathway coupled to IP3 second messenger | 3.32e-02 | 1 | 12 | 42 | 14950 | 8.33% | 0.28% | 2.38% | 0.08% | Fraction F up | Cxcr2 |
| GO:0042592 | homeostatic process | 3.38e-02 | 3 | 12 | 922 | 14950 | 25.00% | 6.17% | 0.33% | 0.08% | Fraction F up | Cxcr2, Lcn2, Ltf |
| GO:0034614 | cellular response to reactive oxygen species | 3.48e-02 | 1 | 12 | 44 | 14950 | 8.33% | 0.29% | 2.27% | 0.08% | Fraction F up | Lcn2 |
| GO:0048016 | inositol phosphate-mediated signaling | 3.55e-02 | 1 | 12 | 45 | 14950 | 8.33% | 0.30% | 2.22% | 0.08% | Fraction F up | Cxcr2 |
| GO:0051707 | response to other organism | 3.61e-02 | 2 | 12 | 381 | 14950 | 16.67% | 2.55% | 0.52% | 0.08% | Fraction F up | Lcn2, Lyz2 |
| GO:0050830 | defense response to Gram-positive bacterium | 3.71e-02 | 1 | 12 | 47 | 14950 | 8.33% | 0.31% | 2.13% | 0.08% | Fraction F up | Lyz2 |
| GO:0009607 | response to biotic stimulus | 4.09e-02 | 2 | 12 | 408 | 14950 | 16.67% | 2.73% | 0.49% | 0.08% | Fraction F up | Lcn2, Lyz2 |
| GO:0007202 | activation of phospholipase C activity | 4.10e-02 | 1 | 12 | 52 | 14950 | 8.33% | 0.35% | 1.92% | 0.08% | Fraction F up | Cxcr2 |
| GO:0006955 | immune response | 4.15e-02 | 2 | 12 | 411 | 14950 | 16.67% | 2.75% | 0.49% | 0.08% | Fraction F up | Igj, Lcn2 |
| GO:0034329 | cell junction assembly | 4.17e-02 | 1 | 12 | 53 | 14950 | 8.33% | 0.35% | 1.89% | 0.08% | Fraction F up | Actn1 |
| GO:0042542 | response to hydrogen peroxide | 4.17e-02 | 1 | 12 | 53 | 14950 | 8.33% | 0.35% | 1.89% | 0.08% | Fraction F up | Lcn2 |
| GO:0010863 | positive regulation of phospholipase C activity | 4.48e-02 | 1 | 12 | 57 | 14950 | 8.33% | 0.38% | 1.75% | 0.08% | Fraction F up | Cxcr2 |
| GO:0009636 | response to toxin | 4.48e-02 | 1 | 12 | 57 | 14950 | 8.33% | 0.38% | 1.75% | 0.08% | Fraction F up | Lcn2 |
| GO:0016477 | cell migration | 4.65e-02 | 2 | 12 | 438 | 14950 | 16.67% | 2.93% | 0.46% | 0.08% | Fraction F up | Cxcr2, S100a9 |
| GO:0002274 | myeloid leukocyte activation | 4.87e-02 | 1 | 12 | 62 | 14950 | 8.33% | 0.41% | 1.61% | 0.08% | Fraction F up | Cxcr2 |
| GO:0006959 | humoral immune response | 4.94e-02 | 1 | 12 | 63 | 14950 | 8.33% | 0.42% | 1.59% | 0.08% | Fraction F up | Igj |
| GO:0010518 | positive regulation of phospholipase activity | 5.02e-02 | 1 | 12 | 64 | 14950 | 8.33% | 0.43% | 1.56% | 0.08% | Fraction F up | Cxcr2 |
| GO:0007160 | cell-matrix adhesion | 5.48e-02 | 1 | 12 | 70 | 14950 | 8.33% | 0.47% | 1.43% | 0.08% | Fraction F up | Actn1 |
| GO:0010517 | regulation of phospholipase activity | 5.48e-02 | 1 | 12 | 70 | 14950 | 8.33% | 0.47% | 1.43% | 0.08% | Fraction F up | Cxcr2 |
| GO:0030001 | metal ion transport | 5.53e-02 | 2 | 12 | 482 | 14950 | 16.67% | 3.22% | 0.41% | 0.08% | Fraction F up | Lcn2, Ltf |
| GO:0034599 | cellular response to oxidative stress | 5.63e-02 | 1 | 12 | 72 | 14950 | 8.33% | 0.48% | 1.39% | 0.08% | Fraction F up | Lcn2 |
| GO:0060193 | positive regulation of lipase activity | 5.63e-02 | 1 | 12 | 72 | 14950 | 8.33% | 0.48% | 1.39% | 0.08% | Fraction F up | Cxcr2 |
| GO:0048870 | cell motility | 5.69e-02 | 2 | 12 | 490 | 14950 | 16.67% | 3.28% | 0.41% | 0.08% | Fraction F up | Cxcr2, S100a9 |
| GO:0051674 | localization of cell | 5.69e-02 | 2 | 12 | 490 | 14950 | 16.67% | 3.28% | 0.41% | 0.08% | Fraction F up | Cxcr2, S100a9 |
| GO:0034330 | cell junction organization | 6.01e-02 | 1 | 12 | 77 | 14950 | 8.33% | 0.52% | 1.30% | 0.08% | Fraction F up | Actn1 |
| GO:0000302 | response to reactive oxygen species | 6.01e-02 | 1 | 12 | 77 | 14950 | 8.33% | 0.52% | 1.30% | 0.08% | Fraction F up | Lcn2 |
| GO:0007015 | actin filament organization | 6.46e-02 | 1 | 12 | 83 | 14950 | 8.33% | 0.56% | 1.20% | 0.08% | Fraction F up | Actn1 |
| GO:0071840 | cellular component organization or biogenesis | 6.86e-02 | 5 | 12 | 2946 | 14950 | 41.67% | 19.71% | 0.17% | 0.08% | Fraction F up | Actn1, Lcn2, Lyz2, Pstpip2, S100a9 |
| GO:0051704 | multi-organism process | 7.12e-02 | 2 | 12 | 556 | 14950 | 16.67% | 3.72% | 0.36% | 0.08% | Fraction F up | Lcn2, Lyz2 |
| GO:0060191 | regulation of lipase activity | 7.44e-02 | 1 | 12 | 96 | 14950 | 8.33% | 0.64% | 1.04% | 0.08% | Fraction F up | Cxcr2 |
| GO:0051271 | negative regulation of cellular component movement | 7.74e-02 | 1 | 12 | 100 | 14950 | 8.33% | 0.67% | 1.00% | 0.08% | Fraction F up | Actn1 |
| GO:0031589 | cell-substrate adhesion | 8.04e-02 | 1 | 12 | 104 | 14950 | 8.33% | 0.70% | 0.96% | 0.08% | Fraction F up | Actn1 |
| GO:0006928 | cellular component movement | 8.30e-02 | 2 | 12 | 607 | 14950 | 16.67% | 4.06% | 0.33% | 0.08% | Fraction F up | Cxcr2, S100a9 |
| GO:0006812 | cation transport | 8.47e-02 | 2 | 12 | 614 | 14950 | 16.67% | 4.11% | 0.33% | 0.08% | Fraction F up | Lcn2, Ltf |
| GO:0006915 | apoptosis | 8.85e-02 | 2 | 12 | 630 | 14950 | 16.67% | 4.21% | 0.32% | 0.08% | Fraction F up | Cxcr2, Lcn2 |
| GO:0012501 | programmed cell death | 9.12e-02 | 2 | 12 | 641 | 14950 | 16.67% | 4.29% | 0.31% | 0.08% | Fraction F up | Cxcr2, Lcn2 |
| GO:0009615 | response to virus | 9.51e-02 | 1 | 12 | 124 | 14950 | 8.33% | 0.83% | 0.81% | 0.08% | Fraction F up | Lcn2 |
| GO:0019221 | cytokine-mediated signaling pathway | 9.88e-02 | 1 | 12 | 129 | 14950 | 8.33% | 0.86% | 0.78% | 0.08% | Fraction F up | Cxcr2 |
| GO:0007204 | elevation of cytosolic calcium ion concentration | 1.00e-01 | 1 | 12 | 131 | 14950 | 8.33% | 0.88% | 0.76% | 0.08% | Fraction F up | Cxcr2 |
| GO:0031346 | positive regulation of cell projection organization | 1.02e-01 | 1 | 12 | 134 | 14950 | 8.33% | 0.90% | 0.75% | 0.08% | Fraction F up | Lcn2 |
| GO:0006996 | organelle organization | 1.10e-01 | 3 | 12 | 1492 | 14950 | 25.00% | 9.98% | 0.20% | 0.08% | Fraction F up | Actn1, Pstpip2, S100a9 |
| GO:0042742 | defense response to bacterium | 1.11e-01 | 1 | 12 | 146 | 14950 | 8.33% | 0.98% | 0.68% | 0.08% | Fraction F up | Lyz2 |
| GO:0051480 | cytosolic calcium ion homeostasis | 1.13e-01 | 1 | 12 | 149 | 14950 | 8.33% | 1.00% | 0.67% | 0.08% | Fraction F up | Cxcr2 |
| GO:0051260 | protein homooligomerization | 1.19e-01 | 1 | 12 | 157 | 14950 | 8.33% | 1.05% | 0.64% | 0.08% | Fraction F up | Lcn2 |
| GO:0042493 | response to drug | 1.21e-01 | 1 | 12 | 160 | 14950 | 8.33% | 1.07% | 0.62% | 0.08% | Fraction F up | Lcn2 |
| GO:0006979 | response to oxidative stress | 1.32e-01 | 1 | 12 | 175 | 14950 | 8.33% | 1.17% | 0.57% | 0.08% | Fraction F up | Lcn2 |
| GO:0019932 | second-messenger-mediated signaling | 1.32e-01 | 1 | 12 | 175 | 14950 | 8.33% | 1.17% | 0.57% | 0.08% | Fraction F up | Cxcr2 |
| GO:0045087 | innate immune response | 1.33e-01 | 1 | 12 | 177 | 14950 | 8.33% | 1.18% | 0.56% | 0.08% | Fraction F up | Lcn2 |
| GO:0006811 | ion transport | 1.35e-01 | 2 | 12 | 810 | 14950 | 16.67% | 5.42% | 0.25% | 0.08% | Fraction F up | Lcn2, Ltf |
| GO:0071345 | cellular response to cytokine stimulus | 1.35e-01 | 1 | 12 | 180 | 14950 | 8.33% | 1.20% | 0.56% | 0.08% | Fraction F up | Cxcr2 |
| GO:0006950 | response to stress | 1.38e-01 | 3 | 12 | 1645 | 14950 | 25.00% | 11.00% | 0.18% | 0.08% | Fraction F up | Cxcr2, Lcn2, Lyz2 |
| GO:0065008 | regulation of biological quality | 1.50e-01 | 3 | 12 | 1708 | 14950 | 25.00% | 11.42% | 0.18% | 0.08% | Fraction F up | Cxcr2, Lcn2, Ltf |
| GO:0006874 | cellular calcium ion homeostasis | 1.58e-01 | 1 | 12 | 212 | 14950 | 8.33% | 1.42% | 0.47% | 0.08% | Fraction F up | Cxcr2 |
| GO:0010035 | response to inorganic substance | 1.60e-01 | 1 | 12 | 216 | 14950 | 8.33% | 1.44% | 0.46% | 0.08% | Fraction F up | Lcn2 |
| GO:0055074 | calcium ion homeostasis | 1.62e-01 | 1 | 12 | 219 | 14950 | 8.33% | 1.46% | 0.46% | 0.08% | Fraction F up | Cxcr2 |
| GO:0072503 | cellular divalent inorganic cation homeostasis | 1.63e-01 | 1 | 12 | 220 | 14950 | 8.33% | 1.47% | 0.45% | 0.08% | Fraction F up | Cxcr2 |
| GO:0022607 | cellular component assembly | 1.65e-01 | 2 | 12 | 915 | 14950 | 16.67% | 6.12% | 0.22% | 0.08% | Fraction F up | Actn1, Lcn2 |
| GO:0072507 | divalent inorganic cation homeostasis | 1.68e-01 | 1 | 12 | 228 | 14950 | 8.33% | 1.53% | 0.44% | 0.08% | Fraction F up | Cxcr2 |
| GO:0050896 | response to stimulus | 1.70e-01 | 7 | 12 | 6077 | 14950 | 58.33% | 40.65% | 0.12% | 0.08% | Fraction F up | Bhlhe41, Cxcr2, Igj, Lcn2, Lyz2, S100a8, S100a9 |
| GO:0016043 | cellular component organization | 1.75e-01 | 4 | 12 | 2815 | 14950 | 33.33% | 18.83% | 0.14% | 0.08% | Fraction F up | Actn1, Lcn2, Pstpip2, S100a9 |
| GO:0031344 | regulation of cell projection organization | 1.81e-01 | 1 | 12 | 246 | 14950 | 8.33% | 1.65% | 0.41% | 0.08% | Fraction F up | Lcn2 |
| GO:0034097 | response to cytokine stimulus | 1.85e-01 | 1 | 12 | 253 | 14950 | 8.33% | 1.69% | 0.40% | 0.08% | Fraction F up | Cxcr2 |
| GO:0009617 | response to bacterium | 1.87e-01 | 1 | 12 | 256 | 14950 | 8.33% | 1.71% | 0.39% | 0.08% | Fraction F up | Lyz2 |
| GO:0051128 | regulation of cellular component organization | 1.94e-01 | 2 | 12 | 1017 | 14950 | 16.67% | 6.80% | 0.20% | 0.08% | Fraction F up | Lcn2, S100a9 |
| GO:0051259 | protein oligomerization | 2.03e-01 | 1 | 12 | 280 | 14950 | 8.33% | 1.87% | 0.36% | 0.08% | Fraction F up | Lcn2 |
| GO:0044085 | cellular component biogenesis | 2.03e-01 | 2 | 12 | 1047 | 14950 | 16.67% | 7.00% | 0.19% | 0.08% | Fraction F up | Actn1, Lcn2 |
| GO:0042221 | response to chemical stimulus | 2.09e-01 | 4 | 12 | 3010 | 14950 | 33.33% | 20.13% | 0.13% | 0.08% | Fraction F up | Cxcr2, Lcn2, S100a8, S100a9 |
| GO:0045321 | leukocyte activation | 2.19e-01 | 1 | 12 | 304 | 14950 | 8.33% | 2.03% | 0.33% | 0.08% | Fraction F up | Cxcr2 |
| GO:0051179 | localization | 2.48e-01 | 4 | 12 | 3220 | 14950 | 33.33% | 21.54% | 0.12% | 0.08% | Fraction F up | Cxcr2, Lcn2, Ltf, S100a9 |
| GO:0001775 | cell activation | 2.48e-01 | 1 | 12 | 351 | 14950 | 8.33% | 2.35% | 0.28% | 0.08% | Fraction F up | Cxcr2 |
| GO:0071842 | cellular component organization at cellular level | 2.48e-01 | 3 | 12 | 2173 | 14950 | 25.00% | 14.54% | 0.14% | 0.08% | Fraction F up | Actn1, Pstpip2, S100a9 |
| GO:0051270 | regulation of cellular component movement | 2.52e-01 | 1 | 12 | 358 | 14950 | 8.33% | 2.39% | 0.28% | 0.08% | Fraction F up | Actn1 |
| GO:0071841 | cellular component organization or biogenesis at cellular level | 2.75e-01 | 3 | 12 | 2289 | 14950 | 25.00% | 15.31% | 0.13% | 0.08% | Fraction F up | Actn1, Pstpip2, S100a9 |
| GO:0051130 | positive regulation of cellular component organization | 2.90e-01 | 1 | 12 | 421 | 14950 | 8.33% | 2.82% | 0.24% | 0.08% | Fraction F up | Lcn2 |
| GO:0051345 | positive regulation of hydrolase activity | 3.01e-01 | 1 | 12 | 440 | 14950 | 8.33% | 2.94% | 0.23% | 0.08% | Fraction F up | Cxcr2 |
| GO:0006461 | protein complex assembly | 3.16e-01 | 1 | 12 | 465 | 14950 | 8.33% | 3.11% | 0.22% | 0.08% | Fraction F up | Lcn2 |
| GO:0070271 | protein complex biogenesis | 3.17e-01 | 1 | 12 | 467 | 14950 | 8.33% | 3.12% | 0.21% | 0.08% | Fraction F up | Lcn2 |
| GO:0000122 | negative regulation of transcription from RNA polymerase II promoter | 3.22e-01 | 1 | 12 | 476 | 14950 | 8.33% | 3.18% | 0.21% | 0.08% | Fraction F up | Bhlhe41 |
| GO:0071822 | protein complex subunit organization | 3.26e-01 | 1 | 12 | 483 | 14950 | 8.33% | 3.23% | 0.21% | 0.08% | Fraction F up | Lcn2 |
| GO:0019538 | protein metabolic process | 3.71e-01 | 3 | 12 | 2696 | 14950 | 25.00% | 18.03% | 0.11% | 0.08% | Fraction F up | Cpd, Ltf, Mmp8 |
| GO:0008284 | positive regulation of cell proliferation | 3.77e-01 | 1 | 12 | 578 | 14950 | 8.33% | 3.87% | 0.17% | 0.08% | Fraction F up | Cxcr2 |
| GO:0043170 | macromolecule metabolic process | 3.86e-01 | 5 | 12 | 5075 | 14950 | 41.67% | 33.95% | 0.10% | 0.08% | Fraction F up | Bhlhe41, Cpd, Ltf, Lyz2, Mmp8 |
| GO:0065003 | macromolecular complex assembly | 3.89e-01 | 1 | 12 | 601 | 14950 | 8.33% | 4.02% | 0.17% | 0.08% | Fraction F up | Lcn2 |
| GO:0071844 | cellular component assembly at cellular level | 3.90e-01 | 1 | 12 | 604 | 14950 | 8.33% | 4.04% | 0.17% | 0.08% | Fraction F up | Actn1 |
| GO:0007155 | cell adhesion | 3.97e-01 | 1 | 12 | 616 | 14950 | 8.33% | 4.12% | 0.16% | 0.08% | Fraction F up | Actn1 |
| GO:0022610 | biological adhesion | 4.00e-01 | 1 | 12 | 622 | 14950 | 8.33% | 4.16% | 0.16% | 0.08% | Fraction F up | Actn1 |
| GO:0071310 | cellular response to organic substance | 4.11e-01 | 1 | 12 | 644 | 14950 | 8.33% | 4.31% | 0.16% | 0.08% | Fraction F up | Cxcr2 |
| GO:0043933 | macromolecular complex subunit organization | 4.17e-01 | 1 | 12 | 657 | 14950 | 8.33% | 4.39% | 0.15% | 0.08% | Fraction F up | Lcn2 |
| GO:0033554 | cellular response to stress | 4.34e-01 | 1 | 12 | 693 | 14950 | 8.33% | 4.64% | 0.14% | 0.08% | Fraction F up | Lcn2 |
| GO:0051336 | regulation of hydrolase activity | 4.44e-01 | 1 | 12 | 713 | 14950 | 8.33% | 4.77% | 0.14% | 0.08% | Fraction F up | Cxcr2 |
| GO:0045892 | negative regulation of transcription, DNA-dependent | 4.54e-01 | 1 | 12 | 734 | 14950 | 8.33% | 4.91% | 0.14% | 0.08% | Fraction F up | Bhlhe41 |
| GO:0019222 | regulation of metabolic process | 4.57e-01 | 4 | 12 | 4242 | 14950 | 33.33% | 28.37% | 0.09% | 0.08% | Fraction F up | Bhlhe41, Cxcr2, Lcn2, S100a9 |
| GO:0051253 | negative regulation of RNA metabolic process | 4.63e-01 | 1 | 12 | 754 | 14950 | 8.33% | 5.04% | 0.13% | 0.08% | Fraction F up | Bhlhe41 |
| GO:0043085 | positive regulation of catalytic activity | 4.79e-01 | 1 | 12 | 790 | 14950 | 8.33% | 5.28% | 0.13% | 0.08% | Fraction F up | Cxcr2 |
| GO:0045934 | negative regulation of nucleobase-containing compound metabolic process | 4.86e-01 | 1 | 12 | 807 | 14950 | 8.33% | 5.40% | 0.12% | 0.08% | Fraction F up | Bhlhe41 |
| GO:2000113 | negative regulation of cellular macromolecule biosynthetic process | 4.87e-01 | 1 | 12 | 808 | 14950 | 8.33% | 5.40% | 0.12% | 0.08% | Fraction F up | Bhlhe41 |
| GO:0051172 | negative regulation of nitrogen compound metabolic process | 4.90e-01 | 1 | 12 | 816 | 14950 | 8.33% | 5.46% | 0.12% | 0.08% | Fraction F up | Bhlhe41 |
| GO:0010558 | negative regulation of macromolecule biosynthetic process | 4.98e-01 | 1 | 12 | 834 | 14950 | 8.33% | 5.58% | 0.12% | 0.08% | Fraction F up | Bhlhe41 |
| GO:0010629 | negative regulation of gene expression | 4.98e-01 | 1 | 12 | 835 | 14950 | 8.33% | 5.59% | 0.12% | 0.08% | Fraction F up | Bhlhe41 |
| GO:0031327 | negative regulation of cellular biosynthetic process | 5.08e-01 | 1 | 12 | 857 | 14950 | 8.33% | 5.73% | 0.12% | 0.08% | Fraction F up | Bhlhe41 |
| GO:0009890 | negative regulation of biosynthetic process | 5.15e-01 | 1 | 12 | 874 | 14950 | 8.33% | 5.85% | 0.11% | 0.08% | Fraction F up | Bhlhe41 |
| GO:0044093 | positive regulation of molecular function | 5.48e-01 | 1 | 12 | 956 | 14950 | 8.33% | 6.39% | 0.10% | 0.08% | Fraction F up | Cxcr2 |
| GO:0060255 | regulation of macromolecule metabolic process | 5.50e-01 | 3 | 12 | 3460 | 14950 | 25.00% | 23.14% | 0.09% | 0.08% | Fraction F up | Bhlhe41, Lcn2, S100a9 |
| GO:0010628 | positive regulation of gene expression | 5.56e-01 | 1 | 12 | 977 | 14950 | 8.33% | 6.54% | 0.10% | 0.08% | Fraction F up | Lcn2 |
| GO:0042981 | regulation of apoptosis | 5.57e-01 | 1 | 12 | 981 | 14950 | 8.33% | 6.56% | 0.10% | 0.08% | Fraction F up | Lcn2 |
| GO:0042127 | regulation of cell proliferation | 5.58e-01 | 1 | 12 | 982 | 14950 | 8.33% | 6.57% | 0.10% | 0.08% | Fraction F up | Cxcr2 |
| GO:0043067 | regulation of programmed cell death | 5.61e-01 | 1 | 12 | 991 | 14950 | 8.33% | 6.63% | 0.10% | 0.08% | Fraction F up | Lcn2 |
| GO:0048523 | negative regulation of cellular process | 5.66e-01 | 2 | 12 | 2278 | 14950 | 16.67% | 15.24% | 0.09% | 0.08% | Fraction F up | Actn1, Bhlhe41 |
| GO:0035556 | intracellular signal transduction | 5.66e-01 | 1 | 12 | 1004 | 14950 | 8.33% | 6.72% | 0.10% | 0.08% | Fraction F up | Cxcr2 |
| GO:0031324 | negative regulation of cellular metabolic process | 5.74e-01 | 1 | 12 | 1026 | 14950 | 8.33% | 6.86% | 0.10% | 0.08% | Fraction F up | Bhlhe41 |
| GO:0010941 | regulation of cell death | 5.74e-01 | 1 | 12 | 1026 | 14950 | 8.33% | 6.86% | 0.10% | 0.08% | Fraction F up | Lcn2 |
| GO:0006357 | regulation of transcription from RNA polymerase II promoter | 5.77e-01 | 1 | 12 | 1034 | 14950 | 8.33% | 6.92% | 0.10% | 0.08% | Fraction F up | Bhlhe41 |
| GO:0010605 | negative regulation of macromolecule metabolic process | 5.90e-01 | 1 | 12 | 1069 | 14950 | 8.33% | 7.15% | 0.09% | 0.08% | Fraction F up | Bhlhe41 |
| GO:0009892 | negative regulation of metabolic process | 6.17e-01 | 1 | 12 | 1148 | 14950 | 8.33% | 7.68% | 0.09% | 0.08% | Fraction F up | Bhlhe41 |
| GO:0048519 | negative regulation of biological process | 6.28e-01 | 2 | 12 | 2531 | 14950 | 16.67% | 16.93% | 0.08% | 0.08% | Fraction F up | Actn1, Bhlhe41 |
| GO:0010033 | response to organic substance | 6.34e-01 | 1 | 12 | 1200 | 14950 | 8.33% | 8.03% | 0.08% | 0.08% | Fraction F up | Cxcr2 |
| GO:0010556 | regulation of macromolecule biosynthetic process | 6.44e-01 | 2 | 12 | 2598 | 14950 | 16.67% | 17.38% | 0.08% | 0.08% | Fraction F up | Bhlhe41, S100a9 |
| GO:0032879 | regulation of localization | 6.44e-01 | 1 | 12 | 1231 | 14950 | 8.33% | 8.23% | 0.08% | 0.08% | Fraction F up | Actn1 |
| GO:0048522 | positive regulation of cellular process | 6.44e-01 | 2 | 12 | 2602 | 14950 | 16.67% | 17.40% | 0.08% | 0.08% | Fraction F up | Cxcr2, Lcn2 |
| GO:0006810 | transport | 6.48e-01 | 2 | 12 | 2619 | 14950 | 16.67% | 17.52% | 0.08% | 0.08% | Fraction F up | Lcn2, Ltf |
| GO:0051234 | establishment of localization | 6.59e-01 | 2 | 12 | 2668 | 14950 | 16.67% | 17.85% | 0.07% | 0.08% | Fraction F up | Lcn2, Ltf |
| GO:0009056 | catabolic process | 6.70e-01 | 1 | 12 | 1320 | 14950 | 8.33% | 8.83% | 0.08% | 0.08% | Fraction F up | Lyz2 |
| GO:0010468 | regulation of gene expression | 6.75e-01 | 2 | 12 | 2740 | 14950 | 16.67% | 18.33% | 0.07% | 0.08% | Fraction F up | Bhlhe41, Lcn2 |
| GO:0009889 | regulation of biosynthetic process | 6.87e-01 | 2 | 12 | 2798 | 14950 | 16.67% | 18.72% | 0.07% | 0.08% | Fraction F up | Bhlhe41, S100a9 |
| GO:0050790 | regulation of catalytic activity | 6.87e-01 | 1 | 12 | 1380 | 14950 | 8.33% | 9.23% | 0.07% | 0.08% | Fraction F up | Cxcr2 |
| GO:0010604 | positive regulation of macromolecule metabolic process | 6.93e-01 | 1 | 12 | 1399 | 14950 | 8.33% | 9.36% | 0.07% | 0.08% | Fraction F up | Lcn2 |
| GO:0048518 | positive regulation of biological process | 7.02e-01 | 2 | 12 | 2872 | 14950 | 16.67% | 19.21% | 0.07% | 0.08% | Fraction F up | Cxcr2, Lcn2 |
| GO:0009893 | positive regulation of metabolic process | 7.22e-01 | 1 | 12 | 1511 | 14950 | 8.33% | 10.11% | 0.07% | 0.08% | Fraction F up | Lcn2 |
| GO:0006351 | transcription, DNA-dependent | 7.52e-01 | 1 | 12 | 1641 | 14950 | 8.33% | 10.98% | 0.06% | 0.08% | Fraction F up | Bhlhe41 |
| GO:0032774 | RNA biosynthetic process | 7.53e-01 | 1 | 12 | 1643 | 14950 | 8.33% | 10.99% | 0.06% | 0.08% | Fraction F up | Bhlhe41 |
| GO:0065009 | regulation of molecular function | 7.70e-01 | 1 | 12 | 1724 | 14950 | 8.33% | 11.53% | 0.06% | 0.08% | Fraction F up | Cxcr2 |
| GO:0051716 | cellular response to stimulus | 7.76e-01 | 3 | 12 | 4669 | 14950 | 25.00% | 31.23% | 0.06% | 0.08% | Fraction F up | Cxcr2, Lcn2, S100a9 |
| GO:0007186 | G-protein coupled receptor signaling pathway | 8.01e-01 | 1 | 12 | 1879 | 14950 | 8.33% | 12.57% | 0.05% | 0.08% | Fraction F up | Cxcr2 |
| GO:0065007 | biological regulation | 8.04e-01 | 6 | 12 | 8668 | 14950 | 50.00% | 57.98% | 0.07% | 0.08% | Fraction F up | Actn1, Bhlhe41, Cxcr2, Lcn2, Ltf, S100a9 |
| GO:0044238 | primary metabolic process | 8.33e-01 | 4 | 12 | 6425 | 14950 | 33.33% | 42.98% | 0.06% | 0.08% | Fraction F up | Bhlhe41, Cpd, Ltf, Mmp8 |
| GO:0008152 | metabolic process | 8.38e-01 | 5 | 12 | 7737 | 14950 | 41.67% | 51.75% | 0.06% | 0.08% | Fraction F up | Bhlhe41, Cpd, Ltf, Lyz2, Mmp8 |
| GO:0050794 | regulation of cellular process | 8.48e-01 | 5 | 12 | 7819 | 14950 | 41.67% | 52.30% | 0.06% | 0.08% | Fraction F up | Actn1, Bhlhe41, Cxcr2, Lcn2, S100a9 |
| GO:0016070 | RNA metabolic process | 8.53e-01 | 1 | 12 | 2209 | 14950 | 8.33% | 14.78% | 0.05% | 0.08% | Fraction F up | Bhlhe41 |
| GO:0034645 | cellular macromolecule biosynthetic process | 8.58e-01 | 1 | 12 | 2247 | 14950 | 8.33% | 15.03% | 0.04% | 0.08% | Fraction F up | Bhlhe41 |
| GO:0009059 | macromolecule biosynthetic process | 8.62e-01 | 1 | 12 | 2270 | 14950 | 8.33% | 15.18% | 0.04% | 0.08% | Fraction F up | Bhlhe41 |
| GO:0006355 | regulation of transcription, DNA-dependent | 8.68e-01 | 1 | 12 | 2324 | 14950 | 8.33% | 15.55% | 0.04% | 0.08% | Fraction F up | Bhlhe41 |
| GO:2001141 | regulation of RNA biosynthetic process | 8.73e-01 | 1 | 12 | 2358 | 14950 | 8.33% | 15.77% | 0.04% | 0.08% | Fraction F up | Bhlhe41 |
| GO:0010467 | gene expression | 8.79e-01 | 1 | 12 | 2415 | 14950 | 8.33% | 16.15% | 0.04% | 0.08% | Fraction F up | Bhlhe41 |
| GO:0051252 | regulation of RNA metabolic process | 8.80e-01 | 1 | 12 | 2423 | 14950 | 8.33% | 16.21% | 0.04% | 0.08% | Fraction F up | Bhlhe41 |
| GO:2000112 | regulation of cellular macromolecule biosynthetic process | 8.92e-01 | 1 | 12 | 2532 | 14950 | 8.33% | 16.94% | 0.04% | 0.08% | Fraction F up | Bhlhe41 |
| GO:0050789 | regulation of biological process | 8.97e-01 | 5 | 12 | 8321 | 14950 | 41.67% | 55.66% | 0.06% | 0.08% | Fraction F up | Actn1, Bhlhe41, Cxcr2, Lcn2, S100a9 |
| GO:0090304 | nucleic acid metabolic process | 9.02e-01 | 1 | 12 | 2627 | 14950 | 8.33% | 17.57% | 0.04% | 0.08% | Fraction F up | Bhlhe41 |
| GO:0007166 | cell surface receptor linked signaling pathway | 9.14e-01 | 1 | 12 | 2758 | 14950 | 8.33% | 18.45% | 0.04% | 0.08% | Fraction F up | Cxcr2 |
| GO:0031326 | regulation of cellular biosynthetic process | 9.14e-01 | 1 | 12 | 2761 | 14950 | 8.33% | 18.47% | 0.04% | 0.08% | Fraction F up | Bhlhe41 |
| GO:0044249 | cellular biosynthetic process | 9.24e-01 | 1 | 12 | 2890 | 14950 | 8.33% | 19.33% | 0.03% | 0.08% | Fraction F up | Bhlhe41 |
| GO:0019219 | regulation of nucleobase-containing compound metabolic process | 9.25e-01 | 1 | 12 | 2896 | 14950 | 8.33% | 19.37% | 0.03% | 0.08% | Fraction F up | Bhlhe41 |
| GO:0051171 | regulation of nitrogen compound metabolic process | 9.27e-01 | 1 | 12 | 2924 | 14950 | 8.33% | 19.56% | 0.03% | 0.08% | Fraction F up | Bhlhe41 |
| GO:0009058 | biosynthetic process | 9.31e-01 | 1 | 12 | 2990 | 14950 | 8.33% | 20.00% | 0.03% | 0.08% | Fraction F up | Bhlhe41 |
| GO:0006139 | nucleobase-containing compound metabolic process | 9.45e-01 | 1 | 12 | 3202 | 14950 | 8.33% | 21.42% | 0.03% | 0.08% | Fraction F up | Bhlhe41 |
| GO:0009987 | cellular process | 9.52e-01 | 8 | 12 | 12275 | 14950 | 66.67% | 82.11% | 0.07% | 0.08% | Fraction F up | Actn1, Bhlhe41, Cxcr2, Lcn2, Ltf, Lyz2, Pstpip2, S100a9 |
| GO:0034641 | cellular nitrogen compound metabolic process | 9.60e-01 | 1 | 12 | 3523 | 14950 | 8.33% | 23.57% | 0.03% | 0.08% | Fraction F up | Bhlhe41 |
| GO:0006807 | nitrogen compound metabolic process | 9.64e-01 | 1 | 12 | 3614 | 14950 | 8.33% | 24.17% | 0.03% | 0.08% | Fraction F up | Bhlhe41 |
| GO:0031323 | regulation of cellular metabolic process | 9.66e-01 | 1 | 12 | 3659 | 14950 | 8.33% | 24.47% | 0.03% | 0.08% | Fraction F up | Bhlhe41 |
| GO:0080090 | regulation of primary metabolic process | 9.66e-01 | 1 | 12 | 3662 | 14950 | 8.33% | 24.49% | 0.03% | 0.08% | Fraction F up | Bhlhe41 |
| GO:0007165 | signal transduction | 9.73e-01 | 1 | 12 | 3899 | 14950 | 8.33% | 26.08% | 0.03% | 0.08% | Fraction F up | Cxcr2 |
| GO:0023052 | signaling | 9.80e-01 | 1 | 12 | 4165 | 14950 | 8.33% | 27.86% | 0.02% | 0.08% | Fraction F up | Cxcr2 |
| GO:0007154 | cell communication | 9.83e-01 | 1 | 12 | 4281 | 14950 | 8.33% | 28.64% | 0.02% | 0.08% | Fraction F up | Cxcr2 |
| GO:0044260 | cellular macromolecule metabolic process | 9.87e-01 | 1 | 12 | 4513 | 14950 | 8.33% | 30.19% | 0.02% | 0.08% | Fraction F up | Bhlhe41 |
| GO:0032501 | multicellular organismal process | 9.94e-01 | 1 | 12 | 5143 | 14950 | 8.33% | 34.40% | 0.02% | 0.08% | Fraction F up | Mmp8 |
| GO:0044237 | cellular metabolic process | 9.99e-01 | 1 | 12 | 6337 | 14950 | 8.33% | 42.39% | 0.02% | 0.08% | Fraction F up | Bhlhe41 |
| GO:0008150 | biological\_process | 1.00e+00 | 12 | 12 | 14950 | 14950 | 100.00% | 100.00% | 0.08% | 0.08% | Fraction F up | Actn1, Bhlhe41, Cpd, Cxcr2, Igj, Lcn2, Ltf, Lyz2, Mmp8, Pstpip2, S100a8, S100a9 |

### cellular\_component (top)

  
  


| TermID | Term | Pval | k | n | K | N | k/n | K/N | k/K | n/N | Qset | Symbols |
| --- | --- | --- | --- | --- | --- | --- | --- | --- | --- | --- | --- | --- |
| GO:0005576 | extracellular region | 2.84e-04 | 7 | 14 | 1735 | 16137 | 50.00% | 10.75% | 0.40% | 0.09% | Fraction F up | Igj, Lcn2, Ltf, Lyz2, Mmp8, S100a8, S100a9 |
| GO:0030141 | stored secretory granule | 7.72e-04 | 3 | 14 | 216 | 16137 | 21.43% | 1.34% | 1.39% | 0.09% | Fraction F up | Actn1, Ltf, Lyz2 |
| GO:0031410 | cytoplasmic vesicle | 2.56e-03 | 4 | 14 | 706 | 16137 | 28.57% | 4.38% | 0.57% | 0.09% | Fraction F up | Actn1, Ltf, Lyz2, Ngp |
| GO:0032127 | dense core granule membrane | 2.60e-03 | 1 | 14 | 3 | 16137 | 7.14% | 0.02% | 33.33% | 0.09% | Fraction F up | Actn1 |
| GO:0042629 | mast cell granule | 2.60e-03 | 1 | 14 | 3 | 16137 | 7.14% | 0.02% | 33.33% | 0.09% | Fraction F up | Cxcr2 |
| GO:0048237 | rough endoplasmic reticulum lumen | 2.60e-03 | 1 | 14 | 3 | 16137 | 7.14% | 0.02% | 33.33% | 0.09% | Fraction F up | Lyz2 |
| GO:0005615 | extracellular space | 3.19e-03 | 4 | 14 | 750 | 16137 | 28.57% | 4.65% | 0.53% | 0.09% | Fraction F up | Lcn2, Mmp8, S100a8, S100a9 |
| GO:0031982 | vesicle | 3.35e-03 | 4 | 14 | 760 | 16137 | 28.57% | 4.71% | 0.53% | 0.09% | Fraction F up | Actn1, Ltf, Lyz2, Ngp |
| GO:0000137 | Golgi cis cisterna | 5.19e-03 | 1 | 14 | 6 | 16137 | 7.14% | 0.04% | 16.67% | 0.09% | Fraction F up | Lyz2 |
| GO:0031045 | dense core granule | 7.78e-03 | 1 | 14 | 9 | 16137 | 7.14% | 0.06% | 11.11% | 0.09% | Fraction F up | Actn1 |
| GO:0016023 | cytoplasmic membrane-bounded vesicle | 8.76e-03 | 3 | 14 | 509 | 16137 | 21.43% | 3.15% | 0.59% | 0.09% | Fraction F up | Actn1, Ltf, Lyz2 |
| GO:0044421 | extracellular region part | 9.02e-03 | 4 | 14 | 1004 | 16137 | 28.57% | 6.22% | 0.40% | 0.09% | Fraction F up | Lcn2, Mmp8, S100a8, S100a9 |
| GO:0031988 | membrane-bounded vesicle | 1.04e-02 | 3 | 14 | 542 | 16137 | 21.43% | 3.36% | 0.55% | 0.09% | Fraction F up | Actn1, Ltf, Lyz2 |
| GO:0005916 | fascia adherens | 1.12e-02 | 1 | 14 | 13 | 16137 | 7.14% | 0.08% | 7.69% | 0.09% | Fraction F up | Actn1 |
| GO:0030140 | trans-Golgi network transport vesicle | 1.64e-02 | 1 | 14 | 19 | 16137 | 7.14% | 0.12% | 5.26% | 0.09% | Fraction F up | Lyz2 |
| GO:0031985 | Golgi cisterna | 2.15e-02 | 1 | 14 | 25 | 16137 | 7.14% | 0.15% | 4.00% | 0.09% | Fraction F up | Lyz2 |
| GO:0014704 | intercalated disc | 2.23e-02 | 1 | 14 | 26 | 16137 | 7.14% | 0.16% | 3.85% | 0.09% | Fraction F up | Actn1 |
| GO:0031984 | organelle subcompartment | 2.23e-02 | 1 | 14 | 26 | 16137 | 7.14% | 0.16% | 3.85% | 0.09% | Fraction F up | Lyz2 |
| GO:0044291 | cell-cell contact zone | 2.40e-02 | 1 | 14 | 28 | 16137 | 7.14% | 0.17% | 3.57% | 0.09% | Fraction F up | Actn1 |
| GO:0030864 | cortical actin cytoskeleton | 2.49e-02 | 1 | 14 | 29 | 16137 | 7.14% | 0.18% | 3.45% | 0.09% | Fraction F up | Actn1 |
| GO:0044431 | Golgi apparatus part | 2.49e-02 | 2 | 14 | 287 | 16137 | 14.29% | 1.78% | 0.70% | 0.09% | Fraction F up | Cpd, Lyz2 |
| GO:0005788 | endoplasmic reticulum lumen | 2.66e-02 | 1 | 14 | 31 | 16137 | 7.14% | 0.19% | 3.23% | 0.09% | Fraction F up | Lyz2 |
| GO:0005795 | Golgi stack | 3.58e-02 | 1 | 14 | 42 | 16137 | 7.14% | 0.26% | 2.38% | 0.09% | Fraction F up | Lyz2 |
| GO:0005791 | rough endoplasmic reticulum | 3.75e-02 | 1 | 14 | 44 | 16137 | 7.14% | 0.27% | 2.27% | 0.09% | Fraction F up | Lyz2 |
| GO:0030667 | secretory granule membrane | 3.75e-02 | 1 | 14 | 44 | 16137 | 7.14% | 0.27% | 2.27% | 0.09% | Fraction F up | Actn1 |
| GO:0005856 | cytoskeleton | 3.94e-02 | 4 | 14 | 1558 | 16137 | 28.57% | 9.65% | 0.26% | 0.09% | Fraction F up | Actn1, Pstpip2, S100a8, S100a9 |
| GO:0005798 | Golgi-associated vesicle | 4.09e-02 | 1 | 14 | 48 | 16137 | 7.14% | 0.30% | 2.08% | 0.09% | Fraction F up | Lyz2 |
| GO:0001725 | stress fiber | 4.09e-02 | 1 | 14 | 48 | 16137 | 7.14% | 0.30% | 2.08% | 0.09% | Fraction F up | Actn1 |
| GO:0005913 | cell-cell adherens junction | 4.25e-02 | 1 | 14 | 50 | 16137 | 7.14% | 0.31% | 2.00% | 0.09% | Fraction F up | Actn1 |
| GO:0032432 | actin filament bundle | 4.34e-02 | 1 | 14 | 51 | 16137 | 7.14% | 0.32% | 1.96% | 0.09% | Fraction F up | Actn1 |
| GO:0017053 | transcriptional repressor complex | 4.42e-02 | 1 | 14 | 52 | 16137 | 7.14% | 0.32% | 1.92% | 0.09% | Fraction F up | Bhlhe41 |
| GO:0030863 | cortical cytoskeleton | 4.75e-02 | 1 | 14 | 56 | 16137 | 7.14% | 0.35% | 1.79% | 0.09% | Fraction F up | Actn1 |
| GO:0042641 | actomyosin | 4.84e-02 | 1 | 14 | 57 | 16137 | 7.14% | 0.35% | 1.75% | 0.09% | Fraction F up | Actn1 |
| GO:0044444 | cytoplasmic part | 4.97e-02 | 8 | 14 | 5241 | 16137 | 57.14% | 32.48% | 0.15% | 0.09% | Fraction F up | Actn1, Cpd, Cxcr2, Lcn2, Ltf, Lyz2, Ngp, Pstpip2 |
| GO:0030133 | transport vesicle | 5.00e-02 | 1 | 14 | 59 | 16137 | 7.14% | 0.37% | 1.69% | 0.09% | Fraction F up | Lyz2 |
| GO:0030018 | Z disc | 5.08e-02 | 1 | 14 | 60 | 16137 | 7.14% | 0.37% | 1.67% | 0.09% | Fraction F up | Actn1 |
| GO:0005902 | microvillus | 5.08e-02 | 1 | 14 | 60 | 16137 | 7.14% | 0.37% | 1.67% | 0.09% | Fraction F up | Lyz2 |
| GO:0031674 | I band | 5.99e-02 | 1 | 14 | 71 | 16137 | 7.14% | 0.44% | 1.41% | 0.09% | Fraction F up | Actn1 |
| GO:0009986 | cell surface | 7.11e-02 | 2 | 14 | 512 | 16137 | 14.29% | 3.17% | 0.39% | 0.09% | Fraction F up | Cxcr2, Ly6g |
| GO:0005925 | focal adhesion | 7.13e-02 | 1 | 14 | 85 | 16137 | 7.14% | 0.53% | 1.18% | 0.09% | Fraction F up | Actn1 |
| GO:0044448 | cell cortex part | 7.37e-02 | 1 | 14 | 88 | 16137 | 7.14% | 0.55% | 1.14% | 0.09% | Fraction F up | Actn1 |
| GO:0005924 | cell-substrate adherens junction | 7.53e-02 | 1 | 14 | 90 | 16137 | 7.14% | 0.56% | 1.11% | 0.09% | Fraction F up | Actn1 |
| GO:0000267 | cell fraction | 7.70e-02 | 3 | 14 | 1177 | 16137 | 21.43% | 7.29% | 0.25% | 0.09% | Fraction F up | Cpd, Lyz2, Pstpip2 |
| GO:0005802 | trans-Golgi network | 7.78e-02 | 1 | 14 | 93 | 16137 | 7.14% | 0.58% | 1.08% | 0.09% | Fraction F up | Cpd |
| GO:0030055 | cell-substrate junction | 8.02e-02 | 1 | 14 | 96 | 16137 | 7.14% | 0.59% | 1.04% | 0.09% | Fraction F up | Actn1 |
| GO:0005737 | cytoplasm | 8.29e-02 | 10 | 14 | 7976 | 16137 | 71.43% | 49.43% | 0.13% | 0.09% | Fraction F up | Actn1, Cpd, Cxcr2, Lcn2, Ltf, Lyz2, Ngp, Pstpip2, S100a8, S100a9 |
| GO:0030017 | sarcomere | 8.42e-02 | 1 | 14 | 101 | 16137 | 7.14% | 0.63% | 0.99% | 0.09% | Fraction F up | Actn1 |
| GO:0044449 | contractile fiber part | 9.29e-02 | 1 | 14 | 112 | 16137 | 7.14% | 0.69% | 0.89% | 0.09% | Fraction F up | Actn1 |
| GO:0030016 | myofibril | 1.02e-01 | 1 | 14 | 124 | 16137 | 7.14% | 0.77% | 0.81% | 0.09% | Fraction F up | Actn1 |
| GO:0030659 | cytoplasmic vesicle membrane | 1.03e-01 | 1 | 14 | 125 | 16137 | 7.14% | 0.77% | 0.80% | 0.09% | Fraction F up | Actn1 |
| GO:0044433 | cytoplasmic vesicle part | 1.08e-01 | 1 | 14 | 131 | 16137 | 7.14% | 0.81% | 0.76% | 0.09% | Fraction F up | Actn1 |
| GO:0031225 | anchored to membrane | 1.09e-01 | 1 | 14 | 133 | 16137 | 7.14% | 0.82% | 0.75% | 0.09% | Fraction F up | Ly6g |
| GO:0043292 | contractile fiber | 1.11e-01 | 1 | 14 | 135 | 16137 | 7.14% | 0.84% | 0.74% | 0.09% | Fraction F up | Actn1 |
| GO:0005912 | adherens junction | 1.15e-01 | 1 | 14 | 140 | 16137 | 7.14% | 0.87% | 0.71% | 0.09% | Fraction F up | Actn1 |
| GO:0043197 | dendritic spine | 1.18e-01 | 1 | 14 | 144 | 16137 | 7.14% | 0.89% | 0.69% | 0.09% | Fraction F up | Actn1 |
| GO:0044309 | neuron spine | 1.18e-01 | 1 | 14 | 144 | 16137 | 7.14% | 0.89% | 0.69% | 0.09% | Fraction F up | Actn1 |
| GO:0012506 | vesicle membrane | 1.20e-01 | 1 | 14 | 146 | 16137 | 7.14% | 0.90% | 0.68% | 0.09% | Fraction F up | Actn1 |
| GO:0070013 | intracellular organelle lumen | 1.24e-01 | 3 | 14 | 1445 | 16137 | 21.43% | 8.95% | 0.21% | 0.09% | Fraction F up | Actn1, Bhlhe41, Lyz2 |
| GO:0043233 | organelle lumen | 1.25e-01 | 3 | 14 | 1449 | 16137 | 21.43% | 8.98% | 0.21% | 0.09% | Fraction F up | Actn1, Bhlhe41, Lyz2 |
| GO:0070161 | anchoring junction | 1.25e-01 | 1 | 14 | 153 | 16137 | 7.14% | 0.95% | 0.65% | 0.09% | Fraction F up | Actn1 |
| GO:0030136 | clathrin-coated vesicle | 1.26e-01 | 1 | 14 | 154 | 16137 | 7.14% | 0.95% | 0.65% | 0.09% | Fraction F up | Lyz2 |
| GO:0005938 | cell cortex | 1.29e-01 | 1 | 14 | 158 | 16137 | 7.14% | 0.98% | 0.63% | 0.09% | Fraction F up | Actn1 |
| GO:0031974 | membrane-enclosed lumen | 1.33e-01 | 3 | 14 | 1490 | 16137 | 21.43% | 9.23% | 0.20% | 0.09% | Fraction F up | Actn1, Bhlhe41, Lyz2 |
| GO:0030135 | coated vesicle | 1.42e-01 | 1 | 14 | 176 | 16137 | 7.14% | 1.09% | 0.57% | 0.09% | Fraction F up | Lyz2 |
| GO:0005886 | plasma membrane | 1.59e-01 | 5 | 14 | 3436 | 16137 | 35.71% | 21.29% | 0.15% | 0.09% | Fraction F up | Actn1, Cxcr2, Ly6g, S100a8, S100a9 |
| GO:0005624 | membrane fraction | 1.67e-01 | 2 | 14 | 851 | 16137 | 14.29% | 5.27% | 0.24% | 0.09% | Fraction F up | Cpd, Pstpip2 |
| GO:0071944 | cell periphery | 1.72e-01 | 5 | 14 | 3519 | 16137 | 35.71% | 21.81% | 0.14% | 0.09% | Fraction F up | Actn1, Cxcr2, Ly6g, S100a8, S100a9 |
| GO:0005626 | insoluble fraction | 1.81e-01 | 2 | 14 | 897 | 16137 | 14.29% | 5.56% | 0.22% | 0.09% | Fraction F up | Cpd, Pstpip2 |
| GO:0005829 | cytosol | 1.83e-01 | 2 | 14 | 902 | 16137 | 14.29% | 5.59% | 0.22% | 0.09% | Fraction F up | Lcn2, Pstpip2 |
| GO:0043232 | intracellular non-membrane-bounded organelle | 1.83e-01 | 4 | 14 | 2632 | 16137 | 28.57% | 16.31% | 0.15% | 0.09% | Fraction F up | Actn1, Pstpip2, S100a8, S100a9 |
| GO:0043228 | non-membrane-bounded organelle | 1.83e-01 | 4 | 14 | 2632 | 16137 | 28.57% | 16.31% | 0.15% | 0.09% | Fraction F up | Actn1, Pstpip2, S100a8, S100a9 |
| GO:0016323 | basolateral plasma membrane | 1.87e-01 | 1 | 14 | 237 | 16137 | 7.14% | 1.47% | 0.42% | 0.09% | Fraction F up | Actn1 |
| GO:0009897 | external side of plasma membrane | 1.87e-01 | 1 | 14 | 237 | 16137 | 7.14% | 1.47% | 0.42% | 0.09% | Fraction F up | Ly6g |
| GO:0044432 | endoplasmic reticulum part | 1.93e-01 | 1 | 14 | 245 | 16137 | 7.14% | 1.52% | 0.41% | 0.09% | Fraction F up | Lyz2 |
| GO:0005764 | lysosome | 1.93e-01 | 1 | 14 | 245 | 16137 | 7.14% | 1.52% | 0.41% | 0.09% | Fraction F up | Cxcr2 |
| GO:0000323 | lytic vacuole | 1.93e-01 | 1 | 14 | 245 | 16137 | 7.14% | 1.52% | 0.41% | 0.09% | Fraction F up | Cxcr2 |
| GO:0005911 | cell-cell junction | 1.96e-01 | 1 | 14 | 250 | 16137 | 7.14% | 1.55% | 0.40% | 0.09% | Fraction F up | Actn1 |
| GO:0005794 | Golgi apparatus | 1.97e-01 | 2 | 14 | 946 | 16137 | 14.29% | 5.86% | 0.21% | 0.09% | Fraction F up | Cpd, Lyz2 |
| GO:0005792 | microsome | 2.04e-01 | 1 | 14 | 261 | 16137 | 7.14% | 1.62% | 0.38% | 0.09% | Fraction F up | Cpd |
| GO:0042598 | vesicular fraction | 2.10e-01 | 1 | 14 | 269 | 16137 | 7.14% | 1.67% | 0.37% | 0.09% | Fraction F up | Cpd |
| GO:0043229 | intracellular organelle | 2.10e-01 | 10 | 14 | 9216 | 16137 | 71.43% | 57.11% | 0.11% | 0.09% | Fraction F up | Actn1, Bhlhe41, Cpd, Cxcr2, Ltf, Lyz2, Ngp, Pstpip2, S100a8, S100a9 |
| GO:0043226 | organelle | 2.14e-01 | 10 | 14 | 9241 | 16137 | 71.43% | 57.27% | 0.11% | 0.09% | Fraction F up | Actn1, Bhlhe41, Cpd, Cxcr2, Ltf, Lyz2, Ngp, Pstpip2, S100a8, S100a9 |
| GO:0005773 | vacuole | 2.26e-01 | 1 | 14 | 292 | 16137 | 7.14% | 1.81% | 0.34% | 0.09% | Fraction F up | Cxcr2 |
| GO:0015629 | actin cytoskeleton | 2.28e-01 | 1 | 14 | 296 | 16137 | 7.14% | 1.83% | 0.34% | 0.09% | Fraction F up | Actn1 |
| GO:0005578 | proteinaceous extracellular matrix | 2.47e-01 | 1 | 14 | 324 | 16137 | 7.14% | 2.01% | 0.31% | 0.09% | Fraction F up | Mmp8 |
| GO:0044424 | intracellular part | 2.49e-01 | 11 | 14 | 10684 | 16137 | 78.57% | 66.21% | 0.10% | 0.09% | Fraction F up | Actn1, Bhlhe41, Cpd, Cxcr2, Lcn2, Ltf, Lyz2, Ngp, Pstpip2, S100a8, S100a9 |
| GO:0030425 | dendrite | 2.70e-01 | 1 | 14 | 358 | 16137 | 7.14% | 2.22% | 0.28% | 0.09% | Fraction F up | Actn1 |
| GO:0031012 | extracellular matrix | 2.74e-01 | 1 | 14 | 364 | 16137 | 7.14% | 2.26% | 0.27% | 0.09% | Fraction F up | Mmp8 |
| GO:0042995 | cell projection | 2.80e-01 | 2 | 14 | 1201 | 16137 | 14.29% | 7.44% | 0.17% | 0.09% | Fraction F up | Actn1, Lyz2 |
| GO:0005730 | nucleolus | 2.86e-01 | 1 | 14 | 383 | 16137 | 7.14% | 2.37% | 0.26% | 0.09% | Fraction F up | Actn1 |
| GO:0005622 | intracellular | 2.89e-01 | 11 | 14 | 10928 | 16137 | 78.57% | 67.72% | 0.10% | 0.09% | Fraction F up | Actn1, Bhlhe41, Cpd, Cxcr2, Lcn2, Ltf, Lyz2, Ngp, Pstpip2, S100a8, S100a9 |
| GO:0005625 | soluble fraction | 2.89e-01 | 1 | 14 | 389 | 16137 | 7.14% | 2.41% | 0.26% | 0.09% | Fraction F up | Lyz2 |
| GO:0031981 | nuclear lumen | 3.03e-01 | 2 | 14 | 1271 | 16137 | 14.29% | 7.88% | 0.16% | 0.09% | Fraction F up | Actn1, Bhlhe41 |
| GO:0044463 | cell projection part | 3.68e-01 | 1 | 14 | 520 | 16137 | 7.14% | 3.22% | 0.19% | 0.09% | Fraction F up | Actn1 |
| GO:0044459 | plasma membrane part | 3.85e-01 | 2 | 14 | 1518 | 16137 | 14.29% | 9.41% | 0.13% | 0.09% | Fraction F up | Actn1, Ly6g |
| GO:0044428 | nuclear part | 4.18e-01 | 2 | 14 | 1621 | 16137 | 14.29% | 10.05% | 0.12% | 0.09% | Fraction F up | Actn1, Bhlhe41 |
| GO:0043005 | neuron projection | 4.39e-01 | 1 | 14 | 652 | 16137 | 7.14% | 4.04% | 0.15% | 0.09% | Fraction F up | Actn1 |
| GO:0030054 | cell junction | 4.39e-01 | 1 | 14 | 653 | 16137 | 7.14% | 4.05% | 0.15% | 0.09% | Fraction F up | Actn1 |
| GO:0012505 | endomembrane system | 4.53e-01 | 1 | 14 | 681 | 16137 | 7.14% | 4.22% | 0.15% | 0.09% | Fraction F up | Actn1 |
| GO:0044451 | nucleoplasm part | 4.59e-01 | 1 | 14 | 693 | 16137 | 7.14% | 4.29% | 0.14% | 0.09% | Fraction F up | Bhlhe41 |
| GO:0044446 | intracellular organelle part | 4.60e-01 | 4 | 14 | 3949 | 16137 | 28.57% | 24.47% | 0.10% | 0.09% | Fraction F up | Actn1, Bhlhe41, Cpd, Lyz2 |
| GO:0044422 | organelle part | 4.78e-01 | 4 | 14 | 4031 | 16137 | 28.57% | 24.98% | 0.10% | 0.09% | Fraction F up | Actn1, Bhlhe41, Cpd, Lyz2 |
| GO:0005654 | nucleoplasm | 5.01e-01 | 1 | 14 | 781 | 16137 | 7.14% | 4.84% | 0.13% | 0.09% | Fraction F up | Bhlhe41 |
| GO:0016020 | membrane | 5.74e-01 | 7 | 14 | 7901 | 16137 | 50.00% | 48.96% | 0.09% | 0.09% | Fraction F up | Actn1, Cpd, Cxcr2, Ly6g, Pstpip2, S100a8, S100a9 |
| GO:0005783 | endoplasmic reticulum | 6.07e-01 | 1 | 14 | 1040 | 16137 | 7.14% | 6.44% | 0.10% | 0.09% | Fraction F up | Lyz2 |
| GO:0044430 | cytoskeletal part | 6.10e-01 | 1 | 14 | 1048 | 16137 | 7.14% | 6.49% | 0.10% | 0.09% | Fraction F up | Actn1 |
| GO:0043231 | intracellular membrane-bounded organelle | 6.17e-01 | 7 | 14 | 8134 | 16137 | 50.00% | 50.41% | 0.09% | 0.09% | Fraction F up | Actn1, Bhlhe41, Cpd, Cxcr2, Ltf, Lyz2, S100a9 |
| GO:0043227 | membrane-bounded organelle | 6.20e-01 | 7 | 14 | 8150 | 16137 | 50.00% | 50.51% | 0.09% | 0.09% | Fraction F up | Actn1, Bhlhe41, Cpd, Cxcr2, Ltf, Lyz2, S100a9 |
| GO:0031090 | organelle membrane | 6.20e-01 | 1 | 14 | 1078 | 16137 | 7.14% | 6.68% | 0.09% | 0.09% | Fraction F up | Actn1 |
| GO:0005634 | nucleus | 6.54e-01 | 4 | 14 | 4887 | 16137 | 28.57% | 30.28% | 0.08% | 0.09% | Fraction F up | Actn1, Bhlhe41, Cpd, S100a9 |
| GO:0044425 | membrane part | 8.77e-01 | 4 | 14 | 6469 | 16137 | 28.57% | 40.09% | 0.06% | 0.09% | Fraction F up | Actn1, Cpd, Cxcr2, Ly6g |
| GO:0031224 | intrinsic to membrane | 9.26e-01 | 3 | 14 | 5792 | 16137 | 21.43% | 35.89% | 0.05% | 0.09% | Fraction F up | Cpd, Cxcr2, Ly6g |
| GO:0043234 | protein complex | 9.34e-01 | 1 | 14 | 2844 | 16137 | 7.14% | 17.62% | 0.04% | 0.09% | Fraction F up | Bhlhe41 |
| GO:0005623 | cell | 9.58e-01 | 12 | 14 | 15217 | 16137 | 85.71% | 94.30% | 0.08% | 0.09% | Fraction F up | Actn1, Bhlhe41, Cpd, Cxcr2, Lcn2, Ltf, Ly6g, Lyz2, Ngp, Pstpip2, S100a8, S100a9 |
| GO:0044464 | cell part | 9.58e-01 | 12 | 14 | 15217 | 16137 | 85.71% | 94.30% | 0.08% | 0.09% | Fraction F up | Actn1, Bhlhe41, Cpd, Cxcr2, Lcn2, Ltf, Ly6g, Lyz2, Ngp, Pstpip2, S100a8, S100a9 |
| GO:0032991 | macromolecular complex | 9.65e-01 | 1 | 14 | 3438 | 16137 | 7.14% | 21.31% | 0.03% | 0.09% | Fraction F up | Bhlhe41 |
| GO:0016021 | integral to membrane | 9.80e-01 | 2 | 14 | 5660 | 16137 | 14.29% | 35.07% | 0.04% | 0.09% | Fraction F up | Cpd, Cxcr2 |
| GO:0005575 | cellular\_component | 1.00e+00 | 14 | 14 | 16137 | 16137 | 100.00% | 100.00% | 0.09% | 0.09% | Fraction F up | Actn1, Bhlhe41, Cpd, Cxcr2, Igj, Lcn2, Ltf, Ly6g, Lyz2, Mmp8, Ngp, Pstpip2, S100a8, S100a9 |

### molecular\_function (top)

  
  


| TermID | Term | Pval | k | n | K | N | k/n | K/N | k/K | n/N | Qset | Symbols |
| --- | --- | --- | --- | --- | --- | --- | --- | --- | --- | --- | --- | --- |
| GO:0008236 | serine-type peptidase activity | 5.00e-04 | 3 | 12 | 201 | 14763 | 25.00% | 1.36% | 1.49% | 0.08% | Fraction F up | Cpd, Ltf, Mmp8 |
| GO:0017171 | serine hydrolase activity | 5.22e-04 | 3 | 12 | 204 | 14763 | 25.00% | 1.38% | 1.47% | 0.08% | Fraction F up | Cpd, Ltf, Mmp8 |
| GO:0005509 | calcium ion binding | 5.30e-04 | 4 | 12 | 503 | 14763 | 33.33% | 3.41% | 0.80% | 0.08% | Fraction F up | Actn1, Mmp8, S100a8, S100a9 |
| GO:0004918 | interleukin-8 receptor activity | 1.63e-03 | 1 | 12 | 2 | 14763 | 8.33% | 0.01% | 50.00% | 0.08% | Fraction F up | Cxcr2 |
| GO:0019958 | C-X-C chemokine binding | 2.44e-03 | 1 | 12 | 3 | 14763 | 8.33% | 0.02% | 33.33% | 0.08% | Fraction F up | Cxcr2 |
| GO:0019959 | interleukin-8 binding | 2.44e-03 | 1 | 12 | 3 | 14763 | 8.33% | 0.02% | 33.33% | 0.08% | Fraction F up | Cxcr2 |
| GO:0004185 | serine-type carboxypeptidase activity | 3.25e-03 | 1 | 12 | 4 | 14763 | 8.33% | 0.03% | 25.00% | 0.08% | Fraction F up | Cpd |
| GO:0016494 | C-X-C chemokine receptor activity | 6.49e-03 | 1 | 12 | 8 | 14763 | 8.33% | 0.05% | 12.50% | 0.08% | Fraction F up | Cxcr2 |
| GO:0008199 | ferric iron binding | 7.29e-03 | 1 | 12 | 9 | 14763 | 8.33% | 0.06% | 11.11% | 0.08% | Fraction F up | Ltf |
| GO:0003796 | lysozyme activity | 7.29e-03 | 1 | 12 | 9 | 14763 | 8.33% | 0.06% | 11.11% | 0.08% | Fraction F up | Lyz2 |
| GO:0070008 | serine-type exopeptidase activity | 7.29e-03 | 1 | 12 | 9 | 14763 | 8.33% | 0.06% | 11.11% | 0.08% | Fraction F up | Cpd |
| GO:0017166 | vinculin binding | 8.10e-03 | 1 | 12 | 10 | 14763 | 8.33% | 0.07% | 10.00% | 0.08% | Fraction F up | Actn1 |
| GO:0008237 | metallopeptidase activity | 8.16e-03 | 2 | 12 | 171 | 14763 | 16.67% | 1.16% | 1.17% | 0.08% | Fraction F up | Cpd, Mmp8 |
| GO:0046872 | metal ion binding | 8.34e-03 | 7 | 12 | 3362 | 14763 | 58.33% | 22.77% | 0.21% | 0.08% | Fraction F up | Actn1, Cpd, Lcn2, Ltf, Mmp8, S100a8, S100a9 |
| GO:0019956 | chemokine binding | 8.91e-03 | 1 | 12 | 11 | 14763 | 8.33% | 0.07% | 9.09% | 0.08% | Fraction F up | Cxcr2 |
| GO:0043169 | cation binding | 8.92e-03 | 7 | 12 | 3401 | 14763 | 58.33% | 23.04% | 0.21% | 0.08% | Fraction F up | Actn1, Cpd, Lcn2, Ltf, Mmp8, S100a8, S100a9 |
| GO:0043167 | ion binding | 9.10e-03 | 7 | 12 | 3413 | 14763 | 58.33% | 23.12% | 0.21% | 0.08% | Fraction F up | Actn1, Cpd, Lcn2, Ltf, Mmp8, S100a8, S100a9 |
| GO:0070011 | peptidase activity, acting on L-amino acid peptides | 9.56e-03 | 3 | 12 | 567 | 14763 | 25.00% | 3.84% | 0.53% | 0.08% | Fraction F up | Cpd, Ltf, Mmp8 |
| GO:0005506 | iron ion binding | 1.05e-02 | 2 | 12 | 195 | 14763 | 16.67% | 1.32% | 1.03% | 0.08% | Fraction F up | Lcn2, Ltf |
| GO:0008233 | peptidase activity | 1.10e-02 | 3 | 12 | 596 | 14763 | 25.00% | 4.04% | 0.50% | 0.08% | Fraction F up | Cpd, Ltf, Mmp8 |
| GO:0004181 | metallocarboxypeptidase activity | 1.69e-02 | 1 | 12 | 21 | 14763 | 8.33% | 0.14% | 4.76% | 0.08% | Fraction F up | Cpd |
| GO:0001637 | G-protein coupled chemoattractant receptor activity | 1.85e-02 | 1 | 12 | 23 | 14763 | 8.33% | 0.16% | 4.35% | 0.08% | Fraction F up | Cxcr2 |
| GO:0004950 | chemokine receptor activity | 1.85e-02 | 1 | 12 | 23 | 14763 | 8.33% | 0.16% | 4.35% | 0.08% | Fraction F up | Cxcr2 |
| GO:0003779 | actin binding | 2.42e-02 | 2 | 12 | 303 | 14763 | 16.67% | 2.05% | 0.66% | 0.08% | Fraction F up | Actn1, Pstpip2 |
| GO:0008235 | metalloexopeptidase activity | 2.57e-02 | 1 | 12 | 32 | 14763 | 8.33% | 0.22% | 3.12% | 0.08% | Fraction F up | Cpd |
| GO:0004180 | carboxypeptidase activity | 2.81e-02 | 1 | 12 | 35 | 14763 | 8.33% | 0.24% | 2.86% | 0.08% | Fraction F up | Cpd |
| GO:0003823 | antigen binding | 3.05e-02 | 1 | 12 | 38 | 14763 | 8.33% | 0.26% | 2.63% | 0.08% | Fraction F up | Igj |
| GO:0046914 | transition metal ion binding | 3.17e-02 | 4 | 12 | 1576 | 14763 | 33.33% | 10.68% | 0.25% | 0.08% | Fraction F up | Cpd, Lcn2, Ltf, Mmp8 |
| GO:0019955 | cytokine binding | 4.15e-02 | 1 | 12 | 52 | 14763 | 8.33% | 0.35% | 1.92% | 0.08% | Fraction F up | Cxcr2 |
| GO:0002020 | protease binding | 4.23e-02 | 1 | 12 | 53 | 14763 | 8.33% | 0.36% | 1.89% | 0.08% | Fraction F up | Lcn2 |
| GO:0051015 | actin filament binding | 4.85e-02 | 1 | 12 | 61 | 14763 | 8.33% | 0.41% | 1.64% | 0.08% | Fraction F up | Actn1 |
| GO:0005178 | integrin binding | 5.47e-02 | 1 | 12 | 69 | 14763 | 8.33% | 0.47% | 1.45% | 0.08% | Fraction F up | Actn1 |
| GO:0004896 | cytokine receptor activity | 5.62e-02 | 1 | 12 | 71 | 14763 | 8.33% | 0.48% | 1.41% | 0.08% | Fraction F up | Cxcr2 |
| GO:0042803 | protein homodimerization activity | 6.34e-02 | 2 | 12 | 514 | 14763 | 16.67% | 3.48% | 0.39% | 0.08% | Fraction F up | Actn1, Lcn2 |
| GO:0008092 | cytoskeletal protein binding | 6.69e-02 | 2 | 12 | 530 | 14763 | 16.67% | 3.59% | 0.38% | 0.08% | Fraction F up | Actn1, Pstpip2 |
| GO:0004553 | hydrolase activity, hydrolyzing O-glycosyl compounds | 6.70e-02 | 1 | 12 | 85 | 14763 | 8.33% | 0.58% | 1.18% | 0.08% | Fraction F up | Lyz2 |
| GO:0008238 | exopeptidase activity | 6.77e-02 | 1 | 12 | 86 | 14763 | 8.33% | 0.58% | 1.16% | 0.08% | Fraction F up | Cpd |
| GO:0004222 | metalloendopeptidase activity | 7.76e-02 | 1 | 12 | 99 | 14763 | 8.33% | 0.67% | 1.01% | 0.08% | Fraction F up | Mmp8 |
| GO:0016798 | hydrolase activity, acting on glycosyl bonds | 8.29e-02 | 1 | 12 | 106 | 14763 | 8.33% | 0.72% | 0.94% | 0.08% | Fraction F up | Lyz2 |
| GO:0003714 | transcription corepressor activity | 8.36e-02 | 1 | 12 | 107 | 14763 | 8.33% | 0.72% | 0.93% | 0.08% | Fraction F up | Bhlhe41 |
| GO:0008528 | G-protein coupled peptide receptor activity | 8.44e-02 | 1 | 12 | 108 | 14763 | 8.33% | 0.73% | 0.93% | 0.08% | Fraction F up | Cxcr2 |
| GO:0016787 | hydrolase activity | 8.51e-02 | 4 | 12 | 2156 | 14763 | 33.33% | 14.60% | 0.19% | 0.08% | Fraction F up | Cpd, Ltf, Lyz2, Mmp8 |
| GO:0001653 | peptide receptor activity | 8.66e-02 | 1 | 12 | 111 | 14763 | 8.33% | 0.75% | 0.90% | 0.08% | Fraction F up | Cxcr2 |
| GO:0008201 | heparin binding | 8.74e-02 | 1 | 12 | 112 | 14763 | 8.33% | 0.76% | 0.89% | 0.08% | Fraction F up | Ltf |
| GO:0005539 | glycosaminoglycan binding | 1.11e-01 | 1 | 12 | 144 | 14763 | 8.33% | 0.98% | 0.69% | 0.08% | Fraction F up | Ltf |
| GO:0005488 | binding | 1.12e-01 | 11 | 12 | 10654 | 14763 | 91.67% | 72.17% | 0.10% | 0.08% | Fraction F up | Actn1, Bhlhe41, Cpd, Cxcr2, Igj, Lcn2, Ltf, Mmp8, Pstpip2, S100a8, S100a9 |
| GO:0004252 | serine-type endopeptidase activity | 1.15e-01 | 1 | 12 | 149 | 14763 | 8.33% | 1.01% | 0.67% | 0.08% | Fraction F up | Mmp8 |
| GO:0042802 | identical protein binding | 1.23e-01 | 2 | 12 | 754 | 14763 | 16.67% | 5.11% | 0.27% | 0.08% | Fraction F up | Actn1, Lcn2 |
| GO:0001871 | pattern binding | 1.23e-01 | 1 | 12 | 161 | 14763 | 8.33% | 1.09% | 0.62% | 0.08% | Fraction F up | Ltf |
| GO:0030247 | polysaccharide binding | 1.23e-01 | 1 | 12 | 161 | 14763 | 8.33% | 1.09% | 0.62% | 0.08% | Fraction F up | Ltf |
| GO:0046983 | protein dimerization activity | 1.25e-01 | 2 | 12 | 763 | 14763 | 16.67% | 5.17% | 0.26% | 0.08% | Fraction F up | Actn1, Lcn2 |
| GO:0003712 | transcription cofactor activity | 2.10e-01 | 1 | 12 | 287 | 14763 | 8.33% | 1.94% | 0.35% | 0.08% | Fraction F up | Bhlhe41 |
| GO:0000989 | transcription factor binding transcription factor activity | 2.16e-01 | 1 | 12 | 297 | 14763 | 8.33% | 2.01% | 0.34% | 0.08% | Fraction F up | Bhlhe41 |
| GO:0000988 | protein binding transcription factor activity | 2.22e-01 | 1 | 12 | 305 | 14763 | 8.33% | 2.07% | 0.33% | 0.08% | Fraction F up | Bhlhe41 |
| GO:0004175 | endopeptidase activity | 2.55e-01 | 1 | 12 | 358 | 14763 | 8.33% | 2.42% | 0.28% | 0.08% | Fraction F up | Mmp8 |
| GO:0032403 | protein complex binding | 2.65e-01 | 1 | 12 | 374 | 14763 | 8.33% | 2.53% | 0.27% | 0.08% | Fraction F up | Actn1 |
| GO:0030246 | carbohydrate binding | 2.78e-01 | 1 | 12 | 395 | 14763 | 8.33% | 2.68% | 0.25% | 0.08% | Fraction F up | Ltf |
| GO:0008270 | zinc ion binding | 2.89e-01 | 2 | 12 | 1310 | 14763 | 16.67% | 8.87% | 0.15% | 0.08% | Fraction F up | Cpd, Mmp8 |
| GO:0019904 | protein domain specific binding | 3.78e-01 | 1 | 12 | 573 | 14763 | 8.33% | 3.88% | 0.17% | 0.08% | Fraction F up | Actn1 |
| GO:0019899 | enzyme binding | 5.57e-01 | 1 | 12 | 968 | 14763 | 8.33% | 6.56% | 0.10% | 0.08% | Fraction F up | Lcn2 |
| GO:0005215 | transporter activity | 5.84e-01 | 1 | 12 | 1041 | 14763 | 8.33% | 7.05% | 0.10% | 0.08% | Fraction F up | Lcn2 |
| GO:0005102 | receptor binding | 5.92e-01 | 1 | 12 | 1061 | 14763 | 8.33% | 7.19% | 0.09% | 0.08% | Fraction F up | Actn1 |
| GO:0003824 | catalytic activity | 6.31e-01 | 4 | 12 | 5048 | 14763 | 33.33% | 34.19% | 0.08% | 0.08% | Fraction F up | Cpd, Ltf, Lyz2, Mmp8 |
| GO:0005515 | protein binding | 7.26e-01 | 4 | 12 | 5586 | 14763 | 33.33% | 37.84% | 0.07% | 0.08% | Fraction F up | Actn1, Cxcr2, Lcn2, Pstpip2 |
| GO:0004930 | G-protein coupled receptor activity | 7.48e-01 | 1 | 12 | 1603 | 14763 | 8.33% | 10.86% | 0.06% | 0.08% | Fraction F up | Cxcr2 |
| GO:0003677 | DNA binding | 7.78e-01 | 1 | 12 | 1738 | 14763 | 8.33% | 11.77% | 0.06% | 0.08% | Fraction F up | Bhlhe41 |
| GO:0004888 | transmembrane signaling receptor activity | 8.30e-01 | 1 | 12 | 2024 | 14763 | 8.33% | 13.71% | 0.05% | 0.08% | Fraction F up | Cxcr2 |
| GO:0038023 | signaling receptor activity | 8.42e-01 | 1 | 12 | 2104 | 14763 | 8.33% | 14.25% | 0.05% | 0.08% | Fraction F up | Cxcr2 |
| GO:0060089 | molecular transducer activity | 8.74e-01 | 1 | 12 | 2336 | 14763 | 8.33% | 15.82% | 0.04% | 0.08% | Fraction F up | Cxcr2 |
| GO:0004871 | signal transducer activity | 8.74e-01 | 1 | 12 | 2336 | 14763 | 8.33% | 15.82% | 0.04% | 0.08% | Fraction F up | Cxcr2 |
| GO:0003676 | nucleic acid binding | 8.96e-01 | 1 | 12 | 2540 | 14763 | 8.33% | 17.21% | 0.04% | 0.08% | Fraction F up | Bhlhe41 |
| GO:0004872 | receptor activity | 9.06e-01 | 1 | 12 | 2640 | 14763 | 8.33% | 17.88% | 0.04% | 0.08% | Fraction F up | Cxcr2 |
| GO:0003674 | molecular\_function | 1.00e+00 | 12 | 12 | 14763 | 14763 | 100.00% | 100.00% | 0.08% | 0.08% | Fraction F up | Actn1, Bhlhe41, Cpd, Cxcr2, Igj, Lcn2, Ltf, Lyz2, Mmp8, Pstpip2, S100a8, S100a9 |

### Unannotated IDs

|  |
| --- |
| **Fraction F up** |

|  |  |  |
| --- | --- | --- |
| [close] | **Legend: Edge Types** | (details) |
|  | | |
