# Supplementary material for: Ontology based molecular signatures for immune cell types via gene expression analysis
Source: BMC Bioinformatics. 2013 Aug 30;14:263. doi: 10.1186/1471-2105-14-263 (PMC3844401; doi:10.1186/1471-2105-14-263)
Supplement: Additional file 1 — OBAMS profiles for all mature B cells. Additional file 1 contains a zip archive of OBAMS profiles for all mature B cells, including for each cell type individual spreadsheets showing up and down regulated genes for that cell type relative to parental cell types, and VLAD (GO term enrichment) results for all mature B cells. [file 1471-2105-14-263-S1.zip › Additional File 1/GC B cell/VLAD.CL_0000844_down/results.html]

# CL\_0000844\_down

|  |  |
| --- | --- |
| Vlad version: | v1.5 |
| Date: | Mon Aug 29 17:26:28 2011 |
| Run time: | 45.61 sec |
| Ontology file: | gene\_ontology.obo |
| Ontology date: | Fri Aug 26 19:30:00 2011 |
| Annotation file: | gene\_association.mgi |
| Annotation date: | ??? |
| Analysis type: | enrichment |
| Excluded evidence codes: | ND |
| Number of query sets: | 1 |
| Query set 1: | CL\_0000844-down.xls (n=524; 2547 not found) |
| Universe set: | default (everything) |
| Graph display: | Top 25 scoring terms and their ancestors. Interior nodes have been culled. |

**Jump to:** biological\_process | cellular\_component | molecular\_function | Unannotated id/symbols

### biological\_process (top)

  
  


### cellular\_component (top)

  
  


### molecular\_function (top)

  
  


### Unannotated IDs

|  |
| --- |
| **CL\_0000844-down.xls**  0.0671532966705087 0.0685772667495683 0.0707744733114004 0.0785249224177332 0.0826460283042496 0.0849450146626529 0.0889410945486417 0.0933199618284615 0.0967662735079317 0.0979134640913211 0.0998191545142826 0.107016794465705 0.109044189191632 0.109646628062481 0.113641403799362 0.11809371866685 0.119104273464143 0.122756036898616 0.125185316100045 0.127303321949255 0.130527499184136 0.130655641598669 0.132230014328031 0.132317549788937 0.134024709012779 0.136514022780094 0.137158754156641 0.137881909001339 0.146355162913803 0.147633717625795 0.14822636761141 0.149229914691953 0.149717051420515 0.15037174615316 0.150717597682439 0.152046604313096 0.155733667105374 0.157769154987212 0.158263265903111 0.162761692358895 0.163804388565908 0.165770655898909 0.165803792373498 0.166621529450621 0.166761733681636 0.168636299404075 0.17080224458692 0.171067602728746 0.171918317233105 0.173782579797959 0.173840999257537 0.174058622836098 0.174298152442448 0.175788607913013 0.177581249090333 0.17836996791155 0.180648528007143 0.181860160376723 0.182794631735624 0.183227949666238 0.183326753707799 0.183940246334994 0.184369432455143 0.184375120557 0.184553151695584 0.184580068756482 0.185151714511813 0.188921011700784 0.18912089566926 0.189602722452036 0.19244940912394 0.194342729607205 0.194883103839808 0.196786835001649 0.197814243235554 0.198291521735896 0.199321580853227 0.199800341941087 0.199907307161211 0.200949052485394 0.201114859260332 0.201391235711443 0.201713555742686 0.203994248469866 0.206042991967349 0.2069372428678 0.20852467315569 0.20855237294356 0.209350757724652 0.209436994019822 0.209824789185217 0.210949947647183 0.212898189978615 0.213221837508192 0.215061649485336 0.215915132123786 0.216233151080776 0.220870700743322 0.223436189093783 0.223901277782458 0.225217059697002 0.226584808113747 0.230364470298959 0.230516111913665 0.231545688601804 0.232453001142052 0.232799126278375 0.235768745380297 0.236561821851447 0.237518958994827 0.239468175161295 0.239647466855831 0.241034328305609 0.241466400519819 0.243141568780499 0.243904041901776 0.244439731244076 0.250074171197982 0.250697730252051 0.251007807105773 0.252159139579732 0.252861364959525 0.253730101965963 0.255190334181369 0.255473961727714 0.255849662772352 0.259937855016628 0.260245056401517 0.26033034235465 0.261155151603296 0.262560642896827 0.264263948007568 0.267366886501845 0.268050389286304 0.268372801892343 0.269947618732539 0.270764053266244 0.272576572234278 0.275305942791972 0.275871623145802 0.275913910172378 0.277030765923652 0.279869206604965 0.281356048672293 0.282573402643287 0.284307568407313 0.286260891032908 0.2886382364838 0.290080575238203 0.291177861569837 0.29224000605413 0.293154662467223 0.293263839578944 0.294468728069782 0.297192706589096 0.298667035528105 0.300200301548435 0.300979979088011 0.301447598085401 0.302539591448765 0.302959157580658 0.305032920379736 0.308268554102539 0.308605149217445 0.308939793536015 0.309138519826984 0.310751530365247 0.310991346970266 0.311342606884855 0.311747774645752 0.312343920554899 0.312490842133326 0.31574522595999 0.316304124236444 0.316513791680177 0.317113211244513 0.318376632078521 0.319470289160169 0.320384119520961 0.321143173720462 0.321642951600219 0.323539754360351 0.325031423707824 0.327646670047336 0.330438579485868 0.331093877568361 0.331196192148541 0.331318349733665 0.331336669740265 0.334427697994012 0.335290595567651 0.336673659395228 0.336815637417742 0.337721021378349 0.338127732781107 0.343064597419428 0.343569928018202 0.34383368414372 0.344323091115464 0.346119062179427 0.347907370670367 0.348861580493409 0.348908713577781 0.35037919895649 0.353206207135949 0.359761460180217 0.360339978424569 0.363194463649289 0.363274057018663 0.363866136183989 0.367477162900948 0.371133529455303 0.371513815107752 0.371663091207718 0.37310010101392 0.374731626554329 0.376896553541973 0.377367162845918 0.382947804992944 0.386285564580276 0.389735117879921 0.390538740186218 0.390720107899736 0.391048647455127 0.39150304418581 0.397112450943743 0.398151295860127 0.400699855196331 0.401231063865666 0.40360107856801 0.403833480890396 0.404983834213337 0.405340480320683 0.405986420481002 0.410655843032665 0.414424525763871 0.416889102700959 0.418726457690648 0.422566512630552 0.422840017540672 0.423255849906171 0.429120120701888 0.431487414632257 0.433971334036314 0.435133721998808 0.438641405367187 0.440675605761163 0.44175797924803 0.442046201183638 0.443928369034745 0.444105233854911 0.45031200095152 0.451114976092299 0.45426692594536 0.456170391581556 0.459384622567856 0.459734368188913 0.462157861593798 0.462635021801589 0.463328566748774 0.463616094010906 0.464441598936655 0.467465951811291 0.467858494128027 0.470417380288058 0.470573229006738 0.47196143598284 0.472435880869784 0.472823734865041 0.473602947721175 0.47426254317792 0.474628181151782 0.476748810995635 0.479413397314639 0.482099278174605 0.483359721978916 0.488638171827122 0.48982847202865 0.490942867101397 0.491248342355828 0.491751434525077 0.491792495806977 0.491987032792896 0.493732623453045 0.497464640431993 0.498582386300891 0.500127554107279 0.503956325150318 0.5059827528053 0.509170848222608 0.510841588708497 0.511342652649456 0.512647934343318 0.512848823590779 0.514439828944629 0.514996408221487 0.516054480550939 0.517966423769664 0.518211123386575 0.518571711488687 0.521132864386951 0.522320979430571 0.523504048516861 0.525403376865554 0.526795672676676 0.528597891473364 0.528641445355619 0.528966073769456 0.535711082893796 0.535886586078899 0.537432387251089 0.539029353158666 0.542391903978262 0.545101807299354 0.547821576244264 0.549380298658438 0.550397841959216 0.557726626713936 0.55809159592837 0.558480397952901 0.570639255232607 0.573467724854327 0.577516385449116 0.581283546073648 0.58167168872231 0.585304433346401 0.586661093789968 0.58833972374303 0.592397966344745 0.595734196320528 0.599222471129696 0.606047839602612 0.608580370558851 0.611467143105606 0.614771983148052 0.619459307047479 0.626147081667587 0.626429739009054 0.630188478644341 0.632479533224091 0.635648268602429 0.642538577588958 0.644224185512777 0.646708634686112 0.649839532968393 0.65378205992556 0.655464408779857 0.656437707196679 0.656510344816577 0.671971081086871 0.678845297950204 0.68019593205797 0.680921582789759 0.682561280563451 0.683645023590796 0.685976345256596 0.693720213864336 0.695001544026769 0.695096891248032 0.696773572087139 0.701324188433358 0.707940566881621 0.709932490145194 0.712257631623891 0.715203490633737 0.721655670083493 0.723821034478678 0.728249821421849 0.732081390827794 0.733679520353798 0.746096055619533 0.746470628360059 0.747041961986169 0.748361902760589 0.749155905030097 0.751689092522968 0.755169691601381 0.763952841498057 0.764333705281123 0.764503609717497 0.768610625951444 0.769507750571552 0.775519118900297 0.776312237601836 0.777507436873831 0.778309641958801 0.780398932223308 0.787336786427356 0.795569983757804 0.79824208828069 0.801947454490211 0.806328974446362 0.80949985785476 0.81052266311587 0.810766232354005 0.813163788025167 0.821060940902085 0.828417814012562 0.832013478701514 0.8420974484012 0.846437495992055 0.850795547904924 0.860321002873577 0.863989051610999 0.864400658032225 0.864516263817498 0.868136591292 0.872064923846689 0.87400841139079 0.87556376477653 0.878157582970418 0.888596360559425 0.890062381701065 0.892913850680893 0.893472016317919 0.895532082669814 0.898807591711365 0.900102850522414 0.901009814486552 0.925920501575169 0.925972656913253 0.926505602465869 0.927168984595585 0.928466760199549 0.939620179810506 0.939951882881252 0.94140875075929 0.943183902121071 0.947249808178559 0.948482600616935 0.948683966709605 0.951160376002686 0.951251678568441 0.954404673909542 0.954992886041668 0.959893170967632 0.962629485247472 0.967580692333905 0.968422344712074 0.970866618687737 0.9710657156126 0.972570347098183 0.981408609892533 0.981465685474733 0.982579375799811 1.00498945717688 1.00510263358197 1.00593426702262 1.01192510013773 1.01933496265171 1.03420574296024 1.04022933160882 1.05061249516973 1.06486893632935 1.06672538728829 1.06776852140266 1.07183149007689 1.07728102115325 1.08383463444967 1.08819680380305 1.08883747941984 1.09857725775678 1.10345889159308 1.10889901693909 1.11374532429774 1.11442309976332 1.11588489464448 1.12217734669621 1.12242187124118 1.1282693631141 1.1385834428133 1.14176762150839 1.16549467547286 1.16742206093202 1.16866185392641 1.17190850063103 1.17417989722605 1.17483928719024 1.18318039162165 1.18820153107582 1.19342902135472 1.19986962506733 1.2263053315171 1.24107143910894 1.24110058120059 1.25254472824171 1.25750698457588 1.2702193462861 1.27146439714718 1.27450788755882 1.28968703879712 1.29695271065885 1.31019661262211 1.31425690234396 1.32462184566413 1.33129060951741 1.34514800995995 1.34998461698496 1.35109842497356 1.36521635150706 1.3661447774798 1.36717243763055 1.37168045997626 1.3778233043925 1.38318708713764 1.39616597565819 1.42739546228396 1.4420225847108 1.45443339126579 1.46253138158871 1.4763917002232 1.4786558922372 1.50512744773657 1.5299540862139 1.56100733925789 1.58555423382983 1.58641918942643 1.58650989530738 1.59714663806455 1.59755274235162 1.60092263898041 1.60895285674797 1.6107530270413 1.61136654697174 1.61160238468045 1.61326565571452 1.61425969052106 1.6181154924977 1.61905623349255 1.62390312243643 1.63346914684061 1.63704810060583 1.63917727246287 1.6512636672491 1.67229105229773 1.67762979926918 1.6802558940551 1.68961912556833 1.69570785188422 1.70305539232582 1.71161829677526 1.71338829825909 1.72214274202841 1.72712958571233 1.72729921301488 1.73152454107104 1.73313876299823 1.73737858923171 1.73978693716916 1.7402915581147 1.74107243014822 1.74184249092801 1.74526086397595 1.75255162766556 1.75357305859146 1.7552368236966 1.75592967876308 1.75869917373987 1.7594544878964 1.76267311634822 1.77146688670217 1.77491034550855 1.77698337743358 1.77725879597406 1.7783120552497 1.78060846003951 1.78743146077807 1.79975292535736 1.80115898383345 1.80639324463142 1.80653102678454 1.80886372581884 1.80991032289268 1.81189290206542 1.81250213172695 1.81461862550262 1.81809732818146 1.81957474193352 1.82145678855184 1.82229231170733 1.82253750375345 1.82359198501097 1.82943248094836 1.83029310396725 1.8303223939121 1.8306607496875 1.83229098867142 1.83276556574374 1.83313315400589 1.83741544821342 1.83774007465244 1.84618705209437 1.84692168351072 1.84837054108343 1.84890257693248 1.85114229119642 1.85228442242855 1.85639306343598 1.85812813615526 1.85947157978114 1.86035025127052 1.86556857666539 1.86748737150582 1.86990100575447 1.87015059474142 1.87304781552375 1.87388834333995 1.87597434853295 1.87812583091056 1.87848103491073 1.88523295244167 1.88595951721604 1.88729738141257 1.89000342637498 1.89027173647309 1.89186586658044 1.89604499809786 1.89808617402285 1.8982050543999 1.90107832386416 1.90654585703768 1.90983846391427 1.91035494247677 1.91521438254215 1.91840025198501 1.92644025128372 1.92964110555826 1.92994571953371 1.93000366415412 1.93246279537169 1.93413500770996 1.93727875793709 1.93872927588404 1.93899801667539 1.94193121122243 1.94364329656276 1.94664767485171 1.94681646416191 1.94801380780162 1.96008161624509 1.96148836214277 1.96532063950507 1.96629193883659 1.96758351039807 1.97265915853805 1.97376911934074 1.97669731090333 1.97976077815819 1.98353810800923 1.98602954951804 1.98605722471489 1.99026718753247 1.99046791818174 1.99150975941634 1.99195990706637 1.99974658278358 10.0558243332349 10.0589882725615 10.1098079464633 10.1169115968026 10.1664342473698 10.1787931966424 10.3867555119551 10.3889382774491 10.5216891896196 10.6855807917226 10.7696599715821 10.7949638318598 10.9708124003499 100017 100038548 100038711 100043197 100043757 100049155 100121 100198 100529082 100637 101202 101476 102098 10338068 10338112 10338160 10338209 10338292 10338326 10338361 10338388 10338393 10338472 10338475 10338504 10338557 10338585 10338673 10338827 10338979 10339056 10339133 10339447 10339491 10339604 10339649 10339773 10339787 10339822 10339917 10339936 10340025 10340177 10340202 10340438 10341115 10341128 10341187 10341455 10341513 10341637 10341646 10341722 10341723 10342025 10342222 10342261 10342311 10342418 10342595 10342733 10343202 10343312 10343407 10343412 10343543 10343739 10343842 10343866 10343994 10344017 10344057 10344141 10344166 10344218 10344243 10344429 10345445 10345967 10345981 10346168 10347564 10348889 10349102 10349782 10349793 10350046 10350742 10350977 10351197 10351679 10351689 10351880 10352092 10352914 10352916 10352918 10354003 10354111 10355141 10355162 10356020 10356932 10357001 10357043 10357371 10357480 10357488 10357535 10358928 10358978 10359303 10360367 10360370 10360373 10360391 10360631 10360684 10361133 10361169 10361215 10361338 10362294 10362811 10362861 10363430 10364030 10365344 10365891 10366667 10366848 10366886 10366956 10367600 10367843 10368380 10368886 10368888 10369154 10369171 10369877 10369911 10370072 10371356 10371387 10372781 10372988 10373027 10373407 10373542 10373740 10373902 10374151 10374406 10374560 10375083 10375402 10375503 10376060 10376208 10376912 10377265 10378068 10379615 10379630 10379901 10380477 10381082 10383799 10384577 10384985 10385500 10385507 10385511 10385526 10385966 10385993 10387372 10388194 10388902 10388958 10389087 10389143 10390103 10390574 10390763 10391378 10392221 10393113 10393341 10393573 10394593 10394912 10395520 10395978 10396440 10396606 10396608 10397645 10397708 10397715 10397763 10397853 10398039 10398459 10399478 10399696 10399897 10401473 10401684 10401698 10402063 10402066 10402444 10402730 10402800 10402986 10403584 10404132 10404152 10404389 10404848 10404874 10406852 10406905 10407173 10407570 10407707 10407766 10408280 10408557 10408935 10409414 10410309 10410311 10410321 10410328 10410644 10410721 10411126 10411609 10411611 10412038 10412078 10412100 10413059 10413100 10413185 10413216 10413220 10413419 10413943 10414548 10414876 10415415 10415576 10415640 10415725 10416199 10416371 10417053 104175 10417734 10418171 10418991 10419288 10419674 10420348 10420372 10421172 10421526 10421555 10421557 10421774 10422013 10422059 10422493 10422496 10424731 10425053 10425092 10425333 10425410 10425430 10426835 10427807 10428796 10428912 10429114 10429128 10429160 10429520 10429926 10430931 10432298 10432675 10432957 10433057 10433578 10434291 10434932 10436182 10436196 104367 10436734 10436830 10436841 10436849 10436865 10436983 10437023 10437695 10438478 10439058 10439239 10439583 10440246 10440926 10442495 10442739 10443063 10443506 10443730 10444780 10444821 10444824 10444830 10444995 10445531 10445803 10445875 10446334 10446986 10447294 10447315 10447356 10448064 10448765 10449266 10449955 10450069 10450075 10450374 10450675 10450699 10450733 10451039 10451736 10452269 10452639 10453178 10453423 10453636 10453736 10454512 10455259 10455769 10456296 10457040 10457225 10457508 10457640 10458028 10459288 10459518 10459723 10459768 10460968 10461487 10461614 10461642 10462237 10462390 10462818 10462957 10463070 10463112 10463486 10463911 10463997 10464647 10465244 10466314 10467068 10467230 10467420 10468059 10468292 10468294 10468853 10469514 10470050 10470320 10470751 10471080 10471912 10471929 10473125 10474006 10474545 10475051 10475912 10476106 10477250 104776 10477986 10479159 10479463 10479726 10481092 10482448 10482802 10483046 10483178 10484371 10486119 10487433 10487945 10488291 10488322 10489484 10490826 10491091 10492180 10492540 10492590 10492628 10492815 10492824 10492890 10492983 10493990 10493995 10494335 10494428 10494574 10494595 10494978 10495659 10496539 10496569 10496580 10497149 10497421 10497548 10497646 10497817 10498992 10499160 10499529 10499612 10501586 10501699 10502284 10502299 10502335 10502469 10502890 10503401 10503523 10504672 10504849 10505451 10506058 10506134 10506488 10508788 10509030 10511290 10512067 10512766 10512847 10512949 10515220 10515429 10516103 10517301 10518743 10519105 10520096 10520388 10520950 10521205 10521678 10522051 10522335 10523547 10523647 10523670 10524310 10524345 10524621 10525439 10525487 10525489 10525495 10527012 10527101 10528723 10529497 10530100 10531724 10531919 10532133 10532744 10532767 10533198 10533213 10533729 10534202 10534324 10535841 10535866 10535938 10537441 10537911 10538115 10538126 10538135 10538138 10538142 10538885 10539135 10540408 10540795 10541532 10541785 10541877 10542060 10542120 10542302 10542310 10542317 10542880 10542911 10542917 10543785 10544148 10544150 10544525 10544538 10544583 10544588 10544891 10545780 10546272 10546661 10546762 10548817 10549361 10549854 10550509 10550906 10551487 10551791 10551883 10554118 10554521 10554945 10555039 10555235 10555425 10555470 10555862 105559 10556082 10556302 10557009 10557399 10558134 10558227 10558910 10559248 10559446 10559454 10559467 10559478 10560911 10561008 10562192 10563099 10563114 10564294 10564573 10565811 10565852 10565924 10566326 10566350 10566366 10566574 10566578 10566580 10566583 10566585 10566709 10566846 10568328 10568863 10569513 10569719 10569733 10569767 10570516 10571325 10571984 10572800 10573747 10575160 10576692 10576696 10577560 10578989 10580033 10580061 10581036 10581996 10582845 10583203 10583207 10583586 10583669 10585301 10585318 10585555 10585825 10585905 10586039 10587012 10587534 10588505 10588876 10589329 10589350 10590801 10591224 10591263 10592266 10592471 10593050 10593492 10593497 10593799 10593856 10593878 10594053 10594404 10594613 10595000 10595402 10595404 10596545 10597978 10598178 10598839 10600308 10600357 10601416 10601421 10601424 10601834 10603026 10604906 10605303 10606009 10606016 10606184 10607870 10608649 10608698 10608706 106581 107526 107607 107723 108089 108097 108105 108655 108937 108960 108995 109689 11.2145386925211 11.2190382232431 11.2686657009709 11.3976627388638 11.4089197261512 11.5300312731841 11.6256998688976 11.8013279819894 11.8583220073948 110168 110557 110956 11303 11305 11307 11426 114615 114664 11492 11513 11540 11555 11717 11747 11749 11796 12.3439781758003 12.4307649596448 12.6939548955829 12.8063644248154 12.9578274346344 12013 12040 12043 12151 12228 12234 12290 12304 12332 12399 12479 12494 12495 12523 12576 12695 12763 12775 12807 13.1518462334974 13.1705900571725 13.2299191105856 13.5002984611969 13.8977188204665 13136 13137 13139 13350 13482 13609 13631 13650 13849 13982 14.0566466789393 14.2502690326472 14017 14020 140482 14055 14073 140742 14245 14421 14470 14528 14590 14667 14744 14964 14972 15.1116534782934 15.1709554128787 15.5058910494608 15018 15019 15042 15162 15242 15468 15925 15931 15939 15944 15950 15975 15976 15980 16.2033265730609 16.6959566027548 16150 16154 16155 16159 16186 16192 16195 16205 16337 16362 16364 16401 16421 16423 16432 16438 16468 16597 16598 16599 16764 16848 16859 16885 17.0779933212265 17068 170716 170743 17127 17155 17281 17355 17425 17444 17951 17969 17972 18.0437555963141 18.2069553224065 18033 18073 18111 18129 18145 18301 18406 18438 18569 18584 18604 18613 18640 18689 18724 18726 18733 18754 18763 18793 18796 18854 19.1134980569535 19.2643120997171 19.4925842861855 19024 19039 19156 19188 19200 192119 192197 192285 19260 19336 193385 19347 19395 19401 19645 19684 19729 19889 2.00303931193454 2.0035913806738 2.00442948233519 2.00592891225899 2.00638174740723 2.01187033708225 2.01343558905643 2.01468142662012 2.02241202997612 2.0247781872432 2.02696252429633 2.03505638628627 2.03518935366789 2.03743089410687 2.038502454539 2.0413836004619 2.04236796083983 2.05279062076231 2.05348811865008 2.05689758484005 2.05719551165226 2.05784699625082 2.05916443870715 2.06847832014444 2.07088308495767 2.07145636925534 2.07230979154752 2.07300471169216 2.07638377392723 2.0803300535955 2.08359907900698 2.08361731397329 2.08373653089465 2.09117687288046 2.09913868355295 2.1018718536932 2.1041918213654 2.10487870385166 2.10529848944272 2.10644612713693 2.10852114697538 2.10999830379582 2.11332357688931 2.11437513340217 2.11538586561345 2.11601565424572 2.1173024899546 2.11989074722802 2.11991961407292 2.12327281786915 2.12895122663388 2.12963122671403 2.12970347761212 2.130216186537 2.13199896657683 2.13202137739196 2.13435350839803 2.13612648962698 2.13672980765436 2.1420567440313 2.14209988621626 2.14390157464068 2.14478056130594 2.15212220309177 2.15214867357669 2.15286867806856 2.16395655331166 2.16522461965197 2.16555497458769 2.17022857814364 2.18229371057686 2.18245820959666 2.18355478966322 2.18441358197255 2.18578589218856 2.18899549446033 2.19082821777518 2.1911990229267 2.1930237778386 2.19865199300572 2.20081459430261 2.20733453693047 2.21056417808831 2.21921021748409 2.21966544030249 2.22071442933173 2.22085222678053 2.22099133898494 2.22711958008342 2.22719721914736 2.23160597516474 2.23239619975308 2.23327739672992 2.23353246322264 2.23742841196825 2.24441291174131 2.24514054462054 2.24540202176177 2.24635233248796 2.2471682045053 2.24734899706933 2.25445149732293 2.25580093010617 2.25902900660945 2.26050596268274 2.26512492518037 2.27018448528279 2.27140998853029 2.27265358570409 2.27449580021108 2.27731956892446 2.27788193139793 2.28297566670073 2.28853123179877 2.29060095956065 2.29408807113672 2.29761872235809 2.30472500374029 2.30900387164201 2.31598531534072 2.31945365410054 2.32125528687079 2.32333663680707 2.32450033337162 2.33187532005609 2.3339538510706 2.33396806397812 2.33549813583376 2.33581059915603 2.34123507455521 2.34699121862137 2.34918449147402 2.35166787999683 2.35303549632273 2.35548469916626 2.36023588539153 2.36942309605364 2.37539563205276 2.37780999478601 2.37886661179305 2.38176107482321 2.38246916980135 2.38600157263498 2.39285921999265 2.39408591800886 2.39625862015594 2.39646369899439 2.40383325849317 2.40468013862217 2.40680481949368 2.40957894181826 2.41489066481645 2.41828547247988 2.41892968164081 2.42250485825324 2.42575421263614 2.43189190569337 2.43686975689611 2.44194444784117 2.4423052085495 2.44296172409023 2.44307930147136 2.4482332809126 2.44911157116738 2.45851566712871 2.4614211436953 2.46325753008919 2.47464751392197 2.47850665888476 2.4795417681219 2.48288775769339 2.4912632013962 2.49593733700401 2.50620136919042 2.50643055433724 2.51495081058882 2.51767236244597 2.51825794817023 2.52167080577905 2.52400362510396 2.52765313089525 2.5328098859175 2.53435398451611 2.54214667119643 2.54634259608809 2.55792328978229 2.55984800887559 2.56363183551861 2.56374520119468 2.56501929217064 2.56919579296949 2.57402316327994 2.57921961524056 2.57965449982334 2.58024989730171 2.59319876580426 2.59458202968269 2.59519933076454 2.59554879844177 2.59667894235534 2.59747756204653 2.60301205663955 2.60999281696449 2.61697427091672 2.61848573880665 2.62139279213201 2.62464176950812 2.63691283968943 2.64160319371862 2.64283980030026 2.64361149332461 2.64453616155786 2.65359012056469 2.65568838929643 2.65930811233298 2.66217663510176 2.66966876902537 2.67180172986163 2.67183600044766 2.67249540984999 2.67633421956033 2.67796091467781 2.68255415596228 2.68491629530173 2.69249713190267 2.6963273895501 2.70835555187121 2.71112668078635 2.71260748107192 2.71323898271357 2.7133802375105 2.72299391745663 2.72716918508459 2.73234718712291 2.74205005441706 2.74423153763537 2.74428494028748 2.74667238961441 2.74914891293851 2.75357798175265 2.75376767404444 2.75401446015039 2.76812386573322 2.77173037789804 2.77825220452614 2.78450850518567 2.78894102129523 2.79023009843585 2.80031049767301 2.80059619036427 2.80440422777721 2.80657482639917 2.80908073265319 2.81628515335258 2.8181716651731 2.82077067296449 2.82127887517521 2.82506398024788 2.83233483967969 2.83257355697215 2.83401170885671 2.83996715384689 2.84906063245594 2.85156364931935 2.8530968363004 2.85533802721021 2.85577699795151 2.85956959075937 2.86505900335721 2.8654007418861 2.86572695775045 2.87886073692321 2.88729420945502 2.89102759941887 2.89350297787413 2.89905396449471 2.90335081453379 2.90723701719761 2.9167257460133 2.91948245601863 2.92560925718792 2.93029956292646 2.93197628701284 2.93450214899398 2.94276691637141 2.94286195995357 2.94489164361396 2.94705283567946 2.96616256460077 2.96811528747739 2.97386529333077 2.97796982663046 2.98083295476683 2.98606192979905 20024 20194 20195 20343 20345 20442 20447 20481 20491 20503 20556 20621 20713 208043 208449 20849 20852 20868 209086 209380 209387 209478 209683 20971 21.0447578149804 210293 212285 212943 213068 213389 213438 213439 213452 213980 213988 214133 21452 214855 215243 216080 216190 216197 216198 216445 216505 21652 216850 216892 217124 217344 217648 217830 217835 218203 21887 219094 219105 219140 21936 21946 21981 22022 22035 22177 22183 22288 22317 22330 224093 224109 224132 225028 225115 225651 225912 22592 226861 22690 22724 227929 228993 229055 229603 229898 229900 230073 230793 231051 231147 231287 231507 231510 231637 231931 231932 232227 232430 232685 233058 234311 234353 235283 235406 235441 237339 238330 238393 238692 238722 238875 239217 23943 23962 239667 24014 24017 240354 24055 240665 24088 240921 241075 24109 24136 241694 241915 242083 242248 242557 243374 244059 244183 244202 244871 244891 244895 245536 245607 245867 246229 246727 246728 246738 26364 26365 26382 26401 26410 26450 26569 268297 269181 26931 270118 270198 27027 27209 27212 272347 27360 27416 276950 277089 27973 29808 3.0001974277628 3.00041380465851 3.00430388604445 3.01517764529817 3.02027521749152 3.02117681776922 3.02937501678567 3.03543770658824 3.03804713978546 3.04016150648852 3.04888220293228 3.07243490143455 3.07326674424685 3.07964921475722 3.0796952300665 3.08253304469126 3.08540946498422 3.08945925694333 3.09481778313402 3.09662168146009 3.09670149583174 3.0999623495803 3.10043970233082 3.10167209739133 3.102196538249 3.1041852236839 3.10526209101482 3.10994922465303 3.11385289098917 3.12824962140093 3.13407622306818 3.13512458133467 3.14278288172054 3.14829864342754 3.15439059196468 3.1583683647392 3.15932400807032 3.1647082310024 3.16900657968897 3.1697101620035 3.18470797148923 3.19097080437735 3.19539930240962 3.20416763594469 3.20847136206944 3.21219515278625 3.21777450839631 3.21961717976103 3.23849325118679 3.2464512768336 3.2518995955739 3.2746112715538 3.27932224757206 3.28552416434208 3.29448074224681 3.30709262069137 3.32760725003102 3.34762500642739 3.35088391606198 3.35709008138166 3.35948769445493 3.36099727176314 3.36352885708781 3.36937213649062 3.37244789287024 3.37301541881009 3.37397895121395 3.38885104051824 3.4302591693632 3.43131761543729 3.43470688879821 3.43513695511808 3.44231180240001 3.44747885914361 3.45674659899738 3.462969786298 3.47223929082703 3.4833527827764 3.48920085723907 3.49128792529138 3.50387004837181 3.5117299258418 3.51256961053381 3.51528478961374 3.53435891089213 3.53908556829095 3.54560183909916 3.54568148324682 3.55471737000558 3.5658047513273 3.56915119449748 3.59463526318086 3.60427167878446 3.62479996833797 3.6342156016127 3.65197481405939 3.66231888748021 3.66476202609399 3.6665236866726 3.66900224302162 3.67440422590164 3.67916969798618 3.68362645424358 3.69730586998601 3.71359062899415 3.72199687848101 3.7225654922141 3.76881581168139 3.78299569880157 3.78770691002059 3.79286587985807 3.80123863125084 3.81188739133416 3.82131303457519 3.86351318026766 3.86910281507077 3.87022975594277 3.88860115184606 3.88915421036637 3.89634164174494 3.89715533809578 3.90660171184074 3.92947545278054 3.93370058789198 3.94060636430937 3.95968095294724 3.96490391522029 3.98394859896147 3.98866290256813 3.98962052935077 3.99821344664223 30.4248120170954 30838 30843 30928 30957 317757 319493 319565 319604 319876 319880 320007 320100 320204 320207 320305 320495 320782 320977 321019 327959 327978 328186 328365 329003 329693 330192 330554 338364 338467 338523 353187 380686 380921 381022 381626 382062 399603 4.02823859349794 4.04407430226817 4.05686112078064 4.06740031012575 4.06913837435198 4.07189365597935 4.08266750941125 4.10605996356431 4.11560127729855 4.13765795856281 4.14034807669135 4.1434092597348 4.1498935035009 4.15209905911671 4.16768762757419 4.16774446982824 4.16890059914814 4.17156301546131 4.18597781546065 4.20938622445061 4.20953400232401 4.22425545352578 4.23375427137458 4.23491402601848 4.25215070034331 4.25856259612983 4.2731356941527 4.27567131862063 4.28074547569519 4.29232420425719 4.30546744593188 4.33544073720218 4.34050478359549 4.34125209857193 4.34555804971317 4.35117306133077 4.36743044879463 4.36809947467452 4.36819618769438 4.43605991999443 4.45025260057631 4.47706616083161 4.48683676997593 4.49453285314733 4.51359271515225 4.54978408705692 4.56016077846725 4.57579690967409 4.57667683516182 4.60358576453883 4.60840559970485 4.61540411560824 4.62935059633816 4.65388370738922 4.70523325454107 4.71913410878529 4.72803941134523 4.75250044147928 4.79605445368593 4.80743365219758 4.82286527038482 4.85259013820299 4.86608594614452 4.86816455726363 4.88544789078643 4.88600409980293 4.88909107717522 4.89560367977455 4.9096637909972 4.93153864360992 4.95899527256934 4.96554932585203 4.97428577380064 4.97692335812526 4.98015785793741 40.772460691952 408065 408067 41.6572124233377 432555 434156 434197 434223 434377 449631 5 5.02053865899718 5.03224177659483 5.08208697752892 5.10859927029761 5.11320075527804 5.11398390871982 5.11993397382078 5.1279398015446 5.13334761732811 5.13745650188763 5.17217707871869 5.24140640140522 5.2807937870553 5.30289621330307 5.3078967154945 5.32116752733459 5.35264653597425 5.36321863415593 5.40484878179158 5.41915644327337 5.42961902538767 5.47406571549901 5.50205749151672 5.57940227370736 5.61733578151599 5.63470083743698 5.67562223442237 5.68775067383066 5.69078537313198 5.69665774394592 5.82879287617987 5.86068967625368 5.90873999250843 5.91652553355858 5.92265966127303 5.95343280471804 5.95958650527368 50766 50875 50931 52065 52323 52392 52915 54132 54137 54169 54446 545182 545216 545242 545384 54598 55932 55936 56048 56338 56376 56468 56484 56696 56737 56812 56857 57748 57785 57813 57869 57908 6.07272409205365 6.11488514605432 6.14832003754198 6.28464099944957 6.31011029926053 6.32037309944066 6.35258123341013 6.38514306976085 6.38629087366678 6.38904702316037 6.48611507319757 6.4907339083298 6.54866717259265 6.72028035887422 6.8115896322478 6.83519270309409 6.83652883524455 6.84253268168389 6.85879182884967 6.85964060121624 6.86230299772226 6.87786454823458 6.94766545924838 6.98988490123374 60533 60611 619313 620913 622320 623121 625046 630294 630499 66222 66257 66505 668108 668139 66826 66857 66885 66940 67125 67155 67168 67263 67287 67338 67426 67464 67488 67512 67580 67623 67647 67712 67727 67731 67916 67938 68146 68151 68631 68682 68732 68737 68778 68897 69106 69146 69537 69710 7.10309475622365 7.12381957353414 7.12687569788906 7.15372005039094 7.25617388148176 7.25689492650825 7.31335101906283 7.47413131200115 7.49421365126052 7.58187006790908 7.63189226621198 7.67163068276851 7.69129214735959 7.69419809097256 7.72367516139288 7.80984546785416 7.81584906185234 7.88062724753977 7.93579646017283 7.9525437183756 7.98953348701596 70028 70425 70785 70789 71302 71375 71683 71704 71706 71918 71962 71994 72007 72160 72180 72194 72238 72278 72289 72318 72461 72477 72519 72691 72972 73389 73656 73910 73914 74011 74039 74137 74154 74182 74270 74366 74558 74735 74901 75255 75605 75612 75796 75914 76007 76041 76117 76491 76589 76681 76820 77041 77559 77592 78416 78826 79196 8.05628585474139 8.09667244038402 8.30083963551225 8.31189609605191 8.34883822625186 8.38892839766164 8.39126636916672 8.42316493671454 8.48319646963885 8.56680043183293 8.59539383849549 8.60190537520538 8.66721289195512 8.76533816858582 8.86455775444428 8.87157142806245 8.91935137101002 80287 80877 81000 83408 9.24259090283822 9.32481705305625 9.46239079644889 9.51231687116728 93675 93691 93757 94089 94094 94281 98314 A330023F24Rik D1 D17H6S56E Evi5 FoldChange Gimap1 Gm10500 Gm10786 Gm4759 Gm5068 Gm5815 Gm6545 Gm7030 Gm8979 Gm8995 H2 Ier5 K1 K2 MGI:104736 MGI:109368 MGI:1306799 MGI:1337072 MGI:1343184 MGI:2153740 MGI:2443958 MGI:3641966 MGI:3642147 MGI:3643874 MGI:3644223 MGI:3645720 MGI:3647514 MGI:3647753 MGI:3648184 MGI:3703149 MGI:95906 NA Q6 Q7 Q8 Samd9l Stdv T24 Zfp358 entrezIDs mgiID symbol |

|  |  |  |
| --- | --- | --- |
| [close] | **Legend: Edge Types** | (details) |
|  | | |
