# Supplementary figures and images for: Ontology based molecular signatures for immune cell types via gene expression analysis
Source: BMC Bioinformatics. 2013 Aug 30;14:263. doi: 10.1186/1471-2105-14-263 (PMC3844401; doi:10.1186/1471-2105-14-263)

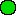

Supplement: Additional file 1 — OBAMS profiles for all mature B cells. Additional file 1 contains a zip archive of OBAMS profiles for all mature B cells, including for each cell type individual spreadsheets showing up and down regulated genes for that cell type relative to parental cell types, and VLAD (GO term enrichment) results for all mature B cells. [file 1471-2105-14-263-S1.zip › Additional File 1/B-1 B cell/VLAD.CL_0000819_down/auxfiles/ball.gif]

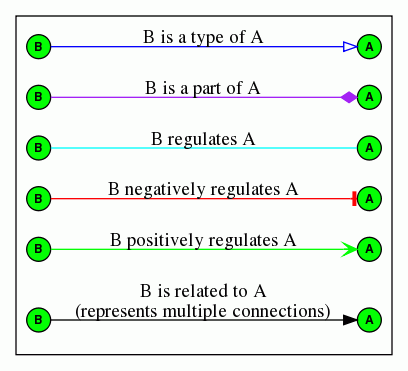

Supplement: Additional file 1 — OBAMS profiles for all mature B cells. Additional file 1 contains a zip archive of OBAMS profiles for all mature B cells, including for each cell type individual spreadsheets showing up and down regulated genes for that cell type relative to parental cell types, and VLAD (GO term enrichment) results for all mature B cells. [file 1471-2105-14-263-S1.zip › Additional File 1/B-1 B cell/VLAD.CL_0000819_down/auxfiles/legend.gif]

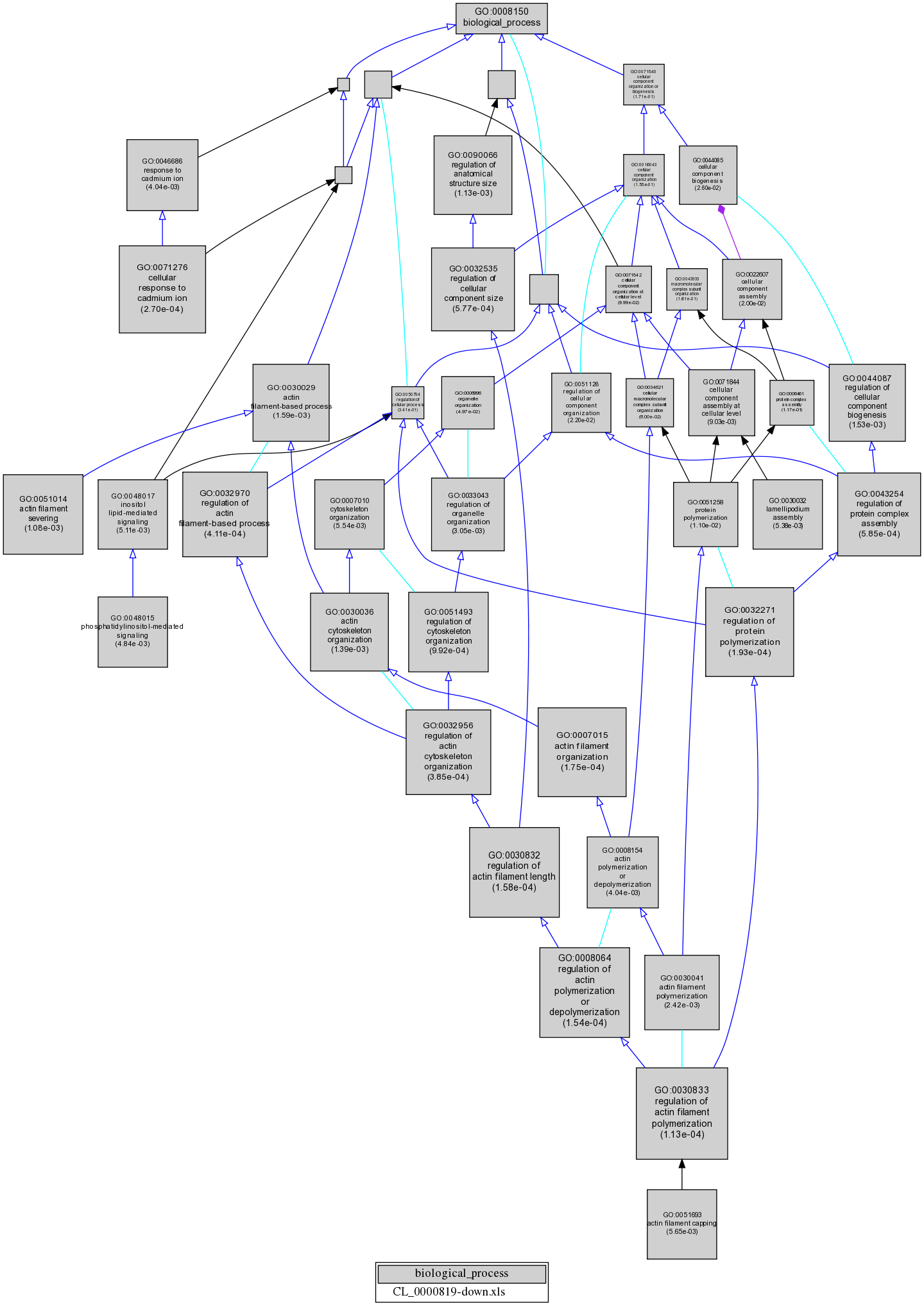

Supplement: Additional file 1 — OBAMS profiles for all mature B cells. Additional file 1 contains a zip archive of OBAMS profiles for all mature B cells, including for each cell type individual spreadsheets showing up and down regulated genes for that cell type relative to parental cell types, and VLAD (GO term enrichment) results for all mature B cells. [file 1471-2105-14-263-S1.zip › Additional File 1/B-1 B cell/VLAD.CL_0000819_down/auxfiles/vlad.results.biological_process.png]

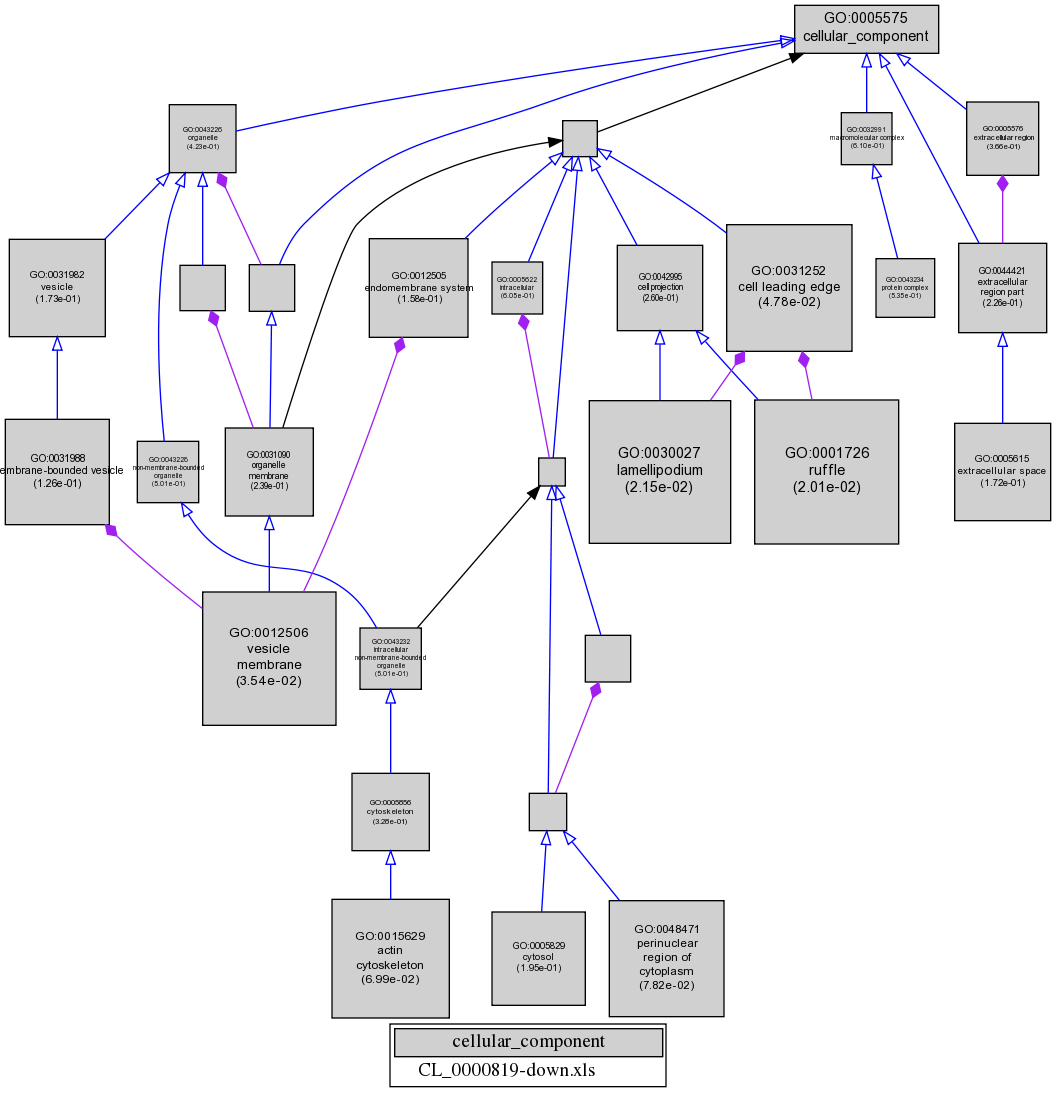

Supplement: Additional file 1 — OBAMS profiles for all mature B cells. Additional file 1 contains a zip archive of OBAMS profiles for all mature B cells, including for each cell type individual spreadsheets showing up and down regulated genes for that cell type relative to parental cell types, and VLAD (GO term enrichment) results for all mature B cells. [file 1471-2105-14-263-S1.zip › Additional File 1/B-1 B cell/VLAD.CL_0000819_down/auxfiles/vlad.results.cellular_component.png]

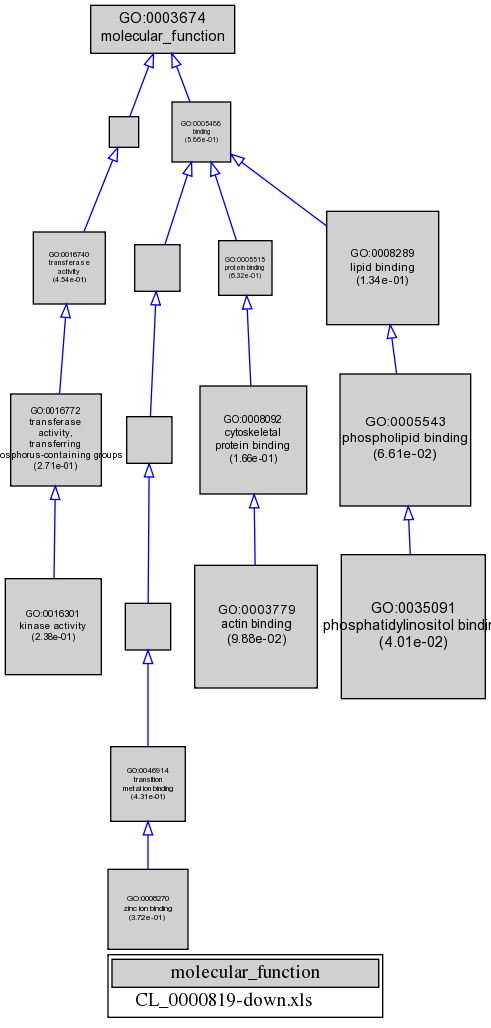

Supplement: Additional file 1 — OBAMS profiles for all mature B cells. Additional file 1 contains a zip archive of OBAMS profiles for all mature B cells, including for each cell type individual spreadsheets showing up and down regulated genes for that cell type relative to parental cell types, and VLAD (GO term enrichment) results for all mature B cells. [file 1471-2105-14-263-S1.zip › Additional File 1/B-1 B cell/VLAD.CL_0000819_down/auxfiles/vlad.results.molecular_function.png]

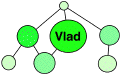

Supplement: Additional file 1 — OBAMS profiles for all mature B cells. Additional file 1 contains a zip archive of OBAMS profiles for all mature B cells, including for each cell type individual spreadsheets showing up and down regulated genes for that cell type relative to parental cell types, and VLAD (GO term enrichment) results for all mature B cells. [file 1471-2105-14-263-S1.zip › Additional File 1/B-1 B cell/VLAD.CL_0000819_down/auxfiles/vlad_logo.gif]

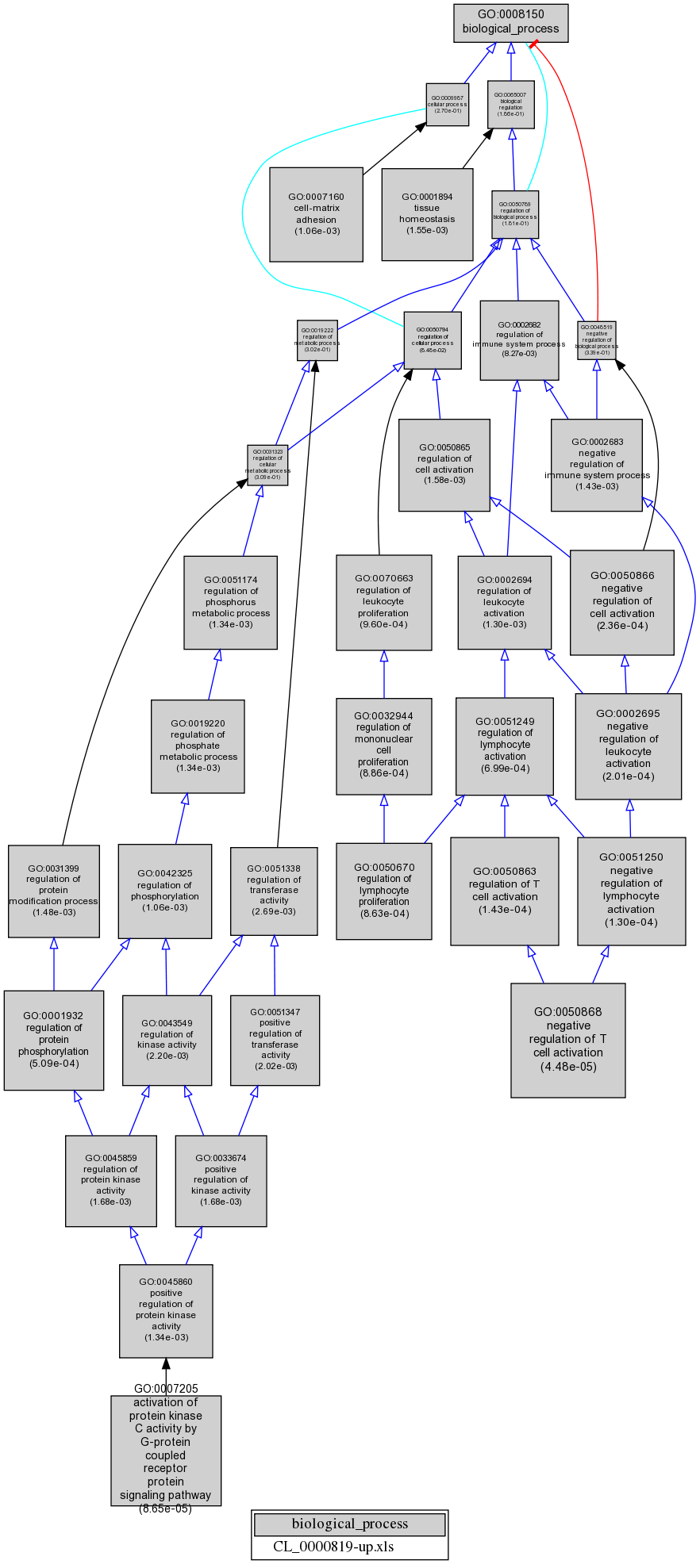

Supplement: Additional file 1 — OBAMS profiles for all mature B cells. Additional file 1 contains a zip archive of OBAMS profiles for all mature B cells, including for each cell type individual spreadsheets showing up and down regulated genes for that cell type relative to parental cell types, and VLAD (GO term enrichment) results for all mature B cells. [file 1471-2105-14-263-S1.zip › Additional File 1/B-1 B cell/VLAD.CL_0000819_up/auxfiles/vlad.results.biological_process.png]

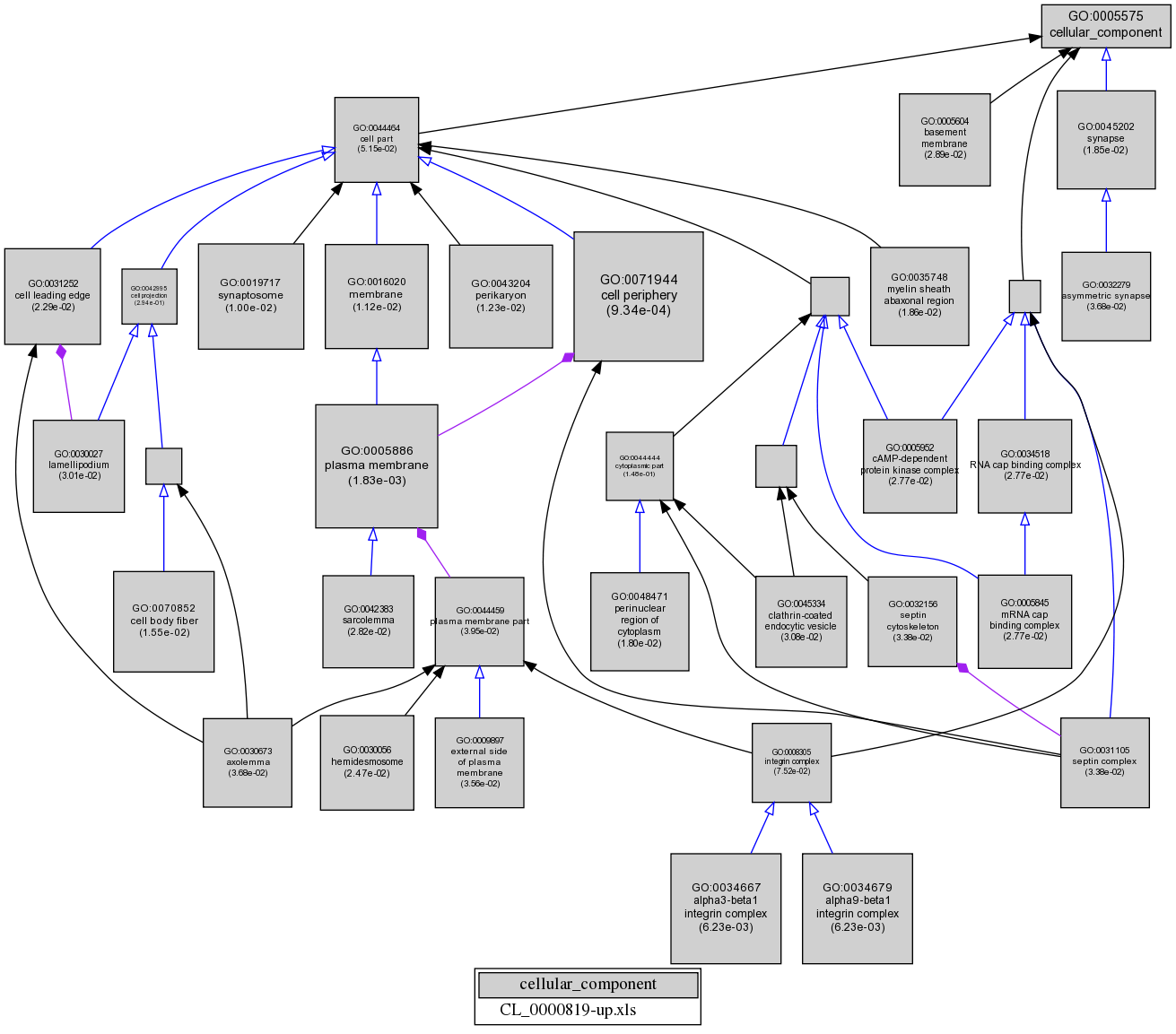

Supplement: Additional file 1 — OBAMS profiles for all mature B cells. Additional file 1 contains a zip archive of OBAMS profiles for all mature B cells, including for each cell type individual spreadsheets showing up and down regulated genes for that cell type relative to parental cell types, and VLAD (GO term enrichment) results for all mature B cells. [file 1471-2105-14-263-S1.zip › Additional File 1/B-1 B cell/VLAD.CL_0000819_up/auxfiles/vlad.results.cellular_component.png]

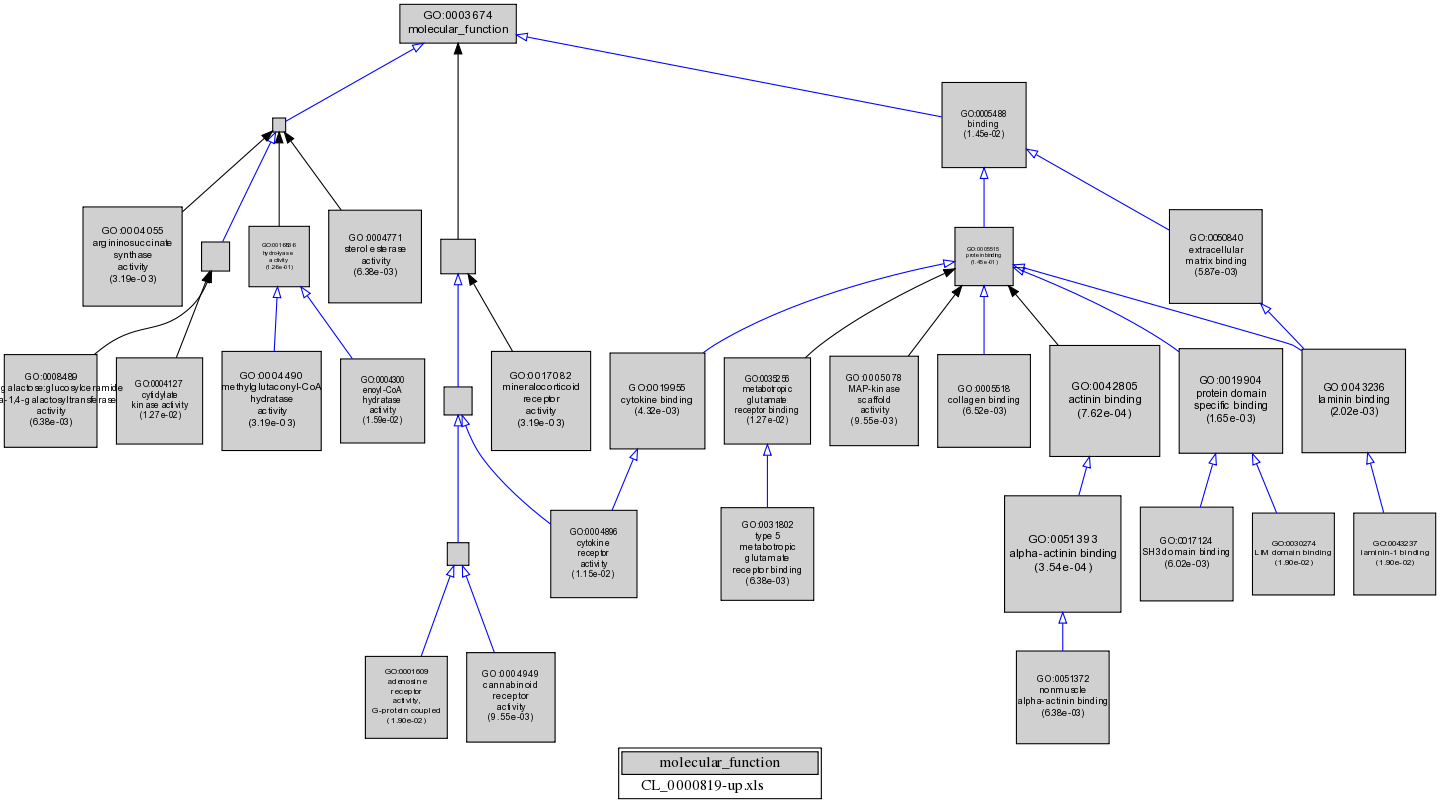

Supplement: Additional file 1 — OBAMS profiles for all mature B cells. Additional file 1 contains a zip archive of OBAMS profiles for all mature B cells, including for each cell type individual spreadsheets showing up and down regulated genes for that cell type relative to parental cell types, and VLAD (GO term enrichment) results for all mature B cells. [file 1471-2105-14-263-S1.zip › Additional File 1/B-1 B cell/VLAD.CL_0000819_up/auxfiles/vlad.results.molecular_function.png]

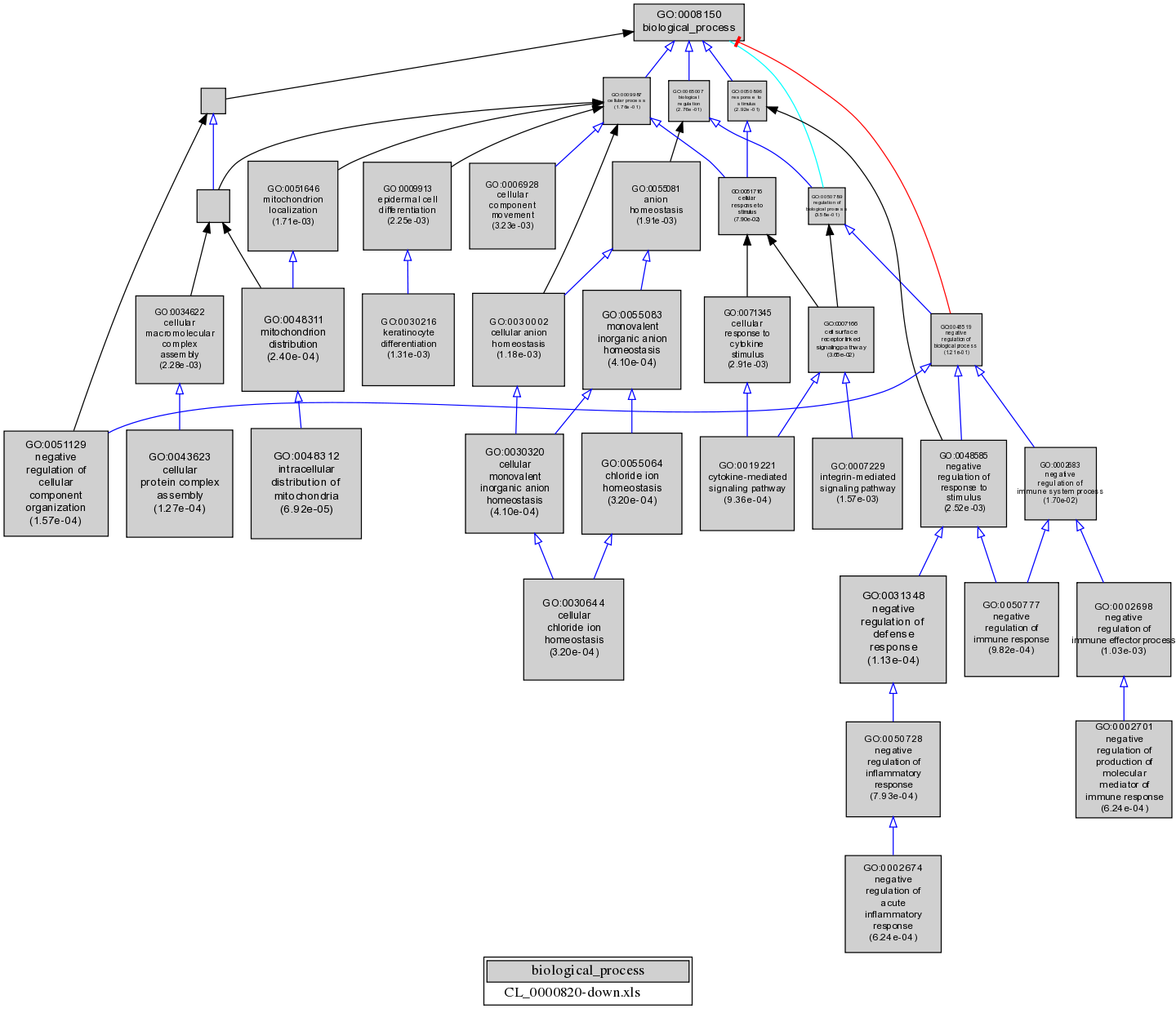

Supplement: Additional file 1 — OBAMS profiles for all mature B cells. Additional file 1 contains a zip archive of OBAMS profiles for all mature B cells, including for each cell type individual spreadsheets showing up and down regulated genes for that cell type relative to parental cell types, and VLAD (GO term enrichment) results for all mature B cells. [file 1471-2105-14-263-S1.zip › Additional File 1/B-1a B cell/VLAD.CL_0000820_down/auxfiles/vlad.results.biological_process.png]

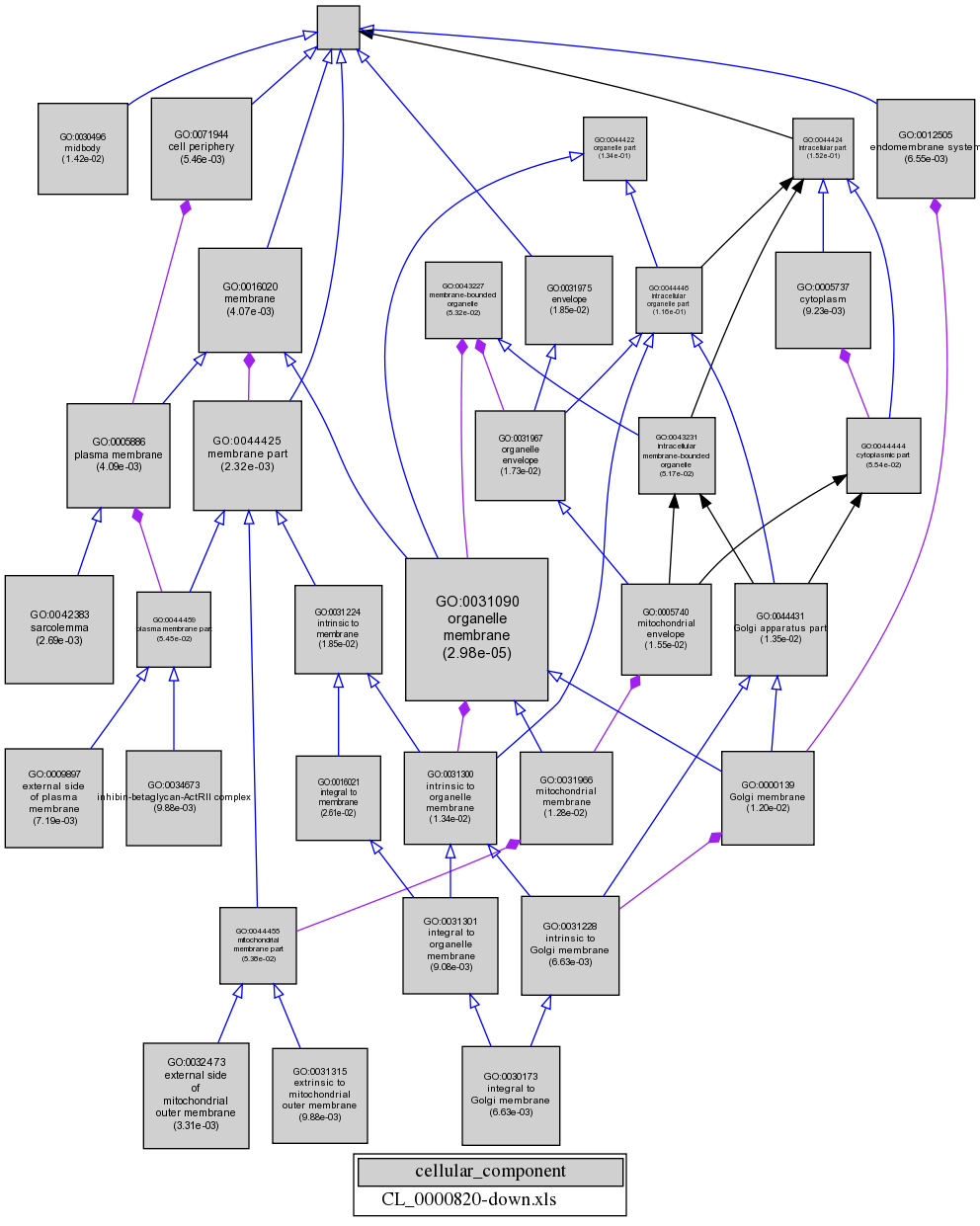

Supplement: Additional file 1 — OBAMS profiles for all mature B cells. Additional file 1 contains a zip archive of OBAMS profiles for all mature B cells, including for each cell type individual spreadsheets showing up and down regulated genes for that cell type relative to parental cell types, and VLAD (GO term enrichment) results for all mature B cells. [file 1471-2105-14-263-S1.zip › Additional File 1/B-1a B cell/VLAD.CL_0000820_down/auxfiles/vlad.results.cellular_component.png]

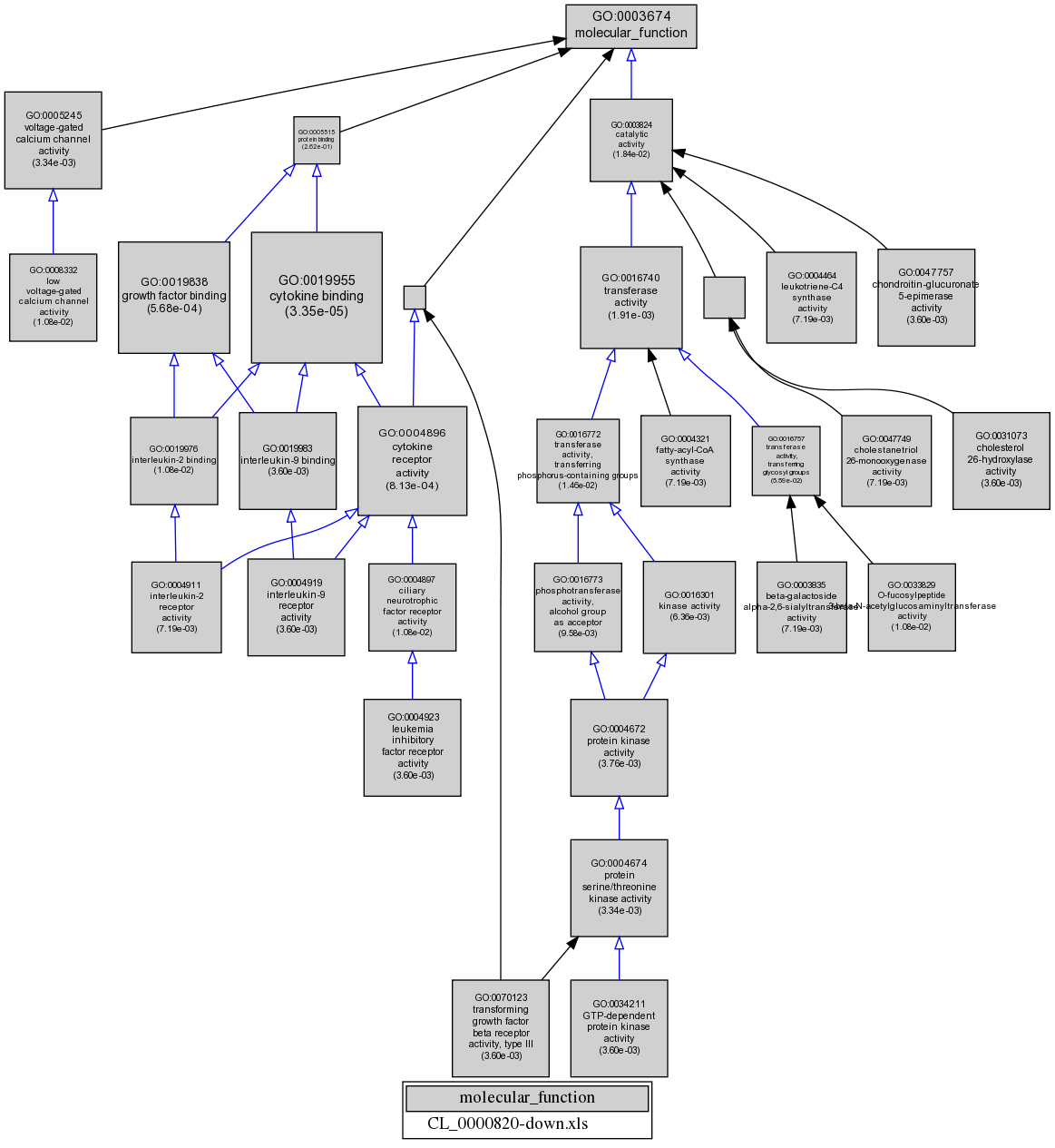

Supplement: Additional file 1 — OBAMS profiles for all mature B cells. Additional file 1 contains a zip archive of OBAMS profiles for all mature B cells, including for each cell type individual spreadsheets showing up and down regulated genes for that cell type relative to parental cell types, and VLAD (GO term enrichment) results for all mature B cells. [file 1471-2105-14-263-S1.zip › Additional File 1/B-1a B cell/VLAD.CL_0000820_down/auxfiles/vlad.results.molecular_function.png]

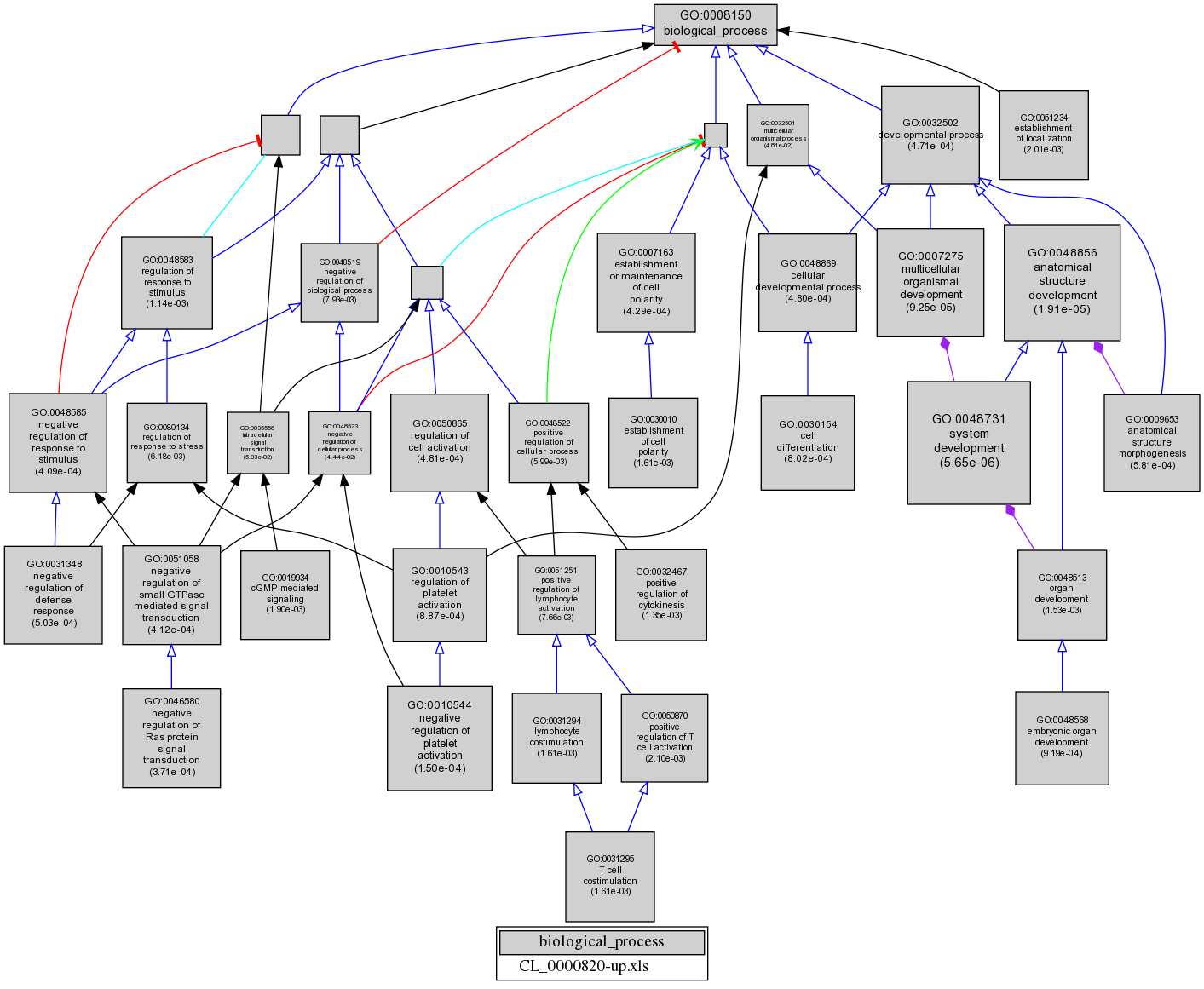

Supplement: Additional file 1 — OBAMS profiles for all mature B cells. Additional file 1 contains a zip archive of OBAMS profiles for all mature B cells, including for each cell type individual spreadsheets showing up and down regulated genes for that cell type relative to parental cell types, and VLAD (GO term enrichment) results for all mature B cells. [file 1471-2105-14-263-S1.zip › Additional File 1/B-1a B cell/VLAD.CL_0000820_up/auxfiles/vlad.results.biological_process.png]

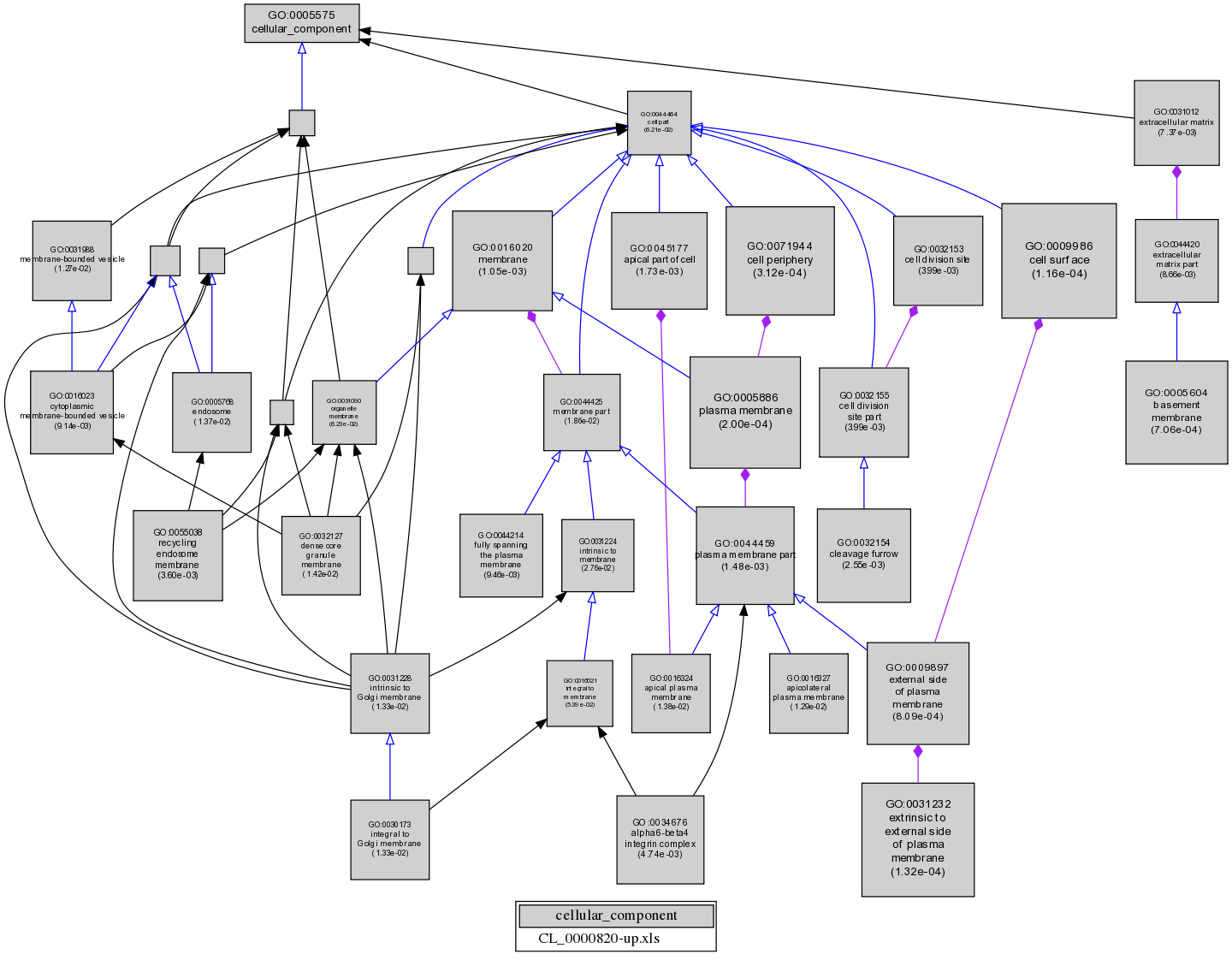

Supplement: Additional file 1 — OBAMS profiles for all mature B cells. Additional file 1 contains a zip archive of OBAMS profiles for all mature B cells, including for each cell type individual spreadsheets showing up and down regulated genes for that cell type relative to parental cell types, and VLAD (GO term enrichment) results for all mature B cells. [file 1471-2105-14-263-S1.zip › Additional File 1/B-1a B cell/VLAD.CL_0000820_up/auxfiles/vlad.results.cellular_component.png]

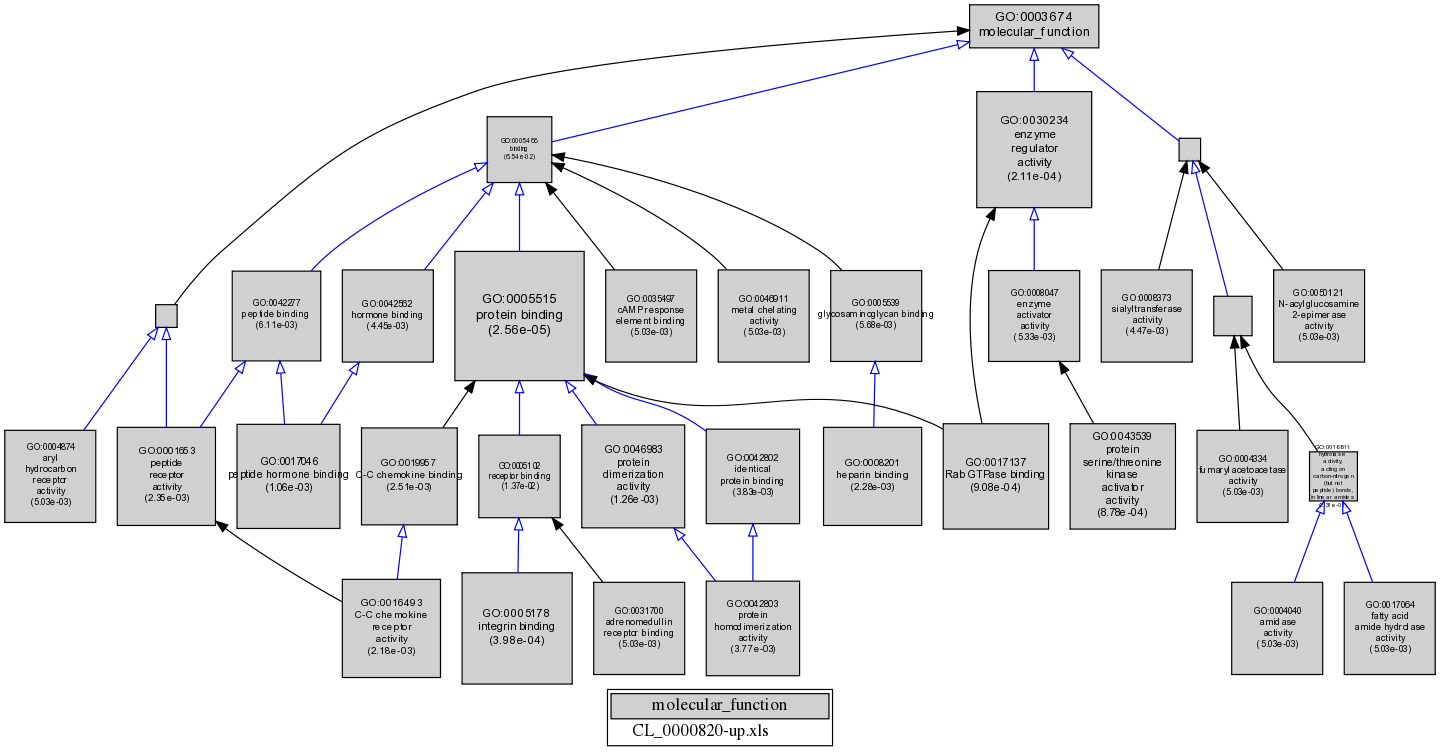

Supplement: Additional file 1 — OBAMS profiles for all mature B cells. Additional file 1 contains a zip archive of OBAMS profiles for all mature B cells, including for each cell type individual spreadsheets showing up and down regulated genes for that cell type relative to parental cell types, and VLAD (GO term enrichment) results for all mature B cells. [file 1471-2105-14-263-S1.zip › Additional File 1/B-1a B cell/VLAD.CL_0000820_up/auxfiles/vlad.results.molecular_function.png]

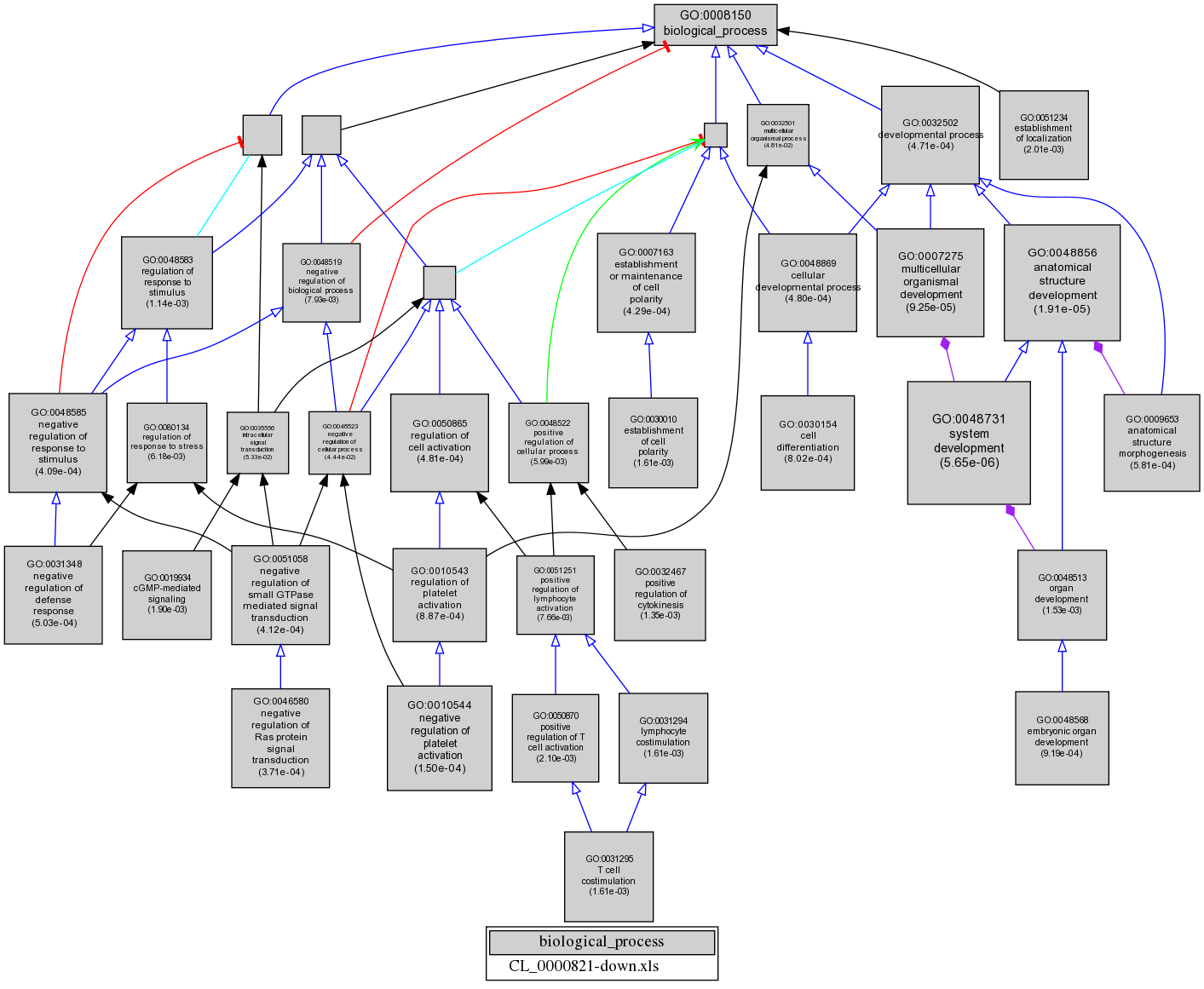

Supplement: Additional file 1 — OBAMS profiles for all mature B cells. Additional file 1 contains a zip archive of OBAMS profiles for all mature B cells, including for each cell type individual spreadsheets showing up and down regulated genes for that cell type relative to parental cell types, and VLAD (GO term enrichment) results for all mature B cells. [file 1471-2105-14-263-S1.zip › Additional File 1/B-1b B cell/VLAD.CL_0000821_down/auxfiles/vlad.results.biological_process.png]

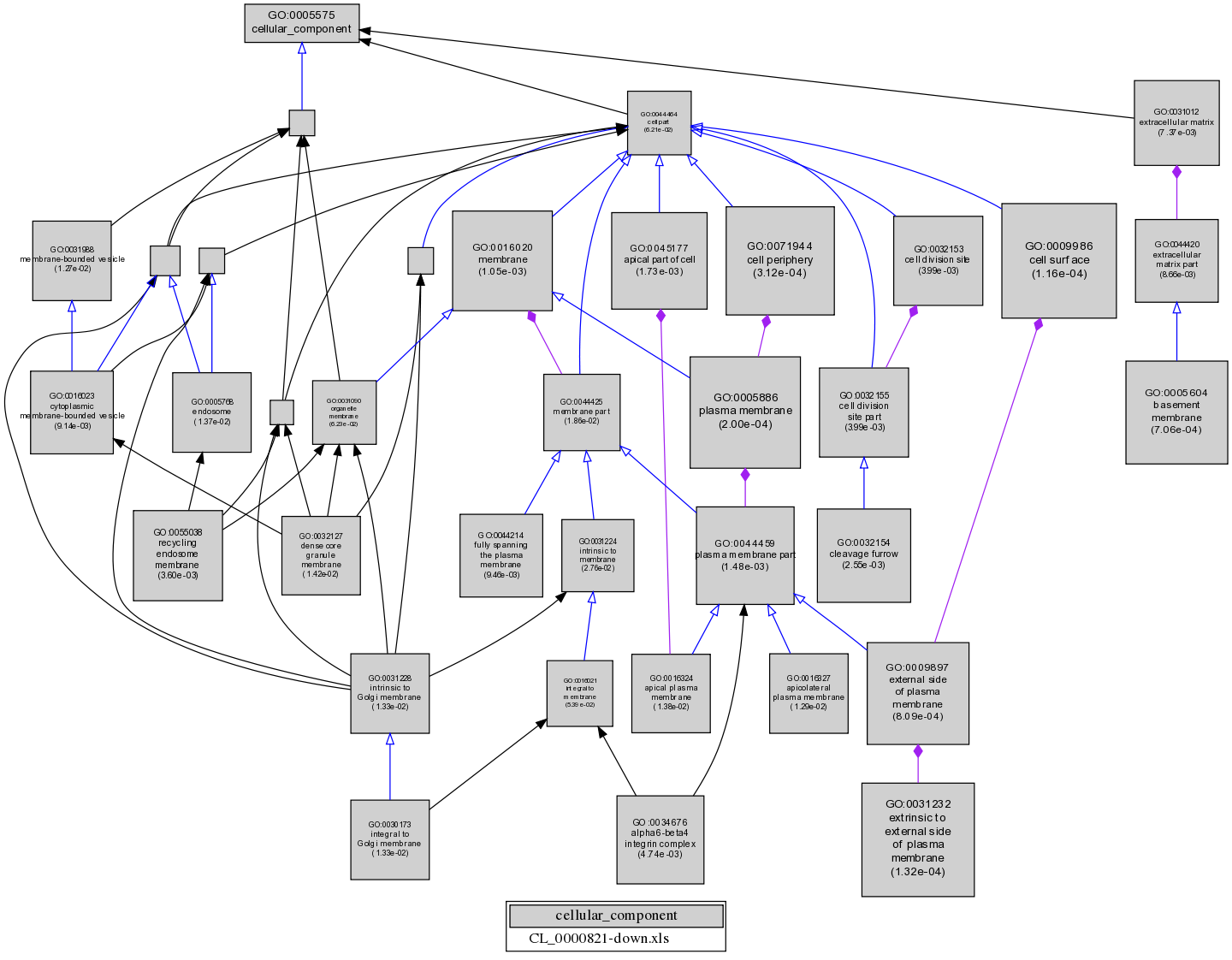

Supplement: Additional file 1 — OBAMS profiles for all mature B cells. Additional file 1 contains a zip archive of OBAMS profiles for all mature B cells, including for each cell type individual spreadsheets showing up and down regulated genes for that cell type relative to parental cell types, and VLAD (GO term enrichment) results for all mature B cells. [file 1471-2105-14-263-S1.zip › Additional File 1/B-1b B cell/VLAD.CL_0000821_down/auxfiles/vlad.results.cellular_component.png]

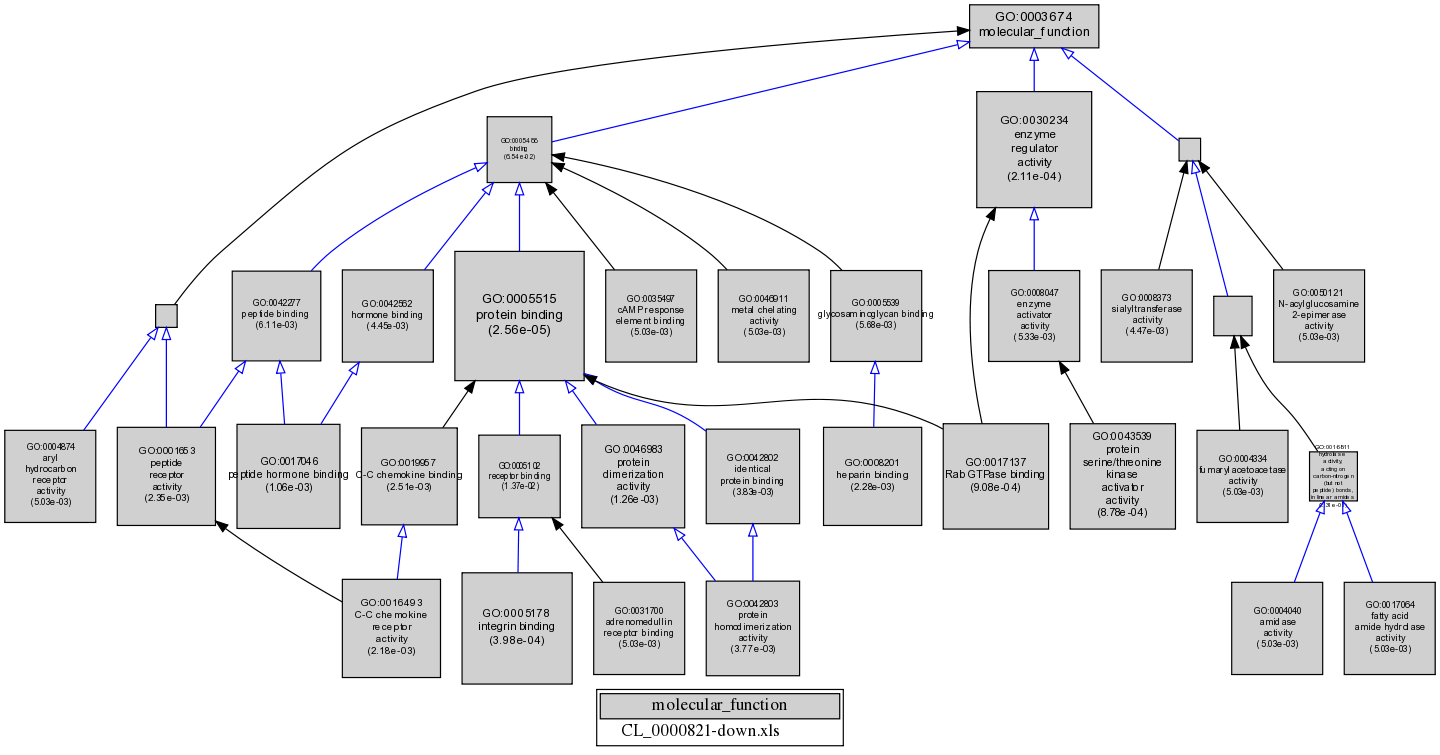

Supplement: Additional file 1 — OBAMS profiles for all mature B cells. Additional file 1 contains a zip archive of OBAMS profiles for all mature B cells, including for each cell type individual spreadsheets showing up and down regulated genes for that cell type relative to parental cell types, and VLAD (GO term enrichment) results for all mature B cells. [file 1471-2105-14-263-S1.zip › Additional File 1/B-1b B cell/VLAD.CL_0000821_down/auxfiles/vlad.results.molecular_function.png]

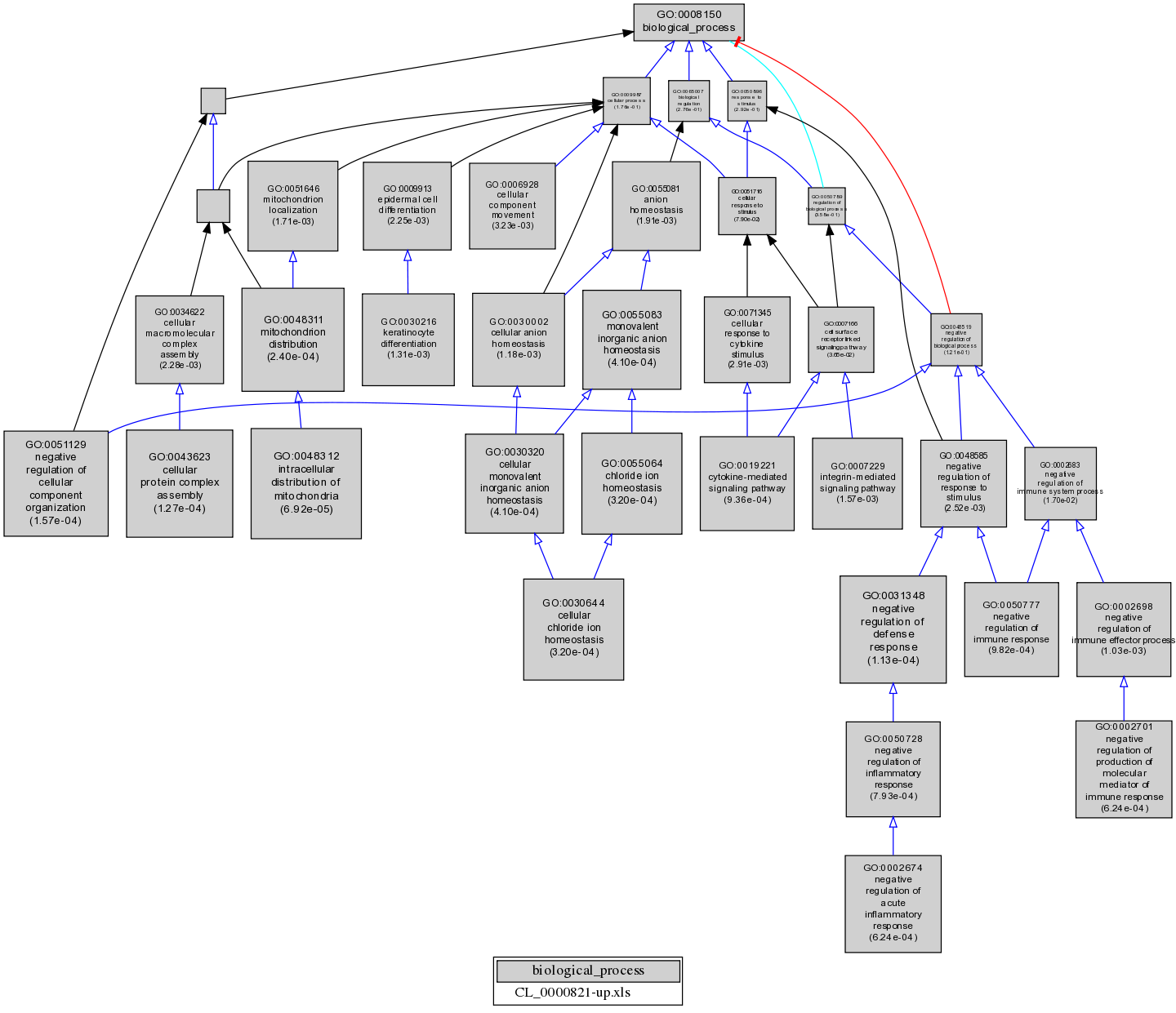

Supplement: Additional file 1 — OBAMS profiles for all mature B cells. Additional file 1 contains a zip archive of OBAMS profiles for all mature B cells, including for each cell type individual spreadsheets showing up and down regulated genes for that cell type relative to parental cell types, and VLAD (GO term enrichment) results for all mature B cells. [file 1471-2105-14-263-S1.zip › Additional File 1/B-1b B cell/VLAD.CL_0000821_up/auxfiles/vlad.results.biological_process.png]

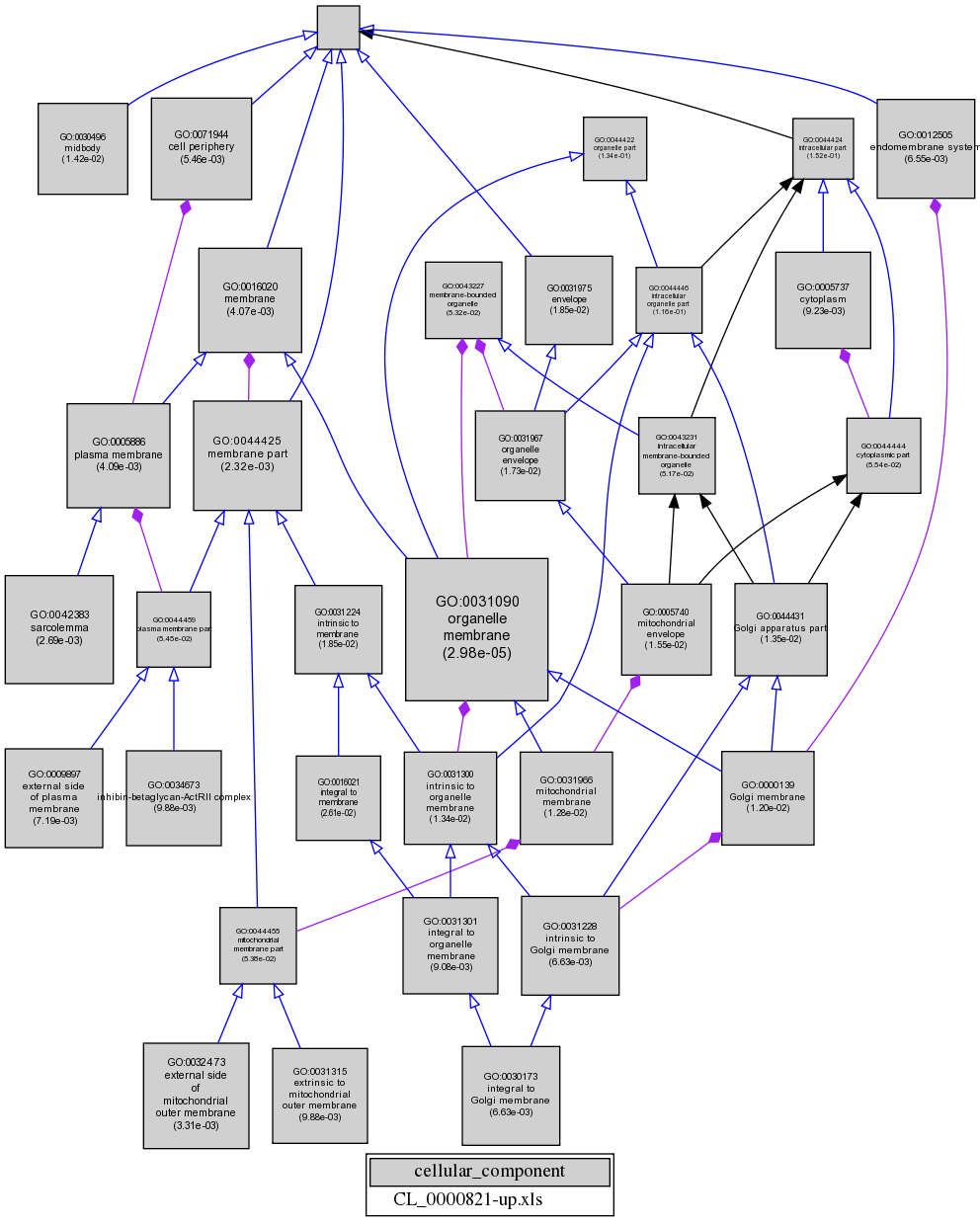

Supplement: Additional file 1 — OBAMS profiles for all mature B cells. Additional file 1 contains a zip archive of OBAMS profiles for all mature B cells, including for each cell type individual spreadsheets showing up and down regulated genes for that cell type relative to parental cell types, and VLAD (GO term enrichment) results for all mature B cells. [file 1471-2105-14-263-S1.zip › Additional File 1/B-1b B cell/VLAD.CL_0000821_up/auxfiles/vlad.results.cellular_component.png]

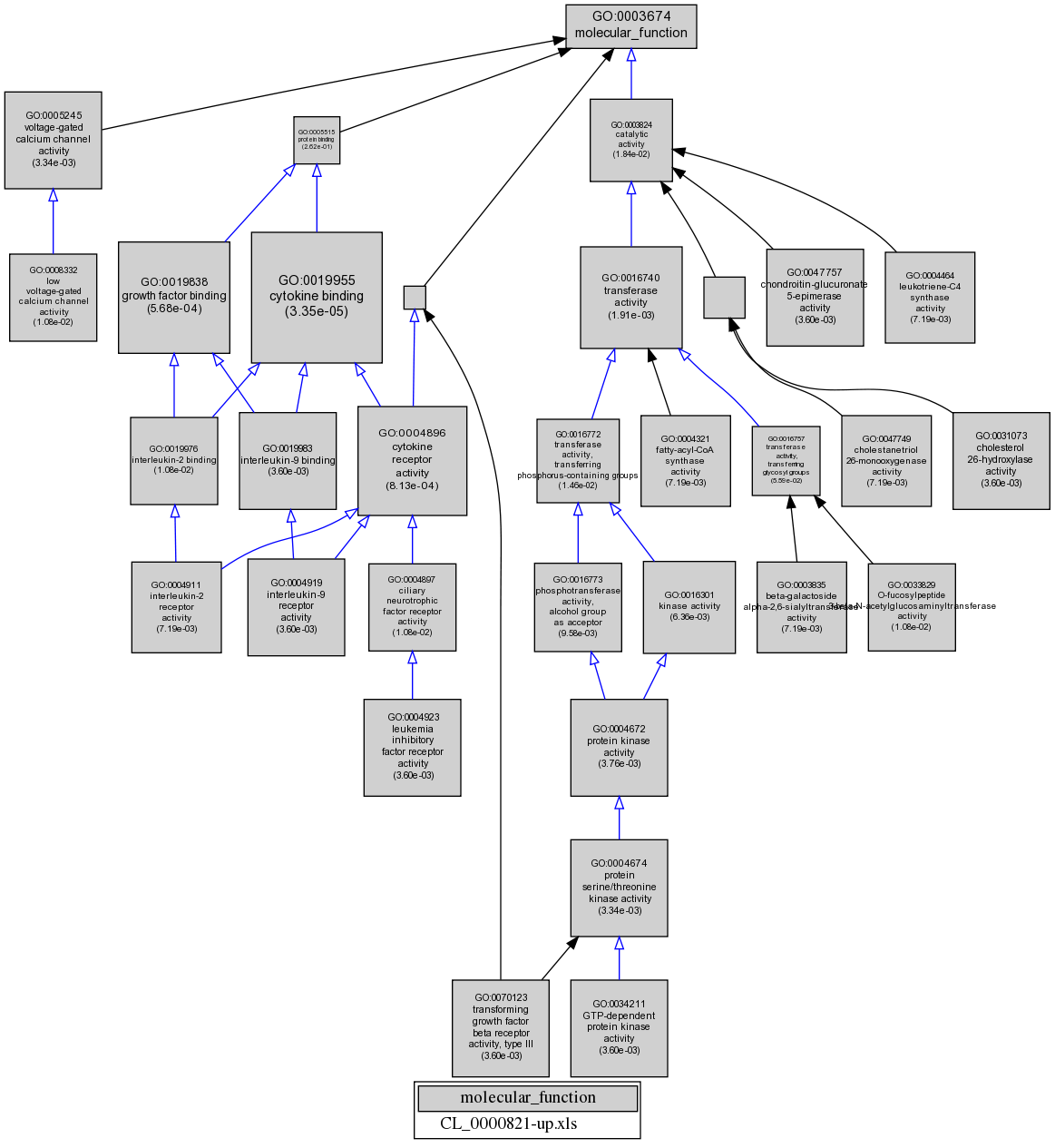

Supplement: Additional file 1 — OBAMS profiles for all mature B cells. Additional file 1 contains a zip archive of OBAMS profiles for all mature B cells, including for each cell type individual spreadsheets showing up and down regulated genes for that cell type relative to parental cell types, and VLAD (GO term enrichment) results for all mature B cells. [file 1471-2105-14-263-S1.zip › Additional File 1/B-1b B cell/VLAD.CL_0000821_up/auxfiles/vlad.results.molecular_function.png]

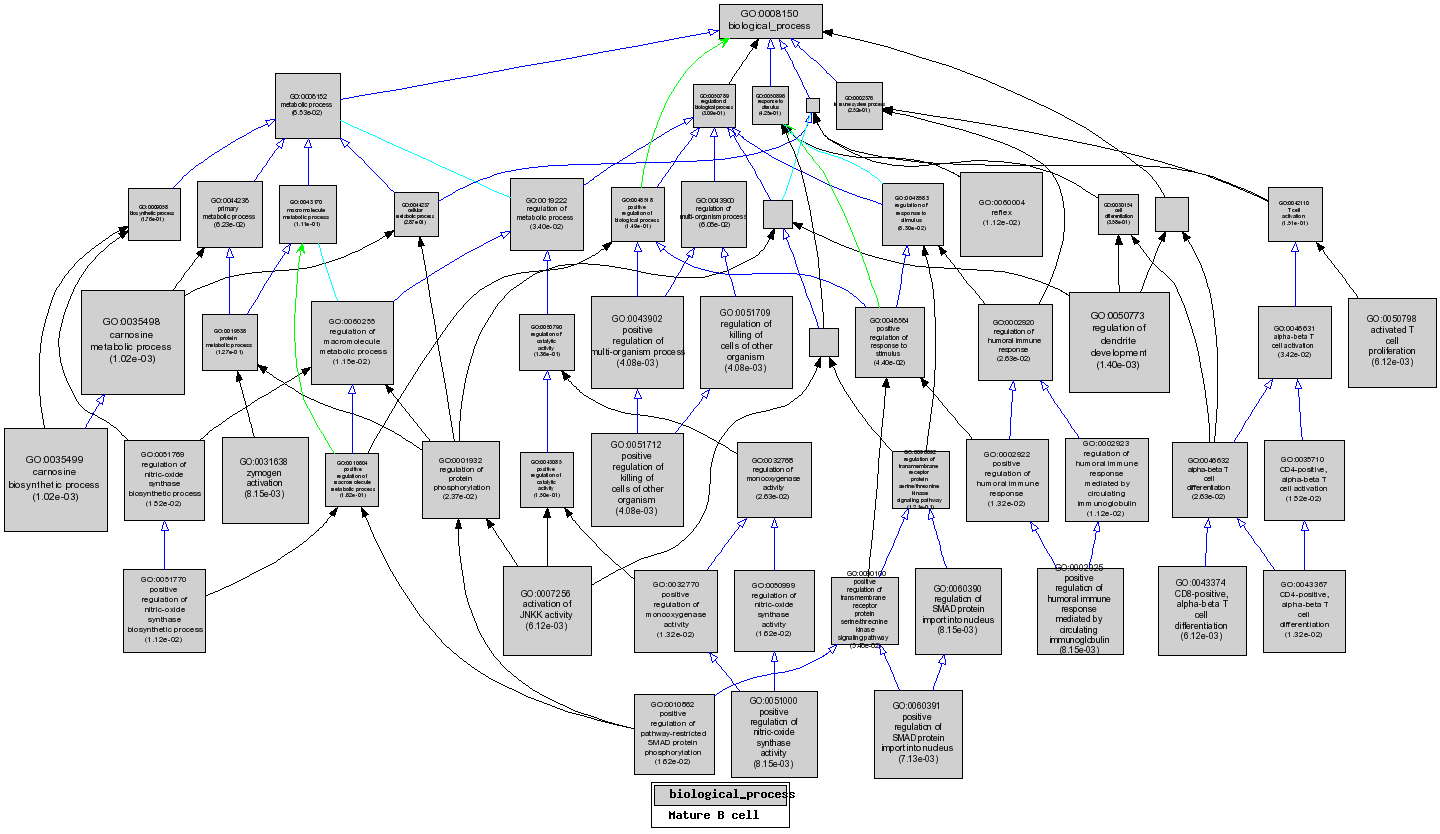

Supplement: Additional file 1 — OBAMS profiles for all mature B cells. Additional file 1 contains a zip archive of OBAMS profiles for all mature B cells, including for each cell type individual spreadsheets showing up and down regulated genes for that cell type relative to parental cell types, and VLAD (GO term enrichment) results for all mature B cells. [file 1471-2105-14-263-S1.zip › Additional File 1/B-2 B cell/VLAD.CL_0000822-up/auxfiles/vlad.results.biological_process.png]

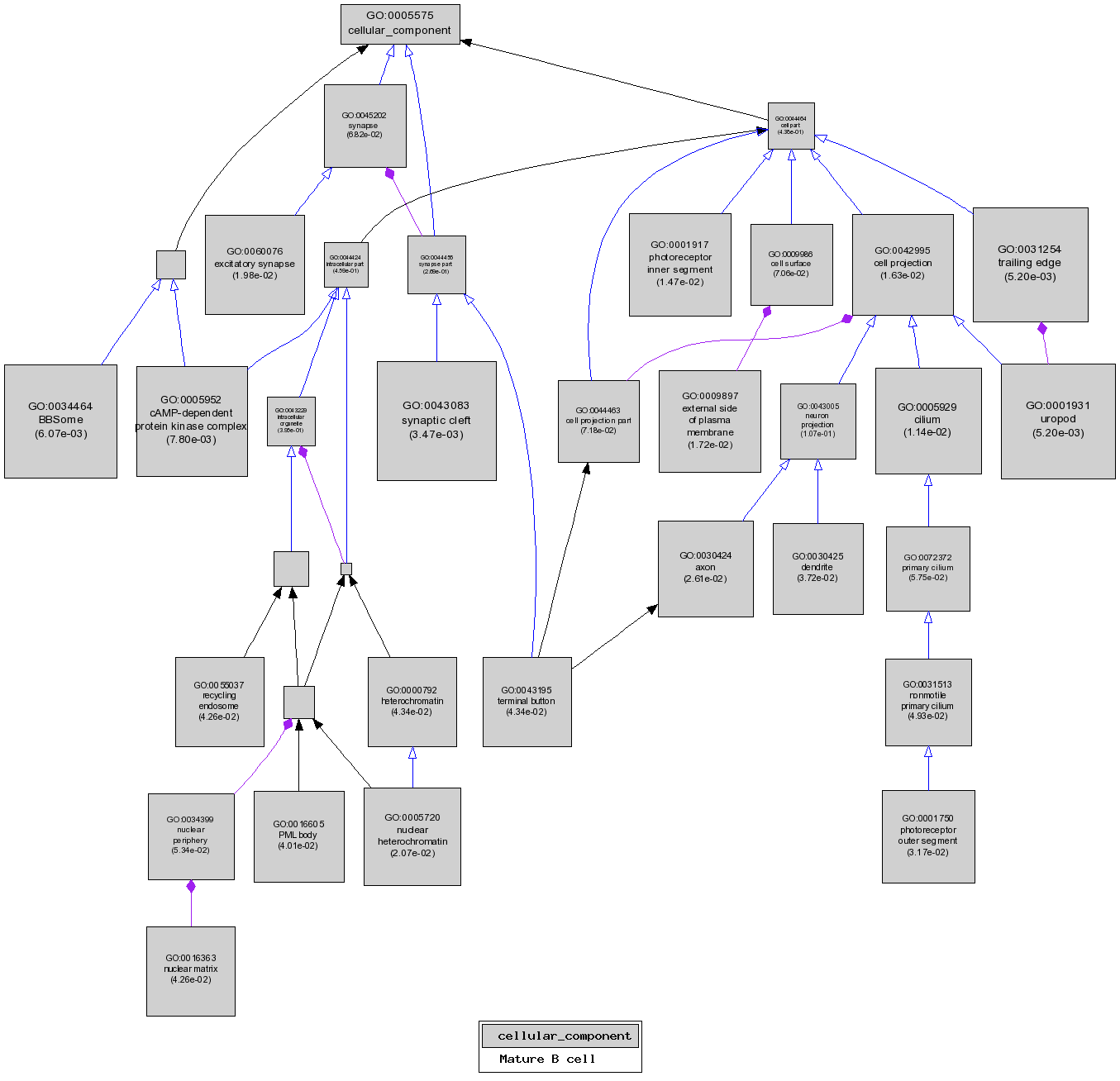

Supplement: Additional file 1 — OBAMS profiles for all mature B cells. Additional file 1 contains a zip archive of OBAMS profiles for all mature B cells, including for each cell type individual spreadsheets showing up and down regulated genes for that cell type relative to parental cell types, and VLAD (GO term enrichment) results for all mature B cells. [file 1471-2105-14-263-S1.zip › Additional File 1/B-2 B cell/VLAD.CL_0000822-up/auxfiles/vlad.results.cellular_component.png]

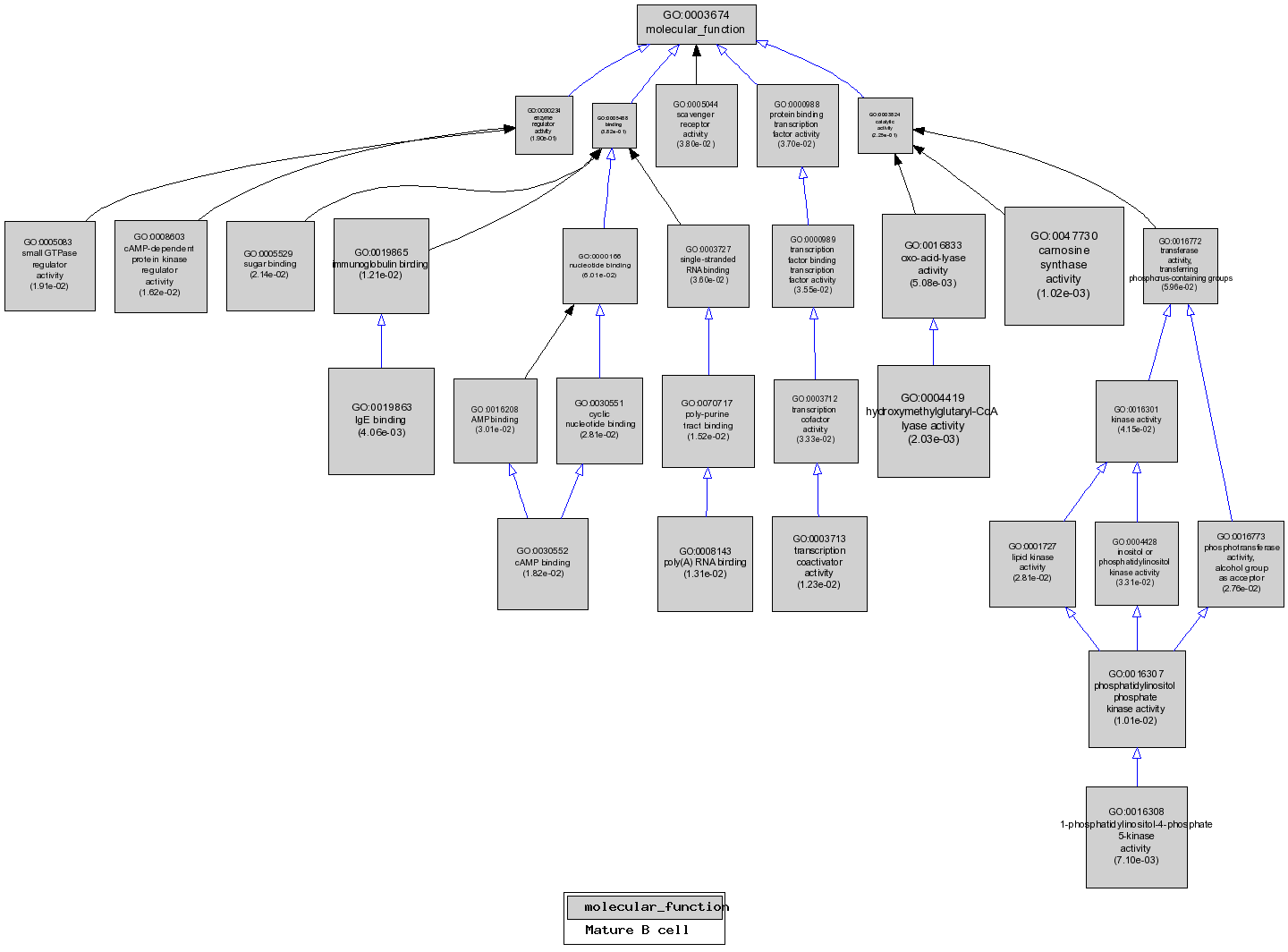

Supplement: Additional file 1 — OBAMS profiles for all mature B cells. Additional file 1 contains a zip archive of OBAMS profiles for all mature B cells, including for each cell type individual spreadsheets showing up and down regulated genes for that cell type relative to parental cell types, and VLAD (GO term enrichment) results for all mature B cells. [file 1471-2105-14-263-S1.zip › Additional File 1/B-2 B cell/VLAD.CL_0000822-up/auxfiles/vlad.results.molecular_function.png]

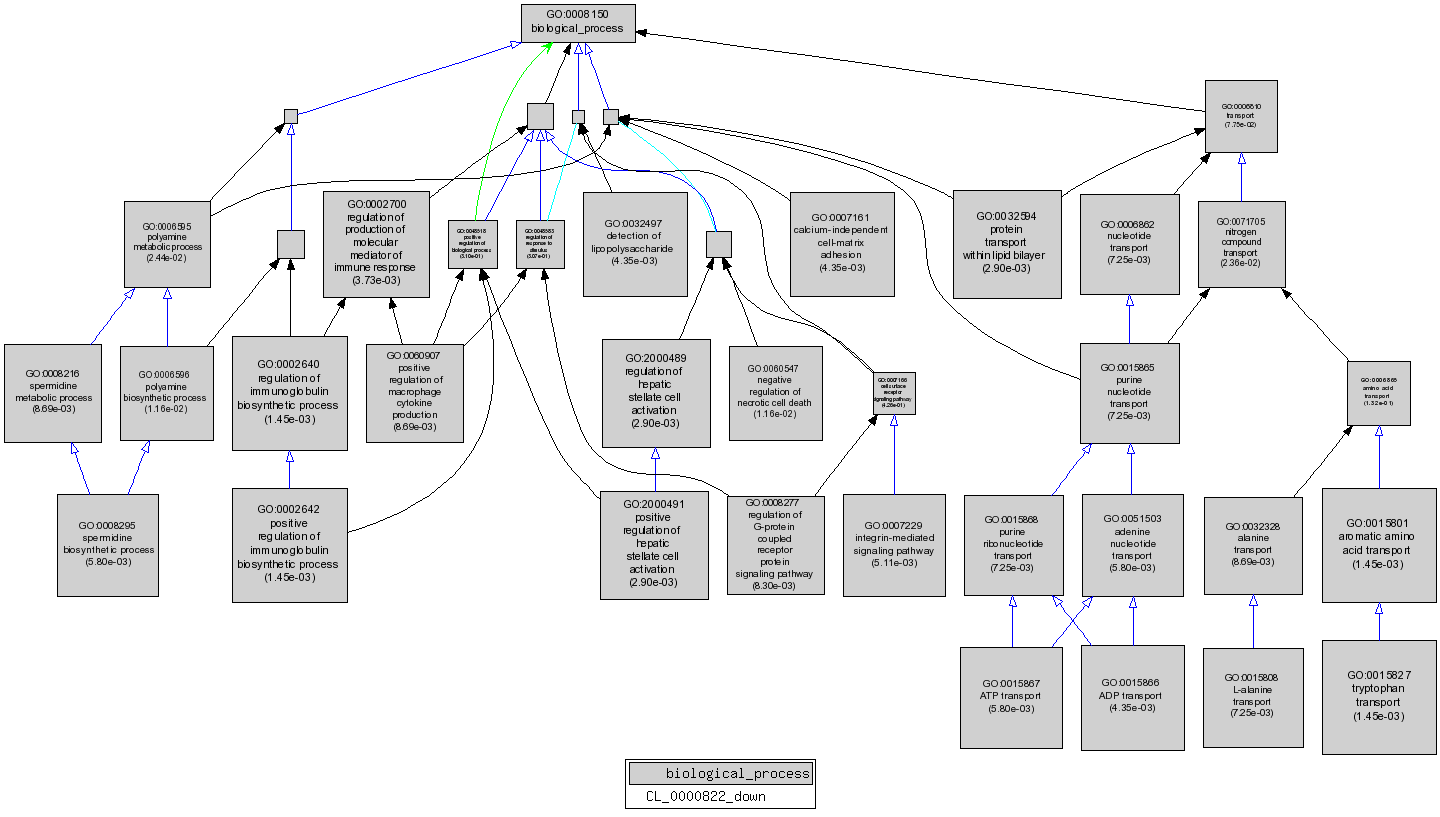

Supplement: Additional file 1 — OBAMS profiles for all mature B cells. Additional file 1 contains a zip archive of OBAMS profiles for all mature B cells, including for each cell type individual spreadsheets showing up and down regulated genes for that cell type relative to parental cell types, and VLAD (GO term enrichment) results for all mature B cells. [file 1471-2105-14-263-S1.zip › Additional File 1/B-2 B cell/VLAD.CL_0000822_down/auxfiles/vlad.results.biological_process.png]

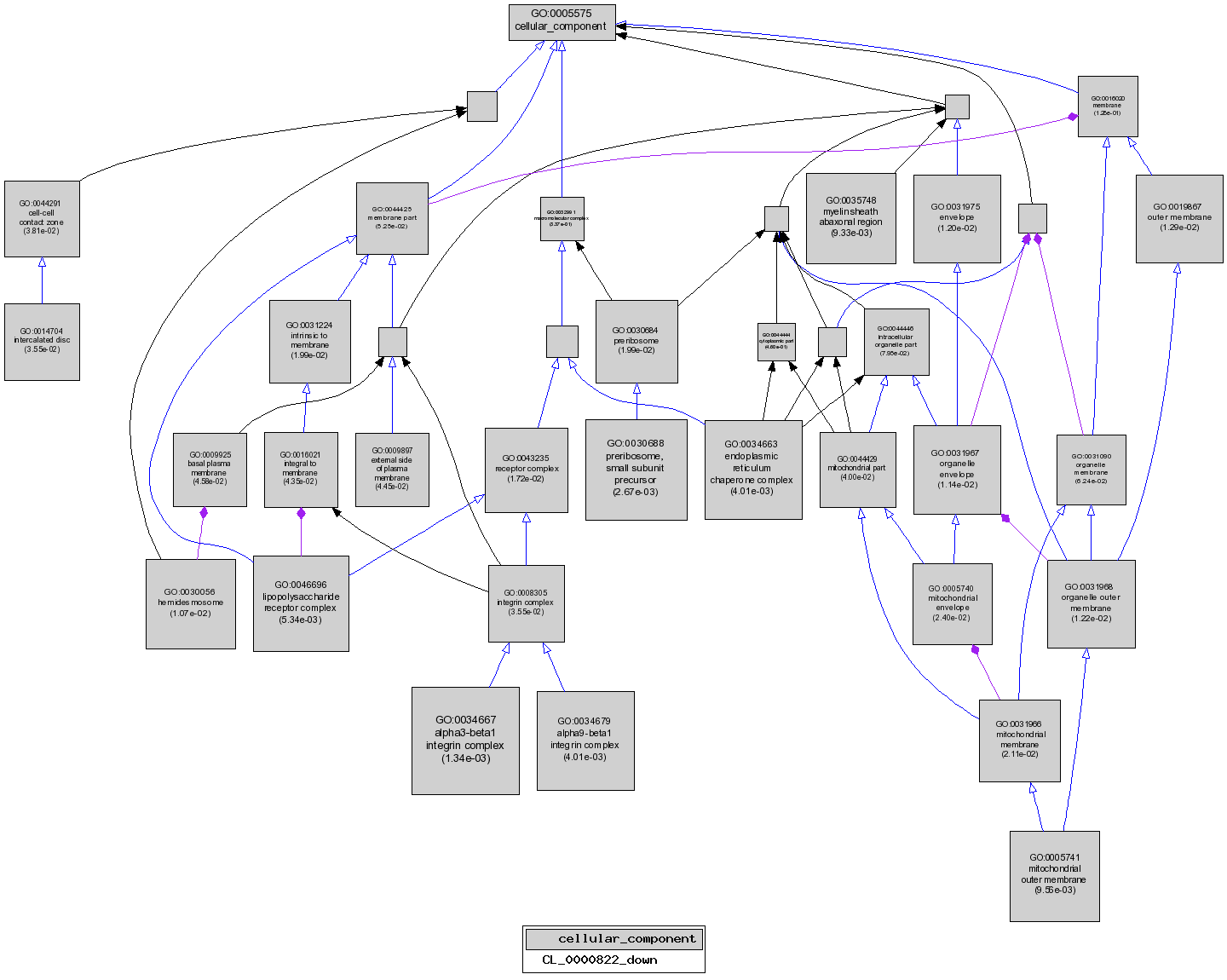

Supplement: Additional file 1 — OBAMS profiles for all mature B cells. Additional file 1 contains a zip archive of OBAMS profiles for all mature B cells, including for each cell type individual spreadsheets showing up and down regulated genes for that cell type relative to parental cell types, and VLAD (GO term enrichment) results for all mature B cells. [file 1471-2105-14-263-S1.zip › Additional File 1/B-2 B cell/VLAD.CL_0000822_down/auxfiles/vlad.results.cellular_component.png]

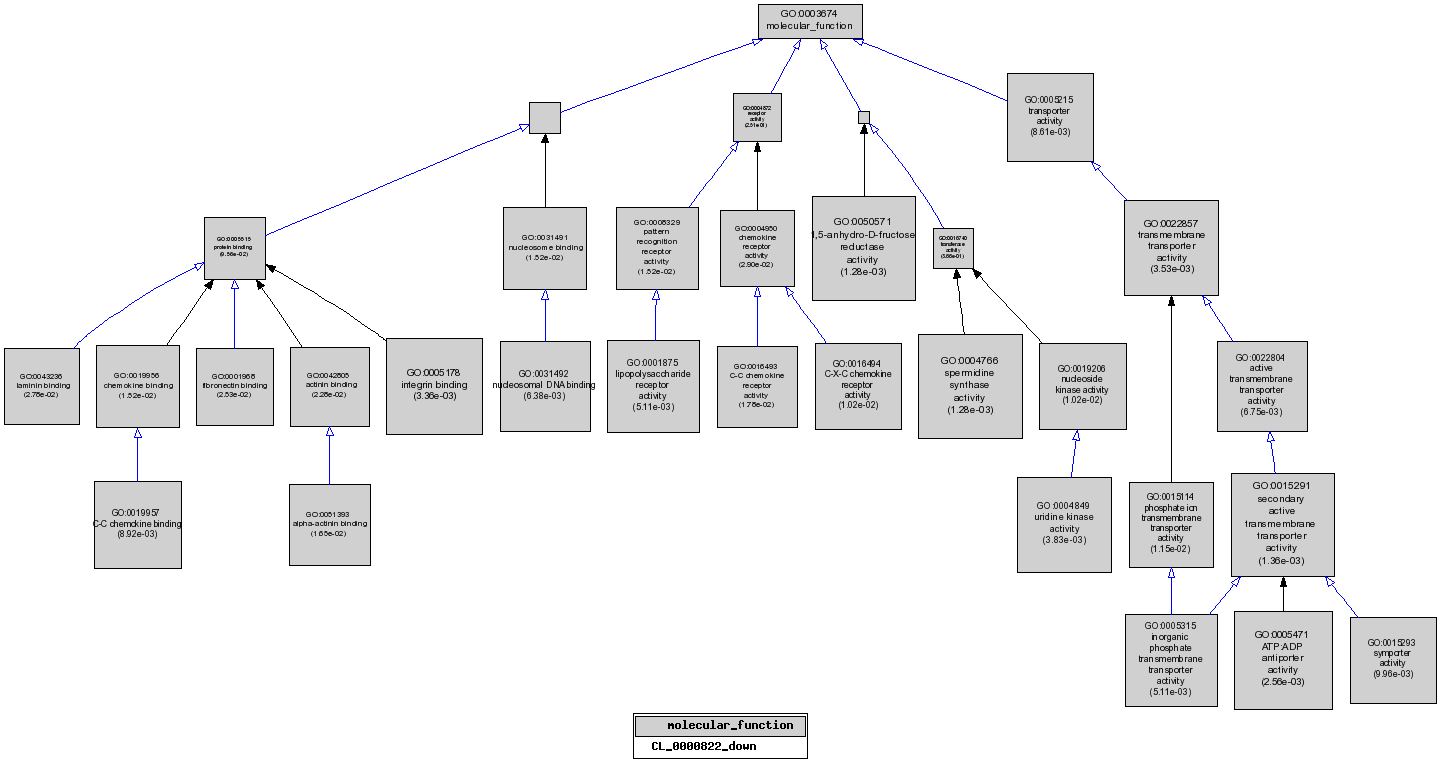

Supplement: Additional file 1 — OBAMS profiles for all mature B cells. Additional file 1 contains a zip archive of OBAMS profiles for all mature B cells, including for each cell type individual spreadsheets showing up and down regulated genes for that cell type relative to parental cell types, and VLAD (GO term enrichment) results for all mature B cells. [file 1471-2105-14-263-S1.zip › Additional File 1/B-2 B cell/VLAD.CL_0000822_down/auxfiles/vlad.results.molecular_function.png]

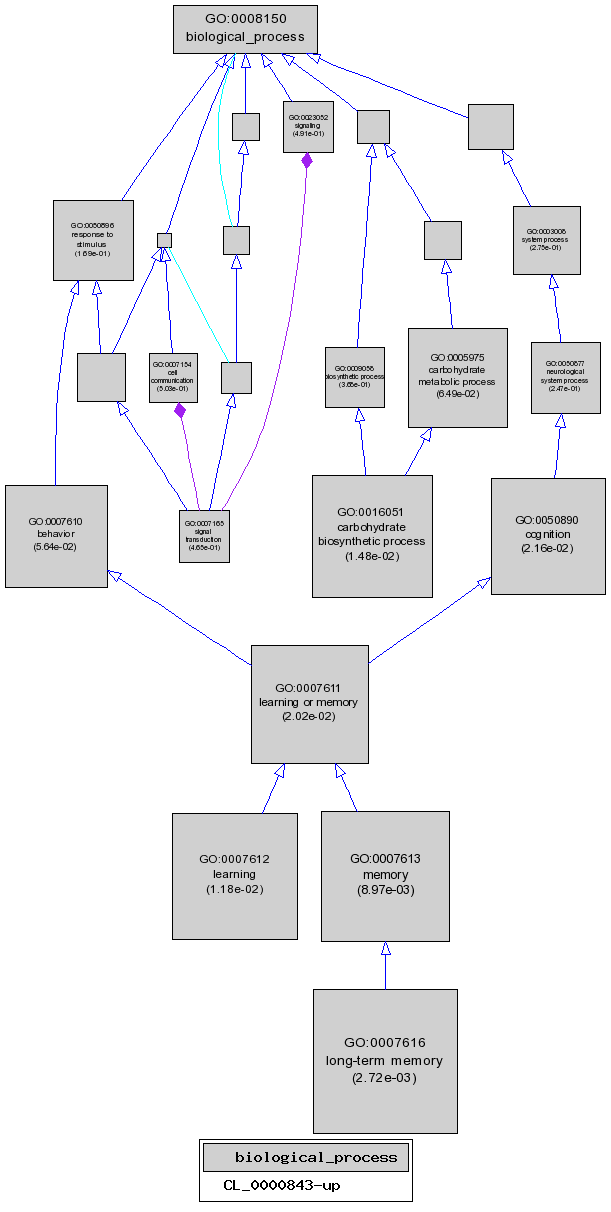

Supplement: Additional file 1 — OBAMS profiles for all mature B cells. Additional file 1 contains a zip archive of OBAMS profiles for all mature B cells, including for each cell type individual spreadsheets showing up and down regulated genes for that cell type relative to parental cell types, and VLAD (GO term enrichment) results for all mature B cells. [file 1471-2105-14-263-S1.zip › Additional File 1/follicular B cell/VLAD.CL_0000843-up/auxfiles/vlad.results.biological_process.png]

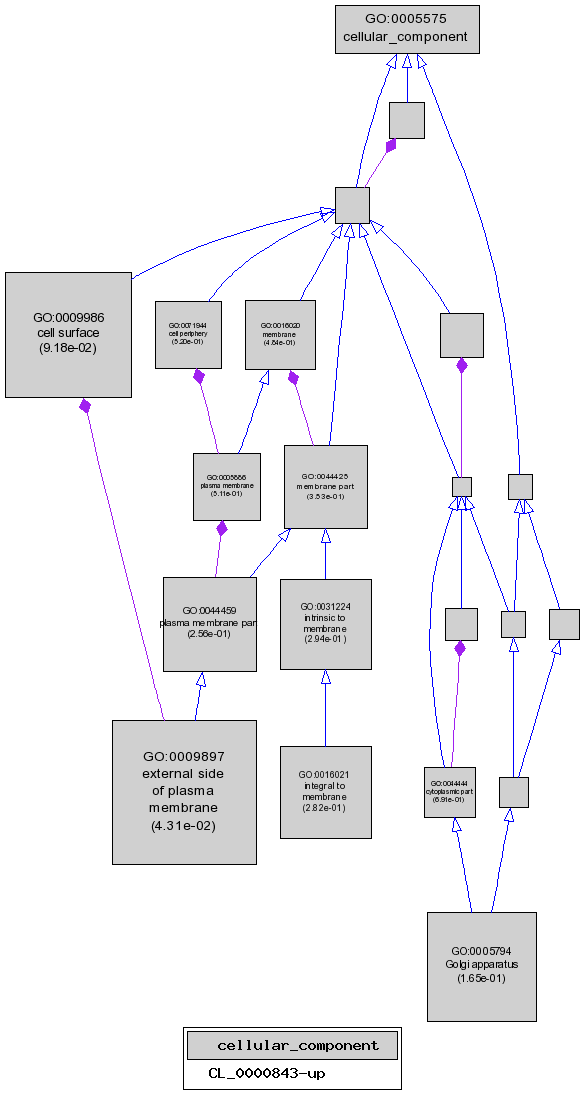

Supplement: Additional file 1 — OBAMS profiles for all mature B cells. Additional file 1 contains a zip archive of OBAMS profiles for all mature B cells, including for each cell type individual spreadsheets showing up and down regulated genes for that cell type relative to parental cell types, and VLAD (GO term enrichment) results for all mature B cells. [file 1471-2105-14-263-S1.zip › Additional File 1/follicular B cell/VLAD.CL_0000843-up/auxfiles/vlad.results.cellular_component.png]

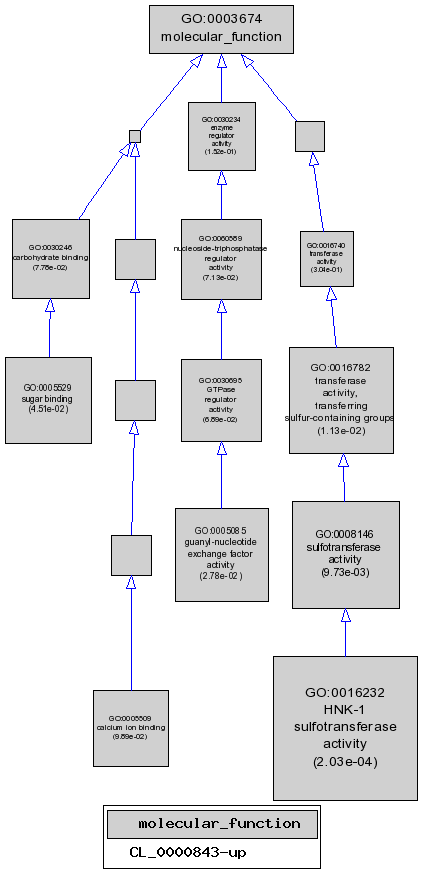

Supplement: Additional file 1 — OBAMS profiles for all mature B cells. Additional file 1 contains a zip archive of OBAMS profiles for all mature B cells, including for each cell type individual spreadsheets showing up and down regulated genes for that cell type relative to parental cell types, and VLAD (GO term enrichment) results for all mature B cells. [file 1471-2105-14-263-S1.zip › Additional File 1/follicular B cell/VLAD.CL_0000843-up/auxfiles/vlad.results.molecular_function.png]

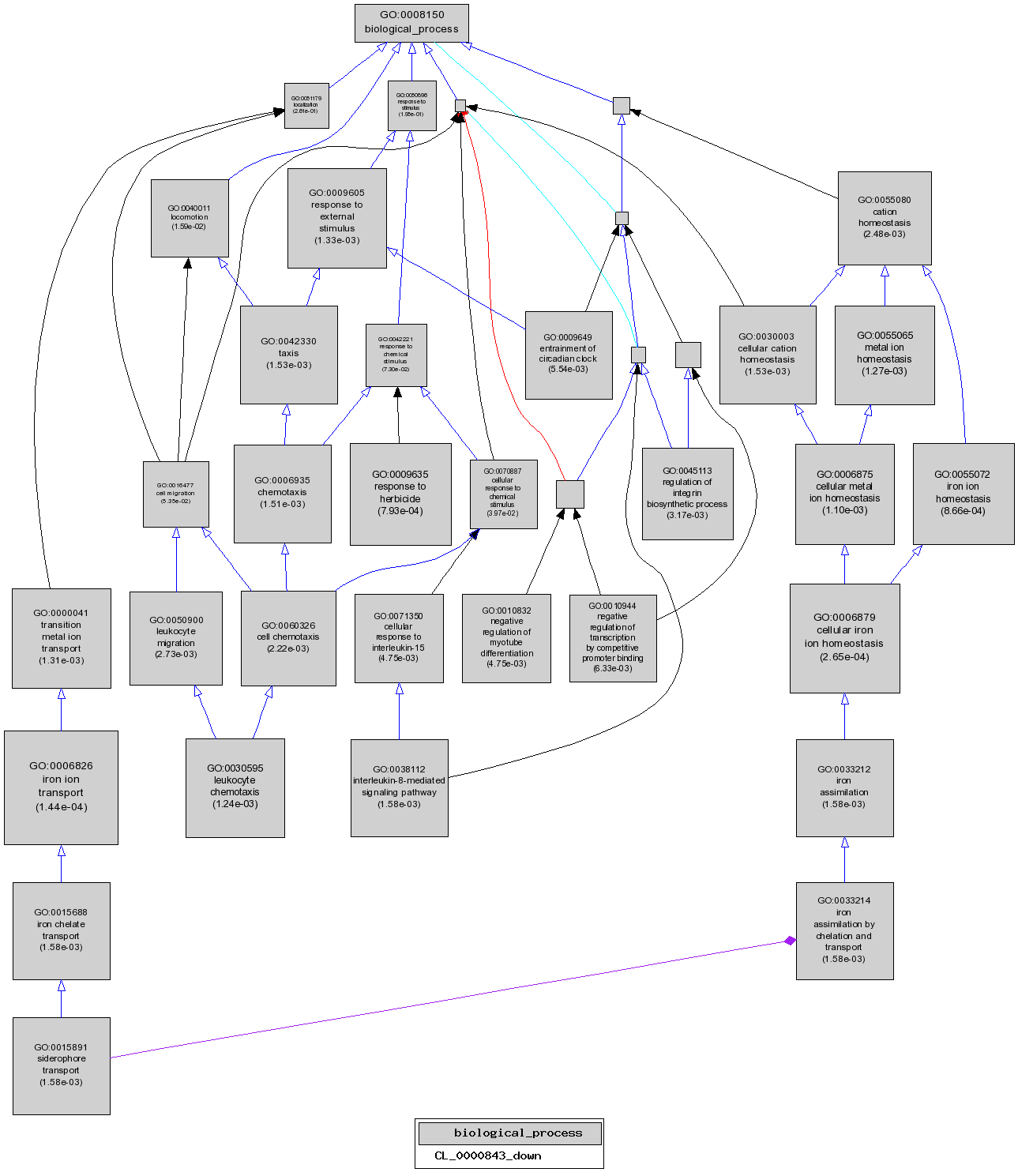

Supplement: Additional file 1 — OBAMS profiles for all mature B cells. Additional file 1 contains a zip archive of OBAMS profiles for all mature B cells, including for each cell type individual spreadsheets showing up and down regulated genes for that cell type relative to parental cell types, and VLAD (GO term enrichment) results for all mature B cells. [file 1471-2105-14-263-S1.zip › Additional File 1/follicular B cell/VLAD.CL_0000843_down/auxfiles/vlad.results.biological_process.png]

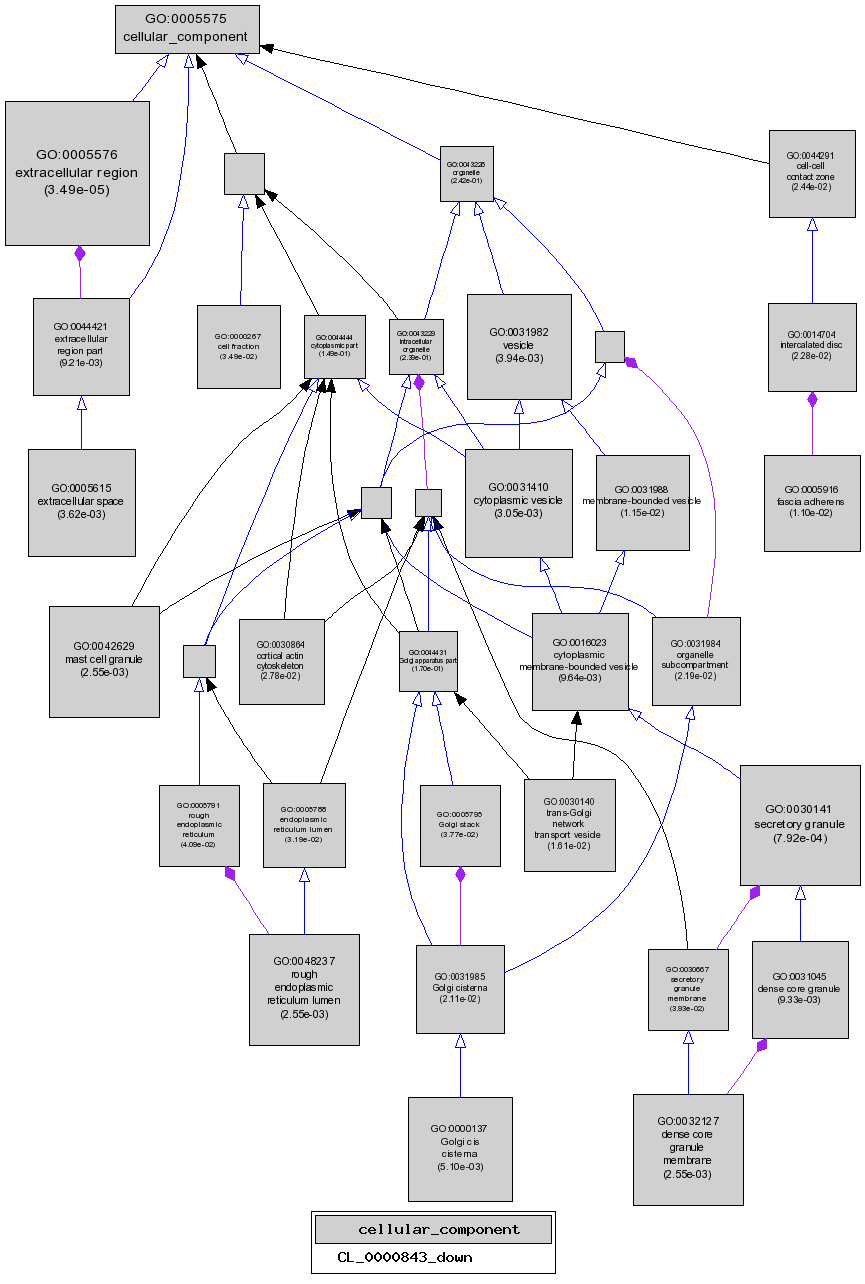

Supplement: Additional file 1 — OBAMS profiles for all mature B cells. Additional file 1 contains a zip archive of OBAMS profiles for all mature B cells, including for each cell type individual spreadsheets showing up and down regulated genes for that cell type relative to parental cell types, and VLAD (GO term enrichment) results for all mature B cells. [file 1471-2105-14-263-S1.zip › Additional File 1/follicular B cell/VLAD.CL_0000843_down/auxfiles/vlad.results.cellular_component.png]

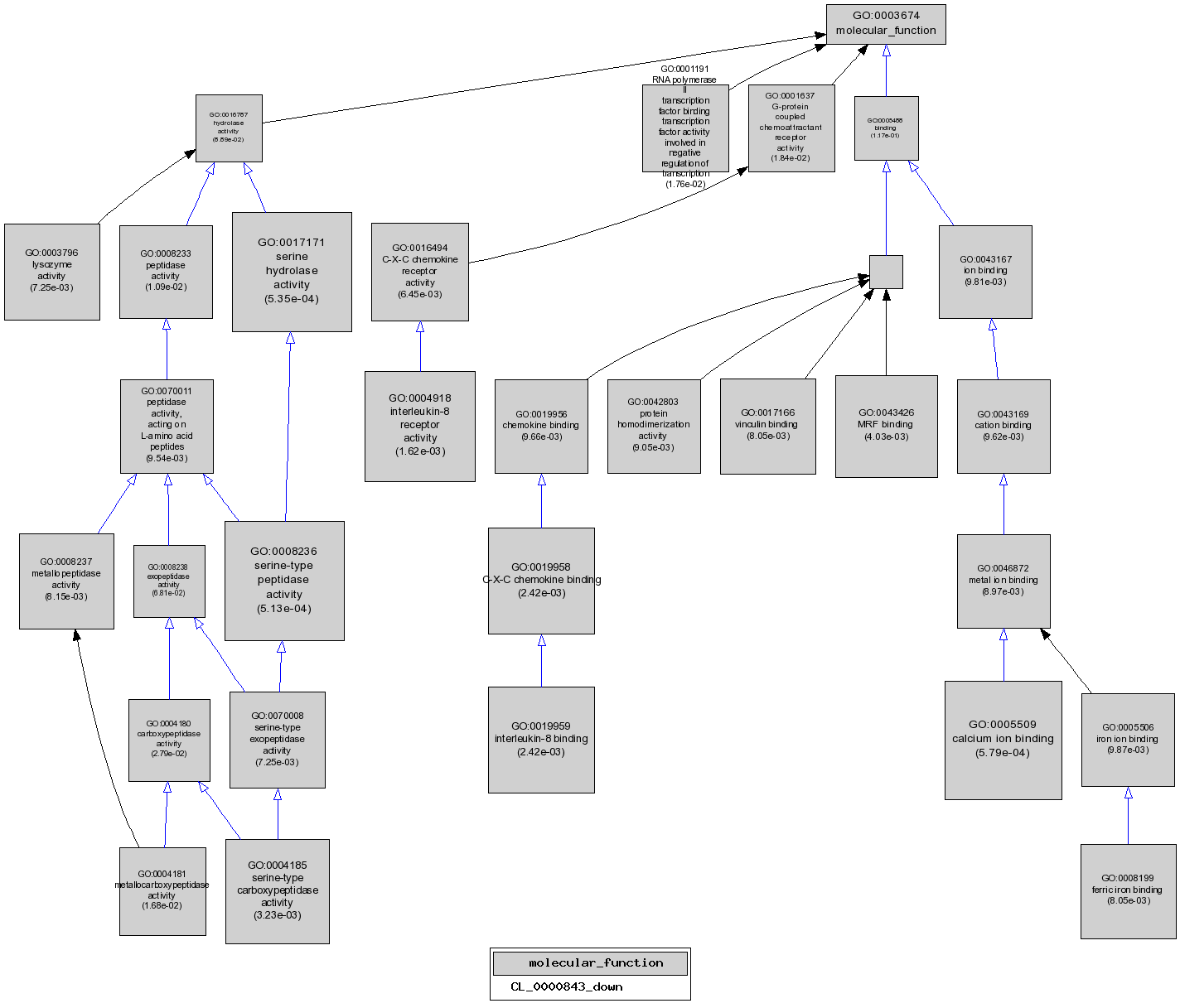

Supplement: Additional file 1 — OBAMS profiles for all mature B cells. Additional file 1 contains a zip archive of OBAMS profiles for all mature B cells, including for each cell type individual spreadsheets showing up and down regulated genes for that cell type relative to parental cell types, and VLAD (GO term enrichment) results for all mature B cells. [file 1471-2105-14-263-S1.zip › Additional File 1/follicular B cell/VLAD.CL_0000843_down/auxfiles/vlad.results.molecular_function.png]

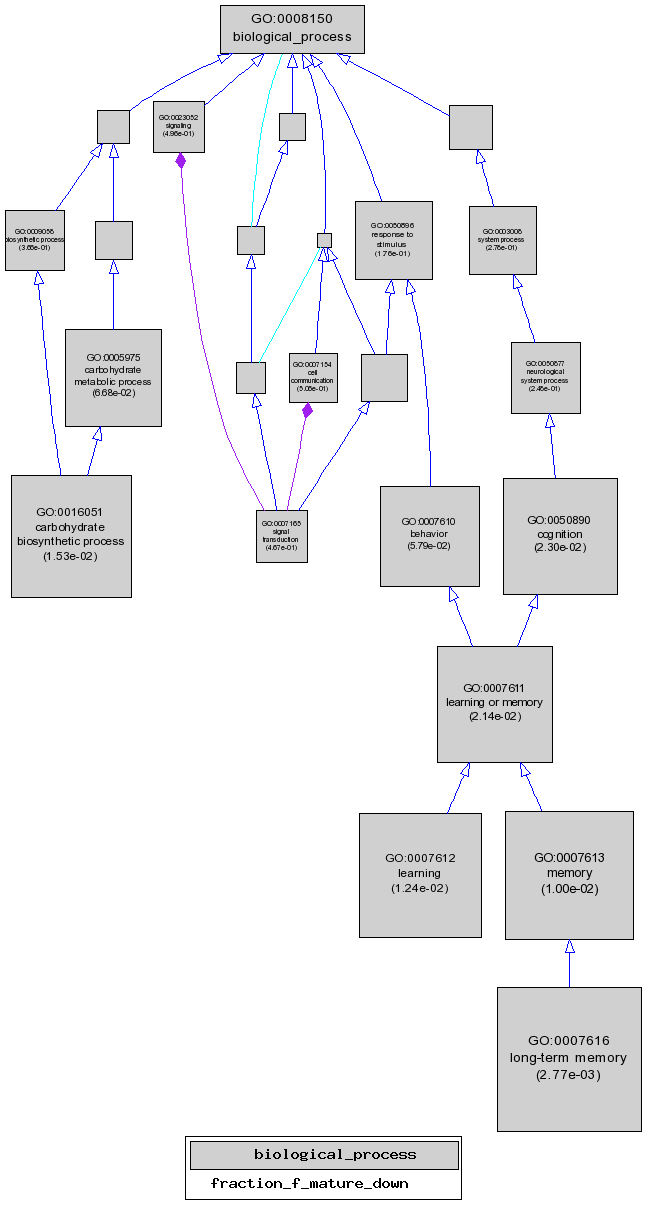

Supplement: Additional file 1 — OBAMS profiles for all mature B cells. Additional file 1 contains a zip archive of OBAMS profiles for all mature B cells, including for each cell type individual spreadsheets showing up and down regulated genes for that cell type relative to parental cell types, and VLAD (GO term enrichment) results for all mature B cells. [file 1471-2105-14-263-S1.zip › Additional File 1/Fraction F mature B cell/VLAD.fraction_F-down/auxfiles/vlad.results.biological_process.png]

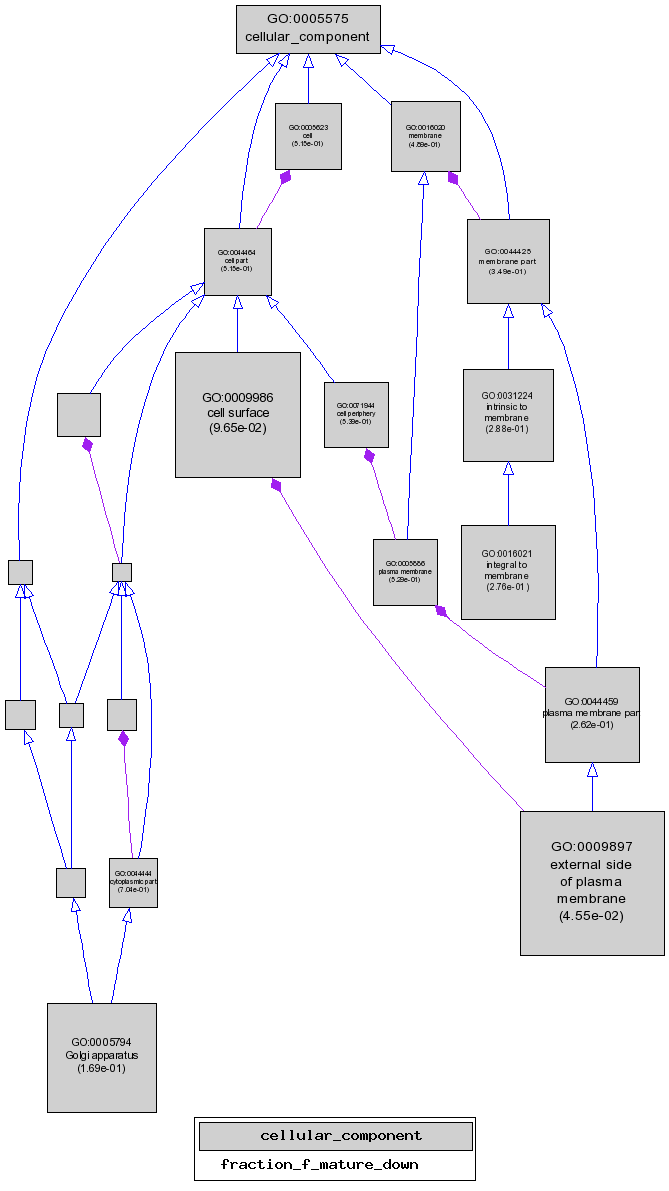

Supplement: Additional file 1 — OBAMS profiles for all mature B cells. Additional file 1 contains a zip archive of OBAMS profiles for all mature B cells, including for each cell type individual spreadsheets showing up and down regulated genes for that cell type relative to parental cell types, and VLAD (GO term enrichment) results for all mature B cells. [file 1471-2105-14-263-S1.zip › Additional File 1/Fraction F mature B cell/VLAD.fraction_F-down/auxfiles/vlad.results.cellular_component.png]

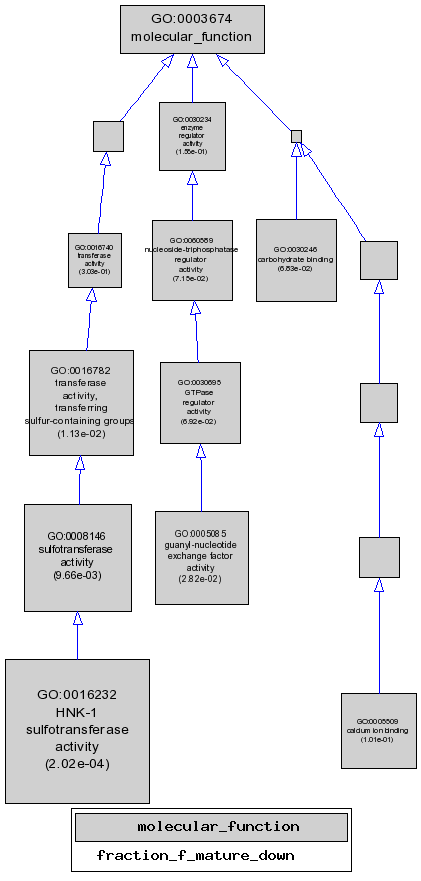

Supplement: Additional file 1 — OBAMS profiles for all mature B cells. Additional file 1 contains a zip archive of OBAMS profiles for all mature B cells, including for each cell type individual spreadsheets showing up and down regulated genes for that cell type relative to parental cell types, and VLAD (GO term enrichment) results for all mature B cells. [file 1471-2105-14-263-S1.zip › Additional File 1/Fraction F mature B cell/VLAD.fraction_F-down/auxfiles/vlad.results.molecular_function.png]

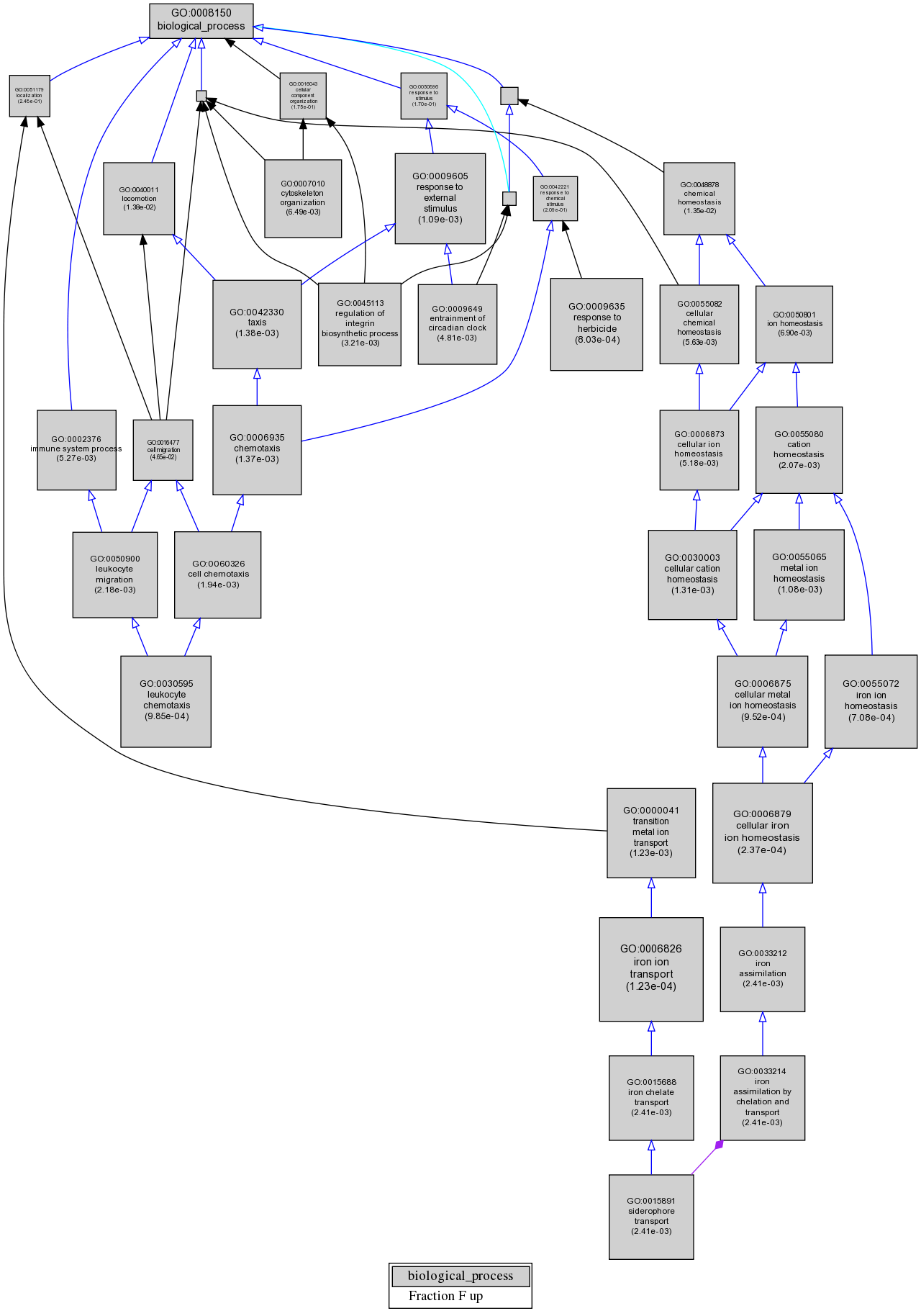

Supplement: Additional file 1 — OBAMS profiles for all mature B cells. Additional file 1 contains a zip archive of OBAMS profiles for all mature B cells, including for each cell type individual spreadsheets showing up and down regulated genes for that cell type relative to parental cell types, and VLAD (GO term enrichment) results for all mature B cells. [file 1471-2105-14-263-S1.zip › Additional File 1/Fraction F mature B cell/VLAD.fraction_F_up/auxfiles/vlad.results.biological_process.png]

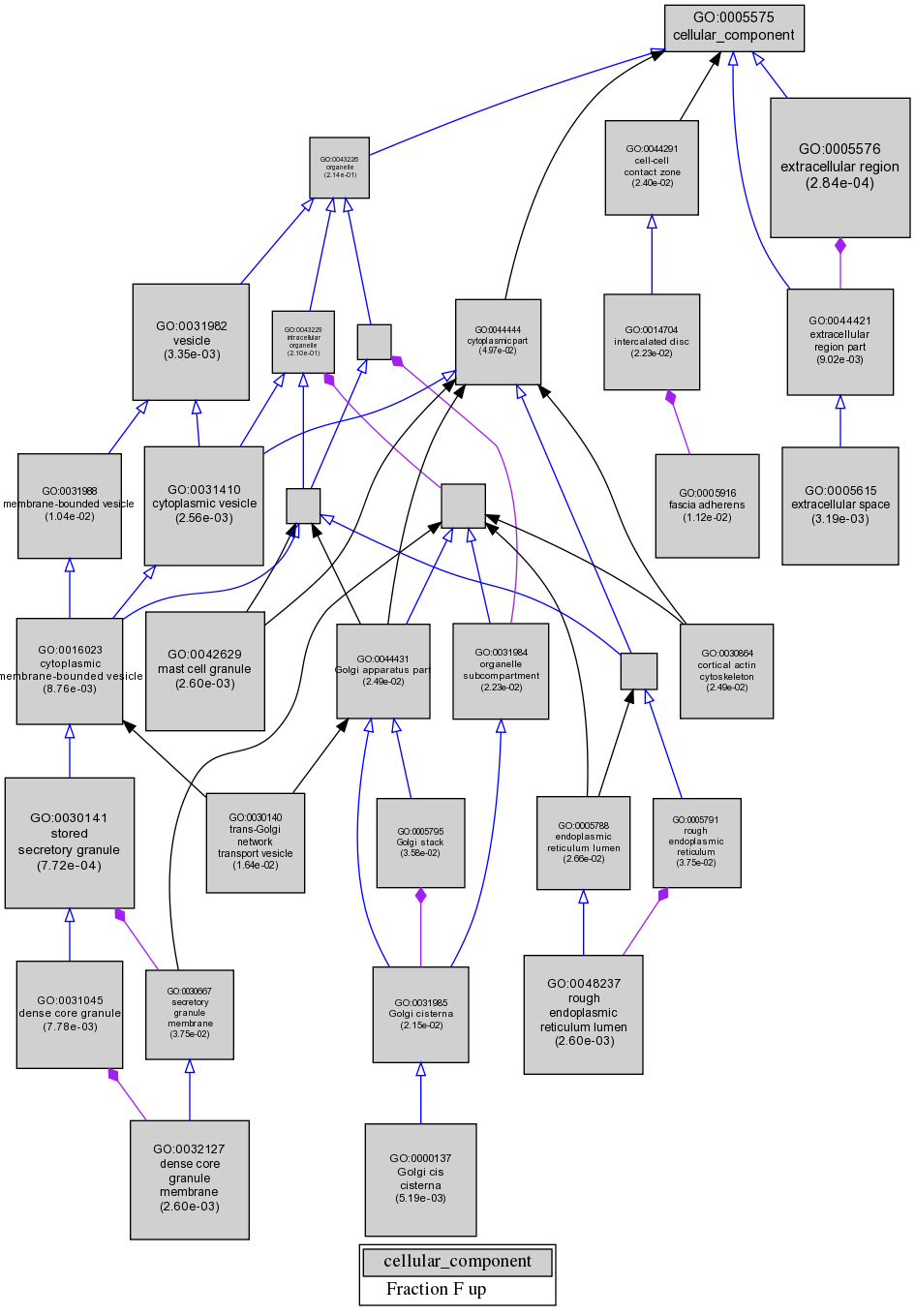

Supplement: Additional file 1 — OBAMS profiles for all mature B cells. Additional file 1 contains a zip archive of OBAMS profiles for all mature B cells, including for each cell type individual spreadsheets showing up and down regulated genes for that cell type relative to parental cell types, and VLAD (GO term enrichment) results for all mature B cells. [file 1471-2105-14-263-S1.zip › Additional File 1/Fraction F mature B cell/VLAD.fraction_F_up/auxfiles/vlad.results.cellular_component.png]

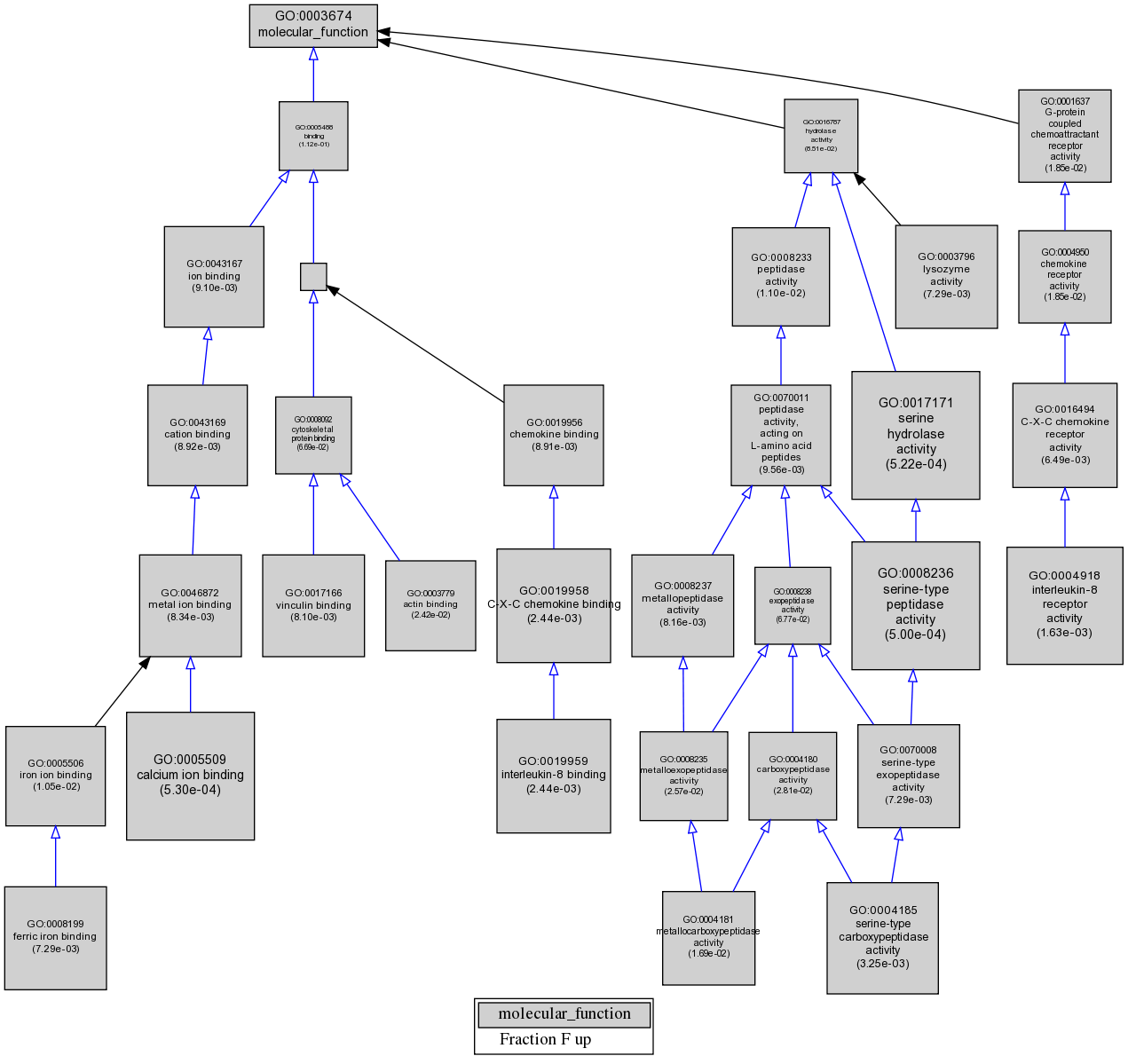

Supplement: Additional file 1 — OBAMS profiles for all mature B cells. Additional file 1 contains a zip archive of OBAMS profiles for all mature B cells, including for each cell type individual spreadsheets showing up and down regulated genes for that cell type relative to parental cell types, and VLAD (GO term enrichment) results for all mature B cells. [file 1471-2105-14-263-S1.zip › Additional File 1/Fraction F mature B cell/VLAD.fraction_F_up/auxfiles/vlad.results.molecular_function.png]

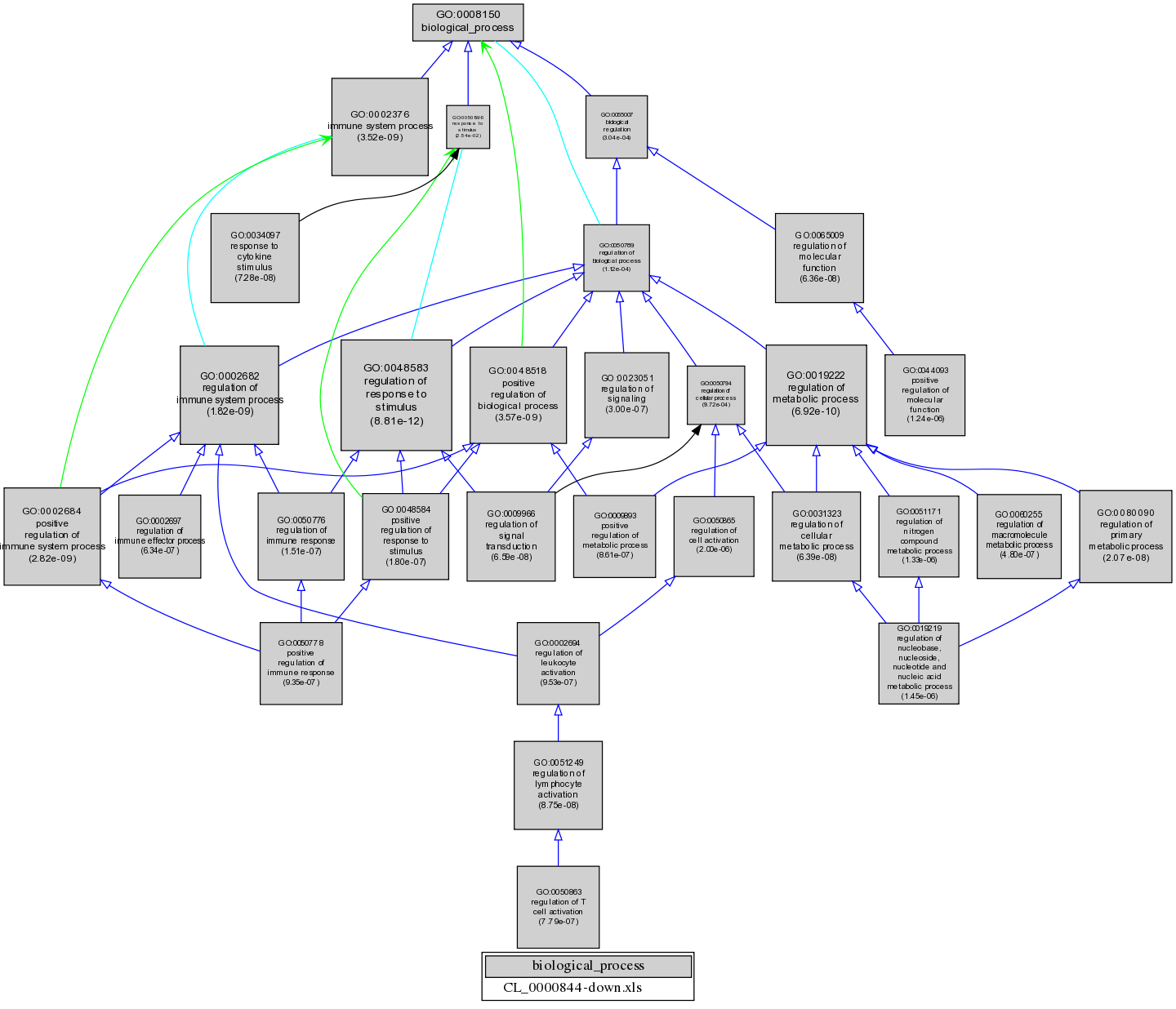

Supplement: Additional file 1 — OBAMS profiles for all mature B cells. Additional file 1 contains a zip archive of OBAMS profiles for all mature B cells, including for each cell type individual spreadsheets showing up and down regulated genes for that cell type relative to parental cell types, and VLAD (GO term enrichment) results for all mature B cells. [file 1471-2105-14-263-S1.zip › Additional File 1/GC B cell/VLAD.CL_0000844_down/auxfiles/vlad.results.biological_process.png]

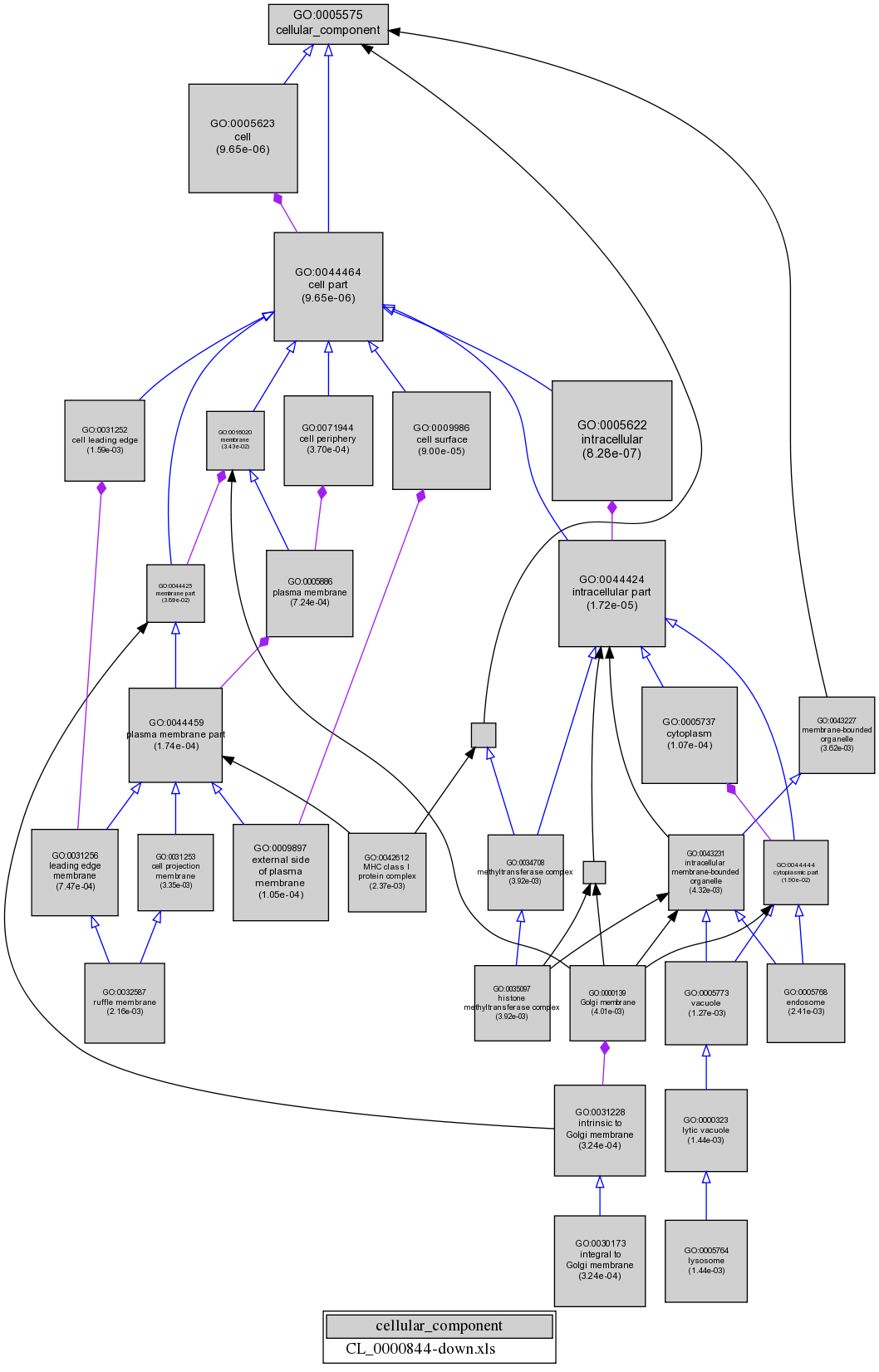

Supplement: Additional file 1 — OBAMS profiles for all mature B cells. Additional file 1 contains a zip archive of OBAMS profiles for all mature B cells, including for each cell type individual spreadsheets showing up and down regulated genes for that cell type relative to parental cell types, and VLAD (GO term enrichment) results for all mature B cells. [file 1471-2105-14-263-S1.zip › Additional File 1/GC B cell/VLAD.CL_0000844_down/auxfiles/vlad.results.cellular_component.png]

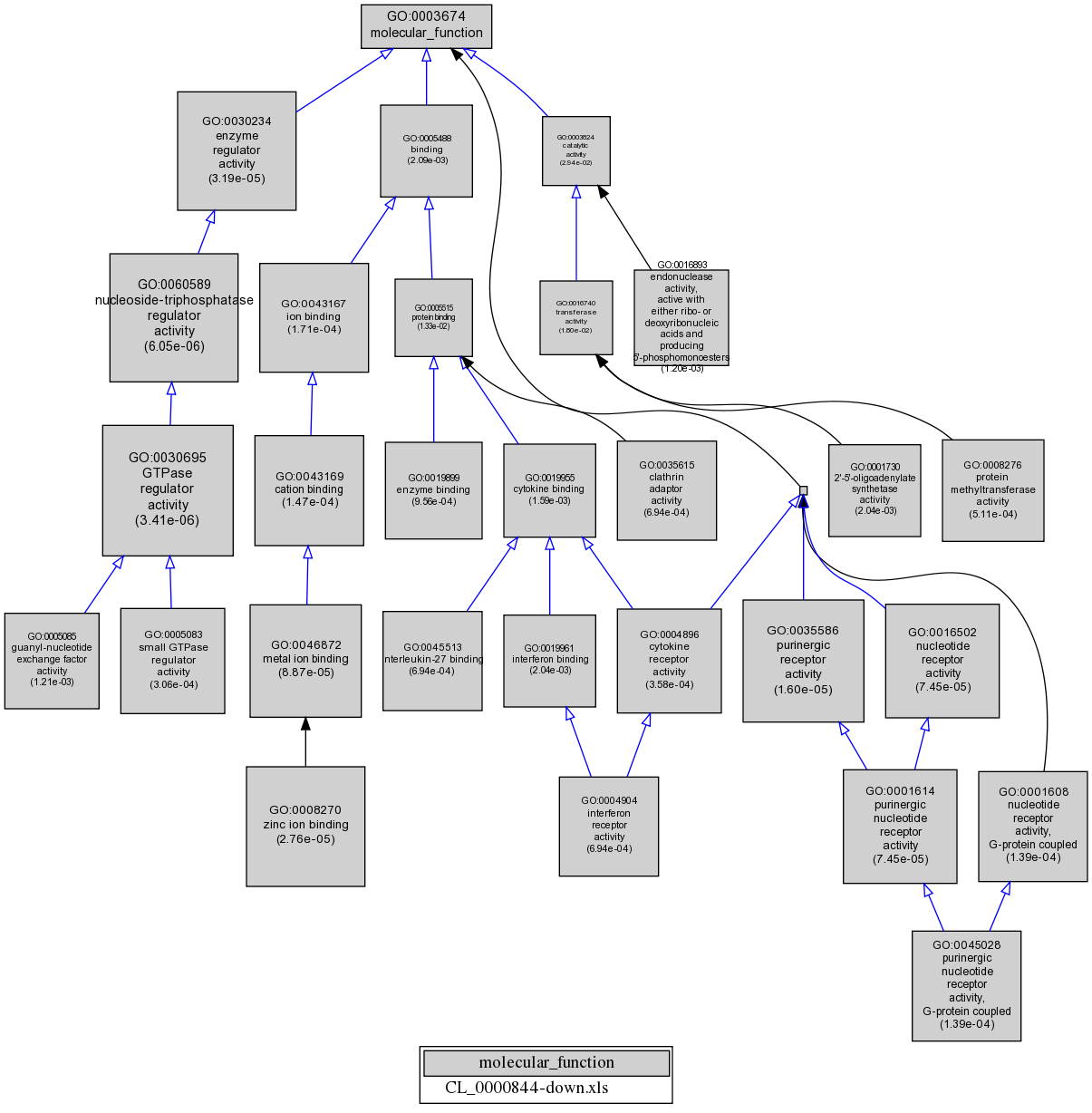

Supplement: Additional file 1 — OBAMS profiles for all mature B cells. Additional file 1 contains a zip archive of OBAMS profiles for all mature B cells, including for each cell type individual spreadsheets showing up and down regulated genes for that cell type relative to parental cell types, and VLAD (GO term enrichment) results for all mature B cells. [file 1471-2105-14-263-S1.zip › Additional File 1/GC B cell/VLAD.CL_0000844_down/auxfiles/vlad.results.molecular_function.png]
