# Supplementary material for: Ontology based molecular signatures for immune cell types via gene expression analysis
Source: BMC Bioinformatics. 2013 Aug 30;14:263. doi: 10.1186/1471-2105-14-263 (PMC3844401; doi:10.1186/1471-2105-14-263)
Supplement: Additional file 1 — OBAMS profiles for all mature B cells. Additional file 1 contains a zip archive of OBAMS profiles for all mature B cells, including for each cell type individual spreadsheets showing up and down regulated genes for that cell type relative to parental cell types, and VLAD (GO term enrichment) results for all mature B cells. [file 1471-2105-14-263-S1.zip › Additional File 1/GC B cell/VLAD.CL_0000844_up/results.html]

# CL\_0000844\_up

|  |  |
| --- | --- |
| Vlad version: | v1.5 |
| Date: | Mon Aug 29 17:26:42 2011 |
| Run time: | 56.06 sec |
| Ontology file: | gene\_ontology.obo |
| Ontology date: | Fri Aug 26 19:30:00 2011 |
| Annotation file: | gene\_association.mgi |
| Annotation date: | ??? |
| Analysis type: | enrichment |
| Excluded evidence codes: | ND |
| Number of query sets: | 1 |
| Query set 1: | CL\_0000844-up.xls (n=1160; 5442 not found) |
| Universe set: | default (everything) |
| Graph display: | Top 25 scoring terms and their ancestors. Interior nodes have been culled. |

**Jump to:** biological\_process | cellular\_component | molecular\_function | Unannotated id/symbols

### biological\_process (top)

  
  


### cellular\_component (top)

  
  


### molecular\_function (top)

  
  


### Unannotated IDs

**CL\_0000844-up.xls**  
0.0498592529399195
0.0542322550429934
0.0573541226770831
0.0659362354140193
0.0664792373401471
0.0694982103944193
0.0781327015182748
0.085635890037617
0.0861903685322887
0.088550414133224
0.0900220456488523
0.0907066073050303
0.091872986266801
0.0932459595585872
0.0937121892765489
0.0950112863294448
0.0970923929594799
0.0977344092894836
0.0978562943110073
0.100158541304149
0.100487985730368
0.100758290934717
0.103271688154505
0.103583455777321
0.103666763313723
0.103913891465207
0.105997751541257
0.106575137886707
0.107440921459701
0.108496834713664
0.108708582291572
0.108909119452831
0.109237755379015
0.112174380432741
0.112350778737866
0.112544940454078
0.113510821596495
0.114572253755819
0.114797297098756
0.114866994684994
0.115396266293601
0.115719260072358
0.116316886567698
0.11638909089274
0.117547590869848
0.117864471921149
0.118178423860988
0.118182575016109
0.119235601803227
0.119307240579014
0.12011183343741
0.123059592870378
0.123955566144029
0.124072554330816
0.124461616747546
0.124955139609091
0.125013495915424
0.127085075297961
0.127288468282809
0.127386904048337
0.13004563912736
0.130164960258383
0.131349563885444
0.131529316260982
0.131590193321678
0.13286478462434
0.134153931309775
0.134228372250418
0.134617002052675
0.134762383097327
0.135503156063883
0.135754641798537
0.137412630525021
0.137503552657906
0.139301706635023
0.139402031786152
0.14015556496295
0.141166602035104
0.143448548823178
0.143488139372109
0.144066135068195
0.144147594422966
0.144315459758879
0.144678655371387
0.144977333767304
0.14650416549264
0.146993159575965
0.147548646820925
0.148230205765189
0.148530717024585
0.148917529252552
0.149910946548994
0.15013806417285
0.150506736590093
0.151087120908348
0.151504922539106
0.151630720652139
0.151720442208377
0.152847800158566
0.15390283317123
0.154589942967802
0.154669253612151
0.155092666654077
0.156630049110108
0.157609832128405
0.158399697217587
0.158455818462211
0.158525019962129
0.158695242094458
0.158702770308165
0.158931430088256
0.159134869504699
0.159509981697193
0.160249141353518
0.160605038345928
0.161147338985975
0.161624064030469
0.162586145180068
0.162923475699573
0.163336571253845
0.163657010089476
0.163944394010784
0.164354313204214
0.165174530555745
0.165208342098845
0.165264624752789
0.166327121049149
0.166716644483647
0.16733942419997
0.167552467089588
0.167559995659175
0.1678964088019
0.168092953528655
0.168101359751632
0.16843028057136
0.168715497810931
0.168901778044247
0.169074062070178
0.169592578190777
0.169664615635465
0.169973599734699
0.170674950673132
0.173781536728403
0.17540109601259
0.175868986375918
0.175903959747768
0.17652273833695
0.17781168771766
0.178445853453803
0.179343886425671
0.1804803447043
0.180726540478259
0.181042951778933
0.181798417903161
0.181999131870806
0.182462646600663
0.182500845533977
0.183574721554259
0.184510132225606
0.18453822285306
0.184944701291306
0.185111720984486
0.18622974443288
0.186936464292191
0.187158496814177
0.187832314860744
0.187981866768865
0.188319363277044
0.188792359009418
0.189608814111454
0.190138322051043
0.190944900469275
0.191817434092991
0.192409949347215
0.192775805352069
0.193003155843885
0.193089151257649
0.193533354082944
0.193980075422434
0.194101662659077
0.195808111663691
0.195910204780978
0.196344850649227
0.196422583898178
0.196510250551442
0.196903105446863
0.196975029032618
0.197052067772569
0.197053250219955
0.197955639979255
0.199174550130189
0.199650911703178
0.199798348267063
0.199952195487412
0.2004943374475
0.200550872888686
0.200815075862544
0.201041607978493
0.201210286083219
0.201518979689597
0.202523633702811
0.203123457432542
0.203683293682538
0.203687454872524
0.204258375467153
0.204285708363356
0.204461184765627
0.206078926866024
0.20674774051734
0.207097305771305
0.207205963934187
0.20766001962037
0.207805486105281
0.207899176329929
0.207955423401563
0.208182683281434
0.208950095790478
0.209591434962726
0.210593325445636
0.210896630375875
0.210911428899932
0.211088725182646
0.211119891931829
0.211180953357033
0.211831640816328
0.212068993901045
0.212303599468272
0.212736348633805
0.214601406752215
0.214688297110852
0.215376326198611
0.215505972353791
0.215898623419993
0.216019882681122
0.216139050400715
0.216318732877909
0.216393842696379
0.216620151285401
0.216917939783876
0.21858500743285
0.219069248548157
0.219481669080517
0.220021920946016
0.220044167402191
0.22023823264659
0.220677026164975
0.221485792292427
0.221657496236479
0.222793129600756
0.223203412539948
0.223746501339569
0.224051343627469
0.224224377543446
0.225720942614211
0.225958262041177
0.22648337784127
0.22684970204291
0.226926476097762
0.227104073292749
0.227117437973009
0.227994347998822
0.228449927433198
0.228497662189784
0.228796221540048
0.229063855802353
0.22971035740738
0.229740637098675
0.23012925930346
0.230305452032878
0.230819730904052
0.232147129985954
0.233832781646528
0.234053263402269
0.234110420753385
0.235154462684795
0.235164516448479
0.235810303090324
0.236432797068459
0.236752010096741
0.236979384633165
0.237521636079769
0.238059274423033
0.239507057242844
0.239907252244566
0.239911799419616
0.240138384938512
0.240784635947453
0.241033873920177
0.241156336649189
0.241693001372003
0.241847696348528
0.242101278739161
0.2422582673508
0.242392802500019
0.242521267243805
0.242716898351524
0.24283113998538
0.243104371456714
0.243337423322405
0.243538795385272
0.243961196025434
0.24406442911664
0.244065129807927
0.244162536872022
0.244506647836564
0.244741763870377
0.245494002362671
0.245547586182793
0.245784403806888
0.245785447176332
0.245820875434938
0.245829119870352
0.246349507902148
0.248342577946359
0.248493331986535
0.248729939031789
0.248789260982425
0.248978341492032
0.249317113340705
0.249388385967981
0.250215189066957
0.251233908265981
0.252047889514353
0.252358741248333
0.252407910169025
0.252736688870146
0.253084833717811
0.253316577630478
0.253761344102144
0.254314545349067
0.254634805270283
0.254830693897022
0.254863334095872
0.255168919263085
0.255187693088019
0.255541361189244
0.255881037150512
0.256107034963028
0.256676239543071
0.258036579722095
0.258309090421413
0.258410142959699
0.258561497819843
0.258821085662645
0.261432043741783
0.261748271487265
0.261851626530686
0.261869238319723
0.261971476376127
0.262591069121292
0.262963241113979
0.263327545384123
0.263786217465494
0.264012553671865
0.264590171945775
0.265107347782011
0.265132972020048
0.265334308477701
0.265857958866276
0.265889419257217
0.265905076675573
0.266081963170392
0.266379271264533
0.266462978039898
0.266523928976489
0.266855307585698
0.266914579436409
0.267607943060501
0.267766497625278
0.267996228016136
0.268237188282374
0.268240882802861
0.268255551996204
0.268306184945564
0.268847967230154
0.26899329581566
0.268997117949532
0.269576974983095
0.26982066381547
0.270434475493358
0.270443008287923
0.270645385238044
0.27122424547832
0.27141111927084
0.271607927445807
0.272504888131822
0.273383794235122
0.274130711451745
0.274427740032657
0.274757611200544
0.274857103986278
0.275349296974506
0.275369781185736
0.275553118300131
0.277525424659361
0.277984704834086
0.278038256411278
0.278216792557426
0.278346053665202
0.279029586234494
0.279055111930134
0.279351323290114
0.279712070377322
0.279855264744923
0.279942919994015
0.280782599708192
0.281709110286508
0.282354109410551
0.282963217061615
0.283206451955118
0.284033640602158
0.28435260457286
0.284575274537357
0.284863338897152
0.285482432199796
0.286230464782032
0.286463083322225
0.28652181006292
0.286593720810183
0.287180090512782
0.287463147366907
0.288414107537348
0.29027671876143
0.290670949185163
0.290835674312369
0.290851481476416
0.291233254785979
0.291622794420621
0.292183996245843
0.293136513488055
0.293353851595894
0.294369454815871
0.29487909383731
0.295024505086928
0.295227025195604
0.295237725144261
0.295942288466931
0.296154483984614
0.296657612134939
0.296801793034257
0.296849918085073
0.297774529153942
0.297983656670036
0.299327747850383
0.299354965443221
0.299556109882322
0.299647970787377
0.299737601944953
0.299836328148247
0.300473898983545
0.301065470815416
0.301150774192154
0.301177315654957
0.301677425175419
0.301752469932005
0.301922090501496
0.30276112242951
0.302821832355088
0.303315708078379
0.303734436702807
0.304178318185241
0.304711957603885
0.30475761639925
0.305640982230661
0.305976031101748
0.306083078387984
0.306556798234642
0.30682073182034
0.307631766242842
0.308145191974123
0.308469021277116
0.308691995708485
0.308909785670228
0.309514261594145
0.30970000489175
0.310248855571925
0.310706791539509
0.3108725450026
0.311168973200341
0.311442780197326
0.311615726666523
0.313158545109447
0.313925781090977
0.314151894943377
0.314191689907969
0.314224220138454
0.314706672572433
0.315121122196471
0.316159515027758
0.316773533348867
0.31739947758768
0.317488222647167
0.317626496222348
0.317660967741996
0.317749325747808
0.318244027295845
0.31917643271807
0.319816543212143
0.320409610130537
0.320509596740993
0.321389907964344
0.322055229516962
0.322284809469112
0.322771383512134
0.323972760522489
0.324153500294075
0.324969346155193
0.325529518688279
0.325674415113623
0.326550241519619
0.32799911268602
0.328211165494217
0.32851732481814
0.328684501468725
0.328902093707796
0.329212333807641
0.329268627199364
0.329441689713471
0.329797517224918
0.329864879484949
0.33138143458545
0.331602904252183
0.332430348080291
0.332890321027222
0.334108289144843
0.334365817146368
0.334544756382849
0.334582251482138
0.334798401762255
0.336071924261159
0.336905497349042
0.337399514601854
0.337864692169024
0.338315613622483
0.33836785224193
0.338904922110939
0.339177528458611
0.339376672706781
0.33969140230153
0.340199156832938
0.340458114143144
0.340774552843455
0.341000902522496
0.342435602776663
0.342778147988959
0.342820673360551
0.345094870568658
0.345328130496379
0.346120589137442
0.346391402339548
0.346406156511815
0.346616221219994
0.347400285432862
0.347518129143784
0.349482266773682
0.350138887021944
0.350160613235523
0.351428918882142
0.351524119952139
0.35230476965515
0.353020660899854
0.353128618453958
0.353291932407783
0.353974667059599
0.354249297804274
0.354253806334006
0.35433342865013
0.354651332805753
0.354673160381426
0.35481747377124
0.354895493813877
0.355253065581156
0.35526349046962
0.355560742760646
0.35642705715099
0.356773701841414
0.360450308548353
0.360584671884142
0.360590081471265
0.360602253402582
0.361198149605721
0.361351599532596
0.361524476892468
0.361683483881935
0.363100942301485
0.363207623847542
0.363543424788506
0.363925912651401
0.364490296101188
0.364525376022733
0.365076152747419
0.365565774154064
0.36587155953516
0.366172128711342
0.366920211340221
0.367307566626703
0.368123714729964
0.368716599609313
0.370382057170176
0.37085209243134
0.371743491044638
0.372049935144981
0.373129266127683
0.373490218761302
0.373538424665151
0.37388224846947
0.373976075137559
0.375334287202195
0.375603416459078
0.37650926079905
0.37726336193087
0.378509550596206
0.379058396924401
0.379408796670635
0.379409851323282
0.379844759608447
0.380117631612154
0.380261742790805
0.380872136941898
0.381918104010599
0.382016524531543
0.382761799806979
0.383039638785219
0.384893770468568
0.385234607540608
0.385587115309121
0.385820296824372
0.385825802851146
0.385854405922348
0.386815433086717
0.3869616716376
0.388642183685738
0.389357811089907
0.390131744133049
0.390550493381941
0.390731358452008
0.391301989602465
0.391385856277677
0.391550614266083
0.393076264464994
0.394316230914842
0.394553387148153
0.394707359976383
0.39498262271985
0.395209181271371
0.395471524277993
0.395765534690183
0.396003679388284
0.396194187604619
0.396197884850745
0.397157608520722
0.397305876775256
0.397309579797601
0.397488531484414
0.398772452414946
0.399291579097958
0.399795843746772
0.401535120007831
0.40262295077193
0.403200435024706
0.403584869533228
0.403655942597001
0.404331256457572
0.404992908368957
0.406329293393123
0.406807562577398
0.4079555368342
0.408094921576065
0.408294389673032
0.408305171667051
0.408803117081235
0.409508172903872
0.4098403829104
0.409872427694554
0.410154775913372
0.41195409645598
0.413214104112156
0.414283638128121
0.414789736923523
0.415452926615616
0.41555516233769
0.415683315102906
0.416312692037332
0.4163829048015
0.417203937196325
0.41744689957075
0.418112663112359
0.418340856629998
0.418535261942874
0.420162390518498
0.420300534368491
0.42248646208252
0.423079773472371
0.424404159459124
0.424871206631857
0.425273425232074
0.428319587293108
0.429333590179914
0.430949175389145
0.431236386086501
0.431826857915221
0.432629319464761
0.43281119922581
0.43316587977888
0.433579711613808
0.435374526411085
0.435420336793667
0.436473661047627
0.436536308620809
0.437304257870193
0.437863564747292
0.437920751184034
0.438644469625393
0.440096297218878
0.440437125428522
0.440607388478201
0.440902207582101
0.44286798991601
0.444106308786996
0.44477105612963
0.444861912001955
0.446975555249449
0.447941983486546
0.449103835900727
0.449908049854748
0.450488813894413
0.451450997860819
0.452896831837824
0.453024620474091
0.453382818534348
0.454821969219265
0.455180626283558
0.456015427198902
0.45863053703713
0.459399386844642
0.459414431286143
0.460067932728798
0.460909724379097
0.461038666651601
0.461139667284238
0.461624329250836
0.462402632527007
0.462852087818475
0.463398866335277
0.463973750548374
0.464281838791289
0.464528333055909
0.464550251182813
0.468041919472551
0.470495912849106
0.471693862992926
0.471796845807249
0.47187944953874
0.472380393526976
0.473277310193677
0.473722295988989
0.473976620451874
0.47538962936278
0.4756345395467
0.476545456171558
0.47746467112213
0.478545949235931
0.478749039111757
0.479171055454061
0.479426547730744
0.480953775738241
0.481301934067182
0.481506751561511
0.482500185539377
0.482561087546444
0.48270601074425
0.483041958528595
0.485193319647418
0.485384043891923
0.485853521296781
0.486431927879614
0.487617824935518
0.487752401873967
0.488565965042234
0.489457611077479
0.490153452550521
0.491060185200159
0.491371729955743
0.491615030809626
0.492329735214643
0.492577303236623
0.493198530513086
0.494154658677507
0.495186339192997
0.497144651437414
0.498330417859463
0.498739011497955
0.500501065193275
0.50111011396999
0.502547507869691
0.502979782672041
0.503484337051417
0.504177924545698
0.504855096910053
0.504873828267992
0.505621559305251
0.507185444892732
0.508038874104319
0.508478484339319
0.509535202464896
0.510794892564889
0.511286427883481
0.511957606587706
0.512500990421481
0.512915965473189
0.513749711477928
0.514489727014443
0.514681350833417
0.515005215532294
0.515054205011369
0.516319158175773
0.517177683937448
0.517721205428832
0.519222003445582
0.520409880007805
0.52188582459434
0.523929665309322
0.524767706435181
0.524975627844814
0.525896302840557
0.526400219253742
0.527550854660188
0.530631044011411
0.530636041533516
0.530907223109411
0.532621344846294
0.538250022646396
0.53901215601392
0.539031550315597
0.539825497865553
0.541639529511105
0.541772705403447
0.541918774211815
0.543799819316768
0.543808373633947
0.547499228732841
0.549399121076015
0.549992518829693
0.550074918041296
0.552866597938163
0.553971032591366
0.554050713798633
0.554599473822564
0.554878823924327
0.556100964678447
0.557074120087697
0.558518294958294
0.560194214680306
0.56036399485022
0.561439482452901
0.564296648851704
0.564997848088749
0.565262435543909
0.567175150838572
0.567447441332981
0.57043640270876
0.575007335276272
0.575805099326863
0.575880043854648
0.575880348357861
0.575992758506537
0.576236295024739
0.576792346343675
0.576973149550071
0.57720501443335
0.577784201255122
0.578234306946236
0.5790367588164
0.580885462320018
0.580978867744492
0.581055284329198
0.581062737518676
0.581275070365644
0.58192131556594
0.581934408460916
0.582060733129318
0.58218703173842
0.582397103129556
0.583823423614702
0.584533694199945
0.587397617183121
0.587884817015017
0.588226455877422
0.589087027132897
0.59237472963524
0.592674720572004
0.593369892192092
0.595431020128545
0.597123370484064
0.597203478612502
0.599019874371137
0.599453612626252
0.59962021973955
0.599711559767802
0.599924744345226
0.60028309263922
0.604316627266179
0.60837117744363
0.608382056420288
0.608813684837139
0.610526898978976
0.611068893358643
0.613217204599425
0.61326413083309
0.613303242241193
0.615352037299031
0.616397103357877
0.616633225142749
0.617120622973068
0.620383742149514
0.620921458098158
0.620997881923934
0.62160365131795
0.623727221847174
0.625239291160914
0.625566115538966
0.629829952573279
0.632157071380293
0.633364577113771
0.634136996153052
0.637326585432313
0.639785929136872
0.64186063934481
0.646373232113881
0.648053481262937
0.649250828312254
0.649272695302059
0.652563447494219
0.653321057191423
0.653846847515454
0.655534629616611
0.656807231019231
0.659545980910804
0.659742032770652
0.66064578964657
0.660793727189773
0.664385372411667
0.665683597067029
0.666309338589025
0.666944456271242
0.668690326898439
0.668915301291251
0.668928640251676
0.669516821112509
0.670241203680008
0.676667802410963
0.679461331332898
0.683765412541108
0.684461900520221
0.685202128700969
0.685724926347822
0.685757858179885
0.685963904359805
0.688358564136937
0.688392061788266
0.688925020654994
0.689376412672925
0.689866056587934
0.692078452707399
0.692210267219485
0.693128050995626
0.693211711904867
0.693816174693457
0.694624587057369
0.696428308240868
0.69685267216984
0.698087353133614
0.698795569205877
0.699074567279911
0.700352354197272
0.705618603756973
0.710235599506295
0.711176847819578
0.712449148644485
0.712826662934234
0.713160415905958
0.715425359569069
0.718763369704269
0.719088180564401
0.719101993587075
0.719773329292155
0.722069955228837
0.722266267942798
0.722302320304528
0.723262378644253
0.723603908438621
0.724630540832575
0.725823666968924
0.726711144604406
0.727792352212221
0.728608399775402
0.728846815299848
0.730143389009772
0.733104106400233
0.734656040285758
0.738254240797185
0.738329437026612
0.738721105543711
0.740936972386028
0.743118761640419
0.747778102488374
0.749393163710113
0.749901430526857
0.750689345653894
0.752198510706134
0.754713614031472
0.756235763095877
0.757646773402721
0.759610141107071
0.759965305122176
0.760000343247781
0.761174070150802
0.770111750555406
0.77180592446639
0.772918344664057
0.772931814251192
0.775009208700837
0.775168432498197
0.776414779454799
0.77670242514794
0.777057701876921
0.779365290011113
0.780254942663486
0.781161651037983
0.781489538245312
0.786012769421435
0.788536123454284
0.789137526894154
0.78918537212185
0.789257044318548
0.79020720877051
0.791553932594139
0.794856354815677
0.795119164060642
0.796705402333505
0.797141630194143
0.797212588690148
0.797345155791076
0.797981999256886
0.798398402934295
0.805272541884358
0.805390839101328
0.806552663514514
0.808034259619855
0.813356901716311
0.81348805165544
0.814615145172586
0.818333592436417
0.823663173315472
0.834972596156424
0.83681964697381
0.838991361863921
0.839697747731111
0.845180596289828
0.8457707069645
0.847302899676196
0.862935069879138
0.864358155219046
0.866107317669082
0.866158749133015
0.866703614830492
0.872352090456991
0.872833392612457
0.873458596182913
0.876651456777148
0.877518676973513
0.884103063003265
0.884566377673694
0.88598087807604
0.887583715518531
0.894335844289337
0.896664753104953
0.898633348219224
0.9027958236676
0.908300191357005
0.909527376275028
0.916160347829737
0.918024624440256
0.920136278605392
0.924362703764145
0.924599892636852
0.927296015058961
0.928469630051838
0.929088821993378
0.929378014857462
0.932144068363485
0.932675565726309
0.933078265371067
0.934928915102672
0.939393214520945
0.948421761566425
0.950648288949391
0.953855089325797
0.958602239433193
0.960241334052688
0.96618962783541
0.966940299932077
0.96846561451007
0.974605402642917
0.974703757139668
0.981694273194505
0.982194315454749
0.990825332640393
1.00517068675356
1.00872333407486
1.00952229971243
1.01446917467937
1.01623831333713
1.01857307451427
1.0204256426187
1.02276637935485
1.02782924612813
1.0329314485776
1.03923518630046
1.04285130047824
1.04759272830046
1.04858043217361
1.05306003375771
1.05583484640197
1.05651713077953
1.0584659640968
1.06118935009981
1.0612092794723
1.06670482420838
1.06683076031366
1.07058435581055
1.0767554275693
1.0800668542567
1.08186351894924
1.08238371891079
1.08345759026952
1.09194116722002
1.09263972993926
1.11683700631394
1.12071139140631
1.12182426723698
1.13188619093285
1.13406882635812
1.1403016432824
1.1459500549341
1.1525707220058
1.15938770698361
1.15954844922141
1.1617148994718
1.16283761071645
1.17215944297323
1.17318253058046
1.176170967453
1.17988801591313
1.18068031338785
1.18111349486411
1.1866536699472
1.18932941254435
1.19034668462826
1.20431121524937
1.20845406901248
1.22365092195181
1.22936804535897
1.23237054662535
1.23629138856466
1.23726774566109
1.23845754557681
1.23969557126017
1.23993215285583
1.24269899179926
1.24558661253877
1.25681842295756
1.27079817292752
1.27698592910358
1.28483656944067
1.28971798127
1.29503338229881
1.30061603828902
1.30084549389725
1.30936023097757
1.31148297912052
1.31376396945776
1.31641137474588
1.34376795714154
1.34730858238906
1.35667697975372
1.35956025491823
1.3622641928941
1.3651440226138
1.36591076743124
1.37326491365726
1.39393284853142
1.39540092992064
1.403890709939
1.41043305806847
1.42236913996085
1.42320028422156
1.42390000647412
1.42910697243054
1.43596778515989
1.43758252997297
1.4485447618477
1.47330272248924
1.48078296180247
1.48764981032554
1.49478833709035
1.4954010105917
1.49933149213532
1.50614891092695
1.54455932163192
1.54496994904572
1.55179244218866
1.55207292065689
1.55329576012327
1.55420851232932
1.55436917989269
1.5613187772684
1.56520408849021
1.57157285104139
1.57608167077538
1.57762191989722
1.59242977201754
1.59660127246314
1.59821816847033
1.60006315505679
1.60124100072997
1.60206599868768
1.60469617595761
1.6059011242609
1.61128156826508
1.61395110548274
1.61775646564981
1.63355586183623
1.63487113273844
1.63870482963219
1.63883963788354
1.64732776633404
1.65087373240136
1.65357524033318
1.65442322070164
1.66440476413846
1.66443356836342
1.66725151614259
1.66819113894956
1.67038859344591
1.67184534838682
1.67389117900322
1.67440063217412
1.67466467797722
1.67492760749042
1.67670523558662
1.67811710273261
1.6783327258633
1.68057159760843
1.68105860402608
1.68602209139238
1.68643117188672
1.68689823382904
1.68839096562437
1.68842998119117
1.69304745303028
1.6950302225252
1.69673679838432
1.69752936074983
1.70052080089736
1.70160719445304
1.7033341121692
1.70353330524664
1.70711306000777
1.71824543834696
1.71906462556969
1.72059805288226
1.72119610185971
1.72164438127861
1.72231893783194
1.72283894279755
1.73049957756654
1.73396434360954
1.73554425361941
1.73730360504502
1.74054430043842
1.74371905159852
1.74418468913539
1.74454356294358
1.74471594977279
1.74687094826053
1.74757226814686
1.74858322232331
1.74953924743286
1.75022828167252
1.75027029151701
1.75115463269711
1.75220046424313
1.75229142940989
1.75452894602003
1.75484162802165
1.75527894739877
1.75649026640708
1.75833106220327
1.7616447785062
1.76190054491033
1.76279724718062
1.76345060531122
1.76443384353198
1.76647935755075
1.76663457323015
1.76730177496499
1.76968290190731
1.76972886953746
1.7716081942356
1.77261897611275
1.77814831377804
1.7784204488655
1.7801135390171
1.78069642339929
1.78164641472058
1.78213615200963
1.78402772603613
1.78407962386245
1.78411563671782
1.78620460026832
1.78646336173374
1.7868820141361
1.78993541292705
1.79007902336805
1.7911736800783
1.79303230814309
1.79350836927016
1.79356053980055
1.79384194884019
1.79387032801223
1.79696665965541
1.79845858866255
1.79919236723537
1.79933667629477
1.80129852130223
1.80145237797142
1.80267720647301
1.80384330723737
1.80387376522941
1.80470584360251
1.80582618248693
1.80907196574293
1.8100313082968
1.81299342032024
1.81429677945297
1.8161352076993
1.81642575544268
1.81761728668721
1.81872770195804
1.81881573020254
1.81899148044529
1.81953758175911
1.82045739582188
1.82343098865744
1.82429973959817
1.8252713871775
1.82588548194494
1.82600048779551
1.82678696613754
1.82704998416382
1.82720559395194
1.82780177945801
1.82811977211095
1.82887436513035
1.82898782413143
1.83137585369982
1.8349668105139
1.83549212267881
1.83578114696732
1.83610341714178
1.83726948997326
1.83810188690778
1.83838585756659
1.83997494432782
1.84046883366499
1.84135379190265
1.84171397198452
1.84193538556571
1.84228186180238
1.84260054078027
1.84705606655225
1.84850162399534
1.85288572915052
1.853400313749
1.8551461051907
1.85545189532814
1.85600177611575
1.85830994294563
1.86101161789502
1.86103433671884
1.86122397828995
1.86124488898625
1.86199651602636
1.86293879395248
1.86354595850609
1.86532655351848
1.86703636603391
1.86760495685943
1.87097384874793
1.87178153243006
1.8720540170952
1.87247837485761
1.87265817545415
1.87321269375422
1.87350005867466
1.87393369929043
1.87429416004239
1.8750424353729
1.87519446033737
1.87669761896987
1.87791825672772
1.87849841896617
1.88093001365922
1.88299722620705
1.88355121144312
1.88483594235842
1.88503112711053
1.88805417962749
1.888811800218
1.89033989041011
1.89236144228709
1.89266910485249
1.89282837251167
1.89305973143253
1.8941962052929
1.89506175311162
1.8955072623393
1.89583104613878
1.89657482361015
1.89764524003951
1.89870526851819
1.8990030444338
1.90016997504171
1.90036674564799
1.9007099634705
1.90122117396664
1.90632159408804
1.90661097175194
1.90747411982896
1.90884654830921
1.90955298706749
1.91001090719997
1.91128643936331
1.9120758562282
1.91450778409009
1.91543207200985
1.91672828080771
1.91886437425829
1.92171864597722
1.921812159475
1.92239593657918
1.92279667138369
1.92305542656081
1.92323778936806
1.92461066442347
1.92490135245266
1.92499554040618
1.92529383195937
1.92675313217257
1.9280622260409
1.9287829975759
1.9306824105277
1.93084095200613
1.93114212223145
1.93131574038323
1.93136347457906
1.9358218701297
1.93607343000361
1.93678437105081
1.93998284391549
1.94281484673117
1.94295385902135
1.94451372923265
1.94522027239045
1.94629397176852
1.94732535003891
1.947829595268
1.94828784454568
1.94868933085753
1.94913774977895
1.94968717375535
1.94998051064654
1.9500556295267
1.95233645455981
1.95235664907188
1.95361469239224
1.95578079648856
1.95816556277169
1.95947254121366
1.96105171096071
1.96137818157371
1.96406169861184
1.96413132148466
1.96433104098273
1.96536334426896
1.9657867854229
1.9660270686363
1.96680468421422
1.96704918335287
1.96723028357545
1.96749460636028
1.96776191329244
1.969332975348
1.9693962958781
1.97007507843334
1.97036842141888
1.97044883220327
1.97138396830859
1.97207069400589
1.97259604360405
1.97318915448765
1.97358063435306
1.97578767615811
1.97623777709494
1.97650870145719
1.97677158855872
1.9772888236821
1.97747064505806
1.97794453251485
1.97815478109015
1.97973806506011
1.98016087066667
1.98132607960459
1.98191855397095
1.98254804672636
1.9828591732242
1.98291874145285
1.98342916557084
1.98398863175539
1.98422102527021
1.98700904498276
1.98920233078668
1.99002624888224
1.99038570542843
1.99240311225205
1.99405026908763
1.99637809759314
1.99720034123467
1.99777798981652
10.005683070058
10.0253457850063
10.0593608789955
10.1435503823785
10.161121328215
10.2039346865253
10.2124843405127
10.2647586626725
10.3765948326639
10.3817375214998
10.4700255865041
10.4750880785348
10.5360157386865
10.5760794854819
10.6424579791831
10.7844321492259
10.8618279068196
10.8630850961685
10.8693965638746
10.8916605107359
10.9187513894762
10.9428228892758
100.588325108607
100036768
100038739
100038753
100038996
100039300
100039375
100040305
100040766
100041230
100041294
100041437
100041932
100042241
100042375
100042970
100043084
100043485
100088
101214
101565
101739
102124
102141
102423
102448
102747
102920
103080
103268
10338120
10338835
10339349
10339471
10340572
10340959
10341342
10341921
10342779
10342997
10344051
10344633
10344723
10344725
10345074
10345529
10345546
10345548
10345550
10345554
10346365
10346764
103468
10347036
10347386
10347748
10348004
10348376
10348521
10349100
10349637
10350090
10350136
10350297
10350392
10350489
10350504
10350590
10350838
10351047
10351140
10351414
10351465
10352048
10352166
10352267
10352306
10352320
10352556
10352709
10352767
10352954
10353004
10353010
10353272
10353420
10353524
10353549
10353674
10353733
10353750
10353947
10353989
10354085
10354275
10354307
10355325
10355327
10355329
10356461
10356780
10356859
10357242
10357391
10357436
10357454
10357808
10358038
10358050
10358389
10358399
10358726
10358754
10359386
10359648
10359851
10359890
10359917
10359948
10360173
10360443
10360662
10360745
10360985
10361110
10361375
10361381
10361651
10361710
10361828
10361995
10362245
10362581
10362633
10362902
10362904
10362941
10363118
10363163
10363312
10363575
10363905
10363962
10364109
10364194
10364502
10364752
10364950
10365574
10365578
10365830
10365833
10365918
10366277
10366293
10366303
10366554
10366705
10366814
10366983
10367076
10367106
10367179
10367292
10367515
10367634
10367641
10368289
10368370
10368508
10368575
10368612
10368845
10369252
10369815
10369885
10369890
10369989
10371107
10371159
10371321
10371591
10371603
10371770
10371888
10372091
10372503
10372629
10372687
10373202
103733
10373454
10373530
10373873
10374106
10374400
10374453
10374455
10375145
10375234
10375463
10375893
10375903
10375909
10375975
10376455
10376459
10376534
10377380
10377405
10377508
10377605
10378053
10378739
10378802
10379127
10379346
10379363
10379646
10379795
10379953
10379968
10379989
10379998
10380210
10380403
10380815
10381072
10381250
10381548
10381664
10381798
10381809
10382136
10382139
10382243
10382257
10382542
10382565
10382797
10382998
10383055
10383395
10383982
10384032
10384145
10384349
10384373
10384782
10384811
10385153
10385236
10385248
10385335
10385455
10385495
10385557
10385686
10385790
10386005
10386236
10386442
10386604
10386909
10387638
10387985
10388234
10388409
10388718
10388880
10389395
10389606
10389627
10389674
10390050
10390352
10390707
10390746
10391130
10391339
10391461
10391768
10391811
10392135
10392284
10392347
10392415
10392437
10392983
10393272
10393395
10393431
10393774
10393844
10394498
10394731
10394770
10394978
10395252
10395277
10395612
10395831
10395908
10396030
10396068
10396094
10396652
10396795
10396849
10397717
10397719
10397741
10397818
10397895
10397966
10398874
10398972
10399011
10399087
10399148
10399178
10399228
10399391
10399430
10399457
10399461
10400030
10400189
10400470
10400589
10400649
10400748
10401852
10402073
10402262
10402294
10402766
10402981
10403413
10403938
10403941
10403943
10403945
10403948
10403978
10403980
10404024
10404026
10404028
10404038
10404049
10404051
10404061
10404063
10404067
10404069
10404422
10405047
10405185
10405189
10405741
10405868
10405994
10406254
10406482
10406519
10406581
10406598
10406733
10406881
10406898
10406968
10407081
10407211
10407390
10407420
10407467
10407481
10407511
10408044
10408072
10408077
10408081
10408083
10408085
10408087
10408092
10408094
10408111
10408113
10408118
10408197
10408200
10408202
10408204
10408210
10408212
10408225
10408239
10408243
10408246
10408266
10408321
10408477
10408677
10408882
10408915
10409031
10409190
10409200
10409322
10409330
10409345
10409643
10409660
10410092
10410207
10410560
10410919
10411019
10411332
10411728
10411739
10411751
10412011
10412054
10412345
10412466
10412559
10412909
10413086
10413138
10413542
10413574
10413710
10414113
10414288
10414315
10414514
10414527
10415645
10415791
10415875
10416006
10416037
10416044
10416290
10416533
10416653
10416655
10416736
10416793
10417070
10417676
10417829
104184
10418804
10418927
10419136
10419296
10419323
10419611
10419779
10420089
10420155
10420198
10420320
10420426
10420637
10420670
10420730
10420877
10420988
10421029
10421810
10421877
10421981
10422608
10422854
10423185
10423498
10424188
10424221
10424349
10424363
10424779
10425046
10425207
10425211
10425226
10425293
10425632
10425757
10425890
10425903
10425905
10425923
10426016
10426225
10426301
10426439
10426669
10427166
10427606
10427816
10427895
10428232
10428310
10428561
10428672
10428763
10429081
10429455
10429968
10430032
10430748
10430778
10430825
10430871
10431035
10431229
10431424
10431856
10431948
10431962
10432398
10432511
10432636
10433088
10433161
10433494
10433721
10434067
10434252
10434643
10434664
10434668
10434782
10434802
10434806
10434869
10435075
10435149
10435329
10435514
10435525
10435581
10436024
10436048
10436106
10436169
10437040
10437292
10437432
10437594
10437639
10437698
10437945
10438091
10438308
10438313
10438378
10438400
10438445
10438572
10438575
10438690
10438738
10439063
10439130
10439346
10439878
10440483
10440794
10441706
10441797
10442435
10442454
10443047
10443215
10444312
10444505
10444595
10444658
10444665
10444858
10445008
10445434
10445640
10445688
10445702
10445941
10446027
10446074
10446229
10446656
10446777
10447004
10447023
10447065
10447097
10447395
10447417
10447880
10447891
10448030
10448089
10448127
10448292
10448506
10448925
10448975
10449295
10449303
10449452
10449575
10449687
10449731
10449989
10450496
10450501
10450519
10450605
10451225
10451434
10451547
10451580
10451805
10452213
10452228
10452709
10452860
10452867
10453026
10453062
10453451
10453512
10453575
10453715
10453825
10453867
10454198
10454286
10454478
10454709
10455439
10455647
10455813
10455873
10455967
10456556
10456566
10456891
10457380
10457409
10457606
10457729
10457853
10458033
10458195
10458213
10458262
10458285
10458428
10458841
10459375
10459576
10459643
10459755
10459844
10460149
10460376
10460573
10460738
10461391
10461439
10461452
10461765
10462100
104625
10462603
10462632
10462796
10462866
10462918
10462973
10463282
10465005
10465282
10465336
10465446
10465638
10465804
10465844
10465861
10465895
10465912
10466410
10466423
10466818
10467041
10467091
10467162
10467637
10467907
10468307
10468489
10468531
10468584
10469035
10469070
10469571
10469712
10469849
10470788
10471844
10471904
104721
10472212
10472350
10472501
10472782
10472893
10472916
10473190
104732
10473250
10473384
10473414
10473737
10473793
10473919
10474223
10474239
10474243
10474381
10474437
10474671
10474689
10474769
10474825
10474875
10474902
10474984
10474998
10475360
10475362
10475378
10475437
10475610
10476033
10476189
10476252
10476362
10476620
10476648
10476889
10476989
10477004
10477187
10477583
10478160
10478196
10478355
10478364
10478572
10478799
10479379
10479765
10479775
10479811
10479833
10480087
10480432
104806
10480628
10480672
10481111
10481291
10481585
10481711
10481931
10482229
10482687
10482762
10482929
10483401
10483563
10484351
10484425
10485340
10485445
10485466
10485562
10485963
10486255
10486396
10487011
10487340
10487480
10487506
10487577
10487622
10487930
104884
10488655
10488785
10488816
10489078
10489127
10489364
10489391
10490104
10490225
10490370
10490894
10490903
10491036
10491182
10491385
10491406
10491551
10491805
10491915
10492205
10492381
10492558
10492658
10492679
10493086
10493137
10493548
10494322
10494386
10494402
10494405
10494407
10494452
10494662
10495035
10495316
10495574
10495685
10495763
10495873
10495935
10495945
10496015
10496204
10496379
10496494
10496796
10497001
10497105
10497122
10497503
10497520
10497689
10497752
10497831
10498058
10498168
10498185
10498309
10498332
10498576
10499035
10499366
10499454
10499483
10499639
10500034
10500054
10500295
10500304
10500324
10500329
10500333
10500543
10500630
10500720
10501402
10501649
10502029
10502510
10503023
10503264
10503315
10503617
10503845
10503911
10504470
10504730
10504743
10504817
10504957
10505064
10505092
10505132
10505276
10505643
10506118
10506603
10506680
10506822
10507112
10507286
10507328
10507653
10507914
10508115
10508151
10508382
10508392
10508420
10508465
10508711
10508759
10508996
10509049
10509161
10509168
10509820
10509858
10510172
10511042
10511207
10511416
10512061
10512065
10512195
10512226
10512236
10512443
10512489
10512757
10512851
10513061
10513181
10513195
10513320
10513622
10513818
10514158
10514366
10514383
10514650
10514658
10514713
10515086
10515090
10515156
10515164
10515257
10515337
10515431
10515744
10515836
10515943
10516246
10516576
10516605
10516778
10516894
10517060
10517141
10517173
10517436
10517682
10518350
10518352
10518484
10518561
10518570
10518714
10519324
10519488
10519516
10520124
10520362
10520521
10521031
10521090
10521136
10521555
10521731
10521757
10522182
10522301
10522668
10522784
10522976
10523012
10523058
10523190
10523541
10523802
10523905
10523923
10524169
10524266
10524450
10524790
10525397
10525542
10525591
10525657
10525983
10526217
10526482
10526502
10526687
10526838
10527158
10527430
10527455
10527598
10527801
10527920
10527940
10527959
10527963
10528077
10528170
10528385
10528648
10528790
10528840
10528915
10529142
10529299
10529613
10530163
10530759
10530892
10531126
10531133
10531261
10531286
10531633
10531737
10531752
10532052
10532753
10532965
10533090
10533474
10533807
10533929
10534281
10534551
10534575
10534585
10534660
10534694
10534974
10534990
10535273
10535629
10535894
10535979
10537179
10537246
10538282
10538420
10538617
10538832
10538842
10539080
10539617
10539640
10539850
10539882
10539894
10540118
10540283
10540579
10540738
10540952
10540999
10541034
10541067
10541091
10541260
10541507
10541711
10541729
10542079
10542164
10542445
10542460
10542691
10542791
10543031
10543067
10543080
10543114
10543859
10544106
10544501
10544570
10544610
10544629
10544660
10545101
10545379
10545534
10545672
10545835
10546088
10546163
10546834
10547088
10547758
10547830
10547936
10547943
10548086
10548176
10548857
10549222
10549341
10550102
10550131
10550326
10550935
10551347
10551554
10551822
10551998
10552311
10552337
10552418
10552740
10553301
10553788
10553935
10553993
10554281
10554325
10554370
10554445
10554938
10555174
10555576
10555620
10555695
10556208
10556266
10556640
10556764
10556790
10557156
10557519
10558248
10558454
10558458
10558723
10560190
10560260
10560304
10561302
10561356
10561388
10561527
10561806
10561868
10562331
10562382
10562563
10562637
10563275
10563780
10563883
10564220
10564565
10564663
10564857
10564978
10565486
10565570
10565862
10566050
10566067
10566254
10566268
10567022
10567072
10567412
10567564
10567663
10568150
10568217
10568461
10568714
10568758
10568897
10569181
10570201
10570373
10571250
10571399
10571514
10571680
10571696
10571815
10571870
10571876
10571911
10572077
10572301
10572449
10572605
10572906
10573194
10573217
10573261
10573451
10573615
10573865
10574033
10574378
10574595
10575550
10575733
10575961
10576883
10576946
10577508
10577534
10577623
10577655
10577757
10578019
10578145
10578155
10578193
10578521
10578690
10578763
10578916
10579437
10579659
10579769
10579830
10580382
10580516
10580590
10580722
10580969
10580986
10581327
10581560
10581729
10581800
10581902
10582008
10582190
10582262
10582275
10582295
10582501
10582599
10582809
10582835
10582981
10583034
10583320
10583324
10583508
10583697
105837
10583952
10584634
10584710
10585358
10585395
10585417
10585586
10585680
10585706
10585932
10586184
10586416
10586448
10586454
10586456
10586484
10586759
10586907
10587495
10587508
10587639
10587780
10588121
10588294
10588482
10588767
10589196
10589420
10589464
10589466
10590149
10590494
10590690
10590892
10590972
10591369
10591412
10591556
10591735
10591773
10591781
10591881
10592058
10592160
10592201
10592585
10592763
10592790
10592802
10592816
10593060
10593490
10593789
10593887
10594221
10594251
10594301
10594315
10594426
10594758
10594774
10594812
10594911
10595371
10595570
10595614
10595702
10595831
10595924
10596190
10596222
10597714
10598586
10598638
105988
10598848
10599612
10599627
10599826
10599893
10600017
10600082
10600476
10600512
10600593
10600765
10600797
10600936
10601011
10601164
10601328
10601335
10601390
10601449
10601471
10601537
10601551
10601567
10601705
10601854
10601857
10601903
10602164
10602223
10602385
10602599
10602795
10603011
10603135
10603431
10603567
10603659
10603785
10603809
10604230
10604405
10604528
10605554
10605674
10605711
10605766
10605943
10606071
10606436
10607062
10607475
10607524
10607747
10607848
10607933
10608656
10608661
10608667
10608671
10608685
10608696
106200
106344
106347
106389
106795
106869
106931
107.960673873336
107271
107272
107321
107435
107652
107823
107869
107993
107995
108000
108037
108115
108121
108143
108671
108811
108900
108907
108911
108912
108961
109079
109145
109212
109242
11.0298403472934
11.0498195372776
11.149388588425
11.1823522684175
11.2666066557973
11.3232497060795
11.3252044862018
11.386474581341
11.405865632444
11.4269168405913
11.4697831516093
11.5626940505935
11.6402642022165
11.677295874618
11.743980099156
11.9264845947662
110033
110052
110074
110196
110208
110312
110350
110460
110749
110750
110809
110826
110842
114584
114714
114715
114716
114741
11486
11502
11534
11628
11637
11641
11651
11677
116870
11769
11777
11799
11946
11947
11957
11982
11991
12.0658314415745
12.1091686582354
12.1241005978815
12.2618131715612
12.3142409344146
12.4115177932777
12.5349526324987
12.7954274238999
12.8519794349607
12.9309173797703
12018
12021
12034
12035
12048
12053
12070
12144
12166
12175
12183
12189
12190
12235
12236
12237
12314
12315
12316
12359
12367
12416
12417
12419
12428
12442
12445
12447
12448
12449
12457
12530
12531
12532
12534
12544
12545
12566
12567
12571
12580
12585
12614
12615
12617
12649
12704
12858
12859
12864
12934
12950
12972
12974
13.1116178997107
13.1343635756736
13.3252116882621
13.3377603109318
13.4094914366125
13.5185390803159
13.6082366589653
13.6427376864868
13.8710832800337
13.9298317230023
13007
13008
13063
13121
13167
13178
13202
13361
13433
13445
13505
13555
13557
13559
13605
13626
13641
13661
13665
13680
13731
13822
13877
13929
14.1528830525591
14.2700275809394
14.3423205213761
14.3430881095241
14.4318524899231
14.5055464942309
14.9138542081585
14.9290169041529
14.9375313219611
14000
140488
14056
140570
14071
14086
14087
14102
14137
14155
14156
14160
14166
14194
14208
14211
14229
14235
14265
14293
14375
14433
14525
14599
14630
14645
14673
14674
14687
14696
14701
14719
14739
14791
14793
14841
14958
15.3241835645549
15.4687418785046
15077
15129
15132
15159
15191
15201
15259
15260
15267
15270
15288
15289
15331
15354
15357
15366
15384
15442
15450
15452
15490
15512
15525
15930
16.0133388080486
16.0589618963899
16.1960568050673
16.6494514760042
16.6627886348734
16.7791100310182
16.9152537875106
16068
16069
16211
16319
16351
16416
16549
16551
16563
16571
16579
16647
16648
16765
16822
16828
16881
16906
16911
16970
16987
16988
17.1574635360895
17.9844512309949
17035
170459
170461
170728
170748
170750
17076
170762
17089
170930
171170
171283
171284
17191
17193
17215
17216
17217
17218
17219
17220
17245
17259
17279
17319
17330
17345
17357
17427
17463
17684
17685
17686
17688
17756
17765
17768
17864
17865
17873
17896
18.3354023977123
18.3426051436125
18.4746733363099
18.7157058122206
18002
18005
18100
18120
18140
18141
18194
18221
18226
18245
18263
18286
18392
18477
18483
18538
18605
18639
18645
18647
18648
18655
18746
18813
18817
18950
18968
18969
18972
18973
18974
19.1043659105221
19.4392219964489
19.8180191799244
19.9039684506156
19011
19012
19027
19045
19047
19072
19075
19076
191578
19172
19177
19179
19182
19183
19185
19193
19205
192170
19231
192657
19348
19357
19359
19361
19362
19363
19366
19383
19384
19385
19387
19646
19650
19656
19687
19699
19714
19718
19735
19739
19826
19891
2.00071155942873
2.00087862040647
2.00353849690792
2.00446762237097
2.00463730809093
2.0054771153408
2.00559193186346
2.00569194115652
2.00644058141765
2.00767609797813
2.00868716464168
2.00870916969608
2.01015061899871
2.0104114335933
2.01081884219541
2.01147073119477
2.01256839197153
2.01376218366602
2.01556607858961
2.01559452556938
2.0166685579007
2.01693409000224
2.01735704078765
2.01760516502217
2.01798923658393
2.01865745475991
2.0201481859773
2.02243376187337
2.02403499066665
2.02550775808586
2.02591397834844
2.02667167657832
2.02715140329971
2.02768155448133
2.02921644698291
2.03192801757345
2.03216519961558
2.03279252851953
2.03429022249653
2.03449156634795
2.03648217250018
2.03654643636397
2.03683742759755
2.03897755413715
2.03903928192478
2.03963821416711
2.04031920389468
2.0403929779485
2.04199391952358
2.04357422769321
2.04530699011682
2.04556755621025
2.04566937070889
2.0475087425525
2.0488740491245
2.05163837048
2.05279439671096
2.05303321365093
2.05313312862752
2.05558403356859
2.05636011116861
2.05665158170745
2.0569525036238
2.05698235750941
2.05802487956699
2.05876004890062
2.0590301327726
2.05923292963834
2.059976874723
2.06053069061911
2.06084816880665
2.06274813068857
2.06558802516599
2.0669377403191
2.06818106368379
2.06880547647074
2.06886171599307
2.07128280743944
2.07153876173358
2.07253301599909
2.07299777742909
2.07375761692362
2.07394531754512
2.07501427461849
2.07517640374102
2.07588671347254
2.0762398916979
2.0779112878628
2.07804635882606
2.0781421503703
2.07903344980546
2.07924443382552
2.08084272994662
2.08334545512599
2.08430961739594
2.08479876555149
2.08574841948327
2.08665326953335
2.08833949940929
2.08957215918125
2.09089385498291
2.09159580171658
2.09376732501837
2.09397645566492
2.09417182523498
2.0953318909723
2.09619377730867
2.09722298260525
2.09727578945846
2.09736493903416
2.09913812020103
2.09999481254946
2.10077879928661
2.10205410045164
2.10356336766125
2.10465122674672
2.10510094138971
2.10809359997719
2.11138977640242
2.1114026882564
2.11174423786071
2.112131535802
2.11403738945164
2.11435455468361
2.11599373420419
2.11738175101668
2.11796330274342
2.12075010994077
2.12111379927227
2.12133867598301
2.12170994602588
2.12224412244027
2.12238446447629
2.12356092003778
2.12411677488391
2.12508563599871
2.12598906856501
2.12618046897178
2.12652572147899
2.12713742655463
2.12751725990969
2.12841867295034
2.1306086015917
2.13100474515919
2.13106922250663
2.13204124800402
2.13281968879887
2.13492193934512
2.13510323074733
2.14472886570015
2.14840355607574
2.14950348347672
2.14975995654802
2.149856902552
2.1542485793173
2.15556059292444
2.15589517681365
2.15757739415033
2.15763456676541
2.15817382195786
2.16051211296459
2.16220154304904
2.16265842346962
2.16467264932023
2.16562644770802
2.16603214934749
2.16632915656115
2.16711380230719
2.17058758501671
2.17074753374597
2.17094374952642
2.1724975824451
2.17364902388541
2.17567386377378
2.17576953278457
2.17587631400387
2.17648291652099
2.17737027051005
2.17765879481113
2.18076906949133
2.18218064555411
2.18446929050862
2.18559840398507
2.18597814036438
2.18626575119305
2.18669111797635
2.18752005432407
2.1877190811484
2.18955638220349
2.19159543512994
2.19253488249929
2.19351274728487
2.19402451857327
2.1942006493925
2.19476491725478
2.19737315524822
2.19997306468267
2.20158224899161
2.20191978617996
2.20233000697903
2.20318679967389
2.20392186845911
2.20627369670273
2.20709395204208
2.21176855948985
2.21193028495849
2.21206564953206
2.21274283439288
2.21562558451942
2.21654222122432
2.21854551654871
2.22063664321495
2.22228500870071
2.22390185268195
2.22458658935041
2.22565222560381
2.2275772863336
2.22866867630994
2.22970665498717
2.23500772480494
2.23701353642069
2.23737898835871
2.23841204305994
2.23858393376802
2.23960874090454
2.2431383902069
2.24435994892155
2.24658602699177
2.24719014518495
2.24727759908086
2.24997470827589
2.25098209814303
2.25127316142515
2.25170094497458
2.25237229382191
2.25550261151629
2.25682634685081
2.2581022255235
2.25851552011511
2.25883132054575
2.25932947678069
2.26032369623668
2.26052844671111
2.26064547658407
2.26168722999753
2.26272113663812
2.26519810778673
2.26526554513446
2.26736339330086
2.26858403638775
2.26893352886989
2.26930603776129
2.2711457981922
2.2713250182792
2.27189339064248
2.27211995696374
2.27514390317657
2.27554845709308
2.27759447467617
2.27828313864912
2.27919884359886
2.2797060634277
2.28005124274341
2.28184611302328
2.28209250778575
2.28630976888268
2.29049141868705
2.29113303765751
2.29123433984572
2.2916619835755
2.29605772619821
2.29637521798256
2.29722367863956
2.29730405450104
2.29869795510967
2.29902912823901
2.30049385189115
2.30077404386377
2.30197931136673
2.30446701059047
2.30620783803106
2.30629675898966
2.3064215035008
2.30650742307546
2.30857574065348
2.31421438241198
2.31445783554172
2.31532147115877
2.31612095629269
2.31621551883366
2.31852001980204
2.31876379582271
2.32095166907528
2.32133703917925
2.32156407876333
2.32361964368618
2.32429814312944
2.32715089692793
2.32740418902974
2.32828316386215
2.32912997129343
2.33133043019264
2.33487635645958
2.33512439863123
2.33573327127101
2.33620830200904
2.33696029406287
2.33723547818726
2.33847700152541
2.33887527091524
2.34130768173189
2.34195371488201
2.34559393441369
2.34861440083527
2.3535823468808
2.35693832280866
2.35801482223652
2.36063324846195
2.36167745407258
2.36217744083172
2.3639364577102
2.36410847524871
2.36415967156116
2.36567406407077
2.36583684173285
2.36731565570898
2.36782106792033
2.37023174673843
2.37087394705657
2.3719929887755
2.37282649741588
2.37386581211423
2.37843583595296
2.37939208452689
2.38092090309188
2.38449455160435
2.39050904438147
2.39176054310849
2.39332814202072
2.39477296656749
2.39528430299085
2.39744140429947
2.40134441495036
2.40210897487898
2.40220118201897
2.40271057530253
2.40348550169181
2.41266104034727
2.41292704230883
2.41311854962405
2.41377921562635
2.41463810536612
2.41633681034895
2.41638278265732
2.41878041429907
2.42466810853678
2.42546462920769
2.42652104749577
2.42987565444222
2.43446552647685
2.43617816578719
2.43909909215877
2.44099684832209
2.44285574611473
2.44558030830672
2.44834879027772
2.44978000295867
2.45191314714429
2.45295502116903
2.45402088799874
2.45417001175979
2.45652445640713
2.45674374345918
2.45796230077671
2.45853966664634
2.46095798696259
2.46170158173368
2.46291491115403
2.46408504076177
2.46445637529259
2.46773759347795
2.46934188798401
2.47134279192619
2.47320423214963
2.4739188787357
2.47402357595698
2.47766163950709
2.47922369533099
2.48353683226232
2.48544241066141
2.48734518324246
2.48880661109586
2.48891868350906
2.4911392901519
2.49136896642191
2.4954557436752
2.49664789235049
2.49910680659946
2.49945126245534
2.50016502398564
2.50198921345776
2.50207294059889
2.50231412428146
2.50438861561838
2.50755224887822
2.51369126437552
2.52166313251404
2.52329494563726
2.52483267002387
2.52638897429833
2.53033280486658
2.53127361176698
2.53192552173349
2.53387913895094
2.53712707151312
2.54122933896364
2.54138629382907
2.54474473958491
2.54593541530664
2.54798220279493
2.54861912137267
2.55113020487208
2.55519922647703
2.55649846694117
2.55808079116521
2.56090555936152
2.56192648033972
2.56262467503452
2.5676695533591
2.56782455584496
2.56837956710595
2.57059734833403
2.57263781028227
2.57457689217293
2.5757561045721
2.57945043314086
2.58001543004073
2.58394945940566
2.58533273897535
2.58795353752764
2.58838260280376
2.5908130571748
2.59445162058987
2.59706055898558
2.60114707190344
2.60461846849628
2.60842172716278
2.6088592269294
2.61253922432899
2.61297063332647
2.61566906447192
2.61645455586292
2.61889410063576
2.62032319501839
2.62078968444108
2.62261773561755
2.62570184555254
2.62826249591168
2.630380693079
2.6313497434512
2.6355486413083
2.63952797097507
2.64044282996831
2.64570994290559
2.64913986908732
2.65672539282857
2.65727686035163
2.66065719132624
2.66172248573978
2.66529665289083
2.66603688811173
2.66728753437814
2.66859014368965
2.66961647882955
2.66997689506231
2.67189435382211
2.67246591374548
2.67323854062688
2.67363805420423
2.67454089870279
2.67790333048554
2.68092315735545
2.68212963129335
2.68385873514301
2.68701079671069
2.6936767283285
2.69589142960236
2.69709937053086
2.69918408001696
2.70261452287023
2.7081686697871
2.7084128850899
2.7133188550334
2.71492271744651
2.71504425800323
2.71526253262057
2.71667742172216
2.71953347501801
2.72080821904138
2.73622566937794
2.74595019228793
2.74851667950832
2.74991887558747
2.75016319895869
2.76553248020602
2.76940938322387
2.77216835567667
2.77241354174915
2.77258803672255
2.77596835514704
2.77634499619601
2.77696568643713
2.78903509319735
2.79068234691646
2.7918514067148
2.79663387123416
2.80422058900783
2.8048740537705
2.81364080662931
2.8164531760198
2.8170675575753
2.81707266092379
2.82369886060541
2.82468912726027
2.83611097310121
2.83681807943762
2.83765201670451
2.85094072941081
2.85113557420493
2.8522480536181
2.85235988057988
2.85810927581555
2.85872476276204
2.86618851601486
2.87052422067876
2.87189382228964
2.87217972273759
2.88351770487487
2.88508772681606
2.88811021464649
2.89143941400286
2.89720825948686
2.89854139903112
2.9006950025997
2.90082907154907
2.90422032545276
2.90500305346947
2.91057312039535
2.9149923186581
2.91821172358222
2.91831788608887
2.91889632722183
2.92616553072775
2.92991726337241
2.93037070357344
2.93342984471768
2.93351279626287
2.93778122478549
2.9388061132533
2.94264086412063
2.9494498046197
2.95253214854577
2.95533777031086
2.95585180936959
2.95675008512172
2.95794619731988
2.95921408221479
2.96028541163125
2.96445054243428
2.96832168591215
2.97351959089197
2.97618697332436
2.97829440159765
2.98285634269729
2.98419986282289
2.98592412902176
2.98609821508962
2.98849886130953
2.99054707862295
2.99216482727654
2.99907926117893
20.1301654825614
20.142142362101
20.5569938484032
20.8950055625817
20.924993949111
20018
20021
20022
20133
20135
20174
20322
20382
20419
20425
20438
20439
20454
20460
20462
20492
20496
20501
20522
20529
20539
20557
20586
20588
20595
20603
20624
20630
20638
20639
20641
20643
20708
207175
20775
20778
207818
208084
20833
208518
208628
208691
208715
20873
20877
20878
208795
208836
20901
20910
209225
209334
20937
209737
21.413972913893
21.6474711702383
210035
210126
210544
210766
211255
211651
212307
212377
213084
213211
21335
21343
213539
21399
214137
21454
214552
214901
215387
215615
216081
216618
21665
21681
217030
217216
217340
217365
217558
217653
21770
21778
21781
217944
217946
21812
218442
21853
21854
218581
21877
21881
218914
218973
218977
219072
219103
219114
21915
21917
21926
21950
21969
21973
21991
22.4378202600216
22036
22042
22064
22092
22137
22143
22154
22166
22171
22224
22247
22256
22273
22294
22334
22335
223453
22364
22365
223691
223723
22381
223828
22390
224171
22439
224829
224836
225182
225280
225363
225659
225745
22594
226043
226144
22627
22629
226409
226414
226539
226744
227613
227937
227960
228012
228410
228421
228482
229279
229317
229363
229841
23.3316225230515
23.4723360585709
23.602481179265
230098
230484
230903
230917
231070
231413
231440
231887
232210
232314
232413
232449
232889
232910
233016
233060
233064
233406
233826
233876
234258
234267
234388
234396
234734
234736
234825
234865
235293
235431
235559
236266
236899
236900
236930
237211
237436
237877
23790
237911
238091
23825
23834
23837
238673
239151
23921
23945
23959
239743
239760
239839
23996
23997
24.0120010364229
24.1480104568834
24.4839331637659
240084
24046
24061
240641
240660
241447
241627
242126
242584
242705
243529
244879
245007
245472
245688
245828
246709
252837
252838
252870
259300
26.0687684251557
26425
26440
26444
26446
26572
268465
268697
26886
268882
26896
269033
26909
26912
269224
26926
26934
26936
269378
269389
269582
269713
269951
270151
27053
270685
270906
27214
27221
272396
272551
27376
27401
27407
27632
276770
277333
27756
28.1775765151027
28.7509878636671
28035
28036
28077
29.9957793978655
2900010J23Rik
29819
29870
3.00175904852634
3.00512745433284
3.00677515530472
3.00879379565105
3.00880897275534
3.01614445388709
3.01924984803732
3.01976830862703
3.02087209870062
3.02225018451216
3.02389355227637
3.02468486507735
3.0285740178831
3.02987483031984
3.03308903744054
3.03697482973848
3.03972026201937
3.04027348843209
3.04401938235038
3.0462970482108
3.0495357914807
3.05563433172506
3.05620196265879
3.05864292192185
3.05899559516453
3.06468614448492
3.06653240262797
3.06727090327716
3.07324156899572
3.07903270222958
3.08537593932455
3.08551256271992
3.09134463691201
3.09217637014711
3.09221219418656
3.09479831824572
3.09564963111616
3.09869319436749
3.10140690804937
3.10294733807362
3.10297014714552
3.10375294705426
3.10944438712096
3.10962948790587
3.11055979263523
3.1161937347569
3.11704707915708
3.11741309493836
3.11741557439324
3.12009181434216
3.12052813384096
3.13202449699136
3.13216007140192
3.13385797588118
3.13617681540269
3.14209245249321
3.14583240898347
3.14727800299193
3.15452337223667
3.15551307135065
3.16376200537327
3.16760440025266
3.17206099200728
3.17325899021252
3.17340953724589
3.17536840174404
3.17878569786183
3.18189392033063
3.18223607781911
3.18690383018047
3.19083828575637
3.19311537600899
3.1998429860889
3.19990914594948
3.20377526216089
3.20891542553855
3.21011360124745
3.2121643043599
3.2193550805624
3.22125817161554
3.22209940952115
3.22390208532633
3.2244298039375
3.22505318633853
3.23042281387926
3.23531382534124
3.24546805508193
3.24751057536992
3.2480408776109
3.25042953126156
3.25546241734335
3.25922412226716
3.26336162939733
3.26813740098747
3.27615726236097
3.28265960548508
3.29524354545452
3.29737267714416
3.30221791426008
3.30354784786009
3.30691741597174
3.30693336937195
3.31803038914384
3.32028226498102
3.32161926416668
3.3220645689575
3.32407295480179
3.3294599702183
3.32987355783357
3.33637174080099
3.34146876595363
3.34464428837085
3.34724830494889
3.35065466665528
3.36377564802814
3.36870687381162
3.37018697413109
3.37302864592441
3.37536560856508
3.38353897662937
3.38864217113723
3.38940536933846
3.39323427767556
3.3944101824921
3.39826991357987
3.42676661540326
3.42730528336981
3.43122714396147
3.43329098354835
3.43588704561181
3.43688642126522
3.44547232389563
3.45626659918264
3.46050549214042
3.47307304746568
3.47452573643207
3.48160279596939
3.4854588420909
3.48722548944622
3.49467532151988
3.50240050839667
3.52986921618295
3.53377311887669
3.53453680476191
3.53638373722942
3.54214189330647
3.54331575915709
3.55363159811471
3.55489320437963
3.55576138091883
3.56651849389105
3.56998440296728
3.57620758635674
3.58397572449604
3.58499269372362
3.59256744286369
3.59383075365763
3.59408399212218
3.59991841822501
3.60405092881916
3.60448250458659
3.6117948089772
3.62572697786758
3.63372506440585
3.63821120206332
3.64448316716012
3.64876897414691
3.64994705256536
3.65482205787485
3.6556469325409
3.65648369288501
3.65918652548326
3.6592823827781
3.66034691610678
3.66893312290029
3.67077862953489
3.67584950393348
3.6904197577231
3.71339515521149
3.72684965429736
3.73538622723663
3.74353886444639
3.74607709024444
3.75581960852942
3.75633916209621
3.78523755466103
3.78896214129634
3.80972946793266
3.81119319984346
3.81259191664693
3.8287152994304
3.83112180073125
3.8337241599444
3.83385792744903
3.87889221384607
3.91414358115154
3.9180549463716
3.92541719550763
3.92642262403371
3.93229937214973
3.93262354174048
3.93505932184983
3.93582970890561
3.93737581589132
3.94026831868572
3.96123735348312
3.96287942761762
3.97106723949749
3.9735655203699
3.98064946402435
3.98592923704867
3.99379987225949
30.9518534588336
30926
30954
31.0546838830916
31.5843218064763
319149
319154
319155
319156
319157
319158
319159
319162
319166
319169
319170
319171
319172
319173
319176
319177
319178
319182
319186
319189
319370
319554
319583
319653
319701
319910
319996
32.4241428048072
32.7798743258163
320226
320299
320332
321022
3230401D17Rik
326620
327762
327799
327957
329165
329207
330149
330409
331487
333883
338365
34.1651943785636
35.206490697106
353172
36.2992754525174
360198
380792
380840
381110
381280
381293
381306
381318
381581
382207
382450
382522
382523
382985
385407
386612
4.00123057109243
4.00154459331538
4.02241786914704
4.03103422531624
4.04442873919264
4.0540091694471
4.08216507907934
4.09601624771222
4.10052547277948
4.11995652813743
4.13798816982824
4.14307987526391
4.14353987752589
4.1447563850635
4.1457161633389
4.14788795277861
4.16723381232815
4.17196819702795
4.17558568487152
4.17617457955521
4.18847633664191
4.19085204303756
4.20097399147746
4.21231846608391
4.22629461383006
4.24107786295458
4.24998382389458
4.25890948503073
4.27442962644719
4.27470864968568
4.27947570100098
4.29550212050308
4.30054791167917
4.30382866211743
4.30959960195336
4.3108598358641
4.31858061849284
4.33736863963138
4.35155790003533
4.3634938710331
4.3990909374043
4.40158302440895
4.41062958426249
4.41244029649082
4.41343495516068
4.41893556092936
4.43586081448235
4.45090250735297
4.4580805013867
4.45971121758666
4.49151542830644
4.51324441477838
4.57427630382361
4.58487103153751
4.59948064479434
4.59999265080202
4.61031428982667
4.62565916420317
4.64654384816453
4.64871786500841
4.6508766515608
4.65183012875957
4.68457073981965
4.68461945262124
4.69436540255163
4.70251282392531
4.70778036281735
4.71182764958256
4.71812985974658
4.7205363924014
4.72527342065562
4.72676494828631
4.74582010125477
4.75254605764015
4.76969104530834
4.77324485331349
4.77450962643477
4.78684740801011
4.78911259447146
4.82234669520355
4.8421620793483
4.84927102555076
4.85124832549495
4.85775282680868
4.85913558200892
4.90960194413442
4.92389905259697
4.92646791693167
4.94301222953898
4.9514797355638
4.95440820595331
4.98064882352438
4.98648681107162
4.99702013316646
40.7636089450209
40.779081203979
404710
407821
432879
432959
433273
433550
433759
433771
433899
434174
434215
434234
435373
445007
45.2970846446972
45.599440841344
4632434I11Rik
5.00679426012444
5.01147700668326
5.08959148602834
5.12765254411714
5.12821768394781
5.146735482949
5.22397680123147
5.24094869465442
5.24512473280371
5.27752597655881
5.31784078430027
5.32238190942293
5.33002712255303
5.33557542185967
5.35674846955759
5.36975309941582
5.38874499527787
5.40267280772464
5.40673540590482
5.42207188784826
5.4244265486792
5.42768648724634
5.43853480858368
5.44003949197247
5.44616639641055
5.44832092285625
5.45958555786494
5.46914100716254
5.47726423596574
5.48001821647534
5.48914136904563
5.49289081121441
5.50414234582237
5.50672708736147
5.50716514466575
5.51980180847891
5.53078823003807
5.5723577850467
5.60301506803771
5.60539746620677
5.6089319992552
5.62215890562419
5.62643908708848
5.62866478278933
5.63190469952475
5.6456900629513
5.67975586799683
5.68197376093736
5.70498247557267
5.72324237483793
5.7446392540026
5.74684020379387
5.77555816988155
5.79013641595262
5.81525712919891
5.81628429334028
5.81730883956313
5.86965452132853
5.87263907454499
5.88714242019106
5.89773997525207
5.96624601976661
5.97860645997748
50.1925413646353
50497
50529
50709
50770
50783
50799
50849
50883
50887
50911
50927
50933
50995
51786
51788
51793
51797
51944
52033
52076
52276
52432
52502
52552
52585
52592
52615
52626
52637
52653
52679
52683
52696
52822
52864
53376
53381
53382
53414
53600
53610
53893
54124
54141
54156
54188
54366
54375
54392
54447
54486
545555
545600
545741
546362
54650
547431
55982
56030
56043
56075
56150
56207
56210
56224
56228
56278
56298
56349
56371
56378
56418
56419
56421
56427
56428
56431
56443
56452
56456
56459
56473
56488
56505
56532
56702
56705
56726
56742
56752
57373
57434
57440
57441
57913
58.4291242488546
58184
58186
58207
58226
59001
59014
59025
59056
59126
6.00976122219541
6.02791207443445
6.08815641900625
6.09502232213883
6.09885323252979
6.12590155409978
6.15514322037493
6.17425560460946
6.18980335077382
6.19099961619946
6.22145312513776
6.2238065705268
6.2444966343338
6.25578768125637
6.28818960948188
6.33607862166711
6.35995547791322
6.41857397220915
6.48299803514352
6.49537196654467
6.49602590169511
6.49781359020086
6.50068452024456
6.50192848669613
6.5039948348082
6.53869108639819
6.553291915562
6.59112099136828
6.59862324785706
6.62757714119227
6.6277259187983
6.63267423723736
6.63709690105852
6.65040418545133
6.65378278389536
6.66665731783074
6.67149403306016
6.70830582145479
6.7140012539683
6.73489589444796
6.78837078824602
6.79482907397745
6.93878111962349
6.95391917832231
6.97984061669324
6.99164123320016
60365
60406
60411
60530
60595
619605
621156
621880
623474
625534
625801
626075
626115
627245
627480
628431
629557
633596
636070
638833
639787
64009
64050
64113
64707
64898
65.2215146113983
65099
65111
65964
66046
66047
66118
66126
66131
66140
66163
66193
66197
66214
66231
66234
66235
66310
66311
66336
66350
66368
66373
66377
66390
66397
66403
66422
66442
66464
66468
66471
66493
664953
66514
66525
66531
66537
665570
665596
665622
66570
66578
665806
66588
66592
66634
666348
66637
66671
66680
66686
66695
667034
667247
667519
66801
668192
668253
668275
668321
66861
66912
66916
66929
66931
66934
66953
66977
66980
66995
66997
67.9693331578317
67037
67041
67052
67054
67070
67078
67089
67092
67103
67117
67121
67141
67144
67153
67179
67187
67196
67203
67220
67242
67255
67276
67283
67286
67333
67465
67492
67501
67533
67574
67628
67629
67664
67675
67678
67680
67681
67683
67710
67733
67824
67834
67840
67849
67966
67967
67979
68014
68026
68041
68051
68070
68097
68106
68149
68184
68187
68201
68215
68219
68243
68250
68275
68278
68298
68475
68479
68501
68549
68565
68603
68612
68729
68735
68743
68815
68816
68861
68926
68948
68964
68970
68981
69029
69048
69051
69066
69071
69186
69241
69263
69270
69386
69554
69639
69706
69716
69736
69823
69860
69871
69885
69912
69922
69928
7.08666739525851
7.10030970030346
7.13742698442254
7.14533544678413
7.175479918385
7.18421286543104
7.21974915763181
7.32566246043899
7.35656582104143
7.41718367360132
7.4522136067951
7.49569936233149
7.55379579081885
7.56551417681835
7.57123483302959
7.59016657757493
7.59932219187247
7.61425799983157
7.62648855412534
7.64593903423963
7.65769388262853
7.66681321395485
7.695943638644
7.69854889869681
7.78116936418736
7.79235164424779
7.87110161263094
7.87595425388564
7.9218123001862
7.99857090938055
70012
70021
70024
70099
70155
70218
70235
70239
70247
70248
70350
70385
70454
70458
70466
70472
70546
70572
70645
70699
70853
70998
70999
71085
71091
71151
71177
71198
71242
71643
71648
71720
71735
71745
71764
71795
71804
71805
71819
71835
71846
71876
71878
71919
71924
71934
71957
71984
71988
72018
72077
72107
72114
72119
72123
72151
72155
72190
72193
72391
72400
72415
72440
72475
72549
72568
72612
72621
72655
72657
72662
72749
72774
72776
72787
72931
73139
73167
73225
73246
73254
73327
73666
73739
73804
73916
73937
74016
74038
74041
74044
74107
74147
74178
74192
74205
74237
74325
74360
74513
74682
74734
74769
75062
75212
75216
75234
75292
75317
75345
75423
75430
75597
75623
75735
75786
75939
75985
76044
76123
76131
76138
76187
76267
76299
76302
76464
76843
76863
76890
76915
77011
77045
77134
77252
77605
77626
77744
77782
77891
78317
78465
78658
78733
78767
78833
8.03290122998573
8.03489876874283
8.08928542264798
8.09447157145818
8.17531334586452
8.22389516276403
8.23401540837813
8.32863245914087
8.33610300338056
8.33720041608941
8.35876296523237
8.35900737201455
8.39877846179016
8.40533303190278
8.42486298646318
8.43119197968716
8.53348153749943
8.58167433140092
8.64866728885833
8.64947049036475
8.75920977328766
8.81425007527919
8.90495787660784
8.98903201234761
80838
80905
80914
80986
81896
83397
83701
83703
84.8296401305807
9.00068872671129
9.02560048301675
9.0453413609741
9.04960321500188
9.10415085894228
9.19521194622251
9.23264320901308
9.31020210689443
9.32386163505756
9.35923256491024
9.5827130902522
9.60770317968586
9.6493740855786
9.68491180030051
9.70772367856051
9.73977586161528
9.74693778486618
9.85341203989457
9.93435937964182
9.95209860797882
9.96384705377387
9.97633608722468
9.98852701135352
93685
93765
93966
94181
94223
94245
94275
94353
97122
97165
97287
97820
97908
98053
98238
98386
98878
99045
99167
FoldChange
Gm12260
Gm12372
Gm12387
Gm13310
Gm13675
Gm15470
Gm16373
Gm16380
Gm3338
Gm3744
Gm3809
Gm4217
Gm4471
Gm4797
Gm4929
Gm4943
Gm4989
Gm5069
Gm5177
Gm5393
Gm5473
Gm5523
Gm5666
Gm5853
Gm5863
Gm5943
Gm6625
Gm6651
Gm6762
Gm6981
Gm7172
Gm7251
Gm7278
Gm7423
Gm7693
Gm7795
Gm8681
Gm9034
Gm9081
Gm9106
Hbb
Hist1h2ab
Hist1h4b
Hist1h4c
Hist2h2aa1
Hist2h3b
Hist3h2bb
Hmgn2l6
Ipp
Lrmp
MGI:104764
MGI:108424
MGI:1201610
MGI:1913930
MGI:1921291
MGI:1922442
MGI:2448306
MGI:2448351
MGI:2448420
MGI:2448421
MGI:3641891
MGI:3642996
MGI:3643000
MGI:3643126
MGI:3643526
MGI:3643564
MGI:3643568
MGI:3643639
MGI:3643860
MGI:3644095
MGI:3644454
MGI:3644516
MGI:3644727
MGI:3645174
MGI:3645261
MGI:3645941
MGI:3646004
MGI:3646014
MGI:3646144
MGI:3646158
MGI:3646579
MGI:3646657
MGI:3646966
MGI:3647491
MGI:3647663
MGI:3648307
MGI:3648580
MGI:3648664
MGI:3648707
MGI:3649039
MGI:3649089
MGI:3649138
MGI:3649532
MGI:3650289
MGI:3650665
MGI:3651326
MGI:3651413
MGI:3708112
MGI:3779834
MGI:3781516
MGI:3781919
MGI:3781982
MGI:3782393
MGI:3782655
MGI:96097
MGI:96581
MGI:98938
NA
Ska2l
Stdv
Tex9
Vpreb3
b1
bh1
entrezIDs
mgiID
ps
ps2
rs17
symbol

|  |  |  |
| --- | --- | --- |
| [close] | **Legend: Edge Types** | (details) |
|  | | |
