# Supplementary material for: Ontology based molecular signatures for immune cell types via gene expression analysis
Source: BMC Bioinformatics. 2013 Aug 30;14:263. doi: 10.1186/1471-2105-14-263 (PMC3844401; doi:10.1186/1471-2105-14-263)
Supplement: Additional file 1 — OBAMS profiles for all mature B cells. Additional file 1 contains a zip archive of OBAMS profiles for all mature B cells, including for each cell type individual spreadsheets showing up and down regulated genes for that cell type relative to parental cell types, and VLAD (GO term enrichment) results for all mature B cells. [file 1471-2105-14-263-S1.zip › Additional File 1/mature B cell vs Mature Lymphocte/VLAD.CL_0000785-up/results.html]

# Vlad Results

|  |  |
| --- | --- |
| Vlad version: | v1.5 |
| Date: | Tue Oct 25 10:03:56 2011 |
| Run time: | 67.48 sec |
| Ontology file: | gene\_ontology.obo |
| Ontology date: | Mon Oct 24 19:30:00 2011 |
| Annotation file: | gene\_association.mgi |
| Annotation date: | Wed Oct 19 00:00:00 2011 |
| Analysis type: | enrichment |
| Excluded evidence codes: | ND |
| Number of query sets: | 1 |
| Query set 1: | Mature B cell (n=134; 45 not found) |
| Universe set: | default (everything) |
| Graph display: | Top 25 scoring terms and their ancestors. Interior nodes have been culled. |

**Jump to:** biological\_process | cellular\_component | molecular\_function | Unannotated id/symbols

### biological\_process (top)

  
  


### cellular\_component (top)

  
  


### molecular\_function (top)

  
  


### Unannotated IDs

**Mature B cell**  
MGI:1330829
MGI:1330831
MGI:1330836
MGI:1891441
MGI:1917970
MGI:2685035
MGI:2685305
MGI:2685923
MGI:3026916
MGI:3525017
MGI:3612703
MGI:3641634
MGI:3642083
MGI:3642250
MGI:3642251
MGI:3642277
MGI:3642626
MGI:3642722
MGI:3642817
MGI:3644536
MGI:3645228
MGI:3645298
MGI:3645723
MGI:3646808
MGI:3647784
MGI:3704124
MGI:3705861
MGI:3708581
MGI:3710533
MGI:4439557
MGI:4439558
MGI:4439561
MGI:4439633
MGI:4439634
MGI:4439641
MGI:4439716
MGI:4439774
MGI:4439784
MGI:4439824
MGI:4439879
MGI:4439894
MGI:96495
MGI:96530
MGI:99548
NA

|  |  |  |
| --- | --- | --- |
| [close] | **Legend: Edge Types** | (details) |
|  | | |
