# Supplementary material for: Ontology based molecular signatures for immune cell types via gene expression analysis
Source: BMC Bioinformatics. 2013 Aug 30;14:263. doi: 10.1186/1471-2105-14-263 (PMC3844401; doi:10.1186/1471-2105-14-263)
Supplement: Additional file 1 — OBAMS profiles for all mature B cells. Additional file 1 contains a zip archive of OBAMS profiles for all mature B cells, including for each cell type individual spreadsheets showing up and down regulated genes for that cell type relative to parental cell types, and VLAD (GO term enrichment) results for all mature B cells. [file 1471-2105-14-263-S1.zip › Additional File 1/mature B cell vs Mature Lymphocte/VLAD.CL_0000785_down/results.html]

# mature\_B\_cell\_down

|  |  |
| --- | --- |
| Vlad version: | v1.5.1 |
| Date: | Wed Jun 20 07:34:15 2012 |
| Run time: | 68.06 sec |
| Ontology file: | gene\_ontology.obo |
| Ontology date: | Tue Jun 19 19:30:00 2012 |
| Annotation file: | gene\_association.mgi |
| Annotation date: | Wed Jun 6 00:00:00 2012 |
| Analysis type: | enrichment |
| Excluded evidence codes: | ND |
| Number of query sets: | 1 |
| Query set 1: | mature\_B\_cell\_down (n=57; 1 not found) |
| Universe set: | default (everything) |
| Graph display: | Top 25 scoring terms and their ancestors. Interior nodes have been culled. |

**Jump to:** biological\_process | cellular\_component | molecular\_function | Unannotated id/symbols

### biological\_process (top)

  
  


| TermID | Term | P | Q | k | n | K | N | k/n | K/N | k/K | n/N | Qset | Symbols |
| --- | --- | --- | --- | --- | --- | --- | --- | --- | --- | --- | --- | --- | --- |
| GO:0050852 | T cell receptor signaling pathway | 3.67e-14 | 5.55e-11 | 8 | 49 | 30 | 15137 | 16.33% | 0.20% | 26.67% | 0.32% | mature\_B\_cell\_down | Cd247, Fyn, Gata3, Itk, Skap1, Thy1, Txk, Zap70 |
| GO:0050851 | antigen receptor-mediated signaling pathway | 1.21e-13 | 9.18e-11 | 9 | 49 | 56 | 15137 | 18.37% | 0.37% | 16.07% | 0.32% | mature\_B\_cell\_down | Cd247, Fyn, Gata3, Itk, Lck, Skap1, Thy1, Txk, Zap70 |
| GO:0002429 | immune response-activating cell surface receptor signaling pathway | 5.79e-13 | 2.92e-10 | 9 | 49 | 66 | 15137 | 18.37% | 0.44% | 13.64% | 0.32% | mature\_B\_cell\_down | Cd247, Fyn, Gata3, Itk, Lck, Skap1, Thy1, Txk, Zap70 |
| GO:0002768 | immune response-regulating cell surface receptor signaling pathway | 1.70e-12 | 6.41e-10 | 9 | 49 | 74 | 15137 | 18.37% | 0.49% | 12.16% | 0.32% | mature\_B\_cell\_down | Cd247, Fyn, Gata3, Itk, Lck, Skap1, Thy1, Txk, Zap70 |
| GO:0002757 | immune response-activating signal transduction | 1.89e-11 | 5.71e-09 | 9 | 49 | 96 | 15137 | 18.37% | 0.63% | 9.38% | 0.32% | mature\_B\_cell\_down | Cd247, Fyn, Gata3, Itk, Lck, Skap1, Thy1, Txk, Zap70 |
| GO:0002764 | immune response-regulating signaling pathway | 4.67e-11 | 1.18e-08 | 9 | 49 | 106 | 15137 | 18.37% | 0.70% | 8.49% | 0.32% | mature\_B\_cell\_down | Cd247, Fyn, Gata3, Itk, Lck, Skap1, Thy1, Txk, Zap70 |
| GO:0050778 | positive regulation of immune response | 8.54e-11 | 1.85e-08 | 11 | 49 | 220 | 15137 | 22.45% | 1.45% | 5.00% | 0.32% | mature\_B\_cell\_down | Cd226, Cd247, Fyn, Gata3, Itk, Lck, Prkcq, Skap1, Thy1, Txk, Zap70 |
| GO:0002684 | positive regulation of immune system process | 2.47e-10 | 4.67e-08 | 13 | 49 | 396 | 15137 | 26.53% | 2.62% | 3.28% | 0.32% | mature\_B\_cell\_down | Cd226, Cd247, Cd27, Fyn, Gata3, Itk, Lck, Prkch, Prkcq, Skap1, Thy1, Txk, Zap70 |
| GO:0002253 | activation of immune response | 3.88e-10 | 6.52e-08 | 9 | 49 | 134 | 15137 | 18.37% | 0.89% | 6.72% | 0.32% | mature\_B\_cell\_down | Cd247, Fyn, Gata3, Itk, Lck, Skap1, Thy1, Txk, Zap70 |
| GO:0032633 | interleukin-4 production | 1.45e-09 | 2.19e-07 | 4 | 49 | 6 | 15137 | 8.16% | 0.04% | 66.67% | 0.32% | mature\_B\_cell\_down | Gata3, Itk, Notch1, Txk |
| GO:0001775 | cell activation | 1.87e-09 | 2.47e-07 | 12 | 49 | 376 | 15137 | 24.49% | 2.48% | 3.19% | 0.32% | mature\_B\_cell\_down | Cd27, F2r, Fyn, Gata3, Itk, Lat, Lck, Lcp2, Sla2, Tcf7, Txk, Zap70 |
| GO:0048584 | positive regulation of response to stimulus | 1.96e-09 | 2.47e-07 | 17 | 49 | 923 | 15137 | 34.69% | 6.10% | 1.84% | 0.32% | mature\_B\_cell\_down | Apol7b, Cd226, Cd247, Cd27, F2r, Fyn, Gata3, Itk, Lck, Notch1, Prkch, Prkcq, Skap1, Thy1, Tnfrsf1a, Txk, Zap70 |
| GO:0045321 | leukocyte activation | 5.15e-09 | 5.99e-07 | 11 | 49 | 324 | 15137 | 22.45% | 2.14% | 3.40% | 0.32% | mature\_B\_cell\_down | Cd27, Fyn, Gata3, Itk, Lat, Lck, Lcp2, Sla2, Tcf7, Txk, Zap70 |
| GO:0050776 | regulation of immune response | 6.84e-09 | 7.40e-07 | 11 | 49 | 333 | 15137 | 22.45% | 2.20% | 3.30% | 0.32% | mature\_B\_cell\_down | Cd226, Cd247, Fyn, Gata3, Itk, Lck, Prkcq, Skap1, Thy1, Txk, Zap70 |
| GO:0002376 | immune system process | 9.12e-09 | 9.20e-07 | 17 | 49 | 1022 | 15137 | 34.69% | 6.75% | 1.66% | 0.32% | mature\_B\_cell\_down | Cd247, Cd27, Fyn, Gata3, Ifih1, Itk, Lat, Lck, Lcp2, Notch1, Selplg, Skap1, Sla2, Tcf7, Thy1, Txk, Zap70 |
| GO:0002682 | regulation of immune system process | 1.28e-08 | 1.21e-06 | 14 | 49 | 660 | 15137 | 28.57% | 4.36% | 2.12% | 0.32% | mature\_B\_cell\_down | Cd226, Cd247, Cd27, Fyn, Gata3, Itk, Lat, Lck, Prkch, Prkcq, Skap1, Thy1, Txk, Zap70 |
| GO:0046632 | alpha-beta T cell differentiation | 5.43e-08 | 4.84e-06 | 5 | 49 | 32 | 15137 | 10.20% | 0.21% | 15.62% | 0.32% | mature\_B\_cell\_down | Gata3, Itk, Tcf7, Txk, Zap70 |
| GO:0042110 | T cell activation | 6.54e-08 | 5.50e-06 | 8 | 49 | 170 | 15137 | 16.33% | 1.12% | 4.71% | 0.32% | mature\_B\_cell\_down | Fyn, Gata3, Itk, Lck, Sla2, Tcf7, Txk, Zap70 |
| GO:0046649 | lymphocyte activation | 1.46e-07 | 1.16e-05 | 9 | 49 | 264 | 15137 | 18.37% | 1.74% | 3.41% | 0.32% | mature\_B\_cell\_down | Cd27, Fyn, Gata3, Itk, Lck, Sla2, Tcf7, Txk, Zap70 |
| GO:0046631 | alpha-beta T cell activation | 1.53e-07 | 1.16e-05 | 5 | 49 | 39 | 15137 | 10.20% | 0.26% | 12.82% | 0.32% | mature\_B\_cell\_down | Gata3, Itk, Tcf7, Txk, Zap70 |
| GO:0001816 | cytokine production | 2.26e-07 | 1.63e-05 | 6 | 49 | 81 | 15137 | 12.24% | 0.54% | 7.41% | 0.32% | mature\_B\_cell\_down | Cd226, Gata3, Itk, Lcp2, Notch1, Txk |
| GO:0048583 | regulation of response to stimulus | 8.46e-07 | 5.82e-05 | 20 | 49 | 1933 | 15137 | 40.82% | 12.77% | 1.03% | 0.32% | mature\_B\_cell\_down | Apol7b, Cd226, Cd247, Cd27, F2r, Fyn, Gata3, Itk, Lck, Notch1, Prkch, Prkcq, Skap1, Sla2, Slc9a3r1, Tbc1d2b, Thy1, Tnfrsf1a, Txk, Zap70 |
| GO:0030217 | T cell differentiation | 1.32e-06 | 8.47e-05 | 6 | 49 | 109 | 15137 | 12.24% | 0.72% | 5.50% | 0.32% | mature\_B\_cell\_down | Gata3, Itk, Lck, Tcf7, Txk, Zap70 |
| GO:0050863 | regulation of T cell activation | 1.34e-06 | 8.47e-05 | 7 | 49 | 174 | 15137 | 14.29% | 1.15% | 4.02% | 0.32% | mature\_B\_cell\_down | Cd27, Gata3, Lat, Lck, Prkcq, Thy1, Zap70 |
| GO:0050870 | positive regulation of T cell activation | 1.63e-06 | 9.81e-05 | 6 | 49 | 113 | 15137 | 12.24% | 0.75% | 5.31% | 0.32% | mature\_B\_cell\_down | Cd27, Gata3, Lck, Prkcq, Thy1, Zap70 |
| GO:0002696 | positive regulation of leukocyte activation | 1.69e-06 | 9.81e-05 | 7 | 49 | 180 | 15137 | 14.29% | 1.19% | 3.89% | 0.32% | mature\_B\_cell\_down | Cd226, Cd27, Gata3, Lck, Prkcq, Thy1, Zap70 |
| GO:0009966 | regulation of signal transduction | 2.28e-06 | 1.26e-04 | 17 | 49 | 1498 | 15137 | 34.69% | 9.90% | 1.13% | 0.32% | mature\_B\_cell\_down | Apol7b, Cd226, Cd27, F2r, Fyn, Gata3, Lck, Notch1, Prkch, Prkcq, Sla2, Slc9a3r1, Tbc1d2b, Thy1, Tnfrsf1a, Txk, Zap70 |
| GO:0050867 | positive regulation of cell activation | 2.33e-06 | 1.26e-04 | 7 | 49 | 189 | 15137 | 14.29% | 1.25% | 3.70% | 0.32% | mature\_B\_cell\_down | Cd226, Cd27, Gata3, Lck, Prkcq, Thy1, Zap70 |
| GO:0010646 | regulation of cell communication | 2.53e-06 | 1.32e-04 | 16 | 49 | 1337 | 15137 | 32.65% | 8.83% | 1.20% | 0.32% | mature\_B\_cell\_down | Apol7b, Cd226, Cd27, F2r, Fyn, Gata3, Lck, Notch1, Prkch, Prkcq, Sla2, Slc9a3r1, Thy1, Tnfrsf1a, Txk, Zap70 |
| GO:0002694 | regulation of leukocyte activation | 3.86e-06 | 1.94e-04 | 8 | 49 | 291 | 15137 | 16.33% | 1.92% | 2.75% | 0.32% | mature\_B\_cell\_down | Cd226, Cd27, Gata3, Lat, Lck, Prkcq, Thy1, Zap70 |
| GO:0050865 | regulation of cell activation | 6.44e-06 | 3.14e-04 | 8 | 49 | 312 | 15137 | 16.33% | 2.06% | 2.56% | 0.32% | mature\_B\_cell\_down | Cd226, Cd27, Gata3, Lat, Lck, Prkcq, Thy1, Zap70 |
| GO:0009967 | positive regulation of signal transduction | 6.66e-06 | 3.15e-04 | 11 | 49 | 663 | 15137 | 22.45% | 4.38% | 1.66% | 0.32% | mature\_B\_cell\_down | Apol7b, Cd226, Cd27, F2r, Gata3, Lck, Notch1, Prkch, Tnfrsf1a, Txk, Zap70 |
| GO:0032609 | interferon-gamma production | 6.87e-06 | 3.15e-04 | 3 | 49 | 12 | 15137 | 6.12% | 0.08% | 25.00% | 0.32% | mature\_B\_cell\_down | Gata3, Itk, Txk |
| GO:0009306 | protein secretion | 9.91e-06 | 4.41e-04 | 4 | 49 | 42 | 15137 | 8.16% | 0.28% | 9.52% | 0.32% | mature\_B\_cell\_down | Cd27, Gata3, Lcp2, Notch1 |
| GO:0001865 | NK T cell differentiation | 1.03e-05 | 4.44e-04 | 2 | 49 | 2 | 15137 | 4.08% | 0.01% | 100.00% | 0.32% | mature\_B\_cell\_down | Itk, Txk |
| GO:0048518 | positive regulation of biological process | 1.26e-05 | 5.21e-04 | 24 | 49 | 3183 | 15137 | 48.98% | 21.03% | 0.75% | 0.32% | mature\_B\_cell\_down | Apol7b, Cd226, Cd247, Cd27, F2r, Fyn, Gata3, Hnrpll, Ifih1, Ipcef1, Itk, Lck, Lpin1, Notch1, Prkch, Prkcq, Skap1, Stk39, Sytl2, Thy1, Tnfrsf1a, Tnik, Txk, Zap70 |
| GO:0030098 | lymphocyte differentiation | 1.31e-05 | 5.21e-04 | 6 | 49 | 162 | 15137 | 12.24% | 1.07% | 3.70% | 0.32% | mature\_B\_cell\_down | Gata3, Itk, Lck, Tcf7, Txk, Zap70 |
| GO:0051251 | positive regulation of lymphocyte activation | 1.31e-05 | 5.21e-04 | 6 | 49 | 162 | 15137 | 12.24% | 1.07% | 3.70% | 0.32% | mature\_B\_cell\_down | Cd27, Gata3, Lck, Prkcq, Thy1, Zap70 |
| GO:0010647 | positive regulation of cell communication | 1.34e-05 | 5.21e-04 | 11 | 49 | 714 | 15137 | 22.45% | 4.72% | 1.54% | 0.32% | mature\_B\_cell\_down | Apol7b, Cd226, Cd27, F2r, Gata3, Lck, Notch1, Prkch, Tnfrsf1a, Txk, Zap70 |
| GO:0051249 | regulation of lymphocyte activation | 1.47e-05 | 5.43e-04 | 7 | 49 | 250 | 15137 | 14.29% | 1.65% | 2.80% | 0.32% | mature\_B\_cell\_down | Cd27, Gata3, Lat, Lck, Prkcq, Thy1, Zap70 |
| GO:0023056 | positive regulation of signaling | 1.47e-05 | 5.43e-04 | 11 | 49 | 721 | 15137 | 22.45% | 4.76% | 1.53% | 0.32% | mature\_B\_cell\_down | Apol7b, Cd226, Cd27, F2r, Gata3, Lck, Notch1, Prkch, Tnfrsf1a, Txk, Zap70 |
| GO:0023051 | regulation of signaling | 1.70e-05 | 6.14e-04 | 17 | 49 | 1738 | 15137 | 34.69% | 11.48% | 0.98% | 0.32% | mature\_B\_cell\_down | Apol7b, Cd226, Cd27, F2r, Fyn, Gata3, Lck, Notch1, Prkch, Prkcq, Sla2, Slc9a3r1, Tbc1d2b, Thy1, Tnfrsf1a, Txk, Zap70 |
| GO:0046427 | positive regulation of JAK-STAT cascade | 2.17e-05 | 7.62e-04 | 4 | 49 | 51 | 15137 | 8.16% | 0.34% | 7.84% | 0.32% | mature\_B\_cell\_down | F2r, Lck, Notch1, Tnfrsf1a |
| GO:0045582 | positive regulation of T cell differentiation | 2.53e-05 | 8.68e-04 | 4 | 49 | 53 | 15137 | 8.16% | 0.35% | 7.55% | 0.32% | mature\_B\_cell\_down | Cd27, Gata3, Lck, Zap70 |
| GO:0008104 | protein localization | 2.59e-05 | 8.72e-04 | 13 | 49 | 1076 | 15137 | 26.53% | 7.11% | 1.21% | 0.32% | mature\_B\_cell\_down | Cd27, Cd3g, F2r, Fam125b, Gata3, Lcp2, Nckap1, Notch1, Rab37, Slc9a3r1, Sytl2, Vps26b, Zdhhc15 |
| GO:0050663 | cytokine secretion | 2.98e-05 | 9.80e-04 | 3 | 49 | 19 | 15137 | 6.12% | 0.13% | 15.79% | 0.32% | mature\_B\_cell\_down | Gata3, Lcp2, Notch1 |
| GO:0045184 | establishment of protein localization | 4.32e-05 | 1.39e-03 | 11 | 49 | 810 | 15137 | 22.45% | 5.35% | 1.36% | 0.32% | mature\_B\_cell\_down | Cd27, Cd3g, F2r, Fam125b, Gata3, Lcp2, Notch1, Rab37, Sytl2, Vps26b, Zdhhc15 |
| GO:0045621 | positive regulation of lymphocyte differentiation | 4.71e-05 | 1.48e-03 | 4 | 49 | 62 | 15137 | 8.16% | 0.41% | 6.45% | 0.32% | mature\_B\_cell\_down | Cd27, Gata3, Lck, Zap70 |
| GO:0018212 | peptidyl-tyrosine modification | 6.03e-05 | 1.79e-03 | 4 | 49 | 66 | 15137 | 8.16% | 0.44% | 6.06% | 0.32% | mature\_B\_cell\_down | F2r, Fyn, Lck, Zap70 |
| GO:0018108 | peptidyl-tyrosine phosphorylation | 6.03e-05 | 1.79e-03 | 4 | 49 | 66 | 15137 | 8.16% | 0.44% | 6.06% | 0.32% | mature\_B\_cell\_down | F2r, Fyn, Lck, Zap70 |
| GO:0072602 | interleukin-4 secretion | 6.13e-05 | 1.79e-03 | 2 | 49 | 4 | 15137 | 4.08% | 0.03% | 50.00% | 0.32% | mature\_B\_cell\_down | Gata3, Notch1 |
| GO:0065009 | regulation of molecular function | 6.16e-05 | 1.79e-03 | 16 | 49 | 1720 | 15137 | 32.65% | 11.36% | 0.93% | 0.32% | mature\_B\_cell\_down | Cd27, F2r, Gata3, Itk, Lck, Notch1, Prkch, Prkcq, Rinl, Slc9a3r1, Sytl2, Tbc1d2b, Thy1, Tmem66, Tnik, Txk |
| GO:0002521 | leukocyte differentiation | 7.49e-05 | 2.14e-03 | 6 | 49 | 221 | 15137 | 12.24% | 1.46% | 2.71% | 0.32% | mature\_B\_cell\_down | Gata3, Itk, Lck, Tcf7, Txk, Zap70 |
| GO:0050854 | regulation of antigen receptor-mediated signaling pathway | 7.87e-05 | 2.20e-03 | 3 | 49 | 26 | 15137 | 6.12% | 0.17% | 11.54% | 0.32% | mature\_B\_cell\_down | Lck, Prkch, Thy1 |
| GO:0046903 | secretion | 8.11e-05 | 2.23e-03 | 7 | 49 | 327 | 15137 | 14.29% | 2.16% | 2.14% | 0.32% | mature\_B\_cell\_down | Cd27, Gata3, Lat, Lcp2, Notch1, Slc9a3r1, Sytl2 |
| GO:0046425 | regulation of JAK-STAT cascade | 8.95e-05 | 2.42e-03 | 4 | 49 | 73 | 15137 | 8.16% | 0.48% | 5.48% | 0.32% | mature\_B\_cell\_down | F2r, Lck, Notch1, Tnfrsf1a |
| GO:0001817 | regulation of cytokine production | 9.79e-05 | 2.60e-03 | 7 | 49 | 337 | 15137 | 14.29% | 2.23% | 2.08% | 0.32% | mature\_B\_cell\_down | Cd27, F2r, Gata3, Ifih1, Prkcq, Tnfrsf1a, Txk |
| GO:0003176 | aortic valve development | 1.02e-04 | 2.62e-03 | 2 | 49 | 5 | 15137 | 4.08% | 0.03% | 40.00% | 0.32% | mature\_B\_cell\_down | Gata3, Notch1 |
| GO:0003180 | aortic valve morphogenesis | 1.02e-04 | 2.62e-03 | 2 | 49 | 5 | 15137 | 4.08% | 0.03% | 40.00% | 0.32% | mature\_B\_cell\_down | Gata3, Notch1 |
| GO:0006468 | protein phosphorylation | 1.06e-04 | 2.68e-03 | 10 | 49 | 740 | 15137 | 20.41% | 4.89% | 1.35% | 0.32% | mature\_B\_cell\_down | F2r, Fyn, Itk, Lck, Prkch, Prkcq, Stk39, Tnik, Txk, Zap70 |
| GO:0045580 | regulation of T cell differentiation | 1.16e-04 | 2.87e-03 | 4 | 49 | 78 | 15137 | 8.16% | 0.52% | 5.13% | 0.32% | mature\_B\_cell\_down | Cd27, Gata3, Lck, Zap70 |
| GO:0010740 | positive regulation of intracellular protein kinase cascade | 1.17e-04 | 2.87e-03 | 7 | 49 | 347 | 15137 | 14.29% | 2.29% | 2.02% | 0.32% | mature\_B\_cell\_down | Apol7b, Cd27, F2r, Gata3, Lck, Notch1, Tnfrsf1a |
| GO:0033036 | macromolecule localization | 1.25e-04 | 3.01e-03 | 13 | 49 | 1253 | 15137 | 26.53% | 8.28% | 1.04% | 0.32% | mature\_B\_cell\_down | Cd27, Cd3g, F2r, Fam125b, Gata3, Lcp2, Nckap1, Notch1, Rab37, Slc9a3r1, Sytl2, Vps26b, Zdhhc15 |
| GO:0046777 | protein autophosphorylation | 1.40e-04 | 3.31e-03 | 5 | 49 | 155 | 15137 | 10.20% | 1.02% | 3.23% | 0.32% | mature\_B\_cell\_down | Fyn, Lck, Tnik, Txk, Zap70 |
| GO:0071353 | cellular response to interleukin-4 | 1.53e-04 | 3.55e-03 | 2 | 49 | 6 | 15137 | 4.08% | 0.04% | 33.33% | 0.32% | mature\_B\_cell\_down | Gata3, Tcf7 |
| GO:0051240 | positive regulation of multicellular organismal process | 1.69e-04 | 3.87e-03 | 8 | 49 | 494 | 15137 | 16.33% | 3.26% | 1.62% | 0.32% | mature\_B\_cell\_down | F2r, Gata3, Ifih1, Lck, Prkcq, Sytl2, Tnfrsf1a, Txk |
| GO:0015031 | protein transport | 1.73e-04 | 3.90e-03 | 10 | 49 | 785 | 15137 | 20.41% | 5.19% | 1.27% | 0.32% | mature\_B\_cell\_down | Cd27, Cd3g, F2r, Fam125b, Gata3, Lcp2, Notch1, Rab37, Sytl2, Vps26b |
| GO:0032940 | secretion by cell | 1.82e-04 | 4.06e-03 | 6 | 49 | 260 | 15137 | 12.24% | 1.72% | 2.31% | 0.32% | mature\_B\_cell\_down | Cd27, Gata3, Lat, Lcp2, Notch1, Sytl2 |
| GO:0001819 | positive regulation of cytokine production | 2.67e-04 | 5.85e-03 | 5 | 49 | 178 | 15137 | 10.20% | 1.18% | 2.81% | 0.32% | mature\_B\_cell\_down | Gata3, Ifih1, Prkcq, Tnfrsf1a, Txk |
| GO:0010627 | regulation of intracellular protein kinase cascade | 2.72e-04 | 5.89e-03 | 8 | 49 | 530 | 15137 | 16.33% | 3.50% | 1.51% | 0.32% | mature\_B\_cell\_down | Apol7b, Cd27, F2r, Gata3, Lck, Notch1, Slc9a3r1, Tnfrsf1a |
| GO:0045618 | positive regulation of keratinocyte differentiation | 2.84e-04 | 5.97e-03 | 2 | 49 | 8 | 15137 | 4.08% | 0.05% | 25.00% | 0.32% | mature\_B\_cell\_down | Notch1, Prkch |
| GO:0070670 | response to interleukin-4 | 2.84e-04 | 5.97e-03 | 2 | 49 | 8 | 15137 | 4.08% | 0.05% | 25.00% | 0.32% | mature\_B\_cell\_down | Gata3, Tcf7 |
| GO:0051239 | regulation of multicellular organismal process | 2.91e-04 | 6.03e-03 | 15 | 49 | 1754 | 15137 | 30.61% | 11.59% | 0.86% | 0.32% | mature\_B\_cell\_down | Cd27, F2r, Gata3, Ifih1, Lck, Notch1, Prkch, Prkcq, Slc9a3r1, Sytl2, Thy1, Tnfrsf1a, Tnik, Txk, Zap70 |
| GO:0045619 | regulation of lymphocyte differentiation | 3.14e-04 | 6.42e-03 | 4 | 49 | 101 | 15137 | 8.16% | 0.67% | 3.96% | 0.32% | mature\_B\_cell\_down | Cd27, Gata3, Lck, Zap70 |
| GO:0045606 | positive regulation of epidermal cell differentiation | 3.64e-04 | 7.35e-03 | 2 | 49 | 9 | 15137 | 4.08% | 0.06% | 22.22% | 0.32% | mature\_B\_cell\_down | Notch1, Prkch |
| GO:0010959 | regulation of metal ion transport | 4.35e-04 | 8.66e-03 | 5 | 49 | 198 | 15137 | 10.20% | 1.31% | 2.53% | 0.32% | mature\_B\_cell\_down | F2r, Slc9a3r1, Stk39, Thy1, Tmem66 |
| GO:0042981 | regulation of apoptotic process | 4.84e-04 | 9.50e-03 | 11 | 49 | 1065 | 15137 | 22.45% | 7.04% | 1.03% | 0.32% | mature\_B\_cell\_down | Cd27, Cd3g, F2r, Gata3, Ifih1, Lck, Notch1, Prkch, Prkcq, Tcf7, Tnfrsf1a |
| GO:0006955 | immune response | 5.06e-04 | 9.82e-03 | 7 | 49 | 441 | 15137 | 14.29% | 2.91% | 1.59% | 0.32% | mature\_B\_cell\_down | Gata3, Ifih1, Itk, Lat, Notch1, Txk, Zap70 |
| GO:0043067 | regulation of programmed cell death | 5.23e-04 | 1.00e-02 | 11 | 49 | 1075 | 15137 | 22.45% | 7.10% | 1.02% | 0.32% | mature\_B\_cell\_down | Cd27, Cd3g, F2r, Gata3, Ifih1, Lck, Notch1, Prkch, Prkcq, Tcf7, Tnfrsf1a |
| GO:0032753 | positive regulation of interleukin-4 production | 5.54e-04 | 1.05e-02 | 2 | 49 | 11 | 15137 | 4.08% | 0.07% | 18.18% | 0.32% | mature\_B\_cell\_down | Gata3, Prkcq |
| GO:0048546 | digestive tract morphogenesis | 5.95e-04 | 1.11e-02 | 3 | 49 | 51 | 15137 | 6.12% | 0.34% | 5.88% | 0.32% | mature\_B\_cell\_down | Nckap1, Notch1, Tcf7 |
| GO:0007440 | foregut morphogenesis | 6.64e-04 | 1.22e-02 | 2 | 49 | 12 | 15137 | 4.08% | 0.08% | 16.67% | 0.32% | mature\_B\_cell\_down | Nckap1, Notch1 |
| GO:0010941 | regulation of cell death | 7.06e-04 | 1.29e-02 | 11 | 49 | 1114 | 15137 | 22.45% | 7.36% | 0.99% | 0.32% | mature\_B\_cell\_down | Cd27, Cd3g, F2r, Gata3, Ifih1, Lck, Notch1, Prkch, Prkcq, Tcf7, Tnfrsf1a |
| GO:0035556 | intracellular signal transduction | 7.21e-04 | 1.30e-02 | 10 | 49 | 939 | 15137 | 20.41% | 6.20% | 1.06% | 0.32% | mature\_B\_cell\_down | F2r, Gata3, Itk, Lat, Prkch, Prkcq, Rab37, Slc9a3r1, Tnik, Zap70 |
| GO:0007202 | activation of phospholipase C activity | 7.83e-04 | 1.39e-02 | 2 | 49 | 13 | 15137 | 4.08% | 0.09% | 15.38% | 0.32% | mature\_B\_cell\_down | Itk, Txk |
| GO:0051050 | positive regulation of transport | 9.10e-04 | 1.58e-02 | 7 | 49 | 487 | 15137 | 14.29% | 3.22% | 1.44% | 0.32% | mature\_B\_cell\_down | F2r, Gata3, Prkcq, Stk39, Sytl2, Thy1, Tnfrsf1a |
| GO:0003215 | cardiac right ventricle morphogenesis | 9.11e-04 | 1.58e-02 | 2 | 49 | 14 | 15137 | 4.08% | 0.09% | 14.29% | 0.32% | mature\_B\_cell\_down | Gata3, Notch1 |
| GO:0050790 | regulation of catalytic activity | 9.18e-04 | 1.58e-02 | 12 | 49 | 1339 | 15137 | 24.49% | 8.85% | 0.90% | 0.32% | mature\_B\_cell\_down | Cd27, F2r, Itk, Lck, Notch1, Rinl, Slc9a3r1, Sytl2, Tbc1d2b, Thy1, Tnik, Txk |
| GO:0032879 | regulation of localization | 9.49e-04 | 1.61e-02 | 12 | 49 | 1344 | 15137 | 24.49% | 8.88% | 0.89% | 0.32% | mature\_B\_cell\_down | F2r, Gata3, Lck, Nckap1, Notch1, Prkcq, Slc9a3r1, Stk39, Sytl2, Thy1, Tmem66, Tnfrsf1a |
| GO:0046634 | regulation of alpha-beta T cell activation | 9.58e-04 | 1.61e-02 | 3 | 49 | 60 | 15137 | 6.12% | 0.40% | 5.00% | 0.32% | mature\_B\_cell\_down | Gata3, Prkcq, Zap70 |
| GO:0016310 | phosphorylation | 1.00e-03 | 1.64e-02 | 10 | 49 | 980 | 15137 | 20.41% | 6.47% | 1.02% | 0.32% | mature\_B\_cell\_down | F2r, Fyn, Itk, Lck, Prkch, Prkcq, Stk39, Tnik, Txk, Zap70 |
| GO:0006796 | phosphate-containing compound metabolic process | 1.00e-03 | 1.64e-02 | 11 | 49 | 1162 | 15137 | 22.45% | 7.68% | 0.95% | 0.32% | mature\_B\_cell\_down | F2r, Fyn, Itk, Lck, Lpin1, Prkch, Prkcq, Stk39, Tnik, Txk, Zap70 |
| GO:0030856 | regulation of epithelial cell differentiation | 1.01e-03 | 1.64e-02 | 3 | 49 | 61 | 15137 | 6.12% | 0.40% | 4.92% | 0.32% | mature\_B\_cell\_down | Gata3, Notch1, Prkch |
| GO:0006793 | phosphorus metabolic process | 1.02e-03 | 1.64e-02 | 11 | 49 | 1164 | 15137 | 22.45% | 7.69% | 0.95% | 0.32% | mature\_B\_cell\_down | F2r, Fyn, Itk, Lck, Lpin1, Prkch, Prkcq, Stk39, Tnik, Txk, Zap70 |
| GO:0051281 | positive regulation of release of sequestered calcium ion into cytosol | 1.20e-03 | 1.91e-02 | 2 | 49 | 16 | 15137 | 4.08% | 0.11% | 12.50% | 0.32% | mature\_B\_cell\_down | F2r, Thy1 |
| GO:0051049 | regulation of transport | 1.28e-03 | 2.02e-02 | 10 | 49 | 1012 | 15137 | 20.41% | 6.69% | 0.99% | 0.32% | mature\_B\_cell\_down | F2r, Gata3, Notch1, Prkcq, Slc9a3r1, Stk39, Sytl2, Thy1, Tmem66, Tnfrsf1a |
| GO:0051246 | regulation of protein metabolic process | 1.35e-03 | 2.08e-02 | 11 | 49 | 1205 | 15137 | 22.45% | 7.96% | 0.91% | 0.32% | mature\_B\_cell\_down | Cd27, F2r, Fyn, Gata3, Lck, Lpin1, Prkcq, Slc9a3r1, Thy1, Tnfrsf1a, Tnik |
| GO:0060251 | regulation of glial cell proliferation | 1.35e-03 | 2.08e-02 | 2 | 49 | 17 | 15137 | 4.08% | 0.11% | 11.76% | 0.32% | mature\_B\_cell\_down | Notch1, Prkch |
| GO:0006954 | inflammatory response | 1.36e-03 | 2.08e-02 | 5 | 49 | 255 | 15137 | 10.20% | 1.68% | 1.96% | 0.32% | mature\_B\_cell\_down | F2r, Lat, Notch1, Prkcq, Tnfrsf1a |
| GO:0032649 | regulation of interferon-gamma production | 1.38e-03 | 2.08e-02 | 3 | 49 | 68 | 15137 | 6.12% | 0.45% | 4.41% | 0.32% | mature\_B\_cell\_down | Cd27, Gata3, Txk |
| GO:0051222 | positive regulation of protein transport | 1.42e-03 | 2.11e-02 | 4 | 49 | 151 | 15137 | 8.16% | 1.00% | 2.65% | 0.32% | mature\_B\_cell\_down | F2r, Gata3, Prkcq, Tnfrsf1a |
| GO:0030097 | hemopoiesis | 1.43e-03 | 2.11e-02 | 6 | 49 | 384 | 15137 | 12.24% | 2.54% | 1.56% | 0.32% | mature\_B\_cell\_down | Gata3, Itk, Lck, Tcf7, Txk, Zap70 |
| GO:0044093 | positive regulation of molecular function | 1.45e-03 | 2.11e-02 | 9 | 49 | 851 | 15137 | 18.37% | 5.62% | 1.06% | 0.32% | mature\_B\_cell\_down | F2r, Gata3, Itk, Lck, Prkch, Prkcq, Thy1, Tnik, Txk |
| GO:0009968 | negative regulation of signal transduction | 1.47e-03 | 2.11e-02 | 7 | 49 | 529 | 15137 | 14.29% | 3.49% | 1.32% | 0.32% | mature\_B\_cell\_down | Fyn, Gata3, Notch1, Prkcq, Sla2, Slc9a3r1, Thy1 |
| GO:0051336 | regulation of hydrolase activity | 1.50e-03 | 2.11e-02 | 8 | 49 | 687 | 15137 | 16.33% | 4.54% | 1.16% | 0.32% | mature\_B\_cell\_down | Cd27, F2r, Itk, Lck, Sytl2, Tbc1d2b, Thy1, Txk |
| GO:0032413 | negative regulation of ion transmembrane transporter activity | 1.52e-03 | 2.11e-02 | 2 | 49 | 18 | 15137 | 4.08% | 0.12% | 11.11% | 0.32% | mature\_B\_cell\_down | Slc9a3r1, Tmem66 |
| GO:0045684 | positive regulation of epidermis development | 1.52e-03 | 2.11e-02 | 2 | 49 | 18 | 15137 | 4.08% | 0.12% | 11.11% | 0.32% | mature\_B\_cell\_down | Notch1, Prkch |
| GO:0010863 | positive regulation of phospholipase C activity | 1.52e-03 | 2.11e-02 | 2 | 49 | 18 | 15137 | 4.08% | 0.12% | 11.11% | 0.32% | mature\_B\_cell\_down | Itk, Txk |
| GO:0045616 | regulation of keratinocyte differentiation | 1.52e-03 | 2.11e-02 | 2 | 49 | 18 | 15137 | 4.08% | 0.12% | 11.11% | 0.32% | mature\_B\_cell\_down | Notch1, Prkch |
| GO:0080134 | regulation of response to stress | 1.66e-03 | 2.28e-02 | 7 | 49 | 540 | 15137 | 14.29% | 3.57% | 1.30% | 0.32% | mature\_B\_cell\_down | Cd226, Cd27, F2r, Gata3, Prkcq, Tnfrsf1a, Txk |
| GO:0045576 | mast cell activation | 1.69e-03 | 2.29e-02 | 2 | 49 | 19 | 15137 | 4.08% | 0.13% | 10.53% | 0.32% | mature\_B\_cell\_down | Lat, Lcp2 |
| GO:1900274 | regulation of phospholipase C activity | 1.69e-03 | 2.29e-02 | 2 | 49 | 19 | 15137 | 4.08% | 0.13% | 10.53% | 0.32% | mature\_B\_cell\_down | Itk, Txk |
| GO:0050856 | regulation of T cell receptor signaling pathway | 1.88e-03 | 2.52e-02 | 2 | 49 | 20 | 15137 | 4.08% | 0.13% | 10.00% | 0.32% | mature\_B\_cell\_down | Lck, Thy1 |
| GO:0003179 | heart valve morphogenesis | 2.07e-03 | 2.70e-02 | 2 | 49 | 21 | 15137 | 4.08% | 0.14% | 9.52% | 0.32% | mature\_B\_cell\_down | Gata3, Notch1 |
| GO:0032673 | regulation of interleukin-4 production | 2.07e-03 | 2.70e-02 | 2 | 49 | 21 | 15137 | 4.08% | 0.14% | 9.52% | 0.32% | mature\_B\_cell\_down | Gata3, Prkcq |
| GO:0045061 | thymic T cell selection | 2.07e-03 | 2.70e-02 | 2 | 49 | 21 | 15137 | 4.08% | 0.14% | 9.52% | 0.32% | mature\_B\_cell\_down | Gata3, Zap70 |
| GO:0050714 | positive regulation of protein secretion | 2.12e-03 | 2.74e-02 | 3 | 49 | 79 | 15137 | 6.12% | 0.52% | 3.80% | 0.32% | mature\_B\_cell\_down | F2r, Gata3, Prkcq |
| GO:0023057 | negative regulation of signaling | 2.21e-03 | 2.83e-02 | 7 | 49 | 568 | 15137 | 14.29% | 3.75% | 1.23% | 0.32% | mature\_B\_cell\_down | Fyn, Gata3, Notch1, Prkcq, Sla2, Slc9a3r1, Thy1 |
| GO:0010648 | negative regulation of cell communication | 2.23e-03 | 2.83e-02 | 7 | 49 | 569 | 15137 | 14.29% | 3.76% | 1.23% | 0.32% | mature\_B\_cell\_down | Fyn, Gata3, Notch1, Prkcq, Sla2, Slc9a3r1, Thy1 |
| GO:0051046 | regulation of secretion | 2.24e-03 | 2.83e-02 | 6 | 49 | 420 | 15137 | 12.24% | 2.77% | 1.43% | 0.32% | mature\_B\_cell\_down | F2r, Gata3, Notch1, Prkcq, Slc9a3r1, Sytl2 |
| GO:0051179 | localization | 2.31e-03 | 2.87e-02 | 20 | 49 | 3328 | 15137 | 40.82% | 21.99% | 0.60% | 0.32% | mature\_B\_cell\_down | Cd27, Cd3g, F2r, Fam125b, Fyn, Gata3, Lat, Lck, Lcp2, Nckap1, Notch1, Rab37, Rinl, Selplg, Slc12a7, Slc9a3r1, Sytl2, Tmem66, Vps26b, Zdhhc15 |
| GO:0051345 | positive regulation of hydrolase activity | 2.32e-03 | 2.87e-02 | 5 | 49 | 288 | 15137 | 10.20% | 1.90% | 1.74% | 0.32% | mature\_B\_cell\_down | F2r, Itk, Lck, Thy1, Txk |
| GO:0003170 | heart valve development | 2.49e-03 | 3.00e-02 | 2 | 49 | 23 | 15137 | 4.08% | 0.15% | 8.70% | 0.32% | mature\_B\_cell\_down | Gata3, Notch1 |
| GO:0045987 | positive regulation of smooth muscle contraction | 2.49e-03 | 3.00e-02 | 2 | 49 | 23 | 15137 | 4.08% | 0.15% | 8.70% | 0.32% | mature\_B\_cell\_down | F2r, Lck |
| GO:0045604 | regulation of epidermal cell differentiation | 2.49e-03 | 3.00e-02 | 2 | 49 | 23 | 15137 | 4.08% | 0.15% | 8.70% | 0.32% | mature\_B\_cell\_down | Notch1, Prkch |
| GO:0048534 | hemopoietic or lymphoid organ development | 2.50e-03 | 3.00e-02 | 6 | 49 | 429 | 15137 | 12.24% | 2.83% | 1.40% | 0.32% | mature\_B\_cell\_down | Gata3, Itk, Lck, Tcf7, Txk, Zap70 |
| GO:0048565 | digestive tract development | 2.53e-03 | 3.01e-02 | 3 | 49 | 84 | 15137 | 6.12% | 0.55% | 3.57% | 0.32% | mature\_B\_cell\_down | Nckap1, Notch1, Tcf7 |
| GO:0019222 | regulation of metabolic process | 2.73e-03 | 3.12e-02 | 24 | 49 | 4423 | 15137 | 48.98% | 29.22% | 0.54% | 0.32% | mature\_B\_cell\_down | Cd27, F2r, Fyn, Gata3, Hnrpll, Ipcef1, Itk, Lck, Lpin1, Nckap1, Notch1, Prkch, Prkcq, Rinl, Skap1, Sla2, Slc9a3r1, Sytl2, Tbc1d2b, Tcf7, Thy1, Tnfrsf1a, Tnik, Txk |
| GO:0048522 | positive regulation of cellular process | 2.74e-03 | 3.12e-02 | 18 | 49 | 2878 | 15137 | 36.73% | 19.01% | 0.63% | 0.32% | mature\_B\_cell\_down | Apol7b, Cd226, Cd27, F2r, Gata3, Hnrpll, Ipcef1, Lck, Lpin1, Notch1, Prkch, Prkcq, Skap1, Thy1, Tnfrsf1a, Tnik, Txk, Zap70 |
| GO:0042035 | regulation of cytokine biosynthetic process | 2.79e-03 | 3.12e-02 | 3 | 49 | 87 | 15137 | 6.12% | 0.57% | 3.45% | 0.32% | mature\_B\_cell\_down | Cd27, Gata3, Prkcq |
| GO:0006464 | cellular protein modification process | 2.80e-03 | 3.12e-02 | 13 | 49 | 1735 | 15137 | 26.53% | 11.46% | 0.75% | 0.32% | mature\_B\_cell\_down | Art2b, F2r, Fyn, Ifih1, Itk, Lck, Prkch, Prkcq, Stk39, Tnik, Txk, Zap70, Zdhhc15 |
| GO:0036211 | protein modification process | 2.80e-03 | 3.12e-02 | 13 | 49 | 1735 | 15137 | 26.53% | 11.46% | 0.75% | 0.32% | mature\_B\_cell\_down | Art2b, F2r, Fyn, Ifih1, Itk, Lck, Prkch, Prkcq, Stk39, Tnik, Txk, Zap70, Zdhhc15 |
| GO:0006952 | defense response | 2.89e-03 | 3.12e-02 | 7 | 49 | 596 | 15137 | 14.29% | 3.94% | 1.17% | 0.32% | mature\_B\_cell\_down | F2r, Ifi47, Ifih1, Lat, Notch1, Prkcq, Tnfrsf1a |
| GO:0048873 | homeostasis of number of cells within a tissue | 2.94e-03 | 3.12e-02 | 2 | 49 | 25 | 15137 | 4.08% | 0.17% | 8.00% | 0.32% | mature\_B\_cell\_down | Cd7, F2r |
| GO:0032410 | negative regulation of transporter activity | 2.94e-03 | 3.12e-02 | 2 | 49 | 25 | 15137 | 4.08% | 0.17% | 8.00% | 0.32% | mature\_B\_cell\_down | Slc9a3r1, Tmem66 |
| GO:0006810 | transport | 3.10e-03 | 3.12e-02 | 17 | 49 | 2667 | 15137 | 34.69% | 17.62% | 0.64% | 0.32% | mature\_B\_cell\_down | Cd27, Cd3g, F2r, Fam125b, Gata3, Lat, Lck, Lcp2, Notch1, Rab37, Rinl, Slc12a7, Slc9a3r1, Sytl2, Tmem66, Vps26b, Zdhhc15 |
| GO:0002520 | immune system development | 3.20e-03 | 3.12e-02 | 6 | 49 | 451 | 15137 | 12.24% | 2.98% | 1.33% | 0.32% | mature\_B\_cell\_down | Gata3, Itk, Lck, Tcf7, Txk, Zap70 |
| GO:0061314 | Notch signaling involved in heart development | 3.24e-03 | 3.12e-02 | 1 | 49 | 1 | 15137 | 2.04% | 0.01% | 100.00% | 0.32% | mature\_B\_cell\_down | Notch1 |
| GO:0003270 | Notch signaling pathway involved in regulation of secondary heart field cardioblast proliferation | 3.24e-03 | 3.12e-02 | 1 | 49 | 1 | 15137 | 2.04% | 0.01% | 100.00% | 0.32% | mature\_B\_cell\_down | Notch1 |
| GO:0045175 | basal protein localization | 3.24e-03 | 3.12e-02 | 1 | 49 | 1 | 15137 | 2.04% | 0.01% | 100.00% | 0.32% | mature\_B\_cell\_down | Nckap1 |
| GO:0032782 | bile acid secretion | 3.24e-03 | 3.12e-02 | 1 | 49 | 1 | 15137 | 2.04% | 0.01% | 100.00% | 0.32% | mature\_B\_cell\_down | Slc9a3r1 |
| GO:0060374 | mast cell differentiation | 3.24e-03 | 3.12e-02 | 1 | 49 | 1 | 15137 | 2.04% | 0.01% | 100.00% | 0.32% | mature\_B\_cell\_down | Gata3 |
| GO:2000607 | negative regulation of cell proliferation involved in mesonephros development | 3.24e-03 | 3.12e-02 | 1 | 49 | 1 | 15137 | 2.04% | 0.01% | 100.00% | 0.32% | mature\_B\_cell\_down | Gata3 |
| GO:2000703 | negative regulation of fibroblast growth factor receptor signaling pathway involved in ureteric bud formation | 3.24e-03 | 3.12e-02 | 1 | 49 | 1 | 15137 | 2.04% | 0.01% | 100.00% | 0.32% | mature\_B\_cell\_down | Gata3 |
| GO:2000734 | negative regulation of glial cell-derived neurotrophic factor receptor signaling pathway involved in ureteric bud formation | 3.24e-03 | 3.12e-02 | 1 | 49 | 1 | 15137 | 2.04% | 0.01% | 100.00% | 0.32% | mature\_B\_cell\_down | Gata3 |
| GO:1900134 | negative regulation of renin secretion into blood stream | 3.24e-03 | 3.12e-02 | 1 | 49 | 1 | 15137 | 2.04% | 0.01% | 100.00% | 0.32% | mature\_B\_cell\_down | F2r |
| GO:0018120 | peptidyl-arginine ADP-ribosylation | 3.24e-03 | 3.12e-02 | 1 | 49 | 1 | 15137 | 2.04% | 0.01% | 100.00% | 0.32% | mature\_B\_cell\_down | Art2b |
| GO:2000570 | positive regulation of T-helper 2 cell activation | 3.24e-03 | 3.12e-02 | 1 | 49 | 1 | 15137 | 2.04% | 0.01% | 100.00% | 0.32% | mature\_B\_cell\_down | Prkcq |
| GO:0061419 | positive regulation of transcription from RNA polymerase II promoter in response to hypoxia | 3.24e-03 | 3.12e-02 | 1 | 49 | 1 | 15137 | 2.04% | 0.01% | 100.00% | 0.32% | mature\_B\_cell\_down | Notch1 |
| GO:2000569 | regulation of T-helper 2 cell activation | 3.24e-03 | 3.12e-02 | 1 | 49 | 1 | 15137 | 2.04% | 0.01% | 100.00% | 0.32% | mature\_B\_cell\_down | Prkcq |
| GO:2000606 | regulation of cell proliferation involved in mesonephros development | 3.24e-03 | 3.12e-02 | 1 | 49 | 1 | 15137 | 2.04% | 0.01% | 100.00% | 0.32% | mature\_B\_cell\_down | Gata3 |
| GO:2000702 | regulation of fibroblast growth factor receptor signaling pathway involved in ureteric bud formation | 3.24e-03 | 3.12e-02 | 1 | 49 | 1 | 15137 | 2.04% | 0.01% | 100.00% | 0.32% | mature\_B\_cell\_down | Gata3 |
| GO:2000733 | regulation of glial cell-derived neurotrophic factor receptor signaling pathway involved in ureteric bud formation | 3.24e-03 | 3.12e-02 | 1 | 49 | 1 | 15137 | 2.04% | 0.01% | 100.00% | 0.32% | mature\_B\_cell\_down | Gata3 |
| GO:0061085 | regulation of histone H3-K27 methylation | 3.24e-03 | 3.12e-02 | 1 | 49 | 1 | 15137 | 2.04% | 0.01% | 100.00% | 0.32% | mature\_B\_cell\_down | Gata3 |
| GO:0061217 | regulation of mesonephros development | 3.24e-03 | 3.12e-02 | 1 | 49 | 1 | 15137 | 2.04% | 0.01% | 100.00% | 0.32% | mature\_B\_cell\_down | Gata3 |
| GO:0014807 | regulation of somitogenesis | 3.24e-03 | 3.12e-02 | 1 | 49 | 1 | 15137 | 2.04% | 0.01% | 100.00% | 0.32% | mature\_B\_cell\_down | Notch1 |
| GO:0097291 | renal phosphate ion absorption | 3.24e-03 | 3.12e-02 | 1 | 49 | 1 | 15137 | 2.04% | 0.01% | 100.00% | 0.32% | mature\_B\_cell\_down | Slc9a3r1 |
| GO:0055123 | digestive system development | 3.27e-03 | 3.13e-02 | 3 | 49 | 92 | 15137 | 6.12% | 0.61% | 3.26% | 0.32% | mature\_B\_cell\_down | Nckap1, Notch1, Tcf7 |
| GO:0010524 | positive regulation of calcium ion transport into cytosol | 3.42e-03 | 3.22e-02 | 2 | 49 | 27 | 15137 | 4.08% | 0.18% | 7.41% | 0.32% | mature\_B\_cell\_down | F2r, Thy1 |
| GO:0030858 | positive regulation of epithelial cell differentiation | 3.42e-03 | 3.22e-02 | 2 | 49 | 27 | 15137 | 4.08% | 0.18% | 7.41% | 0.32% | mature\_B\_cell\_down | Notch1, Prkch |
| GO:0051279 | regulation of release of sequestered calcium ion into cytosol | 3.42e-03 | 3.22e-02 | 2 | 49 | 27 | 15137 | 4.08% | 0.18% | 7.41% | 0.32% | mature\_B\_cell\_down | F2r, Thy1 |
| GO:0032880 | regulation of protein localization | 3.64e-03 | 3.35e-02 | 5 | 49 | 320 | 15137 | 10.20% | 2.11% | 1.56% | 0.32% | mature\_B\_cell\_down | F2r, Gata3, Nckap1, Prkcq, Tnfrsf1a |
| GO:0045058 | T cell selection | 3.68e-03 | 3.35e-02 | 2 | 49 | 28 | 15137 | 4.08% | 0.18% | 7.14% | 0.32% | mature\_B\_cell\_down | Gata3, Zap70 |
| GO:0051283 | negative regulation of sequestering of calcium ion | 3.68e-03 | 3.35e-02 | 2 | 49 | 28 | 15137 | 4.08% | 0.18% | 7.14% | 0.32% | mature\_B\_cell\_down | F2r, Lck |
| GO:0010518 | positive regulation of phospholipase activity | 3.68e-03 | 3.35e-02 | 2 | 49 | 28 | 15137 | 4.08% | 0.18% | 7.14% | 0.32% | mature\_B\_cell\_down | Itk, Txk |
| GO:0051209 | release of sequestered calcium ion into cytosol | 3.68e-03 | 3.35e-02 | 2 | 49 | 28 | 15137 | 4.08% | 0.18% | 7.14% | 0.32% | mature\_B\_cell\_down | F2r, Lck |
| GO:0051234 | establishment of localization | 3.81e-03 | 3.46e-02 | 17 | 49 | 2719 | 15137 | 34.69% | 17.96% | 0.63% | 0.32% | mature\_B\_cell\_down | Cd27, Cd3g, F2r, Fam125b, Gata3, Lat, Lck, Lcp2, Notch1, Rab37, Rinl, Slc12a7, Slc9a3r1, Sytl2, Tmem66, Vps26b, Zdhhc15 |
| GO:0045933 | positive regulation of muscle contraction | 3.94e-03 | 3.51e-02 | 2 | 49 | 29 | 15137 | 4.08% | 0.19% | 6.90% | 0.32% | mature\_B\_cell\_down | F2r, Lck |
| GO:2000514 | regulation of CD4-positive, alpha-beta T cell activation | 3.94e-03 | 3.51e-02 | 2 | 49 | 29 | 15137 | 4.08% | 0.19% | 6.90% | 0.32% | mature\_B\_cell\_down | Gata3, Prkcq |
| GO:0051282 | regulation of sequestering of calcium ion | 3.94e-03 | 3.51e-02 | 2 | 49 | 29 | 15137 | 4.08% | 0.19% | 6.90% | 0.32% | mature\_B\_cell\_down | F2r, Lck |
| GO:0043270 | positive regulation of ion transport | 4.02e-03 | 3.56e-02 | 3 | 49 | 99 | 15137 | 6.12% | 0.65% | 3.03% | 0.32% | mature\_B\_cell\_down | F2r, Stk39, Thy1 |
| GO:0045595 | regulation of cell differentiation | 4.05e-03 | 3.56e-02 | 9 | 49 | 990 | 15137 | 18.37% | 6.54% | 0.91% | 0.32% | mature\_B\_cell\_down | Cd27, Gata3, Lck, Lpin1, Notch1, Prkch, Thy1, Tnik, Zap70 |
| GO:0031399 | regulation of protein modification process | 4.13e-03 | 3.61e-02 | 8 | 49 | 809 | 15137 | 16.33% | 5.34% | 0.99% | 0.32% | mature\_B\_cell\_down | F2r, Gata3, Lck, Lpin1, Slc9a3r1, Thy1, Tnfrsf1a, Tnik |
| GO:0043412 | macromolecule modification | 4.19e-03 | 3.65e-02 | 13 | 49 | 1817 | 15137 | 26.53% | 12.00% | 0.72% | 0.32% | mature\_B\_cell\_down | Art2b, F2r, Fyn, Ifih1, Itk, Lck, Prkch, Prkcq, Stk39, Tnik, Txk, Zap70, Zdhhc15 |
| GO:0050793 | regulation of developmental process | 4.24e-03 | 3.67e-02 | 11 | 49 | 1393 | 15137 | 22.45% | 9.20% | 0.79% | 0.32% | mature\_B\_cell\_down | Cd27, Fyn, Gata3, Lck, Lpin1, Notch1, Prkch, Thy1, Tnfrsf1a, Tnik, Zap70 |
| GO:0045597 | positive regulation of cell differentiation | 4.52e-03 | 3.88e-02 | 6 | 49 | 484 | 15137 | 12.24% | 3.20% | 1.24% | 0.32% | mature\_B\_cell\_down | Cd27, Gata3, Lck, Notch1, Prkch, Zap70 |
| GO:0016055 | Wnt receptor signaling pathway | 5.01e-03 | 4.28e-02 | 4 | 49 | 214 | 15137 | 8.16% | 1.41% | 1.87% | 0.32% | mature\_B\_cell\_down | Gata3, Slc9a3r1, Tcf7, Tnik |
| GO:0000186 | activation of MAPKK activity | 5.08e-03 | 4.30e-02 | 2 | 49 | 33 | 15137 | 4.08% | 0.22% | 6.06% | 0.32% | mature\_B\_cell\_down | F2r, Tnik |
| GO:0060402 | calcium ion transport into cytosol | 5.08e-03 | 4.30e-02 | 2 | 49 | 33 | 15137 | 4.08% | 0.22% | 6.06% | 0.32% | mature\_B\_cell\_down | F2r, Lck |
| GO:0043269 | regulation of ion transport | 5.38e-03 | 4.48e-02 | 5 | 49 | 351 | 15137 | 10.20% | 2.32% | 1.42% | 0.32% | mature\_B\_cell\_down | F2r, Slc9a3r1, Stk39, Thy1, Tmem66 |
| GO:0031532 | actin cytoskeleton reorganization | 5.39e-03 | 4.48e-02 | 2 | 49 | 34 | 15137 | 4.08% | 0.22% | 5.88% | 0.32% | mature\_B\_cell\_down | Lpin1, Tnik |
| GO:0060401 | cytosolic calcium ion transport | 5.39e-03 | 4.48e-02 | 2 | 49 | 34 | 15137 | 4.08% | 0.22% | 5.88% | 0.32% | mature\_B\_cell\_down | F2r, Lck |
| GO:0051094 | positive regulation of developmental process | 5.58e-03 | 4.58e-02 | 7 | 49 | 672 | 15137 | 14.29% | 4.44% | 1.04% | 0.32% | mature\_B\_cell\_down | Cd27, Gata3, Lck, Notch1, Prkch, Tnfrsf1a, Zap70 |
| GO:0050848 | regulation of calcium-mediated signaling | 5.71e-03 | 4.58e-02 | 2 | 49 | 35 | 15137 | 4.08% | 0.23% | 5.71% | 0.32% | mature\_B\_cell\_down | Sla2, Zap70 |
| GO:0010517 | regulation of phospholipase activity | 5.71e-03 | 4.58e-02 | 2 | 49 | 35 | 15137 | 4.08% | 0.23% | 5.71% | 0.32% | mature\_B\_cell\_down | Itk, Txk |
| GO:0048585 | negative regulation of response to stimulus | 5.85e-03 | 4.58e-02 | 7 | 49 | 678 | 15137 | 14.29% | 4.48% | 1.03% | 0.32% | mature\_B\_cell\_down | Fyn, Gata3, Notch1, Prkcq, Sla2, Slc9a3r1, Thy1 |
| GO:0051247 | positive regulation of protein metabolic process | 5.99e-03 | 4.58e-02 | 7 | 49 | 681 | 15137 | 14.29% | 4.50% | 1.03% | 0.32% | mature\_B\_cell\_down | Cd27, F2r, Lck, Lpin1, Prkcq, Tnfrsf1a, Tnik |
| GO:0060193 | positive regulation of lipase activity | 6.03e-03 | 4.58e-02 | 2 | 49 | 36 | 15137 | 4.08% | 0.24% | 5.56% | 0.32% | mature\_B\_cell\_down | Itk, Txk |
| GO:0031325 | positive regulation of cellular metabolic process | 6.20e-03 | 4.58e-02 | 12 | 49 | 1680 | 15137 | 24.49% | 11.10% | 0.71% | 0.32% | mature\_B\_cell\_down | Cd27, F2r, Gata3, Hnrpll, Ipcef1, Lck, Lpin1, Notch1, Prkcq, Skap1, Tnfrsf1a, Tnik |
| GO:0050708 | regulation of protein secretion | 6.25e-03 | 4.58e-02 | 3 | 49 | 116 | 15137 | 6.12% | 0.77% | 2.59% | 0.32% | mature\_B\_cell\_down | F2r, Gata3, Prkcq |
| GO:0045682 | regulation of epidermis development | 6.36e-03 | 4.58e-02 | 2 | 49 | 37 | 15137 | 4.08% | 0.24% | 5.41% | 0.32% | mature\_B\_cell\_down | Notch1, Prkch |
| GO:0061289 | Wnt receptor signaling pathway involved in kidney development | 6.46e-03 | 4.58e-02 | 1 | 49 | 2 | 15137 | 2.04% | 0.01% | 50.00% | 0.32% | mature\_B\_cell\_down | Gata3 |
| GO:0043366 | beta selection | 6.46e-03 | 4.58e-02 | 1 | 49 | 2 | 15137 | 2.04% | 0.01% | 50.00% | 0.32% | mature\_B\_cell\_down | Zap70 |
| GO:0061290 | canonical Wnt receptor signaling pathway involved in metanephric kidney development | 6.46e-03 | 4.58e-02 | 1 | 49 | 2 | 15137 | 2.04% | 0.01% | 50.00% | 0.32% | mature\_B\_cell\_down | Gata3 |
| GO:0060995 | cell-cell signaling involved in kidney development | 6.46e-03 | 4.58e-02 | 1 | 49 | 2 | 15137 | 2.04% | 0.01% | 50.00% | 0.32% | mature\_B\_cell\_down | Gata3 |
| GO:0002248 | connective tissue replacement involved in inflammatory response wound healing | 6.46e-03 | 4.58e-02 | 1 | 49 | 2 | 15137 | 2.04% | 0.01% | 50.00% | 0.32% | mature\_B\_cell\_down | F2r |
| GO:0030950 | establishment or maintenance of actin cytoskeleton polarity | 6.46e-03 | 4.58e-02 | 1 | 49 | 2 | 15137 | 2.04% | 0.01% | 50.00% | 0.32% | mature\_B\_cell\_down | Nckap1 |
| GO:2000650 | negative regulation of sodium ion transmembrane transporter activity | 6.46e-03 | 4.58e-02 | 1 | 49 | 2 | 15137 | 2.04% | 0.01% | 50.00% | 0.32% | mature\_B\_cell\_down | Slc9a3r1 |
| GO:0032416 | negative regulation of sodium:hydrogen antiporter activity | 6.46e-03 | 4.58e-02 | 1 | 49 | 2 | 15137 | 2.04% | 0.01% | 50.00% | 0.32% | mature\_B\_cell\_down | Slc9a3r1 |
| GO:0035898 | parathyroid hormone secretion | 6.46e-03 | 4.58e-02 | 1 | 49 | 2 | 15137 | 2.04% | 0.01% | 50.00% | 0.32% | mature\_B\_cell\_down | Gata3 |
| GO:0060369 | positive regulation of Fc receptor mediated stimulatory signaling pathway | 6.46e-03 | 4.58e-02 | 1 | 49 | 2 | 15137 | 2.04% | 0.01% | 50.00% | 0.32% | mature\_B\_cell\_down | Cd226 |
| GO:0033126 | positive regulation of GTP catabolic process | 6.46e-03 | 4.58e-02 | 1 | 49 | 2 | 15137 | 2.04% | 0.01% | 50.00% | 0.32% | mature\_B\_cell\_down | Ipcef1 |
| GO:2000318 | positive regulation of T-helper 17 type immune response | 6.46e-03 | 4.58e-02 | 1 | 49 | 2 | 15137 | 2.04% | 0.01% | 50.00% | 0.32% | mature\_B\_cell\_down | Prkcq |
| GO:2000667 | positive regulation of interleukin-13 secretion | 6.46e-03 | 4.58e-02 | 1 | 49 | 2 | 15137 | 2.04% | 0.01% | 50.00% | 0.32% | mature\_B\_cell\_down | Gata3 |
| GO:2000664 | positive regulation of interleukin-5 secretion | 6.46e-03 | 4.58e-02 | 1 | 49 | 2 | 15137 | 2.04% | 0.01% | 50.00% | 0.32% | mature\_B\_cell\_down | Gata3 |
| GO:0030813 | positive regulation of nucleotide catabolic process | 6.46e-03 | 4.58e-02 | 1 | 49 | 2 | 15137 | 2.04% | 0.01% | 50.00% | 0.32% | mature\_B\_cell\_down | Ipcef1 |
| GO:0033123 | positive regulation of purine nucleotide catabolic process | 6.46e-03 | 4.58e-02 | 1 | 49 | 2 | 15137 | 2.04% | 0.01% | 50.00% | 0.32% | mature\_B\_cell\_down | Ipcef1 |
| GO:0002572 | pro-T cell differentiation | 6.46e-03 | 4.58e-02 | 1 | 49 | 2 | 15137 | 2.04% | 0.01% | 50.00% | 0.32% | mature\_B\_cell\_down | Gata3 |
| GO:2000683 | regulation of cellular response to X-ray | 6.46e-03 | 4.58e-02 | 1 | 49 | 2 | 15137 | 2.04% | 0.01% | 50.00% | 0.32% | mature\_B\_cell\_down | Gata3 |
| GO:2000665 | regulation of interleukin-13 secretion | 6.46e-03 | 4.58e-02 | 1 | 49 | 2 | 15137 | 2.04% | 0.01% | 50.00% | 0.32% | mature\_B\_cell\_down | Gata3 |
| GO:2000662 | regulation of interleukin-5 secretion | 6.46e-03 | 4.58e-02 | 1 | 49 | 2 | 15137 | 2.04% | 0.01% | 50.00% | 0.32% | mature\_B\_cell\_down | Gata3 |
| GO:1900133 | regulation of renin secretion into blood stream | 6.46e-03 | 4.58e-02 | 1 | 49 | 2 | 15137 | 2.04% | 0.01% | 50.00% | 0.32% | mature\_B\_cell\_down | F2r |
| GO:0003096 | renal sodium ion transport | 6.46e-03 | 4.58e-02 | 1 | 49 | 2 | 15137 | 2.04% | 0.01% | 50.00% | 0.32% | mature\_B\_cell\_down | Slc9a3r1 |
| GO:0018193 | peptidyl-amino acid modification | 6.48e-03 | 4.58e-02 | 5 | 49 | 367 | 15137 | 10.20% | 2.42% | 1.36% | 0.32% | mature\_B\_cell\_down | Art2b, F2r, Fyn, Lck, Zap70 |
| GO:2000146 | negative regulation of cell motility | 6.55e-03 | 4.61e-02 | 3 | 49 | 118 | 15137 | 6.12% | 0.78% | 2.54% | 0.32% | mature\_B\_cell\_down | Gata3, Slc9a3r1, Thy1 |
| GO:0051047 | positive regulation of secretion | 6.84e-03 | 4.79e-02 | 4 | 49 | 234 | 15137 | 8.16% | 1.55% | 1.71% | 0.32% | mature\_B\_cell\_down | F2r, Gata3, Prkcq, Sytl2 |
| GO:0042531 | positive regulation of tyrosine phosphorylation of STAT protein | 7.05e-03 | 4.91e-02 | 2 | 49 | 39 | 15137 | 4.08% | 0.26% | 5.13% | 0.32% | mature\_B\_cell\_down | Lck, Tnfrsf1a |
| GO:2000021 | regulation of ion homeostasis | 7.19e-03 | 4.99e-02 | 3 | 49 | 122 | 15137 | 6.12% | 0.81% | 2.46% | 0.32% | mature\_B\_cell\_down | F2r, Lck, Thy1 |
| GO:0051271 | negative regulation of cellular component movement | 7.35e-03 | 5.08e-02 | 3 | 49 | 123 | 15137 | 6.12% | 0.81% | 2.44% | 0.32% | mature\_B\_cell\_down | Gata3, Slc9a3r1, Thy1 |
| GO:0032663 | regulation of interleukin-2 production | 7.40e-03 | 5.09e-02 | 2 | 49 | 40 | 15137 | 4.08% | 0.26% | 5.00% | 0.32% | mature\_B\_cell\_down | Gata3, Prkcq |
| GO:0071695 | anatomical structure maturation | 7.77e-03 | 5.23e-02 | 2 | 49 | 41 | 15137 | 4.08% | 0.27% | 4.88% | 0.32% | mature\_B\_cell\_down | Gata3, Notch1 |
| GO:0014015 | positive regulation of gliogenesis | 7.77e-03 | 5.23e-02 | 2 | 49 | 41 | 15137 | 4.08% | 0.27% | 4.88% | 0.32% | mature\_B\_cell\_down | Notch1, Prkch |
| GO:0046637 | regulation of alpha-beta T cell differentiation | 7.77e-03 | 5.23e-02 | 2 | 49 | 41 | 15137 | 4.08% | 0.27% | 4.88% | 0.32% | mature\_B\_cell\_down | Gata3, Zap70 |
| GO:0010522 | regulation of calcium ion transport into cytosol | 7.77e-03 | 5.23e-02 | 2 | 49 | 41 | 15137 | 4.08% | 0.27% | 4.88% | 0.32% | mature\_B\_cell\_down | F2r, Thy1 |
| GO:2000026 | regulation of multicellular organismal development | 7.78e-03 | 5.23e-02 | 9 | 49 | 1095 | 15137 | 18.37% | 7.23% | 0.82% | 0.32% | mature\_B\_cell\_down | Cd27, Gata3, Lck, Notch1, Prkch, Thy1, Tnfrsf1a, Tnik, Zap70 |
| GO:0051649 | establishment of localization in cell | 7.93e-03 | 5.31e-02 | 8 | 49 | 903 | 15137 | 16.33% | 5.97% | 0.89% | 0.32% | mature\_B\_cell\_down | Cd27, F2r, Gata3, Lat, Lcp2, Notch1, Sytl2, Vps26b |
| GO:0022603 | regulation of anatomical structure morphogenesis | 7.97e-03 | 5.31e-02 | 6 | 49 | 545 | 15137 | 12.24% | 3.60% | 1.10% | 0.32% | mature\_B\_cell\_down | Fyn, Gata3, Notch1, Thy1, Tnfrsf1a, Tnik |
| GO:0007154 | cell communication | 8.16e-03 | 5.41e-02 | 23 | 49 | 4503 | 15137 | 46.94% | 29.75% | 0.51% | 0.32% | mature\_B\_cell\_down | Apol7b, Cd247, Cd3g, Ctsd, F2r, Fyn, Gata3, Itk, Lat, Lck, Notch1, Prkch, Prkcq, Rab37, Skap1, Slc9a3r1, Tcf7, Thy1, Tnfrsf1a, Tnik, Txk, Zap70, Zdhhc15 |
| GO:0007166 | cell surface receptor signaling pathway | 8.23e-03 | 5.44e-02 | 17 | 49 | 2927 | 15137 | 34.69% | 19.34% | 0.58% | 0.32% | mature\_B\_cell\_down | Cd247, Cd3g, F2r, Fyn, Gata3, Itk, Lat, Lck, Notch1, Skap1, Slc9a3r1, Tcf7, Thy1, Tnfrsf1a, Tnik, Txk, Zap70 |
| GO:0080090 | regulation of primary metabolic process | 8.95e-03 | 5.83e-02 | 20 | 49 | 3724 | 15137 | 40.82% | 24.60% | 0.54% | 0.32% | mature\_B\_cell\_down | Cd27, F2r, Fyn, Gata3, Hnrpll, Ipcef1, Lck, Lpin1, Notch1, Prkch, Prkcq, Skap1, Sla2, Slc9a3r1, Tbc1d2b, Tcf7, Thy1, Tnfrsf1a, Tnik, Txk |
| GO:0051223 | regulation of protein transport | 9.08e-03 | 5.83e-02 | 4 | 49 | 254 | 15137 | 8.16% | 1.68% | 1.57% | 0.32% | mature\_B\_cell\_down | F2r, Gata3, Prkcq, Tnfrsf1a |
| GO:0040013 | negative regulation of locomotion | 9.10e-03 | 5.83e-02 | 3 | 49 | 133 | 15137 | 6.12% | 0.88% | 2.26% | 0.32% | mature\_B\_cell\_down | Gata3, Slc9a3r1, Thy1 |
| GO:0051641 | cellular localization | 9.22e-03 | 5.83e-02 | 9 | 49 | 1125 | 15137 | 18.37% | 7.43% | 0.80% | 0.32% | mature\_B\_cell\_down | Cd27, F2r, Gata3, Lat, Lcp2, Notch1, Slc9a3r1, Sytl2, Vps26b |
| GO:0006940 | regulation of smooth muscle contraction | 9.30e-03 | 5.83e-02 | 2 | 49 | 45 | 15137 | 4.08% | 0.30% | 4.44% | 0.32% | mature\_B\_cell\_down | F2r, Lck |
| GO:0045064 | T-helper 2 cell differentiation | 9.68e-03 | 5.83e-02 | 1 | 49 | 3 | 15137 | 2.04% | 0.02% | 33.33% | 0.32% | mature\_B\_cell\_down | Gata3 |
| GO:0007529 | establishment of synaptic specificity at neuromuscular junction | 9.68e-03 | 5.83e-02 | 1 | 49 | 3 | 15137 | 2.04% | 0.02% | 33.33% | 0.32% | mature\_B\_cell\_down | F2r |
| GO:0034635 | glutathione transport | 9.68e-03 | 5.83e-02 | 1 | 49 | 3 | 15137 | 2.04% | 0.02% | 33.33% | 0.32% | mature\_B\_cell\_down | Slc9a3r1 |
| GO:0072643 | interferon-gamma secretion | 9.68e-03 | 5.83e-02 | 1 | 49 | 3 | 15137 | 2.04% | 0.02% | 33.33% | 0.32% | mature\_B\_cell\_down | Gata3 |
| GO:2000811 | negative regulation of anoikis | 9.68e-03 | 5.83e-02 | 1 | 49 | 3 | 15137 | 2.04% | 0.02% | 33.33% | 0.32% | mature\_B\_cell\_down | Notch1 |
| GO:0032235 | negative regulation of calcium ion transport via store-operated calcium channel activity | 9.68e-03 | 5.83e-02 | 1 | 49 | 3 | 15137 | 2.04% | 0.02% | 33.33% | 0.32% | mature\_B\_cell\_down | Tmem66 |
| GO:0050849 | negative regulation of calcium-mediated signaling | 9.68e-03 | 5.83e-02 | 1 | 49 | 3 | 15137 | 2.04% | 0.02% | 33.33% | 0.32% | mature\_B\_cell\_down | Sla2 |
| GO:2000974 | negative regulation of pro-B cell differentiation | 9.68e-03 | 5.83e-02 | 1 | 49 | 3 | 15137 | 2.04% | 0.02% | 33.33% | 0.32% | mature\_B\_cell\_down | Notch1 |
| GO:0050861 | positive regulation of B cell receptor signaling pathway | 9.68e-03 | 5.83e-02 | 1 | 49 | 3 | 15137 | 2.04% | 0.02% | 33.33% | 0.32% | mature\_B\_cell\_down | Prkch |
| GO:2000611 | positive regulation of thyroid hormone generation | 9.68e-03 | 5.83e-02 | 1 | 49 | 3 | 15137 | 2.04% | 0.02% | 33.33% | 0.32% | mature\_B\_cell\_down | Gata3 |
| GO:0036003 | positive regulation of transcription from RNA polymerase II promoter in response to stress | 9.68e-03 | 5.83e-02 | 1 | 49 | 3 | 15137 | 2.04% | 0.02% | 33.33% | 0.32% | mature\_B\_cell\_down | Notch1 |
| GO:0007221 | positive regulation of transcription of Notch receptor target | 9.68e-03 | 5.83e-02 | 1 | 49 | 3 | 15137 | 2.04% | 0.02% | 33.33% | 0.32% | mature\_B\_cell\_down | Notch1 |
| GO:2000973 | regulation of pro-B cell differentiation | 9.68e-03 | 5.83e-02 | 1 | 49 | 3 | 15137 | 2.04% | 0.02% | 33.33% | 0.32% | mature\_B\_cell\_down | Notch1 |
| GO:2001256 | regulation of store-operated calcium entry | 9.68e-03 | 5.83e-02 | 1 | 49 | 3 | 15137 | 2.04% | 0.02% | 33.33% | 0.32% | mature\_B\_cell\_down | Tmem66 |
| GO:2000609 | regulation of thyroid hormone generation | 9.68e-03 | 5.83e-02 | 1 | 49 | 3 | 15137 | 2.04% | 0.02% | 33.33% | 0.32% | mature\_B\_cell\_down | Gata3 |
| GO:0042939 | tripeptide transport | 9.68e-03 | 5.83e-02 | 1 | 49 | 3 | 15137 | 2.04% | 0.02% | 33.33% | 0.32% | mature\_B\_cell\_down | Slc9a3r1 |
| GO:0007354 | zygotic determination of anterior/posterior axis, embryo | 9.68e-03 | 5.83e-02 | 1 | 49 | 3 | 15137 | 2.04% | 0.02% | 33.33% | 0.32% | mature\_B\_cell\_down | Nckap1 |
| GO:0014066 | regulation of phosphatidylinositol 3-kinase cascade | 9.70e-03 | 5.83e-02 | 2 | 49 | 46 | 15137 | 4.08% | 0.30% | 4.35% | 0.32% | mature\_B\_cell\_down | F2r, Slc9a3r1 |
| GO:0031323 | regulation of cellular metabolic process | 9.99e-03 | 5.97e-02 | 20 | 49 | 3760 | 15137 | 40.82% | 24.84% | 0.53% | 0.32% | mature\_B\_cell\_down | Cd27, F2r, Gata3, Hnrpll, Ipcef1, Lck, Lpin1, Notch1, Prkch, Prkcq, Skap1, Sla2, Slc9a3r1, Sytl2, Tbc1d2b, Tcf7, Thy1, Tnfrsf1a, Tnik, Txk |
| GO:0009893 | positive regulation of metabolic process | 1.01e-02 | 5.98e-02 | 12 | 49 | 1788 | 15137 | 24.49% | 11.81% | 0.67% | 0.32% | mature\_B\_cell\_down | Cd27, F2r, Gata3, Hnrpll, Ipcef1, Lck, Lpin1, Notch1, Prkcq, Skap1, Tnfrsf1a, Tnik |
| GO:0007200 | phospholipase C-activating G-protein coupled receptor signaling pathway | 1.01e-02 | 5.98e-02 | 2 | 49 | 47 | 15137 | 4.08% | 0.31% | 4.26% | 0.32% | mature\_B\_cell\_down | F2r, Slc9a3r1 |
| GO:0042509 | regulation of tyrosine phosphorylation of STAT protein | 1.01e-02 | 5.98e-02 | 2 | 49 | 47 | 15137 | 4.08% | 0.31% | 4.26% | 0.32% | mature\_B\_cell\_down | Lck, Tnfrsf1a |
| GO:0030036 | actin cytoskeleton organization | 1.05e-02 | 6.15e-02 | 4 | 49 | 265 | 15137 | 8.16% | 1.75% | 1.51% | 0.32% | mature\_B\_cell\_down | Lpin1, Nckap1, Slc9a3r1, Tnik |
| GO:0043271 | negative regulation of ion transport | 1.05e-02 | 6.15e-02 | 2 | 49 | 48 | 15137 | 4.08% | 0.32% | 4.17% | 0.32% | mature\_B\_cell\_down | Slc9a3r1, Tmem66 |
| GO:0046635 | positive regulation of alpha-beta T cell activation | 1.05e-02 | 6.15e-02 | 2 | 49 | 48 | 15137 | 4.08% | 0.32% | 4.17% | 0.32% | mature\_B\_cell\_down | Prkcq, Zap70 |
| GO:0043066 | negative regulation of apoptotic process | 1.06e-02 | 6.15e-02 | 6 | 49 | 579 | 15137 | 12.24% | 3.83% | 1.04% | 0.32% | mature\_B\_cell\_down | Cd27, F2r, Gata3, Notch1, Prkch, Prkcq |
| GO:0021915 | neural tube development | 1.07e-02 | 6.18e-02 | 3 | 49 | 141 | 15137 | 6.12% | 0.93% | 2.13% | 0.32% | mature\_B\_cell\_down | Nckap1, Notch1, Tcf7 |
| GO:0030030 | cell projection organization | 1.07e-02 | 6.20e-02 | 6 | 49 | 581 | 15137 | 12.24% | 3.84% | 1.03% | 0.32% | mature\_B\_cell\_down | Fyn, Gata3, Lpin1, Nckap1, Notch1, Slc9a3r1 |
| GO:0043069 | negative regulation of programmed cell death | 1.10e-02 | 6.32e-02 | 6 | 49 | 584 | 15137 | 12.24% | 3.86% | 1.03% | 0.32% | mature\_B\_cell\_down | Cd27, F2r, Gata3, Notch1, Prkch, Prkcq |
| GO:0022604 | regulation of cell morphogenesis | 1.12e-02 | 6.39e-02 | 4 | 49 | 270 | 15137 | 8.16% | 1.78% | 1.48% | 0.32% | mature\_B\_cell\_down | Fyn, Notch1, Thy1, Tnik |
| GO:0031347 | regulation of defense response | 1.12e-02 | 6.39e-02 | 4 | 49 | 270 | 15137 | 8.16% | 1.78% | 1.48% | 0.32% | mature\_B\_cell\_down | Cd226, Gata3, Tnfrsf1a, Txk |
| GO:0007165 | signal transduction | 1.18e-02 | 6.43e-02 | 21 | 49 | 4085 | 15137 | 42.86% | 26.99% | 0.51% | 0.32% | mature\_B\_cell\_down | Apol7b, Cd247, Cd3g, F2r, Fyn, Gata3, Itk, Lat, Lck, Notch1, Prkch, Prkcq, Rab37, Skap1, Slc9a3r1, Tcf7, Thy1, Tnfrsf1a, Tnik, Txk, Zap70 |
| GO:0033077 | T cell differentiation in thymus | 1.18e-02 | 6.43e-02 | 2 | 49 | 51 | 15137 | 4.08% | 0.34% | 3.92% | 0.32% | mature\_B\_cell\_down | Gata3, Zap70 |
| GO:0046651 | lymphocyte proliferation | 1.18e-02 | 6.43e-02 | 2 | 49 | 51 | 15137 | 4.08% | 0.34% | 3.92% | 0.32% | mature\_B\_cell\_down | Cd27, Fyn |
| GO:0030193 | regulation of blood coagulation | 1.18e-02 | 6.43e-02 | 2 | 49 | 51 | 15137 | 4.08% | 0.34% | 3.92% | 0.32% | mature\_B\_cell\_down | F2r, Prkcq |
| GO:1900046 | regulation of hemostasis | 1.18e-02 | 6.43e-02 | 2 | 49 | 51 | 15137 | 4.08% | 0.34% | 3.92% | 0.32% | mature\_B\_cell\_down | F2r, Prkcq |
| GO:0070201 | regulation of establishment of protein localization | 1.22e-02 | 6.43e-02 | 4 | 49 | 277 | 15137 | 8.16% | 1.83% | 1.44% | 0.32% | mature\_B\_cell\_down | F2r, Gata3, Prkcq, Tnfrsf1a |
| GO:0032943 | mononuclear cell proliferation | 1.23e-02 | 6.43e-02 | 2 | 49 | 52 | 15137 | 4.08% | 0.34% | 3.85% | 0.32% | mature\_B\_cell\_down | Cd27, Fyn |
| GO:0051051 | negative regulation of transport | 1.23e-02 | 6.43e-02 | 4 | 49 | 278 | 15137 | 8.16% | 1.84% | 1.44% | 0.32% | mature\_B\_cell\_down | F2r, Notch1, Slc9a3r1, Tmem66 |
| GO:0070661 | leukocyte proliferation | 1.27e-02 | 6.43e-02 | 2 | 49 | 53 | 15137 | 4.08% | 0.35% | 3.77% | 0.32% | mature\_B\_cell\_down | Cd27, Fyn |
| GO:0050715 | positive regulation of cytokine secretion | 1.27e-02 | 6.43e-02 | 2 | 49 | 53 | 15137 | 4.08% | 0.35% | 3.77% | 0.32% | mature\_B\_cell\_down | F2r, Gata3 |
| GO:0023052 | signaling | 1.28e-02 | 6.43e-02 | 22 | 49 | 4387 | 15137 | 44.90% | 28.98% | 0.50% | 0.32% | mature\_B\_cell\_down | Apol7b, Cd247, Cd3g, F2r, Fyn, Gata3, Itk, Lat, Lck, Notch1, Prkch, Prkcq, Rab37, Skap1, Slc9a3r1, Tcf7, Thy1, Tnfrsf1a, Tnik, Txk, Zap70, Zdhhc15 |
| GO:0003219 | cardiac right ventricle formation | 1.29e-02 | 6.43e-02 | 1 | 49 | 4 | 15137 | 2.04% | 0.03% | 25.00% | 0.32% | mature\_B\_cell\_down | Notch1 |
| GO:0007386 | compartment pattern specification | 1.29e-02 | 6.43e-02 | 1 | 49 | 4 | 15137 | 2.04% | 0.03% | 25.00% | 0.32% | mature\_B\_cell\_down | Notch1 |
| GO:0030538 | embryonic genitalia morphogenesis | 1.29e-02 | 6.43e-02 | 1 | 49 | 4 | 15137 | 2.04% | 0.03% | 25.00% | 0.32% | mature\_B\_cell\_down | Tcf7 |
| GO:0048619 | embryonic hindgut morphogenesis | 1.29e-02 | 6.43e-02 | 1 | 49 | 4 | 15137 | 2.04% | 0.03% | 25.00% | 0.32% | mature\_B\_cell\_down | Tcf7 |
| GO:0008078 | mesodermal cell migration | 1.29e-02 | 6.43e-02 | 1 | 49 | 4 | 15137 | 2.04% | 0.03% | 25.00% | 0.32% | mature\_B\_cell\_down | Nckap1 |
| GO:1901020 | negative regulation of calcium ion transmembrane transporter activity | 1.29e-02 | 6.43e-02 | 1 | 49 | 4 | 15137 | 2.04% | 0.03% | 25.00% | 0.32% | mature\_B\_cell\_down | Tmem66 |
| GO:0003105 | negative regulation of glomerular filtration | 1.29e-02 | 6.43e-02 | 1 | 49 | 4 | 15137 | 2.04% | 0.03% | 25.00% | 0.32% | mature\_B\_cell\_down | F2r |
| GO:0046533 | negative regulation of photoreceptor cell differentiation | 1.29e-02 | 6.43e-02 | 1 | 49 | 4 | 15137 | 2.04% | 0.03% | 25.00% | 0.32% | mature\_B\_cell\_down | Notch1 |
| GO:0048799 | organ maturation | 1.29e-02 | 6.43e-02 | 1 | 49 | 4 | 15137 | 2.04% | 0.03% | 25.00% | 0.32% | mature\_B\_cell\_down | Gata3 |
| GO:0060335 | positive regulation of interferon-gamma-mediated signaling pathway | 1.29e-02 | 6.43e-02 | 1 | 49 | 4 | 15137 | 2.04% | 0.03% | 25.00% | 0.32% | mature\_B\_cell\_down | Txk |
| GO:0032754 | positive regulation of interleukin-5 production | 1.29e-02 | 6.43e-02 | 1 | 49 | 4 | 15137 | 2.04% | 0.03% | 25.00% | 0.32% | mature\_B\_cell\_down | Gata3 |
| GO:2000778 | positive regulation of interleukin-6 secretion | 1.29e-02 | 6.43e-02 | 1 | 49 | 4 | 15137 | 2.04% | 0.03% | 25.00% | 0.32% | mature\_B\_cell\_down | F2r |
| GO:2000484 | positive regulation of interleukin-8 secretion | 1.29e-02 | 6.43e-02 | 1 | 49 | 4 | 15137 | 2.04% | 0.03% | 25.00% | 0.32% | mature\_B\_cell\_down | F2r |
| GO:0070257 | positive regulation of mucus secretion | 1.29e-02 | 6.43e-02 | 1 | 49 | 4 | 15137 | 2.04% | 0.03% | 25.00% | 0.32% | mature\_B\_cell\_down | Sytl2 |
| GO:0060332 | positive regulation of response to interferon-gamma | 1.29e-02 | 6.43e-02 | 1 | 49 | 4 | 15137 | 2.04% | 0.03% | 25.00% | 0.32% | mature\_B\_cell\_down | Txk |
| GO:0072107 | positive regulation of ureteric bud formation | 1.29e-02 | 6.43e-02 | 1 | 49 | 4 | 15137 | 2.04% | 0.03% | 25.00% | 0.32% | mature\_B\_cell\_down | Gata3 |
| GO:0060368 | regulation of Fc receptor mediated stimulatory signaling pathway | 1.29e-02 | 6.43e-02 | 1 | 49 | 4 | 15137 | 2.04% | 0.03% | 25.00% | 0.32% | mature\_B\_cell\_down | Cd226 |
| GO:2000316 | regulation of T-helper 17 type immune response | 1.29e-02 | 6.43e-02 | 1 | 49 | 4 | 15137 | 2.04% | 0.03% | 25.00% | 0.32% | mature\_B\_cell\_down | Prkcq |
| GO:2000114 | regulation of establishment of cell polarity | 1.29e-02 | 6.43e-02 | 1 | 49 | 4 | 15137 | 2.04% | 0.03% | 25.00% | 0.32% | mature\_B\_cell\_down | Gata3 |
| GO:2000482 | regulation of interleukin-8 secretion | 1.29e-02 | 6.43e-02 | 1 | 49 | 4 | 15137 | 2.04% | 0.03% | 25.00% | 0.32% | mature\_B\_cell\_down | F2r |
| GO:0032415 | regulation of sodium:hydrogen antiporter activity | 1.29e-02 | 6.43e-02 | 1 | 49 | 4 | 15137 | 2.04% | 0.03% | 25.00% | 0.32% | mature\_B\_cell\_down | Slc9a3r1 |
| GO:2000810 | regulation of tight junction assembly | 1.29e-02 | 6.43e-02 | 1 | 49 | 4 | 15137 | 2.04% | 0.03% | 25.00% | 0.32% | mature\_B\_cell\_down | Prkch |
| GO:0061418 | regulation of transcription from RNA polymerase II promoter in response to hypoxia | 1.29e-02 | 6.43e-02 | 1 | 49 | 4 | 15137 | 2.04% | 0.03% | 25.00% | 0.32% | mature\_B\_cell\_down | Notch1 |
| GO:0072106 | regulation of ureteric bud formation | 1.29e-02 | 6.43e-02 | 1 | 49 | 4 | 15137 | 2.04% | 0.03% | 25.00% | 0.32% | mature\_B\_cell\_down | Gata3 |
| GO:0060528 | secretory columnal luminar epithelial cell differentiation involved in prostate glandular acinus development | 1.29e-02 | 6.43e-02 | 1 | 49 | 4 | 15137 | 2.04% | 0.03% | 25.00% | 0.32% | mature\_B\_cell\_down | Notch1 |
| GO:0042092 | type 2 immune response | 1.29e-02 | 6.43e-02 | 1 | 49 | 4 | 15137 | 2.04% | 0.03% | 25.00% | 0.32% | mature\_B\_cell\_down | Gata3 |
| GO:0035799 | ureter maturation | 1.29e-02 | 6.43e-02 | 1 | 49 | 4 | 15137 | 2.04% | 0.03% | 25.00% | 0.32% | mature\_B\_cell\_down | Gata3 |
| GO:0051924 | regulation of calcium ion transport | 1.31e-02 | 6.50e-02 | 3 | 49 | 152 | 15137 | 6.12% | 1.00% | 1.97% | 0.32% | mature\_B\_cell\_down | F2r, Thy1, Tmem66 |
| GO:0030029 | actin filament-based process | 1.33e-02 | 6.58e-02 | 4 | 49 | 284 | 15137 | 8.16% | 1.88% | 1.41% | 0.32% | mature\_B\_cell\_down | Lpin1, Nckap1, Slc9a3r1, Tnik |
| GO:0009612 | response to mechanical stimulus | 1.37e-02 | 6.76e-02 | 2 | 49 | 55 | 15137 | 4.08% | 0.36% | 3.64% | 0.32% | mature\_B\_cell\_down | Fyn, Lck |
| GO:0060548 | negative regulation of cell death | 1.37e-02 | 6.76e-02 | 6 | 49 | 613 | 15137 | 12.24% | 4.05% | 0.98% | 0.32% | mature\_B\_cell\_down | Cd27, F2r, Gata3, Notch1, Prkch, Prkcq |
| GO:0009611 | response to wounding | 1.45e-02 | 7.14e-02 | 5 | 49 | 448 | 15137 | 10.20% | 2.96% | 1.12% | 0.32% | mature\_B\_cell\_down | F2r, Lat, Notch1, Prkcq, Tnfrsf1a |
| GO:0050818 | regulation of coagulation | 1.46e-02 | 7.16e-02 | 2 | 49 | 57 | 15137 | 4.08% | 0.38% | 3.51% | 0.32% | mature\_B\_cell\_down | F2r, Prkcq |
| GO:0043281 | regulation of cysteine-type endopeptidase activity involved in apoptotic process | 1.47e-02 | 7.18e-02 | 3 | 49 | 159 | 15137 | 6.12% | 1.05% | 1.89% | 0.32% | mature\_B\_cell\_down | Cd27, F2r, Lck |
| GO:0010604 | positive regulation of macromolecule metabolic process | 1.48e-02 | 7.22e-02 | 11 | 49 | 1653 | 15137 | 22.45% | 10.92% | 0.67% | 0.32% | mature\_B\_cell\_down | Cd27, F2r, Gata3, Hnrpll, Lck, Lpin1, Notch1, Prkcq, Skap1, Tnfrsf1a, Tnik |
| GO:0021700 | developmental maturation | 1.50e-02 | 7.22e-02 | 3 | 49 | 160 | 15137 | 6.12% | 1.06% | 1.88% | 0.32% | mature\_B\_cell\_down | Gata3, Notch1, Zdhhc15 |
| GO:0031056 | regulation of histone modification | 1.51e-02 | 7.22e-02 | 2 | 49 | 58 | 15137 | 4.08% | 0.38% | 3.45% | 0.32% | mature\_B\_cell\_down | Gata3, Lpin1 |
| GO:0003014 | renal system process | 1.51e-02 | 7.22e-02 | 2 | 49 | 58 | 15137 | 4.08% | 0.38% | 3.45% | 0.32% | mature\_B\_cell\_down | F2r, Slc9a3r1 |
| GO:0019220 | regulation of phosphate metabolic process | 1.53e-02 | 7.22e-02 | 7 | 49 | 816 | 15137 | 14.29% | 5.39% | 0.86% | 0.32% | mature\_B\_cell\_down | F2r, Lck, Slc9a3r1, Sytl2, Thy1, Tnfrsf1a, Tnik |
| GO:0051174 | regulation of phosphorus metabolic process | 1.53e-02 | 7.22e-02 | 7 | 49 | 816 | 15137 | 14.29% | 5.39% | 0.86% | 0.32% | mature\_B\_cell\_down | F2r, Lck, Slc9a3r1, Sytl2, Thy1, Tnfrsf1a, Tnik |
| GO:2000116 | regulation of cysteine-type endopeptidase activity | 1.55e-02 | 7.22e-02 | 3 | 49 | 162 | 15137 | 6.12% | 1.07% | 1.85% | 0.32% | mature\_B\_cell\_down | Cd27, F2r, Lck |
| GO:0042108 | positive regulation of cytokine biosynthetic process | 1.56e-02 | 7.22e-02 | 2 | 49 | 59 | 15137 | 4.08% | 0.39% | 3.39% | 0.32% | mature\_B\_cell\_down | Cd27, Prkcq |
| GO:0032268 | regulation of cellular protein metabolic process | 1.60e-02 | 7.22e-02 | 8 | 49 | 1022 | 15137 | 16.33% | 6.75% | 0.78% | 0.32% | mature\_B\_cell\_down | F2r, Gata3, Lck, Lpin1, Slc9a3r1, Thy1, Tnfrsf1a, Tnik |
| GO:0033153 | T cell receptor V(D)J recombination | 1.61e-02 | 7.22e-02 | 1 | 49 | 5 | 15137 | 2.04% | 0.03% | 20.00% | 0.32% | mature\_B\_cell\_down | Tcf7 |
| GO:0030952 | establishment or maintenance of cytoskeleton polarity | 1.61e-02 | 7.22e-02 | 1 | 49 | 5 | 15137 | 2.04% | 0.03% | 20.00% | 0.32% | mature\_B\_cell\_down | Nckap1 |
| GO:0030033 | microvillus assembly | 1.61e-02 | 7.22e-02 | 1 | 49 | 5 | 15137 | 2.04% | 0.03% | 20.00% | 0.32% | mature\_B\_cell\_down | Slc9a3r1 |
| GO:0032528 | microvillus organization | 1.61e-02 | 7.22e-02 | 1 | 49 | 5 | 15137 | 2.04% | 0.03% | 20.00% | 0.32% | mature\_B\_cell\_down | Slc9a3r1 |
| GO:2001240 | negative regulation of extrinsic apoptotic signaling pathway in absence of ligand | 1.61e-02 | 7.22e-02 | 1 | 49 | 5 | 15137 | 2.04% | 0.03% | 20.00% | 0.32% | mature\_B\_cell\_down | Fyn |
| GO:0010766 | negative regulation of sodium ion transport | 1.61e-02 | 7.22e-02 | 1 | 49 | 5 | 15137 | 2.04% | 0.03% | 20.00% | 0.32% | mature\_B\_cell\_down | Slc9a3r1 |
| GO:2000737 | negative regulation of stem cell differentiation | 1.61e-02 | 7.22e-02 | 1 | 49 | 5 | 15137 | 2.04% | 0.03% | 20.00% | 0.32% | mature\_B\_cell\_down | Notch1 |
| GO:0072179 | nephric duct formation | 1.61e-02 | 7.22e-02 | 1 | 49 | 5 | 15137 | 2.04% | 0.03% | 20.00% | 0.32% | mature\_B\_cell\_down | Gata3 |
| GO:0042421 | norepinephrine biosynthetic process | 1.61e-02 | 7.22e-02 | 1 | 49 | 5 | 15137 | 2.04% | 0.03% | 20.00% | 0.32% | mature\_B\_cell\_down | Gata3 |
| GO:0002051 | osteoblast fate commitment | 1.61e-02 | 7.22e-02 | 1 | 49 | 5 | 15137 | 2.04% | 0.03% | 20.00% | 0.32% | mature\_B\_cell\_down | Notch1 |
| GO:0045764 | positive regulation of cellular amino acid metabolic process | 1.61e-02 | 7.22e-02 | 1 | 49 | 5 | 15137 | 2.04% | 0.03% | 20.00% | 0.32% | mature\_B\_cell\_down | Gata3 |
| GO:0032736 | positive regulation of interleukin-13 production | 1.61e-02 | 7.22e-02 | 1 | 49 | 5 | 15137 | 2.04% | 0.03% | 20.00% | 0.32% | mature\_B\_cell\_down | Gata3 |
| GO:0046532 | regulation of photoreceptor cell differentiation | 1.61e-02 | 7.22e-02 | 1 | 49 | 5 | 15137 | 2.04% | 0.03% | 20.00% | 0.32% | mature\_B\_cell\_down | Notch1 |
| GO:0002568 | somatic diversification of T cell receptor genes | 1.61e-02 | 7.22e-02 | 1 | 49 | 5 | 15137 | 2.04% | 0.03% | 20.00% | 0.32% | mature\_B\_cell\_down | Tcf7 |
| GO:0002681 | somatic recombination of T cell receptor gene segments | 1.61e-02 | 7.22e-02 | 1 | 49 | 5 | 15137 | 2.04% | 0.03% | 20.00% | 0.32% | mature\_B\_cell\_down | Tcf7 |
| GO:0060676 | ureteric bud formation | 1.61e-02 | 7.22e-02 | 1 | 49 | 5 | 15137 | 2.04% | 0.03% | 20.00% | 0.32% | mature\_B\_cell\_down | Gata3 |
| GO:0002246 | wound healing involved in inflammatory response | 1.61e-02 | 7.22e-02 | 1 | 49 | 5 | 15137 | 2.04% | 0.03% | 20.00% | 0.32% | mature\_B\_cell\_down | F2r |
| GO:0003208 | cardiac ventricle morphogenesis | 1.61e-02 | 7.22e-02 | 2 | 49 | 60 | 15137 | 4.08% | 0.40% | 3.33% | 0.32% | mature\_B\_cell\_down | Gata3, Notch1 |
| GO:0007492 | endoderm development | 1.61e-02 | 7.22e-02 | 2 | 49 | 60 | 15137 | 4.08% | 0.40% | 3.33% | 0.32% | mature\_B\_cell\_down | Nckap1, Notch1 |
| GO:0002824 | positive regulation of adaptive immune response based on somatic recombination of immune receptors built from immunoglobulin superfamily domains | 1.66e-02 | 7.43e-02 | 2 | 49 | 61 | 15137 | 4.08% | 0.40% | 3.28% | 0.32% | mature\_B\_cell\_down | Cd226, Prkcq |
| GO:0048872 | homeostasis of number of cells | 1.68e-02 | 7.46e-02 | 3 | 49 | 167 | 15137 | 6.12% | 1.10% | 1.80% | 0.32% | mature\_B\_cell\_down | Cd7, F2r, Gata3 |
| GO:0030001 | metal ion transport | 1.68e-02 | 7.47e-02 | 5 | 49 | 465 | 15137 | 10.20% | 3.07% | 1.08% | 0.32% | mature\_B\_cell\_down | F2r, Lck, Slc12a7, Slc9a3r1, Tmem66 |
| GO:0045598 | regulation of fat cell differentiation | 1.72e-02 | 7.59e-02 | 2 | 49 | 62 | 15137 | 4.08% | 0.41% | 3.23% | 0.32% | mature\_B\_cell\_down | Gata3, Lpin1 |
| GO:0006816 | calcium ion transport | 1.73e-02 | 7.63e-02 | 3 | 49 | 169 | 15137 | 6.12% | 1.12% | 1.78% | 0.32% | mature\_B\_cell\_down | F2r, Lck, Tmem66 |
| GO:0010769 | regulation of cell morphogenesis involved in differentiation | 1.76e-02 | 7.73e-02 | 3 | 49 | 170 | 15137 | 6.12% | 1.12% | 1.76% | 0.32% | mature\_B\_cell\_down | Notch1, Thy1, Tnik |
| GO:0060191 | regulation of lipase activity | 1.77e-02 | 7.75e-02 | 2 | 49 | 63 | 15137 | 4.08% | 0.42% | 3.17% | 0.32% | mature\_B\_cell\_down | Itk, Txk |
| GO:0051241 | negative regulation of multicellular organismal process | 1.81e-02 | 7.80e-02 | 4 | 49 | 312 | 15137 | 8.16% | 2.06% | 1.28% | 0.32% | mature\_B\_cell\_down | F2r, Gata3, Notch1, Tnfrsf1a |
| GO:0060255 | regulation of macromolecule metabolic process | 1.85e-02 | 7.80e-02 | 19 | 49 | 3706 | 15137 | 38.78% | 24.48% | 0.51% | 0.32% | mature\_B\_cell\_down | Cd27, F2r, Fyn, Gata3, Hnrpll, Lck, Lpin1, Nckap1, Notch1, Prkch, Prkcq, Skap1, Sla2, Slc9a3r1, Tcf7, Thy1, Tnfrsf1a, Tnik, Txk |
| GO:0042127 | regulation of cell proliferation | 1.85e-02 | 7.80e-02 | 8 | 49 | 1050 | 15137 | 16.33% | 6.94% | 0.76% | 0.32% | mature\_B\_cell\_down | F2r, Gata3, Notch1, Prkch, Prkcq, Slc9a3r1, Tcf7, Zap70 |
| GO:0042102 | positive regulation of T cell proliferation | 1.88e-02 | 7.80e-02 | 2 | 49 | 65 | 15137 | 4.08% | 0.43% | 3.08% | 0.32% | mature\_B\_cell\_down | Prkcq, Zap70 |
| GO:0002821 | positive regulation of adaptive immune response | 1.88e-02 | 7.80e-02 | 2 | 49 | 65 | 15137 | 4.08% | 0.43% | 3.08% | 0.32% | mature\_B\_cell\_down | Cd226, Prkcq |
| GO:0032846 | positive regulation of homeostatic process | 1.88e-02 | 7.80e-02 | 2 | 49 | 65 | 15137 | 4.08% | 0.43% | 3.08% | 0.32% | mature\_B\_cell\_down | F2r, Thy1 |
| GO:0007262 | STAT protein import into nucleus | 1.93e-02 | 7.80e-02 | 1 | 49 | 6 | 15137 | 2.04% | 0.04% | 16.67% | 0.32% | mature\_B\_cell\_down | F2r |
| GO:0050798 | activated T cell proliferation | 1.93e-02 | 7.80e-02 | 1 | 49 | 6 | 15137 | 2.04% | 0.04% | 16.67% | 0.32% | mature\_B\_cell\_down | Fyn |
| GO:0007256 | activation of JNKK activity | 1.93e-02 | 7.80e-02 | 1 | 49 | 6 | 15137 | 2.04% | 0.04% | 16.67% | 0.32% | mature\_B\_cell\_down | Tnik |
| GO:0009912 | auditory receptor cell fate commitment | 1.93e-02 | 7.80e-02 | 1 | 49 | 6 | 15137 | 2.04% | 0.04% | 16.67% | 0.32% | mature\_B\_cell\_down | Notch1 |
| GO:0030643 | cellular phosphate ion homeostasis | 1.93e-02 | 7.80e-02 | 1 | 49 | 6 | 15137 | 2.04% | 0.04% | 16.67% | 0.32% | mature\_B\_cell\_down | Slc9a3r1 |
| GO:0072502 | cellular trivalent inorganic anion homeostasis | 1.93e-02 | 7.80e-02 | 1 | 49 | 6 | 15137 | 2.04% | 0.04% | 16.67% | 0.32% | mature\_B\_cell\_down | Slc9a3r1 |
| GO:0048305 | immunoglobulin secretion | 1.93e-02 | 7.80e-02 | 1 | 49 | 6 | 15137 | 2.04% | 0.04% | 16.67% | 0.32% | mature\_B\_cell\_down | Cd27 |
| GO:0060120 | inner ear receptor cell fate commitment | 1.93e-02 | 7.80e-02 | 1 | 49 | 6 | 15137 | 2.04% | 0.04% | 16.67% | 0.32% | mature\_B\_cell\_down | Notch1 |
| GO:0007509 | mesoderm migration involved in gastrulation | 1.93e-02 | 7.80e-02 | 1 | 49 | 6 | 15137 | 2.04% | 0.04% | 16.67% | 0.32% | mature\_B\_cell\_down | Nckap1 |
| GO:0045955 | negative regulation of calcium ion-dependent exocytosis | 1.93e-02 | 7.80e-02 | 1 | 49 | 6 | 15137 | 2.04% | 0.04% | 16.67% | 0.32% | mature\_B\_cell\_down | Notch1 |
| GO:0034351 | negative regulation of glial cell apoptotic process | 1.93e-02 | 7.80e-02 | 1 | 49 | 6 | 15137 | 2.04% | 0.04% | 16.67% | 0.32% | mature\_B\_cell\_down | Prkch |
| GO:0033600 | negative regulation of mammary gland epithelial cell proliferation | 1.93e-02 | 7.80e-02 | 1 | 49 | 6 | 15137 | 2.04% | 0.04% | 16.67% | 0.32% | mature\_B\_cell\_down | Gata3 |
| GO:0010832 | negative regulation of myotube differentiation | 1.93e-02 | 7.80e-02 | 1 | 49 | 6 | 15137 | 2.04% | 0.04% | 16.67% | 0.32% | mature\_B\_cell\_down | Notch1 |
| GO:0002860 | positive regulation of natural killer cell mediated cytotoxicity directed against tumor cell target | 1.93e-02 | 7.80e-02 | 1 | 49 | 6 | 15137 | 2.04% | 0.04% | 16.67% | 0.32% | mature\_B\_cell\_down | Cd226 |
| GO:0002857 | positive regulation of natural killer cell mediated immune response to tumor cell | 1.93e-02 | 7.80e-02 | 1 | 49 | 6 | 15137 | 2.04% | 0.04% | 16.67% | 0.32% | mature\_B\_cell\_down | Cd226 |
| GO:2000679 | positive regulation of transcription regulatory region DNA binding | 1.93e-02 | 7.80e-02 | 1 | 49 | 6 | 15137 | 2.04% | 0.04% | 16.67% | 0.32% | mature\_B\_cell\_down | Gata3 |
| GO:0050855 | regulation of B cell receptor signaling pathway | 1.93e-02 | 7.80e-02 | 1 | 49 | 6 | 15137 | 2.04% | 0.04% | 16.67% | 0.32% | mature\_B\_cell\_down | Prkch |
| GO:2000209 | regulation of anoikis | 1.93e-02 | 7.80e-02 | 1 | 49 | 6 | 15137 | 2.04% | 0.04% | 16.67% | 0.32% | mature\_B\_cell\_down | Notch1 |
| GO:0006521 | regulation of cellular amino acid metabolic process | 1.93e-02 | 7.80e-02 | 1 | 49 | 6 | 15137 | 2.04% | 0.04% | 16.67% | 0.32% | mature\_B\_cell\_down | Gata3 |
| GO:0034350 | regulation of glial cell apoptotic process | 1.93e-02 | 7.80e-02 | 1 | 49 | 6 | 15137 | 2.04% | 0.04% | 16.67% | 0.32% | mature\_B\_cell\_down | Prkch |
| GO:0070255 | regulation of mucus secretion | 1.93e-02 | 7.80e-02 | 1 | 49 | 6 | 15137 | 2.04% | 0.04% | 16.67% | 0.32% | mature\_B\_cell\_down | Sytl2 |
| GO:0016188 | synaptic vesicle maturation | 1.93e-02 | 7.80e-02 | 1 | 49 | 6 | 15137 | 2.04% | 0.04% | 16.67% | 0.32% | mature\_B\_cell\_down | Zdhhc15 |
| GO:0006642 | triglyceride mobilization | 1.93e-02 | 7.80e-02 | 1 | 49 | 6 | 15137 | 2.04% | 0.04% | 16.67% | 0.32% | mature\_B\_cell\_down | Lpin1 |
| GO:0097202 | activation of cysteine-type endopeptidase activity | 1.99e-02 | 7.95e-02 | 2 | 49 | 67 | 15137 | 4.08% | 0.44% | 2.99% | 0.32% | mature\_B\_cell\_down | F2r, Lck |
| GO:0006919 | activation of cysteine-type endopeptidase activity involved in apoptotic process | 1.99e-02 | 7.95e-02 | 2 | 49 | 67 | 15137 | 4.08% | 0.44% | 2.99% | 0.32% | mature\_B\_cell\_down | F2r, Lck |
| GO:0061041 | regulation of wound healing | 1.99e-02 | 7.95e-02 | 2 | 49 | 67 | 15137 | 4.08% | 0.44% | 2.99% | 0.32% | mature\_B\_cell\_down | F2r, Prkcq |
| GO:0043085 | positive regulation of catalytic activity | 1.99e-02 | 7.95e-02 | 6 | 49 | 666 | 15137 | 12.24% | 4.40% | 0.90% | 0.32% | mature\_B\_cell\_down | F2r, Itk, Lck, Thy1, Tnik, Txk |
| GO:0006959 | humoral immune response | 2.04e-02 | 8.15e-02 | 2 | 49 | 68 | 15137 | 4.08% | 0.45% | 2.94% | 0.32% | mature\_B\_cell\_down | Gata3, Notch1 |
| GO:0001932 | regulation of protein phosphorylation | 2.05e-02 | 8.17e-02 | 6 | 49 | 671 | 15137 | 12.24% | 4.43% | 0.89% | 0.32% | mature\_B\_cell\_down | F2r, Lck, Slc9a3r1, Thy1, Tnfrsf1a, Tnik |
| GO:0002274 | myeloid leukocyte activation | 2.16e-02 | 8.41e-02 | 2 | 49 | 70 | 15137 | 4.08% | 0.46% | 2.86% | 0.32% | mature\_B\_cell\_down | Lat, Lcp2 |
| GO:0032675 | regulation of interleukin-6 production | 2.16e-02 | 8.41e-02 | 2 | 49 | 70 | 15137 | 4.08% | 0.46% | 2.86% | 0.32% | mature\_B\_cell\_down | F2r, Tnfrsf1a |
| GO:0051928 | positive regulation of calcium ion transport | 2.21e-02 | 8.41e-02 | 2 | 49 | 71 | 15137 | 4.08% | 0.47% | 2.82% | 0.32% | mature\_B\_cell\_down | F2r, Thy1 |
| GO:0014013 | regulation of gliogenesis | 2.21e-02 | 8.41e-02 | 2 | 49 | 71 | 15137 | 4.08% | 0.47% | 2.82% | 0.32% | mature\_B\_cell\_down | Notch1, Prkch |
| GO:0010172 | embryonic body morphogenesis | 2.24e-02 | 8.41e-02 | 1 | 49 | 7 | 15137 | 2.04% | 0.05% | 14.29% | 0.32% | mature\_B\_cell\_down | Nckap1 |
| GO:0002067 | glandular epithelial cell differentiation | 2.24e-02 | 8.41e-02 | 1 | 49 | 7 | 15137 | 2.04% | 0.05% | 14.29% | 0.32% | mature\_B\_cell\_down | Notch1 |
| GO:0014067 | negative regulation of phosphatidylinositol 3-kinase cascade | 2.24e-02 | 8.41e-02 | 1 | 49 | 7 | 15137 | 2.04% | 0.05% | 14.29% | 0.32% | mature\_B\_cell\_down | Slc9a3r1 |
| GO:0010642 | negative regulation of platelet-derived growth factor receptor signaling pathway | 2.24e-02 | 8.41e-02 | 1 | 49 | 7 | 15137 | 2.04% | 0.05% | 14.29% | 0.32% | mature\_B\_cell\_down | Slc9a3r1 |
| GO:0060017 | parathyroid gland development | 2.24e-02 | 8.41e-02 | 1 | 49 | 7 | 15137 | 2.04% | 0.05% | 14.29% | 0.32% | mature\_B\_cell\_down | Gata3 |
| GO:0060158 | phospholipase C-activating dopamine receptor signaling pathway | 2.24e-02 | 8.41e-02 | 1 | 49 | 7 | 15137 | 2.04% | 0.05% | 14.29% | 0.32% | mature\_B\_cell\_down | Slc9a3r1 |
| GO:0046645 | positive regulation of gamma-delta T cell activation | 2.24e-02 | 8.41e-02 | 1 | 49 | 7 | 15137 | 2.04% | 0.05% | 14.29% | 0.32% | mature\_B\_cell\_down | Lck |
| GO:0045588 | positive regulation of gamma-delta T cell differentiation | 2.24e-02 | 8.41e-02 | 1 | 49 | 7 | 15137 | 2.04% | 0.05% | 14.29% | 0.32% | mature\_B\_cell\_down | Lck |
| GO:0070474 | positive regulation of uterine smooth muscle contraction | 2.24e-02 | 8.41e-02 | 1 | 49 | 7 | 15137 | 2.04% | 0.05% | 14.29% | 0.32% | mature\_B\_cell\_down | Lck |
| GO:0032878 | regulation of establishment or maintenance of cell polarity | 2.24e-02 | 8.41e-02 | 1 | 49 | 7 | 15137 | 2.04% | 0.05% | 14.29% | 0.32% | mature\_B\_cell\_down | Gata3 |
| GO:0046643 | regulation of gamma-delta T cell activation | 2.24e-02 | 8.41e-02 | 1 | 49 | 7 | 15137 | 2.04% | 0.05% | 14.29% | 0.32% | mature\_B\_cell\_down | Lck |
| GO:0045586 | regulation of gamma-delta T cell differentiation | 2.24e-02 | 8.41e-02 | 1 | 49 | 7 | 15137 | 2.04% | 0.05% | 14.29% | 0.32% | mature\_B\_cell\_down | Lck |
| GO:0060334 | regulation of interferon-gamma-mediated signaling pathway | 2.24e-02 | 8.41e-02 | 1 | 49 | 7 | 15137 | 2.04% | 0.05% | 14.29% | 0.32% | mature\_B\_cell\_down | Txk |
| GO:0002858 | regulation of natural killer cell mediated cytotoxicity directed against tumor cell target | 2.24e-02 | 8.41e-02 | 1 | 49 | 7 | 15137 | 2.04% | 0.05% | 14.29% | 0.32% | mature\_B\_cell\_down | Cd226 |
| GO:0002855 | regulation of natural killer cell mediated immune response to tumor cell | 2.24e-02 | 8.41e-02 | 1 | 49 | 7 | 15137 | 2.04% | 0.05% | 14.29% | 0.32% | mature\_B\_cell\_down | Cd226 |
| GO:0090330 | regulation of platelet aggregation | 2.24e-02 | 8.41e-02 | 1 | 49 | 7 | 15137 | 2.04% | 0.05% | 14.29% | 0.32% | mature\_B\_cell\_down | Prkcq |
| GO:0070472 | regulation of uterine smooth muscle contraction | 2.24e-02 | 8.41e-02 | 1 | 49 | 7 | 15137 | 2.04% | 0.05% | 14.29% | 0.32% | mature\_B\_cell\_down | Lck |
| GO:0046549 | retinal cone cell development | 2.24e-02 | 8.41e-02 | 1 | 49 | 7 | 15137 | 2.04% | 0.05% | 14.29% | 0.32% | mature\_B\_cell\_down | Thy1 |
| GO:0042670 | retinal cone cell differentiation | 2.24e-02 | 8.41e-02 | 1 | 49 | 7 | 15137 | 2.04% | 0.05% | 14.29% | 0.32% | mature\_B\_cell\_down | Thy1 |
| GO:0070493 | thrombin receptor signaling pathway | 2.24e-02 | 8.41e-02 | 1 | 49 | 7 | 15137 | 2.04% | 0.05% | 14.29% | 0.32% | mature\_B\_cell\_down | F2r |
| GO:0050707 | regulation of cytokine secretion | 2.27e-02 | 8.49e-02 | 2 | 49 | 72 | 15137 | 4.08% | 0.48% | 2.78% | 0.32% | mature\_B\_cell\_down | F2r, Gata3 |
| GO:0032101 | regulation of response to external stimulus | 2.31e-02 | 8.60e-02 | 4 | 49 | 336 | 15137 | 8.16% | 2.22% | 1.19% | 0.32% | mature\_B\_cell\_down | F2r, Gata3, Prkcq, Tnfrsf1a |
| GO:0045995 | regulation of embryonic development | 2.33e-02 | 8.67e-02 | 2 | 49 | 73 | 15137 | 4.08% | 0.48% | 2.74% | 0.32% | mature\_B\_cell\_down | Gata3, Notch1 |
| GO:0033124 | regulation of GTP catabolic process | 2.38e-02 | 8.84e-02 | 3 | 49 | 191 | 15137 | 6.12% | 1.26% | 1.57% | 0.32% | mature\_B\_cell\_down | Ipcef1, Tbc1d2b, Thy1 |
| GO:0030154 | cell differentiation | 2.39e-02 | 8.86e-02 | 13 | 49 | 2252 | 15137 | 26.53% | 14.88% | 0.58% | 0.32% | mature\_B\_cell\_down | F2r, Fyn, Gata3, Itk, Lck, Lpin1, Notch1, Prkch, Tcf7, Thy1, Tnik, Txk, Zap70 |
| GO:0034762 | regulation of transmembrane transport | 2.44e-02 | 8.93e-02 | 4 | 49 | 342 | 15137 | 8.16% | 2.26% | 1.17% | 0.32% | mature\_B\_cell\_down | Prkcq, Slc9a3r1, Tmem66, Tnfrsf1a |
| GO:0044267 | cellular protein metabolic process | 2.50e-02 | 8.93e-02 | 13 | 49 | 2265 | 15137 | 26.53% | 14.96% | 0.57% | 0.32% | mature\_B\_cell\_down | Art2b, F2r, Fyn, Ifih1, Itk, Lck, Prkch, Prkcq, Stk39, Tnik, Txk, Zap70, Zdhhc15 |
| GO:0044092 | negative regulation of molecular function | 2.55e-02 | 8.93e-02 | 6 | 49 | 705 | 15137 | 12.24% | 4.66% | 0.85% | 0.32% | mature\_B\_cell\_down | Cd27, Notch1, Slc9a3r1, Sytl2, Thy1, Tmem66 |
| GO:0045176 | apical protein localization | 2.56e-02 | 8.93e-02 | 1 | 49 | 8 | 15137 | 2.04% | 0.05% | 12.50% | 0.32% | mature\_B\_cell\_down | Nckap1 |
| GO:0071773 | cellular response to BMP stimulus | 2.56e-02 | 8.93e-02 | 1 | 49 | 8 | 15137 | 2.04% | 0.05% | 12.50% | 0.32% | mature\_B\_cell\_down | Gata3 |
| GO:0050966 | detection of mechanical stimulus involved in sensory perception of pain | 2.56e-02 | 8.93e-02 | 1 | 49 | 8 | 15137 | 2.04% | 0.05% | 12.50% | 0.32% | mature\_B\_cell\_down | Fyn |
| GO:0060986 | endocrine hormone secretion | 2.56e-02 | 8.93e-02 | 1 | 49 | 8 | 15137 | 2.04% | 0.05% | 12.50% | 0.32% | mature\_B\_cell\_down | Gata3 |
| GO:0007442 | hindgut morphogenesis | 2.56e-02 | 8.93e-02 | 1 | 49 | 8 | 15137 | 2.04% | 0.05% | 12.50% | 0.32% | mature\_B\_cell\_down | Tcf7 |
| GO:0050901 | leukocyte tethering or rolling | 2.56e-02 | 8.93e-02 | 1 | 49 | 8 | 15137 | 2.04% | 0.05% | 12.50% | 0.32% | mature\_B\_cell\_down | Selplg |
| GO:0072176 | nephric duct development | 2.56e-02 | 8.93e-02 | 1 | 49 | 8 | 15137 | 2.04% | 0.05% | 12.50% | 0.32% | mature\_B\_cell\_down | Gata3 |
| GO:0072178 | nephric duct morphogenesis | 2.56e-02 | 8.93e-02 | 1 | 49 | 8 | 15137 | 2.04% | 0.05% | 12.50% | 0.32% | mature\_B\_cell\_down | Gata3 |
| GO:0048570 | notochord morphogenesis | 2.56e-02 | 8.93e-02 | 1 | 49 | 8 | 15137 | 2.04% | 0.05% | 12.50% | 0.32% | mature\_B\_cell\_down | Nckap1 |
| GO:0035025 | positive regulation of Rho protein signal transduction | 2.56e-02 | 8.93e-02 | 1 | 49 | 8 | 15137 | 2.04% | 0.05% | 12.50% | 0.32% | mature\_B\_cell\_down | F2r |
| GO:0033240 | positive regulation of cellular amine metabolic process | 2.56e-02 | 8.93e-02 | 1 | 49 | 8 | 15137 | 2.04% | 0.05% | 12.50% | 0.32% | mature\_B\_cell\_down | Gata3 |
| GO:0032727 | positive regulation of interferon-alpha production | 2.56e-02 | 8.93e-02 | 1 | 49 | 8 | 15137 | 2.04% | 0.05% | 12.50% | 0.32% | mature\_B\_cell\_down | Ifih1 |
| GO:0042511 | positive regulation of tyrosine phosphorylation of Stat1 protein | 2.56e-02 | 8.93e-02 | 1 | 49 | 8 | 15137 | 2.04% | 0.05% | 12.50% | 0.32% | mature\_B\_cell\_down | Tnfrsf1a |
| GO:0070528 | protein kinase C signaling cascade | 2.56e-02 | 8.93e-02 | 1 | 49 | 8 | 15137 | 2.04% | 0.05% | 12.50% | 0.32% | mature\_B\_cell\_down | Prkch |
| GO:0003264 | regulation of cardioblast proliferation | 2.56e-02 | 8.93e-02 | 1 | 49 | 8 | 15137 | 2.04% | 0.05% | 12.50% | 0.32% | mature\_B\_cell\_down | Notch1 |
| GO:2001239 | regulation of extrinsic apoptotic signaling pathway in absence of ligand | 2.56e-02 | 8.93e-02 | 1 | 49 | 8 | 15137 | 2.04% | 0.05% | 12.50% | 0.32% | mature\_B\_cell\_down | Fyn |
| GO:0060330 | regulation of response to interferon-gamma | 2.56e-02 | 8.93e-02 | 1 | 49 | 8 | 15137 | 2.04% | 0.05% | 12.50% | 0.32% | mature\_B\_cell\_down | Txk |
| GO:0003266 | regulation of secondary heart field cardioblast proliferation | 2.56e-02 | 8.93e-02 | 1 | 49 | 8 | 15137 | 2.04% | 0.05% | 12.50% | 0.32% | mature\_B\_cell\_down | Notch1 |
| GO:2000649 | regulation of sodium ion transmembrane transporter activity | 2.56e-02 | 8.93e-02 | 1 | 49 | 8 | 15137 | 2.04% | 0.05% | 12.50% | 0.32% | mature\_B\_cell\_down | Slc9a3r1 |
| GO:0070293 | renal absorption | 2.56e-02 | 8.93e-02 | 1 | 49 | 8 | 15137 | 2.04% | 0.05% | 12.50% | 0.32% | mature\_B\_cell\_down | Slc9a3r1 |
| GO:0071772 | response to BMP stimulus | 2.56e-02 | 8.93e-02 | 1 | 49 | 8 | 15137 | 2.04% | 0.05% | 12.50% | 0.32% | mature\_B\_cell\_down | Gata3 |
| GO:0007260 | tyrosine phosphorylation of STAT protein | 2.56e-02 | 8.93e-02 | 1 | 49 | 8 | 15137 | 2.04% | 0.05% | 12.50% | 0.32% | mature\_B\_cell\_down | F2r |
| GO:0090316 | positive regulation of intracellular protein transport | 2.57e-02 | 8.93e-02 | 2 | 49 | 77 | 15137 | 4.08% | 0.51% | 2.60% | 0.32% | mature\_B\_cell\_down | Prkcq, Tnfrsf1a |
| GO:0051896 | regulation of protein kinase B signaling cascade | 2.57e-02 | 8.93e-02 | 2 | 49 | 77 | 15137 | 4.08% | 0.51% | 2.60% | 0.32% | mature\_B\_cell\_down | Gata3, Slc9a3r1 |
| GO:0048699 | generation of neurons | 2.65e-02 | 9.17e-02 | 7 | 49 | 912 | 15137 | 14.29% | 6.02% | 0.77% | 0.32% | mature\_B\_cell\_down | Fyn, Gata3, Notch1, Prkch, Tcf7, Thy1, Tnik |
| GO:0010557 | positive regulation of macromolecule biosynthetic process | 2.66e-02 | 9.18e-02 | 8 | 49 | 1123 | 15137 | 16.33% | 7.42% | 0.71% | 0.32% | mature\_B\_cell\_down | Cd27, F2r, Gata3, Lpin1, Notch1, Prkcq, Skap1, Tnfrsf1a |
| GO:0019538 | protein metabolic process | 2.68e-02 | 9.25e-02 | 15 | 49 | 2789 | 15137 | 30.61% | 18.43% | 0.54% | 0.32% | mature\_B\_cell\_down | Art2b, Ctsd, Ctsw, F2r, Fyn, Ifih1, Itk, Lck, Prkch, Prkcq, Stk39, Tnik, Txk, Zap70, Zdhhc15 |
| GO:0051092 | positive regulation of NF-kappaB transcription factor activity | 2.70e-02 | 9.26e-02 | 2 | 49 | 79 | 15137 | 4.08% | 0.52% | 2.53% | 0.32% | mature\_B\_cell\_down | Prkch, Prkcq |
| GO:0006937 | regulation of muscle contraction | 2.70e-02 | 9.26e-02 | 2 | 49 | 79 | 15137 | 4.08% | 0.52% | 2.53% | 0.32% | mature\_B\_cell\_down | F2r, Lck |
| GO:0002263 | cell activation involved in immune response | 2.76e-02 | 9.44e-02 | 2 | 49 | 80 | 15137 | 4.08% | 0.53% | 2.50% | 0.32% | mature\_B\_cell\_down | Gata3, Lat |
| GO:0002366 | leukocyte activation involved in immune response | 2.76e-02 | 9.44e-02 | 2 | 49 | 80 | 15137 | 4.08% | 0.53% | 2.50% | 0.32% | mature\_B\_cell\_down | Gata3, Lat |
| GO:0032388 | positive regulation of intracellular transport | 2.83e-02 | 9.46e-02 | 2 | 49 | 81 | 15137 | 4.08% | 0.54% | 2.47% | 0.32% | mature\_B\_cell\_down | Prkcq, Tnfrsf1a |
| GO:0045935 | positive regulation of nucleobase-containing compound metabolic process | 2.84e-02 | 9.46e-02 | 8 | 49 | 1137 | 15137 | 16.33% | 7.51% | 0.70% | 0.32% | mature\_B\_cell\_down | F2r, Gata3, Hnrpll, Ipcef1, Lpin1, Notch1, Skap1, Tnfrsf1a |
| GO:0031929 | TOR signaling cascade | 2.88e-02 | 9.46e-02 | 1 | 49 | 9 | 15137 | 2.04% | 0.06% | 11.11% | 0.32% | mature\_B\_cell\_down | Gata3 |
| GO:0007191 | adenylate cyclase-activating dopamine receptor signaling pathway | 2.88e-02 | 9.46e-02 | 1 | 49 | 9 | 15137 | 2.04% | 0.06% | 11.11% | 0.32% | mature\_B\_cell\_down | Slc9a3r1 |
| GO:0060231 | mesenchymal to epithelial transition | 2.88e-02 | 9.46e-02 | 1 | 49 | 9 | 15137 | 2.04% | 0.06% | 11.11% | 0.32% | mature\_B\_cell\_down | Gata3 |
| GO:2000352 | negative regulation of endothelial cell apoptotic process | 2.88e-02 | 9.46e-02 | 1 | 49 | 9 | 15137 | 2.04% | 0.06% | 11.11% | 0.32% | mature\_B\_cell\_down | Gata3 |
| GO:0060253 | negative regulation of glial cell proliferation | 2.88e-02 | 9.46e-02 | 1 | 49 | 9 | 15137 | 2.04% | 0.06% | 11.11% | 0.32% | mature\_B\_cell\_down | Notch1 |
| GO:0033120 | positive regulation of RNA splicing | 2.88e-02 | 9.46e-02 | 1 | 49 | 9 | 15137 | 2.04% | 0.06% | 11.11% | 0.32% | mature\_B\_cell\_down | Hnrpll |
| GO:0050857 | positive regulation of antigen receptor-mediated signaling pathway | 2.88e-02 | 9.46e-02 | 1 | 49 | 9 | 15137 | 2.04% | 0.06% | 11.11% | 0.32% | mature\_B\_cell\_down | Prkch |
| GO:0045603 | positive regulation of endothelial cell differentiation | 2.88e-02 | 9.46e-02 | 1 | 49 | 9 | 15137 | 2.04% | 0.06% | 11.11% | 0.32% | mature\_B\_cell\_down | Notch1 |
| GO:0060252 | positive regulation of glial cell proliferation | 2.88e-02 | 9.46e-02 | 1 | 49 | 9 | 15137 | 2.04% | 0.06% | 11.11% | 0.32% | mature\_B\_cell\_down | Prkch |
| GO:0031065 | positive regulation of histone deacetylation | 2.88e-02 | 9.46e-02 | 1 | 49 | 9 | 15137 | 2.04% | 0.06% | 11.11% | 0.32% | mature\_B\_cell\_down | Lpin1 |
| GO:0010744 | positive regulation of macrophage derived foam cell differentiation | 2.88e-02 | 9.46e-02 | 1 | 49 | 9 | 15137 | 2.04% | 0.06% | 11.11% | 0.32% | mature\_B\_cell\_down | Prkch |
| GO:0043268 | positive regulation of potassium ion transport | 2.88e-02 | 9.46e-02 | 1 | 49 | 9 | 15137 | 2.04% | 0.06% | 11.11% | 0.32% | mature\_B\_cell\_down | Stk39 |
| GO:0003093 | regulation of glomerular filtration | 2.88e-02 | 9.46e-02 | 1 | 49 | 9 | 15137 | 2.04% | 0.06% | 11.11% | 0.32% | mature\_B\_cell\_down | F2r |
| GO:0010640 | regulation of platelet-derived growth factor receptor signaling pathway | 2.88e-02 | 9.46e-02 | 1 | 49 | 9 | 15137 | 2.04% | 0.06% | 11.11% | 0.32% | mature\_B\_cell\_down | Slc9a3r1 |
| GO:0032495 | response to muramyl dipeptide | 2.88e-02 | 9.46e-02 | 1 | 49 | 9 | 15137 | 2.04% | 0.06% | 11.11% | 0.32% | mature\_B\_cell\_down | Notch1 |
| GO:0019932 | second-messenger-mediated signaling | 2.89e-02 | 9.49e-02 | 2 | 49 | 82 | 15137 | 4.08% | 0.54% | 2.44% | 0.32% | mature\_B\_cell\_down | Lat, Slc9a3r1 |
| GO:0070838 | divalent metal ion transport | 2.93e-02 | 9.60e-02 | 3 | 49 | 207 | 15137 | 6.12% | 1.37% | 1.45% | 0.32% | mature\_B\_cell\_down | F2r, Lck, Tmem66 |
| GO:0006357 | regulation of transcription from RNA polymerase II promoter | 2.95e-02 | 9.63e-02 | 8 | 49 | 1146 | 15137 | 16.33% | 7.57% | 0.70% | 0.32% | mature\_B\_cell\_down | Gata3, Lpin1, Notch1, Skap1, Sla2, Tcf7, Tnfrsf1a, Txk |
| GO:0051716 | cellular response to stimulus | 2.95e-02 | 9.63e-02 | 23 | 49 | 4999 | 15137 | 46.94% | 33.03% | 0.46% | 0.32% | mature\_B\_cell\_down | Apol7b, Cd247, Cd3g, Ctsd, F2r, Fyn, Gata3, Itk, Lat, Lck, Lpin1, Notch1, Prkch, Prkcq, Rab37, Skap1, Slc9a3r1, Tcf7, Thy1, Tnfrsf1a, Tnik, Txk, Zap70 |
| GO:0042325 | regulation of phosphorylation | 3.01e-02 | 9.78e-02 | 6 | 49 | 733 | 15137 | 12.24% | 4.84% | 0.82% | 0.32% | mature\_B\_cell\_down | F2r, Lck, Slc9a3r1, Thy1, Tnfrsf1a, Tnik |
| GO:0050768 | negative regulation of neurogenesis | 3.02e-02 | 9.82e-02 | 2 | 49 | 84 | 15137 | 4.08% | 0.55% | 2.38% | 0.32% | mature\_B\_cell\_down | Notch1, Thy1 |
| GO:0072511 | divalent inorganic cation transport | 3.04e-02 | 9.83e-02 | 3 | 49 | 210 | 15137 | 6.12% | 1.39% | 1.43% | 0.32% | mature\_B\_cell\_down | F2r, Lck, Tmem66 |
| GO:0031401 | positive regulation of protein modification process | 3.04e-02 | 9.83e-02 | 5 | 49 | 543 | 15137 | 10.20% | 3.59% | 0.92% | 0.32% | mature\_B\_cell\_down | F2r, Lck, Lpin1, Tnfrsf1a, Tnik |
| GO:0051128 | regulation of cellular component organization | 3.08e-02 | 9.93e-02 | 8 | 49 | 1155 | 15137 | 16.33% | 7.63% | 0.69% | 0.32% | mature\_B\_cell\_down | Fyn, Gata3, Lpin1, Notch1, Prkch, Prkcq, Thy1, Tnik |
| GO:0051173 | positive regulation of nitrogen compound metabolic process | 3.15e-02 | 9.96e-02 | 8 | 49 | 1160 | 15137 | 16.33% | 7.66% | 0.69% | 0.32% | mature\_B\_cell\_down | F2r, Gata3, Hnrpll, Ipcef1, Lpin1, Notch1, Skap1, Tnfrsf1a |
| GO:0007163 | establishment or maintenance of cell polarity | 3.16e-02 | 9.96e-02 | 2 | 49 | 86 | 15137 | 4.08% | 0.57% | 2.33% | 0.32% | mature\_B\_cell\_down | Cd3g, Nckap1 |
| GO:0001894 | tissue homeostasis | 3.16e-02 | 9.96e-02 | 2 | 49 | 86 | 15137 | 4.08% | 0.57% | 2.33% | 0.32% | mature\_B\_cell\_down | Cd7, F2r |
| GO:0030811 | regulation of nucleotide catabolic process | 3.19e-02 | 9.96e-02 | 3 | 49 | 214 | 15137 | 6.12% | 1.41% | 1.40% | 0.32% | mature\_B\_cell\_down | Ipcef1, Tbc1d2b, Thy1 |
| GO:0033121 | regulation of purine nucleotide catabolic process | 3.19e-02 | 9.96e-02 | 3 | 49 | 214 | 15137 | 6.12% | 1.41% | 1.40% | 0.32% | mature\_B\_cell\_down | Ipcef1, Tbc1d2b, Thy1 |
| GO:0003211 | cardiac ventricle formation | 3.19e-02 | 9.96e-02 | 1 | 49 | 10 | 15137 | 2.04% | 0.07% | 10.00% | 0.32% | mature\_B\_cell\_down | Notch1 |
| GO:0042415 | norepinephrine metabolic process | 3.19e-02 | 9.96e-02 | 1 | 49 | 10 | 15137 | 2.04% | 0.07% | 10.00% | 0.32% | mature\_B\_cell\_down | Gata3 |
| GO:0055062 | phosphate ion homeostasis | 3.19e-02 | 9.96e-02 | 1 | 49 | 10 | 15137 | 2.04% | 0.07% | 10.00% | 0.32% | mature\_B\_cell\_down | Slc9a3r1 |
| GO:0048711 | positive regulation of astrocyte differentiation | 3.19e-02 | 9.96e-02 | 1 | 49 | 10 | 15137 | 2.04% | 0.07% | 10.00% | 0.32% | mature\_B\_cell\_down | Notch1 |
| GO:0032740 | positive regulation of interleukin-17 production | 3.19e-02 | 9.96e-02 | 1 | 49 | 10 | 15137 | 2.04% | 0.07% | 10.00% | 0.32% | mature\_B\_cell\_down | Prkcq |
| GO:0060768 | regulation of epithelial cell proliferation involved in prostate gland development | 3.19e-02 | 9.96e-02 | 1 | 49 | 10 | 15137 | 2.04% | 0.07% | 10.00% | 0.32% | mature\_B\_cell\_down | Notch1 |
| GO:0032656 | regulation of interleukin-13 production | 3.19e-02 | 9.96e-02 | 1 | 49 | 10 | 15137 | 2.04% | 0.07% | 10.00% | 0.32% | mature\_B\_cell\_down | Gata3 |
| GO:0032674 | regulation of interleukin-5 production | 3.19e-02 | 9.96e-02 | 1 | 49 | 10 | 15137 | 2.04% | 0.07% | 10.00% | 0.32% | mature\_B\_cell\_down | Gata3 |
| GO:0072182 | regulation of nephron tubule epithelial cell differentiation | 3.19e-02 | 9.96e-02 | 1 | 49 | 10 | 15137 | 2.04% | 0.07% | 10.00% | 0.32% | mature\_B\_cell\_down | Gata3 |
| GO:0010155 | regulation of proton transport | 3.19e-02 | 9.96e-02 | 1 | 49 | 10 | 15137 | 2.04% | 0.07% | 10.00% | 0.32% | mature\_B\_cell\_down | Slc9a3r1 |
| GO:0072506 | trivalent inorganic anion homeostasis | 3.19e-02 | 9.96e-02 | 1 | 49 | 10 | 15137 | 2.04% | 0.07% | 10.00% | 0.32% | mature\_B\_cell\_down | Slc9a3r1 |
| GO:0060070 | canonical Wnt receptor signaling pathway | 3.23e-02 | 1.00e-01 | 2 | 49 | 87 | 15137 | 4.08% | 0.57% | 2.30% | 0.32% | mature\_B\_cell\_down | Gata3, Tcf7 |
| GO:0071345 | cellular response to cytokine stimulus | 3.23e-02 | 1.00e-01 | 3 | 49 | 215 | 15137 | 6.12% | 1.42% | 1.40% | 0.32% | mature\_B\_cell\_down | Gata3, Tcf7, Tnfrsf1a |
| GO:0048869 | cellular developmental process | 3.23e-02 | 1.00e-01 | 13 | 49 | 2345 | 15137 | 26.53% | 15.49% | 0.55% | 0.32% | mature\_B\_cell\_down | F2r, Fyn, Gata3, Itk, Lck, Lpin1, Notch1, Prkch, Tcf7, Thy1, Tnik, Txk, Zap70 |
| GO:0007010 | cytoskeleton organization | 3.25e-02 | 1.01e-01 | 5 | 49 | 553 | 15137 | 10.20% | 3.65% | 0.90% | 0.32% | mature\_B\_cell\_down | Lpin1, Nckap1, Slc9a3r1, Thy1, Tnik |
| GO:0030334 | regulation of cell migration | 3.30e-02 | 1.02e-01 | 4 | 49 | 376 | 15137 | 8.16% | 2.48% | 1.06% | 0.32% | mature\_B\_cell\_down | F2r, Gata3, Slc9a3r1, Thy1 |
| GO:2001056 | positive regulation of cysteine-type endopeptidase activity | 3.43e-02 | 1.03e-01 | 2 | 49 | 90 | 15137 | 4.08% | 0.59% | 2.22% | 0.32% | mature\_B\_cell\_down | F2r, Lck |
| GO:0043280 | positive regulation of cysteine-type endopeptidase activity involved in apoptotic process | 3.43e-02 | 1.03e-01 | 2 | 49 | 90 | 15137 | 4.08% | 0.59% | 2.22% | 0.32% | mature\_B\_cell\_down | F2r, Lck |
| GO:0033044 | regulation of chromosome organization | 3.50e-02 | 1.03e-01 | 2 | 49 | 91 | 15137 | 4.08% | 0.60% | 2.20% | 0.32% | mature\_B\_cell\_down | Gata3, Lpin1 |
| GO:0042640 | anagen | 3.50e-02 | 1.03e-01 | 1 | 49 | 11 | 15137 | 2.04% | 0.07% | 9.09% | 0.32% | mature\_B\_cell\_down | Notch1 |
| GO:0008595 | anterior/posterior axis specification, embryo | 3.50e-02 | 1.03e-01 | 1 | 49 | 11 | 15137 | 2.04% | 0.07% | 9.09% | 0.32% | mature\_B\_cell\_down | Nckap1 |
| GO:0007350 | blastoderm segmentation | 3.50e-02 | 1.03e-01 | 1 | 49 | 11 | 15137 | 2.04% | 0.07% | 9.09% | 0.32% | mature\_B\_cell\_down | Nckap1 |
| GO:0003207 | cardiac chamber formation | 3.50e-02 | 1.03e-01 | 1 | 49 | 11 | 15137 | 2.04% | 0.07% | 9.09% | 0.32% | mature\_B\_cell\_down | Notch1 |
| GO:0061311 | cell surface receptor signaling pathway involved in heart development | 3.50e-02 | 1.03e-01 | 1 | 49 | 11 | 15137 | 2.04% | 0.07% | 9.09% | 0.32% | mature\_B\_cell\_down | Notch1 |
| GO:0048617 | embryonic foregut morphogenesis | 3.50e-02 | 1.03e-01 | 1 | 49 | 11 | 15137 | 2.04% | 0.07% | 9.09% | 0.32% | mature\_B\_cell\_down | Nckap1 |
| GO:0002320 | lymphoid progenitor cell differentiation | 3.50e-02 | 1.03e-01 | 1 | 49 | 11 | 15137 | 2.04% | 0.07% | 9.09% | 0.32% | mature\_B\_cell\_down | Gata3 |
| GO:0002279 | mast cell activation involved in immune response | 3.50e-02 | 1.03e-01 | 1 | 49 | 11 | 15137 | 2.04% | 0.07% | 9.09% | 0.32% | mature\_B\_cell\_down | Lat |
| GO:0043303 | mast cell degranulation | 3.50e-02 | 1.03e-01 | 1 | 49 | 11 | 15137 | 2.04% | 0.07% | 9.09% | 0.32% | mature\_B\_cell\_down | Lat |
| GO:0002448 | mast cell mediated immunity | 3.50e-02 | 1.03e-01 | 1 | 49 | 11 | 15137 | 2.04% | 0.07% | 9.09% | 0.32% | mature\_B\_cell\_down | Lat |
| GO:0050860 | negative regulation of T cell receptor signaling pathway | 3.50e-02 | 1.03e-01 | 1 | 49 | 11 | 15137 | 2.04% | 0.07% | 9.09% | 0.32% | mature\_B\_cell\_down | Thy1 |
| GO:0032703 | negative regulation of interleukin-2 production | 3.50e-02 | 1.03e-01 | 1 | 49 | 11 | 15137 | 2.04% | 0.07% | 9.09% | 0.32% | mature\_B\_cell\_down | Gata3 |
| GO:0045662 | negative regulation of myoblast differentiation | 3.50e-02 | 1.03e-01 | 1 | 49 | 11 | 15137 | 2.04% | 0.07% | 9.09% | 0.32% | mature\_B\_cell\_down | Notch1 |
| GO:0006857 | oligopeptide transport | 3.50e-02 | 1.03e-01 | 1 | 49 | 11 | 15137 | 2.04% | 0.07% | 9.09% | 0.32% | mature\_B\_cell\_down | Slc9a3r1 |
| GO:0048340 | paraxial mesoderm morphogenesis | 3.50e-02 | 1.03e-01 | 1 | 49 | 11 | 15137 | 2.04% | 0.07% | 9.09% | 0.32% | mature\_B\_cell\_down | Nckap1 |
| GO:0045059 | positive thymic T cell selection | 3.50e-02 | 1.03e-01 | 1 | 49 | 11 | 15137 | 2.04% | 0.07% | 9.09% | 0.32% | mature\_B\_cell\_down | Zap70 |
| GO:0032234 | regulation of calcium ion transport via store-operated calcium channel activity | 3.50e-02 | 1.03e-01 | 1 | 49 | 11 | 15137 | 2.04% | 0.07% | 9.09% | 0.32% | mature\_B\_cell\_down | Tmem66 |
| GO:0042510 | regulation of tyrosine phosphorylation of Stat1 protein | 3.50e-02 | 1.03e-01 | 1 | 49 | 11 | 15137 | 2.04% | 0.07% | 9.09% | 0.32% | mature\_B\_cell\_down | Tnfrsf1a |
| GO:0001977 | renal system process involved in regulation of blood volume | 3.50e-02 | 1.03e-01 | 1 | 49 | 11 | 15137 | 2.04% | 0.07% | 9.09% | 0.32% | mature\_B\_cell\_down | F2r |
| GO:0007351 | tripartite regional subdivision | 3.50e-02 | 1.03e-01 | 1 | 49 | 11 | 15137 | 2.04% | 0.07% | 9.09% | 0.32% | mature\_B\_cell\_down | Nckap1 |
| GO:0072189 | ureter development | 3.50e-02 | 1.03e-01 | 1 | 49 | 11 | 15137 | 2.04% | 0.07% | 9.09% | 0.32% | mature\_B\_cell\_down | Gata3 |
| GO:0060341 | regulation of cellular localization | 3.52e-02 | 1.03e-01 | 5 | 49 | 565 | 15137 | 10.20% | 3.73% | 0.88% | 0.32% | mature\_B\_cell\_down | F2r, Gata3, Notch1, Prkcq, Tnfrsf1a |
| GO:0003231 | cardiac ventricle development | 3.57e-02 | 1.05e-01 | 2 | 49 | 92 | 15137 | 4.08% | 0.61% | 2.17% | 0.32% | mature\_B\_cell\_down | Gata3, Notch1 |
| GO:0051291 | protein heterooligomerization | 3.57e-02 | 1.05e-01 | 2 | 49 | 92 | 15137 | 4.08% | 0.61% | 2.17% | 0.32% | mature\_B\_cell\_down | Sept9, Tnfrsf1a |
| GO:0048562 | embryonic organ morphogenesis | 3.58e-02 | 1.05e-01 | 3 | 49 | 224 | 15137 | 6.12% | 1.48% | 1.34% | 0.32% | mature\_B\_cell\_down | Gata3, Nckap1, Tcf7 |
| GO:0022008 | neurogenesis | 3.65e-02 | 1.06e-01 | 7 | 49 | 976 | 15137 | 14.29% | 6.45% | 0.72% | 0.32% | mature\_B\_cell\_down | Fyn, Gata3, Notch1, Prkch, Tcf7, Thy1, Tnik |
| GO:0019219 | regulation of nucleobase-containing compound metabolic process | 3.70e-02 | 1.06e-01 | 15 | 49 | 2902 | 15137 | 30.61% | 19.17% | 0.52% | 0.32% | mature\_B\_cell\_down | F2r, Gata3, Hnrpll, Ipcef1, Lpin1, Notch1, Prkch, Prkcq, Skap1, Sla2, Tbc1d2b, Tcf7, Thy1, Tnfrsf1a, Txk |
| GO:2000145 | regulation of cell motility | 3.70e-02 | 1.06e-01 | 4 | 49 | 390 | 15137 | 8.16% | 2.58% | 1.03% | 0.32% | mature\_B\_cell\_down | F2r, Gata3, Slc9a3r1, Thy1 |
| GO:0002294 | CD4-positive, alpha-beta T cell differentiation involved in immune response | 3.82e-02 | 1.06e-01 | 1 | 49 | 12 | 15137 | 2.04% | 0.08% | 8.33% | 0.32% | mature\_B\_cell\_down | Gata3 |
| GO:0042093 | T-helper cell differentiation | 3.82e-02 | 1.06e-01 | 1 | 49 | 12 | 15137 | 2.04% | 0.08% | 8.33% | 0.32% | mature\_B\_cell\_down | Gata3 |
| GO:0033151 | V(D)J recombination | 3.82e-02 | 1.06e-01 | 1 | 49 | 12 | 15137 | 2.04% | 0.08% | 8.33% | 0.32% | mature\_B\_cell\_down | Tcf7 |
| GO:0008105 | asymmetric protein localization | 3.82e-02 | 1.06e-01 | 1 | 49 | 12 | 15137 | 2.04% | 0.08% | 8.33% | 0.32% | mature\_B\_cell\_down | Nckap1 |
| GO:0060219 | camera-type eye photoreceptor cell differentiation | 3.82e-02 | 1.06e-01 | 1 | 49 | 12 | 15137 | 2.04% | 0.08% | 8.33% | 0.32% | mature\_B\_cell\_down | Thy1 |
| GO:0001823 | mesonephros development | 3.82e-02 | 1.06e-01 | 1 | 49 | 12 | 15137 | 2.04% | 0.08% | 8.33% | 0.32% | mature\_B\_cell\_down | Gata3 |
| GO:0000266 | mitochondrial fission | 3.82e-02 | 1.06e-01 | 1 | 49 | 12 | 15137 | 2.04% | 0.08% | 8.33% | 0.32% | mature\_B\_cell\_down | Lpin1 |
| GO:0072337 | modified amino acid transport | 3.82e-02 | 1.06e-01 | 1 | 49 | 12 | 15137 | 2.04% | 0.08% | 8.33% | 0.32% | mature\_B\_cell\_down | Slc9a3r1 |
| GO:0070233 | negative regulation of T cell apoptotic process | 3.82e-02 | 1.06e-01 | 1 | 49 | 12 | 15137 | 2.04% | 0.08% | 8.33% | 0.32% | mature\_B\_cell\_down | Prkcq |
| GO:0050858 | negative regulation of antigen receptor-mediated signaling pathway | 3.82e-02 | 1.06e-01 | 1 | 49 | 12 | 15137 | 2.04% | 0.08% | 8.33% | 0.32% | mature\_B\_cell\_down | Thy1 |
| GO:0040037 | negative regulation of fibroblast growth factor receptor signaling pathway | 3.82e-02 | 1.06e-01 | 1 | 49 | 12 | 15137 | 2.04% | 0.08% | 8.33% | 0.32% | mature\_B\_cell\_down | Gata3 |
| GO:0048715 | negative regulation of oligodendrocyte differentiation | 3.82e-02 | 1.06e-01 | 1 | 49 | 12 | 15137 | 2.04% | 0.08% | 8.33% | 0.32% | mature\_B\_cell\_down | Notch1 |
| GO:0045060 | negative thymic T cell selection | 3.82e-02 | 1.06e-01 | 1 | 49 | 12 | 15137 | 2.04% | 0.08% | 8.33% | 0.32% | mature\_B\_cell\_down | Zap70 |
| GO:0001961 | positive regulation of cytokine-mediated signaling pathway | 3.82e-02 | 1.06e-01 | 1 | 49 | 12 | 15137 | 2.04% | 0.08% | 8.33% | 0.32% | mature\_B\_cell\_down | Txk |
| GO:0045078 | positive regulation of interferon-gamma biosynthetic process | 3.82e-02 | 1.06e-01 | 1 | 49 | 12 | 15137 | 2.04% | 0.08% | 8.33% | 0.32% | mature\_B\_cell\_down | Cd27 |
| GO:0045086 | positive regulation of interleukin-2 biosynthetic process | 3.82e-02 | 1.06e-01 | 1 | 49 | 12 | 15137 | 2.04% | 0.08% | 8.33% | 0.32% | mature\_B\_cell\_down | Prkcq |
| GO:0033005 | positive regulation of mast cell activation | 3.82e-02 | 1.06e-01 | 1 | 49 | 12 | 15137 | 2.04% | 0.08% | 8.33% | 0.32% | mature\_B\_cell\_down | Cd226 |
| GO:0090312 | positive regulation of protein deacetylation | 3.82e-02 | 1.06e-01 | 1 | 49 | 12 | 15137 | 2.04% | 0.08% | 8.33% | 0.32% | mature\_B\_cell\_down | Lpin1 |
| GO:0033160 | positive regulation of protein import into nucleus, translocation | 3.82e-02 | 1.06e-01 | 1 | 49 | 12 | 15137 | 2.04% | 0.08% | 8.33% | 0.32% | mature\_B\_cell\_down | Tnfrsf1a |
| GO:0060525 | prostate glandular acinus development | 3.82e-02 | 1.06e-01 | 1 | 49 | 12 | 15137 | 2.04% | 0.08% | 8.33% | 0.32% | mature\_B\_cell\_down | Notch1 |
| GO:0018345 | protein palmitoylation | 3.82e-02 | 1.06e-01 | 1 | 49 | 12 | 15137 | 2.04% | 0.08% | 8.33% | 0.32% | mature\_B\_cell\_down | Zdhhc15 |
| GO:0045601 | regulation of endothelial cell differentiation | 3.82e-02 | 1.06e-01 | 1 | 49 | 12 | 15137 | 2.04% | 0.08% | 8.33% | 0.32% | mature\_B\_cell\_down | Notch1 |
| GO:2000696 | regulation of epithelial cell differentiation involved in kidney development | 3.82e-02 | 1.06e-01 | 1 | 49 | 12 | 15137 | 2.04% | 0.08% | 8.33% | 0.32% | mature\_B\_cell\_down | Gata3 |
| GO:0050671 | positive regulation of lymphocyte proliferation | 3.86e-02 | 1.07e-01 | 2 | 49 | 96 | 15137 | 4.08% | 0.63% | 2.08% | 0.32% | mature\_B\_cell\_down | Prkcq, Zap70 |
| GO:0006812 | cation transport | 3.92e-02 | 1.09e-01 | 5 | 49 | 582 | 15137 | 10.20% | 3.84% | 0.86% | 0.32% | mature\_B\_cell\_down | F2r, Lck, Slc12a7, Slc9a3r1, Tmem66 |
| GO:0010950 | positive regulation of endopeptidase activity | 3.93e-02 | 1.09e-01 | 2 | 49 | 97 | 15137 | 4.08% | 0.64% | 2.06% | 0.32% | mature\_B\_cell\_down | F2r, Lck |
| GO:0032946 | positive regulation of mononuclear cell proliferation | 3.93e-02 | 1.09e-01 | 2 | 49 | 97 | 15137 | 4.08% | 0.64% | 2.06% | 0.32% | mature\_B\_cell\_down | Prkcq, Zap70 |
| GO:0031328 | positive regulation of cellular biosynthetic process | 4.00e-02 | 1.09e-01 | 8 | 49 | 1216 | 15137 | 16.33% | 8.03% | 0.66% | 0.32% | mature\_B\_cell\_down | Cd27, F2r, Gata3, Lpin1, Notch1, Prkcq, Skap1, Tnfrsf1a |
| GO:0003206 | cardiac chamber morphogenesis | 4.01e-02 | 1.09e-01 | 2 | 49 | 98 | 15137 | 4.08% | 0.65% | 2.04% | 0.32% | mature\_B\_cell\_down | Gata3, Notch1 |
| GO:0001818 | negative regulation of cytokine production | 4.01e-02 | 1.09e-01 | 2 | 49 | 98 | 15137 | 4.08% | 0.65% | 2.04% | 0.32% | mature\_B\_cell\_down | Gata3, Tnfrsf1a |
| GO:0001764 | neuron migration | 4.01e-02 | 1.09e-01 | 2 | 49 | 98 | 15137 | 4.08% | 0.65% | 2.04% | 0.32% | mature\_B\_cell\_down | Fyn, Gata3 |
| GO:0032412 | regulation of ion transmembrane transporter activity | 4.01e-02 | 1.09e-01 | 2 | 49 | 98 | 15137 | 4.08% | 0.65% | 2.04% | 0.32% | mature\_B\_cell\_down | Slc9a3r1, Tmem66 |
| GO:0090257 | regulation of muscle system process | 4.01e-02 | 1.09e-01 | 2 | 49 | 98 | 15137 | 4.08% | 0.65% | 2.04% | 0.32% | mature\_B\_cell\_down | F2r, Lck |
| GO:0051171 | regulation of nitrogen compound metabolic process | 4.05e-02 | 1.10e-01 | 15 | 49 | 2935 | 15137 | 30.61% | 19.39% | 0.51% | 0.32% | mature\_B\_cell\_down | F2r, Gata3, Hnrpll, Ipcef1, Lpin1, Notch1, Prkch, Prkcq, Skap1, Sla2, Tbc1d2b, Tcf7, Thy1, Tnfrsf1a, Txk |
| GO:0002822 | regulation of adaptive immune response based on somatic recombination of immune receptors built from immunoglobulin superfamily domains | 4.08e-02 | 1.10e-01 | 2 | 49 | 99 | 15137 | 4.08% | 0.65% | 2.02% | 0.32% | mature\_B\_cell\_down | Cd226, Prkcq |
| GO:0043383 | negative T cell selection | 4.13e-02 | 1.10e-01 | 1 | 49 | 13 | 15137 | 2.04% | 0.09% | 7.69% | 0.32% | mature\_B\_cell\_down | Zap70 |
| GO:2001237 | negative regulation of extrinsic apoptotic signaling pathway | 4.13e-02 | 1.10e-01 | 1 | 49 | 13 | 15137 | 2.04% | 0.09% | 7.69% | 0.32% | mature\_B\_cell\_down | Fyn |
| GO:0002839 | positive regulation of immune response to tumor cell | 4.13e-02 | 1.10e-01 | 1 | 49 | 13 | 15137 | 2.04% | 0.09% | 7.69% | 0.32% | mature\_B\_cell\_down | Cd226 |
| GO:0060760 | positive regulation of response to cytokine stimulus | 4.13e-02 | 1.10e-01 | 1 | 49 | 13 | 15137 | 2.04% | 0.09% | 7.69% | 0.32% | mature\_B\_cell\_down | Txk |
| GO:0002836 | positive regulation of response to tumor cell | 4.13e-02 | 1.10e-01 | 1 | 49 | 13 | 15137 | 2.04% | 0.09% | 7.69% | 0.32% | mature\_B\_cell\_down | Cd226 |
| GO:2000351 | regulation of endothelial cell apoptotic process | 4.13e-02 | 1.10e-01 | 1 | 49 | 13 | 15137 | 2.04% | 0.09% | 7.69% | 0.32% | mature\_B\_cell\_down | Gata3 |
| GO:0031063 | regulation of histone deacetylation | 4.13e-02 | 1.10e-01 | 1 | 49 | 13 | 15137 | 2.04% | 0.09% | 7.69% | 0.32% | mature\_B\_cell\_down | Lpin1 |
| GO:0032647 | regulation of interferon-alpha production | 4.13e-02 | 1.10e-01 | 1 | 49 | 13 | 15137 | 2.04% | 0.09% | 7.69% | 0.32% | mature\_B\_cell\_down | Ifih1 |
| GO:0045072 | regulation of interferon-gamma biosynthetic process | 4.13e-02 | 1.10e-01 | 1 | 49 | 13 | 15137 | 2.04% | 0.09% | 7.69% | 0.32% | mature\_B\_cell\_down | Cd27 |
| GO:0090130 | tissue migration | 4.13e-02 | 1.10e-01 | 1 | 49 | 13 | 15137 | 2.04% | 0.09% | 7.69% | 0.32% | mature\_B\_cell\_down | Nckap1 |
| GO:0060065 | uterus development | 4.13e-02 | 1.10e-01 | 1 | 49 | 13 | 15137 | 2.04% | 0.09% | 7.69% | 0.32% | mature\_B\_cell\_down | Gata3 |
| GO:0050900 | leukocyte migration | 4.16e-02 | 1.11e-01 | 2 | 49 | 100 | 15137 | 4.08% | 0.66% | 2.00% | 0.32% | mature\_B\_cell\_down | Gata3, Selplg |
| GO:0070665 | positive regulation of leukocyte proliferation | 4.16e-02 | 1.11e-01 | 2 | 49 | 100 | 15137 | 4.08% | 0.66% | 2.00% | 0.32% | mature\_B\_cell\_down | Prkcq, Zap70 |
| GO:0065007 | biological regulation | 4.19e-02 | 1.11e-01 | 36 | 49 | 9171 | 15137 | 73.47% | 60.59% | 0.39% | 0.32% | mature\_B\_cell\_down | Apol7b, Cd226, Cd247, Cd27, Cd3g, Cd7, F2r, Fyn, Gata3, Hnrpll, Ifih1, Ipcef1, Itk, Lat, Lck, Lpin1, Nckap1, Notch1, Prkch, Prkcq, Rab37, Rinl, Skap1, Sla2, Slc12a7, Slc9a3r1, Stk39, Sytl2, Tbc1d2b, Tcf7, Thy1, Tmem66, Tnfrsf1a, Tnik, Txk, Zap70 |
| GO:0051254 | positive regulation of RNA metabolic process | 4.23e-02 | 1.12e-01 | 7 | 49 | 1008 | 15137 | 14.29% | 6.66% | 0.69% | 0.32% | mature\_B\_cell\_down | F2r, Gata3, Hnrpll, Lpin1, Notch1, Skap1, Tnfrsf1a |
| GO:0010721 | negative regulation of cell development | 4.23e-02 | 1.12e-01 | 2 | 49 | 101 | 15137 | 4.08% | 0.67% | 1.98% | 0.32% | mature\_B\_cell\_down | Notch1, Thy1 |
| GO:0042592 | homeostatic process | 4.29e-02 | 1.13e-01 | 7 | 49 | 1011 | 15137 | 14.29% | 6.68% | 0.69% | 0.32% | mature\_B\_cell\_down | Cd7, F2r, Fyn, Gata3, Lck, Slc12a7, Slc9a3r1 |
| GO:0032844 | regulation of homeostatic process | 4.30e-02 | 1.13e-01 | 3 | 49 | 241 | 15137 | 6.12% | 1.59% | 1.24% | 0.32% | mature\_B\_cell\_down | F2r, Lck, Thy1 |
| GO:0045088 | regulation of innate immune response | 4.31e-02 | 1.13e-01 | 2 | 49 | 102 | 15137 | 4.08% | 0.67% | 1.96% | 0.32% | mature\_B\_cell\_down | Cd226, Txk |
| GO:0022898 | regulation of transmembrane transporter activity | 4.38e-02 | 1.13e-01 | 2 | 49 | 103 | 15137 | 4.08% | 0.68% | 1.94% | 0.32% | mature\_B\_cell\_down | Slc9a3r1, Tmem66 |
| GO:0051270 | regulation of cellular component movement | 4.42e-02 | 1.13e-01 | 4 | 49 | 413 | 15137 | 8.16% | 2.73% | 0.97% | 0.32% | mature\_B\_cell\_down | F2r, Gata3, Slc9a3r1, Thy1 |
| GO:0009891 | positive regulation of biosynthetic process | 4.43e-02 | 1.13e-01 | 8 | 49 | 1241 | 15137 | 16.33% | 8.20% | 0.64% | 0.32% | mature\_B\_cell\_down | Cd27, F2r, Gata3, Lpin1, Notch1, Prkcq, Skap1, Tnfrsf1a |
| GO:0002292 | T cell differentiation involved in immune response | 4.44e-02 | 1.13e-01 | 1 | 49 | 14 | 15137 | 2.04% | 0.09% | 7.14% | 0.32% | mature\_B\_cell\_down | Gata3 |
| GO:0002293 | alpha-beta T cell differentiation involved in immune response | 4.44e-02 | 1.13e-01 | 1 | 49 | 14 | 15137 | 2.04% | 0.09% | 7.14% | 0.32% | mature\_B\_cell\_down | Gata3 |
| GO:0006884 | cell volume homeostasis | 4.44e-02 | 1.13e-01 | 1 | 49 | 14 | 15137 | 2.04% | 0.09% | 7.14% | 0.32% | mature\_B\_cell\_down | Slc12a7 |
| GO:0006968 | cellular defense response | 4.44e-02 | 1.13e-01 | 1 | 49 | 14 | 15137 | 2.04% | 0.09% | 7.14% | 0.32% | mature\_B\_cell\_down | Lat |
| GO:0051482 | elevation of cytosolic calcium ion concentration involved in phospholipase C-activating G-protein coupled signaling pathway | 4.44e-02 | 1.13e-01 | 1 | 49 | 14 | 15137 | 2.04% | 0.09% | 7.14% | 0.32% | mature\_B\_cell\_down | F2r |
| GO:0060742 | epithelial cell differentiation involved in prostate gland development | 4.44e-02 | 1.13e-01 | 1 | 49 | 14 | 15137 | 2.04% | 0.09% | 7.14% | 0.32% | mature\_B\_cell\_down | Notch1 |
| GO:0035112 | genitalia morphogenesis | 4.44e-02 | 1.13e-01 | 1 | 49 | 14 | 15137 | 2.04% | 0.09% | 7.14% | 0.32% | mature\_B\_cell\_down | Tcf7 |
| GO:2001258 | negative regulation of cation channel activity | 4.44e-02 | 1.13e-01 | 1 | 49 | 14 | 15137 | 2.04% | 0.09% | 7.14% | 0.32% | mature\_B\_cell\_down | Tmem66 |
| GO:0045920 | negative regulation of exocytosis | 4.44e-02 | 1.13e-01 | 1 | 49 | 14 | 15137 | 2.04% | 0.09% | 7.14% | 0.32% | mature\_B\_cell\_down | Notch1 |
| GO:0018195 | peptidyl-arginine modification | 4.44e-02 | 1.13e-01 | 1 | 49 | 14 | 15137 | 2.04% | 0.09% | 7.14% | 0.32% | mature\_B\_cell\_down | Art2b |
| GO:0060037 | pharyngeal system development | 4.44e-02 | 1.13e-01 | 1 | 49 | 14 | 15137 | 2.04% | 0.09% | 7.14% | 0.32% | mature\_B\_cell\_down | Gata3 |
| GO:0014065 | phosphatidylinositol 3-kinase cascade | 4.44e-02 | 1.13e-01 | 1 | 49 | 14 | 15137 | 2.04% | 0.09% | 7.14% | 0.32% | mature\_B\_cell\_down | Gata3 |
| GO:0043368 | positive T cell selection | 4.44e-02 | 1.13e-01 | 1 | 49 | 14 | 15137 | 2.04% | 0.09% | 7.14% | 0.32% | mature\_B\_cell\_down | Zap70 |
| GO:0042523 | positive regulation of tyrosine phosphorylation of Stat5 protein | 4.44e-02 | 1.13e-01 | 1 | 49 | 14 | 15137 | 2.04% | 0.09% | 7.14% | 0.32% | mature\_B\_cell\_down | Lck |
| GO:0043618 | regulation of transcription from RNA polymerase II promoter in response to stress | 4.44e-02 | 1.13e-01 | 1 | 49 | 14 | 15137 | 2.04% | 0.09% | 7.14% | 0.32% | mature\_B\_cell\_down | Notch1 |
| GO:0031529 | ruffle organization | 4.44e-02 | 1.13e-01 | 1 | 49 | 14 | 15137 | 2.04% | 0.09% | 7.14% | 0.32% | mature\_B\_cell\_down | Lpin1 |
| GO:0042129 | regulation of T cell proliferation | 4.54e-02 | 1.15e-01 | 2 | 49 | 105 | 15137 | 4.08% | 0.69% | 1.90% | 0.32% | mature\_B\_cell\_down | Prkcq, Zap70 |
| GO:0008284 | positive regulation of cell proliferation | 4.62e-02 | 1.16e-01 | 5 | 49 | 609 | 15137 | 10.20% | 4.02% | 0.82% | 0.32% | mature\_B\_cell\_down | F2r, Notch1, Prkch, Prkcq, Zap70 |
| GO:0032270 | positive regulation of cellular protein metabolic process | 4.62e-02 | 1.16e-01 | 5 | 49 | 609 | 15137 | 10.20% | 4.02% | 0.82% | 0.32% | mature\_B\_cell\_down | F2r, Lck, Lpin1, Tnfrsf1a, Tnik |
| GO:0019725 | cellular homeostasis | 4.65e-02 | 1.16e-01 | 5 | 49 | 610 | 15137 | 10.20% | 4.03% | 0.82% | 0.32% | mature\_B\_cell\_down | F2r, Fyn, Lck, Slc12a7, Slc9a3r1 |
| GO:0010468 | regulation of gene expression | 4.65e-02 | 1.16e-01 | 15 | 49 | 2988 | 15137 | 30.61% | 19.74% | 0.50% | 0.32% | mature\_B\_cell\_down | F2r, Fyn, Gata3, Hnrpll, Lck, Lpin1, Nckap1, Notch1, Prkch, Prkcq, Skap1, Sla2, Tcf7, Tnfrsf1a, Txk |
| GO:0040012 | regulation of locomotion | 4.68e-02 | 1.16e-01 | 4 | 49 | 421 | 15137 | 8.16% | 2.78% | 0.95% | 0.32% | mature\_B\_cell\_down | F2r, Gata3, Slc9a3r1, Thy1 |
| GO:0002819 | regulation of adaptive immune response | 4.69e-02 | 1.16e-01 | 2 | 49 | 107 | 15137 | 4.08% | 0.71% | 1.87% | 0.32% | mature\_B\_cell\_down | Cd226, Prkcq |
| GO:0042100 | B cell proliferation | 4.75e-02 | 1.16e-01 | 1 | 49 | 15 | 15137 | 2.04% | 0.10% | 6.67% | 0.32% | mature\_B\_cell\_down | Cd27 |
| GO:0002287 | alpha-beta T cell activation involved in immune response | 4.75e-02 | 1.16e-01 | 1 | 49 | 15 | 15137 | 2.04% | 0.10% | 6.67% | 0.32% | mature\_B\_cell\_down | Gata3 |
| GO:0009713 | catechol-containing compound biosynthetic process | 4.75e-02 | 1.16e-01 | 1 | 49 | 15 | 15137 | 2.04% | 0.10% | 6.67% | 0.32% | mature\_B\_cell\_down | Gata3 |
| GO:0042423 | catecholamine biosynthetic process | 4.75e-02 | 1.16e-01 | 1 | 49 | 15 | 15137 | 2.04% | 0.10% | 6.67% | 0.32% | mature\_B\_cell\_down | Gata3 |
| GO:0030002 | cellular anion homeostasis | 4.75e-02 | 1.16e-01 | 1 | 49 | 15 | 15137 | 2.04% | 0.10% | 6.67% | 0.32% | mature\_B\_cell\_down | Slc9a3r1 |
| GO:0045123 | cellular extravasation | 4.75e-02 | 1.16e-01 | 1 | 49 | 15 | 15137 | 2.04% | 0.10% | 6.67% | 0.32% | mature\_B\_cell\_down | Selplg |
| GO:0034312 | diol biosynthetic process | 4.75e-02 | 1.16e-01 | 1 | 49 | 15 | 15137 | 2.04% | 0.10% | 6.67% | 0.32% | mature\_B\_cell\_down | Gata3 |
| GO:0046641 | positive regulation of alpha-beta T cell proliferation | 4.75e-02 | 1.16e-01 | 1 | 49 | 15 | 15137 | 2.04% | 0.10% | 6.67% | 0.32% | mature\_B\_cell\_down | Zap70 |
| GO:0032352 | positive regulation of hormone metabolic process | 4.75e-02 | 1.16e-01 | 1 | 49 | 15 | 15137 | 2.04% | 0.10% | 6.67% | 0.32% | mature\_B\_cell\_down | Gata3 |
| GO:0016925 | protein sumoylation | 4.75e-02 | 1.16e-01 | 1 | 49 | 15 | 15137 | 2.04% | 0.10% | 6.67% | 0.32% | mature\_B\_cell\_down | Ifih1 |
| GO:2000826 | regulation of heart morphogenesis | 4.75e-02 | 1.16e-01 | 1 | 49 | 15 | 15137 | 2.04% | 0.10% | 6.67% | 0.32% | mature\_B\_cell\_down | Notch1 |
| GO:0002837 | regulation of immune response to tumor cell | 4.75e-02 | 1.16e-01 | 1 | 49 | 15 | 15137 | 2.04% | 0.10% | 6.67% | 0.32% | mature\_B\_cell\_down | Cd226 |
| GO:0010830 | regulation of myotube differentiation | 4.75e-02 | 1.16e-01 | 1 | 49 | 15 | 15137 | 2.04% | 0.10% | 6.67% | 0.32% | mature\_B\_cell\_down | Notch1 |
| GO:0002834 | regulation of response to tumor cell | 4.75e-02 | 1.16e-01 | 1 | 49 | 15 | 15137 | 2.04% | 0.10% | 6.67% | 0.32% | mature\_B\_cell\_down | Cd226 |
| GO:2000677 | regulation of transcription regulatory region DNA binding | 4.75e-02 | 1.16e-01 | 1 | 49 | 15 | 15137 | 2.04% | 0.10% | 6.67% | 0.32% | mature\_B\_cell\_down | Gata3 |
| GO:0032494 | response to peptidoglycan | 4.75e-02 | 1.16e-01 | 1 | 49 | 15 | 15137 | 2.04% | 0.10% | 6.67% | 0.32% | mature\_B\_cell\_down | Notch1 |
| GO:0003205 | cardiac chamber development | 4.77e-02 | 1.16e-01 | 2 | 49 | 108 | 15137 | 4.08% | 0.71% | 1.85% | 0.32% | mature\_B\_cell\_down | Gata3, Notch1 |
| GO:0010952 | positive regulation of peptidase activity | 4.77e-02 | 1.16e-01 | 2 | 49 | 108 | 15137 | 4.08% | 0.71% | 1.85% | 0.32% | mature\_B\_cell\_down | F2r, Lck |
| GO:0042306 | regulation of protein import into nucleus | 4.77e-02 | 1.16e-01 | 2 | 49 | 108 | 15137 | 4.08% | 0.71% | 1.85% | 0.32% | mature\_B\_cell\_down | Prkcq, Tnfrsf1a |
| GO:1900180 | regulation of protein localization to nucleus | 4.77e-02 | 1.16e-01 | 2 | 49 | 108 | 15137 | 4.08% | 0.71% | 1.85% | 0.32% | mature\_B\_cell\_down | Prkcq, Tnfrsf1a |
| GO:0046578 | regulation of Ras protein signal transduction | 4.80e-02 | 1.17e-01 | 3 | 49 | 252 | 15137 | 6.12% | 1.66% | 1.19% | 0.32% | mature\_B\_cell\_down | F2r, Tbc1d2b, Thy1 |
| GO:0052548 | regulation of endopeptidase activity | 4.84e-02 | 1.18e-01 | 3 | 49 | 253 | 15137 | 6.12% | 1.67% | 1.19% | 0.32% | mature\_B\_cell\_down | Cd27, F2r, Lck |
| GO:0002250 | adaptive immune response | 4.85e-02 | 1.18e-01 | 2 | 49 | 109 | 15137 | 4.08% | 0.72% | 1.83% | 0.32% | mature\_B\_cell\_down | Itk, Txk |
| GO:0050767 | regulation of neurogenesis | 4.85e-02 | 1.18e-01 | 4 | 49 | 426 | 15137 | 8.16% | 2.81% | 0.94% | 0.32% | mature\_B\_cell\_down | Notch1, Prkch, Thy1, Tnik |
| GO:0042074 | cell migration involved in gastrulation | 5.06e-02 | 1.21e-01 | 1 | 49 | 16 | 15137 | 2.04% | 0.11% | 6.25% | 0.32% | mature\_B\_cell\_down | Nckap1 |
| GO:0072676 | lymphocyte migration | 5.06e-02 | 1.21e-01 | 1 | 49 | 16 | 15137 | 2.04% | 0.11% | 6.25% | 0.32% | mature\_B\_cell\_down | Gata3 |
| GO:0071599 | otic vesicle development | 5.06e-02 | 1.21e-01 | 1 | 49 | 16 | 15137 | 2.04% | 0.11% | 6.25% | 0.32% | mature\_B\_cell\_down | Gata3 |
| GO:0030194 | positive regulation of blood coagulation | 5.06e-02 | 1.21e-01 | 1 | 49 | 16 | 15137 | 2.04% | 0.11% | 6.25% | 0.32% | mature\_B\_cell\_down | F2r |
| GO:0051491 | positive regulation of filopodium assembly | 5.06e-02 | 1.21e-01 | 1 | 49 | 16 | 15137 | 2.04% | 0.11% | 6.25% | 0.32% | mature\_B\_cell\_down | Prkcq |
| GO:1900048 | positive regulation of hemostasis | 5.06e-02 | 1.21e-01 | 1 | 49 | 16 | 15137 | 2.04% | 0.11% | 6.25% | 0.32% | mature\_B\_cell\_down | F2r |
| GO:0002833 | positive regulation of response to biotic stimulus | 5.06e-02 | 1.21e-01 | 1 | 49 | 16 | 15137 | 2.04% | 0.11% | 6.25% | 0.32% | mature\_B\_cell\_down | Cd226 |
| GO:0033599 | regulation of mammary gland epithelial cell proliferation | 5.06e-02 | 1.21e-01 | 1 | 49 | 16 | 15137 | 2.04% | 0.11% | 6.25% | 0.32% | mature\_B\_cell\_down | Gata3 |
| GO:0070372 | regulation of ERK1 and ERK2 cascade | 5.18e-02 | 1.23e-01 | 2 | 49 | 113 | 15137 | 4.08% | 0.75% | 1.77% | 0.32% | mature\_B\_cell\_down | F2r, Slc9a3r1 |
| GO:0032409 | regulation of transporter activity | 5.18e-02 | 1.23e-01 | 2 | 49 | 113 | 15137 | 4.08% | 0.75% | 1.77% | 0.32% | mature\_B\_cell\_down | Slc9a3r1, Tmem66 |
| GO:0045859 | regulation of protein kinase activity | 5.35e-02 | 1.25e-01 | 4 | 49 | 440 | 15137 | 8.16% | 2.91% | 0.91% | 0.32% | mature\_B\_cell\_down | F2r, Slc9a3r1, Thy1, Tnik |
| GO:0019933 | cAMP-mediated signaling | 5.37e-02 | 1.25e-01 | 1 | 49 | 17 | 15137 | 2.04% | 0.11% | 5.88% | 0.32% | mature\_B\_cell\_down | Slc9a3r1 |
| GO:0048041 | focal adhesion assembly | 5.37e-02 | 1.25e-01 | 1 | 49 | 17 | 15137 | 2.04% | 0.11% | 5.88% | 0.32% | mature\_B\_cell\_down | Thy1 |
| GO:0048820 | hair follicle maturation | 5.37e-02 | 1.25e-01 | 1 | 49 | 17 | 15137 | 2.04% | 0.11% | 5.88% | 0.32% | mature\_B\_cell\_down | Notch1 |
| GO:2001015 | negative regulation of skeletal muscle cell differentiation | 5.37e-02 | 1.25e-01 | 1 | 49 | 17 | 15137 | 2.04% | 0.11% | 5.88% | 0.32% | mature\_B\_cell\_down | Notch1 |
| GO:0032728 | positive regulation of interferon-beta production | 5.37e-02 | 1.25e-01 | 1 | 49 | 17 | 15137 | 2.04% | 0.11% | 5.88% | 0.32% | mature\_B\_cell\_down | Ifih1 |
| GO:0043620 | regulation of DNA-dependent transcription in response to stress | 5.37e-02 | 1.25e-01 | 1 | 49 | 17 | 15137 | 2.04% | 0.11% | 5.88% | 0.32% | mature\_B\_cell\_down | Notch1 |
| GO:0051569 | regulation of histone H3-K4 methylation | 5.37e-02 | 1.25e-01 | 1 | 49 | 17 | 15137 | 2.04% | 0.11% | 5.88% | 0.32% | mature\_B\_cell\_down | Gata3 |
| GO:0034110 | regulation of homotypic cell-cell adhesion | 5.37e-02 | 1.25e-01 | 1 | 49 | 17 | 15137 | 2.04% | 0.11% | 5.88% | 0.32% | mature\_B\_cell\_down | Prkcq |
| GO:0032660 | regulation of interleukin-17 production | 5.37e-02 | 1.25e-01 | 1 | 49 | 17 | 15137 | 2.04% | 0.11% | 5.88% | 0.32% | mature\_B\_cell\_down | Prkcq |
| GO:2000736 | regulation of stem cell differentiation | 5.37e-02 | 1.25e-01 | 1 | 49 | 17 | 15137 | 2.04% | 0.11% | 5.88% | 0.32% | mature\_B\_cell\_down | Notch1 |
| GO:0042522 | regulation of tyrosine phosphorylation of Stat5 protein | 5.37e-02 | 1.25e-01 | 1 | 49 | 17 | 15137 | 2.04% | 0.11% | 5.88% | 0.32% | mature\_B\_cell\_down | Lck |
| GO:0003071 | renal system process involved in regulation of systemic arterial blood pressure | 5.37e-02 | 1.25e-01 | 1 | 49 | 17 | 15137 | 2.04% | 0.11% | 5.88% | 0.32% | mature\_B\_cell\_down | F2r |
| GO:0048513 | organ development | 5.37e-02 | 1.25e-01 | 11 | 49 | 2013 | 15137 | 22.45% | 13.30% | 0.55% | 0.32% | mature\_B\_cell\_down | F2r, Fyn, Gata3, Itk, Lck, Nckap1, Notch1, Tcf7, Thy1, Txk, Zap70 |
| GO:0001838 | embryonic epithelial tube formation | 5.42e-02 | 1.26e-01 | 2 | 49 | 116 | 15137 | 4.08% | 0.77% | 1.72% | 0.32% | mature\_B\_cell\_down | Gata3, Nckap1 |
| GO:0072175 | epithelial tube formation | 5.42e-02 | 1.26e-01 | 2 | 49 | 116 | 15137 | 4.08% | 0.77% | 1.72% | 0.32% | mature\_B\_cell\_down | Gata3, Nckap1 |
| GO:0030336 | negative regulation of cell migration | 5.42e-02 | 1.26e-01 | 2 | 49 | 116 | 15137 | 4.08% | 0.77% | 1.72% | 0.32% | mature\_B\_cell\_down | Slc9a3r1, Thy1 |
| GO:0006950 | response to stress | 5.52e-02 | 1.28e-01 | 10 | 49 | 1775 | 15137 | 20.41% | 11.73% | 0.56% | 0.32% | mature\_B\_cell\_down | Ctsd, F2r, Ifi47, Ifih1, Lat, Lck, Notch1, Prkcq, Tnfrsf1a, Tnik |
| GO:0009894 | regulation of catabolic process | 5.53e-02 | 1.28e-01 | 4 | 49 | 445 | 15137 | 8.16% | 2.94% | 0.90% | 0.32% | mature\_B\_cell\_down | Fyn, Ipcef1, Tbc1d2b, Thy1 |
| GO:0043367 | CD4-positive, alpha-beta T cell differentiation | 5.67e-02 | 1.28e-01 | 1 | 49 | 18 | 15137 | 2.04% | 0.12% | 5.56% | 0.32% | mature\_B\_cell\_down | Gata3 |
| GO:0002437 | inflammatory response to antigenic stimulus | 5.67e-02 | 1.28e-01 | 1 | 49 | 18 | 15137 | 2.04% | 0.12% | 5.56% | 0.32% | mature\_B\_cell\_down | Notch1 |
| GO:0043299 | leukocyte degranulation | 5.67e-02 | 1.28e-01 | 1 | 49 | 18 | 15137 | 2.04% | 0.12% | 5.56% | 0.32% | mature\_B\_cell\_down | Lat |
| GO:0006509 | membrane protein ectodomain proteolysis | 5.67e-02 | 1.28e-01 | 1 | 49 | 18 | 15137 | 2.04% | 0.12% | 5.56% | 0.32% | mature\_B\_cell\_down | Prkcq |
| GO:2001234 | negative regulation of apoptotic signaling pathway | 5.67e-02 | 1.28e-01 | 1 | 49 | 18 | 15137 | 2.04% | 0.12% | 5.56% | 0.32% | mature\_B\_cell\_down | Fyn |
| GO:0051898 | negative regulation of protein kinase B signaling cascade | 5.67e-02 | 1.28e-01 | 1 | 49 | 18 | 15137 | 2.04% | 0.12% | 5.56% | 0.32% | mature\_B\_cell\_down | Slc9a3r1 |
| GO:0030903 | notochord development | 5.67e-02 | 1.28e-01 | 1 | 49 | 18 | 15137 | 2.04% | 0.12% | 5.56% | 0.32% | mature\_B\_cell\_down | Nckap1 |
| GO:0042346 | positive regulation of NF-kappaB import into nucleus | 5.67e-02 | 1.28e-01 | 1 | 49 | 18 | 15137 | 2.04% | 0.12% | 5.56% | 0.32% | mature\_B\_cell\_down | Prkcq |
| GO:0032967 | positive regulation of collagen biosynthetic process | 5.67e-02 | 1.28e-01 | 1 | 49 | 18 | 15137 | 2.04% | 0.12% | 5.56% | 0.32% | mature\_B\_cell\_down | F2r |
| GO:0010714 | positive regulation of collagen metabolic process | 5.67e-02 | 1.28e-01 | 1 | 49 | 18 | 15137 | 2.04% | 0.12% | 5.56% | 0.32% | mature\_B\_cell\_down | F2r |
| GO:0006471 | protein ADP-ribosylation | 5.67e-02 | 1.28e-01 | 1 | 49 | 18 | 15137 | 2.04% | 0.12% | 5.56% | 0.32% | mature\_B\_cell\_down | Art2b |
| GO:0070232 | regulation of T cell apoptotic process | 5.67e-02 | 1.28e-01 | 1 | 49 | 18 | 15137 | 2.04% | 0.12% | 5.56% | 0.32% | mature\_B\_cell\_down | Prkcq |
| GO:0045076 | regulation of interleukin-2 biosynthetic process | 5.67e-02 | 1.28e-01 | 1 | 49 | 18 | 15137 | 2.04% | 0.12% | 5.56% | 0.32% | mature\_B\_cell\_down | Prkcq |
| GO:0010743 | regulation of macrophage derived foam cell differentiation | 5.67e-02 | 1.28e-01 | 1 | 49 | 18 | 15137 | 2.04% | 0.12% | 5.56% | 0.32% | mature\_B\_cell\_down | Prkch |
| GO:0090311 | regulation of protein deacetylation | 5.67e-02 | 1.28e-01 | 1 | 49 | 18 | 15137 | 2.04% | 0.12% | 5.56% | 0.32% | mature\_B\_cell\_down | Lpin1 |
| GO:0048103 | somatic stem cell division | 5.67e-02 | 1.28e-01 | 1 | 49 | 18 | 15137 | 2.04% | 0.12% | 5.56% | 0.32% | mature\_B\_cell\_down | Notch1 |
| GO:0048488 | synaptic vesicle endocytosis | 5.67e-02 | 1.28e-01 | 1 | 49 | 18 | 15137 | 2.04% | 0.12% | 5.56% | 0.32% | mature\_B\_cell\_down | Zdhhc15 |
| GO:0006904 | vesicle docking involved in exocytosis | 5.67e-02 | 1.28e-01 | 1 | 49 | 18 | 15137 | 2.04% | 0.12% | 5.56% | 0.32% | mature\_B\_cell\_down | Sytl2 |
| GO:0048871 | multicellular organismal homeostasis | 5.67e-02 | 1.28e-01 | 2 | 49 | 119 | 15137 | 4.08% | 0.79% | 1.68% | 0.32% | mature\_B\_cell\_down | Cd7, F2r |
| GO:0010628 | positive regulation of gene expression | 5.71e-02 | 1.28e-01 | 7 | 49 | 1078 | 15137 | 14.29% | 7.12% | 0.65% | 0.32% | mature\_B\_cell\_down | F2r, Gata3, Lck, Lpin1, Notch1, Skap1, Tnfrsf1a |
| GO:0008285 | negative regulation of cell proliferation | 5.76e-02 | 1.29e-01 | 4 | 49 | 451 | 15137 | 8.16% | 2.98% | 0.89% | 0.32% | mature\_B\_cell\_down | F2r, Gata3, Notch1, Slc9a3r1 |
| GO:0031344 | regulation of cell projection organization | 5.78e-02 | 1.29e-01 | 3 | 49 | 272 | 15137 | 6.12% | 1.80% | 1.10% | 0.32% | mature\_B\_cell\_down | Prkcq, Thy1, Tnik |
| GO:0001934 | positive regulation of protein phosphorylation | 5.87e-02 | 1.31e-01 | 4 | 49 | 454 | 15137 | 8.16% | 3.00% | 0.88% | 0.32% | mature\_B\_cell\_down | F2r, Lck, Tnfrsf1a, Tnik |
| GO:0050731 | positive regulation of peptidyl-tyrosine phosphorylation | 5.93e-02 | 1.32e-01 | 2 | 49 | 122 | 15137 | 4.08% | 0.81% | 1.64% | 0.32% | mature\_B\_cell\_down | Lck, Tnfrsf1a |
| GO:0035710 | CD4-positive, alpha-beta T cell activation | 5.98e-02 | 1.32e-01 | 1 | 49 | 19 | 15137 | 2.04% | 0.13% | 5.26% | 0.32% | mature\_B\_cell\_down | Gata3 |
| GO:0032715 | negative regulation of interleukin-6 production | 5.98e-02 | 1.32e-01 | 1 | 49 | 19 | 15137 | 2.04% | 0.13% | 5.26% | 0.32% | mature\_B\_cell\_down | Tnfrsf1a |
| GO:0050820 | positive regulation of coagulation | 5.98e-02 | 1.32e-01 | 1 | 49 | 19 | 15137 | 2.04% | 0.13% | 5.26% | 0.32% | mature\_B\_cell\_down | F2r |
| GO:0033158 | regulation of protein import into nucleus, translocation | 5.98e-02 | 1.32e-01 | 1 | 49 | 19 | 15137 | 2.04% | 0.13% | 5.26% | 0.32% | mature\_B\_cell\_down | Tnfrsf1a |
| GO:0048485 | sympathetic nervous system development | 5.98e-02 | 1.32e-01 | 1 | 49 | 19 | 15137 | 2.04% | 0.13% | 5.26% | 0.32% | mature\_B\_cell\_down | Gata3 |
| GO:0033209 | tumor necrosis factor-mediated signaling pathway | 5.98e-02 | 1.32e-01 | 1 | 49 | 19 | 15137 | 2.04% | 0.13% | 5.26% | 0.32% | mature\_B\_cell\_down | Tnfrsf1a |
| GO:0035148 | tube formation | 6.01e-02 | 1.33e-01 | 2 | 49 | 123 | 15137 | 4.08% | 0.81% | 1.63% | 0.32% | mature\_B\_cell\_down | Gata3, Nckap1 |
| GO:0051056 | regulation of small GTPase mediated signal transduction | 6.03e-02 | 1.33e-01 | 3 | 49 | 277 | 15137 | 6.12% | 1.83% | 1.08% | 0.32% | mature\_B\_cell\_down | F2r, Tbc1d2b, Thy1 |
| GO:0048519 | negative regulation of biological process | 6.23e-02 | 1.36e-01 | 14 | 49 | 2839 | 15137 | 28.57% | 18.76% | 0.49% | 0.32% | mature\_B\_cell\_down | Cd27, F2r, Fyn, Gata3, Lck, Lpin1, Notch1, Prkch, Prkcq, Sla2, Slc9a3r1, Thy1, Tmem66, Tnfrsf1a |
| GO:0046717 | acid secretion | 6.28e-02 | 1.36e-01 | 1 | 49 | 20 | 15137 | 2.04% | 0.13% | 5.00% | 0.32% | mature\_B\_cell\_down | Slc9a3r1 |
| GO:0048557 | embryonic digestive tract morphogenesis | 6.28e-02 | 1.36e-01 | 1 | 49 | 20 | 15137 | 2.04% | 0.13% | 5.00% | 0.32% | mature\_B\_cell\_down | Tcf7 |
| GO:0048339 | paraxial mesoderm development | 6.28e-02 | 1.36e-01 | 1 | 49 | 20 | 15137 | 2.04% | 0.13% | 5.00% | 0.32% | mature\_B\_cell\_down | Nckap1 |
| GO:0002714 | positive regulation of B cell mediated immunity | 6.28e-02 | 1.36e-01 | 1 | 49 | 20 | 15137 | 2.04% | 0.13% | 5.00% | 0.32% | mature\_B\_cell\_down | Cd226 |
| GO:0002891 | positive regulation of immunoglobulin mediated immune response | 6.28e-02 | 1.36e-01 | 1 | 49 | 20 | 15137 | 2.04% | 0.13% | 5.00% | 0.32% | mature\_B\_cell\_down | Cd226 |
| GO:0045954 | positive regulation of natural killer cell mediated cytotoxicity | 6.28e-02 | 1.36e-01 | 1 | 49 | 20 | 15137 | 2.04% | 0.13% | 5.00% | 0.32% | mature\_B\_cell\_down | Cd226 |
| GO:0002717 | positive regulation of natural killer cell mediated immunity | 6.28e-02 | 1.36e-01 | 1 | 49 | 20 | 15137 | 2.04% | 0.13% | 5.00% | 0.32% | mature\_B\_cell\_down | Cd226 |
| GO:0002052 | positive regulation of neuroblast proliferation | 6.28e-02 | 1.36e-01 | 1 | 49 | 20 | 15137 | 2.04% | 0.13% | 5.00% | 0.32% | mature\_B\_cell\_down | Notch1 |
| GO:0046640 | regulation of alpha-beta T cell proliferation | 6.28e-02 | 1.36e-01 | 1 | 49 | 20 | 15137 | 2.04% | 0.13% | 5.00% | 0.32% | mature\_B\_cell\_down | Zap70 |
| GO:1901019 | regulation of calcium ion transmembrane transporter activity | 6.28e-02 | 1.36e-01 | 1 | 49 | 20 | 15137 | 2.04% | 0.13% | 5.00% | 0.32% | mature\_B\_cell\_down | Tmem66 |
| GO:0010543 | regulation of platelet activation | 6.28e-02 | 1.36e-01 | 1 | 49 | 20 | 15137 | 2.04% | 0.13% | 5.00% | 0.32% | mature\_B\_cell\_down | Prkcq |
| GO:0048278 | vesicle docking | 6.28e-02 | 1.36e-01 | 1 | 49 | 20 | 15137 | 2.04% | 0.13% | 5.00% | 0.32% | mature\_B\_cell\_down | Sytl2 |
| GO:0031349 | positive regulation of defense response | 6.36e-02 | 1.37e-01 | 2 | 49 | 127 | 15137 | 4.08% | 0.84% | 1.57% | 0.32% | mature\_B\_cell\_down | Cd226, Tnfrsf1a |
| GO:0048667 | cell morphogenesis involved in neuron differentiation | 6.40e-02 | 1.38e-01 | 3 | 49 | 284 | 15137 | 6.12% | 1.88% | 1.06% | 0.32% | mature\_B\_cell\_down | Fyn, Gata3, Notch1 |
| GO:0051707 | response to other organism | 6.42e-02 | 1.38e-01 | 4 | 49 | 468 | 15137 | 8.16% | 3.09% | 0.85% | 0.32% | mature\_B\_cell\_down | F2r, Ifih1, Notch1, Tnfrsf1a |
| GO:2000027 | regulation of organ morphogenesis | 6.45e-02 | 1.39e-01 | 2 | 49 | 128 | 15137 | 4.08% | 0.85% | 1.56% | 0.32% | mature\_B\_cell\_down | Gata3, Notch1 |
| GO:0006915 | apoptotic process | 6.48e-02 | 1.39e-01 | 5 | 49 | 671 | 15137 | 10.20% | 4.43% | 0.75% | 0.32% | mature\_B\_cell\_down | Cd27, F2r, Lck, Slc9a3r1, Tnfrsf1a |
| GO:0042327 | positive regulation of phosphorylation | 6.59e-02 | 1.40e-01 | 4 | 49 | 472 | 15137 | 8.16% | 3.12% | 0.85% | 0.32% | mature\_B\_cell\_down | F2r, Lck, Tnfrsf1a, Tnik |
| GO:0051960 | regulation of nervous system development | 6.59e-02 | 1.40e-01 | 4 | 49 | 472 | 15137 | 8.16% | 3.12% | 0.85% | 0.32% | mature\_B\_cell\_down | Notch1, Prkch, Thy1, Tnik |
| GO:0055081 | anion homeostasis | 6.59e-02 | 1.40e-01 | 1 | 49 | 21 | 15137 | 2.04% | 0.14% | 4.76% | 0.32% | mature\_B\_cell\_down | Slc9a3r1 |
| GO:0019935 | cyclic-nucleotide-mediated signaling | 6.59e-02 | 1.40e-01 | 1 | 49 | 21 | 15137 | 2.04% | 0.14% | 4.76% | 0.32% | mature\_B\_cell\_down | Slc9a3r1 |
| GO:0030032 | lamellipodium assembly | 6.59e-02 | 1.40e-01 | 1 | 49 | 21 | 15137 | 2.04% | 0.14% | 4.76% | 0.32% | mature\_B\_cell\_down | Nckap1 |
| GO:0022406 | membrane docking | 6.59e-02 | 1.40e-01 | 1 | 49 | 21 | 15137 | 2.04% | 0.14% | 4.76% | 0.32% | mature\_B\_cell\_down | Sytl2 |
| GO:0070373 | negative regulation of ERK1 and ERK2 cascade | 6.59e-02 | 1.40e-01 | 1 | 49 | 21 | 15137 | 2.04% | 0.14% | 4.76% | 0.32% | mature\_B\_cell\_down | Slc9a3r1 |
| GO:0070229 | negative regulation of lymphocyte apoptotic process | 6.59e-02 | 1.40e-01 | 1 | 49 | 21 | 15137 | 2.04% | 0.14% | 4.76% | 0.32% | mature\_B\_cell\_down | Prkcq |
| GO:2000516 | positive regulation of CD4-positive, alpha-beta T cell activation | 6.59e-02 | 1.40e-01 | 1 | 49 | 21 | 15137 | 2.04% | 0.14% | 4.76% | 0.32% | mature\_B\_cell\_down | Prkcq |
| GO:0010718 | positive regulation of epithelial to mesenchymal transition | 6.59e-02 | 1.40e-01 | 1 | 49 | 21 | 15137 | 2.04% | 0.14% | 4.76% | 0.32% | mature\_B\_cell\_down | Notch1 |
| GO:0046822 | regulation of nucleocytoplasmic transport | 6.63e-02 | 1.40e-01 | 2 | 49 | 130 | 15137 | 4.08% | 0.86% | 1.54% | 0.32% | mature\_B\_cell\_down | Prkcq, Tnfrsf1a |
| GO:0043549 | regulation of kinase activity | 6.63e-02 | 1.40e-01 | 4 | 49 | 473 | 15137 | 8.16% | 3.12% | 0.85% | 0.32% | mature\_B\_cell\_down | F2r, Slc9a3r1, Thy1, Tnik |
| GO:0034097 | response to cytokine stimulus | 6.68e-02 | 1.41e-01 | 3 | 49 | 289 | 15137 | 6.12% | 1.91% | 1.04% | 0.32% | mature\_B\_cell\_down | Gata3, Tcf7, Tnfrsf1a |
| GO:0035239 | tube morphogenesis | 6.73e-02 | 1.42e-01 | 3 | 49 | 290 | 15137 | 6.12% | 1.92% | 1.03% | 0.32% | mature\_B\_cell\_down | Gata3, Nckap1, Notch1 |
| GO:0016477 | cell migration | 6.87e-02 | 1.42e-01 | 4 | 49 | 479 | 15137 | 8.16% | 3.16% | 0.84% | 0.32% | mature\_B\_cell\_down | Fyn, Gata3, Nckap1, Selplg |
| GO:0045937 | positive regulation of phosphate metabolic process | 6.87e-02 | 1.42e-01 | 4 | 49 | 479 | 15137 | 8.16% | 3.16% | 0.84% | 0.32% | mature\_B\_cell\_down | F2r, Lck, Tnfrsf1a, Tnik |
| GO:0010562 | positive regulation of phosphorus metabolic process | 6.87e-02 | 1.42e-01 | 4 | 49 | 479 | 15137 | 8.16% | 3.16% | 0.84% | 0.32% | mature\_B\_cell\_down | F2r, Lck, Tnfrsf1a, Tnik |
| GO:0000045 | autophagic vacuole assembly | 6.89e-02 | 1.42e-01 | 1 | 49 | 22 | 15137 | 2.04% | 0.15% | 4.55% | 0.32% | mature\_B\_cell\_down | Ctsd |
| GO:0002065 | columnar/cuboidal epithelial cell differentiation | 6.89e-02 | 1.42e-01 | 1 | 49 | 22 | 15137 | 2.04% | 0.15% | 4.55% | 0.32% | mature\_B\_cell\_down | Notch1 |
| GO:0050974 | detection of mechanical stimulus involved in sensory perception | 6.89e-02 | 1.42e-01 | 1 | 49 | 22 | 15137 | 2.04% | 0.15% | 4.55% | 0.32% | mature\_B\_cell\_down | Fyn |
| GO:0033619 | membrane protein proteolysis | 6.89e-02 | 1.42e-01 | 1 | 49 | 22 | 15137 | 2.04% | 0.15% | 4.55% | 0.32% | mature\_B\_cell\_down | Prkcq |
| GO:1900077 | negative regulation of cellular response to insulin stimulus | 6.89e-02 | 1.42e-01 | 1 | 49 | 22 | 15137 | 2.04% | 0.15% | 4.55% | 0.32% | mature\_B\_cell\_down | Prkcq |
| GO:0046627 | negative regulation of insulin receptor signaling pathway | 6.89e-02 | 1.42e-01 | 1 | 49 | 22 | 15137 | 2.04% | 0.15% | 4.55% | 0.32% | mature\_B\_cell\_down | Prkcq |
| GO:0032689 | negative regulation of interferon-gamma production | 6.89e-02 | 1.42e-01 | 1 | 49 | 22 | 15137 | 2.04% | 0.15% | 4.55% | 0.32% | mature\_B\_cell\_down | Gata3 |
| GO:0051154 | negative regulation of striated muscle cell differentiation | 6.89e-02 | 1.42e-01 | 1 | 49 | 22 | 15137 | 2.04% | 0.15% | 4.55% | 0.32% | mature\_B\_cell\_down | Notch1 |
| GO:0032481 | positive regulation of type I interferon production | 6.89e-02 | 1.42e-01 | 1 | 49 | 22 | 15137 | 2.04% | 0.15% | 4.55% | 0.32% | mature\_B\_cell\_down | Ifih1 |
| GO:0045055 | regulated secretory pathway | 6.89e-02 | 1.42e-01 | 1 | 49 | 22 | 15137 | 2.04% | 0.15% | 4.55% | 0.32% | mature\_B\_cell\_down | Lat |
| GO:0032965 | regulation of collagen biosynthetic process | 6.89e-02 | 1.42e-01 | 1 | 49 | 22 | 15137 | 2.04% | 0.15% | 4.55% | 0.32% | mature\_B\_cell\_down | F2r |
| GO:0010712 | regulation of collagen metabolic process | 6.89e-02 | 1.42e-01 | 1 | 49 | 22 | 15137 | 2.04% | 0.15% | 4.55% | 0.32% | mature\_B\_cell\_down | F2r |
| GO:0044062 | regulation of excretion | 6.89e-02 | 1.42e-01 | 1 | 49 | 22 | 15137 | 2.04% | 0.15% | 4.55% | 0.32% | mature\_B\_cell\_down | Slc9a3r1 |
| GO:0007243 | intracellular protein kinase cascade | 6.90e-02 | 1.42e-01 | 3 | 49 | 293 | 15137 | 6.12% | 1.94% | 1.02% | 0.32% | mature\_B\_cell\_down | F2r, Prkch, Tnik |
| GO:0012501 | programmed cell death | 6.92e-02 | 1.42e-01 | 5 | 49 | 684 | 15137 | 10.20% | 4.52% | 0.73% | 0.32% | mature\_B\_cell\_down | Cd27, F2r, Lck, Slc9a3r1, Tnfrsf1a |
| GO:0060249 | anatomical structure homeostasis | 6.99e-02 | 1.43e-01 | 2 | 49 | 134 | 15137 | 4.08% | 0.89% | 1.49% | 0.32% | mature\_B\_cell\_down | Cd7, F2r |
| GO:0006887 | exocytosis | 6.99e-02 | 1.43e-01 | 2 | 49 | 134 | 15137 | 4.08% | 0.89% | 1.49% | 0.32% | mature\_B\_cell\_down | Lat, Sytl2 |
| GO:0033157 | regulation of intracellular protein transport | 7.08e-02 | 1.45e-01 | 2 | 49 | 135 | 15137 | 4.08% | 0.89% | 1.48% | 0.32% | mature\_B\_cell\_down | Prkcq, Tnfrsf1a |
| GO:0048812 | neuron projection morphogenesis | 7.12e-02 | 1.45e-01 | 3 | 49 | 297 | 15137 | 6.12% | 1.96% | 1.01% | 0.32% | mature\_B\_cell\_down | Fyn, Gata3, Notch1 |
| GO:0007204 | elevation of cytosolic calcium ion concentration | 7.17e-02 | 1.45e-01 | 2 | 49 | 136 | 15137 | 4.08% | 0.90% | 1.47% | 0.32% | mature\_B\_cell\_down | F2r, Lck |
| GO:0051048 | negative regulation of secretion | 7.17e-02 | 1.45e-01 | 2 | 49 | 136 | 15137 | 4.08% | 0.90% | 1.47% | 0.32% | mature\_B\_cell\_down | F2r, Notch1 |
| GO:0007399 | nervous system development | 7.18e-02 | 1.45e-01 | 8 | 49 | 1372 | 15137 | 16.33% | 9.06% | 0.58% | 0.32% | mature\_B\_cell\_down | Fyn, Gata3, Nckap1, Notch1, Prkch, Tcf7, Thy1, Tnik |
| GO:0006693 | prostaglandin metabolic process | 7.19e-02 | 1.45e-01 | 1 | 49 | 23 | 15137 | 2.04% | 0.15% | 4.35% | 0.32% | mature\_B\_cell\_down | Tnfrsf1a |
| GO:0006692 | prostanoid metabolic process | 7.19e-02 | 1.45e-01 | 1 | 49 | 23 | 15137 | 2.04% | 0.15% | 4.35% | 0.32% | mature\_B\_cell\_down | Tnfrsf1a |
| GO:0033238 | regulation of cellular amine metabolic process | 7.19e-02 | 1.45e-01 | 1 | 49 | 23 | 15137 | 2.04% | 0.15% | 4.35% | 0.32% | mature\_B\_cell\_down | Gata3 |
| GO:0051489 | regulation of filopodium assembly | 7.19e-02 | 1.45e-01 | 1 | 49 | 23 | 15137 | 2.04% | 0.15% | 4.35% | 0.32% | mature\_B\_cell\_down | Prkcq |
| GO:0031060 | regulation of histone methylation | 7.19e-02 | 1.45e-01 | 1 | 49 | 23 | 15137 | 2.04% | 0.15% | 4.35% | 0.32% | mature\_B\_cell\_down | Gata3 |
| GO:0032648 | regulation of interferon-beta production | 7.19e-02 | 1.45e-01 | 1 | 49 | 23 | 15137 | 2.04% | 0.15% | 4.35% | 0.32% | mature\_B\_cell\_down | Ifih1 |
| GO:0050789 | regulation of biological process | 7.19e-02 | 1.45e-01 | 34 | 49 | 8805 | 15137 | 69.39% | 58.17% | 0.39% | 0.32% | mature\_B\_cell\_down | Apol7b, Cd226, Cd247, Cd27, Cd3g, F2r, Fyn, Gata3, Hnrpll, Ifih1, Ipcef1, Itk, Lat, Lck, Lpin1, Nckap1, Notch1, Prkch, Prkcq, Rab37, Rinl, Skap1, Sla2, Slc9a3r1, Stk39, Sytl2, Tbc1d2b, Tcf7, Thy1, Tmem66, Tnfrsf1a, Tnik, Txk, Zap70 |
| GO:0048666 | neuron development | 7.26e-02 | 1.46e-01 | 4 | 49 | 488 | 15137 | 8.16% | 3.22% | 0.82% | 0.32% | mature\_B\_cell\_down | Fyn, Gata3, Notch1, Thy1 |
| GO:0035023 | regulation of Rho protein signal transduction | 7.26e-02 | 1.46e-01 | 2 | 49 | 137 | 15137 | 4.08% | 0.91% | 1.46% | 0.32% | mature\_B\_cell\_down | F2r, Thy1 |
| GO:0030031 | cell projection assembly | 7.44e-02 | 1.49e-01 | 2 | 49 | 139 | 15137 | 4.08% | 0.92% | 1.44% | 0.32% | mature\_B\_cell\_down | Nckap1, Slc9a3r1 |
| GO:0051338 | regulation of transferase activity | 7.47e-02 | 1.49e-01 | 4 | 49 | 493 | 15137 | 8.16% | 3.26% | 0.81% | 0.32% | mature\_B\_cell\_down | F2r, Slc9a3r1, Thy1, Tnik |
| GO:0009607 | response to biotic stimulus | 7.47e-02 | 1.49e-01 | 4 | 49 | 493 | 15137 | 8.16% | 3.26% | 0.81% | 0.32% | mature\_B\_cell\_down | F2r, Ifih1, Notch1, Tnfrsf1a |
| GO:0035162 | embryonic hemopoiesis | 7.49e-02 | 1.49e-01 | 1 | 49 | 24 | 15137 | 2.04% | 0.16% | 4.17% | 0.32% | mature\_B\_cell\_down | Gata3 |
| GO:0050850 | positive regulation of calcium-mediated signaling | 7.49e-02 | 1.49e-01 | 1 | 49 | 24 | 15137 | 2.04% | 0.16% | 4.17% | 0.32% | mature\_B\_cell\_down | Zap70 |
| GO:0044253 | positive regulation of multicellular organismal metabolic process | 7.49e-02 | 1.49e-01 | 1 | 49 | 24 | 15137 | 2.04% | 0.16% | 4.17% | 0.32% | mature\_B\_cell\_down | F2r |
| GO:0051496 | positive regulation of stress fiber assembly | 7.49e-02 | 1.49e-01 | 1 | 49 | 24 | 15137 | 2.04% | 0.16% | 4.17% | 0.32% | mature\_B\_cell\_down | Prkcq |
| GO:0040036 | regulation of fibroblast growth factor receptor signaling pathway | 7.49e-02 | 1.49e-01 | 1 | 49 | 24 | 15137 | 2.04% | 0.16% | 4.17% | 0.32% | mature\_B\_cell\_down | Gata3 |
| GO:0045661 | regulation of myoblast differentiation | 7.49e-02 | 1.49e-01 | 1 | 49 | 24 | 15137 | 2.04% | 0.16% | 4.17% | 0.32% | mature\_B\_cell\_down | Notch1 |
| GO:0016331 | morphogenesis of embryonic epithelium | 7.54e-02 | 1.50e-01 | 2 | 49 | 140 | 15137 | 4.08% | 0.92% | 1.43% | 0.32% | mature\_B\_cell\_down | Gata3, Nckap1 |
| GO:0050769 | positive regulation of neurogenesis | 7.63e-02 | 1.51e-01 | 2 | 49 | 141 | 15137 | 4.08% | 0.93% | 1.42% | 0.32% | mature\_B\_cell\_down | Notch1, Prkch |
| GO:0045944 | positive regulation of transcription from RNA polymerase II promoter | 7.73e-02 | 1.53e-01 | 5 | 49 | 707 | 15137 | 10.20% | 4.67% | 0.71% | 0.32% | mature\_B\_cell\_down | Gata3, Lpin1, Notch1, Skap1, Tnfrsf1a |
| GO:0048598 | embryonic morphogenesis | 7.74e-02 | 1.53e-01 | 4 | 49 | 499 | 15137 | 8.16% | 3.30% | 0.80% | 0.32% | mature\_B\_cell\_down | Gata3, Nckap1, Notch1, Tcf7 |
| GO:1900542 | regulation of purine nucleotide metabolic process | 7.75e-02 | 1.53e-01 | 3 | 49 | 308 | 15137 | 6.12% | 2.03% | 0.97% | 0.32% | mature\_B\_cell\_down | Ipcef1, Tbc1d2b, Thy1 |
| GO:0009887 | organ morphogenesis | 7.77e-02 | 1.53e-01 | 5 | 49 | 708 | 15137 | 10.20% | 4.68% | 0.71% | 0.32% | mature\_B\_cell\_down | Gata3, Nckap1, Notch1, Tcf7, Thy1 |
| GO:0048708 | astrocyte differentiation | 7.79e-02 | 1.53e-01 | 1 | 49 | 25 | 15137 | 2.04% | 0.17% | 4.00% | 0.32% | mature\_B\_cell\_down | Notch1 |
| GO:0007212 | dopamine receptor signaling pathway | 7.79e-02 | 1.53e-01 | 1 | 49 | 25 | 15137 | 2.04% | 0.17% | 4.00% | 0.32% | mature\_B\_cell\_down | Slc9a3r1 |
| GO:0045686 | negative regulation of glial cell differentiation | 7.79e-02 | 1.53e-01 | 1 | 49 | 25 | 15137 | 2.04% | 0.17% | 4.00% | 0.32% | mature\_B\_cell\_down | Notch1 |
| GO:0010389 | regulation of G2/M transition of mitotic cell cycle | 7.79e-02 | 1.53e-01 | 1 | 49 | 25 | 15137 | 2.04% | 0.17% | 4.00% | 0.32% | mature\_B\_cell\_down | Prkcq |
| GO:0048710 | regulation of astrocyte differentiation | 7.79e-02 | 1.53e-01 | 1 | 49 | 25 | 15137 | 2.04% | 0.17% | 4.00% | 0.32% | mature\_B\_cell\_down | Notch1 |
| GO:0043266 | regulation of potassium ion transport | 7.79e-02 | 1.53e-01 | 1 | 49 | 25 | 15137 | 2.04% | 0.17% | 4.00% | 0.32% | mature\_B\_cell\_down | Stk39 |
| GO:0006140 | regulation of nucleotide metabolic process | 7.99e-02 | 1.56e-01 | 3 | 49 | 312 | 15137 | 6.12% | 2.06% | 0.96% | 0.32% | mature\_B\_cell\_down | Ipcef1, Tbc1d2b, Thy1 |
| GO:0010629 | negative regulation of gene expression | 8.04e-02 | 1.56e-01 | 6 | 49 | 937 | 15137 | 12.24% | 6.19% | 0.64% | 0.32% | mature\_B\_cell\_down | Fyn, Gata3, Lpin1, Notch1, Sla2, Tnfrsf1a |
| GO:0002286 | T cell activation involved in immune response | 8.09e-02 | 1.56e-01 | 1 | 49 | 26 | 15137 | 2.04% | 0.17% | 3.85% | 0.32% | mature\_B\_cell\_down | Gata3 |
| GO:0050982 | detection of mechanical stimulus | 8.09e-02 | 1.56e-01 | 1 | 49 | 26 | 15137 | 2.04% | 0.17% | 3.85% | 0.32% | mature\_B\_cell\_down | Fyn |
| GO:0003382 | epithelial cell morphogenesis | 8.09e-02 | 1.56e-01 | 1 | 49 | 26 | 15137 | 2.04% | 0.17% | 3.85% | 0.32% | mature\_B\_cell\_down | Gata3 |
| GO:0002244 | hemopoietic progenitor cell differentiation | 8.09e-02 | 1.56e-01 | 1 | 49 | 26 | 15137 | 2.04% | 0.17% | 3.85% | 0.32% | mature\_B\_cell\_down | Gata3 |
| GO:0002444 | myeloid leukocyte mediated immunity | 8.09e-02 | 1.56e-01 | 1 | 49 | 26 | 15137 | 2.04% | 0.17% | 3.85% | 0.32% | mature\_B\_cell\_down | Lat |
| GO:0051926 | negative regulation of calcium ion transport | 8.09e-02 | 1.56e-01 | 1 | 49 | 26 | 15137 | 2.04% | 0.17% | 3.85% | 0.32% | mature\_B\_cell\_down | Tmem66 |
| GO:0046579 | positive regulation of Ras protein signal transduction | 8.09e-02 | 1.56e-01 | 1 | 49 | 26 | 15137 | 2.04% | 0.17% | 3.85% | 0.32% | mature\_B\_cell\_down | F2r |
| GO:0010770 | positive regulation of cell morphogenesis involved in differentiation | 8.09e-02 | 1.56e-01 | 1 | 49 | 26 | 15137 | 2.04% | 0.17% | 3.85% | 0.32% | mature\_B\_cell\_down | Notch1 |
| GO:0044060 | regulation of endocrine process | 8.09e-02 | 1.56e-01 | 1 | 49 | 26 | 15137 | 2.04% | 0.17% | 3.85% | 0.32% | mature\_B\_cell\_down | F2r |
| GO:0033003 | regulation of mast cell activation | 8.09e-02 | 1.56e-01 | 1 | 49 | 26 | 15137 | 2.04% | 0.17% | 3.85% | 0.32% | mature\_B\_cell\_down | Cd226 |
| GO:0017145 | stem cell division | 8.09e-02 | 1.56e-01 | 1 | 49 | 26 | 15137 | 2.04% | 0.17% | 3.85% | 0.32% | mature\_B\_cell\_down | Notch1 |
| GO:0043408 | regulation of MAPK cascade | 8.11e-02 | 1.56e-01 | 3 | 49 | 314 | 15137 | 6.12% | 2.07% | 0.96% | 0.32% | mature\_B\_cell\_down | Cd27, F2r, Slc9a3r1 |
| GO:0006873 | cellular ion homeostasis | 8.28e-02 | 1.59e-01 | 4 | 49 | 511 | 15137 | 8.16% | 3.38% | 0.78% | 0.32% | mature\_B\_cell\_down | F2r, Fyn, Lck, Slc9a3r1 |
| GO:0050670 | regulation of lymphocyte proliferation | 8.29e-02 | 1.59e-01 | 2 | 49 | 148 | 15137 | 4.08% | 0.98% | 1.35% | 0.32% | mature\_B\_cell\_down | Prkcq, Zap70 |
| GO:0032318 | regulation of Ras GTPase activity | 8.39e-02 | 1.59e-01 | 2 | 49 | 149 | 15137 | 4.08% | 0.98% | 1.34% | 0.32% | mature\_B\_cell\_down | Tbc1d2b, Thy1 |
| GO:0032944 | regulation of mononuclear cell proliferation | 8.39e-02 | 1.59e-01 | 2 | 49 | 149 | 15137 | 4.08% | 0.98% | 1.34% | 0.32% | mature\_B\_cell\_down | Prkcq, Zap70 |
| GO:0050853 | B cell receptor signaling pathway | 8.39e-02 | 1.59e-01 | 1 | 49 | 27 | 15137 | 2.04% | 0.18% | 3.70% | 0.32% | mature\_B\_cell\_down | Lck |
| GO:0016236 | macroautophagy | 8.39e-02 | 1.59e-01 | 1 | 49 | 27 | 15137 | 2.04% | 0.18% | 3.70% | 0.32% | mature\_B\_cell\_down | Ctsd |
| GO:0030513 | positive regulation of BMP signaling pathway | 8.39e-02 | 1.59e-01 | 1 | 49 | 27 | 15137 | 2.04% | 0.18% | 3.70% | 0.32% | mature\_B\_cell\_down | Notch1 |
| GO:0042993 | positive regulation of transcription factor import into nucleus | 8.39e-02 | 1.59e-01 | 1 | 49 | 27 | 15137 | 2.04% | 0.18% | 3.70% | 0.32% | mature\_B\_cell\_down | Prkcq |
| GO:0045907 | positive regulation of vasoconstriction | 8.39e-02 | 1.59e-01 | 1 | 49 | 27 | 15137 | 2.04% | 0.18% | 3.70% | 0.32% | mature\_B\_cell\_down | F2r |
| GO:0060740 | prostate gland epithelium morphogenesis | 8.39e-02 | 1.59e-01 | 1 | 49 | 27 | 15137 | 2.04% | 0.18% | 3.70% | 0.32% | mature\_B\_cell\_down | Notch1 |
| GO:0043370 | regulation of CD4-positive, alpha-beta T cell differentiation | 8.39e-02 | 1.59e-01 | 1 | 49 | 27 | 15137 | 2.04% | 0.18% | 3.70% | 0.32% | mature\_B\_cell\_down | Gata3 |
| GO:0017158 | regulation of calcium ion-dependent exocytosis | 8.39e-02 | 1.59e-01 | 1 | 49 | 27 | 15137 | 2.04% | 0.18% | 3.70% | 0.32% | mature\_B\_cell\_down | Notch1 |
| GO:0060284 | regulation of cell development | 8.46e-02 | 1.60e-01 | 4 | 49 | 515 | 15137 | 8.16% | 3.40% | 0.78% | 0.32% | mature\_B\_cell\_down | Notch1, Prkch, Thy1, Tnik |
| GO:0051091 | positive regulation of sequence-specific DNA binding transcription factor activity | 8.58e-02 | 1.61e-01 | 2 | 49 | 151 | 15137 | 4.08% | 1.00% | 1.32% | 0.32% | mature\_B\_cell\_down | Prkch, Prkcq |
| GO:0042491 | auditory receptor cell differentiation | 8.69e-02 | 1.61e-01 | 1 | 49 | 28 | 15137 | 2.04% | 0.18% | 3.57% | 0.32% | mature\_B\_cell\_down | Notch1 |
| GO:0007044 | cell-substrate junction assembly | 8.69e-02 | 1.61e-01 | 1 | 49 | 28 | 15137 | 2.04% | 0.18% | 3.57% | 0.32% | mature\_B\_cell\_down | Thy1 |
| GO:0010812 | negative regulation of cell-substrate adhesion | 8.69e-02 | 1.61e-01 | 1 | 49 | 28 | 15137 | 2.04% | 0.18% | 3.57% | 0.32% | mature\_B\_cell\_down | Notch1 |
| GO:0003407 | neural retina development | 8.69e-02 | 1.61e-01 | 1 | 49 | 28 | 15137 | 2.04% | 0.18% | 3.57% | 0.32% | mature\_B\_cell\_down | Thy1 |
| GO:0032233 | positive regulation of actin filament bundle assembly | 8.69e-02 | 1.61e-01 | 1 | 49 | 28 | 15137 | 2.04% | 0.18% | 3.57% | 0.32% | mature\_B\_cell\_down | Prkcq |
| GO:0060512 | prostate gland morphogenesis | 8.69e-02 | 1.61e-01 | 1 | 49 | 28 | 15137 | 2.04% | 0.18% | 3.57% | 0.32% | mature\_B\_cell\_down | Notch1 |
| GO:0007205 | protein kinase C-activating G-protein coupled receptor signaling pathway | 8.69e-02 | 1.61e-01 | 1 | 49 | 28 | 15137 | 2.04% | 0.18% | 3.57% | 0.32% | mature\_B\_cell\_down | F2r |
| GO:0042345 | regulation of NF-kappaB import into nucleus | 8.69e-02 | 1.61e-01 | 1 | 49 | 28 | 15137 | 2.04% | 0.18% | 3.57% | 0.32% | mature\_B\_cell\_down | Prkcq |
| GO:2001236 | regulation of extrinsic apoptotic signaling pathway | 8.69e-02 | 1.61e-01 | 1 | 49 | 28 | 15137 | 2.04% | 0.18% | 3.57% | 0.32% | mature\_B\_cell\_down | Fyn |
| GO:0032350 | regulation of hormone metabolic process | 8.69e-02 | 1.61e-01 | 1 | 49 | 28 | 15137 | 2.04% | 0.18% | 3.57% | 0.32% | mature\_B\_cell\_down | Gata3 |
| GO:0032651 | regulation of interleukin-1 beta production | 8.69e-02 | 1.61e-01 | 1 | 49 | 28 | 15137 | 2.04% | 0.18% | 3.57% | 0.32% | mature\_B\_cell\_down | F2r |
| GO:0044246 | regulation of multicellular organismal metabolic process | 8.69e-02 | 1.61e-01 | 1 | 49 | 28 | 15137 | 2.04% | 0.18% | 3.57% | 0.32% | mature\_B\_cell\_down | F2r |
| GO:0042269 | regulation of natural killer cell mediated cytotoxicity | 8.69e-02 | 1.61e-01 | 1 | 49 | 28 | 15137 | 2.04% | 0.18% | 3.57% | 0.32% | mature\_B\_cell\_down | Cd226 |
| GO:0002715 | regulation of natural killer cell mediated immunity | 8.69e-02 | 1.61e-01 | 1 | 49 | 28 | 15137 | 2.04% | 0.18% | 3.57% | 0.32% | mature\_B\_cell\_down | Cd226 |
| GO:0048713 | regulation of oligodendrocyte differentiation | 8.69e-02 | 1.61e-01 | 1 | 49 | 28 | 15137 | 2.04% | 0.18% | 3.57% | 0.32% | mature\_B\_cell\_down | Notch1 |
| GO:0032479 | regulation of type I interferon production | 8.69e-02 | 1.61e-01 | 1 | 49 | 28 | 15137 | 2.04% | 0.18% | 3.57% | 0.32% | mature\_B\_cell\_down | Ifih1 |
| GO:0070663 | regulation of leukocyte proliferation | 8.77e-02 | 1.62e-01 | 2 | 49 | 153 | 15137 | 4.08% | 1.01% | 1.31% | 0.32% | mature\_B\_cell\_down | Prkcq, Zap70 |
| GO:0051480 | cytosolic calcium ion homeostasis | 8.87e-02 | 1.64e-01 | 2 | 49 | 154 | 15137 | 4.08% | 1.02% | 1.30% | 0.32% | mature\_B\_cell\_down | F2r, Lck |
| GO:0052547 | regulation of peptidase activity | 8.97e-02 | 1.64e-01 | 3 | 49 | 328 | 15137 | 6.12% | 2.17% | 0.91% | 0.32% | mature\_B\_cell\_down | Cd27, F2r, Lck |
| GO:0071456 | cellular response to hypoxia | 8.98e-02 | 1.64e-01 | 1 | 49 | 29 | 15137 | 2.04% | 0.19% | 3.45% | 0.32% | mature\_B\_cell\_down | Notch1 |
| GO:0000578 | embryonic axis specification | 8.98e-02 | 1.64e-01 | 1 | 49 | 29 | 15137 | 2.04% | 0.19% | 3.45% | 0.32% | mature\_B\_cell\_down | Nckap1 |
| GO:0042462 | eye photoreceptor cell development | 8.98e-02 | 1.64e-01 | 1 | 49 | 29 | 15137 | 2.04% | 0.19% | 3.45% | 0.32% | mature\_B\_cell\_down | Thy1 |
| GO:0031069 | hair follicle morphogenesis | 8.98e-02 | 1.64e-01 | 1 | 49 | 29 | 15137 | 2.04% | 0.19% | 3.45% | 0.32% | mature\_B\_cell\_down | Notch1 |
| GO:0045599 | negative regulation of fat cell differentiation | 8.98e-02 | 1.64e-01 | 1 | 49 | 29 | 15137 | 2.04% | 0.19% | 3.45% | 0.32% | mature\_B\_cell\_down | Gata3 |
| GO:2000107 | negative regulation of leukocyte apoptotic process | 8.98e-02 | 1.64e-01 | 1 | 49 | 29 | 15137 | 2.04% | 0.19% | 3.45% | 0.32% | mature\_B\_cell\_down | Prkcq |
| GO:0045687 | positive regulation of glial cell differentiation | 8.98e-02 | 1.64e-01 | 1 | 49 | 29 | 15137 | 2.04% | 0.19% | 3.45% | 0.32% | mature\_B\_cell\_down | Notch1 |
| GO:2001014 | regulation of skeletal muscle cell differentiation | 8.98e-02 | 1.64e-01 | 1 | 49 | 29 | 15137 | 2.04% | 0.19% | 3.45% | 0.32% | mature\_B\_cell\_down | Notch1 |
| GO:0016444 | somatic cell DNA recombination | 8.98e-02 | 1.64e-01 | 1 | 49 | 29 | 15137 | 2.04% | 0.19% | 3.45% | 0.32% | mature\_B\_cell\_down | Tcf7 |
| GO:0002562 | somatic diversification of immune receptors via germline recombination within a single locus | 8.98e-02 | 1.64e-01 | 1 | 49 | 29 | 15137 | 2.04% | 0.19% | 3.45% | 0.32% | mature\_B\_cell\_down | Tcf7 |
| GO:0008219 | cell death | 9.07e-02 | 1.65e-01 | 5 | 49 | 742 | 15137 | 10.20% | 4.90% | 0.67% | 0.32% | mature\_B\_cell\_down | Cd27, F2r, Lck, Slc9a3r1, Tnfrsf1a |
| GO:0000122 | negative regulation of transcription from RNA polymerase II promoter | 9.12e-02 | 1.66e-01 | 4 | 49 | 529 | 15137 | 8.16% | 3.49% | 0.76% | 0.32% | mature\_B\_cell\_down | Gata3, Lpin1, Notch1, Sla2 |
| GO:0009617 | response to bacterium | 9.16e-02 | 1.66e-01 | 3 | 49 | 331 | 15137 | 6.12% | 2.19% | 0.91% | 0.32% | mature\_B\_cell\_down | F2r, Notch1, Tnfrsf1a |
| GO:0046165 | alcohol biosynthetic process | 9.28e-02 | 1.68e-01 | 1 | 49 | 30 | 15137 | 2.04% | 0.20% | 3.33% | 0.32% | mature\_B\_cell\_down | Gata3 |
| GO:2001257 | regulation of cation channel activity | 9.28e-02 | 1.68e-01 | 1 | 49 | 30 | 15137 | 2.04% | 0.20% | 3.33% | 0.32% | mature\_B\_cell\_down | Tmem66 |
| GO:0051931 | regulation of sensory perception | 9.28e-02 | 1.68e-01 | 1 | 49 | 30 | 15137 | 2.04% | 0.20% | 3.33% | 0.32% | mature\_B\_cell\_down | F2r |
| GO:0051930 | regulation of sensory perception of pain | 9.28e-02 | 1.68e-01 | 1 | 49 | 30 | 15137 | 2.04% | 0.20% | 3.33% | 0.32% | mature\_B\_cell\_down | F2r |
| GO:0048839 | inner ear development | 9.37e-02 | 1.69e-01 | 2 | 49 | 159 | 15137 | 4.08% | 1.05% | 1.26% | 0.32% | mature\_B\_cell\_down | Gata3, Notch1 |
| GO:0055082 | cellular chemical homeostasis | 9.37e-02 | 1.69e-01 | 4 | 49 | 534 | 15137 | 8.16% | 3.53% | 0.75% | 0.32% | mature\_B\_cell\_down | F2r, Fyn, Lck, Slc9a3r1 |
| GO:0016265 | death | 9.43e-02 | 1.70e-01 | 5 | 49 | 751 | 15137 | 10.20% | 4.96% | 0.67% | 0.32% | mature\_B\_cell\_down | Cd27, F2r, Lck, Slc9a3r1, Tnfrsf1a |
| GO:0048870 | cell motility | 9.46e-02 | 1.70e-01 | 4 | 49 | 536 | 15137 | 8.16% | 3.54% | 0.75% | 0.32% | mature\_B\_cell\_down | Fyn, Gata3, Nckap1, Selplg |
| GO:0051674 | localization of cell | 9.46e-02 | 1.70e-01 | 4 | 49 | 536 | 15137 | 8.16% | 3.54% | 0.75% | 0.32% | mature\_B\_cell\_down | Fyn, Gata3, Nckap1, Selplg |
| GO:0002009 | morphogenesis of an epithelium | 9.54e-02 | 1.71e-01 | 3 | 49 | 337 | 15137 | 6.12% | 2.23% | 0.89% | 0.32% | mature\_B\_cell\_down | Gata3, Nckap1, Notch1 |
| GO:0050730 | regulation of peptidyl-tyrosine phosphorylation | 9.56e-02 | 1.71e-01 | 2 | 49 | 161 | 15137 | 4.08% | 1.06% | 1.24% | 0.32% | mature\_B\_cell\_down | Lck, Tnfrsf1a |
| GO:0048813 | dendrite morphogenesis | 9.57e-02 | 1.71e-01 | 1 | 49 | 31 | 15137 | 2.04% | 0.20% | 3.23% | 0.32% | mature\_B\_cell\_down | Fyn |
| GO:0002275 | myeloid cell activation involved in immune response | 9.57e-02 | 1.71e-01 | 1 | 49 | 31 | 15137 | 2.04% | 0.20% | 3.23% | 0.32% | mature\_B\_cell\_down | Lat |
| GO:0007528 | neuromuscular junction development | 9.57e-02 | 1.71e-01 | 1 | 49 | 31 | 15137 | 2.04% | 0.20% | 3.23% | 0.32% | mature\_B\_cell\_down | F2r |
| GO:0051057 | positive regulation of small GTPase mediated signal transduction | 9.57e-02 | 1.71e-01 | 1 | 49 | 31 | 15137 | 2.04% | 0.20% | 3.23% | 0.32% | mature\_B\_cell\_down | F2r |
| GO:0048568 | embryonic organ development | 9.60e-02 | 1.71e-01 | 3 | 49 | 338 | 15137 | 6.12% | 2.23% | 0.89% | 0.32% | mature\_B\_cell\_down | Gata3, Nckap1, Tcf7 |
| GO:0045893 | positive regulation of transcription, DNA-dependent | 9.71e-02 | 1.73e-01 | 6 | 49 | 986 | 15137 | 12.24% | 6.51% | 0.61% | 0.32% | mature\_B\_cell\_down | F2r, Gata3, Lpin1, Notch1, Skap1, Tnfrsf1a |
| GO:0032147 | activation of protein kinase activity | 9.77e-02 | 1.73e-01 | 2 | 49 | 163 | 15137 | 4.08% | 1.08% | 1.23% | 0.32% | mature\_B\_cell\_down | F2r, Tnik |
| GO:0048731 | system development | 9.84e-02 | 1.73e-01 | 13 | 49 | 2767 | 15137 | 26.53% | 18.28% | 0.47% | 0.32% | mature\_B\_cell\_down | F2r, Fyn, Gata3, Itk, Lck, Nckap1, Notch1, Prkch, Tcf7, Thy1, Tnik, Txk, Zap70 |
| GO:0007259 | JAK-STAT cascade | 9.87e-02 | 1.73e-01 | 1 | 49 | 32 | 15137 | 2.04% | 0.21% | 3.12% | 0.32% | mature\_B\_cell\_down | F2r |
| GO:0071453 | cellular response to oxygen levels | 9.87e-02 | 1.73e-01 | 1 | 49 | 32 | 15137 | 2.04% | 0.21% | 3.12% | 0.32% | mature\_B\_cell\_down | Notch1 |
| GO:0048566 | embryonic digestive tract development | 9.87e-02 | 1.73e-01 | 1 | 49 | 32 | 15137 | 2.04% | 0.21% | 3.12% | 0.32% | mature\_B\_cell\_down | Tcf7 |
| GO:0035116 | embryonic hindlimb morphogenesis | 9.87e-02 | 1.73e-01 | 1 | 49 | 32 | 15137 | 2.04% | 0.21% | 3.12% | 0.32% | mature\_B\_cell\_down | Notch1 |
| GO:0007159 | leukocyte cell-cell adhesion | 9.87e-02 | 1.73e-01 | 1 | 49 | 32 | 15137 | 2.04% | 0.21% | 3.12% | 0.32% | mature\_B\_cell\_down | Selplg |
| GO:0050771 | negative regulation of axonogenesis | 9.87e-02 | 1.73e-01 | 1 | 49 | 32 | 15137 | 2.04% | 0.21% | 3.12% | 0.32% | mature\_B\_cell\_down | Thy1 |
| GO:0014014 | negative regulation of gliogenesis | 9.87e-02 | 1.73e-01 | 1 | 49 | 32 | 15137 | 2.04% | 0.21% | 3.12% | 0.32% | mature\_B\_cell\_down | Notch1 |
| GO:0031058 | positive regulation of histone modification | 9.87e-02 | 1.73e-01 | 1 | 49 | 32 | 15137 | 2.04% | 0.21% | 3.12% | 0.32% | mature\_B\_cell\_down | Lpin1 |
| GO:0032652 | regulation of interleukin-1 production | 9.87e-02 | 1.73e-01 | 1 | 49 | 32 | 15137 | 2.04% | 0.21% | 3.12% | 0.32% | mature\_B\_cell\_down | F2r |
| GO:0003156 | regulation of organ formation | 9.87e-02 | 1.73e-01 | 1 | 49 | 32 | 15137 | 2.04% | 0.21% | 3.12% | 0.32% | mature\_B\_cell\_down | Notch1 |
| GO:0002200 | somatic diversification of immune receptors | 9.87e-02 | 1.73e-01 | 1 | 49 | 32 | 15137 | 2.04% | 0.21% | 3.12% | 0.32% | mature\_B\_cell\_down | Tcf7 |
| GO:0002040 | sprouting angiogenesis | 9.87e-02 | 1.73e-01 | 1 | 49 | 32 | 15137 | 2.04% | 0.21% | 3.12% | 0.32% | mature\_B\_cell\_down | Notch1 |
| GO:0043086 | negative regulation of catalytic activity | 1.00e-01 | 1.75e-01 | 4 | 49 | 547 | 15137 | 8.16% | 3.61% | 0.73% | 0.32% | mature\_B\_cell\_down | Cd27, Notch1, Sytl2, Thy1 |
| GO:0050727 | regulation of inflammatory response | 1.01e-01 | 1.75e-01 | 2 | 49 | 166 | 15137 | 4.08% | 1.10% | 1.20% | 0.32% | mature\_B\_cell\_down | Gata3, Tnfrsf1a |
| GO:0032386 | regulation of intracellular transport | 1.01e-01 | 1.75e-01 | 2 | 49 | 166 | 15137 | 4.08% | 1.10% | 1.20% | 0.32% | mature\_B\_cell\_down | Prkcq, Tnfrsf1a |
| GO:0001701 | in utero embryonic development | 1.01e-01 | 1.75e-01 | 3 | 49 | 346 | 15137 | 6.12% | 2.29% | 0.87% | 0.32% | mature\_B\_cell\_down | Gata3, Nckap1, Notch1 |
| GO:0001709 | cell fate determination | 1.02e-01 | 1.75e-01 | 1 | 49 | 33 | 15137 | 2.04% | 0.22% | 3.03% | 0.32% | mature\_B\_cell\_down | Gata3 |
| GO:0030514 | negative regulation of BMP signaling pathway | 1.02e-01 | 1.75e-01 | 1 | 49 | 33 | 15137 | 2.04% | 0.22% | 3.03% | 0.32% | mature\_B\_cell\_down | Notch1 |
| GO:0042177 | negative regulation of protein catabolic process | 1.02e-01 | 1.75e-01 | 1 | 49 | 33 | 15137 | 2.04% | 0.22% | 3.03% | 0.32% | mature\_B\_cell\_down | Fyn |
| GO:0030168 | platelet activation | 1.02e-01 | 1.75e-01 | 1 | 49 | 33 | 15137 | 2.04% | 0.22% | 3.03% | 0.32% | mature\_B\_cell\_down | F2r |
| GO:0043388 | positive regulation of DNA binding | 1.02e-01 | 1.75e-01 | 1 | 49 | 33 | 15137 | 2.04% | 0.22% | 3.03% | 0.32% | mature\_B\_cell\_down | Gata3 |
| GO:0046638 | positive regulation of alpha-beta T cell differentiation | 1.02e-01 | 1.75e-01 | 1 | 49 | 33 | 15137 | 2.04% | 0.22% | 3.03% | 0.32% | mature\_B\_cell\_down | Zap70 |
| GO:2000179 | positive regulation of neural precursor cell proliferation | 1.02e-01 | 1.75e-01 | 1 | 49 | 33 | 15137 | 2.04% | 0.22% | 3.03% | 0.32% | mature\_B\_cell\_down | Notch1 |
| GO:0032760 | positive regulation of tumor necrosis factor production | 1.02e-01 | 1.75e-01 | 1 | 49 | 33 | 15137 | 2.04% | 0.22% | 3.03% | 0.32% | mature\_B\_cell\_down | Tnfrsf1a |
| GO:0010717 | regulation of epithelial to mesenchymal transition | 1.02e-01 | 1.75e-01 | 1 | 49 | 33 | 15137 | 2.04% | 0.22% | 3.03% | 0.32% | mature\_B\_cell\_down | Notch1 |
| GO:0046626 | regulation of insulin receptor signaling pathway | 1.02e-01 | 1.75e-01 | 1 | 49 | 33 | 15137 | 2.04% | 0.22% | 3.03% | 0.32% | mature\_B\_cell\_down | Prkcq |
| GO:0042098 | T cell proliferation | 1.04e-01 | 1.79e-01 | 1 | 49 | 34 | 15137 | 2.04% | 0.22% | 2.94% | 0.32% | mature\_B\_cell\_down | Fyn |
| GO:0009712 | catechol-containing compound metabolic process | 1.04e-01 | 1.79e-01 | 1 | 49 | 34 | 15137 | 2.04% | 0.22% | 2.94% | 0.32% | mature\_B\_cell\_down | Gata3 |
| GO:0006584 | catecholamine metabolic process | 1.04e-01 | 1.79e-01 | 1 | 49 | 34 | 15137 | 2.04% | 0.22% | 2.94% | 0.32% | mature\_B\_cell\_down | Gata3 |
| GO:0034311 | diol metabolic process | 1.04e-01 | 1.79e-01 | 1 | 49 | 34 | 15137 | 2.04% | 0.22% | 2.94% | 0.32% | mature\_B\_cell\_down | Gata3 |
| GO:0070228 | regulation of lymphocyte apoptotic process | 1.04e-01 | 1.79e-01 | 1 | 49 | 34 | 15137 | 2.04% | 0.22% | 2.94% | 0.32% | mature\_B\_cell\_down | Prkcq |
| GO:0060042 | retina morphogenesis in camera-type eye | 1.04e-01 | 1.79e-01 | 1 | 49 | 34 | 15137 | 2.04% | 0.22% | 2.94% | 0.32% | mature\_B\_cell\_down | Thy1 |
| GO:0048538 | thymus development | 1.04e-01 | 1.79e-01 | 1 | 49 | 34 | 15137 | 2.04% | 0.22% | 2.94% | 0.32% | mature\_B\_cell\_down | Gata3 |
| GO:0007507 | heart development | 1.05e-01 | 1.80e-01 | 3 | 49 | 352 | 15137 | 6.12% | 2.33% | 0.85% | 0.32% | mature\_B\_cell\_down | Gata3, Nckap1, Notch1 |
| GO:0006811 | ion transport | 1.06e-01 | 1.80e-01 | 5 | 49 | 778 | 15137 | 10.20% | 5.14% | 0.64% | 0.32% | mature\_B\_cell\_down | F2r, Lck, Slc12a7, Slc9a3r1, Tmem66 |
| GO:0045664 | regulation of neuron differentiation | 1.06e-01 | 1.80e-01 | 3 | 49 | 353 | 15137 | 6.12% | 2.33% | 0.85% | 0.32% | mature\_B\_cell\_down | Notch1, Thy1, Tnik |
| GO:0051252 | regulation of RNA metabolic process | 1.06e-01 | 1.80e-01 | 12 | 49 | 2532 | 15137 | 24.49% | 16.73% | 0.47% | 0.32% | mature\_B\_cell\_down | F2r, Gata3, Hnrpll, Lpin1, Notch1, Prkch, Prkcq, Skap1, Sla2, Tcf7, Tnfrsf1a, Txk |
| GO:0010720 | positive regulation of cell development | 1.07e-01 | 1.82e-01 | 2 | 49 | 172 | 15137 | 4.08% | 1.14% | 1.16% | 0.32% | mature\_B\_cell\_down | Notch1, Prkch |
| GO:0043009 | chordate embryonic development | 1.07e-01 | 1.82e-01 | 4 | 49 | 561 | 15137 | 8.16% | 3.71% | 0.71% | 0.32% | mature\_B\_cell\_down | Gata3, Nckap1, Notch1, Tcf7 |
| GO:0042401 | cellular biogenic amine biosynthetic process | 1.07e-01 | 1.82e-01 | 1 | 49 | 35 | 15137 | 2.04% | 0.23% | 2.86% | 0.32% | mature\_B\_cell\_down | Gata3 |
| GO:0048806 | genitalia development | 1.07e-01 | 1.82e-01 | 1 | 49 | 35 | 15137 | 2.04% | 0.23% | 2.86% | 0.32% | mature\_B\_cell\_down | Tcf7 |
| GO:0050709 | negative regulation of protein secretion | 1.07e-01 | 1.82e-01 | 1 | 49 | 35 | 15137 | 2.04% | 0.23% | 2.86% | 0.32% | mature\_B\_cell\_down | F2r |
| GO:0032729 | positive regulation of interferon-gamma production | 1.07e-01 | 1.82e-01 | 1 | 49 | 35 | 15137 | 2.04% | 0.23% | 2.86% | 0.32% | mature\_B\_cell\_down | Txk |
| GO:0048589 | developmental growth | 1.08e-01 | 1.82e-01 | 2 | 49 | 173 | 15137 | 4.08% | 1.14% | 1.16% | 0.32% | mature\_B\_cell\_down | Gata3, Notch1 |
| GO:0003007 | heart morphogenesis | 1.09e-01 | 1.84e-01 | 2 | 49 | 174 | 15137 | 4.08% | 1.15% | 1.15% | 0.32% | mature\_B\_cell\_down | Gata3, Notch1 |
| GO:0071356 | cellular response to tumor necrosis factor | 1.10e-01 | 1.85e-01 | 1 | 49 | 36 | 15137 | 2.04% | 0.24% | 2.78% | 0.32% | mature\_B\_cell\_down | Tnfrsf1a |
| GO:0048015 | phosphatidylinositol-mediated signaling | 1.10e-01 | 1.85e-01 | 1 | 49 | 36 | 15137 | 2.04% | 0.24% | 2.78% | 0.32% | mature\_B\_cell\_down | Gata3 |
| GO:0010595 | positive regulation of endothelial cell migration | 1.10e-01 | 1.85e-01 | 1 | 49 | 36 | 15137 | 2.04% | 0.24% | 2.78% | 0.32% | mature\_B\_cell\_down | Gata3 |
| GO:0032313 | regulation of Rab GTPase activity | 1.10e-01 | 1.85e-01 | 1 | 49 | 36 | 15137 | 2.04% | 0.24% | 2.78% | 0.32% | mature\_B\_cell\_down | Tbc1d2b |
| GO:0032483 | regulation of Rab protein signal transduction | 1.10e-01 | 1.85e-01 | 1 | 49 | 36 | 15137 | 2.04% | 0.24% | 2.78% | 0.32% | mature\_B\_cell\_down | Tbc1d2b |
| GO:1900076 | regulation of cellular response to insulin stimulus | 1.10e-01 | 1.85e-01 | 1 | 49 | 36 | 15137 | 2.04% | 0.24% | 2.78% | 0.32% | mature\_B\_cell\_down | Prkcq |
| GO:0032677 | regulation of interleukin-8 production | 1.10e-01 | 1.85e-01 | 1 | 49 | 36 | 15137 | 2.04% | 0.24% | 2.78% | 0.32% | mature\_B\_cell\_down | F2r |
| GO:0009792 | embryo development ending in birth or egg hatching | 1.10e-01 | 1.85e-01 | 4 | 49 | 567 | 15137 | 8.16% | 3.75% | 0.71% | 0.32% | mature\_B\_cell\_down | Gata3, Nckap1, Notch1, Tcf7 |
| GO:0050801 | ion homeostasis | 1.11e-01 | 1.85e-01 | 4 | 49 | 568 | 15137 | 8.16% | 3.75% | 0.70% | 0.32% | mature\_B\_cell\_down | F2r, Fyn, Lck, Slc9a3r1 |
| GO:0001754 | eye photoreceptor cell differentiation | 1.13e-01 | 1.88e-01 | 1 | 49 | 37 | 15137 | 2.04% | 0.24% | 2.70% | 0.32% | mature\_B\_cell\_down | Thy1 |
| GO:0048017 | inositol lipid-mediated signaling | 1.13e-01 | 1.88e-01 | 1 | 49 | 37 | 15137 | 2.04% | 0.24% | 2.70% | 0.32% | mature\_B\_cell\_down | Gata3 |
| GO:0042461 | photoreceptor cell development | 1.13e-01 | 1.88e-01 | 1 | 49 | 37 | 15137 | 2.04% | 0.24% | 2.70% | 0.32% | mature\_B\_cell\_down | Thy1 |
| GO:0001912 | positive regulation of leukocyte mediated cytotoxicity | 1.13e-01 | 1.88e-01 | 1 | 49 | 37 | 15137 | 2.04% | 0.24% | 2.70% | 0.32% | mature\_B\_cell\_down | Cd226 |
| GO:0014068 | positive regulation of phosphatidylinositol 3-kinase cascade | 1.13e-01 | 1.88e-01 | 1 | 49 | 37 | 15137 | 2.04% | 0.24% | 2.70% | 0.32% | mature\_B\_cell\_down | F2r |
| GO:0001959 | regulation of cytokine-mediated signaling pathway | 1.13e-01 | 1.88e-01 | 1 | 49 | 37 | 15137 | 2.04% | 0.24% | 2.70% | 0.32% | mature\_B\_cell\_down | Txk |
| GO:0002028 | regulation of sodium ion transport | 1.16e-01 | 1.92e-01 | 1 | 49 | 38 | 15137 | 2.04% | 0.25% | 2.63% | 0.32% | mature\_B\_cell\_down | Slc9a3r1 |
| GO:0051492 | regulation of stress fiber assembly | 1.16e-01 | 1.92e-01 | 1 | 49 | 38 | 15137 | 2.04% | 0.25% | 2.63% | 0.32% | mature\_B\_cell\_down | Prkcq |
| GO:0043523 | regulation of neuron apoptotic process | 1.17e-01 | 1.94e-01 | 2 | 49 | 182 | 15137 | 4.08% | 1.20% | 1.10% | 0.32% | mature\_B\_cell\_down | F2r, Gata3 |
| GO:0003006 | developmental process involved in reproduction | 1.18e-01 | 1.94e-01 | 3 | 49 | 370 | 15137 | 6.12% | 2.44% | 0.81% | 0.32% | mature\_B\_cell\_down | Gata3, Notch1, Tcf7 |
| GO:0043410 | positive regulation of MAPK cascade | 1.18e-01 | 1.95e-01 | 2 | 49 | 183 | 15137 | 4.08% | 1.21% | 1.09% | 0.32% | mature\_B\_cell\_down | Cd27, F2r |
| GO:0009948 | anterior/posterior axis specification | 1.19e-01 | 1.96e-01 | 1 | 49 | 39 | 15137 | 2.04% | 0.26% | 2.56% | 0.32% | mature\_B\_cell\_down | Nckap1 |
| GO:0010605 | negative regulation of macromolecule metabolic process | 1.20e-01 | 1.98e-01 | 7 | 49 | 1290 | 15137 | 14.29% | 8.52% | 0.54% | 0.32% | mature\_B\_cell\_down | Fyn, Gata3, Lpin1, Notch1, Sla2, Thy1, Tnfrsf1a |
| GO:0000904 | cell morphogenesis involved in differentiation | 1.20e-01 | 1.98e-01 | 3 | 49 | 374 | 15137 | 6.12% | 2.47% | 0.80% | 0.32% | mature\_B\_cell\_down | Fyn, Gata3, Notch1 |
| GO:0022607 | cellular component assembly | 1.21e-01 | 1.99e-01 | 6 | 49 | 1049 | 15137 | 12.24% | 6.93% | 0.57% | 0.32% | mature\_B\_cell\_down | Ctsd, Nckap1, Sept9, Slc9a3r1, Thy1, Tnfrsf1a |
| GO:0043583 | ear development | 1.22e-01 | 1.99e-01 | 2 | 49 | 186 | 15137 | 4.08% | 1.23% | 1.08% | 0.32% | mature\_B\_cell\_down | Gata3, Notch1 |
| GO:0050886 | endocrine process | 1.22e-01 | 1.99e-01 | 1 | 49 | 40 | 15137 | 2.04% | 0.26% | 2.50% | 0.32% | mature\_B\_cell\_down | Gata3 |
| GO:0051148 | negative regulation of muscle cell differentiation | 1.22e-01 | 1.99e-01 | 1 | 49 | 40 | 15137 | 2.04% | 0.26% | 2.50% | 0.32% | mature\_B\_cell\_down | Notch1 |
| GO:2001252 | positive regulation of chromosome organization | 1.22e-01 | 1.99e-01 | 1 | 49 | 40 | 15137 | 2.04% | 0.26% | 2.50% | 0.32% | mature\_B\_cell\_down | Lpin1 |
| GO:0007034 | vacuolar transport | 1.22e-01 | 1.99e-01 | 1 | 49 | 40 | 15137 | 2.04% | 0.26% | 2.50% | 0.32% | mature\_B\_cell\_down | Vps26b |
| GO:0043087 | regulation of GTPase activity | 1.23e-01 | 2.00e-01 | 2 | 49 | 187 | 15137 | 4.08% | 1.24% | 1.07% | 0.32% | mature\_B\_cell\_down | Tbc1d2b, Thy1 |
| GO:0031175 | neuron projection development | 1.24e-01 | 2.02e-01 | 3 | 49 | 379 | 15137 | 6.12% | 2.50% | 0.79% | 0.32% | mature\_B\_cell\_down | Fyn, Gata3, Notch1 |
| GO:0048483 | autonomic nervous system development | 1.25e-01 | 2.02e-01 | 1 | 49 | 41 | 15137 | 2.04% | 0.27% | 2.44% | 0.32% | mature\_B\_cell\_down | Gata3 |
| GO:0002377 | immunoglobulin production | 1.25e-01 | 2.02e-01 | 1 | 49 | 41 | 15137 | 2.04% | 0.27% | 2.44% | 0.32% | mature\_B\_cell\_down | Cd27 |
| GO:0030850 | prostate gland development | 1.25e-01 | 2.02e-01 | 1 | 49 | 41 | 15137 | 2.04% | 0.27% | 2.44% | 0.32% | mature\_B\_cell\_down | Notch1 |
| GO:0002712 | regulation of B cell mediated immunity | 1.25e-01 | 2.02e-01 | 1 | 49 | 41 | 15137 | 2.04% | 0.27% | 2.44% | 0.32% | mature\_B\_cell\_down | Cd226 |
| GO:0002889 | regulation of immunoglobulin mediated immune response | 1.25e-01 | 2.02e-01 | 1 | 49 | 41 | 15137 | 2.04% | 0.27% | 2.44% | 0.32% | mature\_B\_cell\_down | Cd226 |
| GO:0048742 | regulation of skeletal muscle fiber development | 1.25e-01 | 2.02e-01 | 1 | 49 | 41 | 15137 | 2.04% | 0.27% | 2.44% | 0.32% | mature\_B\_cell\_down | Notch1 |
| GO:0050896 | response to stimulus | 1.26e-01 | 2.04e-01 | 25 | 49 | 6348 | 15137 | 51.02% | 41.94% | 0.39% | 0.32% | mature\_B\_cell\_down | Apol7b, Cd247, Cd3g, Ctsd, F2r, Fyn, Gata3, Ifi47, Ifih1, Itk, Lat, Lck, Lpin1, Notch1, Prkch, Prkcq, Rab37, Skap1, Slc9a3r1, Tcf7, Thy1, Tnfrsf1a, Tnik, Txk, Zap70 |
| GO:0010638 | positive regulation of organelle organization | 1.27e-01 | 2.05e-01 | 2 | 49 | 191 | 15137 | 4.08% | 1.26% | 1.05% | 0.32% | mature\_B\_cell\_down | Lpin1, Prkcq |
| GO:0031343 | positive regulation of cell killing | 1.27e-01 | 2.05e-01 | 1 | 49 | 42 | 15137 | 2.04% | 0.28% | 2.38% | 0.32% | mature\_B\_cell\_down | Cd226 |
| GO:0048814 | regulation of dendrite morphogenesis | 1.27e-01 | 2.05e-01 | 1 | 49 | 42 | 15137 | 2.04% | 0.28% | 2.38% | 0.32% | mature\_B\_cell\_down | Tnik |
| GO:0003281 | ventricular septum development | 1.27e-01 | 2.05e-01 | 1 | 49 | 42 | 15137 | 2.04% | 0.28% | 2.38% | 0.32% | mature\_B\_cell\_down | Gata3 |
| GO:0031329 | regulation of cellular catabolic process | 1.27e-01 | 2.05e-01 | 3 | 49 | 384 | 15137 | 6.12% | 2.54% | 0.78% | 0.32% | mature\_B\_cell\_down | Ipcef1, Tbc1d2b, Thy1 |
| GO:0048858 | cell projection morphogenesis | 1.29e-01 | 2.07e-01 | 3 | 49 | 386 | 15137 | 6.12% | 2.55% | 0.78% | 0.32% | mature\_B\_cell\_down | Fyn, Gata3, Notch1 |
| GO:0008283 | cell proliferation | 1.30e-01 | 2.08e-01 | 3 | 49 | 387 | 15137 | 6.12% | 2.56% | 0.78% | 0.32% | mature\_B\_cell\_down | Cd27, Fyn, Sh2d2a |
| GO:0007189 | adenylate cyclase-activating G-protein coupled receptor signaling pathway | 1.30e-01 | 2.08e-01 | 1 | 49 | 43 | 15137 | 2.04% | 0.28% | 2.33% | 0.32% | mature\_B\_cell\_down | Slc9a3r1 |
| GO:0010171 | body morphogenesis | 1.30e-01 | 2.08e-01 | 1 | 49 | 43 | 15137 | 2.04% | 0.28% | 2.33% | 0.32% | mature\_B\_cell\_down | Nckap1 |
| GO:0048730 | epidermis morphogenesis | 1.30e-01 | 2.08e-01 | 1 | 49 | 43 | 15137 | 2.04% | 0.28% | 2.33% | 0.32% | mature\_B\_cell\_down | Notch1 |
| GO:0009062 | fatty acid catabolic process | 1.30e-01 | 2.08e-01 | 1 | 49 | 43 | 15137 | 2.04% | 0.28% | 2.33% | 0.32% | mature\_B\_cell\_down | Lpin1 |
| GO:0060759 | regulation of response to cytokine stimulus | 1.30e-01 | 2.08e-01 | 1 | 49 | 43 | 15137 | 2.04% | 0.28% | 2.33% | 0.32% | mature\_B\_cell\_down | Txk |
| GO:0048489 | synaptic vesicle transport | 1.30e-01 | 2.08e-01 | 1 | 49 | 43 | 15137 | 2.04% | 0.28% | 2.33% | 0.32% | mature\_B\_cell\_down | Zdhhc15 |
| GO:0050794 | regulation of cellular process | 1.32e-01 | 2.11e-01 | 31 | 49 | 8223 | 15137 | 63.27% | 54.32% | 0.38% | 0.32% | mature\_B\_cell\_down | Apol7b, Cd226, Cd247, Cd27, Cd3g, F2r, Fyn, Gata3, Hnrpll, Ifih1, Ipcef1, Itk, Lat, Lck, Lpin1, Notch1, Prkch, Prkcq, Rab37, Skap1, Sla2, Slc9a3r1, Sytl2, Tbc1d2b, Tcf7, Thy1, Tmem66, Tnfrsf1a, Tnik, Txk, Zap70 |
| GO:0001837 | epithelial to mesenchymal transition | 1.33e-01 | 2.12e-01 | 1 | 49 | 44 | 15137 | 2.04% | 0.29% | 2.27% | 0.32% | mature\_B\_cell\_down | Notch1 |
| GO:0016043 | cellular component organization | 1.34e-01 | 2.13e-01 | 13 | 49 | 2914 | 15137 | 26.53% | 19.25% | 0.45% | 0.32% | mature\_B\_cell\_down | Ctsd, F2r, Fyn, Gata3, Lpin1, Nckap1, Notch1, Sept9, Slc9a3r1, Thy1, Tnfrsf1a, Tnik, Zdhhc15 |
| GO:0050678 | regulation of epithelial cell proliferation | 1.35e-01 | 2.14e-01 | 2 | 49 | 198 | 15137 | 4.08% | 1.31% | 1.01% | 0.32% | mature\_B\_cell\_down | Gata3, Notch1 |
| GO:0048608 | reproductive structure development | 1.35e-01 | 2.14e-01 | 2 | 49 | 198 | 15137 | 4.08% | 1.31% | 1.01% | 0.32% | mature\_B\_cell\_down | Gata3, Notch1 |
| GO:0007420 | brain development | 1.35e-01 | 2.14e-01 | 3 | 49 | 394 | 15137 | 6.12% | 2.60% | 0.76% | 0.32% | mature\_B\_cell\_down | Fyn, Notch1, Tcf7 |
| GO:0045165 | cell fate commitment | 1.36e-01 | 2.14e-01 | 2 | 49 | 199 | 15137 | 4.08% | 1.31% | 1.01% | 0.32% | mature\_B\_cell\_down | Gata3, Notch1 |
| GO:0007548 | sex differentiation | 1.36e-01 | 2.14e-01 | 2 | 49 | 199 | 15137 | 4.08% | 1.31% | 1.01% | 0.32% | mature\_B\_cell\_down | Gata3, Tcf7 |
| GO:0060113 | inner ear receptor cell differentiation | 1.36e-01 | 2.14e-01 | 1 | 49 | 45 | 15137 | 2.04% | 0.30% | 2.22% | 0.32% | mature\_B\_cell\_down | Notch1 |
| GO:0046530 | photoreceptor cell differentiation | 1.36e-01 | 2.14e-01 | 1 | 49 | 45 | 15137 | 2.04% | 0.30% | 2.22% | 0.32% | mature\_B\_cell\_down | Thy1 |
| GO:0032231 | regulation of actin filament bundle assembly | 1.36e-01 | 2.14e-01 | 1 | 49 | 45 | 15137 | 2.04% | 0.30% | 2.22% | 0.32% | mature\_B\_cell\_down | Prkcq |
| GO:0048741 | skeletal muscle fiber development | 1.36e-01 | 2.14e-01 | 1 | 49 | 45 | 15137 | 2.04% | 0.30% | 2.22% | 0.32% | mature\_B\_cell\_down | F2r |
| GO:0002237 | response to molecule of bacterial origin | 1.37e-01 | 2.15e-01 | 2 | 49 | 200 | 15137 | 4.08% | 1.32% | 1.00% | 0.32% | mature\_B\_cell\_down | F2r, Notch1 |
| GO:0030182 | neuron differentiation | 1.37e-01 | 2.16e-01 | 4 | 49 | 615 | 15137 | 8.16% | 4.06% | 0.65% | 0.32% | mature\_B\_cell\_down | Fyn, Gata3, Notch1, Thy1 |
| GO:0019722 | calcium-mediated signaling | 1.39e-01 | 2.17e-01 | 1 | 49 | 46 | 15137 | 2.04% | 0.30% | 2.17% | 0.32% | mature\_B\_cell\_down | Lat |
| GO:0035137 | hindlimb morphogenesis | 1.39e-01 | 2.17e-01 | 1 | 49 | 46 | 15137 | 2.04% | 0.30% | 2.17% | 0.32% | mature\_B\_cell\_down | Notch1 |
| GO:0045668 | negative regulation of osteoblast differentiation | 1.39e-01 | 2.17e-01 | 1 | 49 | 46 | 15137 | 2.04% | 0.30% | 2.17% | 0.32% | mature\_B\_cell\_down | Notch1 |
| GO:0002440 | production of molecular mediator of immune response | 1.39e-01 | 2.17e-01 | 1 | 49 | 46 | 15137 | 2.04% | 0.30% | 2.17% | 0.32% | mature\_B\_cell\_down | Cd27 |
| GO:2000106 | regulation of leukocyte apoptotic process | 1.39e-01 | 2.17e-01 | 1 | 49 | 46 | 15137 | 2.04% | 0.30% | 2.17% | 0.32% | mature\_B\_cell\_down | Prkcq |
| GO:0032990 | cell part morphogenesis | 1.39e-01 | 2.17e-01 | 3 | 49 | 400 | 15137 | 6.12% | 2.64% | 0.75% | 0.32% | mature\_B\_cell\_down | Fyn, Gata3, Notch1 |
| GO:0043170 | macromolecule metabolic process | 1.39e-01 | 2.17e-01 | 21 | 49 | 5219 | 15137 | 42.86% | 34.48% | 0.40% | 0.32% | mature\_B\_cell\_down | Art2b, Ctsd, Ctsw, F2r, Fyn, Gata3, Hnrpll, Ifih1, Itk, Lat, Lck, Lpin1, Notch1, Prkch, Prkcq, Stk39, Tcf7, Tnik, Txk, Zap70, Zdhhc15 |
| GO:0072358 | cardiovascular system development | 1.39e-01 | 2.17e-01 | 4 | 49 | 619 | 15137 | 8.16% | 4.09% | 0.65% | 0.32% | mature\_B\_cell\_down | Gata3, Nckap1, Notch1, Thy1 |
| GO:0072359 | circulatory system development | 1.39e-01 | 2.17e-01 | 4 | 49 | 619 | 15137 | 8.16% | 4.09% | 0.65% | 0.32% | mature\_B\_cell\_down | Gata3, Nckap1, Notch1, Thy1 |
| GO:0001655 | urogenital system development | 1.40e-01 | 2.18e-01 | 2 | 49 | 203 | 15137 | 4.08% | 1.34% | 0.99% | 0.32% | mature\_B\_cell\_down | Gata3, Notch1 |
| GO:0046330 | positive regulation of JNK cascade | 1.42e-01 | 2.20e-01 | 1 | 49 | 47 | 15137 | 2.04% | 0.31% | 2.13% | 0.32% | mature\_B\_cell\_down | Cd27 |
| GO:0001910 | regulation of leukocyte mediated cytotoxicity | 1.42e-01 | 2.20e-01 | 1 | 49 | 47 | 15137 | 2.04% | 0.31% | 2.13% | 0.32% | mature\_B\_cell\_down | Cd226 |
| GO:0019229 | regulation of vasoconstriction | 1.42e-01 | 2.20e-01 | 1 | 49 | 47 | 15137 | 2.04% | 0.31% | 2.13% | 0.32% | mature\_B\_cell\_down | F2r |
| GO:0009952 | anterior/posterior pattern specification | 1.43e-01 | 2.22e-01 | 2 | 49 | 206 | 15137 | 4.08% | 1.36% | 0.97% | 0.32% | mature\_B\_cell\_down | Nckap1, Notch1 |
| GO:0002285 | lymphocyte activation involved in immune response | 1.44e-01 | 2.23e-01 | 1 | 49 | 48 | 15137 | 2.04% | 0.32% | 2.08% | 0.32% | mature\_B\_cell\_down | Gata3 |
| GO:2001233 | regulation of apoptotic signaling pathway | 1.44e-01 | 2.23e-01 | 1 | 49 | 48 | 15137 | 2.04% | 0.32% | 2.08% | 0.32% | mature\_B\_cell\_down | Fyn |
| GO:0045471 | response to ethanol | 1.44e-01 | 2.23e-01 | 1 | 49 | 48 | 15137 | 2.04% | 0.32% | 2.08% | 0.32% | mature\_B\_cell\_down | Fyn |
| GO:0060675 | ureteric bud morphogenesis | 1.44e-01 | 2.23e-01 | 1 | 49 | 48 | 15137 | 2.04% | 0.32% | 2.08% | 0.32% | mature\_B\_cell\_down | Gata3 |
| GO:0010033 | response to organic substance | 1.46e-01 | 2.25e-01 | 7 | 49 | 1358 | 15137 | 14.29% | 8.97% | 0.52% | 0.32% | mature\_B\_cell\_down | F2r, Fyn, Gata3, Lpin1, Notch1, Tcf7, Tnfrsf1a |
| GO:0007155 | cell adhesion | 1.47e-01 | 2.26e-01 | 4 | 49 | 631 | 15137 | 8.16% | 4.17% | 0.63% | 0.32% | mature\_B\_cell\_down | Cd226, Cd96, Selplg, Thy1 |
| GO:0072329 | monocarboxylic acid catabolic process | 1.47e-01 | 2.26e-01 | 1 | 49 | 49 | 15137 | 2.04% | 0.32% | 2.04% | 0.32% | mature\_B\_cell\_down | Lpin1 |
| GO:0060491 | regulation of cell projection assembly | 1.47e-01 | 2.26e-01 | 1 | 49 | 49 | 15137 | 2.04% | 0.32% | 2.04% | 0.32% | mature\_B\_cell\_down | Prkcq |
| GO:0090183 | regulation of kidney development | 1.47e-01 | 2.26e-01 | 1 | 49 | 49 | 15137 | 2.04% | 0.32% | 2.04% | 0.32% | mature\_B\_cell\_down | Gata3 |
| GO:0097305 | response to alcohol | 1.47e-01 | 2.26e-01 | 1 | 49 | 49 | 15137 | 2.04% | 0.32% | 2.04% | 0.32% | mature\_B\_cell\_down | Fyn |
| GO:0006641 | triglyceride metabolic process | 1.47e-01 | 2.26e-01 | 1 | 49 | 49 | 15137 | 2.04% | 0.32% | 2.04% | 0.32% | mature\_B\_cell\_down | Lpin1 |
| GO:0002252 | immune effector process | 1.48e-01 | 2.26e-01 | 2 | 49 | 210 | 15137 | 4.08% | 1.39% | 0.95% | 0.32% | mature\_B\_cell\_down | Gata3, Lat |
| GO:0035295 | tube development | 1.48e-01 | 2.27e-01 | 3 | 49 | 412 | 15137 | 6.12% | 2.72% | 0.73% | 0.32% | mature\_B\_cell\_down | Gata3, Nckap1, Notch1 |
| GO:0018958 | phenol-containing compound metabolic process | 1.50e-01 | 2.28e-01 | 1 | 49 | 50 | 15137 | 2.04% | 0.33% | 2.00% | 0.32% | mature\_B\_cell\_down | Gata3 |
| GO:0042307 | positive regulation of protein import into nucleus | 1.50e-01 | 2.28e-01 | 1 | 49 | 50 | 15137 | 2.04% | 0.33% | 2.00% | 0.32% | mature\_B\_cell\_down | Prkcq |
| GO:0006497 | protein lipidation | 1.50e-01 | 2.28e-01 | 1 | 49 | 50 | 15137 | 2.04% | 0.33% | 2.00% | 0.32% | mature\_B\_cell\_down | Zdhhc15 |
| GO:0002831 | regulation of response to biotic stimulus | 1.50e-01 | 2.28e-01 | 1 | 49 | 50 | 15137 | 2.04% | 0.33% | 2.00% | 0.32% | mature\_B\_cell\_down | Cd226 |
| GO:0022610 | biological adhesion | 1.50e-01 | 2.29e-01 | 4 | 49 | 637 | 15137 | 8.16% | 4.21% | 0.63% | 0.32% | mature\_B\_cell\_down | Cd226, Cd96, Selplg, Thy1 |
| GO:0009892 | negative regulation of metabolic process | 1.52e-01 | 2.31e-01 | 7 | 49 | 1372 | 15137 | 14.29% | 9.06% | 0.51% | 0.32% | mature\_B\_cell\_down | Fyn, Gata3, Lpin1, Notch1, Sla2, Thy1, Tnfrsf1a |
| GO:0009880 | embryonic pattern specification | 1.53e-01 | 2.31e-01 | 1 | 49 | 51 | 15137 | 2.04% | 0.34% | 1.96% | 0.32% | mature\_B\_cell\_down | Nckap1 |
| GO:0042490 | mechanoreceptor differentiation | 1.53e-01 | 2.31e-01 | 1 | 49 | 51 | 15137 | 2.04% | 0.34% | 1.96% | 0.32% | mature\_B\_cell\_down | Notch1 |
| GO:0032321 | positive regulation of Rho GTPase activity | 1.53e-01 | 2.31e-01 | 1 | 49 | 51 | 15137 | 2.04% | 0.34% | 1.96% | 0.32% | mature\_B\_cell\_down | Thy1 |
| GO:2000177 | regulation of neural precursor cell proliferation | 1.53e-01 | 2.31e-01 | 1 | 49 | 51 | 15137 | 2.04% | 0.34% | 1.96% | 0.32% | mature\_B\_cell\_down | Notch1 |
| GO:0031341 | regulation of cell killing | 1.55e-01 | 2.35e-01 | 1 | 49 | 52 | 15137 | 2.04% | 0.34% | 1.92% | 0.32% | mature\_B\_cell\_down | Cd226 |
| GO:0034612 | response to tumor necrosis factor | 1.55e-01 | 2.35e-01 | 1 | 49 | 52 | 15137 | 2.04% | 0.34% | 1.92% | 0.32% | mature\_B\_cell\_down | Tnfrsf1a |
| GO:0007033 | vacuole organization | 1.55e-01 | 2.35e-01 | 1 | 49 | 52 | 15137 | 2.04% | 0.34% | 1.92% | 0.32% | mature\_B\_cell\_down | Ctsd |
| GO:0030855 | epithelial cell differentiation | 1.56e-01 | 2.35e-01 | 2 | 49 | 217 | 15137 | 4.08% | 1.43% | 0.92% | 0.32% | mature\_B\_cell\_down | Gata3, Notch1 |
| GO:0003279 | cardiac septum development | 1.58e-01 | 2.38e-01 | 1 | 49 | 53 | 15137 | 2.04% | 0.35% | 1.89% | 0.32% | mature\_B\_cell\_down | Gata3 |
| GO:0032874 | positive regulation of stress-activated MAPK cascade | 1.58e-01 | 2.38e-01 | 1 | 49 | 53 | 15137 | 2.04% | 0.35% | 1.89% | 0.32% | mature\_B\_cell\_down | Cd27 |
| GO:0045685 | regulation of glial cell differentiation | 1.58e-01 | 2.38e-01 | 1 | 49 | 53 | 15137 | 2.04% | 0.35% | 1.89% | 0.32% | mature\_B\_cell\_down | Notch1 |
| GO:0042990 | regulation of transcription factor import into nucleus | 1.58e-01 | 2.38e-01 | 1 | 49 | 53 | 15137 | 2.04% | 0.35% | 1.89% | 0.32% | mature\_B\_cell\_down | Prkcq |
| GO:0045596 | negative regulation of cell differentiation | 1.58e-01 | 2.38e-01 | 3 | 49 | 425 | 15137 | 6.12% | 2.81% | 0.71% | 0.32% | mature\_B\_cell\_down | Gata3, Notch1, Thy1 |
| GO:0016192 | vesicle-mediated transport | 1.59e-01 | 2.39e-01 | 4 | 49 | 652 | 15137 | 8.16% | 4.31% | 0.61% | 0.32% | mature\_B\_cell\_down | Lat, Rinl, Sytl2, Zdhhc15 |
| GO:0007254 | JNK cascade | 1.61e-01 | 2.41e-01 | 1 | 49 | 54 | 15137 | 2.04% | 0.36% | 1.85% | 0.32% | mature\_B\_cell\_down | Tnik |
| GO:0070304 | positive regulation of stress-activated protein kinase signaling cascade | 1.61e-01 | 2.41e-01 | 1 | 49 | 54 | 15137 | 2.04% | 0.36% | 1.85% | 0.32% | mature\_B\_cell\_down | Cd27 |
| GO:0001525 | angiogenesis | 1.63e-01 | 2.43e-01 | 2 | 49 | 223 | 15137 | 4.08% | 1.47% | 0.90% | 0.32% | mature\_B\_cell\_down | Notch1, Thy1 |
| GO:0030335 | positive regulation of cell migration | 1.63e-01 | 2.43e-01 | 2 | 49 | 223 | 15137 | 4.08% | 1.47% | 0.90% | 0.32% | mature\_B\_cell\_down | F2r, Gata3 |
| GO:0033043 | regulation of organelle organization | 1.63e-01 | 2.43e-01 | 3 | 49 | 431 | 15137 | 6.12% | 2.85% | 0.70% | 0.32% | mature\_B\_cell\_down | Gata3, Lpin1, Prkcq |
| GO:0042158 | lipoprotein biosynthetic process | 1.64e-01 | 2.43e-01 | 1 | 49 | 55 | 15137 | 2.04% | 0.36% | 1.82% | 0.32% | mature\_B\_cell\_down | Zdhhc15 |
| GO:0001707 | mesoderm formation | 1.64e-01 | 2.43e-01 | 1 | 49 | 55 | 15137 | 2.04% | 0.36% | 1.82% | 0.32% | mature\_B\_cell\_down | Nckap1 |
| GO:0009605 | response to external stimulus | 1.64e-01 | 2.43e-01 | 4 | 49 | 659 | 15137 | 8.16% | 4.35% | 0.61% | 0.32% | mature\_B\_cell\_down | Ctsd, Fyn, Gata3, Lck |
| GO:0034765 | regulation of ion transmembrane transport | 1.64e-01 | 2.43e-01 | 2 | 49 | 224 | 15137 | 4.08% | 1.48% | 0.89% | 0.32% | mature\_B\_cell\_down | Slc9a3r1, Tmem66 |
| GO:0006874 | cellular calcium ion homeostasis | 1.65e-01 | 2.44e-01 | 2 | 49 | 225 | 15137 | 4.08% | 1.49% | 0.89% | 0.32% | mature\_B\_cell\_down | F2r, Lck |
| GO:0060562 | epithelial tube morphogenesis | 1.65e-01 | 2.44e-01 | 2 | 49 | 225 | 15137 | 4.08% | 1.49% | 0.89% | 0.32% | mature\_B\_cell\_down | Gata3, Nckap1 |
| GO:0006355 | regulation of transcription, DNA-dependent | 1.66e-01 | 2.45e-01 | 11 | 49 | 2472 | 15137 | 22.45% | 16.33% | 0.44% | 0.32% | mature\_B\_cell\_down | F2r, Gata3, Lpin1, Notch1, Prkch, Prkcq, Skap1, Sla2, Tcf7, Tnfrsf1a, Txk |
| GO:0001649 | osteoblast differentiation | 1.66e-01 | 2.46e-01 | 1 | 49 | 56 | 15137 | 2.04% | 0.37% | 1.79% | 0.32% | mature\_B\_cell\_down | Notch1 |
| GO:0048641 | regulation of skeletal muscle tissue development | 1.66e-01 | 2.46e-01 | 1 | 49 | 56 | 15137 | 2.04% | 0.37% | 1.79% | 0.32% | mature\_B\_cell\_down | Notch1 |
| GO:0042542 | response to hydrogen peroxide | 1.66e-01 | 2.46e-01 | 1 | 49 | 56 | 15137 | 2.04% | 0.37% | 1.79% | 0.32% | mature\_B\_cell\_down | Lck |
| GO:2001141 | regulation of RNA biosynthetic process | 1.67e-01 | 2.46e-01 | 11 | 49 | 2476 | 15137 | 22.45% | 16.36% | 0.44% | 0.32% | mature\_B\_cell\_down | F2r, Gata3, Lpin1, Notch1, Prkch, Prkcq, Skap1, Sla2, Tcf7, Tnfrsf1a, Txk |
| GO:0010975 | regulation of neuron projection development | 1.67e-01 | 2.46e-01 | 2 | 49 | 227 | 15137 | 4.08% | 1.50% | 0.88% | 0.32% | mature\_B\_cell\_down | Thy1, Tnik |
| GO:0048646 | anatomical structure formation involved in morphogenesis | 1.67e-01 | 2.47e-01 | 4 | 49 | 665 | 15137 | 8.16% | 4.39% | 0.60% | 0.32% | mature\_B\_cell\_down | Gata3, Nckap1, Notch1, Thy1 |
| GO:2000147 | positive regulation of cell motility | 1.68e-01 | 2.48e-01 | 2 | 49 | 228 | 15137 | 4.08% | 1.51% | 0.88% | 0.32% | mature\_B\_cell\_down | F2r, Gata3 |
| GO:0001656 | metanephros development | 1.69e-01 | 2.48e-01 | 1 | 49 | 57 | 15137 | 2.04% | 0.38% | 1.75% | 0.32% | mature\_B\_cell\_down | Gata3 |
| GO:0001756 | somitogenesis | 1.69e-01 | 2.48e-01 | 1 | 49 | 57 | 15137 | 2.04% | 0.38% | 1.75% | 0.32% | mature\_B\_cell\_down | Nckap1 |
| GO:0051704 | multi-organism process | 1.69e-01 | 2.48e-01 | 4 | 49 | 668 | 15137 | 8.16% | 4.41% | 0.60% | 0.32% | mature\_B\_cell\_down | F2r, Ifih1, Notch1, Tnfrsf1a |
| GO:0010556 | regulation of macromolecule biosynthetic process | 1.69e-01 | 2.48e-01 | 12 | 49 | 2760 | 15137 | 24.49% | 18.23% | 0.43% | 0.32% | mature\_B\_cell\_down | Cd27, F2r, Gata3, Lpin1, Notch1, Prkch, Prkcq, Skap1, Sla2, Tcf7, Tnfrsf1a, Txk |
| GO:0006928 | cellular component movement | 1.71e-01 | 2.51e-01 | 4 | 49 | 671 | 15137 | 8.16% | 4.43% | 0.60% | 0.32% | mature\_B\_cell\_down | Fyn, Gata3, Nckap1, Selplg |
| GO:0006690 | icosanoid metabolic process | 1.72e-01 | 2.51e-01 | 1 | 49 | 58 | 15137 | 2.04% | 0.38% | 1.72% | 0.32% | mature\_B\_cell\_down | Tnfrsf1a |
| GO:0002088 | lens development in camera-type eye | 1.72e-01 | 2.51e-01 | 1 | 49 | 58 | 15137 | 2.04% | 0.38% | 1.72% | 0.32% | mature\_B\_cell\_down | Gata3 |
| GO:0051897 | positive regulation of protein kinase B signaling cascade | 1.72e-01 | 2.51e-01 | 1 | 49 | 58 | 15137 | 2.04% | 0.38% | 1.72% | 0.32% | mature\_B\_cell\_down | Gata3 |
| GO:0071840 | cellular component organization or biogenesis | 1.74e-01 | 2.53e-01 | 13 | 49 | 3053 | 15137 | 26.53% | 20.17% | 0.43% | 0.32% | mature\_B\_cell\_down | Ctsd, F2r, Fyn, Gata3, Lpin1, Nckap1, Notch1, Sept9, Slc9a3r1, Thy1, Tnfrsf1a, Tnik, Zdhhc15 |
| GO:0001889 | liver development | 1.74e-01 | 2.53e-01 | 1 | 49 | 59 | 15137 | 2.04% | 0.39% | 1.69% | 0.32% | mature\_B\_cell\_down | Notch1 |
| GO:0048332 | mesoderm morphogenesis | 1.74e-01 | 2.53e-01 | 1 | 49 | 59 | 15137 | 2.04% | 0.39% | 1.69% | 0.32% | mature\_B\_cell\_down | Nckap1 |
| GO:0050821 | protein stabilization | 1.74e-01 | 2.53e-01 | 1 | 49 | 59 | 15137 | 2.04% | 0.39% | 1.69% | 0.32% | mature\_B\_cell\_down | Nckap1 |
| GO:0043484 | regulation of RNA splicing | 1.74e-01 | 2.53e-01 | 1 | 49 | 59 | 15137 | 2.04% | 0.39% | 1.69% | 0.32% | mature\_B\_cell\_down | Hnrpll |
| GO:0051403 | stress-activated MAPK cascade | 1.74e-01 | 2.53e-01 | 1 | 49 | 59 | 15137 | 2.04% | 0.39% | 1.69% | 0.32% | mature\_B\_cell\_down | Tnik |
| GO:0055074 | calcium ion homeostasis | 1.75e-01 | 2.54e-01 | 2 | 49 | 234 | 15137 | 4.08% | 1.55% | 0.85% | 0.32% | mature\_B\_cell\_down | F2r, Lck |
| GO:0071844 | cellular component assembly at cellular level | 1.76e-01 | 2.55e-01 | 4 | 49 | 678 | 15137 | 8.16% | 4.48% | 0.59% | 0.32% | mature\_B\_cell\_down | Ctsd, Nckap1, Slc9a3r1, Thy1 |
| GO:0051272 | positive regulation of cellular component movement | 1.76e-01 | 2.55e-01 | 2 | 49 | 235 | 15137 | 4.08% | 1.55% | 0.85% | 0.32% | mature\_B\_cell\_down | F2r, Gata3 |
| GO:0034329 | cell junction assembly | 1.77e-01 | 2.55e-01 | 1 | 49 | 60 | 15137 | 2.04% | 0.40% | 1.67% | 0.32% | mature\_B\_cell\_down | Thy1 |
| GO:0048747 | muscle fiber development | 1.77e-01 | 2.55e-01 | 1 | 49 | 60 | 15137 | 2.04% | 0.40% | 1.67% | 0.32% | mature\_B\_cell\_down | F2r |
| GO:0045665 | negative regulation of neuron differentiation | 1.77e-01 | 2.55e-01 | 1 | 49 | 60 | 15137 | 2.04% | 0.40% | 1.67% | 0.32% | mature\_B\_cell\_down | Notch1 |
| GO:0048663 | neuron fate commitment | 1.77e-01 | 2.55e-01 | 1 | 49 | 60 | 15137 | 2.04% | 0.40% | 1.67% | 0.32% | mature\_B\_cell\_down | Notch1 |
| GO:0033559 | unsaturated fatty acid metabolic process | 1.77e-01 | 2.55e-01 | 1 | 49 | 60 | 15137 | 2.04% | 0.40% | 1.67% | 0.32% | mature\_B\_cell\_down | Tnfrsf1a |
| GO:0007423 | sensory organ development | 1.79e-01 | 2.56e-01 | 3 | 49 | 451 | 15137 | 6.12% | 2.98% | 0.67% | 0.32% | mature\_B\_cell\_down | Gata3, Notch1, Thy1 |
| GO:0006639 | acylglycerol metabolic process | 1.80e-01 | 2.56e-01 | 1 | 49 | 61 | 15137 | 2.04% | 0.40% | 1.64% | 0.32% | mature\_B\_cell\_down | Lpin1 |
| GO:0009267 | cellular response to starvation | 1.80e-01 | 2.56e-01 | 1 | 49 | 61 | 15137 | 2.04% | 0.40% | 1.64% | 0.32% | mature\_B\_cell\_down | Ctsd |
| GO:0035050 | embryonic heart tube development | 1.80e-01 | 2.56e-01 | 1 | 49 | 61 | 15137 | 2.04% | 0.40% | 1.64% | 0.32% | mature\_B\_cell\_down | Nckap1 |
| GO:0061008 | hepaticobiliary system development | 1.80e-01 | 2.56e-01 | 1 | 49 | 61 | 15137 | 2.04% | 0.40% | 1.64% | 0.32% | mature\_B\_cell\_down | Notch1 |
| GO:0010923 | negative regulation of phosphatase activity | 1.80e-01 | 2.56e-01 | 1 | 49 | 61 | 15137 | 2.04% | 0.40% | 1.64% | 0.32% | mature\_B\_cell\_down | Sytl2 |
| GO:0034764 | positive regulation of transmembrane transport | 1.80e-01 | 2.56e-01 | 1 | 49 | 61 | 15137 | 2.04% | 0.40% | 1.64% | 0.32% | mature\_B\_cell\_down | Prkcq |
| GO:0016337 | cell-cell adhesion | 1.80e-01 | 2.56e-01 | 2 | 49 | 238 | 15137 | 4.08% | 1.57% | 0.84% | 0.32% | mature\_B\_cell\_down | Selplg, Thy1 |
| GO:0072503 | cellular divalent inorganic cation homeostasis | 1.80e-01 | 2.56e-01 | 2 | 49 | 238 | 15137 | 4.08% | 1.57% | 0.84% | 0.32% | mature\_B\_cell\_down | F2r, Lck |
| GO:0048732 | gland development | 1.80e-01 | 2.56e-01 | 2 | 49 | 238 | 15137 | 4.08% | 1.57% | 0.84% | 0.32% | mature\_B\_cell\_down | Gata3, Notch1 |
| GO:0040017 | positive regulation of locomotion | 1.80e-01 | 2.56e-01 | 2 | 49 | 238 | 15137 | 4.08% | 1.57% | 0.84% | 0.32% | mature\_B\_cell\_down | F2r, Gata3 |
| GO:0048729 | tissue morphogenesis | 1.80e-01 | 2.57e-01 | 3 | 49 | 453 | 15137 | 6.12% | 2.99% | 0.66% | 0.32% | mature\_B\_cell\_down | Gata3, Nckap1, Notch1 |
| GO:0044057 | regulation of system process | 1.81e-01 | 2.58e-01 | 3 | 49 | 454 | 15137 | 6.12% | 3.00% | 0.66% | 0.32% | mature\_B\_cell\_down | F2r, Lck, Slc9a3r1 |
| GO:0050729 | positive regulation of inflammatory response | 1.82e-01 | 2.58e-01 | 1 | 49 | 62 | 15137 | 2.04% | 0.41% | 1.61% | 0.32% | mature\_B\_cell\_down | Tnfrsf1a |
| GO:0002705 | positive regulation of leukocyte mediated immunity | 1.82e-01 | 2.58e-01 | 1 | 49 | 62 | 15137 | 2.04% | 0.41% | 1.61% | 0.32% | mature\_B\_cell\_down | Cd226 |
| GO:0002708 | positive regulation of lymphocyte mediated immunity | 1.82e-01 | 2.58e-01 | 1 | 49 | 62 | 15137 | 2.04% | 0.41% | 1.61% | 0.32% | mature\_B\_cell\_down | Cd226 |
| GO:0046824 | positive regulation of nucleocytoplasmic transport | 1.82e-01 | 2.58e-01 | 1 | 49 | 62 | 15137 | 2.04% | 0.41% | 1.61% | 0.32% | mature\_B\_cell\_down | Prkcq |
| GO:0030510 | regulation of BMP signaling pathway | 1.82e-01 | 2.58e-01 | 1 | 49 | 62 | 15137 | 2.04% | 0.41% | 1.61% | 0.32% | mature\_B\_cell\_down | Notch1 |
| GO:0006631 | fatty acid metabolic process | 1.84e-01 | 2.60e-01 | 2 | 49 | 242 | 15137 | 4.08% | 1.60% | 0.83% | 0.32% | mature\_B\_cell\_down | Lpin1, Tnfrsf1a |
| GO:0044085 | cellular component biogenesis | 1.85e-01 | 2.60e-01 | 6 | 49 | 1190 | 15137 | 12.24% | 7.86% | 0.50% | 0.32% | mature\_B\_cell\_down | Ctsd, Nckap1, Sept9, Slc9a3r1, Thy1, Tnfrsf1a |
| GO:0001708 | cell fate specification | 1.85e-01 | 2.60e-01 | 1 | 49 | 63 | 15137 | 2.04% | 0.42% | 1.59% | 0.32% | mature\_B\_cell\_down | Notch1 |
| GO:0046879 | hormone secretion | 1.85e-01 | 2.60e-01 | 1 | 49 | 63 | 15137 | 2.04% | 0.42% | 1.59% | 0.32% | mature\_B\_cell\_down | Gata3 |
| GO:0050728 | negative regulation of inflammatory response | 1.85e-01 | 2.60e-01 | 1 | 49 | 63 | 15137 | 2.04% | 0.42% | 1.59% | 0.32% | mature\_B\_cell\_down | Gata3 |
| GO:0006638 | neutral lipid metabolic process | 1.85e-01 | 2.60e-01 | 1 | 49 | 63 | 15137 | 2.04% | 0.42% | 1.59% | 0.32% | mature\_B\_cell\_down | Lpin1 |
| GO:0090100 | positive regulation of transmembrane receptor protein serine/threonine kinase signaling pathway | 1.85e-01 | 2.60e-01 | 1 | 49 | 63 | 15137 | 2.04% | 0.42% | 1.59% | 0.32% | mature\_B\_cell\_down | Notch1 |
| GO:0061053 | somite development | 1.85e-01 | 2.60e-01 | 1 | 49 | 63 | 15137 | 2.04% | 0.42% | 1.59% | 0.32% | mature\_B\_cell\_down | Nckap1 |
| GO:0031098 | stress-activated protein kinase signaling cascade | 1.85e-01 | 2.60e-01 | 1 | 49 | 63 | 15137 | 2.04% | 0.42% | 1.59% | 0.32% | mature\_B\_cell\_down | Tnik |
| GO:0010594 | regulation of endothelial cell migration | 1.88e-01 | 2.64e-01 | 1 | 49 | 64 | 15137 | 2.04% | 0.42% | 1.56% | 0.32% | mature\_B\_cell\_down | Gata3 |
| GO:0043010 | camera-type eye development | 1.88e-01 | 2.64e-01 | 2 | 49 | 245 | 15137 | 4.08% | 1.62% | 0.82% | 0.32% | mature\_B\_cell\_down | Gata3, Thy1 |
| GO:0030155 | regulation of cell adhesion | 1.89e-01 | 2.65e-01 | 2 | 49 | 246 | 15137 | 4.08% | 1.63% | 0.81% | 0.32% | mature\_B\_cell\_down | Notch1, Prkcq |
| GO:0006914 | autophagy | 1.90e-01 | 2.65e-01 | 1 | 49 | 65 | 15137 | 2.04% | 0.43% | 1.54% | 0.32% | mature\_B\_cell\_down | Ctsd |
| GO:0030216 | keratinocyte differentiation | 1.90e-01 | 2.65e-01 | 1 | 49 | 65 | 15137 | 2.04% | 0.43% | 1.54% | 0.32% | mature\_B\_cell\_down | Notch1 |
| GO:0043154 | negative regulation of cysteine-type endopeptidase activity involved in apoptotic process | 1.90e-01 | 2.65e-01 | 1 | 49 | 65 | 15137 | 2.04% | 0.43% | 1.54% | 0.32% | mature\_B\_cell\_down | Cd27 |
| GO:0015833 | peptide transport | 1.90e-01 | 2.65e-01 | 1 | 49 | 65 | 15137 | 2.04% | 0.43% | 1.54% | 0.32% | mature\_B\_cell\_down | Slc9a3r1 |
| GO:0042594 | response to starvation | 1.90e-01 | 2.65e-01 | 1 | 49 | 65 | 15137 | 2.04% | 0.43% | 1.54% | 0.32% | mature\_B\_cell\_down | Ctsd |
| GO:0001657 | ureteric bud development | 1.90e-01 | 2.65e-01 | 1 | 49 | 65 | 15137 | 2.04% | 0.43% | 1.54% | 0.32% | mature\_B\_cell\_down | Gata3 |
| GO:0040011 | locomotion | 1.91e-01 | 2.65e-01 | 4 | 49 | 701 | 15137 | 8.16% | 4.63% | 0.57% | 0.32% | mature\_B\_cell\_down | Fyn, Gata3, Nckap1, Selplg |
| GO:0032502 | developmental process | 1.92e-01 | 2.67e-01 | 15 | 49 | 3680 | 15137 | 30.61% | 24.31% | 0.41% | 0.32% | mature\_B\_cell\_down | F2r, Fyn, Gata3, Itk, Lck, Lpin1, Nckap1, Notch1, Prkch, Tcf7, Thy1, Tnik, Txk, Zap70, Zdhhc15 |
| GO:0072507 | divalent inorganic cation homeostasis | 1.93e-01 | 2.67e-01 | 2 | 49 | 249 | 15137 | 4.08% | 1.64% | 0.80% | 0.32% | mature\_B\_cell\_down | F2r, Lck |
| GO:0016358 | dendrite development | 1.93e-01 | 2.67e-01 | 1 | 49 | 66 | 15137 | 2.04% | 0.44% | 1.52% | 0.32% | mature\_B\_cell\_down | Fyn |
| GO:0045444 | fat cell differentiation | 1.93e-01 | 2.67e-01 | 1 | 49 | 66 | 15137 | 2.04% | 0.44% | 1.52% | 0.32% | mature\_B\_cell\_down | Lpin1 |
| GO:0002573 | myeloid leukocyte differentiation | 1.93e-01 | 2.67e-01 | 1 | 49 | 66 | 15137 | 2.04% | 0.44% | 1.52% | 0.32% | mature\_B\_cell\_down | Gata3 |
| GO:0048523 | negative regulation of cellular process | 1.93e-01 | 2.68e-01 | 11 | 49 | 2554 | 15137 | 22.45% | 16.87% | 0.43% | 0.32% | mature\_B\_cell\_down | Cd27, F2r, Fyn, Gata3, Lpin1, Notch1, Prkch, Prkcq, Sla2, Slc9a3r1, Thy1 |
| GO:0007409 | axonogenesis | 1.95e-01 | 2.70e-01 | 2 | 49 | 251 | 15137 | 4.08% | 1.66% | 0.80% | 0.32% | mature\_B\_cell\_down | Gata3, Notch1 |
| GO:0007219 | Notch signaling pathway | 1.96e-01 | 2.70e-01 | 1 | 49 | 67 | 15137 | 2.04% | 0.44% | 1.49% | 0.32% | mature\_B\_cell\_down | Notch1 |
| GO:0008584 | male gonad development | 1.96e-01 | 2.70e-01 | 1 | 49 | 67 | 15137 | 2.04% | 0.44% | 1.49% | 0.32% | mature\_B\_cell\_down | Gata3 |
| GO:0003073 | regulation of systemic arterial blood pressure | 1.96e-01 | 2.70e-01 | 1 | 49 | 67 | 15137 | 2.04% | 0.44% | 1.49% | 0.32% | mature\_B\_cell\_down | F2r |
| GO:0006917 | induction of apoptosis | 1.97e-01 | 2.71e-01 | 2 | 49 | 253 | 15137 | 4.08% | 1.67% | 0.79% | 0.32% | mature\_B\_cell\_down | Cd27, Lck |
| GO:0043409 | negative regulation of MAPK cascade | 1.98e-01 | 2.71e-01 | 1 | 49 | 68 | 15137 | 2.04% | 0.45% | 1.47% | 0.32% | mature\_B\_cell\_down | Slc9a3r1 |
| GO:0009895 | negative regulation of catabolic process | 1.98e-01 | 2.71e-01 | 1 | 49 | 68 | 15137 | 2.04% | 0.45% | 1.47% | 0.32% | mature\_B\_cell\_down | Fyn |
| GO:0031345 | negative regulation of cell projection organization | 1.98e-01 | 2.71e-01 | 1 | 49 | 68 | 15137 | 2.04% | 0.45% | 1.47% | 0.32% | mature\_B\_cell\_down | Thy1 |
| GO:2000117 | negative regulation of cysteine-type endopeptidase activity | 1.98e-01 | 2.71e-01 | 1 | 49 | 68 | 15137 | 2.04% | 0.45% | 1.47% | 0.32% | mature\_B\_cell\_down | Cd27 |
| GO:0045089 | positive regulation of innate immune response | 1.98e-01 | 2.71e-01 | 1 | 49 | 68 | 15137 | 2.04% | 0.45% | 1.47% | 0.32% | mature\_B\_cell\_down | Cd226 |
| GO:0051101 | regulation of DNA binding | 1.98e-01 | 2.71e-01 | 1 | 49 | 68 | 15137 | 2.04% | 0.45% | 1.47% | 0.32% | mature\_B\_cell\_down | Gata3 |
| GO:0050773 | regulation of dendrite development | 1.98e-01 | 2.71e-01 | 1 | 49 | 68 | 15137 | 2.04% | 0.45% | 1.47% | 0.32% | mature\_B\_cell\_down | Tnik |
| GO:0030900 | forebrain development | 1.99e-01 | 2.72e-01 | 2 | 49 | 254 | 15137 | 4.08% | 1.68% | 0.79% | 0.32% | mature\_B\_cell\_down | Fyn, Notch1 |
| GO:0012502 | induction of programmed cell death | 2.00e-01 | 2.73e-01 | 2 | 49 | 255 | 15137 | 4.08% | 1.68% | 0.78% | 0.32% | mature\_B\_cell\_down | Cd27, Lck |
| GO:0050906 | detection of stimulus involved in sensory perception | 2.01e-01 | 2.74e-01 | 1 | 49 | 69 | 15137 | 2.04% | 0.46% | 1.45% | 0.32% | mature\_B\_cell\_down | Fyn |
| GO:0042157 | lipoprotein metabolic process | 2.01e-01 | 2.74e-01 | 1 | 49 | 69 | 15137 | 2.04% | 0.46% | 1.45% | 0.32% | mature\_B\_cell\_down | Zdhhc15 |
| GO:0032680 | regulation of tumor necrosis factor production | 2.01e-01 | 2.74e-01 | 1 | 49 | 69 | 15137 | 2.04% | 0.46% | 1.45% | 0.32% | mature\_B\_cell\_down | Tnfrsf1a |
| GO:0016050 | vesicle organization | 2.01e-01 | 2.74e-01 | 1 | 49 | 69 | 15137 | 2.04% | 0.46% | 1.45% | 0.32% | mature\_B\_cell\_down | Zdhhc15 |
| GO:0030218 | erythrocyte differentiation | 2.03e-01 | 2.76e-01 | 1 | 49 | 70 | 15137 | 2.04% | 0.46% | 1.43% | 0.32% | mature\_B\_cell\_down | Gata3 |
| GO:0009914 | hormone transport | 2.03e-01 | 2.76e-01 | 1 | 49 | 70 | 15137 | 2.04% | 0.46% | 1.43% | 0.32% | mature\_B\_cell\_down | Gata3 |
| GO:0022407 | regulation of cell-cell adhesion | 2.03e-01 | 2.76e-01 | 1 | 49 | 70 | 15137 | 2.04% | 0.46% | 1.43% | 0.32% | mature\_B\_cell\_down | Prkcq |
| GO:0048878 | chemical homeostasis | 2.05e-01 | 2.79e-01 | 4 | 49 | 723 | 15137 | 8.16% | 4.78% | 0.55% | 0.32% | mature\_B\_cell\_down | F2r, Fyn, Lck, Slc9a3r1 |
| GO:0009582 | detection of abiotic stimulus | 2.06e-01 | 2.79e-01 | 1 | 49 | 71 | 15137 | 2.04% | 0.47% | 1.41% | 0.32% | mature\_B\_cell\_down | Fyn |
| GO:0002064 | epithelial cell development | 2.06e-01 | 2.79e-01 | 1 | 49 | 71 | 15137 | 2.04% | 0.47% | 1.41% | 0.32% | mature\_B\_cell\_down | Gata3 |
| GO:0090101 | negative regulation of transmembrane receptor protein serine/threonine kinase signaling pathway | 2.06e-01 | 2.79e-01 | 1 | 49 | 71 | 15137 | 2.04% | 0.47% | 1.41% | 0.32% | mature\_B\_cell\_down | Notch1 |
| GO:0019233 | sensory perception of pain | 2.06e-01 | 2.79e-01 | 1 | 49 | 71 | 15137 | 2.04% | 0.47% | 1.41% | 0.32% | mature\_B\_cell\_down | Fyn |
| GO:0031326 | regulation of cellular biosynthetic process | 2.07e-01 | 2.79e-01 | 12 | 49 | 2872 | 15137 | 24.49% | 18.97% | 0.42% | 0.32% | mature\_B\_cell\_down | Cd27, F2r, Gata3, Lpin1, Notch1, Prkch, Prkcq, Skap1, Sla2, Tcf7, Tnfrsf1a, Txk |
| GO:0032319 | regulation of Rho GTPase activity | 2.09e-01 | 2.81e-01 | 1 | 49 | 72 | 15137 | 2.04% | 0.48% | 1.39% | 0.32% | mature\_B\_cell\_down | Thy1 |
| GO:0051153 | regulation of striated muscle cell differentiation | 2.09e-01 | 2.81e-01 | 1 | 49 | 72 | 15137 | 2.04% | 0.48% | 1.39% | 0.32% | mature\_B\_cell\_down | Notch1 |
| GO:0007264 | small GTPase mediated signal transduction | 2.09e-01 | 2.82e-01 | 2 | 49 | 263 | 15137 | 4.08% | 1.74% | 0.76% | 0.32% | mature\_B\_cell\_down | Lat, Rab37 |
| GO:0043065 | positive regulation of apoptotic process | 2.09e-01 | 2.82e-01 | 3 | 49 | 488 | 15137 | 6.12% | 3.22% | 0.61% | 0.32% | mature\_B\_cell\_down | Cd27, Lck, Notch1 |
| GO:0001667 | ameboidal cell migration | 2.11e-01 | 2.83e-01 | 1 | 49 | 73 | 15137 | 2.04% | 0.48% | 1.37% | 0.32% | mature\_B\_cell\_down | Nckap1 |
| GO:0007160 | cell-matrix adhesion | 2.11e-01 | 2.83e-01 | 1 | 49 | 73 | 15137 | 2.04% | 0.48% | 1.37% | 0.32% | mature\_B\_cell\_down | Thy1 |
| GO:0045981 | positive regulation of nucleotide metabolic process | 2.11e-01 | 2.83e-01 | 1 | 49 | 73 | 15137 | 2.04% | 0.48% | 1.37% | 0.32% | mature\_B\_cell\_down | Ipcef1 |
| GO:1900544 | positive regulation of purine nucleotide metabolic process | 2.11e-01 | 2.83e-01 | 1 | 49 | 73 | 15137 | 2.04% | 0.48% | 1.37% | 0.32% | mature\_B\_cell\_down | Ipcef1 |
| GO:0051130 | positive regulation of cellular component organization | 2.12e-01 | 2.84e-01 | 3 | 49 | 491 | 15137 | 6.12% | 3.24% | 0.61% | 0.32% | mature\_B\_cell\_down | Lpin1, Notch1, Prkcq |
| GO:0043068 | positive regulation of programmed cell death | 2.14e-01 | 2.86e-01 | 3 | 49 | 493 | 15137 | 6.12% | 3.26% | 0.61% | 0.32% | mature\_B\_cell\_down | Cd27, Lck, Notch1 |
| GO:0007229 | integrin-mediated signaling pathway | 2.14e-01 | 2.86e-01 | 1 | 49 | 74 | 15137 | 2.04% | 0.49% | 1.35% | 0.32% | mature\_B\_cell\_down | Lat |
| GO:0090090 | negative regulation of canonical Wnt receptor signaling pathway | 2.14e-01 | 2.86e-01 | 1 | 49 | 74 | 15137 | 2.04% | 0.49% | 1.35% | 0.32% | mature\_B\_cell\_down | Notch1 |
| GO:0006897 | endocytosis | 2.16e-01 | 2.88e-01 | 2 | 49 | 269 | 15137 | 4.08% | 1.78% | 0.74% | 0.32% | mature\_B\_cell\_down | Rinl, Zdhhc15 |
| GO:0001704 | formation of primary germ layer | 2.16e-01 | 2.88e-01 | 1 | 49 | 75 | 15137 | 2.04% | 0.50% | 1.33% | 0.32% | mature\_B\_cell\_down | Nckap1 |
| GO:0032320 | positive regulation of Ras GTPase activity | 2.16e-01 | 2.88e-01 | 1 | 49 | 75 | 15137 | 2.04% | 0.50% | 1.33% | 0.32% | mature\_B\_cell\_down | Thy1 |
| GO:0035282 | segmentation | 2.16e-01 | 2.88e-01 | 1 | 49 | 75 | 15137 | 2.04% | 0.50% | 1.33% | 0.32% | mature\_B\_cell\_down | Nckap1 |
| GO:0070887 | cellular response to chemical stimulus | 2.17e-01 | 2.89e-01 | 5 | 49 | 994 | 15137 | 10.20% | 6.57% | 0.50% | 0.32% | mature\_B\_cell\_down | Gata3, Lpin1, Notch1, Tcf7, Tnfrsf1a |
| GO:0006875 | cellular metal ion homeostasis | 2.18e-01 | 2.89e-01 | 2 | 49 | 270 | 15137 | 4.08% | 1.78% | 0.74% | 0.32% | mature\_B\_cell\_down | F2r, Lck |
| GO:0009889 | regulation of biosynthetic process | 2.18e-01 | 2.90e-01 | 12 | 49 | 2905 | 15137 | 24.49% | 19.19% | 0.41% | 0.32% | mature\_B\_cell\_down | Cd27, F2r, Gata3, Lpin1, Notch1, Prkch, Prkcq, Skap1, Sla2, Tcf7, Tnfrsf1a, Txk |
| GO:0009798 | axis specification | 2.19e-01 | 2.90e-01 | 1 | 49 | 76 | 15137 | 2.04% | 0.50% | 1.32% | 0.32% | mature\_B\_cell\_down | Nckap1 |
| GO:0032869 | cellular response to insulin stimulus | 2.19e-01 | 2.90e-01 | 1 | 49 | 76 | 15137 | 2.04% | 0.50% | 1.32% | 0.32% | mature\_B\_cell\_down | Lpin1 |
| GO:0007368 | determination of left/right symmetry | 2.21e-01 | 2.93e-01 | 1 | 49 | 77 | 15137 | 2.04% | 0.51% | 1.30% | 0.32% | mature\_B\_cell\_down | Notch1 |
| GO:0042552 | myelination | 2.21e-01 | 2.93e-01 | 1 | 49 | 77 | 15137 | 2.04% | 0.51% | 1.30% | 0.32% | mature\_B\_cell\_down | Fyn |
| GO:0060429 | epithelium development | 2.24e-01 | 2.96e-01 | 3 | 49 | 505 | 15137 | 6.12% | 3.34% | 0.59% | 0.32% | mature\_B\_cell\_down | Gata3, Nckap1, Notch1 |
| GO:0034101 | erythrocyte homeostasis | 2.24e-01 | 2.96e-01 | 1 | 49 | 78 | 15137 | 2.04% | 0.52% | 1.28% | 0.32% | mature\_B\_cell\_down | Gata3 |
| GO:0006662 | glycerol ether metabolic process | 2.24e-01 | 2.96e-01 | 1 | 49 | 78 | 15137 | 2.04% | 0.52% | 1.28% | 0.32% | mature\_B\_cell\_down | Lpin1 |
| GO:0044260 | cellular macromolecule metabolic process | 2.25e-01 | 2.96e-01 | 18 | 49 | 4660 | 15137 | 36.73% | 30.79% | 0.39% | 0.32% | mature\_B\_cell\_down | Art2b, F2r, Fyn, Gata3, Hnrpll, Ifih1, Itk, Lck, Lpin1, Notch1, Prkch, Prkcq, Stk39, Tcf7, Tnik, Txk, Zap70, Zdhhc15 |
| GO:0010942 | positive regulation of cell death | 2.26e-01 | 2.97e-01 | 3 | 49 | 508 | 15137 | 6.12% | 3.36% | 0.59% | 0.32% | mature\_B\_cell\_down | Cd27, Lck, Notch1 |
| GO:0008366 | axon ensheathment | 2.26e-01 | 2.97e-01 | 1 | 49 | 79 | 15137 | 2.04% | 0.52% | 1.27% | 0.32% | mature\_B\_cell\_down | Fyn |
| GO:0007272 | ensheathment of neurons | 2.26e-01 | 2.97e-01 | 1 | 49 | 79 | 15137 | 2.04% | 0.52% | 1.27% | 0.32% | mature\_B\_cell\_down | Fyn |
| GO:0009913 | epidermal cell differentiation | 2.26e-01 | 2.97e-01 | 1 | 49 | 79 | 15137 | 2.04% | 0.52% | 1.27% | 0.32% | mature\_B\_cell\_down | Notch1 |
| GO:0001942 | hair follicle development | 2.26e-01 | 2.97e-01 | 1 | 49 | 79 | 15137 | 2.04% | 0.52% | 1.27% | 0.32% | mature\_B\_cell\_down | Notch1 |
| GO:0050680 | negative regulation of epithelial cell proliferation | 2.26e-01 | 2.97e-01 | 1 | 49 | 79 | 15137 | 2.04% | 0.52% | 1.27% | 0.32% | mature\_B\_cell\_down | Gata3 |
| GO:0001843 | neural tube closure | 2.26e-01 | 2.97e-01 | 1 | 49 | 79 | 15137 | 2.04% | 0.52% | 1.27% | 0.32% | mature\_B\_cell\_down | Nckap1 |
| GO:0045860 | positive regulation of protein kinase activity | 2.27e-01 | 2.97e-01 | 2 | 49 | 278 | 15137 | 4.08% | 1.84% | 0.72% | 0.32% | mature\_B\_cell\_down | F2r, Tnik |
| GO:0071842 | cellular component organization at cellular level | 2.28e-01 | 2.98e-01 | 10 | 49 | 2366 | 15137 | 20.41% | 15.63% | 0.42% | 0.32% | mature\_B\_cell\_down | Ctsd, Fyn, Gata3, Lpin1, Nckap1, Notch1, Slc9a3r1, Thy1, Tnik, Zdhhc15 |
| GO:0022405 | hair cycle process | 2.29e-01 | 2.98e-01 | 1 | 49 | 80 | 15137 | 2.04% | 0.53% | 1.25% | 0.32% | mature\_B\_cell\_down | Notch1 |
| GO:0022404 | molting cycle process | 2.29e-01 | 2.98e-01 | 1 | 49 | 80 | 15137 | 2.04% | 0.53% | 1.25% | 0.32% | mature\_B\_cell\_down | Notch1 |
| GO:0007162 | negative regulation of cell adhesion | 2.29e-01 | 2.98e-01 | 1 | 49 | 80 | 15137 | 2.04% | 0.53% | 1.25% | 0.32% | mature\_B\_cell\_down | Notch1 |
| GO:0018904 | organic ether metabolic process | 2.29e-01 | 2.98e-01 | 1 | 49 | 80 | 15137 | 2.04% | 0.53% | 1.25% | 0.32% | mature\_B\_cell\_down | Lpin1 |
| GO:0060606 | tube closure | 2.29e-01 | 2.98e-01 | 1 | 49 | 80 | 15137 | 2.04% | 0.53% | 1.25% | 0.32% | mature\_B\_cell\_down | Nckap1 |
| GO:0051099 | positive regulation of binding | 2.32e-01 | 3.01e-01 | 1 | 49 | 81 | 15137 | 2.04% | 0.54% | 1.23% | 0.32% | mature\_B\_cell\_down | Gata3 |
| GO:0050808 | synapse organization | 2.32e-01 | 3.01e-01 | 1 | 49 | 81 | 15137 | 2.04% | 0.54% | 1.23% | 0.32% | mature\_B\_cell\_down | F2r |
| GO:0001654 | eye development | 2.32e-01 | 3.01e-01 | 2 | 49 | 282 | 15137 | 4.08% | 1.86% | 0.71% | 0.32% | mature\_B\_cell\_down | Gata3, Thy1 |
| GO:0071310 | cellular response to organic substance | 2.32e-01 | 3.01e-01 | 4 | 49 | 762 | 15137 | 8.16% | 5.03% | 0.52% | 0.32% | mature\_B\_cell\_down | Gata3, Lpin1, Tcf7, Tnfrsf1a |
| GO:0048856 | anatomical structure development | 2.33e-01 | 3.03e-01 | 13 | 49 | 3232 | 15137 | 26.53% | 21.35% | 0.40% | 0.32% | mature\_B\_cell\_down | F2r, Fyn, Gata3, Itk, Lck, Nckap1, Notch1, Prkch, Tcf7, Thy1, Tnik, Txk, Zap70 |
| GO:0006606 | protein import into nucleus | 2.34e-01 | 3.03e-01 | 1 | 49 | 82 | 15137 | 2.04% | 0.54% | 1.22% | 0.32% | mature\_B\_cell\_down | F2r |
| GO:0010565 | regulation of cellular ketone metabolic process | 2.34e-01 | 3.03e-01 | 1 | 49 | 82 | 15137 | 2.04% | 0.54% | 1.22% | 0.32% | mature\_B\_cell\_down | Gata3 |
| GO:0055065 | metal ion homeostasis | 2.34e-01 | 3.03e-01 | 2 | 49 | 284 | 15137 | 4.08% | 1.88% | 0.70% | 0.32% | mature\_B\_cell\_down | F2r, Lck |
| GO:0009855 | determination of bilateral symmetry | 2.37e-01 | 3.06e-01 | 1 | 49 | 83 | 15137 | 2.04% | 0.55% | 1.20% | 0.32% | mature\_B\_cell\_down | Notch1 |
| GO:0046546 | development of primary male sexual characteristics | 2.37e-01 | 3.06e-01 | 1 | 49 | 83 | 15137 | 2.04% | 0.55% | 1.20% | 0.32% | mature\_B\_cell\_down | Gata3 |
| GO:0007417 | central nervous system development | 2.38e-01 | 3.07e-01 | 3 | 49 | 522 | 15137 | 6.12% | 3.45% | 0.57% | 0.32% | mature\_B\_cell\_down | Fyn, Notch1, Tcf7 |
| GO:0007265 | Ras protein signal transduction | 2.39e-01 | 3.07e-01 | 1 | 49 | 84 | 15137 | 2.04% | 0.55% | 1.19% | 0.32% | mature\_B\_cell\_down | Lat |
| GO:0031348 | negative regulation of defense response | 2.39e-01 | 3.07e-01 | 1 | 49 | 84 | 15137 | 2.04% | 0.55% | 1.19% | 0.32% | mature\_B\_cell\_down | Gata3 |
| GO:0051170 | nuclear import | 2.39e-01 | 3.07e-01 | 1 | 49 | 84 | 15137 | 2.04% | 0.55% | 1.19% | 0.32% | mature\_B\_cell\_down | F2r |
| GO:0070374 | positive regulation of ERK1 and ERK2 cascade | 2.39e-01 | 3.07e-01 | 1 | 49 | 84 | 15137 | 2.04% | 0.55% | 1.19% | 0.32% | mature\_B\_cell\_down | F2r |
| GO:0014020 | primary neural tube formation | 2.39e-01 | 3.07e-01 | 1 | 49 | 84 | 15137 | 2.04% | 0.55% | 1.19% | 0.32% | mature\_B\_cell\_down | Nckap1 |
| GO:0009799 | specification of symmetry | 2.39e-01 | 3.07e-01 | 1 | 49 | 84 | 15137 | 2.04% | 0.55% | 1.19% | 0.32% | mature\_B\_cell\_down | Notch1 |
| GO:0003002 | regionalization | 2.39e-01 | 3.07e-01 | 2 | 49 | 288 | 15137 | 4.08% | 1.90% | 0.69% | 0.32% | mature\_B\_cell\_down | Nckap1, Notch1 |
| GO:0051090 | regulation of sequence-specific DNA binding transcription factor activity | 2.41e-01 | 3.09e-01 | 2 | 49 | 290 | 15137 | 4.08% | 1.92% | 0.69% | 0.32% | mature\_B\_cell\_down | Prkch, Prkcq |
| GO:0042633 | hair cycle | 2.41e-01 | 3.09e-01 | 1 | 49 | 85 | 15137 | 2.04% | 0.56% | 1.18% | 0.32% | mature\_B\_cell\_down | Notch1 |
| GO:0042303 | molting cycle | 2.41e-01 | 3.09e-01 | 1 | 49 | 85 | 15137 | 2.04% | 0.56% | 1.18% | 0.32% | mature\_B\_cell\_down | Notch1 |
| GO:0051224 | negative regulation of protein transport | 2.41e-01 | 3.09e-01 | 1 | 49 | 85 | 15137 | 2.04% | 0.56% | 1.18% | 0.32% | mature\_B\_cell\_down | F2r |
| GO:0000302 | response to reactive oxygen species | 2.44e-01 | 3.12e-01 | 1 | 49 | 86 | 15137 | 2.04% | 0.57% | 1.16% | 0.32% | mature\_B\_cell\_down | Lck |
| GO:2000112 | regulation of cellular macromolecule biosynthetic process | 2.46e-01 | 3.14e-01 | 11 | 49 | 2694 | 15137 | 22.45% | 17.80% | 0.41% | 0.32% | mature\_B\_cell\_down | F2r, Gata3, Lpin1, Notch1, Prkch, Prkcq, Skap1, Sla2, Tcf7, Tnfrsf1a, Txk |
| GO:0015672 | monovalent inorganic cation transport | 2.46e-01 | 3.14e-01 | 2 | 49 | 294 | 15137 | 4.08% | 1.94% | 0.68% | 0.32% | mature\_B\_cell\_down | Slc12a7, Slc9a3r1 |
| GO:0051093 | negative regulation of developmental process | 2.48e-01 | 3.15e-01 | 3 | 49 | 533 | 15137 | 6.12% | 3.52% | 0.56% | 0.32% | mature\_B\_cell\_down | Gata3, Notch1, Thy1 |
| GO:0048468 | cell development | 2.49e-01 | 3.15e-01 | 5 | 49 | 1046 | 15137 | 10.20% | 6.91% | 0.48% | 0.32% | mature\_B\_cell\_down | F2r, Fyn, Gata3, Notch1, Thy1 |
| GO:0007596 | blood coagulation | 2.49e-01 | 3.15e-01 | 1 | 49 | 88 | 15137 | 2.04% | 0.58% | 1.14% | 0.32% | mature\_B\_cell\_down | F2r |
| GO:0048593 | camera-type eye morphogenesis | 2.49e-01 | 3.15e-01 | 1 | 49 | 88 | 15137 | 2.04% | 0.58% | 1.14% | 0.32% | mature\_B\_cell\_down | Thy1 |
| GO:0050817 | coagulation | 2.49e-01 | 3.15e-01 | 1 | 49 | 88 | 15137 | 2.04% | 0.58% | 1.14% | 0.32% | mature\_B\_cell\_down | F2r |
| GO:0009581 | detection of external stimulus | 2.49e-01 | 3.15e-01 | 1 | 49 | 88 | 15137 | 2.04% | 0.58% | 1.14% | 0.32% | mature\_B\_cell\_down | Fyn |
| GO:0031331 | positive regulation of cellular catabolic process | 2.49e-01 | 3.15e-01 | 1 | 49 | 88 | 15137 | 2.04% | 0.58% | 1.14% | 0.32% | mature\_B\_cell\_down | Ipcef1 |
| GO:0017157 | regulation of exocytosis | 2.49e-01 | 3.15e-01 | 1 | 49 | 88 | 15137 | 2.04% | 0.58% | 1.14% | 0.32% | mature\_B\_cell\_down | Notch1 |
| GO:0055002 | striated muscle cell development | 2.49e-01 | 3.15e-01 | 1 | 49 | 88 | 15137 | 2.04% | 0.58% | 1.14% | 0.32% | mature\_B\_cell\_down | F2r |
| GO:0048771 | tissue remodeling | 2.49e-01 | 3.15e-01 | 1 | 49 | 88 | 15137 | 2.04% | 0.58% | 1.14% | 0.32% | mature\_B\_cell\_down | F2r |
| GO:0000902 | cell morphogenesis | 2.50e-01 | 3.16e-01 | 3 | 49 | 535 | 15137 | 6.12% | 3.53% | 0.56% | 0.32% | mature\_B\_cell\_down | Fyn, Gata3, Notch1 |
| GO:0007599 | hemostasis | 2.51e-01 | 3.17e-01 | 1 | 49 | 89 | 15137 | 2.04% | 0.59% | 1.12% | 0.32% | mature\_B\_cell\_down | F2r |
| GO:0070925 | organelle assembly | 2.51e-01 | 3.17e-01 | 1 | 49 | 89 | 15137 | 2.04% | 0.59% | 1.12% | 0.32% | mature\_B\_cell\_down | Ctsd |
| GO:0051495 | positive regulation of cytoskeleton organization | 2.51e-01 | 3.17e-01 | 1 | 49 | 89 | 15137 | 2.04% | 0.59% | 1.12% | 0.32% | mature\_B\_cell\_down | Prkcq |
| GO:0010921 | regulation of phosphatase activity | 2.51e-01 | 3.17e-01 | 1 | 49 | 89 | 15137 | 2.04% | 0.59% | 1.12% | 0.32% | mature\_B\_cell\_down | Sytl2 |
| GO:0033674 | positive regulation of kinase activity | 2.53e-01 | 3.19e-01 | 2 | 49 | 300 | 15137 | 4.08% | 1.98% | 0.67% | 0.32% | mature\_B\_cell\_down | F2r, Tnik |
| GO:0046661 | male sex differentiation | 2.54e-01 | 3.19e-01 | 1 | 49 | 90 | 15137 | 2.04% | 0.59% | 1.11% | 0.32% | mature\_B\_cell\_down | Gata3 |
| GO:0008360 | regulation of cell shape | 2.54e-01 | 3.19e-01 | 1 | 49 | 90 | 15137 | 2.04% | 0.59% | 1.11% | 0.32% | mature\_B\_cell\_down | Fyn |
| GO:0045892 | negative regulation of transcription, DNA-dependent | 2.55e-01 | 3.21e-01 | 4 | 49 | 795 | 15137 | 8.16% | 5.25% | 0.50% | 0.32% | mature\_B\_cell\_down | Gata3, Lpin1, Notch1, Sla2 |
| GO:0034330 | cell junction organization | 2.56e-01 | 3.21e-01 | 1 | 49 | 91 | 15137 | 2.04% | 0.60% | 1.10% | 0.32% | mature\_B\_cell\_down | Thy1 |
| GO:0014031 | mesenchymal cell development | 2.56e-01 | 3.21e-01 | 1 | 49 | 91 | 15137 | 2.04% | 0.60% | 1.10% | 0.32% | mature\_B\_cell\_down | Notch1 |
| GO:0045766 | positive regulation of angiogenesis | 2.56e-01 | 3.21e-01 | 1 | 49 | 91 | 15137 | 2.04% | 0.60% | 1.10% | 0.32% | mature\_B\_cell\_down | Tnfrsf1a |
| GO:0051147 | regulation of muscle cell differentiation | 2.56e-01 | 3.21e-01 | 1 | 49 | 91 | 15137 | 2.04% | 0.60% | 1.10% | 0.32% | mature\_B\_cell\_down | Notch1 |
| GO:0065008 | regulation of biological quality | 2.57e-01 | 3.21e-01 | 8 | 49 | 1876 | 15137 | 16.33% | 12.39% | 0.43% | 0.32% | mature\_B\_cell\_down | Cd7, F2r, Fyn, Gata3, Lck, Nckap1, Slc12a7, Slc9a3r1 |
| GO:0030003 | cellular cation homeostasis | 2.57e-01 | 3.21e-01 | 2 | 49 | 303 | 15137 | 4.08% | 2.00% | 0.66% | 0.32% | mature\_B\_cell\_down | F2r, Lck |
| GO:0007498 | mesoderm development | 2.59e-01 | 3.23e-01 | 1 | 49 | 92 | 15137 | 2.04% | 0.61% | 1.09% | 0.32% | mature\_B\_cell\_down | Nckap1 |
| GO:2000602 | regulation of interphase of mitotic cell cycle | 2.59e-01 | 3.23e-01 | 1 | 49 | 92 | 15137 | 2.04% | 0.61% | 1.09% | 0.32% | mature\_B\_cell\_down | Prkcq |
| GO:0031669 | cellular response to nutrient levels | 2.61e-01 | 3.25e-01 | 1 | 49 | 93 | 15137 | 2.04% | 0.61% | 1.08% | 0.32% | mature\_B\_cell\_down | Ctsd |
| GO:0006996 | organelle organization | 2.62e-01 | 3.26e-01 | 7 | 49 | 1610 | 15137 | 14.29% | 10.64% | 0.43% | 0.32% | mature\_B\_cell\_down | Ctsd, Lpin1, Nckap1, Slc9a3r1, Thy1, Tnik, Zdhhc15 |
| GO:0040007 | growth | 2.63e-01 | 3.27e-01 | 2 | 49 | 308 | 15137 | 4.08% | 2.03% | 0.65% | 0.32% | mature\_B\_cell\_down | Gata3, Notch1 |
| GO:0071375 | cellular response to peptide hormone stimulus | 2.63e-01 | 3.27e-01 | 1 | 49 | 94 | 15137 | 2.04% | 0.62% | 1.06% | 0.32% | mature\_B\_cell\_down | Lpin1 |
| GO:0010810 | regulation of cell-substrate adhesion | 2.63e-01 | 3.27e-01 | 1 | 49 | 94 | 15137 | 2.04% | 0.62% | 1.06% | 0.32% | mature\_B\_cell\_down | Notch1 |
| GO:0044242 | cellular lipid catabolic process | 2.66e-01 | 3.29e-01 | 1 | 49 | 95 | 15137 | 2.04% | 0.63% | 1.05% | 0.32% | mature\_B\_cell\_down | Lpin1 |
| GO:0002706 | regulation of lymphocyte mediated immunity | 2.66e-01 | 3.29e-01 | 1 | 49 | 95 | 15137 | 2.04% | 0.63% | 1.05% | 0.32% | mature\_B\_cell\_down | Cd226 |
| GO:0031647 | regulation of protein stability | 2.66e-01 | 3.29e-01 | 1 | 49 | 95 | 15137 | 2.04% | 0.63% | 1.05% | 0.32% | mature\_B\_cell\_down | Nckap1 |
| GO:0048514 | blood vessel morphogenesis | 2.67e-01 | 3.30e-01 | 2 | 49 | 311 | 15137 | 4.08% | 2.05% | 0.64% | 0.32% | mature\_B\_cell\_down | Notch1, Thy1 |
| GO:0051347 | positive regulation of transferase activity | 2.68e-01 | 3.31e-01 | 2 | 49 | 312 | 15137 | 4.08% | 2.06% | 0.64% | 0.32% | mature\_B\_cell\_down | F2r, Tnik |
| GO:0007188 | adenylate cyclase-modulating G-protein coupled receptor signaling pathway | 2.68e-01 | 3.31e-01 | 1 | 49 | 96 | 15137 | 2.04% | 0.63% | 1.04% | 0.32% | mature\_B\_cell\_down | Slc9a3r1 |
| GO:0042472 | inner ear morphogenesis | 2.68e-01 | 3.31e-01 | 1 | 49 | 96 | 15137 | 2.04% | 0.63% | 1.04% | 0.32% | mature\_B\_cell\_down | Gata3 |
| GO:0007275 | multicellular organismal development | 2.68e-01 | 3.31e-01 | 13 | 49 | 3327 | 15137 | 26.53% | 21.98% | 0.39% | 0.32% | mature\_B\_cell\_down | F2r, Fyn, Gata3, Itk, Lck, Nckap1, Notch1, Prkch, Tcf7, Thy1, Tnik, Txk, Zap70 |
| GO:0051346 | negative regulation of hydrolase activity | 2.70e-01 | 3.33e-01 | 2 | 49 | 314 | 15137 | 4.08% | 2.07% | 0.64% | 0.32% | mature\_B\_cell\_down | Cd27, Sytl2 |
| GO:0006865 | amino acid transport | 2.71e-01 | 3.33e-01 | 1 | 49 | 97 | 15137 | 2.04% | 0.64% | 1.03% | 0.32% | mature\_B\_cell\_down | Slc9a3r1 |
| GO:0050770 | regulation of axonogenesis | 2.71e-01 | 3.33e-01 | 1 | 49 | 97 | 15137 | 2.04% | 0.64% | 1.03% | 0.32% | mature\_B\_cell\_down | Thy1 |
| GO:0044087 | regulation of cellular component biogenesis | 2.73e-01 | 3.35e-01 | 2 | 49 | 316 | 15137 | 4.08% | 2.09% | 0.63% | 0.32% | mature\_B\_cell\_down | Prkch, Prkcq |
| GO:0006576 | cellular biogenic amine metabolic process | 2.73e-01 | 3.35e-01 | 1 | 49 | 98 | 15137 | 2.04% | 0.65% | 1.02% | 0.32% | mature\_B\_cell\_down | Gata3 |
| GO:0022612 | gland morphogenesis | 2.75e-01 | 3.37e-01 | 1 | 49 | 99 | 15137 | 2.04% | 0.65% | 1.01% | 0.32% | mature\_B\_cell\_down | Notch1 |
| GO:0043524 | negative regulation of neuron apoptotic process | 2.75e-01 | 3.37e-01 | 1 | 49 | 99 | 15137 | 2.04% | 0.65% | 1.01% | 0.32% | mature\_B\_cell\_down | F2r |
| GO:0001841 | neural tube formation | 2.75e-01 | 3.37e-01 | 1 | 49 | 99 | 15137 | 2.04% | 0.65% | 1.01% | 0.32% | mature\_B\_cell\_down | Nckap1 |
| GO:0016202 | regulation of striated muscle tissue development | 2.75e-01 | 3.37e-01 | 1 | 49 | 99 | 15137 | 2.04% | 0.65% | 1.01% | 0.32% | mature\_B\_cell\_down | Notch1 |
| GO:0051253 | negative regulation of RNA metabolic process | 2.77e-01 | 3.39e-01 | 4 | 49 | 825 | 15137 | 8.16% | 5.45% | 0.48% | 0.32% | mature\_B\_cell\_down | Gata3, Lpin1, Notch1, Sla2 |
| GO:0010001 | glial cell differentiation | 2.78e-01 | 3.39e-01 | 1 | 49 | 100 | 15137 | 2.04% | 0.66% | 1.00% | 0.32% | mature\_B\_cell\_down | Notch1 |
| GO:0019228 | regulation of action potential in neuron | 2.78e-01 | 3.39e-01 | 1 | 49 | 100 | 15137 | 2.04% | 0.66% | 1.00% | 0.32% | mature\_B\_cell\_down | Fyn |
| GO:0048634 | regulation of muscle organ development | 2.78e-01 | 3.39e-01 | 1 | 49 | 100 | 15137 | 2.04% | 0.66% | 1.00% | 0.32% | mature\_B\_cell\_down | Notch1 |
| GO:0071841 | cellular component organization or biogenesis at cellular level | 2.79e-01 | 3.41e-01 | 10 | 49 | 2490 | 15137 | 20.41% | 16.45% | 0.40% | 0.32% | mature\_B\_cell\_down | Ctsd, Fyn, Gata3, Lpin1, Nckap1, Notch1, Slc9a3r1, Thy1, Tnik, Zdhhc15 |
| GO:0030178 | negative regulation of Wnt receptor signaling pathway | 2.80e-01 | 3.41e-01 | 1 | 49 | 101 | 15137 | 2.04% | 0.67% | 0.99% | 0.32% | mature\_B\_cell\_down | Notch1 |
| GO:0042113 | B cell activation | 2.82e-01 | 3.44e-01 | 1 | 49 | 102 | 15137 | 2.04% | 0.67% | 0.98% | 0.32% | mature\_B\_cell\_down | Cd27 |
| GO:0032787 | monocarboxylic acid metabolic process | 2.84e-01 | 3.45e-01 | 2 | 49 | 325 | 15137 | 4.08% | 2.15% | 0.62% | 0.32% | mature\_B\_cell\_down | Lpin1, Tnfrsf1a |
| GO:0048762 | mesenchymal cell differentiation | 2.85e-01 | 3.45e-01 | 1 | 49 | 103 | 15137 | 2.04% | 0.68% | 0.97% | 0.32% | mature\_B\_cell\_down | Notch1 |
| GO:0009791 | post-embryonic development | 2.85e-01 | 3.45e-01 | 1 | 49 | 103 | 15137 | 2.04% | 0.68% | 0.97% | 0.32% | mature\_B\_cell\_down | Gata3 |
| GO:2000241 | regulation of reproductive process | 2.85e-01 | 3.45e-01 | 1 | 49 | 103 | 15137 | 2.04% | 0.68% | 0.97% | 0.32% | mature\_B\_cell\_down | Notch1 |
| GO:0032868 | response to insulin stimulus | 2.85e-01 | 3.45e-01 | 1 | 49 | 103 | 15137 | 2.04% | 0.68% | 0.97% | 0.32% | mature\_B\_cell\_down | Lpin1 |
| GO:0043547 | positive regulation of GTPase activity | 2.87e-01 | 3.48e-01 | 1 | 49 | 104 | 15137 | 2.04% | 0.69% | 0.96% | 0.32% | mature\_B\_cell\_down | Thy1 |
| GO:0043123 | positive regulation of I-kappaB kinase/NF-kappaB cascade | 2.87e-01 | 3.48e-01 | 1 | 49 | 104 | 15137 | 2.04% | 0.69% | 0.96% | 0.32% | mature\_B\_cell\_down | Apol7b |
| GO:0045667 | regulation of osteoblast differentiation | 2.89e-01 | 3.50e-01 | 1 | 49 | 105 | 15137 | 2.04% | 0.69% | 0.95% | 0.32% | mature\_B\_cell\_down | Notch1 |
| GO:0060041 | retina development in camera-type eye | 2.89e-01 | 3.50e-01 | 1 | 49 | 105 | 15137 | 2.04% | 0.69% | 0.95% | 0.32% | mature\_B\_cell\_down | Thy1 |
| GO:0007187 | G-protein coupled receptor signaling pathway, coupled to cyclic nucleotide second messenger | 2.92e-01 | 3.53e-01 | 1 | 49 | 106 | 15137 | 2.04% | 0.70% | 0.94% | 0.32% | mature\_B\_cell\_down | Slc9a3r1 |
| GO:0034613 | cellular protein localization | 2.94e-01 | 3.55e-01 | 3 | 49 | 585 | 15137 | 6.12% | 3.86% | 0.51% | 0.32% | mature\_B\_cell\_down | F2r, Slc9a3r1, Sytl2 |
| GO:0031589 | cell-substrate adhesion | 2.96e-01 | 3.57e-01 | 1 | 49 | 108 | 15137 | 2.04% | 0.71% | 0.93% | 0.32% | mature\_B\_cell\_down | Thy1 |
| GO:0035270 | endocrine system development | 2.96e-01 | 3.57e-01 | 1 | 49 | 108 | 15137 | 2.04% | 0.71% | 0.93% | 0.32% | mature\_B\_cell\_down | Gata3 |
| GO:0010741 | negative regulation of intracellular protein kinase cascade | 2.96e-01 | 3.57e-01 | 1 | 49 | 108 | 15137 | 2.04% | 0.71% | 0.93% | 0.32% | mature\_B\_cell\_down | Slc9a3r1 |
| GO:0035303 | regulation of dephosphorylation | 2.96e-01 | 3.57e-01 | 1 | 49 | 108 | 15137 | 2.04% | 0.71% | 0.93% | 0.32% | mature\_B\_cell\_down | Sytl2 |
| GO:0070727 | cellular macromolecule localization | 2.97e-01 | 3.57e-01 | 3 | 49 | 588 | 15137 | 6.12% | 3.88% | 0.51% | 0.32% | mature\_B\_cell\_down | F2r, Slc9a3r1, Sytl2 |
| GO:0002443 | leukocyte mediated immunity | 2.99e-01 | 3.59e-01 | 1 | 49 | 109 | 15137 | 2.04% | 0.72% | 0.92% | 0.32% | mature\_B\_cell\_down | Lat |
| GO:0055001 | muscle cell development | 2.99e-01 | 3.59e-01 | 1 | 49 | 109 | 15137 | 2.04% | 0.72% | 0.92% | 0.32% | mature\_B\_cell\_down | F2r |
| GO:0032989 | cellular component morphogenesis | 2.99e-01 | 3.59e-01 | 3 | 49 | 591 | 15137 | 6.12% | 3.90% | 0.51% | 0.32% | mature\_B\_cell\_down | Fyn, Gata3, Notch1 |
| GO:0048469 | cell maturation | 3.05e-01 | 3.66e-01 | 1 | 49 | 112 | 15137 | 2.04% | 0.74% | 0.89% | 0.32% | mature\_B\_cell\_down | Gata3 |
| GO:0035113 | embryonic appendage morphogenesis | 3.08e-01 | 3.68e-01 | 1 | 49 | 113 | 15137 | 2.04% | 0.75% | 0.88% | 0.32% | mature\_B\_cell\_down | Notch1 |
| GO:0030326 | embryonic limb morphogenesis | 3.08e-01 | 3.68e-01 | 1 | 49 | 113 | 15137 | 2.04% | 0.75% | 0.88% | 0.32% | mature\_B\_cell\_down | Notch1 |
| GO:0051259 | protein oligomerization | 3.09e-01 | 3.69e-01 | 2 | 49 | 346 | 15137 | 4.08% | 2.29% | 0.58% | 0.32% | mature\_B\_cell\_down | Sept9, Tnfrsf1a |
| GO:0031668 | cellular response to extracellular stimulus | 3.12e-01 | 3.73e-01 | 1 | 49 | 115 | 15137 | 2.04% | 0.76% | 0.87% | 0.32% | mature\_B\_cell\_down | Ctsd |
| GO:0042471 | ear morphogenesis | 3.12e-01 | 3.73e-01 | 1 | 49 | 115 | 15137 | 2.04% | 0.76% | 0.87% | 0.32% | mature\_B\_cell\_down | Gata3 |
| GO:0002699 | positive regulation of immune effector process | 3.12e-01 | 3.73e-01 | 1 | 49 | 115 | 15137 | 2.04% | 0.76% | 0.87% | 0.32% | mature\_B\_cell\_down | Cd226 |
| GO:0032102 | negative regulation of response to external stimulus | 3.14e-01 | 3.74e-01 | 1 | 49 | 116 | 15137 | 2.04% | 0.77% | 0.86% | 0.32% | mature\_B\_cell\_down | Gata3 |
| GO:0034504 | protein localization to nucleus | 3.14e-01 | 3.74e-01 | 1 | 49 | 116 | 15137 | 2.04% | 0.77% | 0.86% | 0.32% | mature\_B\_cell\_down | F2r |
| GO:0002703 | regulation of leukocyte mediated immunity | 3.14e-01 | 3.74e-01 | 1 | 49 | 116 | 15137 | 2.04% | 0.77% | 0.86% | 0.32% | mature\_B\_cell\_down | Cd226 |
| GO:0007519 | skeletal muscle tissue development | 3.14e-01 | 3.74e-01 | 1 | 49 | 116 | 15137 | 2.04% | 0.77% | 0.86% | 0.32% | mature\_B\_cell\_down | F2r |
| GO:0050679 | positive regulation of epithelial cell proliferation | 3.17e-01 | 3.76e-01 | 1 | 49 | 117 | 15137 | 2.04% | 0.77% | 0.85% | 0.32% | mature\_B\_cell\_down | Notch1 |
| GO:0042063 | gliogenesis | 3.19e-01 | 3.79e-01 | 1 | 49 | 118 | 15137 | 2.04% | 0.78% | 0.85% | 0.32% | mature\_B\_cell\_down | Notch1 |
| GO:2000113 | negative regulation of cellular macromolecule biosynthetic process | 3.20e-01 | 3.80e-01 | 4 | 49 | 883 | 15137 | 8.16% | 5.83% | 0.45% | 0.32% | mature\_B\_cell\_down | Gata3, Lpin1, Notch1, Sla2 |
| GO:0003001 | generation of a signal involved in cell-cell signaling | 3.21e-01 | 3.80e-01 | 1 | 49 | 119 | 15137 | 2.04% | 0.79% | 0.84% | 0.32% | mature\_B\_cell\_down | Gata3 |
| GO:0009896 | positive regulation of catabolic process | 3.21e-01 | 3.80e-01 | 1 | 49 | 119 | 15137 | 2.04% | 0.79% | 0.84% | 0.32% | mature\_B\_cell\_down | Ipcef1 |
| GO:0043543 | protein acylation | 3.21e-01 | 3.80e-01 | 1 | 49 | 119 | 15137 | 2.04% | 0.79% | 0.84% | 0.32% | mature\_B\_cell\_down | Zdhhc15 |
| GO:0046328 | regulation of JNK cascade | 3.21e-01 | 3.80e-01 | 1 | 49 | 119 | 15137 | 2.04% | 0.79% | 0.84% | 0.32% | mature\_B\_cell\_down | Cd27 |
| GO:0023061 | signal release | 3.21e-01 | 3.80e-01 | 1 | 49 | 119 | 15137 | 2.04% | 0.79% | 0.84% | 0.32% | mature\_B\_cell\_down | Gata3 |
| GO:0009309 | amine biosynthetic process | 3.23e-01 | 3.82e-01 | 1 | 49 | 120 | 15137 | 2.04% | 0.79% | 0.83% | 0.32% | mature\_B\_cell\_down | Gata3 |
| GO:0045934 | negative regulation of nucleobase-containing compound metabolic process | 3.24e-01 | 3.82e-01 | 4 | 49 | 888 | 15137 | 8.16% | 5.87% | 0.45% | 0.32% | mature\_B\_cell\_down | Gata3, Lpin1, Notch1, Sla2 |
| GO:0055080 | cation homeostasis | 3.24e-01 | 3.83e-01 | 2 | 49 | 359 | 15137 | 4.08% | 2.37% | 0.56% | 0.32% | mature\_B\_cell\_down | F2r, Lck |
| GO:0048592 | eye morphogenesis | 3.26e-01 | 3.83e-01 | 1 | 49 | 121 | 15137 | 2.04% | 0.80% | 0.83% | 0.32% | mature\_B\_cell\_down | Thy1 |
| GO:0060828 | regulation of canonical Wnt receptor signaling pathway | 3.26e-01 | 3.83e-01 | 1 | 49 | 121 | 15137 | 2.04% | 0.80% | 0.83% | 0.32% | mature\_B\_cell\_down | Notch1 |
| GO:0007369 | gastrulation | 3.30e-01 | 3.88e-01 | 1 | 49 | 123 | 15137 | 2.04% | 0.81% | 0.81% | 0.32% | mature\_B\_cell\_down | Nckap1 |
| GO:0060485 | mesenchyme development | 3.30e-01 | 3.88e-01 | 1 | 49 | 123 | 15137 | 2.04% | 0.81% | 0.81% | 0.32% | mature\_B\_cell\_down | Notch1 |
| GO:0060538 | skeletal muscle organ development | 3.30e-01 | 3.88e-01 | 1 | 49 | 123 | 15137 | 2.04% | 0.81% | 0.81% | 0.32% | mature\_B\_cell\_down | F2r |
| GO:0006886 | intracellular protein transport | 3.32e-01 | 3.89e-01 | 2 | 49 | 365 | 15137 | 4.08% | 2.41% | 0.55% | 0.32% | mature\_B\_cell\_down | F2r, Sytl2 |
| GO:0051172 | negative regulation of nitrogen compound metabolic process | 3.33e-01 | 3.91e-01 | 4 | 49 | 901 | 15137 | 8.16% | 5.95% | 0.44% | 0.32% | mature\_B\_cell\_down | Gata3, Lpin1, Notch1, Sla2 |
| GO:0071496 | cellular response to external stimulus | 3.34e-01 | 3.91e-01 | 1 | 49 | 125 | 15137 | 2.04% | 0.83% | 0.80% | 0.32% | mature\_B\_cell\_down | Ctsd |
| GO:0071363 | cellular response to growth factor stimulus | 3.34e-01 | 3.91e-01 | 1 | 49 | 125 | 15137 | 2.04% | 0.83% | 0.80% | 0.32% | mature\_B\_cell\_down | Gata3 |
| GO:0001666 | response to hypoxia | 3.34e-01 | 3.91e-01 | 1 | 49 | 125 | 15137 | 2.04% | 0.83% | 0.80% | 0.32% | mature\_B\_cell\_down | Notch1 |
| GO:0051301 | cell division | 3.36e-01 | 3.93e-01 | 2 | 49 | 369 | 15137 | 4.08% | 2.44% | 0.54% | 0.32% | mature\_B\_cell\_down | Notch1, Sept9 |
| GO:0046395 | carboxylic acid catabolic process | 3.37e-01 | 3.93e-01 | 1 | 49 | 126 | 15137 | 2.04% | 0.83% | 0.79% | 0.32% | mature\_B\_cell\_down | Lpin1 |
| GO:0016054 | organic acid catabolic process | 3.37e-01 | 3.93e-01 | 1 | 49 | 126 | 15137 | 2.04% | 0.83% | 0.79% | 0.32% | mature\_B\_cell\_down | Lpin1 |
| GO:0001508 | regulation of action potential | 3.37e-01 | 3.93e-01 | 1 | 49 | 126 | 15137 | 2.04% | 0.83% | 0.79% | 0.32% | mature\_B\_cell\_down | Fyn |
| GO:0046907 | intracellular transport | 3.38e-01 | 3.94e-01 | 3 | 49 | 634 | 15137 | 6.12% | 4.19% | 0.47% | 0.32% | mature\_B\_cell\_down | F2r, Sytl2, Vps26b |
| GO:0009790 | embryo development | 3.38e-01 | 3.94e-01 | 4 | 49 | 907 | 15137 | 8.16% | 5.99% | 0.44% | 0.32% | mature\_B\_cell\_down | Gata3, Nckap1, Notch1, Tcf7 |
| GO:0010558 | negative regulation of macromolecule biosynthetic process | 3.39e-01 | 3.94e-01 | 4 | 49 | 908 | 15137 | 8.16% | 6.00% | 0.44% | 0.32% | mature\_B\_cell\_down | Gata3, Lpin1, Notch1, Sla2 |
| GO:0017038 | protein import | 3.39e-01 | 3.94e-01 | 1 | 49 | 127 | 15137 | 2.04% | 0.84% | 0.79% | 0.32% | mature\_B\_cell\_down | F2r |
| GO:0001568 | blood vessel development | 3.40e-01 | 3.95e-01 | 2 | 49 | 372 | 15137 | 4.08% | 2.46% | 0.54% | 0.32% | mature\_B\_cell\_down | Notch1, Thy1 |
| GO:0051248 | negative regulation of protein metabolic process | 3.40e-01 | 3.95e-01 | 2 | 49 | 372 | 15137 | 4.08% | 2.46% | 0.54% | 0.32% | mature\_B\_cell\_down | Fyn, Thy1 |
| GO:0006814 | sodium ion transport | 3.41e-01 | 3.95e-01 | 1 | 49 | 128 | 15137 | 2.04% | 0.85% | 0.78% | 0.32% | mature\_B\_cell\_down | Slc9a3r1 |
| GO:0006813 | potassium ion transport | 3.45e-01 | 4.00e-01 | 1 | 49 | 130 | 15137 | 2.04% | 0.86% | 0.77% | 0.32% | mature\_B\_cell\_down | Slc12a7 |
| GO:0070848 | response to growth factor stimulus | 3.49e-01 | 4.05e-01 | 1 | 49 | 132 | 15137 | 2.04% | 0.87% | 0.76% | 0.32% | mature\_B\_cell\_down | Gata3 |
| GO:0006310 | DNA recombination | 3.52e-01 | 4.06e-01 | 1 | 49 | 133 | 15137 | 2.04% | 0.88% | 0.75% | 0.32% | mature\_B\_cell\_down | Tcf7 |
| GO:0006469 | negative regulation of protein kinase activity | 3.52e-01 | 4.06e-01 | 1 | 49 | 133 | 15137 | 2.04% | 0.88% | 0.75% | 0.32% | mature\_B\_cell\_down | Thy1 |
| GO:0043122 | regulation of I-kappaB kinase/NF-kappaB cascade | 3.52e-01 | 4.06e-01 | 1 | 49 | 133 | 15137 | 2.04% | 0.88% | 0.75% | 0.32% | mature\_B\_cell\_down | Apol7b |
| GO:0032872 | regulation of stress-activated MAPK cascade | 3.52e-01 | 4.06e-01 | 1 | 49 | 133 | 15137 | 2.04% | 0.88% | 0.75% | 0.32% | mature\_B\_cell\_down | Cd27 |
| GO:0000165 | MAPK cascade | 3.54e-01 | 4.08e-01 | 1 | 49 | 134 | 15137 | 2.04% | 0.89% | 0.75% | 0.32% | mature\_B\_cell\_down | Tnik |
| GO:0070302 | regulation of stress-activated protein kinase signaling cascade | 3.54e-01 | 4.08e-01 | 1 | 49 | 134 | 15137 | 2.04% | 0.89% | 0.75% | 0.32% | mature\_B\_cell\_down | Cd27 |
| GO:0031324 | negative regulation of cellular metabolic process | 3.55e-01 | 4.09e-01 | 5 | 49 | 1211 | 15137 | 10.20% | 8.00% | 0.41% | 0.32% | mature\_B\_cell\_down | Gata3, Lpin1, Notch1, Sla2, Thy1 |
| GO:0032103 | positive regulation of response to external stimulus | 3.60e-01 | 4.14e-01 | 1 | 49 | 137 | 15137 | 2.04% | 0.91% | 0.73% | 0.32% | mature\_B\_cell\_down | Tnfrsf1a |
| GO:0009615 | response to virus | 3.60e-01 | 4.14e-01 | 1 | 49 | 137 | 15137 | 2.04% | 0.91% | 0.73% | 0.32% | mature\_B\_cell\_down | Ifih1 |
| GO:0015837 | amine transport | 3.62e-01 | 4.16e-01 | 1 | 49 | 138 | 15137 | 2.04% | 0.91% | 0.72% | 0.32% | mature\_B\_cell\_down | Slc9a3r1 |
| GO:0090092 | regulation of transmembrane receptor protein serine/threonine kinase signaling pathway | 3.64e-01 | 4.18e-01 | 1 | 49 | 139 | 15137 | 2.04% | 0.92% | 0.72% | 0.32% | mature\_B\_cell\_down | Notch1 |
| GO:0050954 | sensory perception of mechanical stimulus | 3.64e-01 | 4.18e-01 | 1 | 49 | 139 | 15137 | 2.04% | 0.92% | 0.72% | 0.32% | mature\_B\_cell\_down | Fyn |
| GO:0019226 | transmission of nerve impulse | 3.65e-01 | 4.18e-01 | 2 | 49 | 393 | 15137 | 4.08% | 2.60% | 0.51% | 0.32% | mature\_B\_cell\_down | Fyn, Zdhhc15 |
| GO:0035107 | appendage morphogenesis | 3.68e-01 | 4.21e-01 | 1 | 49 | 141 | 15137 | 2.04% | 0.93% | 0.71% | 0.32% | mature\_B\_cell\_down | Notch1 |
| GO:0048754 | branching morphogenesis of a tube | 3.68e-01 | 4.21e-01 | 1 | 49 | 141 | 15137 | 2.04% | 0.93% | 0.71% | 0.32% | mature\_B\_cell\_down | Notch1 |
| GO:0035108 | limb morphogenesis | 3.68e-01 | 4.21e-01 | 1 | 49 | 141 | 15137 | 2.04% | 0.93% | 0.71% | 0.32% | mature\_B\_cell\_down | Notch1 |
| GO:0008217 | regulation of blood pressure | 3.68e-01 | 4.21e-01 | 1 | 49 | 141 | 15137 | 2.04% | 0.93% | 0.71% | 0.32% | mature\_B\_cell\_down | F2r |
| GO:0031327 | negative regulation of cellular biosynthetic process | 3.69e-01 | 4.21e-01 | 4 | 49 | 948 | 15137 | 8.16% | 6.26% | 0.42% | 0.32% | mature\_B\_cell\_down | Gata3, Lpin1, Notch1, Sla2 |
| GO:0042221 | response to chemical stimulus | 3.69e-01 | 4.21e-01 | 8 | 49 | 2101 | 15137 | 16.33% | 13.88% | 0.38% | 0.32% | mature\_B\_cell\_down | F2r, Fyn, Gata3, Lck, Lpin1, Notch1, Tcf7, Tnfrsf1a |
| GO:0042176 | regulation of protein catabolic process | 3.70e-01 | 4.23e-01 | 1 | 49 | 142 | 15137 | 2.04% | 0.94% | 0.70% | 0.32% | mature\_B\_cell\_down | Fyn |
| GO:0051606 | detection of stimulus | 3.72e-01 | 4.25e-01 | 1 | 49 | 143 | 15137 | 2.04% | 0.94% | 0.70% | 0.32% | mature\_B\_cell\_down | Fyn |
| GO:0035637 | multicellular organismal signaling | 3.73e-01 | 4.25e-01 | 2 | 49 | 400 | 15137 | 4.08% | 2.64% | 0.50% | 0.32% | mature\_B\_cell\_down | Fyn, Zdhhc15 |
| GO:0007411 | axon guidance | 3.74e-01 | 4.26e-01 | 1 | 49 | 144 | 15137 | 2.04% | 0.95% | 0.69% | 0.32% | mature\_B\_cell\_down | Gata3 |
| GO:0033673 | negative regulation of kinase activity | 3.74e-01 | 4.26e-01 | 1 | 49 | 144 | 15137 | 2.04% | 0.95% | 0.69% | 0.32% | mature\_B\_cell\_down | Thy1 |
| GO:0007389 | pattern specification process | 3.79e-01 | 4.31e-01 | 2 | 49 | 405 | 15137 | 4.08% | 2.68% | 0.49% | 0.32% | mature\_B\_cell\_down | Nckap1, Notch1 |
| GO:0006184 | GTP catabolic process | 3.81e-01 | 4.31e-01 | 1 | 49 | 147 | 15137 | 2.04% | 0.97% | 0.68% | 0.32% | mature\_B\_cell\_down | Rab37 |
| GO:0019221 | cytokine-mediated signaling pathway | 3.81e-01 | 4.31e-01 | 1 | 49 | 147 | 15137 | 2.04% | 0.97% | 0.68% | 0.32% | mature\_B\_cell\_down | Tnfrsf1a |
| GO:0010038 | response to metal ion | 3.81e-01 | 4.31e-01 | 1 | 49 | 147 | 15137 | 2.04% | 0.97% | 0.68% | 0.32% | mature\_B\_cell\_down | Lck |
| GO:0009890 | negative regulation of biosynthetic process | 3.81e-01 | 4.31e-01 | 4 | 49 | 964 | 15137 | 8.16% | 6.37% | 0.41% | 0.32% | mature\_B\_cell\_down | Gata3, Lpin1, Notch1, Sla2 |
| GO:0001944 | vasculature development | 3.82e-01 | 4.33e-01 | 2 | 49 | 408 | 15137 | 4.08% | 2.70% | 0.49% | 0.32% | mature\_B\_cell\_down | Notch1, Thy1 |
| GO:0048736 | appendage development | 3.85e-01 | 4.34e-01 | 1 | 49 | 149 | 15137 | 2.04% | 0.98% | 0.67% | 0.32% | mature\_B\_cell\_down | Notch1 |
| GO:0008406 | gonad development | 3.85e-01 | 4.34e-01 | 1 | 49 | 149 | 15137 | 2.04% | 0.98% | 0.67% | 0.32% | mature\_B\_cell\_down | Gata3 |
| GO:0060173 | limb development | 3.85e-01 | 4.34e-01 | 1 | 49 | 149 | 15137 | 2.04% | 0.98% | 0.67% | 0.32% | mature\_B\_cell\_down | Notch1 |
| GO:0030099 | myeloid cell differentiation | 3.85e-01 | 4.34e-01 | 1 | 49 | 149 | 15137 | 2.04% | 0.98% | 0.67% | 0.32% | mature\_B\_cell\_down | Gata3 |
| GO:0010951 | negative regulation of endopeptidase activity | 3.87e-01 | 4.36e-01 | 1 | 49 | 150 | 15137 | 2.04% | 0.99% | 0.67% | 0.32% | mature\_B\_cell\_down | Cd27 |
| GO:0031346 | positive regulation of cell projection organization | 3.87e-01 | 4.36e-01 | 1 | 49 | 150 | 15137 | 2.04% | 0.99% | 0.67% | 0.32% | mature\_B\_cell\_down | Prkcq |
| GO:0045765 | regulation of angiogenesis | 3.89e-01 | 4.37e-01 | 1 | 49 | 151 | 15137 | 2.04% | 1.00% | 0.66% | 0.32% | mature\_B\_cell\_down | Tnfrsf1a |
| GO:0051146 | striated muscle cell differentiation | 3.89e-01 | 4.37e-01 | 1 | 49 | 151 | 15137 | 2.04% | 1.00% | 0.66% | 0.32% | mature\_B\_cell\_down | F2r |
| GO:0042742 | defense response to bacterium | 3.91e-01 | 4.39e-01 | 1 | 49 | 152 | 15137 | 2.04% | 1.00% | 0.66% | 0.32% | mature\_B\_cell\_down | Tnfrsf1a |
| GO:0001822 | kidney development | 3.91e-01 | 4.39e-01 | 1 | 49 | 152 | 15137 | 2.04% | 1.00% | 0.66% | 0.32% | mature\_B\_cell\_down | Gata3 |
| GO:0044282 | small molecule catabolic process | 3.91e-01 | 4.39e-01 | 1 | 49 | 152 | 15137 | 2.04% | 1.00% | 0.66% | 0.32% | mature\_B\_cell\_down | Lpin1 |
| GO:0071495 | cellular response to endogenous stimulus | 3.91e-01 | 4.39e-01 | 2 | 49 | 416 | 15137 | 4.08% | 2.75% | 0.48% | 0.32% | mature\_B\_cell\_down | Gata3, Lpin1 |
| GO:0051348 | negative regulation of transferase activity | 3.93e-01 | 4.40e-01 | 1 | 49 | 153 | 15137 | 2.04% | 1.01% | 0.65% | 0.32% | mature\_B\_cell\_down | Thy1 |
| GO:0030324 | lung development | 3.97e-01 | 4.44e-01 | 1 | 49 | 155 | 15137 | 2.04% | 1.02% | 0.65% | 0.32% | mature\_B\_cell\_down | Notch1 |
| GO:0001503 | ossification | 3.97e-01 | 4.44e-01 | 1 | 49 | 155 | 15137 | 2.04% | 1.02% | 0.65% | 0.32% | mature\_B\_cell\_down | Notch1 |
| GO:0046039 | GTP metabolic process | 3.99e-01 | 4.45e-01 | 1 | 49 | 156 | 15137 | 2.04% | 1.03% | 0.64% | 0.32% | mature\_B\_cell\_down | Rab37 |
| GO:0032956 | regulation of actin cytoskeleton organization | 3.99e-01 | 4.45e-01 | 1 | 49 | 156 | 15137 | 2.04% | 1.03% | 0.64% | 0.32% | mature\_B\_cell\_down | Prkcq |
| GO:0043434 | response to peptide hormone stimulus | 3.99e-01 | 4.45e-01 | 1 | 49 | 156 | 15137 | 2.04% | 1.03% | 0.64% | 0.32% | mature\_B\_cell\_down | Lpin1 |
| GO:0046942 | carboxylic acid transport | 4.01e-01 | 4.47e-01 | 1 | 49 | 157 | 15137 | 2.04% | 1.04% | 0.64% | 0.32% | mature\_B\_cell\_down | Slc9a3r1 |
| GO:0030323 | respiratory tube development | 4.01e-01 | 4.47e-01 | 1 | 49 | 157 | 15137 | 2.04% | 1.04% | 0.64% | 0.32% | mature\_B\_cell\_down | Notch1 |
| GO:0015849 | organic acid transport | 4.04e-01 | 4.51e-01 | 1 | 49 | 159 | 15137 | 2.04% | 1.05% | 0.63% | 0.32% | mature\_B\_cell\_down | Slc9a3r1 |
| GO:0006916 | anti-apoptosis | 4.08e-01 | 4.55e-01 | 1 | 49 | 161 | 15137 | 2.04% | 1.06% | 0.62% | 0.32% | mature\_B\_cell\_down | Cd27 |
| GO:0006913 | nucleocytoplasmic transport | 4.08e-01 | 4.55e-01 | 1 | 49 | 161 | 15137 | 2.04% | 1.06% | 0.62% | 0.32% | mature\_B\_cell\_down | F2r |
| GO:0009653 | anatomical structure morphogenesis | 4.09e-01 | 4.55e-01 | 6 | 49 | 1586 | 15137 | 12.24% | 10.48% | 0.38% | 0.32% | mature\_B\_cell\_down | Fyn, Gata3, Nckap1, Notch1, Tcf7, Thy1 |
| GO:0072001 | renal system development | 4.10e-01 | 4.56e-01 | 1 | 49 | 162 | 15137 | 2.04% | 1.07% | 0.62% | 0.32% | mature\_B\_cell\_down | Gata3 |
| GO:0006725 | cellular aromatic compound metabolic process | 4.14e-01 | 4.60e-01 | 1 | 49 | 164 | 15137 | 2.04% | 1.08% | 0.61% | 0.32% | mature\_B\_cell\_down | Gata3 |
| GO:0050878 | regulation of body fluid levels | 4.14e-01 | 4.60e-01 | 1 | 49 | 164 | 15137 | 2.04% | 1.08% | 0.61% | 0.32% | mature\_B\_cell\_down | F2r |
| GO:0071705 | nitrogen compound transport | 4.16e-01 | 4.61e-01 | 1 | 49 | 165 | 15137 | 2.04% | 1.09% | 0.61% | 0.32% | mature\_B\_cell\_down | Slc9a3r1 |
| GO:0051169 | nuclear transport | 4.16e-01 | 4.61e-01 | 1 | 49 | 165 | 15137 | 2.04% | 1.09% | 0.61% | 0.32% | mature\_B\_cell\_down | F2r |
| GO:0030111 | regulation of Wnt receptor signaling pathway | 4.18e-01 | 4.62e-01 | 1 | 49 | 166 | 15137 | 2.04% | 1.10% | 0.60% | 0.32% | mature\_B\_cell\_down | Notch1 |
| GO:0032970 | regulation of actin filament-based process | 4.18e-01 | 4.62e-01 | 1 | 49 | 166 | 15137 | 2.04% | 1.10% | 0.60% | 0.32% | mature\_B\_cell\_down | Prkcq |
| GO:0030278 | regulation of ossification | 4.18e-01 | 4.62e-01 | 1 | 49 | 166 | 15137 | 2.04% | 1.10% | 0.60% | 0.32% | mature\_B\_cell\_down | Notch1 |
| GO:0045137 | development of primary sexual characteristics | 4.26e-01 | 4.70e-01 | 1 | 49 | 170 | 15137 | 2.04% | 1.12% | 0.59% | 0.32% | mature\_B\_cell\_down | Gata3 |
| GO:0009628 | response to abiotic stimulus | 4.26e-01 | 4.70e-01 | 2 | 49 | 446 | 15137 | 4.08% | 2.95% | 0.45% | 0.32% | mature\_B\_cell\_down | Fyn, Lck |
| GO:0001763 | morphogenesis of a branching structure | 4.29e-01 | 4.74e-01 | 1 | 49 | 172 | 15137 | 2.04% | 1.14% | 0.58% | 0.32% | mature\_B\_cell\_down | Notch1 |
| GO:0016042 | lipid catabolic process | 4.31e-01 | 4.75e-01 | 1 | 49 | 173 | 15137 | 2.04% | 1.14% | 0.58% | 0.32% | mature\_B\_cell\_down | Lpin1 |
| GO:0051098 | regulation of binding | 4.31e-01 | 4.75e-01 | 1 | 49 | 173 | 15137 | 2.04% | 1.14% | 0.58% | 0.32% | mature\_B\_cell\_down | Gata3 |
| GO:0042493 | response to drug | 4.33e-01 | 4.76e-01 | 1 | 49 | 174 | 15137 | 2.04% | 1.15% | 0.57% | 0.32% | mature\_B\_cell\_down | Lck |
| GO:0042060 | wound healing | 4.33e-01 | 4.76e-01 | 1 | 49 | 174 | 15137 | 2.04% | 1.15% | 0.57% | 0.32% | mature\_B\_cell\_down | F2r |
| GO:0009888 | tissue development | 4.34e-01 | 4.77e-01 | 4 | 49 | 1035 | 15137 | 8.16% | 6.84% | 0.39% | 0.32% | mature\_B\_cell\_down | F2r, Gata3, Nckap1, Notch1 |
| GO:0070482 | response to oxygen levels | 4.39e-01 | 4.82e-01 | 1 | 49 | 177 | 15137 | 2.04% | 1.17% | 0.56% | 0.32% | mature\_B\_cell\_down | Notch1 |
| GO:0045786 | negative regulation of cell cycle | 4.40e-01 | 4.84e-01 | 1 | 49 | 178 | 15137 | 2.04% | 1.18% | 0.56% | 0.32% | mature\_B\_cell\_down | Gata3 |
| GO:0060541 | respiratory system development | 4.42e-01 | 4.85e-01 | 1 | 49 | 179 | 15137 | 2.04% | 1.18% | 0.56% | 0.32% | mature\_B\_cell\_down | Notch1 |
| GO:0002683 | negative regulation of immune system process | 4.44e-01 | 4.87e-01 | 1 | 49 | 180 | 15137 | 2.04% | 1.19% | 0.56% | 0.32% | mature\_B\_cell\_down | Thy1 |
| GO:0032496 | response to lipopolysaccharide | 4.44e-01 | 4.87e-01 | 1 | 49 | 180 | 15137 | 2.04% | 1.19% | 0.56% | 0.32% | mature\_B\_cell\_down | F2r |
| GO:0007005 | mitochondrion organization | 4.48e-01 | 4.90e-01 | 1 | 49 | 182 | 15137 | 2.04% | 1.20% | 0.55% | 0.32% | mature\_B\_cell\_down | Lpin1 |
| GO:0007267 | cell-cell signaling | 4.48e-01 | 4.90e-01 | 2 | 49 | 466 | 15137 | 4.08% | 3.08% | 0.43% | 0.32% | mature\_B\_cell\_down | Gata3, Zdhhc15 |
| GO:0006508 | proteolysis | 4.50e-01 | 4.92e-01 | 3 | 49 | 761 | 15137 | 6.12% | 5.03% | 0.39% | 0.32% | mature\_B\_cell\_down | Ctsd, Ctsw, Prkcq |
| GO:0001933 | negative regulation of protein phosphorylation | 4.57e-01 | 4.99e-01 | 1 | 49 | 187 | 15137 | 2.04% | 1.24% | 0.53% | 0.32% | mature\_B\_cell\_down | Thy1 |
| GO:0006979 | response to oxidative stress | 4.57e-01 | 4.99e-01 | 1 | 49 | 187 | 15137 | 2.04% | 1.24% | 0.53% | 0.32% | mature\_B\_cell\_down | Lck |
| GO:0033554 | cellular response to stress | 4.59e-01 | 5.01e-01 | 3 | 49 | 772 | 15137 | 6.12% | 5.10% | 0.39% | 0.32% | mature\_B\_cell\_down | Ctsd, Notch1, Tnik |
| GO:0044238 | primary metabolic process | 4.60e-01 | 5.02e-01 | 22 | 49 | 6542 | 15137 | 44.90% | 43.22% | 0.34% | 0.32% | mature\_B\_cell\_down | Art2b, Ctsd, Ctsw, F2r, Fyn, Gata3, Hnrpll, Ifih1, Itk, Lck, Lpin1, Notch1, Prkch, Prkcq, Rab37, Stk39, Tcf7, Tnfrsf1a, Tnik, Txk, Zap70, Zdhhc15 |
| GO:0010817 | regulation of hormone levels | 4.62e-01 | 5.03e-01 | 1 | 49 | 190 | 15137 | 2.04% | 1.26% | 0.53% | 0.32% | mature\_B\_cell\_down | Gata3 |
| GO:0016311 | dephosphorylation | 4.66e-01 | 5.07e-01 | 1 | 49 | 192 | 15137 | 2.04% | 1.27% | 0.52% | 0.32% | mature\_B\_cell\_down | Lpin1 |
| GO:0045087 | innate immune response | 4.67e-01 | 5.08e-01 | 1 | 49 | 193 | 15137 | 2.04% | 1.28% | 0.52% | 0.32% | mature\_B\_cell\_down | Ifih1 |
| GO:0031667 | response to nutrient levels | 4.69e-01 | 5.10e-01 | 1 | 49 | 194 | 15137 | 2.04% | 1.28% | 0.52% | 0.32% | mature\_B\_cell\_down | Ctsd |
| GO:0008544 | epidermis development | 4.71e-01 | 5.11e-01 | 1 | 49 | 195 | 15137 | 2.04% | 1.29% | 0.51% | 0.32% | mature\_B\_cell\_down | Notch1 |
| GO:0002697 | regulation of immune effector process | 4.71e-01 | 5.11e-01 | 1 | 49 | 195 | 15137 | 2.04% | 1.29% | 0.51% | 0.32% | mature\_B\_cell\_down | Cd226 |
| GO:0042326 | negative regulation of phosphorylation | 4.78e-01 | 5.18e-01 | 1 | 49 | 199 | 15137 | 2.04% | 1.31% | 0.50% | 0.32% | mature\_B\_cell\_down | Thy1 |
| GO:0046486 | glycerolipid metabolic process | 4.81e-01 | 5.21e-01 | 1 | 49 | 201 | 15137 | 2.04% | 1.33% | 0.50% | 0.32% | mature\_B\_cell\_down | Lpin1 |
| GO:0032501 | multicellular organismal process | 4.88e-01 | 5.29e-01 | 18 | 49 | 5391 | 15137 | 36.73% | 35.61% | 0.33% | 0.32% | mature\_B\_cell\_down | Cd226, Cd7, F2r, Fyn, Gata3, Itk, Lck, Lcp2, Nckap1, Notch1, Prkch, Slc9a3r1, Tcf7, Thy1, Tnik, Txk, Zap70, Zdhhc15 |
| GO:0045936 | negative regulation of phosphate metabolic process | 4.90e-01 | 5.29e-01 | 1 | 49 | 206 | 15137 | 2.04% | 1.36% | 0.49% | 0.32% | mature\_B\_cell\_down | Thy1 |
| GO:0010563 | negative regulation of phosphorus metabolic process | 4.90e-01 | 5.29e-01 | 1 | 49 | 206 | 15137 | 2.04% | 1.36% | 0.49% | 0.32% | mature\_B\_cell\_down | Thy1 |
| GO:0006605 | protein targeting | 4.90e-01 | 5.29e-01 | 1 | 49 | 206 | 15137 | 2.04% | 1.36% | 0.49% | 0.32% | mature\_B\_cell\_down | F2r |
| GO:0042692 | muscle cell differentiation | 4.91e-01 | 5.31e-01 | 1 | 49 | 207 | 15137 | 2.04% | 1.37% | 0.48% | 0.32% | mature\_B\_cell\_down | F2r |
| GO:0010466 | negative regulation of peptidase activity | 5.06e-01 | 5.46e-01 | 1 | 49 | 216 | 15137 | 2.04% | 1.43% | 0.46% | 0.32% | mature\_B\_cell\_down | Cd27 |
| GO:0009991 | response to extracellular stimulus | 5.08e-01 | 5.48e-01 | 1 | 49 | 217 | 15137 | 2.04% | 1.43% | 0.46% | 0.32% | mature\_B\_cell\_down | Ctsd |
| GO:0007517 | muscle organ development | 5.09e-01 | 5.48e-01 | 1 | 49 | 218 | 15137 | 2.04% | 1.44% | 0.46% | 0.32% | mature\_B\_cell\_down | F2r |
| GO:0014706 | striated muscle tissue development | 5.09e-01 | 5.48e-01 | 1 | 49 | 218 | 15137 | 2.04% | 1.44% | 0.46% | 0.32% | mature\_B\_cell\_down | F2r |
| GO:0006066 | alcohol metabolic process | 5.13e-01 | 5.51e-01 | 1 | 49 | 220 | 15137 | 2.04% | 1.45% | 0.45% | 0.32% | mature\_B\_cell\_down | Gata3 |
| GO:0008015 | blood circulation | 5.13e-01 | 5.51e-01 | 1 | 49 | 220 | 15137 | 2.04% | 1.45% | 0.45% | 0.32% | mature\_B\_cell\_down | F2r |
| GO:0022414 | reproductive process | 5.16e-01 | 5.55e-01 | 3 | 49 | 840 | 15137 | 6.12% | 5.55% | 0.36% | 0.32% | mature\_B\_cell\_down | Gata3, Notch1, Tcf7 |
| GO:0003013 | circulatory system process | 5.17e-01 | 5.56e-01 | 1 | 49 | 223 | 15137 | 2.04% | 1.47% | 0.45% | 0.32% | mature\_B\_cell\_down | F2r |
| GO:0000003 | reproduction | 5.19e-01 | 5.56e-01 | 3 | 49 | 843 | 15137 | 6.12% | 5.57% | 0.36% | 0.32% | mature\_B\_cell\_down | Gata3, Notch1, Tcf7 |
| GO:0060627 | regulation of vesicle-mediated transport | 5.19e-01 | 5.56e-01 | 1 | 49 | 224 | 15137 | 2.04% | 1.48% | 0.45% | 0.32% | mature\_B\_cell\_down | Notch1 |
| GO:0042391 | regulation of membrane potential | 5.20e-01 | 5.58e-01 | 1 | 49 | 225 | 15137 | 2.04% | 1.49% | 0.44% | 0.32% | mature\_B\_cell\_down | Fyn |
| GO:0044237 | cellular metabolic process | 5.22e-01 | 5.59e-01 | 21 | 49 | 6399 | 15137 | 42.86% | 42.27% | 0.33% | 0.32% | mature\_B\_cell\_down | Art2b, Ctsd, F2r, Fyn, Gata3, Hnrpll, Ifih1, Itk, Lck, Lpin1, Notch1, Prkch, Prkcq, Rab37, Stk39, Tcf7, Tnfrsf1a, Tnik, Txk, Zap70, Zdhhc15 |
| GO:0032870 | cellular response to hormone stimulus | 5.33e-01 | 5.70e-01 | 1 | 49 | 233 | 15137 | 2.04% | 1.54% | 0.43% | 0.32% | mature\_B\_cell\_down | Lpin1 |
| GO:0007346 | regulation of mitotic cell cycle | 5.33e-01 | 5.70e-01 | 1 | 49 | 233 | 15137 | 2.04% | 1.54% | 0.43% | 0.32% | mature\_B\_cell\_down | Prkcq |
| GO:0006461 | protein complex assembly | 5.35e-01 | 5.72e-01 | 2 | 49 | 549 | 15137 | 4.08% | 3.63% | 0.36% | 0.32% | mature\_B\_cell\_down | Sept9, Tnfrsf1a |
| GO:0060537 | muscle tissue development | 5.36e-01 | 5.72e-01 | 1 | 49 | 235 | 15137 | 2.04% | 1.55% | 0.43% | 0.32% | mature\_B\_cell\_down | F2r |
| GO:0070271 | protein complex biogenesis | 5.37e-01 | 5.73e-01 | 2 | 49 | 551 | 15137 | 4.08% | 3.64% | 0.36% | 0.32% | mature\_B\_cell\_down | Sept9, Tnfrsf1a |
| GO:0010035 | response to inorganic substance | 5.38e-01 | 5.73e-01 | 1 | 49 | 236 | 15137 | 2.04% | 1.56% | 0.42% | 0.32% | mature\_B\_cell\_down | Lck |
| GO:0031400 | negative regulation of protein modification process | 5.41e-01 | 5.75e-01 | 1 | 49 | 238 | 15137 | 2.04% | 1.57% | 0.42% | 0.32% | mature\_B\_cell\_down | Thy1 |
| GO:0033365 | protein localization to organelle | 5.41e-01 | 5.75e-01 | 1 | 49 | 238 | 15137 | 2.04% | 1.57% | 0.42% | 0.32% | mature\_B\_cell\_down | F2r |
| GO:0007169 | transmembrane receptor protein tyrosine kinase signaling pathway | 5.41e-01 | 5.75e-01 | 1 | 49 | 238 | 15137 | 2.04% | 1.57% | 0.42% | 0.32% | mature\_B\_cell\_down | Lat |
| GO:0051726 | regulation of cell cycle | 5.47e-01 | 5.81e-01 | 2 | 49 | 561 | 15137 | 4.08% | 3.71% | 0.36% | 0.32% | mature\_B\_cell\_down | Gata3, Prkcq |
| GO:0080135 | regulation of cellular response to stress | 5.48e-01 | 5.82e-01 | 1 | 49 | 243 | 15137 | 2.04% | 1.61% | 0.41% | 0.32% | mature\_B\_cell\_down | Cd27 |
| GO:0051493 | regulation of cytoskeleton organization | 5.48e-01 | 5.82e-01 | 1 | 49 | 243 | 15137 | 2.04% | 1.61% | 0.41% | 0.32% | mature\_B\_cell\_down | Prkcq |
| GO:0031644 | regulation of neurological system process | 5.51e-01 | 5.85e-01 | 1 | 49 | 245 | 15137 | 2.04% | 1.62% | 0.41% | 0.32% | mature\_B\_cell\_down | F2r |
| GO:0071822 | protein complex subunit organization | 5.53e-01 | 5.86e-01 | 2 | 49 | 567 | 15137 | 4.08% | 3.75% | 0.35% | 0.32% | mature\_B\_cell\_down | Sept9, Tnfrsf1a |
| GO:0009207 | purine ribonucleoside triphosphate catabolic process | 5.74e-01 | 6.08e-01 | 1 | 49 | 261 | 15137 | 2.04% | 1.72% | 0.38% | 0.32% | mature\_B\_cell\_down | Rab37 |
| GO:0048285 | organelle fission | 5.76e-01 | 6.09e-01 | 1 | 49 | 262 | 15137 | 2.04% | 1.73% | 0.38% | 0.32% | mature\_B\_cell\_down | Lpin1 |
| GO:0009203 | ribonucleoside triphosphate catabolic process | 5.76e-01 | 6.09e-01 | 1 | 49 | 262 | 15137 | 2.04% | 1.73% | 0.38% | 0.32% | mature\_B\_cell\_down | Rab37 |
| GO:0009146 | purine nucleoside triphosphate catabolic process | 5.80e-01 | 6.13e-01 | 1 | 49 | 265 | 15137 | 2.04% | 1.75% | 0.38% | 0.32% | mature\_B\_cell\_down | Rab37 |
| GO:0009154 | purine ribonucleotide catabolic process | 5.81e-01 | 6.14e-01 | 1 | 49 | 266 | 15137 | 2.04% | 1.76% | 0.38% | 0.32% | mature\_B\_cell\_down | Rab37 |
| GO:0044283 | small molecule biosynthetic process | 5.84e-01 | 6.16e-01 | 1 | 49 | 268 | 15137 | 2.04% | 1.77% | 0.37% | 0.32% | mature\_B\_cell\_down | Gata3 |
| GO:0009143 | nucleoside triphosphate catabolic process | 5.87e-01 | 6.18e-01 | 1 | 49 | 270 | 15137 | 2.04% | 1.78% | 0.37% | 0.32% | mature\_B\_cell\_down | Rab37 |
| GO:0009261 | ribonucleotide catabolic process | 5.87e-01 | 6.18e-01 | 1 | 49 | 270 | 15137 | 2.04% | 1.78% | 0.37% | 0.32% | mature\_B\_cell\_down | Rab37 |
| GO:0009719 | response to endogenous stimulus | 5.94e-01 | 6.26e-01 | 2 | 49 | 611 | 15137 | 4.08% | 4.04% | 0.33% | 0.32% | mature\_B\_cell\_down | Gata3, Lpin1 |
| GO:0044255 | cellular lipid metabolic process | 5.99e-01 | 6.31e-01 | 2 | 49 | 617 | 15137 | 4.08% | 4.08% | 0.32% | 0.32% | mature\_B\_cell\_down | Lpin1, Tnfrsf1a |
| GO:0008152 | metabolic process | 6.03e-01 | 6.35e-01 | 24 | 49 | 7543 | 15137 | 48.98% | 49.83% | 0.32% | 0.32% | mature\_B\_cell\_down | Art2b, Ctsd, Ctsw, Enpp4, F2r, Fyn, Gata3, Hnrpll, Ifih1, Itk, Lat, Lck, Lpin1, Notch1, Prkch, Prkcq, Rab37, Stk39, Tcf7, Tnfrsf1a, Tnik, Txk, Zap70, Zdhhc15 |
| GO:0051129 | negative regulation of cellular component organization | 6.04e-01 | 6.35e-01 | 1 | 49 | 283 | 15137 | 2.04% | 1.87% | 0.35% | 0.32% | mature\_B\_cell\_down | Thy1 |
| GO:0006195 | purine nucleotide catabolic process | 6.05e-01 | 6.36e-01 | 1 | 49 | 284 | 15137 | 2.04% | 1.88% | 0.35% | 0.32% | mature\_B\_cell\_down | Rab37 |
| GO:0072523 | purine-containing compound catabolic process | 6.13e-01 | 6.44e-01 | 1 | 49 | 290 | 15137 | 2.04% | 1.92% | 0.34% | 0.32% | mature\_B\_cell\_down | Rab37 |
| GO:0019752 | carboxylic acid metabolic process | 6.17e-01 | 6.47e-01 | 2 | 49 | 637 | 15137 | 4.08% | 4.21% | 0.31% | 0.32% | mature\_B\_cell\_down | Lpin1, Tnfrsf1a |
| GO:0043436 | oxoacid metabolic process | 6.17e-01 | 6.47e-01 | 2 | 49 | 637 | 15137 | 4.08% | 4.21% | 0.31% | 0.32% | mature\_B\_cell\_down | Lpin1, Tnfrsf1a |
| GO:0006397 | mRNA processing | 6.21e-01 | 6.50e-01 | 1 | 49 | 296 | 15137 | 2.04% | 1.96% | 0.34% | 0.32% | mature\_B\_cell\_down | Hnrpll |
| GO:0032446 | protein modification by small protein conjugation | 6.22e-01 | 6.51e-01 | 1 | 49 | 297 | 15137 | 2.04% | 1.96% | 0.34% | 0.32% | mature\_B\_cell\_down | Ifih1 |
| GO:0007268 | synaptic transmission | 6.24e-01 | 6.53e-01 | 1 | 49 | 299 | 15137 | 2.04% | 1.98% | 0.33% | 0.32% | mature\_B\_cell\_down | Zdhhc15 |
| GO:0006935 | chemotaxis | 6.28e-01 | 6.57e-01 | 1 | 49 | 302 | 15137 | 2.04% | 2.00% | 0.33% | 0.32% | mature\_B\_cell\_down | Gata3 |
| GO:0006082 | organic acid metabolic process | 6.29e-01 | 6.57e-01 | 2 | 49 | 651 | 15137 | 4.08% | 4.30% | 0.31% | 0.32% | mature\_B\_cell\_down | Lpin1, Tnfrsf1a |
| GO:0010564 | regulation of cell cycle process | 6.29e-01 | 6.57e-01 | 1 | 49 | 303 | 15137 | 2.04% | 2.00% | 0.33% | 0.32% | mature\_B\_cell\_down | Prkcq |
| GO:0042330 | taxis | 6.29e-01 | 6.57e-01 | 1 | 49 | 303 | 15137 | 2.04% | 2.00% | 0.33% | 0.32% | mature\_B\_cell\_down | Gata3 |
| GO:0009166 | nucleotide catabolic process | 6.31e-01 | 6.57e-01 | 1 | 49 | 304 | 15137 | 2.04% | 2.01% | 0.33% | 0.32% | mature\_B\_cell\_down | Rab37 |
| GO:0009205 | purine ribonucleoside triphosphate metabolic process | 6.31e-01 | 6.57e-01 | 1 | 49 | 304 | 15137 | 2.04% | 2.01% | 0.33% | 0.32% | mature\_B\_cell\_down | Rab37 |
| GO:0042180 | cellular ketone metabolic process | 6.31e-01 | 6.58e-01 | 2 | 49 | 654 | 15137 | 4.08% | 4.32% | 0.31% | 0.32% | mature\_B\_cell\_down | Lpin1, Tnfrsf1a |
| GO:0009199 | ribonucleoside triphosphate metabolic process | 6.34e-01 | 6.60e-01 | 1 | 49 | 307 | 15137 | 2.04% | 2.03% | 0.33% | 0.32% | mature\_B\_cell\_down | Rab37 |
| GO:0048232 | male gamete generation | 6.37e-01 | 6.62e-01 | 1 | 49 | 309 | 15137 | 2.04% | 2.04% | 0.32% | 0.32% | mature\_B\_cell\_down | Notch1 |
| GO:0007283 | spermatogenesis | 6.37e-01 | 6.62e-01 | 1 | 49 | 309 | 15137 | 2.04% | 2.04% | 0.32% | 0.32% | mature\_B\_cell\_down | Notch1 |
| GO:0009144 | purine nucleoside triphosphate metabolic process | 6.39e-01 | 6.64e-01 | 1 | 49 | 311 | 15137 | 2.04% | 2.05% | 0.32% | 0.32% | mature\_B\_cell\_down | Rab37 |
| GO:0034655 | nucleobase-containing compound catabolic process | 6.51e-01 | 6.75e-01 | 1 | 49 | 321 | 15137 | 2.04% | 2.12% | 0.31% | 0.32% | mature\_B\_cell\_down | Rab37 |
| GO:0009141 | nucleoside triphosphate metabolic process | 6.51e-01 | 6.75e-01 | 1 | 49 | 321 | 15137 | 2.04% | 2.12% | 0.31% | 0.32% | mature\_B\_cell\_down | Rab37 |
| GO:0061061 | muscle structure development | 6.53e-01 | 6.77e-01 | 1 | 49 | 323 | 15137 | 2.04% | 2.13% | 0.31% | 0.32% | mature\_B\_cell\_down | F2r |
| GO:0006351 | transcription, DNA-dependent | 6.54e-01 | 6.77e-01 | 5 | 49 | 1694 | 15137 | 10.20% | 11.19% | 0.30% | 0.32% | mature\_B\_cell\_down | Gata3, Lpin1, Notch1, Tcf7, Txk |
| GO:0032774 | RNA biosynthetic process | 6.55e-01 | 6.77e-01 | 5 | 49 | 1696 | 15137 | 10.20% | 11.20% | 0.29% | 0.32% | mature\_B\_cell\_down | Gata3, Lpin1, Notch1, Tcf7, Txk |
| GO:0032269 | negative regulation of cellular protein metabolic process | 6.55e-01 | 6.77e-01 | 1 | 49 | 325 | 15137 | 2.04% | 2.15% | 0.31% | 0.32% | mature\_B\_cell\_down | Thy1 |
| GO:0010608 | posttranscriptional regulation of gene expression | 6.55e-01 | 6.77e-01 | 1 | 49 | 325 | 15137 | 2.04% | 2.15% | 0.31% | 0.32% | mature\_B\_cell\_down | Nckap1 |
| GO:0009150 | purine ribonucleotide metabolic process | 6.59e-01 | 6.80e-01 | 1 | 49 | 328 | 15137 | 2.04% | 2.17% | 0.30% | 0.32% | mature\_B\_cell\_down | Rab37 |
| GO:0044271 | cellular nitrogen compound biosynthetic process | 6.62e-01 | 6.83e-01 | 1 | 49 | 331 | 15137 | 2.04% | 2.19% | 0.30% | 0.32% | mature\_B\_cell\_down | Gata3 |
| GO:0044248 | cellular catabolic process | 6.62e-01 | 6.83e-01 | 3 | 49 | 1038 | 15137 | 6.12% | 6.86% | 0.29% | 0.32% | mature\_B\_cell\_down | Ctsd, Lpin1, Rab37 |
| GO:0065003 | macromolecular complex assembly | 6.62e-01 | 6.83e-01 | 2 | 49 | 692 | 15137 | 4.08% | 4.57% | 0.29% | 0.32% | mature\_B\_cell\_down | Sept9, Tnfrsf1a |
| GO:0046700 | heterocycle catabolic process | 6.67e-01 | 6.87e-01 | 1 | 49 | 335 | 15137 | 2.04% | 2.21% | 0.30% | 0.32% | mature\_B\_cell\_down | Rab37 |
| GO:0048610 | cellular process involved in reproduction | 6.68e-01 | 6.87e-01 | 1 | 49 | 336 | 15137 | 2.04% | 2.22% | 0.30% | 0.32% | mature\_B\_cell\_down | Notch1 |
| GO:0044270 | cellular nitrogen compound catabolic process | 6.70e-01 | 6.89e-01 | 1 | 49 | 338 | 15137 | 2.04% | 2.23% | 0.30% | 0.32% | mature\_B\_cell\_down | Rab37 |
| GO:0009259 | ribonucleotide metabolic process | 6.72e-01 | 6.91e-01 | 1 | 49 | 340 | 15137 | 2.04% | 2.25% | 0.29% | 0.32% | mature\_B\_cell\_down | Rab37 |
| GO:0016071 | mRNA metabolic process | 6.76e-01 | 6.95e-01 | 1 | 49 | 344 | 15137 | 2.04% | 2.27% | 0.29% | 0.32% | mature\_B\_cell\_down | Hnrpll |
| GO:0044281 | small molecule metabolic process | 6.81e-01 | 7.00e-01 | 4 | 49 | 1410 | 15137 | 8.16% | 9.31% | 0.28% | 0.32% | mature\_B\_cell\_down | Gata3, Lpin1, Rab37, Tnfrsf1a |
| GO:0071702 | organic substance transport | 6.95e-01 | 7.13e-01 | 1 | 49 | 362 | 15137 | 2.04% | 2.39% | 0.28% | 0.32% | mature\_B\_cell\_down | Slc9a3r1 |
| GO:0070647 | protein modification by small protein conjugation or removal | 7.00e-01 | 7.18e-01 | 1 | 49 | 367 | 15137 | 2.04% | 2.42% | 0.27% | 0.32% | mature\_B\_cell\_down | Ifih1 |
| GO:0043933 | macromolecular complex subunit organization | 7.05e-01 | 7.22e-01 | 2 | 49 | 749 | 15137 | 4.08% | 4.95% | 0.27% | 0.32% | mature\_B\_cell\_down | Sept9, Tnfrsf1a |
| GO:0044106 | cellular amine metabolic process | 7.07e-01 | 7.24e-01 | 1 | 49 | 374 | 15137 | 2.04% | 2.47% | 0.27% | 0.32% | mature\_B\_cell\_down | Gata3 |
| GO:0007167 | enzyme linked receptor protein signaling pathway | 7.13e-01 | 7.29e-01 | 1 | 49 | 380 | 15137 | 2.04% | 2.51% | 0.26% | 0.32% | mature\_B\_cell\_down | Lat |
| GO:0010467 | gene expression | 7.15e-01 | 7.31e-01 | 7 | 49 | 2486 | 15137 | 14.29% | 16.42% | 0.28% | 0.32% | mature\_B\_cell\_down | Gata3, Hnrpll, Lat, Lpin1, Notch1, Tcf7, Txk |
| GO:0006163 | purine nucleotide metabolic process | 7.22e-01 | 7.38e-01 | 1 | 49 | 390 | 15137 | 2.04% | 2.58% | 0.26% | 0.32% | mature\_B\_cell\_down | Rab37 |
| GO:0007276 | gamete generation | 7.30e-01 | 7.46e-01 | 1 | 49 | 399 | 15137 | 2.04% | 2.64% | 0.25% | 0.32% | mature\_B\_cell\_down | Notch1 |
| GO:0009725 | response to hormone stimulus | 7.32e-01 | 7.47e-01 | 1 | 49 | 401 | 15137 | 2.04% | 2.65% | 0.25% | 0.32% | mature\_B\_cell\_down | Lpin1 |
| GO:0006629 | lipid metabolic process | 7.56e-01 | 7.71e-01 | 2 | 49 | 827 | 15137 | 4.08% | 5.46% | 0.24% | 0.32% | mature\_B\_cell\_down | Lpin1, Tnfrsf1a |
| GO:0016070 | RNA metabolic process | 7.58e-01 | 7.72e-01 | 6 | 49 | 2255 | 15137 | 12.24% | 14.90% | 0.27% | 0.32% | mature\_B\_cell\_down | Gata3, Hnrpll, Lpin1, Notch1, Tcf7, Txk |
| GO:0072521 | purine-containing compound metabolic process | 7.59e-01 | 7.72e-01 | 1 | 49 | 432 | 15137 | 2.04% | 2.85% | 0.23% | 0.32% | mature\_B\_cell\_down | Rab37 |
| GO:0009987 | cellular process | 7.59e-01 | 7.73e-01 | 39 | 49 | 12456 | 15137 | 79.59% | 82.29% | 0.31% | 0.32% | mature\_B\_cell\_down | Apol7b, Art2b, Cd226, Cd247, Cd27, Cd3g, Cd96, Ctsd, F2r, Fyn, Gata3, Hnrpll, Ifih1, Itk, Lat, Lck, Lcp2, Lpin1, Nckap1, Notch1, Prkch, Prkcq, Rab37, Selplg, Sept9, Skap1, Sla2, Slc12a7, Slc9a3r1, Stk39, Sytl2, Tcf7, Thy1, Tnfrsf1a, Tnik, Txk, Vps26b, Zap70, Zdhhc15 |
| GO:0019953 | sexual reproduction | 7.77e-01 | 7.90e-01 | 1 | 49 | 456 | 15137 | 2.04% | 3.01% | 0.22% | 0.32% | mature\_B\_cell\_down | Notch1 |
| GO:0034645 | cellular macromolecule biosynthetic process | 7.83e-01 | 7.95e-01 | 6 | 49 | 2320 | 15137 | 12.24% | 15.33% | 0.26% | 0.32% | mature\_B\_cell\_down | Gata3, Lpin1, Notch1, Tcf7, Txk, Zdhhc15 |
| GO:0009308 | amine metabolic process | 7.84e-01 | 7.97e-01 | 1 | 49 | 466 | 15137 | 2.04% | 3.08% | 0.21% | 0.32% | mature\_B\_cell\_down | Gata3 |
| GO:0009056 | catabolic process | 7.88e-01 | 8.00e-01 | 3 | 49 | 1264 | 15137 | 6.12% | 8.35% | 0.24% | 0.32% | mature\_B\_cell\_down | Ctsd, Lpin1, Rab37 |
| GO:0009059 | macromolecule biosynthetic process | 7.92e-01 | 8.03e-01 | 6 | 49 | 2346 | 15137 | 12.24% | 15.50% | 0.26% | 0.32% | mature\_B\_cell\_down | Gata3, Lpin1, Notch1, Tcf7, Txk, Zdhhc15 |
| GO:0006396 | RNA processing | 8.09e-01 | 8.19e-01 | 1 | 49 | 502 | 15137 | 2.04% | 3.32% | 0.20% | 0.32% | mature\_B\_cell\_down | Hnrpll |
| GO:0006753 | nucleoside phosphate metabolic process | 8.09e-01 | 8.19e-01 | 1 | 49 | 502 | 15137 | 2.04% | 3.32% | 0.20% | 0.32% | mature\_B\_cell\_down | Rab37 |
| GO:0009117 | nucleotide metabolic process | 8.09e-01 | 8.19e-01 | 1 | 49 | 502 | 15137 | 2.04% | 3.32% | 0.20% | 0.32% | mature\_B\_cell\_down | Rab37 |
| GO:0032504 | multicellular organism reproduction | 8.16e-01 | 8.25e-01 | 1 | 49 | 513 | 15137 | 2.04% | 3.39% | 0.19% | 0.32% | mature\_B\_cell\_down | Notch1 |
| GO:0048609 | multicellular organismal reproductive process | 8.16e-01 | 8.25e-01 | 1 | 49 | 513 | 15137 | 2.04% | 3.39% | 0.19% | 0.32% | mature\_B\_cell\_down | Notch1 |
| GO:0055086 | nucleobase-containing small molecule metabolic process | 8.35e-01 | 8.44e-01 | 1 | 49 | 546 | 15137 | 2.04% | 3.61% | 0.18% | 0.32% | mature\_B\_cell\_down | Rab37 |
| GO:0006259 | DNA metabolic process | 8.62e-01 | 8.70e-01 | 1 | 49 | 598 | 15137 | 2.04% | 3.95% | 0.17% | 0.32% | mature\_B\_cell\_down | Tcf7 |
| GO:0003008 | system process | 8.79e-01 | 8.86e-01 | 5 | 49 | 2275 | 15137 | 10.20% | 15.03% | 0.22% | 0.32% | mature\_B\_cell\_down | F2r, Fyn, Gata3, Slc9a3r1, Zdhhc15 |
| GO:0046483 | heterocycle metabolic process | 8.81e-01 | 8.88e-01 | 1 | 49 | 642 | 15137 | 2.04% | 4.24% | 0.16% | 0.32% | mature\_B\_cell\_down | Rab37 |
| GO:0090304 | nucleic acid metabolic process | 8.90e-01 | 8.96e-01 | 6 | 49 | 2692 | 15137 | 12.24% | 17.78% | 0.22% | 0.32% | mature\_B\_cell\_down | Gata3, Hnrpll, Lpin1, Notch1, Tcf7, Txk |
| GO:0055085 | transmembrane transport | 8.90e-01 | 8.96e-01 | 1 | 49 | 667 | 15137 | 2.04% | 4.41% | 0.15% | 0.32% | mature\_B\_cell\_down | Slc12a7 |
| GO:0006139 | nucleobase-containing compound metabolic process | 9.21e-01 | 9.26e-01 | 7 | 49 | 3219 | 15137 | 14.29% | 21.27% | 0.22% | 0.32% | mature\_B\_cell\_down | Gata3, Hnrpll, Lpin1, Notch1, Rab37, Tcf7, Txk |
| GO:0007049 | cell cycle | 9.37e-01 | 9.42e-01 | 1 | 49 | 830 | 15137 | 2.04% | 5.48% | 0.12% | 0.32% | mature\_B\_cell\_down | Sept9 |
| GO:0044249 | cellular biosynthetic process | 9.40e-01 | 9.44e-01 | 6 | 49 | 2982 | 15137 | 12.24% | 19.70% | 0.20% | 0.32% | mature\_B\_cell\_down | Gata3, Lpin1, Notch1, Tcf7, Txk, Zdhhc15 |
| GO:0009058 | biosynthetic process | 9.52e-01 | 9.56e-01 | 6 | 49 | 3082 | 15137 | 12.24% | 20.36% | 0.19% | 0.32% | mature\_B\_cell\_down | Gata3, Lpin1, Notch1, Tcf7, Txk, Zdhhc15 |
| GO:0034641 | cellular nitrogen compound metabolic process | 9.61e-01 | 9.65e-01 | 7 | 49 | 3557 | 15137 | 14.29% | 23.50% | 0.20% | 0.32% | mature\_B\_cell\_down | Gata3, Hnrpll, Lpin1, Notch1, Rab37, Tcf7, Txk |
| GO:0006807 | nitrogen compound metabolic process | 9.70e-01 | 9.72e-01 | 7 | 49 | 3665 | 15137 | 14.29% | 24.21% | 0.19% | 0.32% | mature\_B\_cell\_down | Gata3, Hnrpll, Lpin1, Notch1, Rab37, Tcf7, Txk |
| GO:0007186 | G-protein coupled receptor signaling pathway | 9.91e-01 | 9.93e-01 | 2 | 49 | 1974 | 15137 | 4.08% | 13.04% | 0.10% | 0.32% | mature\_B\_cell\_down | F2r, Slc9a3r1 |
| GO:0050877 | neurological system process | 9.92e-01 | 9.94e-01 | 2 | 49 | 2011 | 15137 | 4.08% | 13.29% | 0.10% | 0.32% | mature\_B\_cell\_down | Fyn, Zdhhc15 |
| GO:0007600 | sensory perception | 9.95e-01 | 9.95e-01 | 1 | 49 | 1531 | 15137 | 2.04% | 10.11% | 0.07% | 0.32% | mature\_B\_cell\_down | Fyn |
| GO:0008150 | biological\_process | 1.00e+00 | 1.00e+00 | 49 | 49 | 15137 | 15137 | 100.00% | 100.00% | 0.32% | 0.32% | mature\_B\_cell\_down | Apol7b, Art2b, Cd226, Cd247, Cd27, Cd3g, Cd7, Cd96, Ctsd, Ctsw, Enpp4, F2r, Fam125b, Fyn, Gata3, Hnrpll, Ifi47, Ifih1, Ipcef1, Itk, Lat, Lck, Lcp2, Lpin1, Nckap1, Notch1, Prkch, Prkcq, Rab37, Rinl, Selplg, Sept9, Sh2d2a, Skap1, Sla2, Slc12a7, Slc9a3r1, Stk39, Sytl2, Tbc1d2b, Tcf7, Thy1, Tmem66, Tnfrsf1a, Tnik, Txk, Vps26b, Zap70, Zdhhc15 |

### cellular\_component (top)

  
  


| TermID | Term | P | Q | k | n | K | N | k/n | K/N | k/K | n/N | Qset | Symbols |
| --- | --- | --- | --- | --- | --- | --- | --- | --- | --- | --- | --- | --- | --- |
| GO:0042101 | T cell receptor complex | 1.56e-06 | 2.35e-04 | 3 | 51 | 8 | 16439 | 5.88% | 0.05% | 37.50% | 0.31% | mature\_B\_cell\_down | Cd247, Cd3g, Zap70 |
| GO:0001772 | immunological synapse | 2.63e-05 | 1.99e-03 | 3 | 51 | 19 | 16439 | 5.88% | 0.12% | 15.79% | 0.31% | mature\_B\_cell\_down | Lat, Prkcq, Zap70 |
| GO:0005886 | plasma membrane | 7.44e-05 | 3.35e-03 | 24 | 51 | 3649 | 16439 | 47.06% | 22.20% | 0.66% | 0.31% | mature\_B\_cell\_down | Art2b, Cd247, Cd27, Cd3g, F2r, Fyn, Ipcef1, Lat, Lck, Nckap1, Notch1, Prkch, Prkcq, Selplg, Sh2d2a, Skap1, Sla2, Slc9a3r1, Stk39, Sytl2, Thy1, Tnfrsf1a, Txk, Zap70 |
| GO:0042105 | alpha-beta T cell receptor complex | 9.38e-05 | 3.35e-03 | 2 | 51 | 5 | 16439 | 3.92% | 0.03% | 40.00% | 0.31% | mature\_B\_cell\_down | Cd247, Cd3g |
| GO:0071944 | cell periphery | 1.11e-04 | 3.35e-03 | 24 | 51 | 3737 | 16439 | 47.06% | 22.73% | 0.64% | 0.31% | mature\_B\_cell\_down | Art2b, Cd247, Cd27, Cd3g, F2r, Fyn, Ipcef1, Lat, Lck, Nckap1, Notch1, Prkch, Prkcq, Selplg, Sh2d2a, Skap1, Sla2, Slc9a3r1, Stk39, Sytl2, Thy1, Tnfrsf1a, Txk, Zap70 |
| GO:0044459 | plasma membrane part | 8.40e-04 | 2.11e-02 | 13 | 51 | 1582 | 16439 | 25.49% | 9.62% | 0.82% | 0.31% | mature\_B\_cell\_down | Art2b, Cd247, Cd27, Cd3g, F2r, Lat, Notch1, Prkcq, Slc9a3r1, Stk39, Sytl2, Thy1, Zap70 |
| GO:0016020 | membrane | 1.63e-03 | 3.52e-02 | 36 | 51 | 8099 | 16439 | 70.59% | 49.27% | 0.44% | 0.31% | mature\_B\_cell\_down | Art2b, Btbd11, Cd226, Cd247, Cd27, Cd3g, Cd7, Cd96, Enpp4, F2r, Fam125b, Fyn, Ipcef1, Lat, Lck, Leprotl1, Lpin1, Nckap1, Notch1, Prkch, Prkcq, Selplg, Sh2d2a, Skap1, Sla2, Slc12a7, Slc9a3r1, Stk39, Sytl2, Thy1, Tmem66, Tnfrsf1a, Txk, Vps26b, Zap70, Zdhhc15 |
| GO:0045121 | membrane raft | 4.47e-03 | 8.44e-02 | 4 | 51 | 216 | 16439 | 7.84% | 1.31% | 1.85% | 0.31% | mature\_B\_cell\_down | F2r, Lck, Thy1, Tnfrsf1a |
| GO:0031594 | neuromuscular junction | 8.21e-03 | 1.27e-01 | 2 | 51 | 44 | 16439 | 3.92% | 0.27% | 4.55% | 0.31% | mature\_B\_cell\_down | F2r, Prkcq |
| GO:0002193 | MAML1-RBP-Jkappa- ICN1 complex | 9.28e-03 | 1.27e-01 | 1 | 51 | 3 | 16439 | 1.96% | 0.02% | 33.33% | 0.31% | mature\_B\_cell\_down | Notch1 |
| GO:0030904 | retromer complex | 9.28e-03 | 1.27e-01 | 1 | 51 | 3 | 16439 | 1.96% | 0.02% | 33.33% | 0.31% | mature\_B\_cell\_down | Vps26b |
| GO:0043235 | receptor complex | 1.15e-02 | 1.43e-01 | 3 | 51 | 151 | 16439 | 5.88% | 0.92% | 1.99% | 0.31% | mature\_B\_cell\_down | Cd247, Cd3g, Zap70 |
| GO:0031094 | platelet dense tubular network | 1.24e-02 | 1.43e-01 | 1 | 51 | 4 | 16439 | 1.96% | 0.02% | 25.00% | 0.31% | mature\_B\_cell\_down | F2r |
| GO:0070382 | exocytic vesicle | 2.15e-02 | 2.31e-01 | 1 | 51 | 7 | 16439 | 1.96% | 0.04% | 14.29% | 0.31% | mature\_B\_cell\_down | Sytl2 |
| GO:0016023 | cytoplasmic membrane-bounded vesicle | 2.50e-02 | 2.31e-01 | 5 | 51 | 537 | 16439 | 9.80% | 3.27% | 0.93% | 0.31% | mature\_B\_cell\_down | Lck, Notch1, Rab37, Sla2, Sytl2 |
| GO:0019897 | extrinsic to plasma membrane | 2.50e-02 | 2.31e-01 | 2 | 51 | 79 | 16439 | 3.92% | 0.48% | 2.53% | 0.31% | mature\_B\_cell\_down | Art2b, Sytl2 |
| GO:0009986 | cell surface | 2.68e-02 | 2.31e-01 | 5 | 51 | 547 | 16439 | 9.80% | 3.33% | 0.91% | 0.31% | mature\_B\_cell\_down | Cd226, Cd27, Notch1, Thy1, Tnfrsf1a |
| GO:0031528 | microvillus membrane | 2.76e-02 | 2.31e-01 | 1 | 51 | 9 | 16439 | 1.96% | 0.05% | 11.11% | 0.31% | mature\_B\_cell\_down | Slc9a3r1 |
| GO:0031988 | membrane-bounded vesicle | 3.16e-02 | 2.51e-01 | 5 | 51 | 572 | 16439 | 9.80% | 3.48% | 0.87% | 0.31% | mature\_B\_cell\_down | Lck, Notch1, Rab37, Sla2, Sytl2 |
| GO:0031362 | anchored to external side of plasma membrane | 3.36e-02 | 2.54e-01 | 1 | 51 | 11 | 16439 | 1.96% | 0.07% | 9.09% | 0.31% | mature\_B\_cell\_down | Thy1 |
| GO:0044425 | membrane part | 3.85e-02 | 2.72e-01 | 27 | 51 | 6537 | 16439 | 52.94% | 39.77% | 0.41% | 0.31% | mature\_B\_cell\_down | Art2b, Btbd11, Cd226, Cd247, Cd27, Cd3g, Cd7, Cd96, Enpp4, F2r, Lat, Lck, Leprotl1, Nckap1, Notch1, Prkcq, Selplg, Slc12a7, Slc9a3r1, Stk39, Sytl2, Thy1, Tmem66, Tnfrsf1a, Vps26b, Zap70, Zdhhc15 |
| GO:0000242 | pericentriolar material | 3.96e-02 | 2.72e-01 | 1 | 51 | 13 | 16439 | 1.96% | 0.08% | 7.69% | 0.31% | mature\_B\_cell\_down | Lck |
| GO:0031233 | intrinsic to external side of plasma membrane | 4.26e-02 | 2.80e-01 | 1 | 51 | 14 | 16439 | 1.96% | 0.09% | 7.14% | 0.31% | mature\_B\_cell\_down | Thy1 |
| GO:0019898 | extrinsic to membrane | 5.19e-02 | 3.21e-01 | 2 | 51 | 118 | 16439 | 3.92% | 0.72% | 1.69% | 0.31% | mature\_B\_cell\_down | Art2b, Sytl2 |
| GO:0005768 | endosome | 5.68e-02 | 3.21e-01 | 4 | 51 | 468 | 16439 | 7.84% | 2.85% | 0.85% | 0.31% | mature\_B\_cell\_down | Fam125b, Fyn, Sla2, Tnik |
| GO:0042470 | melanosome | 5.74e-02 | 3.21e-01 | 1 | 51 | 19 | 16439 | 1.96% | 0.12% | 5.26% | 0.31% | mature\_B\_cell\_down | Sytl2 |
| GO:0048770 | pigment granule | 5.74e-02 | 3.21e-01 | 1 | 51 | 19 | 16439 | 1.96% | 0.12% | 5.26% | 0.31% | mature\_B\_cell\_down | Sytl2 |
| GO:0031225 | anchored to membrane | 6.58e-02 | 3.55e-01 | 2 | 51 | 135 | 16439 | 3.92% | 0.82% | 1.48% | 0.31% | mature\_B\_cell\_down | Art2b, Thy1 |
| GO:0031410 | cytoplasmic vesicle | 8.37e-02 | 4.36e-01 | 5 | 51 | 755 | 16439 | 9.80% | 4.59% | 0.66% | 0.31% | mature\_B\_cell\_down | Lck, Notch1, Rab37, Sla2, Sytl2 |
| GO:0046658 | anchored to plasma membrane | 8.91e-02 | 4.48e-01 | 1 | 51 | 30 | 16439 | 1.96% | 0.18% | 3.33% | 0.31% | mature\_B\_cell\_down | Thy1 |
| GO:0031982 | vesicle | 1.05e-01 | 5.00e-01 | 5 | 51 | 810 | 16439 | 9.80% | 4.93% | 0.62% | 0.31% | mature\_B\_cell\_down | Lck, Notch1, Rab37, Sla2, Sytl2 |
| GO:0005793 | endoplasmic reticulum-Golgi intermediate compartment | 1.06e-01 | 5.00e-01 | 1 | 51 | 36 | 16439 | 1.96% | 0.22% | 2.78% | 0.31% | mature\_B\_cell\_down | Rab37 |
| GO:0005624 | membrane fraction | 1.10e-01 | 5.05e-01 | 4 | 51 | 591 | 16439 | 7.84% | 3.60% | 0.68% | 0.31% | mature\_B\_cell\_down | Cd27, Fyn, Prkch, Prkcq |
| GO:0031526 | brush border membrane | 1.20e-01 | 5.05e-01 | 1 | 51 | 41 | 16439 | 1.96% | 0.25% | 2.44% | 0.31% | mature\_B\_cell\_down | Slc9a3r1 |
| GO:0000267 | cell fraction | 1.32e-01 | 5.05e-01 | 5 | 51 | 872 | 16439 | 9.80% | 5.30% | 0.57% | 0.31% | mature\_B\_cell\_down | Cd27, Ctsd, Fyn, Prkch, Prkcq |
| GO:0005626 | insoluble fraction | 1.32e-01 | 5.05e-01 | 4 | 51 | 633 | 16439 | 7.84% | 3.85% | 0.63% | 0.31% | mature\_B\_cell\_down | Cd27, Fyn, Prkch, Prkcq |
| GO:0016324 | apical plasma membrane | 1.41e-01 | 5.05e-01 | 2 | 51 | 213 | 16439 | 3.92% | 1.30% | 0.94% | 0.31% | mature\_B\_cell\_down | Slc9a3r1, Stk39 |
| GO:0031252 | cell leading edge | 1.42e-01 | 5.05e-01 | 2 | 51 | 214 | 16439 | 3.92% | 1.30% | 0.93% | 0.31% | mature\_B\_cell\_down | Nckap1, Rinl |
| GO:0005622 | intracellular | 1.44e-01 | 5.05e-01 | 39 | 51 | 11282 | 16439 | 76.47% | 68.63% | 0.35% | 0.31% | mature\_B\_cell\_down | Cd247, Ctsd, F2r, Fam125b, Fyb, Fyn, Gata3, Hnrpll, Ifi47, Ifih1, Ipcef1, Itk, Lat, Lck, Lcp2, Lpin1, Nckap1, Notch1, Prkch, Prkcq, Rab37, Rinl, Rnf125, Sept9, Sh2d2a, Skap1, Sla2, Slc9a3r1, Stk39, Sytl2, Tbc1d2b, Tcf7, Thy1, Tmem66, Tnfrsf1a, Tnik, Txk, Vps26b, Zap70 |
| GO:0055037 | recycling endosome | 1.49e-01 | 5.05e-01 | 1 | 51 | 52 | 16439 | 1.96% | 0.32% | 1.92% | 0.31% | mature\_B\_cell\_down | Tnik |
| GO:0030141 | secretory granule | 1.51e-01 | 5.05e-01 | 2 | 51 | 222 | 16439 | 3.92% | 1.35% | 0.90% | 0.31% | mature\_B\_cell\_down | Notch1, Rab37 |
| GO:0001725 | stress fiber | 1.52e-01 | 5.05e-01 | 1 | 51 | 53 | 16439 | 1.96% | 0.32% | 1.89% | 0.31% | mature\_B\_cell\_down | Sept9 |
| GO:0005737 | cytoplasm | 1.53e-01 | 5.05e-01 | 30 | 51 | 8331 | 16439 | 58.82% | 50.68% | 0.36% | 0.31% | mature\_B\_cell\_down | Cd247, Ctsd, Fam125b, Fyb, Fyn, Ifi47, Ifih1, Ipcef1, Itk, Lck, Lcp2, Lpin1, Nckap1, Notch1, Prkch, Prkcq, Rab37, Sept9, Sh2d2a, Skap1, Sla2, Slc9a3r1, Stk39, Sytl2, Thy1, Tmem66, Tnik, Txk, Vps26b, Zap70 |
| GO:0032432 | actin filament bundle | 1.60e-01 | 5.05e-01 | 1 | 51 | 56 | 16439 | 1.96% | 0.34% | 1.79% | 0.31% | mature\_B\_cell\_down | Sept9 |
| GO:0030139 | endocytic vesicle | 1.65e-01 | 5.05e-01 | 1 | 51 | 58 | 16439 | 1.96% | 0.35% | 1.72% | 0.31% | mature\_B\_cell\_down | Lck |
| GO:0042641 | actomyosin | 1.76e-01 | 5.05e-01 | 1 | 51 | 62 | 16439 | 1.96% | 0.38% | 1.61% | 0.31% | mature\_B\_cell\_down | Sept9 |
| GO:0044450 | microtubule organizing center part | 1.76e-01 | 5.05e-01 | 1 | 51 | 62 | 16439 | 1.96% | 0.38% | 1.61% | 0.31% | mature\_B\_cell\_down | Lck |
| GO:0030176 | integral to endoplasmic reticulum membrane | 1.78e-01 | 5.05e-01 | 1 | 51 | 63 | 16439 | 1.96% | 0.38% | 1.59% | 0.31% | mature\_B\_cell\_down | Tmem66 |
| GO:0005902 | microvillus | 1.78e-01 | 5.05e-01 | 1 | 51 | 63 | 16439 | 1.96% | 0.38% | 1.59% | 0.31% | mature\_B\_cell\_down | Slc9a3r1 |
| GO:0030133 | transport vesicle | 1.78e-01 | 5.05e-01 | 1 | 51 | 63 | 16439 | 1.96% | 0.38% | 1.59% | 0.31% | mature\_B\_cell\_down | Sytl2 |
| GO:0044464 | cell part | 1.79e-01 | 5.05e-01 | 44 | 51 | 13174 | 16439 | 86.27% | 80.14% | 0.33% | 0.31% | mature\_B\_cell\_down | Art2b, Cd226, Cd247, Cd27, Cd3g, Ctsd, F2r, Fam125b, Fyb, Fyn, Gata3, Hnrpll, Ifi47, Ifih1, Ipcef1, Itk, Lat, Lck, Lcp2, Lpin1, Nckap1, Notch1, Prkch, Prkcq, Rab37, Rinl, Rnf125, Selplg, Sept9, Sh2d2a, Skap1, Sla2, Slc9a3r1, Stk39, Sytl2, Tbc1d2b, Tcf7, Thy1, Tmem66, Tnfrsf1a, Tnik, Txk, Vps26b, Zap70 |
| GO:0005623 | cell | 1.79e-01 | 5.05e-01 | 44 | 51 | 13175 | 16439 | 86.27% | 80.14% | 0.33% | 0.31% | mature\_B\_cell\_down | Art2b, Cd226, Cd247, Cd27, Cd3g, Ctsd, F2r, Fam125b, Fyb, Fyn, Gata3, Hnrpll, Ifi47, Ifih1, Ipcef1, Itk, Lat, Lck, Lcp2, Lpin1, Nckap1, Notch1, Prkch, Prkcq, Rab37, Rinl, Rnf125, Selplg, Sept9, Sh2d2a, Skap1, Sla2, Slc9a3r1, Stk39, Sytl2, Tbc1d2b, Tcf7, Thy1, Tmem66, Tnfrsf1a, Tnik, Txk, Vps26b, Zap70 |
| GO:0005903 | brush border | 1.81e-01 | 5.05e-01 | 1 | 51 | 64 | 16439 | 1.96% | 0.39% | 1.56% | 0.31% | mature\_B\_cell\_down | Slc9a3r1 |
| GO:0005901 | caveola | 1.81e-01 | 5.05e-01 | 1 | 51 | 64 | 16439 | 1.96% | 0.39% | 1.56% | 0.31% | mature\_B\_cell\_down | F2r |
| GO:0009897 | external side of plasma membrane | 1.85e-01 | 5.08e-01 | 2 | 51 | 253 | 16439 | 3.92% | 1.54% | 0.79% | 0.31% | mature\_B\_cell\_down | Cd27, Thy1 |
| GO:0001669 | acrosomal vesicle | 1.96e-01 | 5.28e-01 | 1 | 51 | 70 | 16439 | 1.96% | 0.43% | 1.43% | 0.31% | mature\_B\_cell\_down | Notch1 |
| GO:0042995 | cell projection | 2.09e-01 | 5.55e-01 | 6 | 51 | 1292 | 16439 | 11.76% | 7.86% | 0.46% | 0.31% | mature\_B\_cell\_down | Fyn, Nckap1, Rinl, Slc9a3r1, Thy1, Tnfrsf1a |
| GO:0031227 | intrinsic to endoplasmic reticulum membrane | 2.21e-01 | 5.74e-01 | 1 | 51 | 80 | 16439 | 1.96% | 0.49% | 1.25% | 0.31% | mature\_B\_cell\_down | Tmem66 |
| GO:0045177 | apical part of cell | 2.32e-01 | 5.85e-01 | 2 | 51 | 294 | 16439 | 3.92% | 1.79% | 0.68% | 0.31% | mature\_B\_cell\_down | Slc9a3r1, Stk39 |
| GO:0045202 | synapse | 2.35e-01 | 5.85e-01 | 3 | 51 | 540 | 16439 | 5.88% | 3.28% | 0.56% | 0.31% | mature\_B\_cell\_down | F2r, Fyn, Prkcq |
| GO:0042383 | sarcolemma | 2.42e-01 | 5.85e-01 | 1 | 51 | 89 | 16439 | 1.96% | 0.54% | 1.12% | 0.31% | mature\_B\_cell\_down | Prkcq |
| GO:0001726 | ruffle | 2.45e-01 | 5.85e-01 | 1 | 51 | 90 | 16439 | 1.96% | 0.55% | 1.11% | 0.31% | mature\_B\_cell\_down | Rinl |
| GO:0005856 | cytoskeleton | 2.54e-01 | 5.85e-01 | 7 | 51 | 1662 | 16439 | 13.73% | 10.11% | 0.42% | 0.31% | mature\_B\_cell\_down | Fyn, Lck, Rinl, Sept9, Slc9a3r1, Stk39, Tnik |
| GO:0030426 | growth cone | 2.54e-01 | 5.85e-01 | 1 | 51 | 94 | 16439 | 1.96% | 0.57% | 1.06% | 0.31% | mature\_B\_cell\_down | Thy1 |
| GO:0030427 | site of polarized growth | 2.54e-01 | 5.85e-01 | 1 | 51 | 94 | 16439 | 1.96% | 0.57% | 1.06% | 0.31% | mature\_B\_cell\_down | Thy1 |
| GO:0030027 | lamellipodium | 2.59e-01 | 5.85e-01 | 1 | 51 | 96 | 16439 | 1.96% | 0.58% | 1.04% | 0.31% | mature\_B\_cell\_down | Nckap1 |
| GO:0044463 | cell projection part | 2.61e-01 | 5.85e-01 | 3 | 51 | 572 | 16439 | 5.88% | 3.48% | 0.52% | 0.31% | mature\_B\_cell\_down | Fyn, Slc9a3r1, Thy1 |
| GO:0015629 | actin cytoskeleton | 2.66e-01 | 5.85e-01 | 2 | 51 | 324 | 16439 | 3.92% | 1.97% | 0.62% | 0.31% | mature\_B\_cell\_down | Rinl, Sept9 |
| GO:0005667 | transcription factor complex | 2.68e-01 | 5.85e-01 | 2 | 51 | 326 | 16439 | 3.92% | 1.98% | 0.61% | 0.31% | mature\_B\_cell\_down | Lpin1, Tcf7 |
| GO:0031965 | nuclear membrane | 2.72e-01 | 5.85e-01 | 1 | 51 | 102 | 16439 | 1.96% | 0.62% | 0.98% | 0.31% | mature\_B\_cell\_down | Lpin1 |
| GO:0005625 | soluble fraction | 2.77e-01 | 5.85e-01 | 2 | 51 | 333 | 16439 | 3.92% | 2.03% | 0.60% | 0.31% | mature\_B\_cell\_down | Ctsd, Prkcq |
| GO:0005770 | late endosome | 2.79e-01 | 5.85e-01 | 1 | 51 | 105 | 16439 | 1.96% | 0.64% | 0.95% | 0.31% | mature\_B\_cell\_down | Sla2 |
| GO:0005813 | centrosome | 2.90e-01 | 5.97e-01 | 2 | 51 | 345 | 16439 | 3.92% | 2.10% | 0.58% | 0.31% | mature\_B\_cell\_down | Lck, Slc9a3r1 |
| GO:0005741 | mitochondrial outer membrane | 2.93e-01 | 5.97e-01 | 1 | 51 | 111 | 16439 | 1.96% | 0.68% | 0.90% | 0.31% | mature\_B\_cell\_down | Lpin1 |
| GO:0044327 | dendritic spine head | 3.01e-01 | 5.99e-01 | 1 | 51 | 115 | 16439 | 1.96% | 0.70% | 0.87% | 0.31% | mature\_B\_cell\_down | Fyn |
| GO:0014069 | postsynaptic density | 3.01e-01 | 5.99e-01 | 1 | 51 | 115 | 16439 | 1.96% | 0.70% | 0.87% | 0.31% | mature\_B\_cell\_down | Fyn |
| GO:0048471 | perinuclear region of cytoplasm | 3.13e-01 | 6.12e-01 | 2 | 51 | 365 | 16439 | 3.92% | 2.22% | 0.55% | 0.31% | mature\_B\_cell\_down | Sept9, Tnik |
| GO:0005815 | microtubule organizing center | 3.17e-01 | 6.12e-01 | 2 | 51 | 368 | 16439 | 3.92% | 2.24% | 0.54% | 0.31% | mature\_B\_cell\_down | Lck, Slc9a3r1 |
| GO:0031968 | organelle outer membrane | 3.25e-01 | 6.12e-01 | 1 | 51 | 126 | 16439 | 1.96% | 0.77% | 0.79% | 0.31% | mature\_B\_cell\_down | Lpin1 |
| GO:0005829 | cytosol | 3.29e-01 | 6.12e-01 | 4 | 51 | 934 | 16439 | 7.84% | 5.68% | 0.43% | 0.31% | mature\_B\_cell\_down | Ipcef1, Prkch, Thy1, Zap70 |
| GO:0031224 | intrinsic to membrane | 3.33e-01 | 6.12e-01 | 20 | 51 | 5824 | 16439 | 39.22% | 35.43% | 0.34% | 0.31% | mature\_B\_cell\_down | Art2b, Btbd11, Cd226, Cd247, Cd27, Cd3g, Cd7, Cd96, Enpp4, F2r, Lat, Leprotl1, Nckap1, Notch1, Selplg, Slc12a7, Thy1, Tmem66, Tnfrsf1a, Zdhhc15 |
| GO:0019867 | outer membrane | 3.33e-01 | 6.12e-01 | 1 | 51 | 130 | 16439 | 1.96% | 0.79% | 0.77% | 0.31% | mature\_B\_cell\_down | Lpin1 |
| GO:0044456 | synapse part | 3.36e-01 | 6.12e-01 | 2 | 51 | 385 | 16439 | 3.92% | 2.34% | 0.52% | 0.31% | mature\_B\_cell\_down | F2r, Fyn |
| GO:0030425 | dendrite | 3.41e-01 | 6.13e-01 | 2 | 51 | 389 | 16439 | 3.92% | 2.37% | 0.51% | 0.31% | mature\_B\_cell\_down | Fyn, Thy1 |
| GO:0000790 | nuclear chromatin | 3.46e-01 | 6.14e-01 | 1 | 51 | 136 | 16439 | 1.96% | 0.83% | 0.74% | 0.31% | mature\_B\_cell\_down | Gata3 |
| GO:0031253 | cell projection membrane | 3.52e-01 | 6.18e-01 | 1 | 51 | 139 | 16439 | 1.96% | 0.85% | 0.72% | 0.31% | mature\_B\_cell\_down | Slc9a3r1 |
| GO:0031226 | intrinsic to plasma membrane | 3.57e-01 | 6.19e-01 | 3 | 51 | 684 | 16439 | 5.88% | 4.16% | 0.44% | 0.31% | mature\_B\_cell\_down | Lat, Notch1, Thy1 |
| GO:0044424 | intracellular part | 3.61e-01 | 6.19e-01 | 36 | 51 | 11042 | 16439 | 70.59% | 67.17% | 0.33% | 0.31% | mature\_B\_cell\_down | Cd247, Ctsd, F2r, Fam125b, Fyb, Fyn, Gata3, Hnrpll, Ifi47, Ifih1, Ipcef1, Itk, Lck, Lcp2, Lpin1, Nckap1, Notch1, Prkch, Prkcq, Rab37, Rinl, Sept9, Sh2d2a, Skap1, Sla2, Slc9a3r1, Stk39, Sytl2, Tcf7, Thy1, Tmem66, Tnfrsf1a, Tnik, Txk, Vps26b, Zap70 |
| GO:0043005 | neuron projection | 3.70e-01 | 6.28e-01 | 3 | 51 | 699 | 16439 | 5.88% | 4.25% | 0.43% | 0.31% | mature\_B\_cell\_down | Fyn, Thy1, Tnfrsf1a |
| GO:0031301 | integral to organelle membrane | 3.76e-01 | 6.28e-01 | 1 | 51 | 151 | 16439 | 1.96% | 0.92% | 0.66% | 0.31% | mature\_B\_cell\_down | Tmem66 |
| GO:0015630 | microtubule cytoskeleton | 3.78e-01 | 6.28e-01 | 3 | 51 | 709 | 16439 | 5.88% | 4.31% | 0.42% | 0.31% | mature\_B\_cell\_down | Lck, Sept9, Slc9a3r1 |
| GO:0043197 | dendritic spine | 3.97e-01 | 6.42e-01 | 1 | 51 | 162 | 16439 | 1.96% | 0.99% | 0.62% | 0.31% | mature\_B\_cell\_down | Fyn |
| GO:0044309 | neuron spine | 3.97e-01 | 6.42e-01 | 1 | 51 | 162 | 16439 | 1.96% | 0.99% | 0.62% | 0.31% | mature\_B\_cell\_down | Fyn |
| GO:0012505 | endomembrane system | 4.00e-01 | 6.42e-01 | 3 | 51 | 734 | 16439 | 5.88% | 4.46% | 0.41% | 0.31% | mature\_B\_cell\_down | Lpin1, Tmem66, Vps26b |
| GO:0031300 | intrinsic to organelle membrane | 4.14e-01 | 6.58e-01 | 1 | 51 | 171 | 16439 | 1.96% | 1.04% | 0.58% | 0.31% | mature\_B\_cell\_down | Tmem66 |
| GO:0045211 | postsynaptic membrane | 4.28e-01 | 6.74e-01 | 1 | 51 | 179 | 16439 | 1.96% | 1.09% | 0.56% | 0.31% | mature\_B\_cell\_down | F2r |
| GO:0044444 | cytoplasmic part | 4.35e-01 | 6.77e-01 | 18 | 51 | 5480 | 16439 | 35.29% | 33.34% | 0.33% | 0.31% | mature\_B\_cell\_down | Ctsd, Fam125b, Fyn, Ifi47, Ipcef1, Lck, Lpin1, Notch1, Prkch, Rab37, Sept9, Sla2, Slc9a3r1, Sytl2, Thy1, Tmem66, Tnik, Zap70 |
| GO:0005783 | endoplasmic reticulum | 4.43e-01 | 6.82e-01 | 4 | 51 | 1093 | 16439 | 7.84% | 6.65% | 0.37% | 0.31% | mature\_B\_cell\_down | Ifi47, Lpin1, Thy1, Tmem66 |
| GO:0044430 | cytoskeletal part | 4.64e-01 | 7.08e-01 | 4 | 51 | 1124 | 16439 | 7.84% | 6.84% | 0.36% | 0.31% | mature\_B\_cell\_down | Fyn, Lck, Sept9, Slc9a3r1 |
| GO:0097060 | synaptic membrane | 4.77e-01 | 7.15e-01 | 1 | 51 | 207 | 16439 | 1.96% | 1.26% | 0.48% | 0.31% | mature\_B\_cell\_down | F2r |
| GO:0005635 | nuclear envelope | 4.81e-01 | 7.15e-01 | 1 | 51 | 210 | 16439 | 1.96% | 1.28% | 0.48% | 0.31% | mature\_B\_cell\_down | Lpin1 |
| GO:0005792 | microsome | 4.83e-01 | 7.15e-01 | 1 | 51 | 211 | 16439 | 1.96% | 1.28% | 0.47% | 0.31% | mature\_B\_cell\_down | Fyn |
| GO:0042598 | vesicular fraction | 4.93e-01 | 7.22e-01 | 1 | 51 | 217 | 16439 | 1.96% | 1.32% | 0.46% | 0.31% | mature\_B\_cell\_down | Fyn |
| GO:0044454 | nuclear chromosome part | 5.04e-01 | 7.31e-01 | 1 | 51 | 224 | 16439 | 1.96% | 1.36% | 0.45% | 0.31% | mature\_B\_cell\_down | Gata3 |
| GO:0016021 | integral to membrane | 5.12e-01 | 7.33e-01 | 18 | 51 | 5690 | 16439 | 35.29% | 34.61% | 0.32% | 0.31% | mature\_B\_cell\_down | Btbd11, Cd226, Cd247, Cd27, Cd3g, Cd7, Cd96, Enpp4, F2r, Lat, Leprotl1, Nckap1, Notch1, Selplg, Slc12a7, Tmem66, Tnfrsf1a, Zdhhc15 |
| GO:0005789 | endoplasmic reticulum membrane | 5.15e-01 | 7.33e-01 | 1 | 51 | 231 | 16439 | 1.96% | 1.41% | 0.43% | 0.31% | mature\_B\_cell\_down | Tmem66 |
| GO:0042175 | nuclear outer membrane-endoplasmic reticulum membrane network | 5.40e-01 | 7.61e-01 | 1 | 51 | 248 | 16439 | 1.96% | 1.51% | 0.40% | 0.31% | mature\_B\_cell\_down | Tmem66 |
| GO:0016323 | basolateral plasma membrane | 5.44e-01 | 7.61e-01 | 1 | 51 | 251 | 16439 | 1.96% | 1.53% | 0.40% | 0.31% | mature\_B\_cell\_down | Stk39 |
| GO:0000228 | nuclear chromosome | 5.53e-01 | 7.64e-01 | 1 | 51 | 257 | 16439 | 1.96% | 1.56% | 0.39% | 0.31% | mature\_B\_cell\_down | Gata3 |
| GO:0005764 | lysosome | 5.64e-01 | 7.64e-01 | 1 | 51 | 265 | 16439 | 1.96% | 1.61% | 0.38% | 0.31% | mature\_B\_cell\_down | Ctsd |
| GO:0000323 | lytic vacuole | 5.64e-01 | 7.64e-01 | 1 | 51 | 265 | 16439 | 1.96% | 1.61% | 0.38% | 0.31% | mature\_B\_cell\_down | Ctsd |
| GO:0000785 | chromatin | 5.68e-01 | 7.64e-01 | 1 | 51 | 268 | 16439 | 1.96% | 1.63% | 0.37% | 0.31% | mature\_B\_cell\_down | Gata3 |
| GO:0044432 | endoplasmic reticulum part | 5.76e-01 | 7.64e-01 | 1 | 51 | 274 | 16439 | 1.96% | 1.67% | 0.36% | 0.31% | mature\_B\_cell\_down | Tmem66 |
| GO:0043234 | protein complex | 5.77e-01 | 7.64e-01 | 9 | 51 | 2944 | 16439 | 17.65% | 17.91% | 0.31% | 0.31% | mature\_B\_cell\_down | Cd247, Cd3g, Lpin1, Notch1, Sept9, Tcf7, Tnfrsf1a, Vps26b, Zap70 |
| GO:0005874 | microtubule | 5.88e-01 | 7.70e-01 | 1 | 51 | 283 | 16439 | 1.96% | 1.72% | 0.35% | 0.31% | mature\_B\_cell\_down | Sept9 |
| GO:0005794 | Golgi apparatus | 5.95e-01 | 7.70e-01 | 3 | 51 | 983 | 16439 | 5.88% | 5.98% | 0.31% | 0.31% | mature\_B\_cell\_down | Lck, Notch1, Sytl2 |
| GO:0005887 | integral to plasma membrane | 5.97e-01 | 7.70e-01 | 2 | 51 | 641 | 16439 | 3.92% | 3.90% | 0.31% | 0.31% | mature\_B\_cell\_down | Lat, Notch1 |
| GO:0005773 | vacuole | 6.22e-01 | 7.94e-01 | 1 | 51 | 310 | 16439 | 1.96% | 1.89% | 0.32% | 0.31% | mature\_B\_cell\_down | Ctsd |
| GO:0030424 | axon | 6.25e-01 | 7.94e-01 | 1 | 51 | 313 | 16439 | 1.96% | 1.90% | 0.32% | 0.31% | mature\_B\_cell\_down | Tnfrsf1a |
| GO:0044451 | nucleoplasm part | 6.70e-01 | 8.41e-01 | 2 | 51 | 732 | 16439 | 3.92% | 4.45% | 0.27% | 0.31% | mature\_B\_cell\_down | Lpin1, Tcf7 |
| GO:0043232 | intracellular non-membrane-bounded organelle | 6.80e-01 | 8.41e-01 | 8 | 51 | 2851 | 16439 | 15.69% | 17.34% | 0.28% | 0.31% | mature\_B\_cell\_down | Fyn, Gata3, Lck, Rinl, Sept9, Slc9a3r1, Stk39, Tnik |
| GO:0043228 | non-membrane-bounded organelle | 6.80e-01 | 8.41e-01 | 8 | 51 | 2851 | 16439 | 15.69% | 17.34% | 0.28% | 0.31% | mature\_B\_cell\_down | Fyn, Gata3, Lck, Rinl, Sept9, Slc9a3r1, Stk39, Tnik |
| GO:0032991 | macromolecular complex | 6.93e-01 | 8.45e-01 | 10 | 51 | 3562 | 16439 | 19.61% | 21.67% | 0.28% | 0.31% | mature\_B\_cell\_down | Cd247, Cd3g, Hnrpll, Lpin1, Notch1, Sept9, Tcf7, Tnfrsf1a, Vps26b, Zap70 |
| GO:0043231 | intracellular membrane-bounded organelle | 6.94e-01 | 8.45e-01 | 25 | 51 | 8480 | 16439 | 49.02% | 51.58% | 0.29% | 0.31% | mature\_B\_cell\_down | Ctsd, F2r, Fam125b, Fyb, Fyn, Gata3, Hnrpll, Ifi47, Ifih1, Lck, Lpin1, Notch1, Prkcq, Rab37, Skap1, Sla2, Slc9a3r1, Stk39, Sytl2, Tcf7, Thy1, Tmem66, Tnfrsf1a, Tnik, Txk |
| GO:0043227 | membrane-bounded organelle | 7.00e-01 | 8.46e-01 | 25 | 51 | 8499 | 16439 | 49.02% | 51.70% | 0.29% | 0.31% | mature\_B\_cell\_down | Ctsd, F2r, Fam125b, Fyb, Fyn, Gata3, Hnrpll, Ifi47, Ifih1, Lck, Lpin1, Notch1, Prkcq, Rab37, Skap1, Sla2, Slc9a3r1, Stk39, Sytl2, Tcf7, Thy1, Tmem66, Tnfrsf1a, Tnik, Txk |
| GO:0005615 | extracellular space | 7.11e-01 | 8.52e-01 | 2 | 51 | 791 | 16439 | 3.92% | 4.81% | 0.25% | 0.31% | mature\_B\_cell\_down | Ctsd, Tnfrsf1a |
| GO:0005654 | nucleoplasm | 7.32e-01 | 8.70e-01 | 2 | 51 | 823 | 16439 | 3.92% | 5.01% | 0.24% | 0.31% | mature\_B\_cell\_down | Lpin1, Tcf7 |
| GO:0031966 | mitochondrial membrane | 7.56e-01 | 8.92e-01 | 1 | 51 | 448 | 16439 | 1.96% | 2.73% | 0.22% | 0.31% | mature\_B\_cell\_down | Lpin1 |
| GO:0005740 | mitochondrial envelope | 7.73e-01 | 8.97e-01 | 1 | 51 | 471 | 16439 | 1.96% | 2.87% | 0.21% | 0.31% | mature\_B\_cell\_down | Lpin1 |
| GO:0005730 | nucleolus | 7.73e-01 | 8.97e-01 | 1 | 51 | 471 | 16439 | 1.96% | 2.87% | 0.21% | 0.31% | mature\_B\_cell\_down | Gata3 |
| GO:0044427 | chromosomal part | 7.84e-01 | 8.97e-01 | 1 | 51 | 486 | 16439 | 1.96% | 2.96% | 0.21% | 0.31% | mature\_B\_cell\_down | Gata3 |
| GO:0005634 | nucleus | 7.84e-01 | 8.97e-01 | 14 | 51 | 5200 | 16439 | 27.45% | 31.63% | 0.27% | 0.31% | mature\_B\_cell\_down | Fyb, Gata3, Hnrpll, Ifih1, Lpin1, Notch1, Prkcq, Skap1, Stk39, Sytl2, Tcf7, Tnfrsf1a, Tnik, Txk |
| GO:0031981 | nuclear lumen | 8.22e-01 | 9.13e-01 | 3 | 51 | 1402 | 16439 | 5.88% | 8.53% | 0.21% | 0.31% | mature\_B\_cell\_down | Gata3, Lpin1, Tcf7 |
| GO:0043229 | intracellular organelle | 8.24e-01 | 9.13e-01 | 27 | 51 | 9595 | 16439 | 52.94% | 58.37% | 0.28% | 0.31% | mature\_B\_cell\_down | Ctsd, F2r, Fam125b, Fyb, Fyn, Gata3, Hnrpll, Ifi47, Ifih1, Lck, Lpin1, Notch1, Prkcq, Rab37, Rinl, Sept9, Skap1, Sla2, Slc9a3r1, Stk39, Sytl2, Tcf7, Thy1, Tmem66, Tnfrsf1a, Tnik, Txk |
| GO:0043226 | organelle | 8.29e-01 | 9.13e-01 | 27 | 51 | 9616 | 16439 | 52.94% | 58.50% | 0.28% | 0.31% | mature\_B\_cell\_down | Ctsd, F2r, Fam125b, Fyb, Fyn, Gata3, Hnrpll, Ifi47, Ifih1, Lck, Lpin1, Notch1, Prkcq, Rab37, Rinl, Sept9, Skap1, Sla2, Slc9a3r1, Stk39, Sytl2, Tcf7, Thy1, Tmem66, Tnfrsf1a, Tnik, Txk |
| GO:0005694 | chromosome | 8.34e-01 | 9.13e-01 | 1 | 51 | 568 | 16439 | 1.96% | 3.46% | 0.18% | 0.31% | mature\_B\_cell\_down | Gata3 |
| GO:0044421 | extracellular region part | 8.37e-01 | 9.13e-01 | 2 | 51 | 1029 | 16439 | 3.92% | 6.26% | 0.19% | 0.31% | mature\_B\_cell\_down | Ctsd, Tnfrsf1a |
| GO:0044429 | mitochondrial part | 8.38e-01 | 9.13e-01 | 1 | 51 | 575 | 16439 | 1.96% | 3.50% | 0.17% | 0.31% | mature\_B\_cell\_down | Lpin1 |
| GO:0030529 | ribonucleoprotein complex | 8.40e-01 | 9.13e-01 | 1 | 51 | 580 | 16439 | 1.96% | 3.53% | 0.17% | 0.31% | mature\_B\_cell\_down | Hnrpll |
| GO:0031090 | organelle membrane | 8.78e-01 | 9.23e-01 | 2 | 51 | 1143 | 16439 | 3.92% | 6.95% | 0.17% | 0.31% | mature\_B\_cell\_down | Lpin1, Tmem66 |
| GO:0005739 | mitochondrion | 8.79e-01 | 9.23e-01 | 3 | 51 | 1575 | 16439 | 5.88% | 9.58% | 0.19% | 0.31% | mature\_B\_cell\_down | Ctsd, Fyn, Lpin1 |
| GO:0031967 | organelle envelope | 8.82e-01 | 9.23e-01 | 1 | 51 | 674 | 16439 | 1.96% | 4.10% | 0.15% | 0.31% | mature\_B\_cell\_down | Lpin1 |
| GO:0070013 | intracellular organelle lumen | 8.83e-01 | 9.23e-01 | 3 | 51 | 1593 | 16439 | 5.88% | 9.69% | 0.19% | 0.31% | mature\_B\_cell\_down | Gata3, Lpin1, Tcf7 |
| GO:0043233 | organelle lumen | 8.85e-01 | 9.23e-01 | 3 | 51 | 1598 | 16439 | 5.88% | 9.72% | 0.19% | 0.31% | mature\_B\_cell\_down | Gata3, Lpin1, Tcf7 |
| GO:0031975 | envelope | 8.86e-01 | 9.23e-01 | 1 | 51 | 685 | 16439 | 1.96% | 4.17% | 0.15% | 0.31% | mature\_B\_cell\_down | Lpin1 |
| GO:0031974 | membrane-enclosed lumen | 8.95e-01 | 9.26e-01 | 3 | 51 | 1639 | 16439 | 5.88% | 9.97% | 0.18% | 0.31% | mature\_B\_cell\_down | Gata3, Lpin1, Tcf7 |
| GO:0044428 | nuclear part | 9.22e-01 | 9.47e-01 | 3 | 51 | 1765 | 16439 | 5.88% | 10.74% | 0.17% | 0.31% | mature\_B\_cell\_down | Gata3, Lpin1, Tcf7 |
| GO:0044446 | intracellular organelle part | 9.65e-01 | 9.84e-01 | 8 | 51 | 4160 | 16439 | 15.69% | 25.31% | 0.19% | 0.31% | mature\_B\_cell\_down | Fyn, Gata3, Lck, Lpin1, Sept9, Slc9a3r1, Tcf7, Tmem66 |
| GO:0044422 | organelle part | 9.71e-01 | 9.84e-01 | 8 | 51 | 4252 | 16439 | 15.69% | 25.87% | 0.19% | 0.31% | mature\_B\_cell\_down | Fyn, Gata3, Lck, Lpin1, Sept9, Slc9a3r1, Tcf7, Tmem66 |
| GO:0005576 | extracellular region | 9.81e-01 | 9.88e-01 | 2 | 51 | 1812 | 16439 | 3.92% | 11.02% | 0.11% | 0.31% | mature\_B\_cell\_down | Ctsd, Tnfrsf1a |
| GO:0005575 | cellular\_component | 1.00e+00 | 1.00e+00 | 51 | 51 | 16439 | 16439 | 100.00% | 100.00% | 0.31% | 0.31% | mature\_B\_cell\_down | Art2b, Btbd11, Cd226, Cd247, Cd27, Cd3g, Cd7, Cd96, Ctsd, Enpp4, F2r, Fam125b, Fyb, Fyn, Gata3, Hnrpll, Ifi47, Ifih1, Ipcef1, Itk, Lat, Lck, Lcp2, Leprotl1, Lpin1, Nckap1, Notch1, Prkch, Prkcq, Rab37, Rinl, Rnf125, Selplg, Sept9, Sh2d2a, Skap1, Sla2, Slc12a7, Slc9a3r1, Stk39, Sytl2, Tbc1d2b, Tcf7, Thy1, Tmem66, Tnfrsf1a, Tnik, Txk, Vps26b, Zap70, Zdhhc15 |

### molecular\_function (top)

  
  


| TermID | Term | P | Q | k | n | K | N | k/n | K/N | k/K | n/N | Qset | Symbols |
| --- | --- | --- | --- | --- | --- | --- | --- | --- | --- | --- | --- | --- | --- |
| GO:0004715 | non-membrane spanning protein tyrosine kinase activity | 2.24e-07 | 4.78e-05 | 5 | 47 | 43 | 14853 | 10.64% | 0.29% | 11.63% | 0.32% | mature\_B\_cell\_down | Fyn, Itk, Lck, Txk, Zap70 |
| GO:0042610 | CD8 receptor binding | 9.80e-06 | 1.04e-03 | 2 | 47 | 2 | 14853 | 4.26% | 0.01% | 100.00% | 0.32% | mature\_B\_cell\_down | Fyn, Lck |
| GO:0005070 | SH3/SH2 adaptor activity | 2.78e-05 | 1.52e-03 | 3 | 47 | 19 | 14853 | 6.38% | 0.13% | 15.79% | 0.32% | mature\_B\_cell\_down | Lat, Sh2d2a, Sla2 |
| GO:0019899 | enzyme binding | 2.86e-05 | 1.52e-03 | 13 | 47 | 1116 | 14853 | 27.66% | 7.51% | 1.16% | 0.32% | mature\_B\_cell\_down | Cd226, Fyn, Lat, Lck, Lpin1, Notch1, Prkch, Skap1, Slc9a3r1, Stk39, Sytl2, Thy1, Tnfrsf1a |
| GO:0032403 | protein complex binding | 4.27e-05 | 1.82e-03 | 8 | 47 | 416 | 14853 | 17.02% | 2.80% | 1.92% | 0.32% | mature\_B\_cell\_down | Cd226, F2r, Fyb, Fyn, Lck, Skap1, Thy1, Tnfrsf1a |
| GO:0004672 | protein kinase activity | 5.54e-05 | 1.97e-03 | 9 | 47 | 561 | 14853 | 19.15% | 3.78% | 1.60% | 0.32% | mature\_B\_cell\_down | Fyn, Itk, Lck, Prkch, Prkcq, Stk39, Tnik, Txk, Zap70 |
| GO:0004713 | protein tyrosine kinase activity | 6.73e-05 | 2.05e-03 | 5 | 47 | 136 | 14853 | 10.64% | 0.92% | 3.68% | 0.32% | mature\_B\_cell\_down | Fyn, Itk, Lck, Txk, Zap70 |
| GO:0042609 | CD4 receptor binding | 9.74e-05 | 2.59e-03 | 2 | 47 | 5 | 14853 | 4.26% | 0.03% | 40.00% | 0.32% | mature\_B\_cell\_down | Fyn, Lck |
| GO:0005515 | protein binding | 1.18e-04 | 2.67e-03 | 32 | 47 | 6013 | 14853 | 68.09% | 40.48% | 0.53% | 0.32% | mature\_B\_cell\_down | Cd226, Cd247, Cd27, Cd3g, F2r, Fyb, Fyn, Gata3, Hpcal1, Ifih1, Ipcef1, Itk, Lat, Lck, Lpin1, Notch1, Prkch, Prkcq, Rab37, Rinl, Selplg, Sh2d2a, Skap1, Sla2, Slc9a3r1, Stk39, Sytl2, Tcf7, Thy1, Tnfrsf1a, Tnik, Zap70 |
| GO:0035591 | signaling adaptor activity | 1.25e-04 | 2.67e-03 | 3 | 47 | 31 | 14853 | 6.38% | 0.21% | 9.68% | 0.32% | mature\_B\_cell\_down | Lat, Sh2d2a, Sla2 |
| GO:0016773 | phosphotransferase activity, alcohol group as acceptor | 2.05e-04 | 3.96e-03 | 9 | 47 | 666 | 14853 | 19.15% | 4.48% | 1.35% | 0.32% | mature\_B\_cell\_down | Fyn, Itk, Lck, Prkch, Prkcq, Stk39, Tnik, Txk, Zap70 |
| GO:0019900 | kinase binding | 4.25e-04 | 7.54e-03 | 7 | 47 | 439 | 14853 | 14.89% | 2.96% | 1.59% | 0.32% | mature\_B\_cell\_down | Cd226, Fyn, Lat, Lck, Skap1, Stk39, Thy1 |
| GO:0016301 | kinase activity | 6.41e-04 | 1.05e-02 | 9 | 47 | 778 | 14853 | 19.15% | 5.24% | 1.16% | 0.32% | mature\_B\_cell\_down | Fyn, Itk, Lck, Prkch, Prkcq, Stk39, Tnik, Txk, Zap70 |
| GO:0019901 | protein kinase binding | 1.23e-03 | 1.84e-02 | 6 | 47 | 382 | 14853 | 12.77% | 2.57% | 1.57% | 0.32% | mature\_B\_cell\_down | Cd226, Lat, Lck, Skap1, Stk39, Thy1 |
| GO:0004697 | protein kinase C activity | 1.29e-03 | 1.84e-02 | 2 | 47 | 17 | 14853 | 4.26% | 0.11% | 11.76% | 0.32% | mature\_B\_cell\_down | Prkch, Prkcq |
| GO:0030674 | protein binding, bridging | 1.84e-03 | 2.32e-02 | 3 | 47 | 77 | 14853 | 6.38% | 0.52% | 3.90% | 0.32% | mature\_B\_cell\_down | Lat, Sh2d2a, Sla2 |
| GO:0016772 | transferase activity, transferring phosphorus-containing groups | 1.85e-03 | 2.32e-02 | 9 | 47 | 904 | 14853 | 19.15% | 6.09% | 1.00% | 0.32% | mature\_B\_cell\_down | Fyn, Itk, Lck, Prkch, Prkcq, Stk39, Tnik, Txk, Zap70 |
| GO:0005543 | phospholipid binding | 1.99e-03 | 2.35e-02 | 6 | 47 | 420 | 14853 | 12.77% | 2.83% | 1.43% | 0.32% | mature\_B\_cell\_down | Ipcef1, Itk, Skap1, Sytl2, Tbc1d2b, Thy1 |
| GO:0043548 | phosphatidylinositol 3-kinase binding | 2.59e-03 | 2.90e-02 | 2 | 47 | 24 | 14853 | 4.26% | 0.16% | 8.33% | 0.32% | mature\_B\_cell\_down | Fyn, Lck |
| GO:0042169 | SH2 domain binding | 4.86e-03 | 5.18e-02 | 2 | 47 | 33 | 14853 | 4.26% | 0.22% | 6.06% | 0.32% | mature\_B\_cell\_down | Lck, Skap1 |
| GO:0060090 | binding, bridging | 5.59e-03 | 5.61e-02 | 3 | 47 | 114 | 14853 | 6.38% | 0.77% | 2.63% | 0.32% | mature\_B\_cell\_down | Lat, Sh2d2a, Sla2 |
| GO:0034235 | GPI anchor binding | 6.32e-03 | 5.61e-02 | 1 | 47 | 2 | 14853 | 2.13% | 0.01% | 50.00% | 0.32% | mature\_B\_cell\_down | Thy1 |
| GO:0043120 | tumor necrosis factor binding | 6.32e-03 | 5.61e-02 | 1 | 47 | 2 | 14853 | 2.13% | 0.01% | 50.00% | 0.32% | mature\_B\_cell\_down | Tnfrsf1a |
| GO:0035639 | purine ribonucleoside triphosphate binding | 6.32e-03 | 5.61e-02 | 12 | 47 | 1728 | 14853 | 25.53% | 11.63% | 0.69% | 0.32% | mature\_B\_cell\_down | Fyn, Ifih1, Itk, Lck, Prkch, Prkcq, Rab37, Sept9, Stk39, Tnik, Txk, Zap70 |
| GO:0032555 | purine ribonucleotide binding | 7.34e-03 | 6.00e-02 | 12 | 47 | 1761 | 14853 | 25.53% | 11.86% | 0.68% | 0.32% | mature\_B\_cell\_down | Fyn, Ifih1, Itk, Lck, Prkch, Prkcq, Rab37, Sept9, Stk39, Tnik, Txk, Zap70 |
| GO:0032553 | ribonucleotide binding | 7.38e-03 | 6.00e-02 | 12 | 47 | 1762 | 14853 | 25.53% | 11.86% | 0.68% | 0.32% | mature\_B\_cell\_down | Fyn, Ifih1, Itk, Lck, Prkch, Prkcq, Rab37, Sept9, Stk39, Tnik, Txk, Zap70 |
| GO:0017076 | purine nucleotide binding | 7.61e-03 | 6.00e-02 | 12 | 47 | 1769 | 14853 | 25.53% | 11.91% | 0.68% | 0.32% | mature\_B\_cell\_down | Fyn, Ifih1, Itk, Lck, Prkch, Prkcq, Rab37, Sept9, Stk39, Tnik, Txk, Zap70 |
| GO:0003823 | antigen binding | 8.90e-03 | 6.77e-02 | 2 | 47 | 45 | 14853 | 4.26% | 0.30% | 4.44% | 0.32% | mature\_B\_cell\_down | Lck, Skap1 |
| GO:0004699 | calcium-independent protein kinase C activity | 9.46e-03 | 6.95e-02 | 1 | 47 | 3 | 14853 | 2.13% | 0.02% | 33.33% | 0.32% | mature\_B\_cell\_down | Prkch |
| GO:0019904 | protein domain specific binding | 1.12e-02 | 7.23e-02 | 6 | 47 | 601 | 14853 | 12.77% | 4.05% | 1.00% | 0.32% | mature\_B\_cell\_down | Gata3, Ipcef1, Lck, Sh2d2a, Skap1, Slc9a3r1 |
| GO:0001047 | core promoter binding | 1.13e-02 | 7.23e-02 | 2 | 47 | 51 | 14853 | 4.26% | 0.34% | 3.92% | 0.32% | mature\_B\_cell\_down | Gata3, Notch1 |
| GO:0005524 | ATP binding | 1.18e-02 | 7.23e-02 | 10 | 47 | 1416 | 14853 | 21.28% | 9.53% | 0.71% | 0.32% | mature\_B\_cell\_down | Fyn, Ifih1, Itk, Lck, Prkch, Prkcq, Stk39, Tnik, Txk, Zap70 |
| GO:0042608 | T cell receptor binding | 1.26e-02 | 7.23e-02 | 1 | 47 | 4 | 14853 | 2.13% | 0.03% | 25.00% | 0.32% | mature\_B\_cell\_down | Fyn |
| GO:0031698 | beta-2 adrenergic receptor binding | 1.26e-02 | 7.23e-02 | 1 | 47 | 4 | 14853 | 2.13% | 0.03% | 25.00% | 0.32% | mature\_B\_cell\_down | Slc9a3r1 |
| GO:0017081 | chloride channel regulator activity | 1.26e-02 | 7.23e-02 | 1 | 47 | 4 | 14853 | 2.13% | 0.03% | 25.00% | 0.32% | mature\_B\_cell\_down | Slc9a3r1 |
| GO:0015379 | potassium:chloride symporter activity | 1.26e-02 | 7.23e-02 | 1 | 47 | 4 | 14853 | 2.13% | 0.03% | 25.00% | 0.32% | mature\_B\_cell\_down | Slc12a7 |
| GO:0015057 | thrombin receptor activity | 1.26e-02 | 7.23e-02 | 1 | 47 | 4 | 14853 | 2.13% | 0.03% | 25.00% | 0.32% | mature\_B\_cell\_down | F2r |
| GO:0032559 | adenyl ribonucleotide binding | 1.32e-02 | 7.23e-02 | 10 | 47 | 1440 | 14853 | 21.28% | 9.70% | 0.69% | 0.32% | mature\_B\_cell\_down | Fyn, Ifih1, Itk, Lck, Prkch, Prkcq, Stk39, Tnik, Txk, Zap70 |
| GO:0008289 | lipid binding | 1.34e-02 | 7.23e-02 | 6 | 47 | 625 | 14853 | 12.77% | 4.21% | 0.96% | 0.32% | mature\_B\_cell\_down | Ipcef1, Itk, Skap1, Sytl2, Tbc1d2b, Thy1 |
| GO:0030554 | adenyl nucleotide binding | 1.36e-02 | 7.23e-02 | 10 | 47 | 1447 | 14853 | 21.28% | 9.74% | 0.69% | 0.32% | mature\_B\_cell\_down | Fyn, Ifih1, Itk, Lck, Prkch, Prkcq, Stk39, Tnik, Txk, Zap70 |
| GO:0016740 | transferase activity | 1.40e-02 | 7.27e-02 | 11 | 47 | 1681 | 14853 | 23.40% | 11.32% | 0.65% | 0.32% | mature\_B\_cell\_down | Art2b, Fyn, Itk, Lck, Prkch, Prkcq, Stk39, Tnik, Txk, Zap70, Zdhhc15 |
| GO:0008013 | beta-catenin binding | 1.50e-02 | 7.44e-02 | 2 | 47 | 59 | 14853 | 4.26% | 0.40% | 3.39% | 0.32% | mature\_B\_cell\_down | Slc9a3r1, Tcf7 |
| GO:0017160 | Ral GTPase binding | 1.57e-02 | 7.44e-02 | 1 | 47 | 5 | 14853 | 2.13% | 0.03% | 20.00% | 0.32% | mature\_B\_cell\_down | Prkch |
| GO:0005134 | interleukin-2 receptor binding | 1.57e-02 | 7.44e-02 | 1 | 47 | 5 | 14853 | 2.13% | 0.03% | 20.00% | 0.32% | mature\_B\_cell\_down | Gata3 |
| GO:0005031 | tumor necrosis factor-activated receptor activity | 1.57e-02 | 7.44e-02 | 1 | 47 | 5 | 14853 | 2.13% | 0.03% | 20.00% | 0.32% | mature\_B\_cell\_down | Tnfrsf1a |
| GO:0000166 | nucleotide binding | 1.75e-02 | 7.80e-02 | 13 | 47 | 2217 | 14853 | 27.66% | 14.93% | 0.59% | 0.32% | mature\_B\_cell\_down | Fyn, Hnrpll, Ifih1, Itk, Lck, Prkch, Prkcq, Rab37, Sept9, Stk39, Tnik, Txk, Zap70 |
| GO:0044212 | transcription regulatory region DNA binding | 1.82e-02 | 7.80e-02 | 4 | 47 | 320 | 14853 | 8.51% | 2.15% | 1.25% | 0.32% | mature\_B\_cell\_down | Gata3, Notch1, Tcf7, Txk |
| GO:0031681 | G-protein beta-subunit binding | 1.88e-02 | 7.80e-02 | 1 | 47 | 6 | 14853 | 2.13% | 0.04% | 16.67% | 0.32% | mature\_B\_cell\_down | F2r |
| GO:0003956 | NAD(P)+-protein-arginine ADP-ribosyltransferase activity | 1.88e-02 | 7.80e-02 | 1 | 47 | 6 | 14853 | 2.13% | 0.04% | 16.67% | 0.32% | mature\_B\_cell\_down | Art2b |
| GO:0005035 | death receptor activity | 1.88e-02 | 7.80e-02 | 1 | 47 | 6 | 14853 | 2.13% | 0.04% | 16.67% | 0.32% | mature\_B\_cell\_down | Tnfrsf1a |
| GO:0001948 | glycoprotein binding | 1.96e-02 | 7.80e-02 | 2 | 47 | 68 | 14853 | 4.26% | 0.46% | 2.94% | 0.32% | mature\_B\_cell\_down | Fyn, Lck |
| GO:0005178 | integrin binding | 1.96e-02 | 7.80e-02 | 2 | 47 | 68 | 14853 | 4.26% | 0.46% | 2.94% | 0.32% | mature\_B\_cell\_down | Cd226, Thy1 |
| GO:0000975 | regulatory region DNA binding | 1.98e-02 | 7.80e-02 | 4 | 47 | 328 | 14853 | 8.51% | 2.21% | 1.22% | 0.32% | mature\_B\_cell\_down | Gata3, Notch1, Tcf7, Txk |
| GO:0001067 | regulatory region nucleic acid binding | 1.98e-02 | 7.80e-02 | 4 | 47 | 328 | 14853 | 8.51% | 2.21% | 1.22% | 0.32% | mature\_B\_cell\_down | Gata3, Notch1, Tcf7, Txk |
| GO:0015377 | cation:chloride symporter activity | 2.19e-02 | 8.35e-02 | 1 | 47 | 7 | 14853 | 2.13% | 0.05% | 14.29% | 0.32% | mature\_B\_cell\_down | Slc12a7 |
| GO:0032795 | heterotrimeric G-protein binding | 2.19e-02 | 8.35e-02 | 1 | 47 | 7 | 14853 | 2.13% | 0.05% | 14.29% | 0.32% | mature\_B\_cell\_down | F2r |
| GO:0030695 | GTPase regulator activity | 2.46e-02 | 9.20e-02 | 4 | 47 | 351 | 14853 | 8.51% | 2.36% | 1.14% | 0.32% | mature\_B\_cell\_down | Rinl, Tbc1d2b, Thy1, Tnik |
| GO:0005096 | GTPase activator activity | 2.63e-02 | 9.64e-02 | 3 | 47 | 203 | 14853 | 6.38% | 1.37% | 1.48% | 0.32% | mature\_B\_cell\_down | Rinl, Tbc1d2b, Thy1 |
| GO:0060589 | nucleoside-triphosphatase regulator activity | 2.74e-02 | 9.79e-02 | 4 | 47 | 363 | 14853 | 8.51% | 2.44% | 1.10% | 0.32% | mature\_B\_cell\_down | Rinl, Tbc1d2b, Thy1, Tnik |
| GO:0005102 | receptor binding | 2.76e-02 | 9.79e-02 | 8 | 47 | 1159 | 14853 | 17.02% | 7.80% | 0.69% | 0.32% | mature\_B\_cell\_down | Cd226, F2r, Fyn, Gata3, Lck, Lpin1, Slc9a3r1, Thy1 |
| GO:0005099 | Ras GTPase activator activity | 2.84e-02 | 9.90e-02 | 2 | 47 | 83 | 14853 | 4.26% | 0.56% | 2.41% | 0.32% | mature\_B\_cell\_down | Tbc1d2b, Thy1 |
| GO:0001012 | RNA polymerase II regulatory region DNA binding | 2.96e-02 | 1.02e-01 | 2 | 47 | 85 | 14853 | 4.26% | 0.57% | 2.35% | 0.32% | mature\_B\_cell\_down | Gata3, Txk |
| GO:0036094 | small molecule binding | 3.11e-02 | 1.05e-01 | 13 | 47 | 2392 | 14853 | 27.66% | 16.10% | 0.54% | 0.32% | mature\_B\_cell\_down | Fyn, Hnrpll, Ifih1, Itk, Lck, Prkch, Prkcq, Rab37, Sept9, Stk39, Tnik, Txk, Zap70 |
| GO:0005083 | small GTPase regulator activity | 3.15e-02 | 1.05e-01 | 3 | 47 | 218 | 14853 | 6.38% | 1.47% | 1.38% | 0.32% | mature\_B\_cell\_down | Tbc1d2b, Thy1, Tnik |
| GO:0005488 | binding | 3.28e-02 | 1.08e-01 | 40 | 47 | 10773 | 14853 | 85.11% | 72.53% | 0.37% | 0.32% | mature\_B\_cell\_down | Btbd11, Cd226, Cd247, Cd27, Cd3g, Ctsd, F2r, Fyb, Fyn, Gata3, Hnrpll, Hpcal1, Ifih1, Ipcef1, Itk, Lat, Lck, Lpin1, Notch1, Prkch, Prkcq, Rab37, Rinl, Rnf125, Selplg, Sept9, Sh2d2a, Skap1, Sla2, Slc9a3r1, Stk39, Sytl2, Tbc1d2b, Tcf7, Thy1, Tnfrsf1a, Tnik, Txk, Zap70, Zdhhc15 |
| GO:0042043 | neurexin family protein binding | 3.43e-02 | 1.09e-01 | 1 | 47 | 11 | 14853 | 2.13% | 0.07% | 9.09% | 0.32% | mature\_B\_cell\_down | Sytl2 |
| GO:0008195 | phosphatidate phosphatase activity | 3.43e-02 | 1.09e-01 | 1 | 47 | 11 | 14853 | 2.13% | 0.07% | 9.09% | 0.32% | mature\_B\_cell\_down | Lpin1 |
| GO:0004674 | protein serine/threonine kinase activity | 3.99e-02 | 1.23e-01 | 4 | 47 | 409 | 14853 | 8.51% | 2.75% | 0.98% | 0.32% | mature\_B\_cell\_down | Prkch, Prkcq, Stk39, Tnik |
| GO:0050780 | dopamine receptor binding | 4.04e-02 | 1.23e-01 | 1 | 47 | 13 | 14853 | 2.13% | 0.09% | 7.69% | 0.32% | mature\_B\_cell\_down | Slc9a3r1 |
| GO:0042975 | peroxisome proliferator activated receptor binding | 4.04e-02 | 1.23e-01 | 1 | 47 | 13 | 14853 | 2.13% | 0.09% | 7.69% | 0.32% | mature\_B\_cell\_down | Lpin1 |
| GO:0016791 | phosphatase activity | 4.10e-02 | 1.23e-01 | 3 | 47 | 242 | 14853 | 6.38% | 1.63% | 1.24% | 0.32% | mature\_B\_cell\_down | Lck, Lpin1, Ppm1h |
| GO:0031690 | adrenergic receptor binding | 4.34e-02 | 1.27e-01 | 1 | 47 | 14 | 14853 | 2.13% | 0.09% | 7.14% | 0.32% | mature\_B\_cell\_down | Slc9a3r1 |
| GO:0001786 | phosphatidylserine binding | 4.34e-02 | 1.27e-01 | 1 | 47 | 14 | 14853 | 2.13% | 0.09% | 7.14% | 0.32% | mature\_B\_cell\_down | Sytl2 |
| GO:0070851 | growth factor receptor binding | 4.51e-02 | 1.30e-01 | 2 | 47 | 107 | 14853 | 4.26% | 0.72% | 1.87% | 0.32% | mature\_B\_cell\_down | Gata3, Slc9a3r1 |
| GO:0030234 | enzyme regulator activity | 4.56e-02 | 1.30e-01 | 6 | 47 | 830 | 14853 | 12.77% | 5.59% | 0.72% | 0.32% | mature\_B\_cell\_down | Cd27, Notch1, Rinl, Tbc1d2b, Thy1, Tnik |
| GO:0017016 | Ras GTPase binding | 4.89e-02 | 1.37e-01 | 2 | 47 | 112 | 14853 | 4.26% | 0.75% | 1.79% | 0.32% | mature\_B\_cell\_down | Prkch, Sytl2 |
| GO:0019902 | phosphatase binding | 5.21e-02 | 1.43e-01 | 2 | 47 | 116 | 14853 | 4.26% | 0.78% | 1.72% | 0.32% | mature\_B\_cell\_down | Slc9a3r1, Sytl2 |
| GO:0001784 | phosphotyrosine binding | 5.25e-02 | 1.43e-01 | 1 | 47 | 17 | 14853 | 2.13% | 0.11% | 5.88% | 0.32% | mature\_B\_cell\_down | Zap70 |
| GO:0016409 | palmitoyltransferase activity | 5.55e-02 | 1.48e-01 | 1 | 47 | 18 | 14853 | 2.13% | 0.12% | 5.56% | 0.32% | mature\_B\_cell\_down | Zdhhc15 |
| GO:0051428 | peptide hormone receptor binding | 5.55e-02 | 1.48e-01 | 1 | 47 | 18 | 14853 | 2.13% | 0.12% | 5.56% | 0.32% | mature\_B\_cell\_down | Fyn |
| GO:0031267 | small GTPase binding | 5.61e-02 | 1.48e-01 | 2 | 47 | 121 | 14853 | 4.26% | 0.81% | 1.65% | 0.32% | mature\_B\_cell\_down | Prkch, Sytl2 |
| GO:0071837 | HMG box domain binding | 6.15e-02 | 1.60e-01 | 1 | 47 | 20 | 14853 | 2.13% | 0.13% | 5.00% | 0.32% | mature\_B\_cell\_down | Gata3 |
| GO:0001965 | G-protein alpha-subunit binding | 6.44e-02 | 1.64e-01 | 1 | 47 | 21 | 14853 | 2.13% | 0.14% | 4.76% | 0.32% | mature\_B\_cell\_down | F2r |
| GO:0035091 | phosphatidylinositol binding | 6.45e-02 | 1.64e-01 | 2 | 47 | 131 | 14853 | 4.26% | 0.88% | 1.53% | 0.32% | mature\_B\_cell\_down | Sytl2, Thy1 |
| GO:0015296 | anion:cation symporter activity | 6.74e-02 | 1.69e-01 | 1 | 47 | 22 | 14853 | 2.13% | 0.15% | 4.55% | 0.32% | mature\_B\_cell\_down | Slc12a7 |
| GO:0051020 | GTPase binding | 7.15e-02 | 1.71e-01 | 2 | 47 | 139 | 14853 | 4.26% | 0.94% | 1.44% | 0.32% | mature\_B\_cell\_down | Prkch, Sytl2 |
| GO:0070888 | E-box binding | 7.33e-02 | 1.71e-01 | 1 | 47 | 24 | 14853 | 2.13% | 0.16% | 4.17% | 0.32% | mature\_B\_cell\_down | Gata3 |
| GO:0043027 | cysteine-type endopeptidase inhibitor activity involved in apoptotic process | 7.33e-02 | 1.71e-01 | 1 | 47 | 24 | 14853 | 2.13% | 0.16% | 4.17% | 0.32% | mature\_B\_cell\_down | Cd27 |
| GO:0044325 | ion channel binding | 7.33e-02 | 1.71e-01 | 1 | 47 | 24 | 14853 | 2.13% | 0.16% | 4.17% | 0.32% | mature\_B\_cell\_down | Fyn |
| GO:0051117 | ATPase binding | 7.62e-02 | 1.71e-01 | 1 | 47 | 25 | 14853 | 2.13% | 0.17% | 4.00% | 0.32% | mature\_B\_cell\_down | Lck |
| GO:0000979 | RNA polymerase II core promoter sequence-specific DNA binding | 7.62e-02 | 1.71e-01 | 1 | 47 | 25 | 14853 | 2.13% | 0.17% | 4.00% | 0.32% | mature\_B\_cell\_down | Gata3 |
| GO:0004190 | aspartic-type endopeptidase activity | 7.62e-02 | 1.71e-01 | 1 | 47 | 25 | 14853 | 2.13% | 0.17% | 4.00% | 0.32% | mature\_B\_cell\_down | Ctsd |
| GO:0070001 | aspartic-type peptidase activity | 7.62e-02 | 1.71e-01 | 1 | 47 | 25 | 14853 | 2.13% | 0.17% | 4.00% | 0.32% | mature\_B\_cell\_down | Ctsd |
| GO:0001158 | enhancer sequence-specific DNA binding | 7.62e-02 | 1.71e-01 | 1 | 47 | 25 | 14853 | 2.13% | 0.17% | 4.00% | 0.32% | mature\_B\_cell\_down | Gata3 |
| GO:0045309 | protein phosphorylated amino acid binding | 7.62e-02 | 1.71e-01 | 1 | 47 | 25 | 14853 | 2.13% | 0.17% | 4.00% | 0.32% | mature\_B\_cell\_down | Zap70 |
| GO:0005546 | phosphatidylinositol-4,5-bisphosphate binding | 7.92e-02 | 1.76e-01 | 1 | 47 | 26 | 14853 | 2.13% | 0.18% | 3.85% | 0.32% | mature\_B\_cell\_down | Sytl2 |
| GO:0008047 | enzyme activator activity | 8.33e-02 | 1.79e-01 | 3 | 47 | 325 | 14853 | 6.38% | 2.19% | 0.92% | 0.32% | mature\_B\_cell\_down | Rinl, Tbc1d2b, Thy1 |
| GO:0042578 | phosphoric ester hydrolase activity | 8.33e-02 | 1.79e-01 | 3 | 47 | 325 | 14853 | 6.38% | 2.19% | 0.92% | 0.32% | mature\_B\_cell\_down | Lck, Lpin1, Ppm1h |
| GO:0005100 | Rho GTPase activator activity | 8.50e-02 | 1.79e-01 | 1 | 47 | 28 | 14853 | 2.13% | 0.19% | 3.57% | 0.32% | mature\_B\_cell\_down | Thy1 |
| GO:0035326 | enhancer binding | 8.50e-02 | 1.79e-01 | 1 | 47 | 28 | 14853 | 2.13% | 0.19% | 3.57% | 0.32% | mature\_B\_cell\_down | Gata3 |
| GO:0046875 | ephrin receptor binding | 8.50e-02 | 1.79e-01 | 1 | 47 | 28 | 14853 | 2.13% | 0.19% | 3.57% | 0.32% | mature\_B\_cell\_down | Fyn |
| GO:0004721 | phosphoprotein phosphatase activity | 8.72e-02 | 1.82e-01 | 2 | 47 | 156 | 14853 | 4.26% | 1.05% | 1.28% | 0.32% | mature\_B\_cell\_down | Lck, Ppm1h |
| GO:0001078 | RNA polymerase II core promoter proximal region sequence-specific DNA binding transcription factor activity involved in negative regulation of transcription | 9.37e-02 | 1.90e-01 | 1 | 47 | 31 | 14853 | 2.13% | 0.21% | 3.23% | 0.32% | mature\_B\_cell\_down | Gata3 |
| GO:0001227 | RNA polymerase II transcription regulatory region sequence-specific DNA binding transcription factor activity involved in negative regulation of transcription | 9.37e-02 | 1.90e-01 | 1 | 47 | 31 | 14853 | 2.13% | 0.21% | 3.23% | 0.32% | mature\_B\_cell\_down | Gata3 |
| GO:0031490 | chromatin DNA binding | 9.37e-02 | 1.90e-01 | 1 | 47 | 31 | 14853 | 2.13% | 0.21% | 3.23% | 0.32% | mature\_B\_cell\_down | Notch1 |
| GO:0001046 | core promoter sequence-specific DNA binding | 1.08e-01 | 2.17e-01 | 1 | 47 | 36 | 14853 | 2.13% | 0.24% | 2.78% | 0.32% | mature\_B\_cell\_down | Gata3 |
| GO:0043028 | cysteine-type endopeptidase regulator activity involved in apoptotic process | 1.11e-01 | 2.21e-01 | 1 | 47 | 37 | 14853 | 2.13% | 0.25% | 2.70% | 0.32% | mature\_B\_cell\_down | Cd27 |
| GO:0000987 | core promoter proximal region sequence-specific DNA binding | 1.14e-01 | 2.23e-01 | 1 | 47 | 38 | 14853 | 2.13% | 0.26% | 2.63% | 0.32% | mature\_B\_cell\_down | Gata3 |
| GO:0003713 | transcription coactivator activity | 1.16e-01 | 2.23e-01 | 2 | 47 | 185 | 14853 | 4.26% | 1.25% | 1.08% | 0.32% | mature\_B\_cell\_down | Gata3, Lpin1 |
| GO:0005097 | Rab GTPase activator activity | 1.16e-01 | 2.23e-01 | 1 | 47 | 39 | 14853 | 2.13% | 0.26% | 2.56% | 0.32% | mature\_B\_cell\_down | Tbc1d2b |
| GO:0043621 | protein self-association | 1.16e-01 | 2.23e-01 | 1 | 47 | 39 | 14853 | 2.13% | 0.26% | 2.56% | 0.32% | mature\_B\_cell\_down | Slc9a3r1 |
| GO:0001159 | core promoter proximal region DNA binding | 1.22e-01 | 2.30e-01 | 1 | 47 | 41 | 14853 | 2.13% | 0.28% | 2.44% | 0.32% | mature\_B\_cell\_down | Gata3 |
| GO:0016763 | transferase activity, transferring pentosyl groups | 1.22e-01 | 2.30e-01 | 1 | 47 | 41 | 14853 | 2.13% | 0.28% | 2.44% | 0.32% | mature\_B\_cell\_down | Art2b |
| GO:0003727 | single-stranded RNA binding | 1.25e-01 | 2.33e-01 | 1 | 47 | 42 | 14853 | 2.13% | 0.28% | 2.38% | 0.32% | mature\_B\_cell\_down | Ifih1 |
| GO:0004722 | protein serine/threonine phosphatase activity | 1.28e-01 | 2.36e-01 | 1 | 47 | 43 | 14853 | 2.13% | 0.29% | 2.33% | 0.32% | mature\_B\_cell\_down | Lck |
| GO:0017137 | Rab GTPase binding | 1.30e-01 | 2.39e-01 | 1 | 47 | 44 | 14853 | 2.13% | 0.30% | 2.27% | 0.32% | mature\_B\_cell\_down | Sytl2 |
| GO:0003725 | double-stranded RNA binding | 1.41e-01 | 2.57e-01 | 1 | 47 | 48 | 14853 | 2.13% | 0.32% | 2.08% | 0.32% | mature\_B\_cell\_down | Ifih1 |
| GO:0004869 | cysteine-type endopeptidase inhibitor activity | 1.60e-01 | 2.89e-01 | 1 | 47 | 55 | 14853 | 2.13% | 0.37% | 1.82% | 0.32% | mature\_B\_cell\_down | Cd27 |
| GO:0050839 | cell adhesion molecule binding | 1.63e-01 | 2.89e-01 | 1 | 47 | 56 | 14853 | 2.13% | 0.38% | 1.79% | 0.32% | mature\_B\_cell\_down | Cd226 |
| GO:0043021 | ribonucleoprotein complex binding | 1.63e-01 | 2.89e-01 | 1 | 47 | 56 | 14853 | 2.13% | 0.38% | 1.79% | 0.32% | mature\_B\_cell\_down | Ifih1 |
| GO:0019955 | cytokine binding | 1.66e-01 | 2.91e-01 | 1 | 47 | 57 | 14853 | 2.13% | 0.38% | 1.75% | 0.32% | mature\_B\_cell\_down | Tnfrsf1a |
| GO:0016247 | channel regulator activity | 1.68e-01 | 2.94e-01 | 1 | 47 | 58 | 14853 | 2.13% | 0.39% | 1.72% | 0.32% | mature\_B\_cell\_down | Slc9a3r1 |
| GO:0051219 | phosphoprotein binding | 1.71e-01 | 2.96e-01 | 1 | 47 | 59 | 14853 | 2.13% | 0.40% | 1.69% | 0.32% | mature\_B\_cell\_down | Zap70 |
| GO:0001085 | RNA polymerase II transcription factor binding | 1.76e-01 | 3.03e-01 | 1 | 47 | 61 | 14853 | 2.13% | 0.41% | 1.64% | 0.32% | mature\_B\_cell\_down | Lpin1 |
| GO:0042826 | histone deacetylase binding | 1.84e-01 | 3.11e-01 | 1 | 47 | 64 | 14853 | 2.13% | 0.43% | 1.56% | 0.32% | mature\_B\_cell\_down | Lpin1 |
| GO:0002020 | protease binding | 1.84e-01 | 3.11e-01 | 1 | 47 | 64 | 14853 | 2.13% | 0.43% | 1.56% | 0.32% | mature\_B\_cell\_down | Tnfrsf1a |
| GO:0015294 | solute:cation symporter activity | 1.89e-01 | 3.17e-01 | 1 | 47 | 66 | 14853 | 2.13% | 0.44% | 1.52% | 0.32% | mature\_B\_cell\_down | Slc12a7 |
| GO:0001077 | RNA polymerase II core promoter proximal region sequence-specific DNA binding transcription factor activity involved in positive regulation of transcription | 2.04e-01 | 3.40e-01 | 1 | 47 | 72 | 14853 | 2.13% | 0.48% | 1.39% | 0.32% | mature\_B\_cell\_down | Gata3 |
| GO:0001228 | RNA polymerase II transcription regulatory region sequence-specific DNA binding transcription factor activity involved in positive regulation of transcription | 2.07e-01 | 3.42e-01 | 1 | 47 | 73 | 14853 | 2.13% | 0.49% | 1.37% | 0.32% | mature\_B\_cell\_down | Gata3 |
| GO:0005057 | receptor signaling protein activity | 2.15e-01 | 3.52e-01 | 1 | 47 | 76 | 14853 | 2.13% | 0.51% | 1.32% | 0.32% | mature\_B\_cell\_down | Lat |
| GO:0000977 | RNA polymerase II regulatory region sequence-specific DNA binding | 2.17e-01 | 3.53e-01 | 1 | 47 | 77 | 14853 | 2.13% | 0.52% | 1.30% | 0.32% | mature\_B\_cell\_down | Gata3 |
| GO:0003682 | chromatin binding | 2.20e-01 | 3.54e-01 | 2 | 47 | 278 | 14853 | 4.26% | 1.87% | 0.72% | 0.32% | mature\_B\_cell\_down | Notch1, Tcf7 |
| GO:0015108 | chloride transmembrane transporter activity | 2.22e-01 | 3.56e-01 | 1 | 47 | 79 | 14853 | 2.13% | 0.53% | 1.27% | 0.32% | mature\_B\_cell\_down | Slc12a7 |
| GO:0003824 | catalytic activity | 2.31e-01 | 3.67e-01 | 19 | 47 | 5097 | 14853 | 40.43% | 34.32% | 0.37% | 0.32% | mature\_B\_cell\_down | Art2b, Ctsd, Ctsw, Enpp4, Fyn, Ifih1, Itk, Lck, Lpin1, Ppm1h, Prkch, Prkcq, Rab37, Rnf125, Stk39, Tnik, Txk, Zap70, Zdhhc15 |
| GO:0004857 | enzyme inhibitor activity | 2.44e-01 | 3.85e-01 | 2 | 47 | 299 | 14853 | 4.26% | 2.01% | 0.67% | 0.32% | mature\_B\_cell\_down | Cd27, Notch1 |
| GO:0047485 | protein N-terminus binding | 2.61e-01 | 4.08e-01 | 1 | 47 | 95 | 14853 | 2.13% | 0.64% | 1.05% | 0.32% | mature\_B\_cell\_down | Sla2 |
| GO:0003712 | transcription cofactor activity | 2.69e-01 | 4.14e-01 | 2 | 47 | 320 | 14853 | 4.26% | 2.15% | 0.62% | 0.32% | mature\_B\_cell\_down | Gata3, Lpin1 |
| GO:0000982 | RNA polymerase II core promoter proximal region sequence-specific DNA binding transcription factor activity | 2.70e-01 | 4.14e-01 | 1 | 47 | 99 | 14853 | 2.13% | 0.67% | 1.01% | 0.32% | mature\_B\_cell\_down | Gata3 |
| GO:0015103 | inorganic anion transmembrane transporter activity | 2.70e-01 | 4.14e-01 | 1 | 47 | 99 | 14853 | 2.13% | 0.67% | 1.01% | 0.32% | mature\_B\_cell\_down | Slc12a7 |
| GO:0003729 | mRNA binding | 2.75e-01 | 4.18e-01 | 1 | 47 | 101 | 14853 | 2.13% | 0.68% | 0.99% | 0.32% | mature\_B\_cell\_down | Hnrpll |
| GO:0030165 | PDZ domain binding | 2.77e-01 | 4.18e-01 | 1 | 47 | 102 | 14853 | 2.13% | 0.69% | 0.98% | 0.32% | mature\_B\_cell\_down | Slc9a3r1 |
| GO:0000989 | transcription factor binding transcription factor activity | 2.90e-01 | 4.35e-01 | 2 | 47 | 338 | 14853 | 4.26% | 2.28% | 0.59% | 0.32% | mature\_B\_cell\_down | Gata3, Lpin1 |
| GO:0005525 | GTP binding | 2.96e-01 | 4.40e-01 | 2 | 47 | 343 | 14853 | 4.26% | 2.31% | 0.58% | 0.32% | mature\_B\_cell\_down | Rab37, Sept9 |
| GO:0008528 | G-protein coupled peptide receptor activity | 2.98e-01 | 4.40e-01 | 1 | 47 | 111 | 14853 | 2.13% | 0.75% | 0.90% | 0.32% | mature\_B\_cell\_down | F2r |
| GO:0001653 | peptide receptor activity | 3.02e-01 | 4.44e-01 | 1 | 47 | 113 | 14853 | 2.13% | 0.76% | 0.88% | 0.32% | mature\_B\_cell\_down | F2r |
| GO:0000988 | protein binding transcription factor activity | 3.05e-01 | 4.45e-01 | 2 | 47 | 351 | 14853 | 4.26% | 2.36% | 0.57% | 0.32% | mature\_B\_cell\_down | Gata3, Lpin1 |
| GO:0019001 | guanyl nucleotide binding | 3.14e-01 | 4.45e-01 | 2 | 47 | 358 | 14853 | 4.26% | 2.41% | 0.56% | 0.32% | mature\_B\_cell\_down | Rab37, Sept9 |
| GO:0032561 | guanyl ribonucleotide binding | 3.14e-01 | 4.45e-01 | 2 | 47 | 358 | 14853 | 4.26% | 2.41% | 0.56% | 0.32% | mature\_B\_cell\_down | Rab37, Sept9 |
| GO:0015293 | symporter activity | 3.15e-01 | 4.45e-01 | 1 | 47 | 119 | 14853 | 2.13% | 0.80% | 0.84% | 0.32% | mature\_B\_cell\_down | Slc12a7 |
| GO:0043565 | sequence-specific DNA binding | 3.16e-01 | 4.45e-01 | 3 | 47 | 624 | 14853 | 6.38% | 4.20% | 0.48% | 0.32% | mature\_B\_cell\_down | Gata3, Notch1, Tcf7 |
| GO:0017124 | SH3 domain binding | 3.17e-01 | 4.45e-01 | 1 | 47 | 120 | 14853 | 2.13% | 0.81% | 0.83% | 0.32% | mature\_B\_cell\_down | Sh2d2a |
| GO:0051427 | hormone receptor binding | 3.17e-01 | 4.45e-01 | 1 | 47 | 120 | 14853 | 2.13% | 0.81% | 0.83% | 0.32% | mature\_B\_cell\_down | Fyn |
| GO:0000976 | transcription regulatory region sequence-specific DNA binding | 3.45e-01 | 4.74e-01 | 1 | 47 | 133 | 14853 | 2.13% | 0.90% | 0.75% | 0.32% | mature\_B\_cell\_down | Gata3 |
| GO:0008509 | anion transmembrane transporter activity | 3.47e-01 | 4.74e-01 | 1 | 47 | 134 | 14853 | 2.13% | 0.90% | 0.75% | 0.32% | mature\_B\_cell\_down | Slc12a7 |
| GO:0008234 | cysteine-type peptidase activity | 3.47e-01 | 4.74e-01 | 1 | 47 | 134 | 14853 | 2.13% | 0.90% | 0.75% | 0.32% | mature\_B\_cell\_down | Ctsw |
| GO:0004386 | helicase activity | 3.47e-01 | 4.74e-01 | 1 | 47 | 134 | 14853 | 2.13% | 0.90% | 0.75% | 0.32% | mature\_B\_cell\_down | Ifih1 |
| GO:0005085 | guanyl-nucleotide exchange factor activity | 3.62e-01 | 4.91e-01 | 1 | 47 | 141 | 14853 | 2.13% | 0.95% | 0.71% | 0.32% | mature\_B\_cell\_down | Rinl |
| GO:0016747 | transferase activity, transferring acyl groups other than amino-acyl groups | 3.66e-01 | 4.93e-01 | 1 | 47 | 143 | 14853 | 2.13% | 0.96% | 0.70% | 0.32% | mature\_B\_cell\_down | Zdhhc15 |
| GO:0008134 | transcription factor binding | 3.70e-01 | 4.96e-01 | 2 | 47 | 407 | 14853 | 4.26% | 2.74% | 0.49% | 0.32% | mature\_B\_cell\_down | Gata3, Lpin1 |
| GO:0015631 | tubulin binding | 3.78e-01 | 5.01e-01 | 1 | 47 | 149 | 14853 | 2.13% | 1.00% | 0.67% | 0.32% | mature\_B\_cell\_down | Fyn |
| GO:0016788 | hydrolase activity, acting on ester bonds | 3.78e-01 | 5.01e-01 | 3 | 47 | 695 | 14853 | 6.38% | 4.68% | 0.43% | 0.32% | mature\_B\_cell\_down | Lck, Lpin1, Ppm1h |
| GO:0042277 | peptide binding | 3.88e-01 | 5.10e-01 | 1 | 47 | 154 | 14853 | 2.13% | 1.04% | 0.65% | 0.32% | mature\_B\_cell\_down | Ctsd |
| GO:0016787 | hydrolase activity | 3.95e-01 | 5.16e-01 | 8 | 47 | 2201 | 14853 | 17.02% | 14.82% | 0.36% | 0.32% | mature\_B\_cell\_down | Ctsd, Ctsw, Enpp4, Ifih1, Lck, Lpin1, Ppm1h, Rab37 |
| GO:0003924 | GTPase activity | 4.26e-01 | 5.43e-01 | 1 | 47 | 174 | 14853 | 2.13% | 1.17% | 0.57% | 0.32% | mature\_B\_cell\_down | Rab37 |
| GO:0004866 | endopeptidase inhibitor activity | 4.28e-01 | 5.43e-01 | 1 | 47 | 175 | 14853 | 2.13% | 1.18% | 0.57% | 0.32% | mature\_B\_cell\_down | Cd27 |
| GO:0008022 | protein C-terminus binding | 4.28e-01 | 5.43e-01 | 1 | 47 | 175 | 14853 | 2.13% | 1.18% | 0.57% | 0.32% | mature\_B\_cell\_down | Lck |
| GO:0015291 | secondary active transmembrane transporter activity | 4.28e-01 | 5.43e-01 | 1 | 47 | 175 | 14853 | 2.13% | 1.18% | 0.57% | 0.32% | mature\_B\_cell\_down | Slc12a7 |
| GO:0003700 | sequence-specific DNA binding transcription factor activity | 4.29e-01 | 5.43e-01 | 3 | 47 | 754 | 14853 | 6.38% | 5.08% | 0.40% | 0.32% | mature\_B\_cell\_down | Gata3, Notch1, Tcf7 |
| GO:0001071 | nucleic acid binding transcription factor activity | 4.31e-01 | 5.43e-01 | 3 | 47 | 756 | 14853 | 6.38% | 5.09% | 0.40% | 0.32% | mature\_B\_cell\_down | Gata3, Notch1, Tcf7 |
| GO:0061135 | endopeptidase regulator activity | 4.39e-01 | 5.49e-01 | 1 | 47 | 181 | 14853 | 2.13% | 1.22% | 0.55% | 0.32% | mature\_B\_cell\_down | Cd27 |
| GO:0030414 | peptidase inhibitor activity | 4.42e-01 | 5.51e-01 | 1 | 47 | 183 | 14853 | 2.13% | 1.23% | 0.55% | 0.32% | mature\_B\_cell\_down | Cd27 |
| GO:0016746 | transferase activity, transferring acyl groups | 4.60e-01 | 5.69e-01 | 1 | 47 | 193 | 14853 | 2.13% | 1.30% | 0.52% | 0.32% | mature\_B\_cell\_down | Zdhhc15 |
| GO:0001664 | G-protein coupled receptor binding | 4.67e-01 | 5.71e-01 | 1 | 47 | 197 | 14853 | 2.13% | 1.33% | 0.51% | 0.32% | mature\_B\_cell\_down | Slc9a3r1 |
| GO:0005126 | cytokine receptor binding | 4.67e-01 | 5.71e-01 | 1 | 47 | 197 | 14853 | 2.13% | 1.33% | 0.51% | 0.32% | mature\_B\_cell\_down | Gata3 |
| GO:0005509 | calcium ion binding | 4.92e-01 | 5.95e-01 | 2 | 47 | 518 | 14853 | 4.26% | 3.49% | 0.39% | 0.32% | mature\_B\_cell\_down | Hpcal1, Notch1 |
| GO:0061134 | peptidase regulator activity | 4.92e-01 | 5.95e-01 | 1 | 47 | 212 | 14853 | 2.13% | 1.43% | 0.47% | 0.32% | mature\_B\_cell\_down | Cd27 |
| GO:0003677 | DNA binding | 4.97e-01 | 5.98e-01 | 6 | 47 | 1774 | 14853 | 12.77% | 11.94% | 0.34% | 0.32% | mature\_B\_cell\_down | Btbd11, Gata3, Ifih1, Notch1, Tcf7, Txk |
| GO:0043566 | structure-specific DNA binding | 5.11e-01 | 6.11e-01 | 1 | 47 | 224 | 14853 | 2.13% | 1.51% | 0.45% | 0.32% | mature\_B\_cell\_down | Notch1 |
| GO:0000981 | sequence-specific DNA binding RNA polymerase II transcription factor activity | 5.29e-01 | 6.30e-01 | 1 | 47 | 236 | 14853 | 2.13% | 1.59% | 0.42% | 0.32% | mature\_B\_cell\_down | Gata3 |
| GO:0016757 | transferase activity, transferring glycosyl groups | 5.39e-01 | 6.37e-01 | 1 | 47 | 242 | 14853 | 2.13% | 1.63% | 0.41% | 0.32% | mature\_B\_cell\_down | Art2b |
| GO:0070011 | peptidase activity, acting on L-amino acid peptides | 5.43e-01 | 6.39e-01 | 2 | 47 | 570 | 14853 | 4.26% | 3.84% | 0.35% | 0.32% | mature\_B\_cell\_down | Ctsd, Ctsw |
| GO:0008233 | peptidase activity | 5.71e-01 | 6.68e-01 | 2 | 47 | 599 | 14853 | 4.26% | 4.03% | 0.33% | 0.32% | mature\_B\_cell\_down | Ctsd, Ctsw |
| GO:0017111 | nucleoside-triphosphatase activity | 5.88e-01 | 6.84e-01 | 2 | 47 | 618 | 14853 | 4.26% | 4.16% | 0.32% | 0.32% | mature\_B\_cell\_down | Ifih1, Rab37 |
| GO:0016462 | pyrophosphatase activity | 6.18e-01 | 7.08e-01 | 2 | 47 | 652 | 14853 | 4.26% | 4.39% | 0.31% | 0.32% | mature\_B\_cell\_down | Ifih1, Rab37 |
| GO:0016818 | hydrolase activity, acting on acid anhydrides, in phosphorus-containing anhydrides | 6.20e-01 | 7.08e-01 | 2 | 47 | 655 | 14853 | 4.26% | 4.41% | 0.31% | 0.32% | mature\_B\_cell\_down | Ifih1, Rab37 |
| GO:0016817 | hydrolase activity, acting on acid anhydrides | 6.21e-01 | 7.08e-01 | 2 | 47 | 656 | 14853 | 4.26% | 4.42% | 0.30% | 0.32% | mature\_B\_cell\_down | Ifih1, Rab37 |
| GO:0008270 | zinc ion binding | 6.21e-01 | 7.08e-01 | 4 | 47 | 1337 | 14853 | 8.51% | 9.00% | 0.30% | 0.32% | mature\_B\_cell\_down | Gata3, Ifih1, Rnf125, Zdhhc15 |
| GO:0022804 | active transmembrane transporter activity | 6.28e-01 | 7.12e-01 | 1 | 47 | 309 | 14853 | 2.13% | 2.08% | 0.32% | 0.32% | mature\_B\_cell\_down | Slc12a7 |
| GO:0004872 | receptor activity | 6.35e-01 | 7.15e-01 | 8 | 47 | 2687 | 14853 | 17.02% | 18.09% | 0.30% | 0.32% | mature\_B\_cell\_down | Cd226, Cd247, Cd27, Cd3g, Cd7, F2r, Notch1, Tnfrsf1a |
| GO:0046982 | protein heterodimerization activity | 6.56e-01 | 7.36e-01 | 1 | 47 | 333 | 14853 | 2.13% | 2.24% | 0.30% | 0.32% | mature\_B\_cell\_down | Cd3g |
| GO:0046872 | metal ion binding | 6.69e-01 | 7.47e-01 | 10 | 47 | 3425 | 14853 | 21.28% | 23.06% | 0.29% | 0.32% | mature\_B\_cell\_down | Fyn, Gata3, Hpcal1, Ifih1, Itk, Notch1, Prkch, Prkcq, Rnf125, Zdhhc15 |
| GO:0004175 | endopeptidase activity | 6.82e-01 | 7.56e-01 | 1 | 47 | 357 | 14853 | 2.13% | 2.40% | 0.28% | 0.32% | mature\_B\_cell\_down | Ctsd |
| GO:0043169 | cation binding | 6.86e-01 | 7.57e-01 | 10 | 47 | 3467 | 14853 | 21.28% | 23.34% | 0.29% | 0.32% | mature\_B\_cell\_down | Fyn, Gata3, Hpcal1, Ifih1, Itk, Notch1, Prkch, Prkcq, Rnf125, Zdhhc15 |
| GO:0043167 | ion binding | 6.90e-01 | 7.58e-01 | 10 | 47 | 3479 | 14853 | 21.28% | 23.42% | 0.29% | 0.32% | mature\_B\_cell\_down | Fyn, Gata3, Hpcal1, Ifih1, Itk, Notch1, Prkch, Prkcq, Rnf125, Zdhhc15 |
| GO:0003723 | RNA binding | 7.05e-01 | 7.70e-01 | 2 | 47 | 765 | 14853 | 4.26% | 5.15% | 0.26% | 0.32% | mature\_B\_cell\_down | Hnrpll, Ifih1 |
| GO:0003676 | nucleic acid binding | 7.36e-01 | 7.99e-01 | 7 | 47 | 2594 | 14853 | 14.89% | 17.46% | 0.27% | 0.32% | mature\_B\_cell\_down | Btbd11, Gata3, Hnrpll, Ifih1, Notch1, Tcf7, Txk |
| GO:0016874 | ligase activity | 7.39e-01 | 7.99e-01 | 1 | 47 | 418 | 14853 | 2.13% | 2.81% | 0.24% | 0.32% | mature\_B\_cell\_down | Rnf125 |
| GO:0046983 | protein dimerization activity | 7.45e-01 | 8.01e-01 | 2 | 47 | 826 | 14853 | 4.26% | 5.56% | 0.24% | 0.32% | mature\_B\_cell\_down | Cd3g, Gata3 |
| GO:0046914 | transition metal ion binding | 7.60e-01 | 8.13e-01 | 4 | 47 | 1599 | 14853 | 8.51% | 10.77% | 0.25% | 0.32% | mature\_B\_cell\_down | Gata3, Ifih1, Rnf125, Zdhhc15 |
| GO:0060089 | molecular transducer activity | 7.76e-01 | 8.22e-01 | 6 | 47 | 2352 | 14853 | 12.77% | 15.84% | 0.26% | 0.32% | mature\_B\_cell\_down | Apol7b, Cd247, Cd3g, F2r, Lat, Tnfrsf1a |
| GO:0004871 | signal transducer activity | 7.76e-01 | 8.22e-01 | 6 | 47 | 2352 | 14853 | 12.77% | 15.84% | 0.26% | 0.32% | mature\_B\_cell\_down | Apol7b, Cd247, Cd3g, F2r, Lat, Tnfrsf1a |
| GO:0008324 | cation transmembrane transporter activity | 7.85e-01 | 8.28e-01 | 1 | 47 | 477 | 14853 | 2.13% | 3.21% | 0.21% | 0.32% | mature\_B\_cell\_down | Slc12a7 |
| GO:0008092 | cytoskeletal protein binding | 8.44e-01 | 8.86e-01 | 1 | 47 | 575 | 14853 | 2.13% | 3.87% | 0.17% | 0.32% | mature\_B\_cell\_down | Fyn |
| GO:0015075 | ion transmembrane transporter activity | 8.79e-01 | 9.18e-01 | 1 | 47 | 651 | 14853 | 2.13% | 4.38% | 0.15% | 0.32% | mature\_B\_cell\_down | Slc12a7 |
| GO:0004888 | transmembrane signaling receptor activity | 9.04e-01 | 9.39e-01 | 4 | 47 | 2046 | 14853 | 8.51% | 13.77% | 0.20% | 0.32% | mature\_B\_cell\_down | Cd247, Cd3g, F2r, Tnfrsf1a |
| GO:0022891 | substrate-specific transmembrane transporter activity | 9.14e-01 | 9.46e-01 | 1 | 47 | 756 | 14853 | 2.13% | 5.09% | 0.13% | 0.32% | mature\_B\_cell\_down | Slc12a7 |
| GO:0038023 | signaling receptor activity | 9.20e-01 | 9.47e-01 | 4 | 47 | 2128 | 14853 | 8.51% | 14.33% | 0.19% | 0.32% | mature\_B\_cell\_down | Cd247, Cd3g, F2r, Tnfrsf1a |
| GO:0042802 | identical protein binding | 9.33e-01 | 9.54e-01 | 1 | 47 | 829 | 14853 | 2.13% | 5.58% | 0.12% | 0.32% | mature\_B\_cell\_down | Cd247 |
| GO:0022857 | transmembrane transporter activity | 9.37e-01 | 9.54e-01 | 1 | 47 | 846 | 14853 | 2.13% | 5.70% | 0.12% | 0.32% | mature\_B\_cell\_down | Slc12a7 |
| GO:0022892 | substrate-specific transporter activity | 9.41e-01 | 9.54e-01 | 1 | 47 | 866 | 14853 | 2.13% | 5.83% | 0.12% | 0.32% | mature\_B\_cell\_down | Slc12a7 |
| GO:0005215 | transporter activity | 9.68e-01 | 9.77e-01 | 1 | 47 | 1044 | 14853 | 2.13% | 7.03% | 0.10% | 0.32% | mature\_B\_cell\_down | Slc12a7 |
| GO:0004930 | G-protein coupled receptor activity | 9.96e-01 | 1.00e+00 | 1 | 47 | 1618 | 14853 | 2.13% | 10.89% | 0.06% | 0.32% | mature\_B\_cell\_down | F2r |
| GO:0003674 | molecular\_function | 1.00e+00 | 1.00e+00 | 47 | 47 | 14853 | 14853 | 100.00% | 100.00% | 0.32% | 0.32% | mature\_B\_cell\_down | Apol7b, Art2b, Btbd11, Cd226, Cd247, Cd27, Cd3g, Cd7, Ctsd, Ctsw, Enpp4, F2r, Fyb, Fyn, Gata3, Hnrpll, Hpcal1, Ifih1, Ipcef1, Itk, Lat, Lck, Lpin1, Notch1, Ppm1h, Prkch, Prkcq, Rab37, Rinl, Rnf125, Selplg, Sept9, Sh2d2a, Skap1, Sla2, Slc12a7, Slc9a3r1, Stk39, Sytl2, Tbc1d2b, Tcf7, Thy1, Tnfrsf1a, Tnik, Txk, Zap70, Zdhhc15 |

### Unannotated IDs

**mature\_B\_cell\_down**  
NA

|  |  |  |
| --- | --- | --- |
| [close] | **Legend: Edge Types** | (details) |
|  | | |
