# Supplementary material for: Ontology based molecular signatures for immune cell types via gene expression analysis
Source: BMC Bioinformatics. 2013 Aug 30;14:263. doi: 10.1186/1471-2105-14-263 (PMC3844401; doi:10.1186/1471-2105-14-263)
Supplement: Additional file 1 — OBAMS profiles for all mature B cells. Additional file 1 contains a zip archive of OBAMS profiles for all mature B cells, including for each cell type individual spreadsheets showing up and down regulated genes for that cell type relative to parental cell types, and VLAD (GO term enrichment) results for all mature B cells. [file 1471-2105-14-263-S1.zip › Additional File 1/MZ B cell/VLAD.CL_0000845_down/results.html]

# CL\_0000845\_down

|  |  |
| --- | --- |
| Vlad version: | v1.5 |
| Date: | Mon Aug 29 17:27:52 2011 |
| Run time: | 29.08 sec |
| Ontology file: | gene\_ontology.obo |
| Ontology date: | Fri Aug 26 19:30:00 2011 |
| Annotation file: | gene\_association.mgi |
| Annotation date: | ??? |
| Analysis type: | enrichment |
| Excluded evidence codes: | ND |
| Number of query sets: | 1 |
| Query set 1: | CL\_0000845-down.xls (n=32; 137 not found) |
| Universe set: | default (everything) |
| Graph display: | Top 25 scoring terms and their ancestors. Interior nodes have been culled. |

**Jump to:** biological\_process | cellular\_component | molecular\_function | Unannotated id/symbols

### biological\_process (top)

  
  


### cellular\_component (top)

  
  


### molecular\_function (top)

  
  


### Unannotated IDs

**CL\_0000845-down.xls**  
0.112495137171619
0.202088804437045
0.21608260120999
0.277748395518206
0.289831489888
0.315743241206844
0.323863811947602
0.345404069352953
0.367103001309965
0.384277022418016
0.408479758589892
0.4177162420186
0.423488840826555
0.45714041004844
0.481597966701298
0.58042531777333
0.722766884935695
0.896213446637194
0.96164413353766
0.975740084458364
1.01703915134535
1.18865202156078
1.20916266123021
1.26219130546247
1.72350179034048
1.72622058239282
1.80701966367437
1.82625782105921
1.83336545307516
1.86379273276182
1.95411886496087
1.96436291942561
1.9801143888955
1.98056297815117
100604
10339667
10351825
10368289
10372844
10410099
10422822
10425370
10428857
10431410
10434758
10437590
10440186
10443598
10449527
10453272
10456492
10468980
10469322
10472820
10476252
10486396
10522160
10523766
10541260
10542981
10555510
10569707
10569890
10572456
10572635
10572786
10591517
10597477
106504
108909
11.6796762019049
11767
12193
12531
12581
13417
14.1111047803753
16403
16478
16880
18605
19094
192678
2.01957921658016
2.10407810188972
2.10907585455344
2.20019027349391
2.25595323859682
2.26175243459764
2.27446876221211
2.36087335420748
2.43604039982516
2.49847186452105
2.60010381360313
2.65439555741935
2.78895740393722
2.88957008762719
2.99647842337469
20440
207728
211401
21346
218294
22352
224273
239556
24.5539471043503
3.3047632885739
3.34381695775266
3.36945979236437
3.41246568137608
3.63511956013234
3.72170662444911
330409
333433
333789
4.48304611998562
4.54927943581177
4.72137836449013
4.82489124947022
5.42904646349773
5.85786328277039
50918
52502
52662
55948
63986
66540
8.80391896235471
8.8117533399027
98878
FoldChange
NA
Stdv
entrezIDs
mgiID
symbol

|  |  |  |
| --- | --- | --- |
| [close] | **Legend: Edge Types** | (details) |
|  | | |
