# Supplementary material for: Ontology based molecular signatures for immune cell types via gene expression analysis
Source: BMC Bioinformatics. 2013 Aug 30;14:263. doi: 10.1186/1471-2105-14-263 (PMC3844401; doi:10.1186/1471-2105-14-263)
Supplement: Additional file 1 — OBAMS profiles for all mature B cells. Additional file 1 contains a zip archive of OBAMS profiles for all mature B cells, including for each cell type individual spreadsheets showing up and down regulated genes for that cell type relative to parental cell types, and VLAD (GO term enrichment) results for all mature B cells. [file 1471-2105-14-263-S1.zip › Additional File 1/MZ B cell/VLAD.CL_0000845_up/results.html]

# CL\_0000845\_up

|  |  |
| --- | --- |
| Vlad version: | v1.5 |
| Date: | Mon Aug 29 17:28:48 2011 |
| Run time: | 31.17 sec |
| Ontology file: | gene\_ontology.obo |
| Ontology date: | Fri Aug 26 19:30:00 2011 |
| Annotation file: | gene\_association.mgi |
| Annotation date: | ??? |
| Analysis type: | enrichment |
| Excluded evidence codes: | ND |
| Number of query sets: | 1 |
| Query set 1: | CL\_0000845-up.xls (n=99; 468 not found) |
| Universe set: | default (everything) |
| Graph display: | Top 25 scoring terms and their ancestors. Interior nodes have been culled. |

**Jump to:** biological\_process | cellular\_component | molecular\_function | Unannotated id/symbols

### biological\_process (top)

  
  


### cellular\_component (top)

  
  


### molecular\_function (top)

  
  


### Unannotated IDs

**CL\_0000845-up.xls**  
0.10127163379043
0.139635820307088
0.16590059205788
0.16904087073757
0.173116185715971
0.193862162414025
0.197546961052175
0.19992894657595
0.204268679948156
0.217645489820859
0.219607506242668
0.222108283867843
0.225293833788756
0.232039161076612
0.240610350864513
0.253006626561541
0.25895810361619
0.26231308219986
0.26874784985115
0.286246234347663
0.289107187571278
0.294773528448974
0.295012323399149
0.297693486425373
0.313617671262204
0.31648880041429
0.322934078539537
0.329247463158483
0.358092483583862
0.363135254126506
0.369078320843711
0.37686881436473
0.381135307095034
0.382048354736069
0.388058466978227
0.391673716144923
0.394376571679529
0.396707170490503
0.400219194157458
0.406469229849149
0.407040154381882
0.435491961398309
0.440373427779208
0.44050961642698
0.456477447261436
0.466523448893432
0.506205293705117
0.540086172668991
0.582026935582155
0.620586557790013
0.632860029001492
0.637583446729924
0.651467425367383
0.677906501287555
0.686831910893044
0.687440064543505
0.701642051440678
0.732138617265871
0.806193171675328
0.821636339639886
0.851644482325385
0.874092900943871
0.878626720000365
0.896746756340618
0.92015614463651
0.947847554959381
0.996190288765407
0.998898808889577
0.999685116214861
1.01366362640824
1.04802705770901
1.08034712309339
1.1008428857186
1.13355388368274
1.14939476728082
1.14975893903802
1.16670767969315
1.1849216755102
1.19272565745689
1.22478027610776
1.25007962224872
1.31566779920901
1.34998408896913
1.35837708055529
1.4382105053666
1.45904473070489
1.47071016783644
1.48976926964358
1.4912818900736
1.54424832370219
1.56306174074712
1.56719532420875
1.57565268432083
1.69356543910703
1.71603897889141
1.72881214226253
1.79107660094874
1.79608445500213
1.83660466758709
1.90022125998808
1.91549043740102
1.92899856103934
1.94817985131886
1.95774581898135
1.9757679831236
1.98719092055589
1.99385607765451
10.0423437746003
10.161565916925
100038524
100040353
10340824
10348451
10352661
10352725
10353450
10354494
10355984
10361292
10363475
10368675
10369531
10369702
10370339
10371662
10372028
10382449
10384974
10388211
10394862
10397476
10402325
10405179
10406663
10406672
10407097
10407124
10407782
10415818
10417759
10421143
10421258
10422518
10428534
10428536
10429515
10429534
10434925
10435661
10435674
10437272
10441422
10445241
10445293
10445308
10445323
10445746
10447275
10456317
10463355
10466190
10466200
10467258
10468898
10469538
10480145
10480284
10480286
10485388
10486833
10495097
10495147
10495530
10497481
10498852
10499160
10500276
10500982
10501051
10503341
10504047
10507872
10508723
10509127
10517689
10519527
10521583
10521667
10524345
10528090
10528207
10529732
10531952
10532630
10533182
10535747
10542140
10542302
10543737
10543762
10544538
10547100
10548735
10554574
10559261
10559486
10561055
10562117
10562358
10571325
10571344
10573939
10578493
10581088
10584208
10584841
10586933
10586967
10587854
10588429
10590427
10590433
10592001
10593492
10593497
10596381
10601456
10608715
10608718
107769
109225
11.0193506260098
11.6603099223821
11542
116732
11881
12.0078121353347
12.4951484434785
12.8825683220833
12182
12479
12491
12520
12557
12778
12902
13492
13522
13610
14.1489196914306
142980
14357
15205
15476
16.9493695497637
16199
17118
171211
17936
17999
18646
18671
19.6192603056586
19143
19250
2.01167582074032
2.0503993063128
2.05874830142707
2.06420664971801
2.08429288128061
2.10062465395615
2.10365399232345
2.14811163420052
2.17652493734473
2.18337651592829
2.19666699343732
2.20100494331156
2.22405508245914
2.22423317772729
2.22825418461312
2.23756262202692
2.27472412384132
2.29083463073717
2.29575683016783
2.29950144526402
2.30552345877326
2.35896369143318
2.38195281399361
2.39254775800333
2.43467703492895
2.50814219521342
2.51186361987819
2.54357832173124
2.60498561715953
2.61794837825084
2.6184425687131
2.61872311638669
2.6734106170658
2.67369374144582
2.67629609470769
2.69226569506729
2.72362085957933
2.76033724121266
2.79035990263348
2.8482422200873
2.89185803620227
2.9303250633911
2.95331634839804
2.95835230017063
2.96097851651273
20163
20250
20720
20728
21.9912691440118
211914
212555
213019
217732
218793
22022
224454
226101
229600
232408
232430
233079
238871
23936
239845
240754
241041
241576
242819
244418
244871
26367
268752
268857
269344
27.1294548373851
270084
270152
27226
27405
28.8055648613994
28240
3.01506927131866
3.04267795059716
3.05212987152963
3.0807752343112
3.08555006099844
3.09580289139322
3.10034579230465
3.13864917777256
3.15165899630066
3.17517879843344
3.20227094693155
3.22043507417085
3.22454521137066
3.26205515032822
3.2858940910393
3.41739834956417
3.428956225264
3.43026563097793
3.44817294534046
3.4674771409454
3.48593232007313
3.64180577045694
3.6587161273214
3.66354968831271
3.68144251756537
3.68717072900792
3.75724242365189
3.77985728494463
3.81650071556218
3.83089137509837
3.89541943639193
3.91361695001225
3.95652603443331
320022
320129
328977
331004
381489
383435
4.2089744318221
4.23373406840474
4.29871266701645
4.44872546467033
4.49554070388799
4.53674198925989
4.54664409850168
4.71914494336939
4.88046005075463
433638
5.06961474113692
5.12636525495177
5.18537243678473
5.26281772332278
5.2745463294388
5.40386721077528
5.4074559256715
5.44556738965624
5.47732188852862
5.56620167610382
5.67115786738954
5.73138733924641
5.84015520970671
52065
52463
52855
54160
54712
56050
57810
58217
58222
59289
6.16374907004494
6.30432113463981
6.50728636238822
6.52616026605563
6.54654149795198
6.76459447928747
622976
625599
639396
65256
66950
67615
67784
69740
7.25514400939545
7.59584399946599
7.65317947809345
7.99700082052562
70423
70551
70686
71665
72121
73582
74103
74629
75669
77577
8.04425291926492
8.13640497487268
8.29686503042773
8.99915593050107
83925
9.2264652766889
9.32449721093675
94185
99712
FoldChange
Gm4956
MGI:1918846
MGI:2686122
MGI:3647976
Ms4a14
Ms4a7
NA
Stdv
entrezIDs
mgiID
symbol

|  |  |  |
| --- | --- | --- |
| [close] | **Legend: Edge Types** | (details) |
|  | | |
